# Identification and characterization of *Prunus persica* miRNAs in response to UVB radiation in greenhouse through high-throughput sequencing

**DOI:** 10.1186/s12864-017-4347-5

**Published:** 2017-12-02

**Authors:** Shaoxuan Li, Zhanru Shao, Xiling Fu, Wei Xiao, Ling Li, Ming Chen, Mingyue Sun, Dongmei Li, Dongsheng Gao

**Affiliations:** 1 College of Horticulture Science and Engineering, Shandong Agricultural University, Tai’an, 271018 People’s Republic of China; 2 State Key Laboratory of Crop Biology, Shandong Agricultural University, Tai’an, 271018 People’s Republic of China; 3 Key Laboratory of Experimental Marine Biology, Institute of Oceanology, Chinese Academy of Sciences, Qingdao, 266071 People’s Republic of China; 4 Laboratory for Marine Biology and Biotechnology, Qingdao National Laboratory for Marine Science and Technology, Qingdao, 266237 People’s Republic of China

**Keywords:** *Prunus persica*, UVB, MicroRNA, High-throughput sequencing, Chlorophyll and carbohydrates

## Abstract

**Background:**

MicroRNAs (miRNAs) are small non-coding RNAs that regulate gene expression of target mRNAs involved in plant growth, development, and abiotic stress. As one of the most important model plants, peach (*Prunus persica*) has high agricultural significance and nutritional values. It is well adapted to be cultivated in greenhouse in which some auxiliary conditions like temperature, humidity, and UVB etc. are needed to ensure the fruit quality. However, little is known about the genomic information of *P. persica* under UVB supplement. Transcriptome and expression profiling data for this species are therefore important resources to better understand the biological mechanism of seed development, formation and plant adaptation to environmental change. Using a high-throughput miRNA sequencing, followed by qRT-PCR tests and physiological properties determination, we identified the responsive-miRNAs under low-dose UVB treatment and described the expression pattern and putative function of related miRNAs and target genes in chlorophyll and carbohydrate metabolism.

**Results:**

A total of 164 known peach miRNAs belonging to 59 miRNA families and 109 putative novel miRNAs were identified. Some of these miRNAs were highly conserved in at least four other plant species. In total, 1794 and 1983 target genes for known and novel miRNAs were predicted, respectively. The differential expression profiles of miRNAs between the control and UVB-supplement group showed that UVB-responsive miRNAs were mainly involved in carbohydrate metabolism and signal transduction. UVB supplement stimulated peach to synthesize more chlorophyll and sugars, which was verified by qRT-PCR tests of related target genes and metabolites’ content measurement.

**Conclusion:**

The high-throughput sequencing data provided the most comprehensive miRNAs resource available for peach study. Our results identified a series of differentially expressed miRNAs/target genes that were predicted to be low-dose UVB-responsive. The correlation between transcriptional profiles and metabolites contents in UVB supplement groups gave novel clues for the regulatory mechanism of miRNAs in *Prunus*. Low-dose UVB supplement could increase the chlorophyll and sugar (sorbitol) contents via miRNA-target genes and therefore improve the fruit quality in protected cultivation of peaches.

**Electronic supplementary material:**

The online version of this article (doi: 10.1186/s12864-017-4347-5) contains supplementary material, which is available to authorized users.

## Background

As an important environment signal, sunlight provides energy for the growth and development of plants [1], but its ultraviolet (UV) radiation part causes abiotic stress potentially influence the biological processes of plants. Since the late 1980s when awareness of stratospheric ozone layer depletion triggered concerns about the potentially harmful effects of increased UVB radiation, many studies have shown that UVB causes non-specific damage to DNA, proteins and lipids [2–4]. On the other hand, there is overwhelming evidence that other than substantially impeding plant growth, low-dose UVB is an environmental regulator affecting gene expression, cellular and metabolic activities, and growth and development [5–8]. Whether UVB radiation is a stressor or a regulator is determined by the fluence rate and exposure time [4]. Nevertheless, the regulatory mechanism of plants responding to the UVB-lack environment, for example in the greenhouse where the UVB radiation level is 30%–70% lower than outdoors, were rarely reported [9].

Most of the photomorphogenic responses to low-dose UVB are mediated by the photoreceptor UV RESISTANCE LOCUS8 (UVR8). Subsequent structural and functional characterization revealed that the UVR8 has a unique regulatory mechanism in photoreception [10–12]. After UVB treatment, UVR8 interacts with the E3 ubiquitin ligase (transducin/WD40 repeat-like superfamily protein) CONSTITUTIVELY PHOTOMORPHOGENIC1 (COP1), following the ubiquitination of the basic leucine-zipper (bZIP family) transcription factor ELONGATED HYPOCOTYL5 (HY5) which is primarily in the initiation of photo-morphogenesis [13–16].

MiRNAs, small endogenous non-coding RNAs approximately 21–24 nucleotides (nt) in length, play an important role in regulating gene expression at the post-transcriptional level [17–19]. A large number of miRNAs have been recently identified in plants via high-throughput sequencing, and numerous miRNAs have been entered into the miRBase 21. MiRNAs are involved in regulating growth, development, root initiation and development, hormone balance, floral morphogenesis and reproductive performance [20, 21]. Stress-regulated miRNAs in plants confer resistance to the extreme conditions, including UVB, drought, salt, cold and heat. In addition, the expression of miRNAs can alter the behavior of plants in response to both abiotic and biotic stresses [21, 22]. Previous reports have shown that miRNA induction was involved in regulating auxin signaling via miR160, miR167 and miR393 thus, becoming an important strategy for photomorphism in plants [23].

China is the largest producer of both outside-grown and inside-grown peaches and nectarines in the world. There are nearly 16,000 ha of protected peach and nectarines cultivation, 2.3% of the total area [24]. As a new agricultural form, protected production has been rapidly developed. Peach (*Prunus persica*), which has been cultivated for more than 4000 years, is one of the most important fruits in the world [25]. Peach has a small genome and it reaches reproductive maturity in a relatively short time. In 2010, the Genome Sequencing Project of the peach double haploid cultivar ‘Lovell’ was completed, which generated*,* 230 Mb genome sequence and 202 assembly scaffolds [26]. Therefore, peach is considered to be a useful forest model species for genetic and ecological research. Following these findings, several reports on the identification of miRNAs in different peach tissues have been published [27–29]. Meanwhile, peach has some unique biological features not commonly found in other model organisms, such as a 3–5 year juvenile period before blossom, the formation of fleshy fruit with a hardened endocarp and chilling-requirement dormancy mechanism [30–34]. Nectarine, because of its low-chill and nutritional value, is selected as one of the most important fruits in the protected cultivation industry which targeted early and high markets. In previous research, we found that the supplement of UVB radiation can improve the fruit quality and the ability to compete for C-assimilate [35, 36]. Considering the distinct environment especially the light condition in greenhouse, more deep studies related to the molecular and metabolic mechanism under UVB irradiance are needed.

In this study, we generated over 2 billion bases of high-quality RNA sequence with Illumina platform. In a single run, we identified 31,763,592 raw sequences including thousands of seed target and metabolism genes. Our results identified a series of differentially expressed miRNAs/target genes that were predicted to be low-dose UVB-responsive. The correlation between transcriptional profiles and metabolites’ contents helped elucidate the regulatory mechanisms of peach under the UVB supplement. Our findings of correlation among miRNA, target genes and metabolites provided clues for breeding with high-quality fruit and other properties which are suitable for greenhouse cultivation.

## Results

### Analysis of miRNA sequences

Using Illumina sequencing, a total of 74,119,581 and 76,123,247 raw reads were obtained from control and UVB-treatment groups, respectively (Table 1). After discarding 3′ adapter deletions, insertion deletions, 5′ adapter contaminants, poly-A sequences and sequences less than 18 nt from the high-quality reads, 52,375,087 and 56,244,851 clean reads were used for further analysis. The proportions of clean reads were 70.79% and 73.85% of the total reads obtained from the two libraries, respectively.

**Table 1:** The quality control of the clean data

Sample	Num. of Reads	Raw Clean Reads %	Remove Adapter%	Insert Null %	N %	Too short %	Poly-A %	Too long %	Low quality %
CK1	23,619,871	76.50%	0.06%	2.18%	0.17%	13.86%	0.06%	7.04%	0.12%
CK2	25,100,163	68.65%	0.09%	1.27%	0.16%	24.63%	0.05%	5.05%	0.11%
CK3	25,399,547	67.22%	0.09%	1.34%	0.16%	29.78%	0.04%	1.25%	0.11%
T1	25,154,335	74.96%	0.07%	2.16%	0.17%	15.86%	0.06%	6.60%	0.11%
T2	26,168,148	75.67%	0.10%	0.65%	0.18%	14.36%	0.05%	8.88%	0.11%
T3	24,800,764	70.92%	0.11%	0.64%	0.16%	21.37%	0.05%	6.65%	0.11%

*CK* control groups without UVB treatment, *T* UVB treatment groups

N% means the percentage of loci that fail to distinguish specific bases

**Fig. 1:**
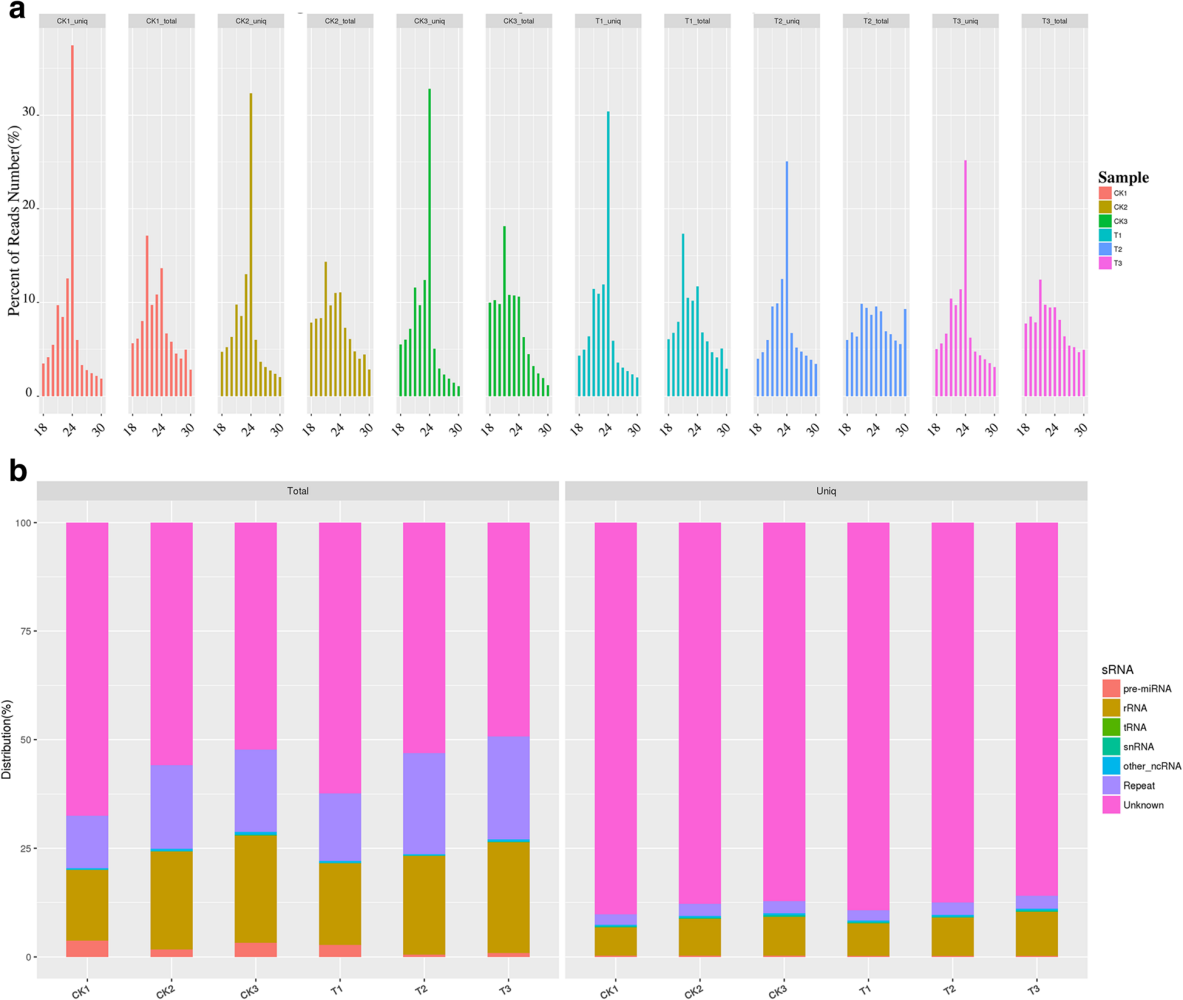
The analysis of sRNA between the CK and UVB-treated groups. **a** Length distribution of sRNAs. The length distribution of high-quality obtained from the UVB and CK libraries. The distributions of the total reads were shown as percentages. **b** The sRNA classification of the total and unique sequences between control and treatment libraries. Total sRNAs and unique sRNAs are shown in the left and the right panel, respectively

The small RNA (sRNA) reads were typically 18 to 30 nt in length (Fig. 1). Among these sequences, 21 nt sRNAs were the most abundant in the two libraries, accounting for 16.56% and 13.22% of the total reads, followed by 24 nt sRNAs, which accounted for 11.79% and 10.26% of the total reads, respectively. Furthermore, we observed that the number of less than 24 nt length sequences in the control libraries was more abundant than that in the treatment libraries (74.08% and 64.14%, respectively). Additionally, a large proportion of unique sequences (>85% in both libraries) were unclassified sRNAs, suggesting a broad existence of miRNAs in peach.

### Known miRNAs in peach

Known miRNAs in peach affected by UVB were identified through homologous alignment analysis using the plant miRNA in miRBase 21. A total of 164 known miRNAs belonging to 59 families were obtained from the deep sequencing. The dominant miRNA families are shown in Additional file 1: Table S1, and most of these miRNAs were largely conserved in various plant species. The expression levels of a few miRNA families, such as miR166, miR1511 and miR398, were evidentially high in both libraries. Some conserved miRNAs were reported only in few species, such as miR3627 in five (*Vitis vinifera*, *Populus trichocarpa*, *Malus domestica*, *Solanum tuberosum*, and *P. persica*), miR1511 in three (*M. domestica*, *S. tuberosum*, and *P. persica*), while miR8133 only in *P. persica.*

MiRNAs have a broad range of expression levels, varying from several to millions of reads. Most of the conserved miRNAs were identified from the two libraries, and certain miRNAs were abundant in some samples but scarce or even lacking in other samples. For example, the expression of miR159 generated 323,238 and 128,812 reads in the CK and UVB libraries respectively. Moreover, the number of reads for different members of the same family varied widely. For example, the expression of miR7122a in both libraries generated 21,291 reads while only 417 reads of miR7122b in both libraries.

### Novel miRNAs in peach

In the present study, 109 novel miRNAs from peach deep sequencing were identified (Additional file 2: Table S2). The most abundant miRNA was Pp04_27840-3p, with 33,630 and 42,292 reads in the UVB libraries and CK libraries, respectively. Many novel miRNAs from our database were conserved with miRNAs from other species to a certain degree (<50%), such as Pp02_15663-5p and Pp03–22,312-3p corresponding to miR172d and miR2950-5p in grape (*Vitis vinifera*), respectively.

### Differential expression of miRNA

**Fig. 2:**
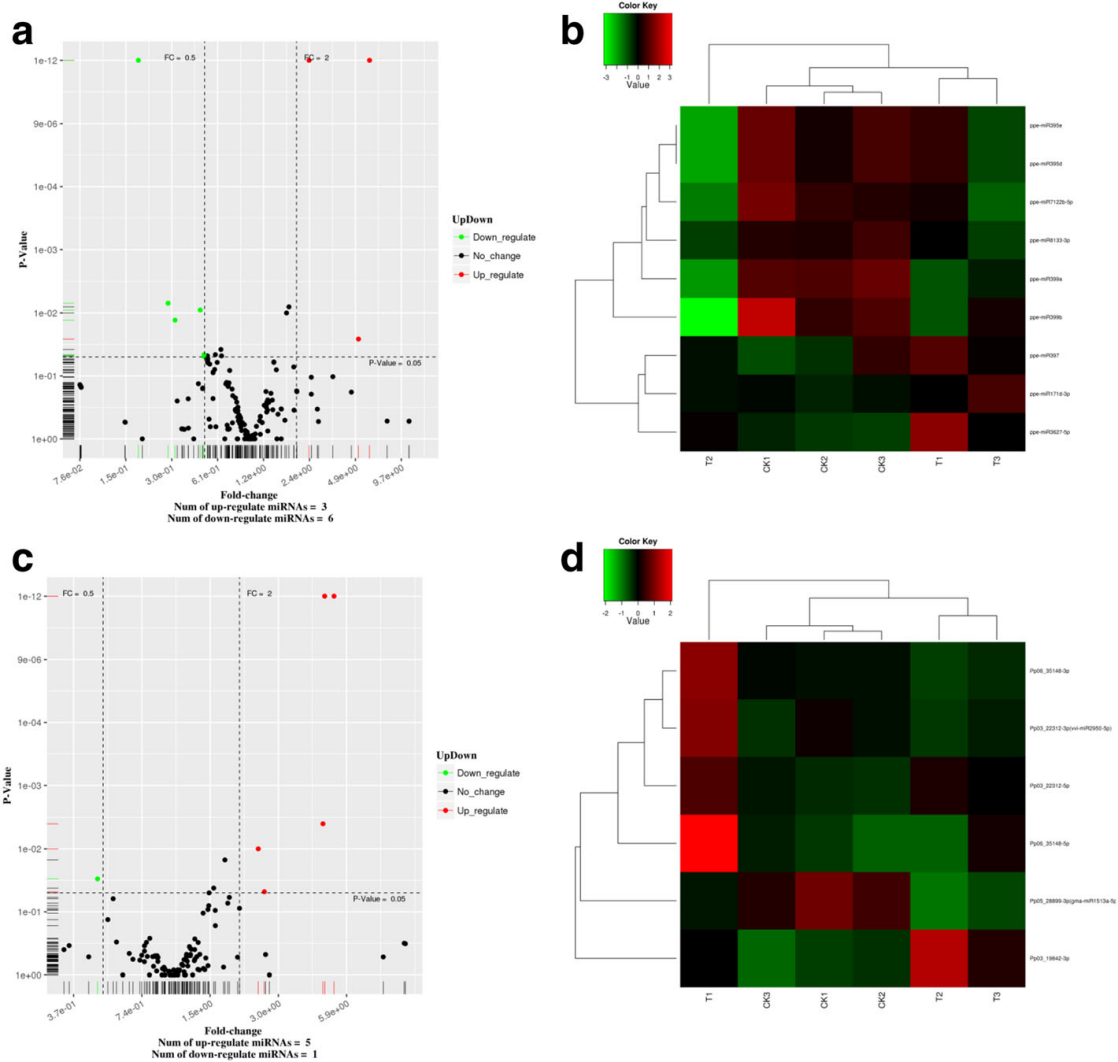
The differentially expressed miRNAs between the CK and UVB-treated groups. **a** Scatter diagram of the differential read counts of known miRNAs. Each point in the figure represents a miRNA. **b** Heat map of differentially expressed known miRNAs between the control and UVB-treated groups. **c** Scatter diagram of the differential read counts of novel miRNAs. Each point in the figure represents a miRNA. **d** Heat map of differentially expressed novel miRNAs between the control and UVB-treated groups. Red points represent miRNAs showing a > 2-fold change of expression; green points represent miRNAs showing 1/2 < fold change ≤ 2; black points represent miRNAs showing a fold change ≤ 1/2

The comparison of the miRNA expression levels in CK and UVB groups showed that 164 known and 109 novel miRNAs were identified in the two libraries. The analysis of the differential expression of miRNAs in the UVB treatment and control libraries showed that 9 known and 6 novel differentially expressed miRNAs from the two libraries might play important roles in the UVB response(Fig. 2). In brief, 8 miRNAs were up-regulated, including Pp03–22,312-3p, Pp03–22,312-5p, Pp05–19,842-3p, Pp06–35,148-3p, Pp06–35,148-5p, miR397, miRNA171d-3p and miRNA3627-5p, and 7 miRNAs were down-regulated, including miRNA395d, miRNA395e, miRNA7122b-5p, miRNA399a, miRNA399b, miRNA8133-3p, and Pp05–28,899-3p in UVB treatment.

### Target prediction and functional analysis

**Fig. 3:**
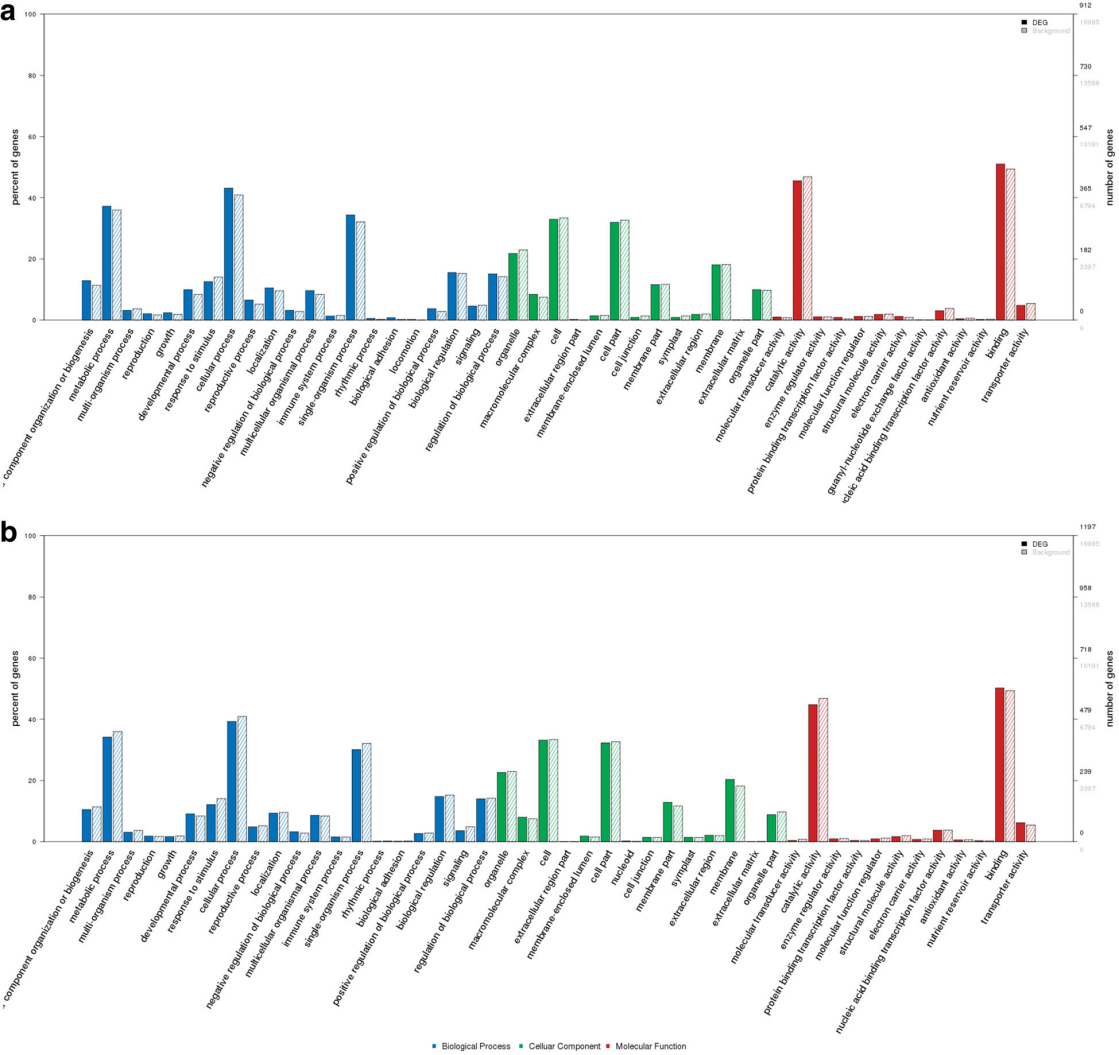
Gene ontology classification of potential target genes for differentially expressed miRNAs. The x-axis indicates the category name of GO annotation. The right y-axis indicates the number of unigenes in a category. The left y-axis indicates the percentage of a specific category of unigenes in that main category. The solid bars show the target genes of differentially expression miRNAs, while the hollow bars show genes from the 3’UTR (or mRNA) database. **a** Gene ontology classification of the known differential miRNA target genes. **b** Gene ontology classification of the novel differential miRNA target genes. Blue, green and red represent three GO ontologies: cellular component, molecular function and biological progress, respectively

**Fig. 4:**
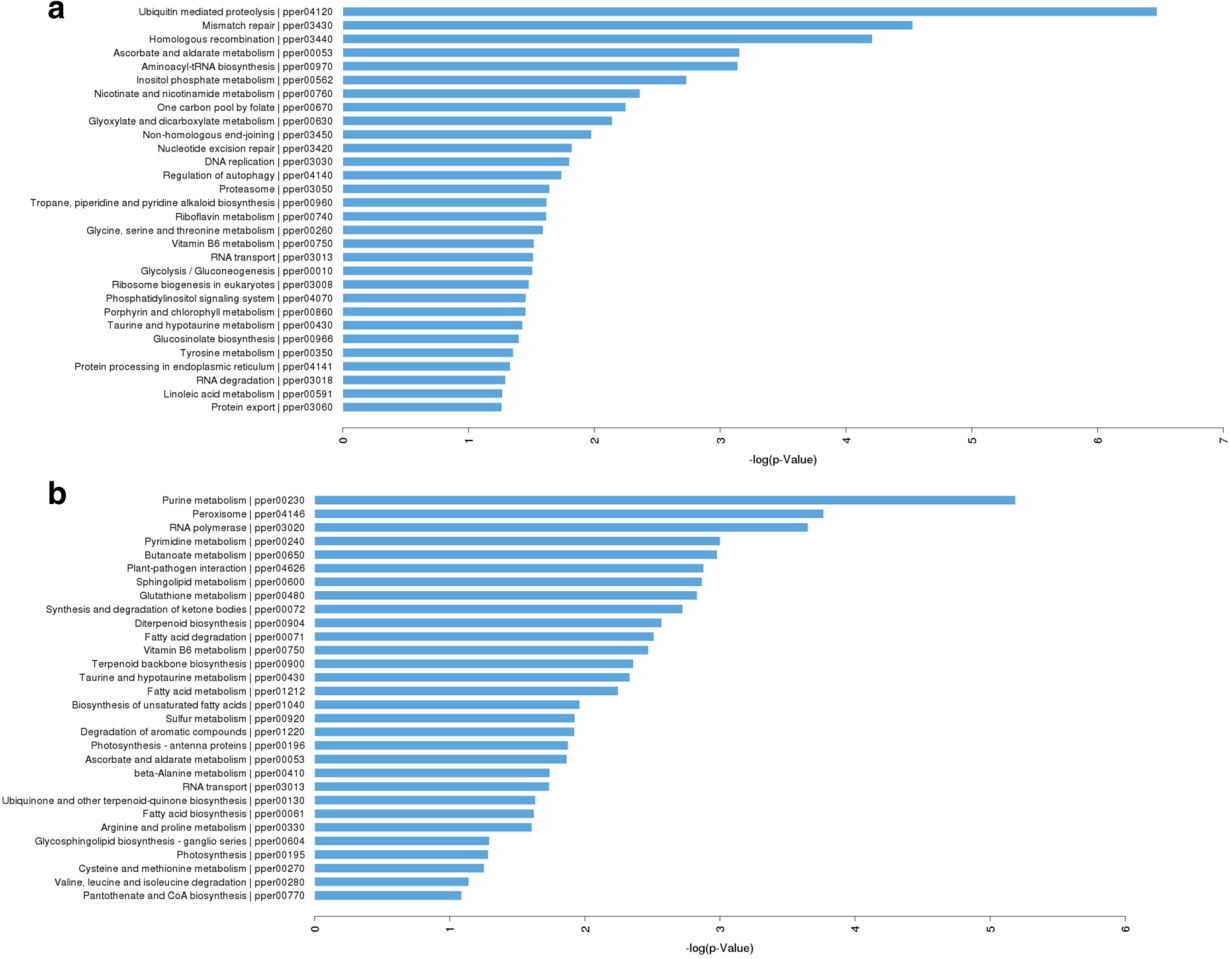
The KEGG analysis of the target genes for differentially expressed miRNAs. **a** Targets of known miRNA. **b** Targets of novel miRNAs

A large number of targets were predicted for most differentially expressed miRNAs. As for the known miRNAs, there were 1928 pathways that accounted for the largest percentage of the total targets. The results of Gene ontology classification and top 30 preferential KEGG pathway analysis revealed that target genes of these miRNAs were involved in various biological and biochemical processes in plant growth and development (Figs. 3 and 4) (Additional file 3: Table S3, Additional file 4: Table S4), such as porphyrin and chlorophyll metabolism (pper00860), pentose and glucuronate interconversions (pper00040), citrate cycle (TCA cycle, pper04712 and pper00020), circadian rhythm - plant and metabolic pathways (pper01100).

### Expression validation of UVB-responsive miRNA

**Fig. 5:**
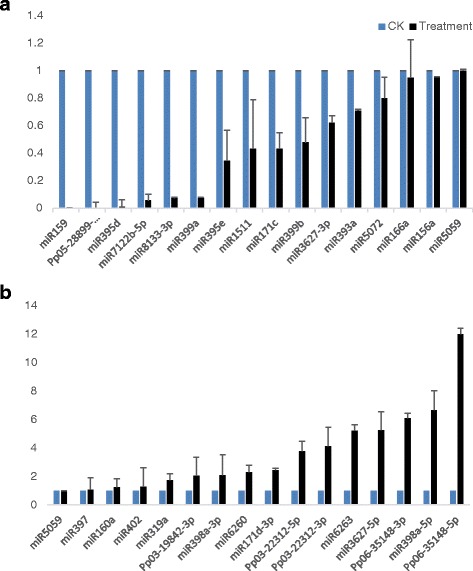
Relative expression levels of the selective UVB-responsive miRNAs by qRT-PCR analysis. **a** Down-regulation. **b** Up-regulation

Totally, we verified the expression of 31 miRNAs (Table 2) via qRT-PCR in which 15 miRNAs were retrieved in the database (Fig. 5). We selected miR5059 as reference miRNA [37] and 15 other miRNAs that related to UVB irradiance. The primers were listed in Additional file 5: Table S5. The qRT-PCR results showed the consistency with sequencing data except that miR397 levels under UVB treatment did not have significant difference with CK. The results showed some difference with the database. Many miRNAs were up-regulated other than down-regulation from high-throughput sequencing, such as miR398a-5p (6.67-fold), miR398a-3p (2.09-fold), miR6263 (5.21-fold), miR6260 (2.31-fold), and miR319a (1.75-fold). On the contrary, miR1511 (2.31-fold), miR171c (2.31-fold), and miR3627-3p (1.61-fold) were down-regulated under UVB treatment (Table 2; Fig. 5). Some conserved UVB-responsive miRNAs were not remarkably expressed such as miR156a, miR160a, miR166a, miR393a, miR402a which were considered to be involved in plant acclimation and adaptation of biotic or abiotic stress.

**Table 2:** The comparison of fold change (from sequencing libraries) and expression levels (via qRT-PCR) of typical miRNAs

	MiRNA	Source	Target	Annotation	Sequencing(Fold-change)	qRT-PCR(Fold-change)
Down-regulation	miR159	Ptc [27],Ath,Pte [22]	MYB33	leaf development	–	–
	Pp05–28,899-3p	Sequencing			0.47	–
	miR395d	Sequencing, Ath [22],Ptc [27]	APS,AST68	sulfate translocation and assimilation.	0.5	80.75
	miR7122b-5p	Sequencing	HOS		0.47	17.55
	miR8133-3p	Sequencing			0.32	13.93
	miR399a	Sequencing, Ath [22],Ptc [27]	UBC24		0.18	13.18
	miR395e	Sequencing, Ath [22],Ptc [27]	APS,AST68	sulfate translocation and assimilation.	0.5	2.89
	miR1511	Ppe [37]		unique	–	2.31
	miR171c	Ath [52]	SCL	chlorophyll synthesis	–	2.31
	miR399b	Sequencing			0.29	2.09
	miR3627-3p	Ppe [58]		TCA, EMP	–	1.61
	miR393a	Ath,Pte [22]	AFB2,TIR1, SCF	Antibacterial Resistance, abiotic stress tolerance	–	1.41
	miR5072	Ppe [37]		alternative reference	–	1.25
	miR166a	Ath,Pte [22]	HD-ZIP	abiotic stress tolerance	–	1.05
	miR156a	Ath,Pte [22]	SPL	abiotic stress tolerance	–	1.05
Reference	miR5059*	Ppe [37]		reference*	–	1
Up-regulation	miR397	Sequencing	Laccase		2.42	1.07
	miR160a	Ath,Pte [22]	ARF17	Leaf development	–	1.24
	miR402	Ppe [37]		abiotic stress tolerance	–	1.28
	miR319a	Ath [21]		flowing time	–	1.75
	Pp03–19,842-3p	Sequencing			5.23	2.04
	miR398a-3p	Ath,Pte [22]	CSD1,2	protection from oxidative stress	–	2.09
	miR6260	Ppe [37]		unique	–	2.31
	miR171d-3p	Sequencing			5.1	2.43
	Pp03–22,312-5p	Sequencing			–	3.77
	Pp03–22,312-3p	Sequencing			2.42	4.15
	miR6263	Ppe [37]		unique	4.67	5.21
	miR3627-5p	Sequencing			6.02	5.24
	Pp06–35,148-3p	Sequencing			2.57	6.09
	miR398a-5p	Ath,Pte [22]	CSD1,2	protection from oxidative stress	–	6.67
	Pp06–35,148-5p	Sequencing			4.75	11.99

Abbreviations: *Ath Arabidopsis thaliana*, *Ptc Populus trichocarpa*, *Pte Populus tremula*, *Ppe Prunus persica*

### Chlorophyll metabolism under UVB treatment

**Fig. 6:**
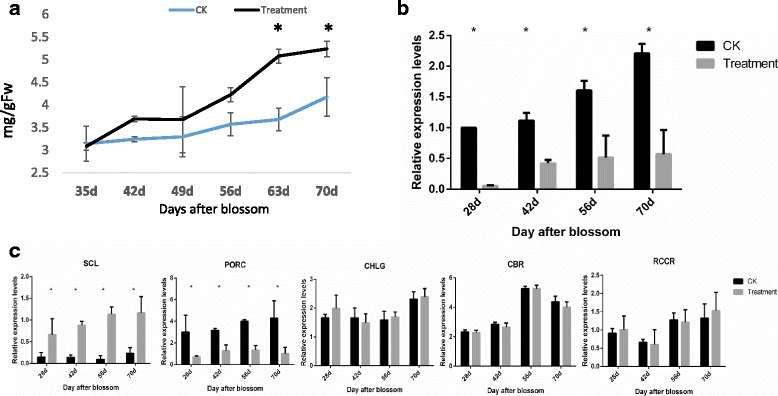
The chlorophyll metabolism under UVB supplement. **a** Chlorophyll content in the development period. **b** qRT-PCR analysis of miR171c in different stage. **c** qRT-PCR analysis of *SCL*, *PORC*, *CBR*, *RCCR*, and *CHLG* transcriptional levels using leaves of different developmental stages until fruit mature period

KEGG pathway analysis of miRNA target genes (Fig. 4) showed that genes involved in chlorophyll metabolism might be significantly regulated by UVB. We determined chlorophyll content and crucial miRNA/genes expression levels to give clues for UVB effects on chlorophyll metabolism. Spectrophotometry results showed that the content of chlorophyll was gradually increased during the whole development period (Fig. 6a). Its content reached up to 5.24 mg/g Fw in the mature stage of UVB-group, higher than 4.17 mg/g Fw of the control group. The expression levels of miR171c in UVB-group were lower than the untreated samples, which was 2.3-fold difference in the mature stage (Fig. 6b). Fig. 6c showed the different expression profiles of genes involved in chlorophyll metabolism. Scarecrow-like protein (*SCL*), the target gene of miR171c, was highly up-regulated during the developmental period. On the contrary, pchlide oxidoreductase C (*PORC*), the downstream gene of *SCL* was remarkably down-regulated. The others did not show significant difference between CK and UVB groups. Specifically, *SCL* was up-regulated by 2.2-fold after UVB treatment in fruit mature phase. *PORC* was down-regulated by 2.8-fold (Fig. 6c). The gene information and sequences of primers were listed in Additional file 6: Table S6.

### Carbohydrate metabolism under UVB treatment

**Fig. 7:**
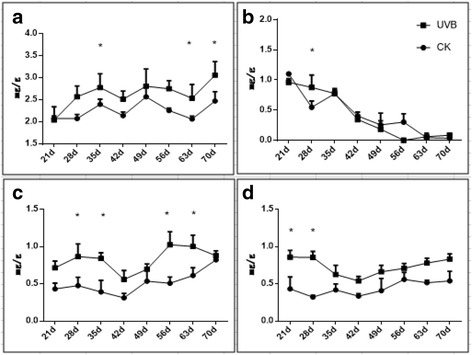
Comparison of the contents of carbohydrates in leaves at mature stage in CK and UVB group. **a** Sorbitol. **b** Sucrose. **c** Glucose. **d** Fructose

Carbohydrate metabolism was another pathway targeted by miRNAs predicted by KEGG analysis. The contents of carbohydrates in leaves were significantly affected by UVB treatment (Fig. 7). Sorbitol, the main form of sugar, was up-regulated by 1.24-fold with UVB stimulation in the mature period (Fig. 7a), while the content of sucrose was dramatically decreased and did not show significant difference with/without UVB treatment (Fig. 7b). Fructose and glucose contents were increased with the developmental process, and the UVB-groups were higher than the control samples (Fig. 7c and d).

**Fig. 8:**
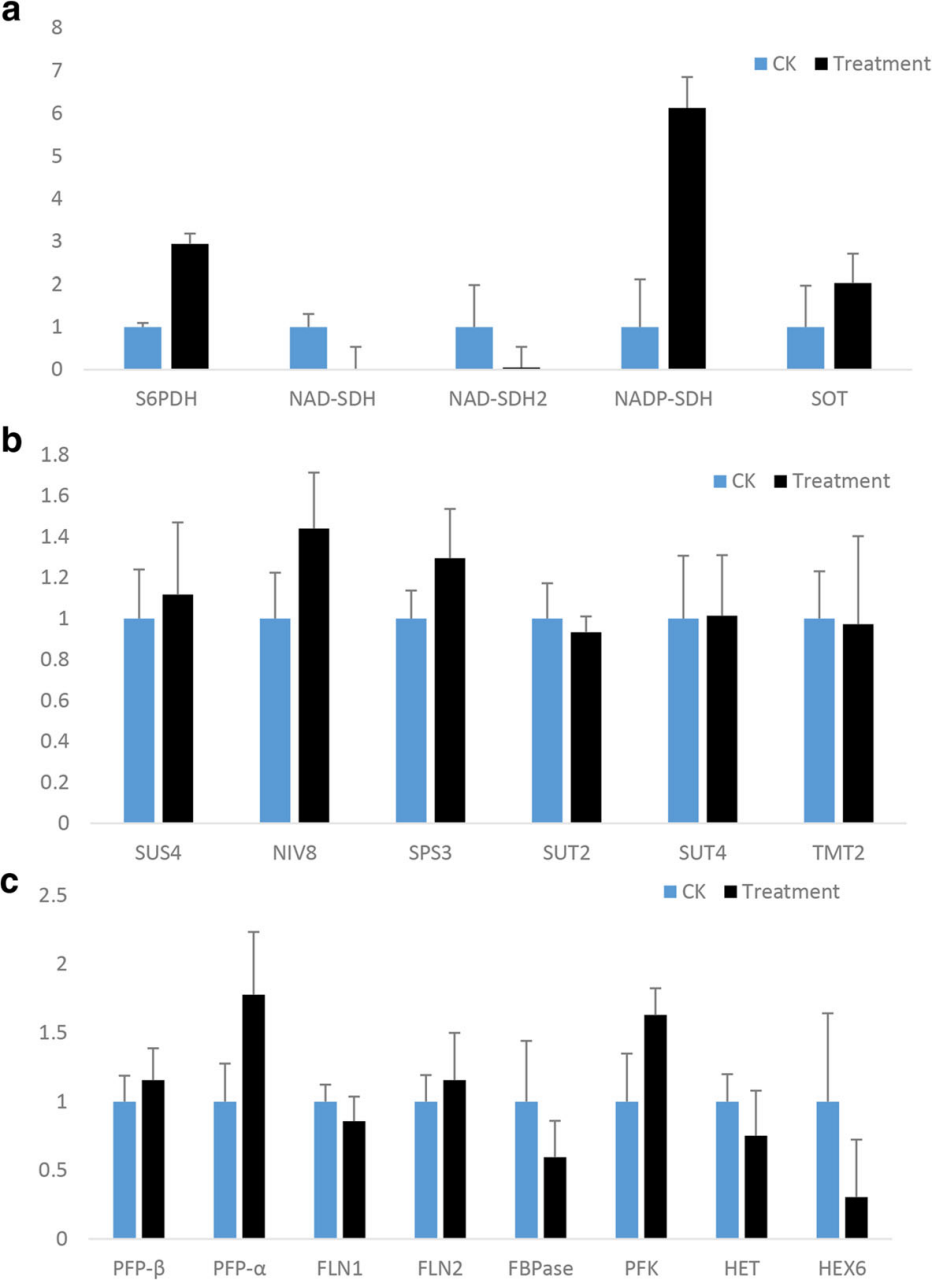
qRT-PCR analysis of key genes which related to sugar metabolism. **a** *S6PDH, NAD-SDH, NAD-SDH2, NADP-SDH*, and *SOT* which are related to sorbitol metabolism. **b** *SUS4, NIV8, SPS3, SUT2, SUT4*, and *TMT2* which related to sucrose metabolism. **c** *PFP-β*, *PFP-α*, *FLN1*, *FLN2*, *FBpase*, *PFK*, *HET*, and *HEX6* which related to Hexose metabolism

We screened 19 genes related with sugar metabolism from *P. persica* genome, and applied qRT-PCR assay to investigate the correlation between sugar synthesis and miRNA under UVB treatment (Fig. 8). The crucial genes involved in sucrose pathway did not show significant difference between UVB and control groups (Fig. 8a). However, genes in sorbitol metabolism were expressed distinctly after UVB stimulation (Fig. 8b). The expression levels of Sorbitol −3-orbitol −6-phosphate dehydrogenase (*S6PDH*), NADP dependent sorbitol dehydrogenase (*NADP-SDH*) and Sorbitol transporter (*SOT*) were increased by 2.9-fold, 6.1-fold and 2.0-fold respectively under UVB treatment compared to the control groups. However, NAD dependent sorbitol dehydrogenase (*NAD-SDH*) was rarely expressed under UVB treatment. Moreover, the expression levels of Pyrophosphate--fructose 6-phosphate 1-phosphotransferase subunit alpha (*PFP-α*) and Phosphofructokinase (*PFK*) in hexose pathway were1.8-fold and 1.6-fold higher in UVB group than those in control group (Fig. 8c). Hexose carrier protein (*HEX6*) showed a 3.2-fold decrease after UVB stimulation (Fig. 8c). These results indicated that UVB had a major influence on the interconversion and transportation of monosaccharides. The gene information and sequences of primers were listed in Additional file 6: Table S6.

## Discussion

There are many problems in the protected cultivation of peach trees, such as the basic theory and growth/development pattern, the environmental intelligent control, the standardization system with high quality and efficiency etc. [38]. It is mainly ascribed to the weak light irradiance and quality, and the quality of fruits in the greenhouse is worse than that in the outdoor cultivation [39]. Our investigation on the regulatory mechanism of complementary UVB will provide significant effects for elucidating the growth and development pattern of *P. persica*, thus increasing the fruit quality.

High-dose UVB may produce phenols, ROS and cause damage to DNA, proteins, and membranes [40–42]. However, low-dose UVB may trigger early adaptation to environment and regulate plants seeding, development and growth such as the induction of alterations in antioxidant status, the regulation of phenylpropanoids, cinnamates, or flavonoids pathways, chlorophyll and pyridoxine biosynthesis pathways [43]. In our previous study, Chen et al. selected three UVB radiation levels and 1.44 Kj m^−2^ d^−1^ showed the most effective function on the improvement of total soluble solid, anthocyanin and the repression of total acid in peach cultivated in greenhouse [44]. Therefore the UVB radiation level (1.44 Kj m^−2^ d^−1^) was applied in this study as the most suitable dose to regulate the growth and fruit quality of *P. persica* in the greenhouse environment.

MiRNAs play important roles in plant response and adaptation to environmental change. Thus, understanding the miRNA-mediated regulatory network of UVB supplement will lay the foundation for unraveling the complex molecular genetic mechanism of positive effects on fruit’s agronomic traits improvement. A growing evidences suggested that miRNA-guided gene regulation could play a vital role in plant response to UVB radiation [42, 45, 46]. In the present study, a total of 4.02 M and 3.83 M unique sRNA sequences were obtained from the control and UVB-treatment libraries, respectively, suggesting adequate sequencing depth for further analysis. The majority of total sRNA reads ranged from 18 nt to 30 nt in length (Fig. 1), which was consistent with the typical size for Dicer-derived products [47]. The most abundant length is 21 nt followed by 24 nt which was consistent with previous studies in peach [48–51].

In our study, the expression levels of 2 known miRNAs were highly up-regulated (miR171d-3p, miR3627-5p) and 6 known miRNAs showed significant down-regulation (miR395d, miR395e, miR399a, miR399b, miR7122b-5p, miR8133-3p). These miRNAs were predicted to be involved in distinct metabolic pathways.

MiR171c was predicted to target *SCL6, SCL22* and *SCL27*, a family of transcription factors which were involved in the morphogenesis, proliferation of meristematic cells, polar organization and chlorophyll synthesis [52–54]. Further study found that its target gene tomato (S*olamum lycopersicum*) gras transcription factor gene (*SlGRAS24*) impacts multiple agronomical traits, such as plant height, flowering time, leaf architecture, lateral branch number, root length, fruit set and development, via regulating gibberellin and auxin homeostasis [55, 56]. It is indicated that the UVB-responsive miRNA171 plays an important role in regulating the growth and development of plant. In the present study, we investigated the potential relation between miR171c and chlorophyll synthesis. The content of chlorophyll was gradually increased during the whole development period under UVB treatment, which was in accordance with the studies by Chen [44]. Although previous reports showed that miRNA171 was up-regulated under light [57], our results found that miRNA171c was less expressed after UVB stimulation. Further analysis on genes involved in miR171-SCL model showed that *SCL* and *PORC* were up-regulated and down-regulated respectively, which was in accordance with this model. *PORC* had positive effects as an upstream gene of chlorophyll synthetic pathway. However, our results showed an opposite regulation pattern between *PORC* and chlorophyll content. Interestingly, the expression levels of Chlorophyll a-b binding protein (*CBR*) and Red chlorophyll catabolite reductase (*PCCR*) (genes involved in chlorophyll degradation) had no change after UVB treatment, while the *CHLG* was up-regulated by 1.6-fold. According to these results, we presumed that the increase of chlorophyll content under UVB might be not only related with miR171-SCL model but also regulated by non miR171-SCL pathway like the regulation of CHLG. Further verification is needed to illustrate the regulatory mechanism of chlorophyll metabolism under UVB.

A conserved miRNA family (miR3627) was reported only in five species (*Solanum tuberosum*, *Malus domestica*, *Populus trichocarpa*, *Citrus trifolia*, and *Prunus persica*), and was identified as a chilling responsive miRNA in *P. persica* [58]. In our study, miRNA3627-5p was up-regulated under UVB treatment, and we found an interesting physiological phenomenon that the germination rate of treatment group (89.2%) was 12% higher than the CK group (77.5%), which occurred 8 months after the termination of UVB treatment. It implied that UVB radiation may have a long-term and sustainable influence on plants via modification called “UVB memory” [59], and this phenomenon involved a series of miRNAs such as miR3627-5p. Bioinformatics analysis of miR3627-5p in our libraries showed that it had 415 target genes which referred to many pathways such as metabolic, protein and amino acid metabolism, RNA transport and circadian rhythm. However, the molecular mechanism of miR3627-5p regulation in chilling still needs more experimental studies.

The target genes of miR3627-5p: ppa025787mg, ppa007623mg, ppa016917mg, ppa021860mg were involved in pentose and glucuronate interconversions, starch and sucrose metabolism. Besides, miR3627-5p target gene ppa007934mg is involved in carbon metabolism and TCA cycle. The expression levels of these five target genes were all down-regulated (Additional file 7: Figure S1), which were consistent with the up-regulation of miR3627-5p. Also the predicted target genes of novel miRNA Pp03_22,312-5p (upregulated) were involved in sugar metabolism. This indicated that miRNA may participate in the formation of fruit sweetness, an essential characteristic of fruit quality which was in accordance with previous reports [60].

Different sugar components in leaves were analyzed by High Performance Liquid Chromatography (HPLC) and we found that the carbohydrate contents changed variously under UVB treatment (Fig. 7). Accordingly, we speculated some UVB-responsive miRNAs which targeted sugar-metabolizing pathway, such as miR3627-5p. Further qRT-PCR showed relative expression of the key genes in different sugar metabolic pathways (Fig. 8). Sorbitol is the main form of sugar in Rosaccae leaves and is transferred to fruits for the storage of carbohydrates in the mature stage. The content of sorbitol in our study was gradually increased with the developmental process, which was assistant with previous reports [61]. qPCR results showed that sorbitol-related genes were distinctly expressed with UVB treatment. The relative expression levels of *S6PDH* was remarkably up-regulated in UVB-group, indicating the increase of *S6PDH* activities and therefore stimulating the synthesis of sorbitol. *NAD-SDH1* and *NAD-SDH2* were down-regulated while the levels of *NADP-SDH* were increased after UVB treatment. This suggested that the plants restricted *NAD-SDHs* expression to reduce sorbitol degradation while increased *NADP-SDH* transcripts for the synthesis of fructose from sorbitol under UVB treatment. *SOT* was up-regulated, responsible for more active transportation of sorbitol, which accorded with the increase of sorbitol contents.

The content of sucrose did not change obviously during the continuous growth period, which could be explained by the slight change of expression levels of genes involved in sucrose metabolism, such as Sucrose synthase (*SUS*), Sucrose transporter (*SUT*), Tonoplast monosaccharide transporter (*TMT*) etc. In hexose pathway, the crucial genes *PFP-a* and *PFK* were up-regulated while Fructose-1,6-bisphosphatase (*FBPase*) and *HEX6* were down-regulated, implying the UVB treatment had an influence on hexose metabolism.

In our study, the UVB treatment led to different expression patterns of genes related with sugar metabolism. Previous research have shown that the regulation was mainly mediated by miRNA. Our results were essentially in agreement with these reports, and more investigations on functional verification need to be performed.

## Conclusions

**Fig. 9:**
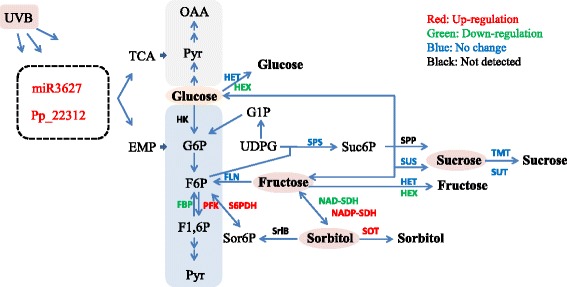
The schemetic of potential carbohydrate pathways regulated by miRNA under UVB radiation in greenhouse. Red: Genes were up-regulated transcriptional levels under UVB; Green: Down-regulation; Blue: No change; Black: Not detected by qRT-PCR

In this study, we constructed 2 miRNA libraries for low-dose UVB radiation groups and control groups of *P. persica* in greenhouse. A total of 164 known and 109 novel miRNAs were identified. In brief, 8 miRNAs were highly up-regulated and 7 miRNAs were significantly down-regulated in the UVB treatment groups, which were mainly predicted to be targeted in signal transduction, carbohydrate metabolism and stress response etc. Combined with qRT-PCR tests and the measurement of the related metabolites, our results showed that low-dose UVB radiation could regulate the expression patterns of some miRNAs e.g. miR3627-5p and Pp_22,312, and cause the expression levels of genes in carbohydrate (sorbitol, fructose, and glucose etc.) and chlorophyll pathway, and therefore indirectly affect sugar contents and fruit quality (Fig. 9). Our study provided a comprehensive database of miRNA for *P. persica* and a theoretical basis for further investigations of the function of miRNA in regulating the biological features of peach in greenhouse.

## Methods

### Plant material and tissue collection

The experiment was carried out on 7-year-old peach trees (*Prunus persica* var. *nectarine* Zhongyou No.5) cultivated in experimental station of Shandong Agriculture university, Taian, China. The trees were treated with 1.44 Kj m^−2^ d^−1^ UVB radiation during the whole growing period. UVB was provided by the dedicated UV lamp of 40 W, 297 nm (Nanjing Kazhi), hanging at the position of 1.5 m above the plants. UVB-type single-channel UV irradiator (Beijing Normal University Photoelectric Instrument Factory) equipped with 297 nm probe was used to determine the UVB radiation dose of 1.44 Kj m^−2^ d^−1^. The function leaves within the range of 80–120 cm below the lamp were selected. The on/off time of UV light was controlled through the electronic automatic control device, from 7 days after blossom to the fruit mature stage. UV light was kept on from 9 h30 to 10 h30 every day and stopped at cloudy, rainy and snowy days. Function leaves were sampled on the 7th day after blossom and every week after, until the mature period. These samples were washed with DEPC-treated H_2_O, immediately frozen in liquid nitrogen and stored at −80 °C until use. The function leaves of fruit mature stage were selected for high-quality deep-sequencing.

### Small RNA isolation and Illumina sequencing

Total RNA in peach was isolated using the mirVan miRNA Isolation kit (Ambion; Thermo Fisher Scientific, USA) and purified using the miRNeasy Mini kit (Qiagen, Germany), following the manufacturer’s instructions. RNA was quantified using a spectrophotometer (NanoDrop, Thermo Fisher Scientific, USA). Purified RNA was frozen in liquid nitrogen and then stored at −80 °C until required.

We constructed an RNA library using the NEBNext Ultra RNA Library Prep kit for Illumina (New England Biolabs, USA). There were four steps: Firstly, total RNA (approx. 1 μg) was spliced into shorter fragments (200–500 bp) in the NEBNext First Strand Synthesis Reaction Buffer and the fragments were used to produce the double-stranded cDNA; secondly, the cDNA was end-repaired and ligated with Illumina-specific adaptors; thirdly, we used 200 bp inserts from the library and selected suitable fragments for PCR amplification; last, we performed PCR using Phusion High-Fidelity DNA polymerase (New England Biolabs, USA) and purified the products with a QIAquick Nucleotide Removal kit (Qiagen, Germany). Then we sequenced the new RNA library on an Illumina HiSeq 2500 system using 2 × 150 base pairs paired-end sequencing.

### Analysis of sequencing data

Clean reads were obtained from raw reads after removing low-quality and adapter reads. SOAP software was used for the mapping of clean reads to the peach genome. The non-coding RNAs, including rRNAs, scRNAs, snoRNAs, snRNAs, and tRNAs deposited in the NCBI GenBank database and Rfam (11.0) database, were removed. We also excluded the small RNAs corresponding to the exons and introns of mRNA and repeat sequences. The remaining sRNA sequences were aligned to the miRBase 21 database, with a maximum of two mismatches, to identify known miRNAs in *P. persica*. The obtained sequences were used to predict hairpin structures using the perpl program. The remaining unannotated sRNAs were used to predict novel miRNAs using Mireap software [62].

### Target prediction of miRNAs and functional analysis

Target prediction of miRNAs followed rules referring to Allen et al. [63]: a. ≤two adjacent mismatches in the miRNA/target duplex; b. ≤ four mismatches between the sRNA and target gene; c. ≤ 2.5 mismatches at positions 1–12 of the 5′-miRNA/target duplex; d. no mismatches at 10–11 of the miRNA/target duplex; e. no adjacent mismatches at 2–12 of the miRNA/target duplex; f. the minimum free energy (MFE) of the miRNA/target duplex should be 75% of the MFE bound to the perfect complement. Target genes were searched using peach genome information. To better understand the roles of miRNAs in peach under low-dose UVB treatment, the potential target functions were annotated using the Gene Ontology and KEGG pathway database.

### Differential expression analysis of miRNAs

The miRNA reads were used to analyze differential expression and determine significant differences between the control and treatment libraries. The frequency of miRNAs was normalized to one million to reduce potential errors before calculating the fold-change, *P*-value and ratio. Normalized expression = (actual miRNA counts/total counts of clean reads) × 1,000,000. Fold-change = log2 (miRNA normalized read counts in the treatment library/ in the control library).

A fold change larger than 1 or less than −1 and a *P*-value less than 0.01 suggested the highly significant difference in the miRNA expression between two libraries. When the fold-change was greater than 1 or less than −1 and the P-value was between 0.01 and 0.05, the expression of the miRNA was significantly different between the two libraries. The ratio of miRNA normalized read counts in treatment library/in control library was used to determine changes in the expression of an miRNA in the treatment samples compared with the control samples. When the ratio was more than 2, the miRNA was indicated as up-regulated, and when the ratio was less than 1/2, the miRNA was down-regulated [64].

### Relative expression of miRNA and target genes

qRT-PCR was performed to verify the expression levels of identified miRNAs and targets genes using the IQ5 Quantitative Real-time PCR Detection System (Bio-Rad, California, USA) with the SYBR® PrimeScript™ miRNA RT-PCR Kit (TaKaRa, Dalian, China). The reactions were performed in a total volume of 25 μL containing 2.0 μL of diluted cDNA (100 ng/μL), 1 μL of each primer (10 μM), and 12.5 μL of SYBR Green premix Ex Taq II with the following reaction conditions: 95 °C for 30 s, followed by 40 cycles of 95 °C for 5 s and 60 °C for 20 s, then dissociation curve with 95 °C for 60 s, 55 °C for 30 s and 95 °C for 30 s. The reference genes for qRT-PCR of miRNA and target genes were miR5059 and beta-actin, respectively [65, 66]. Each sample was processed in triplicate. All validated primer sequences of miRNAs/target genes are listed in Table S5 and Table S6.

### Metabolites analysis by HPLC

Four sugar components: sucrose, sorbitol, glucose and fructose of leaves, were analyzed according to the method of Karkacier et al. [67]. In a mortar pre-cooled in the −20 °C refrigerator, 100 mg of fresh leaves were ground with liquid nitrogen and extracted with 1 mL NANO pure water into a 2 ml tube. The tubes were then vigorously shaken for 15 s, sonicated for 15 min and centrifuged at 12,000 rpm for 15 min. The supernatant was sterilized by filtering through a 0.45 μm membrane filter and stored at −20 °C prior to sugar components’ measurement using HPLC. The HPLC system was programmed to inject 50 μL crude extracts automatically. Online detection was performed using a Waters 410 differential refractrometer detector and the data were analyzed by Oirigin75 software. The whole program used a MetaCarb 87 °C equipped with a guard column as the analytical column, and the deionized water as the mobile phase with a 0.5 mL min^−1^ flow rate. Glucose, sorbitol, fructose and sucrose purchased from company were used as standards [68].

### Chlorophyll content

Function Leaves were taken every 7 days after flowering to measure chlorophyll content via spectrophotometry. The maximum UV absorption wavelength of chlorophyll a and chlorophyll b is 645 nm and 663 nm respectively. The total chlorophyll content was analyzed with the following formula [69]:

C_T_ = C_a_ + C_b_ = 20.29 A_645_ + 8.05 A_663_.

## Additional files

Additional file 1: Table S1. The summary of known miRNAs prediction and expression in control and UVB supplement libraries. (PDF 250 kb)

Additional file 2: Table S2. The summary of novel miRNAs prediction and expression in control and UVB supplement libraries. (PDF 162 kb)

Additional file 3: Table S3. Details of targets genes and their annotation, GO classification, and KEGG pathway for the known miRNAs. (PDF 2499 kb)

Additional file 4: Table S4. Details of targets genes and their annotation, GO classification, and KEGG pathway for the known miRNAs. (PDF 2823 kb)

Additional file 5: Table S5. Primers for qRT-PCR verification of miRNAs. (PDF 17 kb)

Additional file 6: Table S6. The names, Genbank IDs, involved pathways of verified target genes and their primers for qRT-PCR tests. (PDF 79 kb)

Additional file 7: Figure S1. qRT-PCR analysis of five target genes predicted for miR3627-5p. Beta-actin was the internal control. Each experiment was performed with three biological replicates. (PDF 66 kb)

## Abbreviations

CBR: Chlorophyll a-b binding protein
CHLG: Chlorophyll synthase
COP1: Constitutively photomorphogenic 1
FBPase: Fructose-1,6-bisphosphatase, chloroplastic
FLN1: Fructokinase-like 1
FLN2: Fructokinase-like 2
HET: Hexose transporter
HEX6: Hexose carrier protein
HPLC: High Performance Liquid Chromatography
HY5: Elongated hypocotyl 5
NADP-SDH: NADP-sorbitol dehydrogenase
NAD-SDH: NAD-sorbitol dehydrogenase
NAD-SDH2: NAD-sorbitol dehydrogenase 2
NIV8: Neutral invertase 8
PFK: Phosphofructokinase
PFP-α: Pyrophosphate--fructose 6-phosphate 1-phosphotransferase subunit alpha
PFP-β: Pyrophosphate--fructose 6-phosphate 1-phosphotransferase subunit beta
PORC: Pchlide oxidoreductase C
RCCR: Red chlorophyll catabolite reductase
S6PDH: Sorbitol −6- phosphate dehydrogenase
SCL: Scarecrow-like protein
SlGRAS24: A tomato (*solamum lycopersicum*) GRAS transcription factor gene
SOT: Sorbitol transporter
SPS: Sucrose phosphate synthase
SUS4: Sucrose synthase 4
SUT2: Sucrose transporter 2
SUT4: Sucrose transporter 4
TMT2: Tonoplast monosaccharide transporter
UVB: Ultraviolet radiation B
UVR8: UV resistance locus 8
