# Supplementary material for: Identification and characterization of Prunus persica miRNAs in response to UVB radiation in greenhouse through high-throughput sequencing
Source: BMC Genomics. 2017 Dec 2;18:938. doi: 10.1186/s12864-017-4347-5 (PMC5712094; doi:10.1186/s12864-017-4347-5)
Supplement: Supplementary file 1 — The summary of known miRNAs prediction and expression in control and UVB supplement libraries. (PDF 250 kb) [file 12864_2017_4347_MOESM1_ESM.pdf]

T_VS_CK	--	--	--	--	--	--	--	--	--	--	--	--	--	--	--	--	--
#miRNA family	#miRNA_ID	T1_read_count	T2_read_count	T3_read_count	CK1_read_count	CK2_read_count	CK3_read_count	T1_norm_read count	T2_norm_read count	T3_norm_read count	CK1_norm_read count	CK2_norm_read count	CK3_norm_read count	PValue	FC	log2(FC)	UpDown
miR1511	ppe-miR1511-3p	34514	11720	12590	34645	19460	29969	6557.7	2689.9	3787.2	5233.2	4368.4	6934.8	0.177	1.262	0.335	No_change
miR1511	ppe-miR1511-5p	217	116	93	181	194	201	41.23	26.62	27.98	27.34	43.55	46.51	0.189	1.359	0.443	No_change
miR156	ppe-miR156b	12	2	0	7	3	3	2.28	0.46	0	1.06	0.67	0.69	0.506	1.678	0.747	No_change
miR156	ppe-miR156c	3	3	7	13	2	4	0.57	0.69	2.11	1.96	0.45	0.93	0.337	1.591	0.67	No_change
miR156	ppe-miR156d	3	3	7	13	2	4	0.57	0.69	2.11	1.96	0.45	0.93	0.334	1.587	0.666	No_change
miR156	ppe-miR156e	3	3	7	14	2	4	0.57	0.69	2.11	2.11	0.45	0.93	0.406	1.512	0.597	No_change
miR156	ppe-miR156f	6	4	1	12	6	11	1.14	0.92	0.3	1.81	1.35	2.55	0.605	0.702	-0.511	No_change
miR156	ppe-miR156g	248	75	79	317	136	172	47.12	17.21	23.76	47.88	30.53	39.8	0.443	1.157	0.211	No_change
miR156	ppe-miR156h	250	75	79	317	136	173	47.5	17.21	23.76	47.88	30.53	40.03	0.439	1.159	0.213	No_change
miR156	ppe-miR156i	249	75	79	317	136	172	47.31	17.21	23.76	47.88	30.53	39.8	0.444	1.159	0.213	No_change
miR159	ppe-miR159	92617	10625	25570	90832	68503	163903	17597	2438.6	7691.7	13720	15378	37927	0.046	0.588	-0.767	No_change
miR160	ppe-miR160a	59	56	57	97	81	112	11.21	12.85	17.15	14.65	18.18	25.92	0.375	1.275	0.35	No_change
miR160	ppe-miR160b	60	58	57	96	80	116	11.4	13.31	17.15	14.5	17.96	26.84	0.349	1.291	0.369	No_change
miR162	ppe-miR162	450	118	143	617	275	421	85.5	27.08	43.02	93.2	61.73	97.42	0.776	0.949	-0.075	No_change
miR164	ppe-miR164a	5	0	1	11	8	3	0.95	0	0.3	1.66	1.8	0.69	0.133	0.455	-1.137	No_change
miR164	ppe-miR164b	5	0	1	11	7	3	0.95	0	0.3	1.66	1.57	0.69	0.156	0.486	-1.042	No_change
miR164	ppe-miR164c	5	0	1	11	7	3	0.95	0	0.3	1.66	1.57	0.69	0.159	0.485	-1.045	No_change
miR166	ppe-miR166a	284829	46792	78819	411829	134306	249179	54118	10739	23709	62208	30149	57660	0.47	0.858	-0.22	No_change
miR166	ppe-miR166b	284670	46749	78751	411530	134254	248978	54087	10729	23689	62163	30138	57614	0.463	0.858	-0.22	No_change
miR166	ppe-miR166c	284547	46741	78723	411316	134144	248918	54064	10728	23680	62130	30113	57600	0.463	0.859	-0.22	No_change
miR166	ppe-miR166d	284680	46749	78753	411533	134241	249037	54089	10729	23690	62163	30135	57627	0.466	0.858	-0.22	No_change
miR166	ppe-miR166e	284680	46749	78753	411533	134241	249037	54089	10729	23690	62163	30135	57627	0.469	0.858	-0.22	No_change
miR167	ppe-miR167a	1071	280	396	1692	912	1155	203.49	64.26	119.12	255.58	204.73	267.27	0.261	0.812	-0.301	No_change
miR167	ppe-miR167b	782	161	186	1162	618	600	148.58	36.95	55.95	175.52	138.73	138.84	0.206	0.764	-0.388	No_change
miR167	ppe-miR167c	6236	1984	2371	10221	4432	6781	1184.8	455.35	713.21	1543.9	994.91	1569.1	0.593	0.911	-0.135	No_change
miR167	ppe-miR167d	1028	266	290	1518	836	896	195.32	61.05	87.23	229.3	187.67	207.33	0.316	0.823	-0.281	No_change
miR168	ppe-miR168	212	128	128	334	195	304	40.28	29.38	38.5	50.45	43.77	70.35	0.516	1.147	0.198	No_change
miR169	ppe-miR169a	0	1	0	4	1	1	0	0.23	0	0.6	0.22	0.23	0.696	0.359	-1.477	No_change
miR169	ppe-miR169b	0	1	0	4	1	1	0	0.23	0	0.6	0.22	0.23	0.705	0.368	-1.443	No_change
miR169	ppe-miR169c	0	1	0	3	1	1	0	0.23	0	0.45	0.22	0.23	1	0.424	-1.238	No_change
miR169	ppe-miR169d	40	26	17	42	45	43	7.6	5.97	5.11	6.34	10.1	9.95	0.548	1.191	0.252	No_change
miR169	ppe-miR169e-5p	40	26	17	42	45	43	7.6	5.97	5.11	6.34	10.1	9.95	0.552	1.192	0.253	No_change

miR169	ppe-miR169f	83	31	47	95	52	87	15.77	7.11	14.14	14.35	11.67	20.13	0.251	1.306	0.385	No_change
miR169	ppe-miR169g	83	31	47	96	52	87	15.77	7.11	14.14	14.5	11.67	20.13	0.256	1.301	0.379	No_change
miR169	ppe-miR169h	88	33	48	105	55	88	16.72	7.57	14.44	15.86	12.35	20.36	0.249	1.294	0.372	No_change
miR169	ppe-miR169i	83	31	47	96	52	87	15.77	7.11	14.14	14.5	11.67	20.13	0.252	1.3	0.379	No_change
miR169	ppe-miR169j	90	33	49	105	56	90	17.1	7.57	14.74	15.86	12.57	20.83	0.241	1.296	0.374	No_change
miR171	ppe-miR171a	108	29	34	174	83	96	20.52	6.66	10.23	26.28	18.63	22.21	0.444	0.852	-0.231	No_change
miR171	ppe-miR171b	4	1	1	3	1	0	0.76	0.23	0.3	0.45	0.22	0	0.195	2.501	1.322	No_change
miR171	ppe-miR171c	109	29	31	170	83	96	20.71	6.66	9.33	25.68	18.63	22.21	0.418	0.841	-0.249	No_change
miR171	ppe-miR171d-3p	2	1	5	2	0	1	0.38	0.23	1.5	0.3	0	0.23	0.026	5.099	2.35	Up_regulate
miR171	ppe-miR171d-5p	7	0	2	2	1	1	1.33	0	0.6	0.3	0.22	0.23	0.103	3.462	1.792	No_change
miR171	ppe-miR171e	17	3	1	22	6	7	3.23	0.69	0.3	3.32	1.35	1.62	1	0.997	-0.004	No_change
miR171	ppe-miR171f	112	31	34	174	88	98	21.28	7.11	10.23	26.28	19.75	22.68	0.482	0.86	-0.218	No_change
miR171	ppe-miR171g	108	29	32	171	83	96	20.52	6.66	9.63	25.83	18.63	22.21	0.426	0.843	-0.246	No_change
miR171	ppe-miR171h	120	23	29	133	57	84	22.8	5.28	8.72	20.09	12.8	19.44	0.908	1.044	0.063	No_change
miR172	ppe-miR172a-3p	40	25	22	58	50	73	7.6	5.74	6.62	8.76	11.22	16.89	0.845	0.926	-0.11	No_change
miR172	ppe-miR172a-5p	189	95	89	156	153	158	35.91	21.8	26.77	23.56	34.35	36.56	0.08	1.476	0.561	No_change
miR172	ppe-miR172b	30	24	15	42	47	64	5.7	5.51	4.51	6.34	10.55	14.81	0.737	0.874	-0.195	No_change
miR172	ppe-miR172c	0	2	1	1	3	5	0	0.46	0.3	0.15	0.67	1.16	1	0.678	-0.561	No_change
miR172	ppe-miR172d	0	0	1	2	0	0	0	0	0.3	0.3	0	0	1	0.996	-0.006	No_change
miR2111	ppe-miR2111a	2	0	0	1	2	2	0.38	0	0	0.15	0.45	0.46	0.699	0.714	-0.486	No_change
miR2111	ppe-miR2111b	2	0	0	1	2	2	0.38	0	0	0.15	0.45	0.46	0.696	0.721	-0.472	No_change
miR2111	ppe-miR2111c	2	0	0	1	2	2	0.38	0	0	0.15	0.45	0.46	0.694	0.725	-0.463	No_change
miR2111	ppe-miR2111d	2	0	0	1	2	2	0.38	0	0	0.15	0.45	0.46	0.701	0.708	-0.499	No_change
miR319	ppe-miR319a	89	17	40	123	116	111	16.91	3.9	12.03	18.58	26.04	25.69	0.134	0.688	-0.539	No_change
miR3627	ppe-miR3627-3p	6	1	2	5	3	0	1.14	0.23	0.6	0.76	0.67	0	0.35	1.926	0.946	No_change
miR3627	ppe-miR3627-5p	54	11	8	10	5	4	10.26	2.52	2.41	1.51	1.12	0.93	0	6.024	2.591	Up_regulate
miR390	ppe-miR390	150	39	78	232	150	158	28.5	8.95	23.46	35.04	33.67	36.56	0.553	0.885	-0.176	No_change
miR393	ppe-miR393a	683	187	178	916	422	516	129.77	42.92	53.54	138.36	94.73	119.4	0.898	0.975	-0.037	No_change
miR393	ppe-miR393b	1395	276	312	2195	832	940	265.05	63.34	93.85	331.56	186.77	217.52	0.357	0.825	-0.277	No_change
miR394	ppe-miR394a	2054	626	696	1535	906	1715	390.26	143.67	209.36	231.87	203.38	396.85	0.06	1.421	0.507	No_change
miR394	ppe-miR394b	1953	606	647	1436	861	1649	371.07	139.08	194.62	216.91	193.28	381.58	0.061	1.42	0.506	No_change
miR395	ppe-miR395a-3p	341	65	87	695	238	242	64.79	14.92	26.17	104.98	53.43	56	0.143	0.713	-0.488	No_change
miR395	ppe-miR395a-5p	45	8	20	64	39	56	8.55	1.84	6.02	9.67	8.75	12.96	0.351	0.794	-0.332	No_change
miR395	ppe-miR395b-3p	341	65	87	695	238	242	64.79	14.92	26.17	104.98	53.43	56	0.147	0.713	-0.488	No_change
miR395	ppe-miR395b-5p	45	8	20	64	39	56	8.55	1.84	6.02	9.67	8.75	12.96	0.345	0.795	-0.331	No_change

miR395	ppe-miR395c	50	2	10	103	32	50	9.5	0.46	3.01	15.56	7.18	11.57	0.048	0.521	-0.94	No_change
miR395	ppe-miR395d	43	2	8	94	28	45	8.17	0.46	2.41	14.2	6.29	10.41	0.049	0.495	-1.014	Down_regulate
miR395	ppe-miR395e	43	2	8	94	28	45	8.17	0.46	2.41	14.2	6.29	10.41	0.046	0.495	-1.014	Down_regulate
miR395	ppe-miR395f	49	2	10	102	31	48	9.31	0.46	3.01	15.41	6.96	11.11	0.062	0.525	-0.93	No_change
miR395	ppe-miR395g	50	2	10	103	32	49	9.5	0.46	3.01	15.56	7.18	11.34	0.056	0.523	-0.935	No_change
miR395	ppe-miR395h	50	2	10	103	32	49	9.5	0.46	3.01	15.56	7.18	11.34	0.055	0.523	-0.934	No_change
miR395	ppe-miR395i	50	2	10	103	32	49	9.5	0.46	3.01	15.56	7.18	11.34	0.053	0.524	-0.933	No_change
miR395	ppe-miR395j	47	3	10	94	30	44	8.93	0.69	3.01	14.2	6.73	10.18	0.089	0.568	-0.816	No_change
miR395	ppe-miR395k	47	3	10	94	30	44	8.93	0.69	3.01	14.2	6.73	10.18	0.087	0.568	-0.815	No_change
miR395	ppe-miR395l	49	2	10	103	31	48	9.31	0.46	3.01	15.56	6.96	11.11	0.06	0.523	-0.936	No_change
miR395	ppe-miR395m	39	3	8	84	26	40	7.41	0.69	2.41	12.69	5.84	9.26	0.065	0.54	-0.889	No_change
miR395	ppe-miR395n	47	4	10	94	30	46	8.93	0.92	3.01	14.2	6.73	10.64	0.079	0.581	-0.782	No_change
miR395	ppe-miR395o	45	8	20	64	39	56	8.55	1.84	6.02	9.67	8.75	12.96	0.341	0.795	-0.331	No_change
miR396	ppe-miR396a	511	73	134	838	412	503	97.09	16.75	40.31	126.58	92.49	116.39	0.048	0.645	-0.632	No_change
miR396	ppe-miR396b	15837	2993	5162	23620	10665	16194	3009	686.92	1552.8	3567.9	2394.1	3747.3	0.224	0.796	-0.329	No_change
miR397	ppe-miR397	438	160	148	139	121	279	83.22	36.72	44.52	21	27.16	64.56	0	2.417	1.273	Up_regulate
miR398	ppe-miR398a-3p	13845	6239	8734	14928	8744	21112	2630.6	1431.9	2627.3	2254.9	1962.9	4885.3	0.262	1.276	0.352	No_change
miR398	ppe-miR398a-5p	1501	532	691	947	892	1993	285.19	122.1	207.86	143.05	200.24	461.18	0.315	1.268	0.342	No_change
miR398	ppe-miR398b	16864	6677	10055	15907	10788	24371	3204.2	1532.4	3024.6	2402.8	2421.7	5639.5	0.286	1.259	0.332	No_change
miR399	ppe-miR399a	4	0	6	43	27	34	0.76	0	1.8	6.5	6.06	7.87	0	0.184	-2.443	Down_regulate
miR399	ppe-miR399b	30	2	50	473	89	105	5.7	0.46	15.04	71.45	19.98	24.3	0.007	0.288	-1.796	Down_regulate
miR399	ppe-miR399c	0	0	0	4	0	2	0	0	0	0.6	0	0.46	0.146	0.077	-3.699	No_change
miR399	ppe-miR399d	0	0	0	4	0	2	0	0	0	0.6	0	0.46	0.144	0.077	-3.703	No_change
miR399	ppe-miR399e	0	0	0	4	0	2	0	0	0	0.6	0	0.46	0.138	0.076	-3.717	No_change
miR399	ppe-miR399f	0	0	0	4	0	2	0	0	0	0.6	0	0.46	0.138	0.076	-3.717	No_change
miR399	ppe-miR399g	0	0	0	4	0	2	0	0	0	0.6	0	0.46	0.145	0.077	-3.702	No_change
miR399	ppe-miR399h	0	0	0	4	0	2	0	0	0	0.6	0	0.46	0.144	0.077	-3.703	No_change
miR399	ppe-miR399i	0	0	0	4	0	2	0	0	0	0.6	0	0.46	0.15	0.077	-3.693	No_change
miR399	ppe-miR399j	0	0	0	4	0	2	0	0	0	0.6	0	0.46	0.152	0.078	-3.688	No_change
miR399	ppe-miR399k	0	0	0	4	0	2	0	0	0	0.6	0	0.46	0.15	0.077	-3.692	No_change
miR399	ppe-miR399l	0	0	0	4	0	2	0	0	0	0.6	0	0.46	0.149	0.077	-3.693	No_change
miR399	ppe-miR399m	0	0	0	3	0	0	0	0	0	0.45	0	0	0.545	0.151	-2.729	No_change
miR399	ppe-miR399n	0	0	0	3	0	0	0	0	0	0.45	0	0	0.541	0.15	-2.736	No_change
miR403	ppe-miR403	1584	489	543	2330	897	1185	300.96	112.23	163.34	351.95	201.36	274.21	0.631	1.092	0.127	No_change
miR477	ppe-miR477-3p	1	0	2	1	5	3	0.19	0	0.6	0.15	1.12	0.69	0.641	0.592	-0.756	No_change

miR477	ppe-miR477-5p	36	11	9	15	17	16	6.84	2.52	2.71	2.27	3.82	3.7	0.072	1.919	0.94	No_change
miR477	ppe-miR477a-3p	5	2	4	3	1	6	0.95	0.46	1.2	0.45	0.22	1.39	0.173	2.005	1.004	No_change
miR477	ppe-miR477a-5p	3	0	0	0	0	0	0.57	0	0	0	0	0	0.525	10.926	3.45	No_change
miR477	ppe-miR477b-3p	5	2	4	3	1	6	0.95	0.46	1.2	0.45	0.22	1.39	0.178	2.011	1.008	No_change
miR477	ppe-miR477b-5p	3	0	0	0	0	0	0.57	0	0	0	0	0	0.524	10.947	3.452	No_change
miR482	ppe-miR482a-3p	136	42	30	175	137	164	25.84	9.64	9.02	26.43	30.75	37.95	0.129	0.722	-0.47	No_change
miR482	ppe-miR482a-5p	88	39	35	152	42	43	16.72	8.95	10.53	22.96	9.43	9.95	0.242	1.389	0.474	No_change
miR482	ppe-miR482b-3p	4557	1210	1286	7192	3634	3774	865.83	277.71	386.84	1086.4	815.77	873.31	0.314	0.826	-0.275	No_change
miR482	ppe-miR482b-5p	228	104	131	185	143	184	43.32	23.87	39.41	27.94	32.1	42.58	0.01	1.72	0.783	No_change
miR482	ppe-miR482c-3p	330	139	111	506	266	364	62.7	31.9	33.39	76.43	59.71	84.23	0.809	0.951	-0.073	No_change
miR482	ppe-miR482c-5p	1939	1134	896	2485	1981	1606	368.41	260.26	269.52	375.37	444.7	371.63	0.292	1.266	0.34	No_change
miR482	ppe-miR482e	1179	270	319	1787	1010	1192	224.01	61.97	95.96	269.93	226.73	275.83	0.082	0.729	-0.455	No_change
miR482	ppe-miR482f	3277	780	686	4418	2639	2958	622.63	179.02	206.35	667.35	592.41	684.48	0.162	0.757	-0.402	No_change
miR5225	ppe-miR5225-5p	1	1	3	5	3	2	0.19	0.23	0.9	0.76	0.67	0.46	1	0.973	-0.04	No_change
miR530	ppe-miR530	239	67	108	369	172	196	45.41	15.38	32.49	55.74	38.61	45.35	0.878	1.032	0.045	No_change
miR535	ppe-miR535a	310	73	104	590	284	152	58.9	16.75	31.28	89.12	63.75	35.17	0.449	0.824	-0.279	No_change
miR535	ppe-miR535b	1547	438	644	3158	1759	925	293.93	100.53	193.72	477.02	394.87	214.05	0.367	0.805	-0.314	No_change
miR6258	ppe-miR6258	54	9	19	98	30	47	10.26	2.07	5.72	14.8	6.73	10.88	0.518	0.832	-0.266	No_change
miR6260	ppe-miR6260	1	2	0	1	0	0	0.19	0.46	0	0.15	0	0	0.181	4.579	2.195	No_change
miR6261	ppe-miR6261	1	1	1	5	2	0	0.19	0.23	0.3	0.76	0.45	0	1	0.817	-0.291	No_change
miR6263	ppe-miR6263	1	0	0	1	0	4	0.19	0	0	0.15	0	0.93	0.675	0.391	-1.354	No_change
miR6267	ppe-miR6267c-3p	45	8	11	23	18	32	8.55	1.84	3.31	3.47	4.04	7.4	0.345	1.398	0.484	No_change
miR6267	ppe-miR6267c-5p	2	0	1	3	1	1	0.38	0	0.3	0.45	0.22	0.23	1	1.067	0.094	No_change
miR6269	ppe-miR6269	2	0	0	2	4	3	0.38	0	0	0.3	0.9	0.69	0.231	0.39	-1.358	No_change
miR6274	ppe-miR6274b-3p	2	0	0	3	2	0	0.38	0	0	0.45	0.45	0	0.704	0.716	-0.482	No_change
miR6274	ppe-miR6274b-5p	3	2	0	2	1	0	0.57	0.46	0	0.3	0.22	0	0.337	2.74	1.454	No_change
miR6277	ppe-miR6277	1	0	0	1	0	1	0.19	0	0	0.15	0	0.23	1	0.915	-0.128	No_change
miR6281	ppe-miR6281	36	2	6	28	18	29	6.84	0.46	1.8	4.23	4.04	6.71	0.652	0.863	-0.213	No_change
miR6282	ppe-miR6282	7	1	2	11	2	5	1.33	0.23	0.6	1.66	0.45	1.16	1	0.998	-0.003	No_change
miR6284	ppe-miR6284	14	3	10	26	8	20	2.66	0.69	3.01	3.93	1.8	4.63	1	0.955	-0.066	No_change
miR6285	ppe-miR6285	9	4	1	19	14	10	1.71	0.92	0.3	2.87	3.14	2.31	0.229	0.567	-0.818	No_change
miR6286	ppe-miR6286	2	2	0	4	2	3	0.38	0.46	0	0.6	0.45	0.69	1	0.823	-0.28	No_change
miR6287	ppe-miR6287	0	0	1	3	1	2	0	0	0.3	0.45	0.22	0.46	0.693	0.354	-1.499	No_change
miR6288	ppe-miR6288a	4	2	7	10	0	2	0.76	0.46	2.11	1.51	0	0.46	0.105	2.501	1.322	No_change
miR6288	ppe-miR6288b-3p	1	0	0	1	3	2	0.19	0	0	0.15	0.67	0.46	0.25	0.33	-1.598	No_change

miR6288	ppe-miR6288c-3p	5	2	2	5	2	7	0.95	0.46	0.6	0.76	0.45	1.62	0.845	1.158	0.212	No_change
miR6289	ppe-miR6289	1	0	0	0	1	0	0.19	0	0	0	0.22	0	1	1.488	0.574	No_change
miR6291	ppe-miR6291b	4	0	0	5	1	3	0.76	0	0	0.76	0.22	0.69	0.556	0.76	-0.395	No_change
miR6291	ppe-miR6291c-3p	2	0	0	0	0	0	0.38	0	0	0	0	0	0.522	7.858	2.974	No_change
miR6291	ppe-miR6291c-5p	0	0	0	1	0	1	0	0	0	0.15	0	0.23	1	0.195	-2.358	No_change
miR6293	ppe-miR6293	109	19	24	142	60	68	20.71	4.36	7.22	21.45	13.47	15.74	0.734	0.923	-0.116	No_change
miR6295	ppe-miR6295	82	30	36	145	68	83	15.58	6.89	10.83	21.9	15.26	19.21	0.814	0.938	-0.092	No_change
miR7122	ppe-miR7122a-3p	2	1	1	7	1	6	0.38	0.23	0.3	1.06	0.22	1.39	0.641	0.545	-0.877	No_change
miR7122	ppe-miR7122a-5p	4746	757	944	8715	2910	3219	901.74	173.74	283.96	1316.4	653.24	744.88	0.127	0.696	-0.523	No_change
miR7122	ppe-miR7122b-3p	9	1	2	10	7	2	1.71	0.23	0.6	1.51	1.57	0.46	1	1.037	0.052	No_change
miR7122	ppe-miR7122b-5p	64	13	13	193	71	63	12.16	2.98	3.91	29.15	15.94	14.58	0.009	0.468	-1.096	Down_regulate
miR7125	ppe-miR7125-3p	67	12	24	80	21	69	12.73	2.75	7.22	12.08	4.71	15.97	0.833	1.073	0.102	No_change
miR8122	ppe-miR8122-3p	3230	1433	1311	4008	2624	2468	613.7	328.89	394.36	605.42	589.04	571.1	0.293	1.229	0.297	No_change
miR8122	ppe-miR8122-5p	554	203	176	768	587	614	105.26	46.59	52.94	116.01	131.77	142.08	0.359	0.825	-0.277	No_change
miR8125	ppe-miR8125	66	17	25	91	35	65	12.54	3.9	7.52	13.75	7.86	15.04	0.95	1.021	0.031	No_change
miR8126	ppe-miR8126-3p	6	0	0	2	0	1	1.14	0	0	0.3	0	0.23	0.53	2.787	1.478	No_change
miR8126	ppe-miR8126-5p	388	76	78	247	110	132	73.72	17.44	23.46	37.31	24.69	30.54	0.008	1.784	0.835	No_change
miR8127	ppe-miR8127-3p	74	21	15	129	94	75	14.06	4.82	4.51	19.49	21.1	17.36	0.061	0.604	-0.727	No_change
miR8127	ppe-miR8127-5p	65	45	48	136	127	106	12.35	10.33	14.44	20.54	28.51	24.53	0.572	0.85	-0.234	No_change
miR8129	ppe-miR8129-3p	2	1	1	7	3	4	0.38	0.23	0.3	1.06	0.67	0.93	0.486	0.534	-0.904	No_change
miR8129	ppe-miR8129-5p	10	2	1	12	4	6	1.9	0.46	0.3	1.81	0.9	1.39	1	1.012	0.017	No_change
miR8131	ppe-miR8131-3p	2	5	0	7	2	3	0.38	1.15	0	1.06	0.45	0.69	0.75	1.296	0.374	No_change
miR8131	ppe-miR8131-5p	67	32	31	86	42	63	12.73	7.34	9.33	12.99	9.43	14.58	0.263	1.316	0.396	No_change
miR8133	ppe-miR8133-3p	4	0	0	9	5	8	0.76	0	0	1.36	1.12	1.85	0.013	0.32	-1.646	Down_regulate
miR8133	ppe-miR8133-5p	1	0	0	1	1	0	0.19	0	0	0.15	0.22	0	1	0.915	-0.128	No_change
miR827	ppe-miR827	574	126	142	1121	548	507	109.06	28.92	42.71	169.33	123.02	117.32	0.038	0.639	-0.646	No_change
miR858	ppe-miR858	4	0	0	3	0	1	0.76	0	0	0.45	0	0.23	1	1.588	0.667	No_change
