# Supplementary material for: Identification and characterization of Prunus persica miRNAs in response to UVB radiation in greenhouse through high-throughput sequencing
Source: BMC Genomics. 2017 Dec 2;18:938. doi: 10.1186/s12864-017-4347-5 (PMC5712094; doi:10.1186/s12864-017-4347-5)
Supplement: Supplementary file 2 — The summary of novel miRNAs prediction and expression in control and UVB supplement libraries. (PDF 162 kb) [file 12864_2017_4347_MOESM2_ESM.pdf]

T_VS_CK	--	--	--	--	--	--	--	--	--	--	--	--	--	--	--	--	--	--
#miRNA_ID	T1_read_count	T2_read_count	T3_read_count	CK1_read_count	CK2_read_count	CK3_read_count	T1_norm_read_count	T2_norm_read_count	T3_norm_read_count	CK1_norm_read_count	CK2_norm_read_count	CK3_norm_read_count	PValue	FC	log2(FC)	UpDown
Pp01_172-5p(osa-miR435)	121	42	40	161	61	76	22.99	9.64	12.03	24.32	13.69	17.59	0.27	1.27	0.35	No_change
Pp01_1934-5p	8	0	1	8	0	0	1.52	0	0.3	1.21	0	0	0.526	1.96	0.97	No_change
Pp01_2199-3p	34	14	7	61	15	19	6.46	3.21	2.11	9.21	3.37	4.4	0.696	1.12	0.16	No_change
Pp01_2969-3p(mtr-miR2118)	1899	542	410	2832	1090	1645	360.81	124.39	123.33	427.78	244.69	380.65	0.498	0.87	-0.21	No_change
Pp01_2969-5p	75	34	28	95	40	44	14.25	7.8	8.42	14.35	8.98	10.18	0.05	1.47	0.55	No_change
Pp01_2975-3p	850	227	227	1370	623	771	161.5	52.1	68.28	206.94	139.85	178.41	0.264	0.8	-0.32	No_change
Pp01_2975-5p	150	56	53	181	98	120	28.5	12.85	15.94	27.34	22	27.77	0.474	1.17	0.22	No_change
Pp01_4331-3p	4	1	0	10	3	3	0.76	0.23	0	1.51	0.67	0.69	0.302	0.57	-0.8	No_change
Pp01_5202-5p	9	3	7	26	9	13	1.71	0.69	2.11	3.93	2.02	3.01	0.49	0.74	-0.43	No_change
Pp01_5909-5p	4	1	4	10	3	3	0.76	0.23	1.2	1.51	0.67	0.69	0.836	1.03	0.04	No_change
Pp01_6039-3p	4	1	0	1	1	1	0.76	0.23	0	0.15	0.22	0.23	0.477	2.61	1.38	No_change
Pp01_6039-5p	817	221	282	807	399	635	155.23	50.72	84.83	121.9	89.57	146.94	0.378	1.21	0.28	No_change
Pp01_634-3p	7	4	3	10	10	4	1.33	0.92	0.9	1.51	2.24	0.93	0.872	1.05	0.07	No_change
Pp01_634-5p	95	13	14	140	48	42	18.05	2.98	4.21	21.15	10.78	9.72	0.532	0.87	-0.2	No_change
Pp01_6591-5p(ptc-miR6475)	4	4	5	14	8	5	0.76	0.92	1.5	2.11	1.8	1.16	1	0.91	-0.14	No_change
Pp01_732-3p	1	0	0	1	0	2	0.19	0	0	0.15	0	0.46	1	0.61	-0.71	No_change
Pp01_732-5p(aly-miR825-3p)	8	0	1	11	7	3	1.52	0	0.3	1.66	1.57	0.69	0.42	0.76	-0.39	No_change
Pp01_7761-3p	25	10	6	40	13	15	4.75	2.3	1.8	6.04	2.92	3.47	0.711	1.13	0.17	No_change
Pp01_7761-5p	23	2	12	35	7	12	4.37	0.46	3.61	5.29	1.57	2.78	0.516	1.3	0.37	No_change
Pp01_7885-3p	1	1	0	5	3	1	0.19	0.23	0	0.76	0.67	0.23	0.519	0.43	-1.21	No_change
Pp01_7885-5p(ath-miR825)	124	11	18	220	69	37	23.56	2.52	5.41	33.23	15.49	8.56	0.347	0.76	-0.39	No_change
Pp01_8036-3p(gma-miR4394)	1	1	0	6	3	2	0.19	0.23	0	0.91	0.67	0.46	0.344	0.35	-1.5	No_change
Pp01_8673-5p	25	10	4	32	18	14	4.75	2.3	1.2	4.83	4.04	3.24	0.882	1.08	0.11	No_change
Pp01_9747-5p(mtr-miR5282)	400	98	94	612	223	224	76	22.49	28.28	92.44	50.06	51.83	0.941	0.99	-0.02	No_change
Pp02_11791-3p	63	20	21	73	26	27	11.97	4.59	6.32	11.03	5.84	6.25	0.042	1.54	0.62	No_change
Pp02_12389-3p	53	11	11	77	41	30	10.07	2.52	3.31	11.63	9.2	6.94	0.521	0.86	-0.22	No_change
Pp02_13946-3p	36	11	17	75	38	31	6.84	2.52	5.11	11.33	8.53	7.17	0.515	0.83	-0.27	No_change
Pp02_14368-3p(hbr-miR9386)	35	9	10	49	22	21	6.65	2.07	3.01	7.4	4.94	4.86	0.895	1.05	0.06	No_change
Pp02_15663-5p(vvi-miR172d)	68	24	32	111	82	71	12.92	5.51	9.63	16.77	18.41	16.43	0.5	0.85	-0.24	No_change
Pp02_16509-5p	30	5	6	24	8	8	5.7	1.15	1.8	3.63	1.8	1.85	0.073	1.78	0.83	No_change
Pp03_17365-3p	13	6	8	21	12	12	2.47	1.38	2.41	3.17	2.69	2.78	0.687	1.12	0.16	No_change
Pp03_17431-3p(lja-miR408)	3	0	1	8	2	1	0.57	0	0.3	1.21	0.45	0.23	0.567	0.68	-0.56	No_change

Pp03_18259-3p	85	22	18	76	52	73	16.15	5.05	5.41	11.48	11.67	16.89	0.878	0.98	-0.03	No_change
Pp03_18370-5p	30	0	3	38	7	5	5.7	0	0.9	5.74	1.57	1.16	1	1.04	0.06	No_change
Pp03_18798-5p	27	2	5	31	15	9	5.13	0.46	1.5	4.68	3.37	2.08	1	1.02	0.03	No_change
Pp03_19156-3p	8	1	2	11	7	1	1.52	0.23	0.6	1.66	1.57	0.23	1	1.04	0.05	No_change
Pp03_19156-5p	95	13	14	140	48	42	18.05	2.98	4.21	21.15	10.78	9.72	0.529	0.87	-0.2	No_change
Pp03_19448-5p	14	1	2	16	2	3	2.66	0.23	0.6	2.42	0.45	0.69	0.542	1.42	0.51	No_change
Pp03_19842-3p	1622	3786	1266	1489	1060	756	308.18	868.93	380.82	224.92	237.95	174.94	0	5.23	2.39	Up_regulate
Pp03_19842-5p	0	2	0	0	0	0	0	0.46	0	0	0	0	0.316	10.7	3.41	No_change
Pp03_20976-3p	38	28	19	58	26	29	7.22	6.43	5.72	8.76	5.84	6.71	0.095	1.56	0.64	No_change
Pp03_20976-5p	2	0	0	2	1	0	0.38	0	0	0.3	0.22	0	1	1.14	0.19	No_change
Pp03_21418-3p(osa-miR5081)	11	2	2	9	11	4	2.09	0.46	0.6	1.36	2.47	0.93	1	1.07	0.1	No_change
Pp03_22312-3p(vvi-miR2950-5p)	23	4	4	12	6	4	4.37	0.92	1.2	1.81	1.35	0.93	0.01	2.42	1.28	Up_regulate
Pp03_22312-5p	8	4	2	2	1	2	1.52	0.92	0.6	0.3	0.22	0.46	0.004	4.67	2.22	Up_regulate
Pp04_22766-5p	20	2	9	28	13	12	3.8	0.46	2.71	4.23	2.92	2.78	1	1.04	0.05	No_change
Pp04_23116-5p	49	7	2	89	23	11	9.31	1.61	0.6	13.44	5.16	2.55	0.512	0.78	-0.35	No_change
Pp04_23748-5p	49	11	21	74	36	43	9.31	2.52	6.32	11.18	8.08	9.95	0.821	0.94	-0.09	No_change
Pp04_25089-3p	64	19	17	128	53	36	12.16	4.36	5.11	19.33	11.9	8.33	0.515	0.86	-0.23	No_change
Pp04_25089-5p	1	0	1	0	0	1	0.19	0	0.3	0	0	0.23	1	2.71	1.44	No_change
Pp04_25168-3p	59	25	24	44	60	47	11.21	5.74	7.22	6.65	13.47	10.88	0.476	1.22	0.28	No_change
Pp04_25168-5p(gma-miR4376-5p)	386	117	169	470	251	572	73.34	26.85	50.84	70.99	56.35	132.36	0.615	0.87	-0.2	No_change
Pp04_25866-3p(gma-miR4401a)	9	3	1	16	5	7	1.71	0.69	0.3	2.42	1.12	1.62	0.733	0.84	-0.26	No_change
Pp04_25866-5p	2	2	0	1	2	1	0.38	0.46	0	0.15	0.45	0.23	0.755	1.7	0.77	No_change
Pp04_25890-3p	4	1	2	3	4	3	0.76	0.23	0.6	0.45	0.9	0.69	1	1.24	0.31	No_change
Pp04_26412-3p	3454	1089	1212	3896	1958	2587	656.26	249.94	364.58	588.5	439.54	598.63	0.357	1.2	0.27	No_change
Pp04_26412-5p	39	17	12	32	28	44	7.41	3.9	3.61	4.83	6.29	10.18	0.836	1.09	0.12	No_change
Pp04_26823-5p	5	0	3	7	1	3	0.95	0	0.9	1.06	0.22	0.69	0.82	1.31	0.39	No_change
Pp04_26958-3p	13	6	8	21	12	12	2.47	1.38	2.41	3.17	2.69	2.78	0.685	1.11	0.16	No_change
Pp04_27840-3p	18276	7980	7374	17536	12785	11971	3472.45	1831.49	2218.16	2648.86	2870.01	2770.1	0.08	1.46	0.55	No_change
Pp04_27840-5p	1	1	2	0	1	4	0.19	0.23	0.6	0	0.22	0.93	0.794	1.39	0.47	No_change
Pp05_28331-3p	11	3	3	8	2	5	2.09	0.69	0.9	1.21	0.45	1.16	0.088	2	1	No_change
Pp05_28381-3p	8	1	0	5	5	0	1.52	0.23	0	0.76	1.12	0	0.829	1.41	0.49	No_change
Pp05_28652-5p	10	2	1	5	8	2	1.9	0.46	0.3	0.76	1.8	0.46	0.717	1.45	0.54	No_change
Pp05_28810-5p	8	2	1	9	4	4	1.52	0.46	0.3	1.36	0.9	0.93	1	1.16	0.21	No_change
Pp05_28899-3p(gma-miR1513a-5p)	8	2	3	27	13	10	1.52	0.46	0.9	4.08	2.92	2.31	0.03	0.47	-1.08	Down_regulate
Pp05_29903-3p	104	29	35	128	66	67	19.76	6.66	10.53	19.33	14.82	15.5	0.576	1.14	0.19	No_change

Pp05_31135-5p	38	6	11	57	18	14	7.22	1.38	3.31	8.61	4.04	3.24	0.709	1.12	0.17	No_change
Pp05_31273-5p(bdi-miR7768a-5p)	7	6	3	18	12	9	1.33	1.38	0.9	2.72	2.69	2.08	0.62	0.76	-0.39	No_change
Pp05_31624-3p	5	2	2	14	7	4	0.95	0.46	0.6	2.11	1.57	0.93	0.459	0.65	-0.62	No_change
Pp05_31752-3p	37	16	11	49	15	27	7.03	3.67	3.31	7.4	3.37	6.25	0.307	1.31	0.39	No_change
Pp05_31752-5p	1	0	1	0	0	0	0.19	0	0.3	0	0	0	0.521	8.62	3.11	No_change
Pp06_32153-3p	405	144	141	663	328	298	76.95	33.05	42.41	100.15	73.63	68.96	0.995	1	0	No_change
Pp06_32860-5p	41	5	8	47	27	34	7.79	1.15	2.41	7.1	6.06	7.87	0.306	0.78	-0.36	No_change
Pp06_33150-5p(gma-miR4364a)	9	3	3	9	5	6	1.71	0.69	0.9	1.36	1.12	1.39	0.474	1.34	0.42	No_change
Pp06_33255-3p(nta-miR6155)	30	7	11	52	17	26	5.7	1.61	3.31	7.85	3.82	6.02	0.8	0.92	-0.13	No_change
Pp06_33255-5p	4	0	0	4	0	3	0.76	0	0	0.6	0	0.69	0.76	0.93	-0.11	No_change
Pp06_33517-3p(stu-miR8016)	8	2	3	13	4	10	1.52	0.46	0.9	1.96	0.9	2.31	0.87	0.86	-0.21	No_change
Pp06_33517-5p	0	2	0	0	0	0	0	0.46	0	0	0	0	0.321	10.8	3.43	No_change
Pp06_33967-3p	63	12	13	82	47	20	11.97	2.75	3.91	12.39	10.55	4.63	1	1	0.01	No_change
Pp06_34002-5p(gma-miR166m)	99	25	36	78	44	41	18.81	5.74	10.83	11.78	9.88	9.49	0.015	1.72	0.79	No_change
Pp06_34059-3p(ptc-miR319i)	115	22	35	132	66	46	21.85	5.05	10.53	19.94	14.82	10.64	0.395	1.23	0.3	No_change
Pp06_34158-3p	22	5	7	18	10	10	4.18	1.15	2.11	2.72	2.24	2.31	0.166	1.57	0.65	No_change
Pp06_34158-5p	52	4	6	57	19	8	9.88	0.92	1.8	8.61	4.27	1.85	0.725	1.18	0.24	No_change
Pp06_35078-5p	44	25	23	81	26	51	8.36	5.74	6.92	12.24	5.84	11.8	0.508	1.17	0.22	No_change
Pp06_35148-3p	18	2	2	6	4	4	3.42	0.46	0.6	0.91	0.9	0.93	0.048	2.57	1.36	Up_regulate
Pp06_35148-5p	87	6	12	13	6	11	16.53	1.38	3.61	1.96	1.35	2.55	0	4.75	2.25	Up_regulate
Pp06_35594-3p	25	9	17	35	17	24	4.75	2.07	5.11	5.29	3.82	5.55	0.398	1.27	0.34	No_change
Pp06_35755-5p(bdi-miR7711-3p.4)	30	6	5	27	16	11	5.7	1.38	1.5	4.08	3.59	2.55	0.565	1.27	0.35	No_change
Pp06_35902-3p	35	14	18	28	38	32	6.65	3.21	5.41	4.23	8.53	7.4	0.608	1.18	0.23	No_change
Pp06_36049-5p	6	2	3	18	9	11	1.14	0.46	0.9	2.72	2.02	2.55	0.133	0.53	-0.93	No_change
Pp06_36107-5p(ath-miR5648-5p)	7	1	4	11	6	6	1.33	0.23	1.2	1.66	1.35	1.39	1	0.94	-0.09	No_change
Pp06_38342-3p	13	6	8	21	12	12	2.47	1.38	2.41	3.17	2.69	2.78	0.681	1.11	0.15	No_change
Pp07_40371-3p(ath-miR5012)	1	3	0	5	2	3	0.19	0.69	0	0.76	0.45	0.69	1	0.8	-0.32	No_change
Pp07_40606-5p	5	4	0	14	5	4	0.95	0.92	0	2.11	1.12	0.93	0.586	0.73	-0.46	No_change
Pp07_40735-3p	847	383	340	1150	589	505	160.93	87.9	102.27	173.71	132.22	116.86	0.105	1.38	0.47	No_change
Pp07_40735-5p	19	1	5	39	22	15	3.61	0.23	1.5	5.89	4.94	3.47	0.062	0.55	-0.85	No_change
Pp07_41762-3p	14	9	4	25	6	12	2.66	2.07	1.2	3.78	1.35	2.78	0.498	1.21	0.28	No_change
Pp07_42551-5p(bdi-miR7740-5p)	8	1	0	12	3	2	1.52	0.23	0	1.81	0.67	0.46	0.835	0.93	-0.1	No_change
Pp07_43050-3p	6	2	2	2	5	6	1.14	0.46	0.6	0.3	1.12	1.39	0.839	1.33	0.41	No_change
Pp07_43490-3p(mtr-miR5292b)	24	12	6	25	10	9	4.56	2.75	1.8	3.78	2.24	2.08	0.059	1.8	0.85	No_change
Pp07_43526-3p(gra-miR7502e)	59	9	17	75	33	39	11.21	2.07	5.11	11.33	7.41	9.02	0.894	0.98	-0.04	No_change

Pp07_43526-5p	1	0	0	2	3	1	0.19	0	0	0.3	0.67	0.23	0.399	0.34	-1.57	No_change
Pp08_43695-3p	11	4	13	23	9	20	2.09	0.92	3.91	3.47	2.02	4.63	0.834	1.07	0.1	No_change
Pp08_43695-5p	4	0	1	5	3	1	0.76	0	0.3	0.76	0.67	0.23	1	0.99	-0.02	No_change
Pp08_44101-3p	67	25	23	77	35	62	12.73	5.74	6.92	11.63	7.86	14.35	0.513	1.17	0.23	No_change
Pp08_44101-5p	18	3	3	20	8	9	3.42	0.69	0.9	3.02	1.8	2.08	0.896	1.14	0.19	No_change
Pp08_44196-5p(stu-miR396-3p)	17	7	3	12	10	14	3.23	1.61	0.9	1.81	2.24	3.24	0.598	1.26	0.34	No_change
Pp08_44917-3p(gma-miR9724)	106	49	39	129	71	57	20.14	11.25	11.73	19.49	15.94	13.19	0.092	1.45	0.54	No_change
