# Supplementary material for: Identification and characterization of Prunus persica miRNAs in response to UVB radiation in greenhouse through high-throughput sequencing
Source: BMC Genomics. 2017 Dec 2;18:938. doi: 10.1186/s12864-017-4347-5 (PMC5712094; doi:10.1186/s12864-017-4347-5)
Supplement: Supplementary file 3 — Details of targets genes and their annotation, GO classification, and KEGG pathway for the known miRNAs. (PDF 2499 kb) [file 12864_2017_4347_MOESM3_ESM.pdf]

#microRNA	gene_id	gene_name	genome_version	scaffold_id	strand	start_position	end_position	length	Pathway - KEGG PATHWAY	GO - Biological Process	GO - Molecular Function	GO - Cellular Component
ppe-miR171d-3p	18766524	PRUPE_ppa018405mg	NCBI_Assembly:GCF_000346465.:	NW_006760194.1	-	1225235	1226474	1239	-	-	-	-
ppe-miR171d-3p	18766953	PRUPE_ppb020334mg	NCBI_Assembly:GCF_000346465.:	NW_006760194.1	+	16975136	16977400	2264	-	-	-	-
ppe-miR171d-3p	18767514	PRUPE_ppa017727mg	NCBI_Assembly:GCF_000346465.:	NW_006760194.1	-	2681925	2684641	2716	-	-	-	-
ppe-miR171d-3p	18767749	PRUPE_ppa011579mg	NCBI_Assembly:GCF_000346465.:	NW_006760194.1	+	4562222	4565892	3670	pper04144:Endocytosis;pper04145:Phagosome	GO:0007264: small GTPase mediated signal transduction; GO:0015031: protein transport;GO: 0016192:vesic[illegible]	GO:0005525: GTP binding	GO:0005774:vacuolar membrane;GO:0005794:Golgi apparatus
ppe-miR171d-3p	18767992	PRUPE_ppa022584mg	NCBI_Assembly:GCF_000346465.:	NW_006760194.1	-	6589904	6592153	2249	-	-	GO:0003676: nucleic acid binding;GO:0008270:zinc ion binding	-
ppe-miR171d-3p	18768760	PRUPE_ppa026391mg	NCBI_Assembly:GCF_000346465.:	NW_006760194.1	-	11362565	11363292	727	-	-	GO:0003700: sequence-specific DNA binding transcription factor activity;GO:0008270:zinc ion binding	GO:0005622:intracellular
ppe-miR171d-3p	18768844	PRUPE_ppa023574mg	NCBI_Assembly:GCF_000346465.:	NW_006760194.1	-	2794315	2795246	931	-	-	-	-
ppe-miR171d-3p	18769216	PRUPE_ppa016545mg	NCBI_Assembly:GCF_000346465.:	NW_006760201.1	-	18235735	18236199	464	pper03010:Ribosome	GO:0006412:translation	GO:0003735: structural constituent of ribosome	GO:0005840:ribosome

ppe-miR171d-3p	18769536	PRUPE_ppa023521mg	NCBI_Assembly:GCF_000346465.:	NW_006760201.1	-	15315096	15316845	1749	pper01110:Biosynthesis of secondary metabolites;pper00460:Cyanoamino acid metabolism;pper01210:2-Oxocarboxylic acid metabolism;pper00966:Glucosinolate biosynthesis	-	GO:0004497: monooxygenase activity;GO:0005506:iron ion binding;GO:0016705:oxidoreductase activity, acting on paired donors, with incorporation or reduction of molecular oxygen;GO:0020037:heme binding	-
ppe-miR171d-3p	18769600	PRUPE_ppa001624mg	NCBI_Assembly:GCF_000346465.:	NW_006760201.1	-	17709395	17716228	6833	-	GO:0015031: protein transport;GO:0042147:retrograde transport, endosome to Golgi	-	GO:0005622:intracellular;GO:0030904:retromer complex
ppe-miR171d-3p	18769672	PRUPE_ppa019635mg	NCBI_Assembly:GCF_000346465.:	NW_006760201.1	+	12832319	12835000	2681	-	-	-	-
ppe-miR171d-3p	18769820	PRUPE_ppa022872mg	NCBI_Assembly:GCF_000346465.:	NW_006760201.1	-	21044777	21046996	2219	-	GO:0031425: chloroplast RNA processing	-	-
ppe-miR171d-3p	18771321	PRUPE_ppa018440mg	NCBI_Assembly:GCF_000346465.:	NW_006760201.1	+	15778418	15779320	902	-	-	GO:0016491: oxidoreductase activity	-
ppe-miR171d-3p	18772638	PRUPE_ppa020201mg	NCBI_Assembly:GCF_000346465.:	NW_006760208.1	-	11290131	11290442	311	-	-	-	-

ppe-miR171d-3p	18773196	PRUPE_ppa000881mg	NCBI_Assembly:GCF_000346465.:	NW_006760208.1	+	27480245	27489756	9511	pper03008:Ribosome biogenesis in eukaryotes;pper03018:RNA degradation	GO:0000291: nuclear-transcribed mRNA catabolic process, exonucleolytic;GO:0006306: DNA methylation;GO:0006342:chromatin silencing;GO:0006487:protein N-linked glycosylation;GO:0007131:reciprocal meiotic recombination;GO:0007155: cell adhesion;GO:0007267:cell-cell signaling;GO:0008284:positive regulation of cell proliferation;GO:0009616:virus induced gene silencing;GO:0009630:gravitropism;GO:0009826:unidimensional cell growth;GO:00	GO:0003676: nucleic acid binding;GO:0004527:exonuclease activity;GO:0008270:zinc ion binding	GO:0005829:cytosol
ppe-miR171d-3p	18773996	PRUPE_ppa013022mg	NCBI_Assembly:GCF_000346465.:	NW_006760208.1	+	4524344	4525074	730	pper04141:Protein processing in endoplasmic reticulum	GO:0006979:response to oxidative stress	-	-
ppe-miR171d-3p	18774511	PRUPE_ppa003378mg	NCBI_Assembly:GCF_000346465.:	NW_006760208.1	-	21939965	21945154	5189	-	-	-	-

ppe-miR171d-3p	18774770	PRUPE_ppa014622mg	NCBI_Assembly:GCF_000346465.:	NW_006760208.1	-	23576710	23578784	2074	-	-	GO:0001104: RNA polymerase II transcription cofactor activity	GO:0016592:mediator complex
ppe-miR171d-3p	18775624	PRUPE_ppa021656mg	NCBI_Assembly:GCF_000346465.:	NW_006760208.1	-	20424251	20424690	439	-	-	-	-
ppe-miR171d-3p	18776779	PRUPE_ppa001305mg	NCBI_Assembly:GCF_000346465.:	NW_006760212.1	+	3610819	3617180	6361	pper01100:Metabolic pathways;pper00500:Starch and sucrose metabolism	GO:0000023: maltose metabolic process;GO:0005992:trehalose biosynthetic process;GO:0019252:starch biosynthetic process;GO:0043085:positive regulation of catalytic activity	GO:0003824: catalytic activity	-
ppe-miR171d-3p	18778483	PRUPE_ppa026799mg	NCBI_Assembly:GCF_000346465.:	NW_006760220.1	-	1229201	1231172	1971	-	GO:0009685: gibberellin metabolic process	GO:0046872: metal ion binding;GO:0052635:C-20 gibberellin 2-beta-dioxygenase activity	-
ppe-miR171d-3p	18779131	PRUPE_ppa004994mg	NCBI_Assembly:GCF_000346465.:	NW_006760220.1	+	1070519	1072026	1507	-	-	GO:0003677: DNA binding	-
ppe-miR171d-3p	18779691	PRUPE_ppa016668mg	NCBI_Assembly:GCF_000346465.:	NW_006760220.1	+	20615857	20616581	724	-	-	-	-
ppe-miR171d-3p	18779786	PRUPE_ppa026693mg	NCBI_Assembly:GCF_000346465.:	NW_006760220.1	-	12719525	12720163	638	-	-	-	-
ppe-miR171d-3p	18780017	PRUPE_ppa021617mg	NCBI_Assembly:GCF_000346465.:	NW_006760220.1	-	22906603	22907646	1043	-	-	-	-
ppe-miR171d-3p	18781880	PRUPE_ppa022099mg	NCBI_Assembly:GCF_000346465.:	NW_006760268.1	+	15446915	15447581	666	-	GO:0055085:transmembrane transport	-	-
ppe-miR171d-3p	18782436	PRUPE_ppa023261mg	NCBI_Assembly:GCF_000346465.:	NW_006760268.1	-	5615523	5616637	1114	-	-	-	-
ppe-miR171d-3p	18783589	PRUPE_ppa015038mg	NCBI_Assembly:GCF_000346465.:	NW_006760268.1	+	17795288	17795677	389	-	-	-	-

ppe-miR171d-3p	18783688	PRUPE_ppa020904mg	NCBI_Assembly:GCF_000346465.:	NW_006760268.1	+	21105725	21107867	2142	-	-	GO:0046872: metal ion binding	-
ppe-miR171d-3p	18784568	PRUPE_ppa011162mg	NCBI_Assembly:GCF_000346465.:	NW_006760324.1	-	13752300	13753142	842	-	-	-	-
ppe-miR171d-3p	18786225	PRUPE_ppb015375mg	NCBI_Assembly:GCF_000346465.:	NW_006760324.1	-	4817171	4817389	218	-	-	-	-
ppe-miR171d-3p	18786500	PRUPE_ppb012804mg	NCBI_Assembly:GCF_000346465.:	NW_006760324.1	-	5359111	5359329	218	-	-	-	-
ppe-miR171d-3p	18786649	PRUPE_ppa002407mg	NCBI_Assembly:GCF_000346465.:	NW_006760324.1	+	1204329	1217078	12749	pper03440:Homologous recombination	GO:0006259: DNA metabolic process	GO:0003677: DNA binding;GO:0004518:nuclease activity	-
ppe-miR171d-3p	18786766	PRUPE_ppa005730mg	NCBI_Assembly:GCF_000346465.:	NW_006760324.1	+	24573621	24576442	2821	-	-	GO:0004222: metalloendopeptidase activity	GO:0016020:membrane
ppe-miR171d-3p	18786811	PRUPE_ppa024059mg	NCBI_Assembly:GCF_000346465.:	NW_006760324.1	-	12168605	12169146	541	-	-	GO:0008270: zinc ion binding	-
ppe-miR171d-3p	18787049	PRUPE_ppa003083mg	NCBI_Assembly:GCF_000346465.:	NW_006760324.1	+	17995363	17997462	2099	-	-	-	-
ppe-miR171d-3p	18787507	PRUPE_ppa006364mg	NCBI_Assembly:GCF_000346465.:	NW_006760324.1	-	15213941	15220956	7015	pper04141:Protein processing in endoplasmic reticulum	GO:0006457: protein folding;GO:0009408:response to heat	GO:0005524: ATP binding;GO:0046872:metal ion binding	-
ppe-miR171d-3p	18787949	PRUPE_ppb024412mg	NCBI_Assembly:GCF_000346465.:	NW_006760376.1	-	74214	77918	3704	-	-	-	-
ppe-miR171d-3p	18788168	PRUPE_ppa003757mg	NCBI_Assembly:GCF_000346465.:	NW_006760385.1	+	7484393	7491635	7242	-	GO:0051604: protein maturation	GO:0046872: metal ion binding	-
ppe-miR171d-3p	18788602	PRUPE_ppa008082mg	NCBI_Assembly:GCF_000346465.:	NW_006760385.1	+	43764550	43766016	1466	-	-	GO:0000179:rRNA (adenine-N6,N6-)-dimethyltransferase activity	-

ppe-miR171d-3p	18789423	PRUPE_ppa000676mg	NCBI_Assembly:GCF_000346465.:	NW_006760385.1	-	37064902	37070931	6029	-	GO:0000280:nuclear division;GO:0000911:cytokinesis by cell plate formation;GO:0009744:response to sucrose;GO:0009749:response to glucose;GO:0009750:response to fructose;GO:0010389:regulation of G2/M transition of mitotic cell cycle;GO:0032875:regulation of DNA endoreduplication;GO:0042023:DNA endoreduplication;GO:0048451:petal formation;GO:0048453:sepal formation;GO:0051225:spindle assembly	GO:0003677:DNA binding;GO:0003682:chromatin binding;GO:0003700:sequence-specific DNA binding transcription factor activity;GO:0003713:transcription coactivator activity	-
ppe-miR171d-3p	18789550	PRUPE_ppa002170mg	NCBI_Assembly:GCF_000346465.:	NW_006760385.1	+	6817159	6821856	4697	-	-	GO:0008234:cysteine-type peptidase activity	-

ppe-miR171d-3p	18790118	PRUPE_ppa010539mg	NCBI_Assembly:GCF_000346465.:	NW_006760385.1	+	6517629	6523766	6137	-	GO:0007264: small GTPase mediated signal transduction; GO:0030244: cellulose biosynthetic process;GO:0048193:Golgi vesicle transport	GO:0005525: GTP binding	GO:0005795:Golgi stack;GO:0005886:plasma membrane
ppe-miR171d-3p	18790208	PRUPE_ppa018317mg	NCBI_Assembly:GCF_000346465.:	NW_006760385.1	-	32554859	32558067	3208	-	-	-	-
ppe-miR171d-3p	18790570	PRUPE_ppa011766mg	NCBI_Assembly:GCF_000346465.:	NW_006760385.1	+	42088447	42093387	4940	pper00350:Tyrosine metabolism;pper01100:Metabolic pathways	-	-	-
ppe-miR171d-3p	18791066	PRUPE_ppa003031mg	NCBI_Assembly:GCF_000346465.:	NW_006760385.1	+	27755997	27760130	4133	-	-	GO:0003993: acid phosphatase activity;GO:0046872:metal ion binding	-
ppe-miR171d-3p	18791073	PRUPE_ppa004132mg	NCBI_Assembly:GCF_000346465.:	NW_006760385.1	-	11903999	11909035	5036	-	-	GO:0022891: substrate-specific transmembrane transporter activity	GO:0016021:integral component of membrane
ppe-miR171d-3p	18791103	PRUPE_ppa016031mg	NCBI_Assembly:GCF_000346465.:	NW_006760385.1	+	25794247	25796117	1870	-	-	GO:0022857:transmembrane transporter activity	GO:0016021:integral component of membrane

ppe-miR171d-3p	18791111	PRUPE_ppa012111mg	NCBI_Assembly:GCF_000346465.:	NW_006760385.1	-	25649119	25650741	1622	pper03040:Spliceosome	GO:0000278: mitotic cell cycle;GO:0000398:mRNA splicing, via spliceosome;GO:0030422: production of siRNA involved in RNA interference;GO:0035196:production of miRNAs involved in gene silencing by miRNA;GO:0043687:post-translational protein modification;GO:0045893:positive regulation of transcription, DNA-templated;GO:0048573:photoperiodism, flowering	GO:0000166: nucleotide binding;GO:0003676:nucleic acid binding;GO:0008270:zinc ion binding	-
ppe-miR171d-3p	18791889	PRUPE_ppa016436mg	NCBI_Assembly:GCF_000346465.:	NW_006760385.1	+	14933644	14936216	2572	-	-	GO:0001104: RNA polymerase II transcription cofactor activity	GO:0016592:mediator complex
ppe-miR171d-3p	18792379	PRUPE_ppa026129mg	NCBI_Assembly:GCF_000346465.:	NW_006760385.1	+	12039101	12040373	1272	-	-	-	-
ppe-miR171d-3p	18794050	PRUPE_ppa014499mg	NCBI_Assembly:GCF_000346465.:	NW_006760385.1	+	31243568	31244167	599	-	-	-	-
ppe-miR3627-5p	18766006	PRUPE_ppb020037mg	NCBI_Assembly:GCF_000346465.:	NW_006760186.1	-	1240782	1245272	4490	-	GO:0015074: DNA integration	GO:0003676: nucleic acid binding	-
ppe-miR3627-5p	18766107	PRUPE_ppa016845mg	NCBI_Assembly:GCF_000346465.:	NW_006760186.1	+	2064836	2065417	581	-	-	-	-

ppe-miR3627-5p	18766156	PRUPE_ppa024570mg	NCBI_Assembly:GCF_000346465.:	NW_006760194.1	-	13427315	13430196	2881	-	-	-	-
ppe-miR3627-5p	18766164	PRUPE_ppa016093mg	NCBI_Assembly:GCF_000346465.:	NW_006760194.1	+	5983601	5988590	4989	-	-	GO:0003676: nucleic acid binding	-
ppe-miR3627-5p	18766270	PRUPE_ppa008449mg	NCBI_Assembly:GCF_000346465.:	NW_006760194.1	-	17728807	17730539	1732	-	GO:0042761: very long-chain fatty acid biosynthetic process	GO:0003723: RNA binding;GO:0050291:sphingosine N-acyltransferase activity	-
ppe-miR3627-5p	18766328	PRUPE_ppb022581mg	NCBI_Assembly:GCF_000346465.:	NW_006760194.1	-	13998436	13999383	947	-	-	GO:0016787: hydrolase activity	-
ppe-miR3627-5p	18766414	PRUPE_ppa015544mg	NCBI_Assembly:GCF_000346465.:	NW_006760194.1	-	12017538	12019869	2331	-	GO:0046274:lignin catabolic process	GO:0005507: copper ion binding;GO:0052716:hydroquinone:oxygen oxidoreductase activity	GO:0048046:apoplast
ppe-miR3627-5p	18766454	PRUPE_ppa008938mg	NCBI_Assembly:GCF_000346465.:	NW_006760194.1	-	16625040	16627124	2084	-	GO:0015996: chlorophyll catabolic process	-	GO:0016020:membrane
ppe-miR3627-5p	18766610	PRUPE_ppb023070mg	NCBI_Assembly:GCF_000346465.:	NW_006760194.1	-	2512071	2514781	2710	-	-	-	-
ppe-miR3627-5p	18766623	PRUPE_ppa024176mg	NCBI_Assembly:GCF_000346465.:	NW_006760194.1	-	139674	140014	340	-	-	-	-
ppe-miR3627-5p	18766739	PRUPE_ppb021369mg	NCBI_Assembly:GCF_000346465.:	NW_006760194.1	-	21111851	21112887	1036	-	-	-	-
ppe-miR3627-5p	18766751	PRUPE_ppa014891mg	NCBI_Assembly:GCF_000346465.:	NW_006760194.1	-	17036459	17036857	398	-	-	-	-
ppe-miR3627-5p	18766835	PRUPE_ppa024736mg	NCBI_Assembly:GCF_000346465.:	NW_006760194.1	-	7379503	7379852	349	-	GO:0006351:transcription, DNA-templated;GO:0006355:regulation of transcription, DNA-templated	GO:0003677: DNA binding	GO:0005634:nucleus

ppe-miR3627-5p	18766860	PRUPE_ppa021045mg	NCBI_Assembly:GCF_000346465.:	NW_006760194.1	-	12816038	12816865	827	-	GO:0006355:regulation of transcription, DNA-templated;GO:0016556:mRNA modification	GO:0003690:double-stranded DNA binding	GO:0005739:mitochondrion;GO:0009507:chloroplast
ppe-miR3627-5p	18766861	PRUPE_ppa000330mg	NCBI_Assembly:GCF_000346465.:	NW_006760194.1	+	10218653	10225775	7122	-	GO:0006351:transcription, DNA-templated;GO:0009165:nucleotide biosynthetic process	GO:0003677:DNA binding;GO:0003887:DNA-directed DNA polymerase activity	GO:0005829:cytosol
ppe-miR3627-5p	18766875	PRUPE_ppa021228mg	NCBI_Assembly:GCF_000346465.:	NW_006760194.1	+	12535762	12536411	649	-	-	-	-
ppe-miR3627-5p	18766950	PRUPE_ppa023428mg	NCBI_Assembly:GCF_000346465.:	NW_006760194.1	+	15133440	15137225	3785	pper01100:Metabolic pathways;pper00330:Arginine and proline metabolism	GO:0031348:negative regulation of defense response	GO:0005506:iron ion binding;GO:0016706:oxidoreductase activity, acting on paired donors, with incorporation or reduction of molecular oxygen, 2-oxoglutarate as one donor, and incorporation of one atom each of oxygen into both donors;GO:0031418:L-ascorbic acid binding	GO:0005794:Golgi apparatus

ppe-miR3627-5p	18766999	PRUPE_ppa019107mg	NCBI_Assembly:GCF_000346465.:	NW_006760194.1	+		12987894	12988664	770	-	-	GO:0008270: zinc ion binding	-
ppe-miR3627-5p	18767014	PRUPE_ppa021781mg	NCBI_Assembly:GCF_000346465.:	NW_006760194.1	-		15734501	15737265	2764	-	-	-	-
ppe-miR3627-5p	18767162	PRUPE_ppa005275mg	NCBI_Assembly:GCF_000346465.:	NW_006760194.1	+		11509666	11513152	3486	-	-	GO:0003993: acid phosphatase activity;GO:0046872:metal ion binding	-
ppe-miR3627-5p	18767201	PRUPE_ppa001167mg	NCBI_Assembly:GCF_000346465.:	NW_006760194.1	-		14516906	14520665	3759	-	GO:0006612: protein targeting to membrane;GO:0009963:positive regulation of flavonoid biosynthetic process;GO:0010363:regulation of plant-type hypersensitive response	-	GO:0005622:intracellular;GO:0005886:plasma membrane
ppe-miR3627-5p	18767257	PRUPE_ppa023069mg	NCBI_Assembly:GCF_000346465.:	NW_006760194.1	-		8196066	8200497	4431	-	-	-	-
ppe-miR3627-5p	18767277	PRUPE_ppa014595mg	NCBI_Assembly:GCF_000346465.:	NW_006760194.1	+		12311873	12312839	966	-	-	-	-
ppe-miR3627-5p	18767382	PRUPE_ppa007505mg	NCBI_Assembly:GCF_000346465.:	NW_006760194.1	-		17405876	17408903	3027	-	-	-	-
ppe-miR3627-5p	18767471	PRUPE_ppa006488mg	NCBI_Assembly:GCF_000346465.:	NW_006760194.1	-		20595430	20598812	3382	-	-	-	-
ppe-miR3627-5p	18767556	PRUPE_ppa003272mg	NCBI_Assembly:GCF_000346465.:	NW_006760194.1	-		15971865	15974528	2663	-	-	GO:0003676: nucleic acid binding;GO:0008270:zinc ion binding	-
ppe-miR3627-5p	18767683	PRUPE_ppa003726mg	NCBI_Assembly:GCF_000346465.:	NW_006760194.1	-		18889322	18892209	2887	-	-	-	-
ppe-miR3627-5p	18767784	PRUPE_ppa015444mg	NCBI_Assembly:GCF_000346465.:	NW_006760194.1	-		14717061	14719757	2696	pper04626:Plant-pathogen interaction	-	GO:0043531: ADP binding	-
ppe-miR3627-5p	18767814	PRUPE_ppa022383m1g	NCBI_Assembly:GCF_000346465.:	NW_006760194.1	-		7861693	7864646	2953	-	-	GO:0008234: cysteine-type peptidase activity	-
ppe-miR3627-5p	18767847	PRUPE_ppa022566mg	NCBI_Assembly:GCF_000346465.:	NW_006760194.1	-		19440096	19440452	356	-	-	-	-

ppe-miR3627-5p	18767897	PRUPE_ppa007085mg	NCBI_Assembly:GCF_000346465.:	NW_006760194.1	+	20350316	20351625	1309	-	GO:0010075:regulation of meristem growth;GO:0016042:lipid catabolic process	GO:0016787: hydrolase activity	-
ppe-miR3627-5p	18767900	PRUPE_ppa018802mg	NCBI_Assembly:GCF_000346465.:	NW_006760194.1	-	13357466	13357759	293	-	-	-	-
ppe-miR3627-5p	18767938	PRUPE_ppb022436mg	NCBI_Assembly:GCF_000346465.:	NW_006760194.1	+	19021683	19022779	1096	-	-	-	-
ppe-miR3627-5p	18768079	PRUPE_ppa025016mg	NCBI_Assembly:GCF_000346465.:	NW_006760194.1	+	13053405	13054471	1066	-	-	GO:0003677: DNA binding	GO:0000786:nucleosome;GO:0005634:nucleus
ppe-miR3627-5p	18768328	PRUPE_ppa001199mg	NCBI_Assembly:GCF_000346465.:	NW_006760194.1	+	20558073	20560724	2651	-	GO:0000023: maltose metabolic process;GO:0010021:amylopectin biosynthetic process;GO:0019761:glucosinolate biosynthetic process;GO:0043085:positive regulation of catalytic activity	GO:0019156:isoamylase activity;GO:0043169:cation binding	-
ppe-miR3627-5p	18768401	PRUPE_ppa004593mg	NCBI_Assembly:GCF_000346465.:	NW_006760194.1	-	19256066	19261310	5244	-	GO:0010038:response to metal ion;GO:0046938:phytochelatin biosynthetic process	GO:0016756: glutathione gamma-glutamylcysteinyltransferase activity;GO:0046872:metal ion binding	-
ppe-miR3627-5p	18768421	PRUPE_ppa026529mg	NCBI_Assembly:GCF_000346465.:	NW_006760194.1	-	3182172	3185993	3821	-	GO:0007165: signal transduction	GO:0043531: ADP binding	-
ppe-miR3627-5p	18768504	PRUPE_ppa024462mg	NCBI_Assembly:GCF_000346465.:	NW_006760194.1	+	17586977	17591118	4141	-	GO:0007165: signal transduction	GO:0043531: ADP binding	-

ppe-miR3627-5p	18768521	PRUPE_ppa002658mg	NCBI_Assembly:GCF_000346465.:	NW_006760194.1	+	17197789	17200357	2568	-	-	GO:0004842: ubiquitin-protein transferase activity;GO:0016874:ligase activity	-
ppe-miR3627-5p	18768528	PRUPE_ppa015646mg	NCBI_Assembly:GCF_000346465.:	NW_006760194.1	+	7728756	7731632	2876	-	GO:0000023: maltose metabolic process;GO:0006655:phosphatidylglycerol biosynthetic process;GO:0009902:chloroplast relocation;GO:0010027:thylakoid membrane organization; GO:0019252: starch biosynthetic process;GO:0019288:isopentenyl diphosphate biosynthetic process, methylerythritol 4-phosphate pathway;GO:0034660:ncRNA metabolic process	GO:0005525: GTP binding	GO:0005623:cell
ppe-miR3627-5p	18768569	PRUPE_ppa006662mg	NCBI_Assembly:GCF_000346465.:	NW_006760194.1	-	20926679	20928999	2320	-	-	-	-
ppe-miR3627-5p	18768599	PRUPE_ppa010243mg	NCBI_Assembly:GCF_000346465.:	NW_006760194.1	-	20378636	20381143	2507	-	-	-	-

ppe-miR3627-5p	18768632	PRUPE_ppa017679mg	NCBI_Assembly:GCF_000346465.:	NW_006760194.1	-	11037074	11042272	5198	pper03008:Ribosome biogenesis in eukaryotes	-	-	-
ppe-miR3627-5p	18768643	PRUPE_ppa016705mg	NCBI_Assembly:GCF_000346465.:	NW_006760194.1	+	18678595	18680442	1847	-	GO:0009560: embryo sac egg cell differentiation	-	-
ppe-miR3627-5p	18768940	PRUPE_ppa016623mg	NCBI_Assembly:GCF_000346465.:	NW_006760194.1	+	403648	409247	5599	-	GO:0007165: signal transduction	GO:0005524: ATP binding;GO:0043531:ADP binding	-
ppe-miR3627-5p	18769067	PRUPE_ppa019028mg	NCBI_Assembly:GCF_000346465.:	NW_006760201.1	-	19045307	19047038	1731	-	-	GO:0004672: protein kinase activity;GO:0005524:ATP binding	-
ppe-miR3627-5p	18769072	PRUPE_ppa003446mg	NCBI_Assembly:GCF_000346465.:	NW_006760201.1	-	18709219	18713135	3916	-	GO:0008380: RNA splicing	-	GO:0005634:nucleus
ppe-miR3627-5p	18769137	PRUPE_ppa011232mg	NCBI_Assembly:GCF_000346465.:	NW_006760201.1	+	19919190	19922228	3038	pper04144:Endocytosis	GO:0007034: vacuolar transport	-	GO:0005622:intracellular
ppe-miR3627-5p	18769171	PRUPE_ppa014118mg	NCBI_Assembly:GCF_000346465.:	NW_006760201.1	+	3521869	3522862	993	-	-	-	-
ppe-miR3627-5p	18769261	PRUPE_ppb016299mg	NCBI_Assembly:GCF_000346465.:	NW_006760201.1	+	11515913	11516741	828	-	-	-	-
ppe-miR3627-5p	18769293	PRUPE_ppa010251mg	NCBI_Assembly:GCF_000346465.:	NW_006760201.1	-	19786201	19788526	2325	pper01100:Metabolic pathways;pper00750:Vitamin B6 metabolism	GO:0051262: protein tetramerization	GO:0016791: phosphatase activity	-
ppe-miR3627-5p	18769301	PRUPE_ppa013582mg	NCBI_Assembly:GCF_000346465.:	NW_006760201.1	+	22615011	22616734	1723	-	-	-	-
ppe-miR3627-5p	18769367	PRUPE_ppa000657mg	NCBI_Assembly:GCF_000346465.:	NW_006760201.1	-	18585286	18593095	7809	pper01100:Metabolic pathways;pper01110:Biosynthesis of secondary metabolites;pper00310:Lysine degradation	-	GO:0016491: oxidoreductase activity	-

ppe-miR3627-5p	18769403	PRUPE_ppa015510mg	NCBI_Assembly:GCF_000346465.:	NW_006760201.1	-	7719922	7723180	3258	-	GO:0010264: myo-inositol hexakisphosphate biosynthetic process	GO:0016844: strictosidine synthase activity	-
ppe-miR3627-5p	18769465	PRUPE_ppa000725mg	NCBI_Assembly:GCF_000346465.:	NW_006760201.1	-	9835321	9845976	10655	-	-	-	-
ppe-miR3627-5p	18769518	PRUPE_ppa013723mg	NCBI_Assembly:GCF_000346465.:	NW_006760201.1	-	16531153	16532863	1710	pper03040:Spliceosome	GO:0008380: RNA splicing	-	GO:0030532:small nuclear ribonucleoprotein complex
ppe-miR3627-5p	18769595	PRUPE_ppa012033mg	NCBI_Assembly:GCF_000346465.:	NW_006760201.1	-	15444568	15446649	2081	-	-	GO:0008171: O-methyltransferase activity	-
ppe-miR3627-5p	18769605	PRUPE_ppa017748mg	NCBI_Assembly:GCF_000346465.:	NW_006760201.1	+	19169636	19171818	2182	pper01100:Metabolic pathways;pper00230:Purine metabolism;pper00240:Pyrimidine metabolism;pper03020:RNA polymerase	GO:0006383:transcription from RNA polymerase III promoter	GO:0003677: DNA binding;GO:0003899:DNA-directed RNA polymerase activity	GO:0005666:DNA-directed RNA polymerase III complex
ppe-miR3627-5p	18769613	PRUPE_ppb024225mg	NCBI_Assembly:GCF_000346465.:	NW_006760201.1	+	18801341	18802900	1559	-	-	-	-
ppe-miR3627-5p	18769706	PRUPE_ppa019287mg	NCBI_Assembly:GCF_000346465.:	NW_006760201.1	-	20214947	20217992	3045	-	GO:0006355:regulation of transcription, DNA-templated	GO:0003690: double-stranded DNA binding	GO:0005739:mitochondrion
ppe-miR3627-5p	18769737	PRUPE_ppa009376mg	NCBI_Assembly:GCF_000346465.:	NW_006760201.1	-	22066184	22067371	1187	pper03018:RNA degradation	-	GO:0003676: nucleic acid binding	GO:0005634:nucleus
ppe-miR3627-5p	18769765	PRUPE_ppa011922mg	NCBI_Assembly:GCF_000346465.:	NW_006760201.1	+	18179453	18180885	1432	pper01100:Metabolic pathways;pper00230:Purine metabolism;pper00730:Thiamine metabolism	-	GO:0005524: ATP binding;GO:0098519:nucleotide phosphatase activity, acting on free nucleotides	-

ppe-miR3627-5p	18769782	PRUPE_ppa008052mg	NCBI_Assembly:GCF_000346465.:	NW_006760201.1	-	20941344	20944756	3412	pper03013:RNA transport	-	-	-
ppe-miR3627-5p	18769820	PRUPE_ppa022872mg	NCBI_Assembly:GCF_000346465.:	NW_006760201.1	-	21044777	21046996	2219	-	GO:0031425: chloroplast RNA processing	-	-
ppe-miR3627-5p	18769848	PRUPE_ppa009206mg	NCBI_Assembly:GCF_000346465.:	NW_006760201.1	+	20623541	20626156	2615	pper04144:Endocytosis;pper04145:Phagosome	GO:0007264: small GTPase mediated signal transduction;GO:0015031: protein transport	GO:0005525: GTP binding	GO:0005622:intracellular
ppe-miR3627-5p	18769849	PRUPE_ppa002244mg	NCBI_Assembly:GCF_000346465.:	NW_006760201.1	-	20046473	20050994	4521	-	-	-	-
ppe-miR3627-5p	18769879	PRUPE_ppb021745mg	NCBI_Assembly:GCF_000346465.:	NW_006760201.1	-	9207786	9208847	1061	-	-	-	-
ppe-miR3627-5p	18769881	PRUPE_ppa021795mg	NCBI_Assembly:GCF_000346465.:	NW_006760201.1	-	1037056	1040520	3464	-	-	-	-
ppe-miR3627-5p	18769901	PRUPE_ppb018294mg	NCBI_Assembly:GCF_000346465.:	NW_006760201.1	-	14611914	14613948	2034	-	-	GO:0004144: diacylglycerol O-acyltransferase activity	-
ppe-miR3627-5p	18770034	PRUPE_ppa014154mg	NCBI_Assembly:GCF_000346465.:	NW_006760201.1	-	11020770	11021449	679	-	-	-	-
ppe-miR3627-5p	18770057	PRUPE_ppa012141mg	NCBI_Assembly:GCF_000346465.:	NW_006760201.1	-	16951048	16953311	2263	-	-	-	GO:0016021:integral component of membrane
ppe-miR3627-5p	18770074	PRUPE_ppa003922mg	NCBI_Assembly:GCF_000346465.:	NW_006760201.1	-	7151466	7156619	5153	-	-	-	GO:0005774:vacuolar membrane;GO:0016021:integral component of membrane
ppe-miR3627-5p	18770085	PRUPE_ppa022516mg	NCBI_Assembly:GCF_000346465.:	NW_006760201.1	-	20105078	20107835	2757	-	-	GO:0004674: protein serine/threonine kinase activity;GO:0005524:ATP binding	-

ppe-miR3627-5p	18770118	PRUPE_ppa012568mg	NCBI_Assembly:GCF_000346465.:	NW_006760201.1	+	4705377	4707026	1649	-	-	GO:0009055:electron carrier activity;GO:0046872:metal ion binding;GO:0051537:2 iron, 2 sulfur cluster binding	-
ppe-miR3627-5p	18770141	PRUPE_ppa015816mg	NCBI_Assembly:GCF_000346465.:	NW_006760201.1	+	20328528	20329642	1114	-	GO:0006351:transcription, DNA-templated;GO:0006355:regulation of transcription, DNA-templated	GO:0003677:DNA binding	GO:0005634:nucleus
ppe-miR3627-5p	18770142	PRUPE_ppa023290mg	NCBI_Assembly:GCF_000346465.:	NW_006760201.1	-	2513368	2513924	556	-	-	-	-
ppe-miR3627-5p	18770191	PRUPE_ppa022316mg	NCBI_Assembly:GCF_000346465.:	NW_006760201.1	-	8662784	8665056	2272	-	-	-	-
ppe-miR3627-5p	18770258	PRUPE_ppa019391mg	NCBI_Assembly:GCF_000346465.:	NW_006760201.1	-	22363069	22365369	2300	-	GO:0007049:cell cycle;GO:0016556:mRNA modification	-	GO:0005634:nucleus
ppe-miR3627-5p	18770266	PRUPE_ppa008223mg	NCBI_Assembly:GCF_000346465.:	NW_006760201.1	-	18355621	18360050	4429	-	GO:0055085:transmembrane transport	-	GO:0016021:integral component of membrane
ppe-miR3627-5p	18770295	PRUPE_ppa020683mg	NCBI_Assembly:GCF_000346465.:	NW_006760201.1	-	17919271	17921446	2175	-	-	-	-
ppe-miR3627-5p	18770338	PRUPE_ppa024920mg	NCBI_Assembly:GCF_000346465.:	NW_006760201.1	+	21926667	21928329	1662	-	-	GO:0005509:calcium ion binding	-
ppe-miR3627-5p	18770440	PRUPE_ppa026685mg	NCBI_Assembly:GCF_000346465.:	NW_006760201.1	+	11079278	11080984	1706	-	-	-	-

ppe-miR3627-5p	18770456	PRUPE_ppa005635mg	NCBI_Assembly:GCF_000346465.:	NW_006760201.1	-	15392669	15394021	1352	-	-	GO:0016747:transferase activity, transferring acyl groups other than amino-acyl groups	-
ppe-miR3627-5p	18770472	PRUPE_ppa008773mg	NCBI_Assembly:GCF_000346465.:	NW_006760201.1	+	20165491	20168796	3305	-	-	GO:0005506:iron ion binding;GO:0016706:oxidoreductase activity, acting on paired donors, with incorporation or reduction of molecular oxygen, 2-oxoglutarate as one donor, and incorporation of one atom each of oxygen into both donors	-
ppe-miR3627-5p	18770882	PRUPE_ppa026570mg	NCBI_Assembly:GCF_000346465.:	NW_006760201.1	-	16298731	16299458	727	-	-	GO:0003676:nucleic acid binding;GO:0046872:metal ion binding	-

ppe-miR3627-5p	18771602	PRUPE_ppa025367mg	NCBI_Assembly:GCF_000346465.:	NW_006760201.1	+	8242474	8247207	4733	pper01100:Metabolic pathways;pper01110:Biosynthesis of secondary metabolites;pper00053:Ascorbate and aldarate metabolism	-	GO:0003885:D-arabinono-1,4-lactone oxidase activity;GO:0008762:UDP-N-acetylmuramate dehydrogenase activity;GO:0016633:galactonolactone dehydrogenase activity;GO:0050660:flavin adenine dinucleotide binding	GO:0016020:membrane
ppe-miR3627-5p	18771761	PRUPE_ppa010984mg	NCBI_Assembly:GCF_000346465.:	NW_006760201.1	+	15247740	15250259	2519	-	-	GO:0008270:zinc ion binding	-
ppe-miR3627-5p	18771952	PRUPE_ppa014669mg	NCBI_Assembly:GCF_000346465.:	NW_006760208.1	-	24092818	24096138	3320	-	-	GO:0004672:protein kinase activity;GO:0005524:ATP binding	GO:0016021:integral component of membrane
ppe-miR3627-5p	18772086	PRUPE_ppa014249mg	NCBI_Assembly:GCF_000346465.:	NW_006760208.1	+	17533894	17534340	446	-	-	-	-
ppe-miR3627-5p	18772098	PRUPE_ppa025092mg	NCBI_Assembly:GCF_000346465.:	NW_006760208.1	-	23857732	23858429	697	-	-	GO:0016491:oxidoreductase activity	-
ppe-miR3627-5p	18772171	PRUPE_ppa008068mg	NCBI_Assembly:GCF_000346465.:	NW_006760208.1	-	27629821	27631484	1663	-	-	-	-
ppe-miR3627-5p	18772263	PRUPE_ppa006242mg	NCBI_Assembly:GCF_000346465.:	NW_006760208.1	-	25352968	25357469	4501	-	GO:0055085:transmembrane transport	-	GO:0016021:integral component of membrane

ppe-miR3627-5p	18772294	PRUPE_ppa006875mg	NCBI_Assembly:GCF_000346465.:	NW_006760208.1	-	4106051	4110236	4185	pper01100:Metabolic pathways;pper00061:Fatty acid biosynthesis;pper01212:Fatty acid metabolism;pper00780:Biotin metabolism	-	-	-
ppe-miR3627-5p	18772369	PRUPE_ppa006043mg	NCBI_Assembly:GCF_000346465.:	NW_006760208.1	-	24175252	24178089	2837	-	-	GO:0015369: calcium:proton antiporter activity	GO:0016021:integral component of membrane
ppe-miR3627-5p	18772448	PRUPE_ppa008584mg	NCBI_Assembly:GCF_000346465.:	NW_006760208.1	+	24230296	24231786	1490	-	GO:0006457: protein folding	-	-
ppe-miR3627-5p	18772481	PRUPE_ppa023289mg	NCBI_Assembly:GCF_000346465.:	NW_006760208.1	-	16792530	16792994	464	-	-	-	-
ppe-miR3627-5p	18772482	PRUPE_ppa017475mg	NCBI_Assembly:GCF_000346465.:	NW_006760208.1	+	5420063	5422051	1988	pper03040:Spliceosome;pper04141:Protein processing in endoplasmic reticulum;pper04144:Endocytosis	-	GO:0005524: ATP binding	-

ppe-miR3627-5p	18772557	PRUPE_ppa003478mg	NCBI_Assembly:GCF_000346465.	NW_006760208.1	-		27533970	27537889	3919	pper01100:Metabolic pathways;pper01110:Biosynthesis of secondary metabolites;pper00906:Carotenoid biosynthesis	GO:0016117: carotenoid biosynthetic process;GO:0052889:9,9'-di-cis-zeta-carotene desaturation to 7,9,7',9'-tetra-cis-lycopene	GO:0016719: carotene 7,8-desaturase activity;GO:0052886:9,9'-dicis-carotene:quinone oxidoreductase activity;GO:0052887:7,9,9'-tricis-neurosporene:quinone oxidoreductase activity	GO:0009509:chromoplast;GO:0009941:chloroplast envelope
ppe-miR3627-5p	18772661	PRUPE_ppa008649mg	NCBI_Assembly:GCF_000346465.	NW_006760208.1	+		5270484	5272264	1780	-	GO:0006816: calcium ion transport;GO:0006869:lipid transport;GO:0006891:intra-Golgi vesicle-mediated transport;GO:0010351:lithium ion transport;GO:0016558:protein import into peroxisome matrix;GO:0048573:photoperiodism, flowering;GO:0051928:positive regulation of calcium ion transport	GO:0003676: nucleic acid binding;GO:0008270:zinc ion binding	GO:0005737:cytoplasm

ppe-miR3627-5p	18772735	PRUPE_ppa010192mg	NCBI_Assembly:GCF_000346465.:	NW_006760208.1	+	24189970	24192385	2415	-	-	GO:0016788: hydrolase activity, acting on ester bonds	-
ppe-miR3627-5p	18772831	PRUPE_ppa017028mg	NCBI_Assembly:GCF_000346465.:	NW_006760208.1	-	27842932	27843327	395	-	-	-	-
ppe-miR3627-5p	18772885	PRUPE_ppa005494mg	NCBI_Assembly:GCF_000346465.:	NW_006760208.1	-	19193904	19197088	3184	-	-	GO:0004672: protein kinase activity;GO:0005524:ATP binding	-
ppe-miR3627-5p	18772946	PRUPE_ppa018192mg	NCBI_Assembly:GCF_000346465.:	NW_006760208.1	-	19082771	19085830	3059	-	-	-	-
ppe-miR3627-5p	18772961	PRUPE_ppa012987mg	NCBI_Assembly:GCF_000346465.:	NW_006760208.1	-	21549387	21553228	3841	-	GO:0045454: cell redox homeostasis	-	GO:0005623:cell
ppe-miR3627-5p	18773082	PRUPE_ppa025915mg	NCBI_Assembly:GCF_000346465.:	NW_006760208.1	-	23185830	23187480	1650	-	GO:0009790: embryo development; GO:0051726:regulation of cell cycle	-	GO:0005634:nucleus
ppe-miR3627-5p	18773106	PRUPE_ppa012106mg	NCBI_Assembly:GCF_000346465.:	NW_006760208.1	-	296235	297110	875	-	-	GO:0008242: omega peptidase activity	-
ppe-miR3627-5p	18773296	PRUPE_ppb022233mg	NCBI_Assembly:GCF_000346465.:	NW_006760208.1	-	23241796	23243314	1518	-	-	-	-
ppe-miR3627-5p	18773376	PRUPE_ppa005731mg	NCBI_Assembly:GCF_000346465.:	NW_006760208.1	+	23264533	23268000	3467	-	-	-	-
ppe-miR3627-5p	18773422	PRUPE_ppa001081mg	NCBI_Assembly:GCF_000346465.:	NW_006760208.1	+	2369883	2379585	9702	-	GO:0006260: DNA replication;GO:0006281:DNA repair;GO:0006310:DNA recombination	GO:0003676: nucleic acid binding;GO:0005524:ATP binding;GO:0043140:ATP-dependent 3'-5' DNA helicase activity	GO:0005622:intracellular

ppe-miR3627-5p	18773481	PRUPE_ppa000563mg	NCBI_Assembly:GCF_000346465.:	NW_006760208.1	-	24496995	24502150	5155	pper04120:Ubiquitin mediated proteolysis	GO:0006464: cellular protein modification process	GO:0005524: ATP binding;GO:0008641:small protein activating enzyme activity	-
ppe-miR3627-5p	18773514	PRUPE_ppa022027mg	NCBI_Assembly:GCF_000346465.:	NW_006760208.1	+	10261566	10263198	1632	-	GO:0015074: DNA integration	GO:0003676: nucleic acid binding	-
ppe-miR3627-5p	18773676	PRUPE_ppa021160mg	NCBI_Assembly:GCF_000346465.:	NW_006760208.1	-	27366638	27368946	2308	-	-	-	-
ppe-miR3627-5p	18773712	PRUPE_ppa000709mg	NCBI_Assembly:GCF_000346465.:	NW_006760208.1	+	24089261	24092491	3230	-	-	GO:0004674: protein serine/threonine kinase activity;GO:0005524:ATP binding	GO:0016021:integral component of membrane
ppe-miR3627-5p	18773767	PRUPE_ppa007247mg	NCBI_Assembly:GCF_000346465.:	NW_006760208.1	+	1552672	1556263	3591	-	-	-	-
ppe-miR3627-5p	18773785	PRUPE_ppa023741mg	NCBI_Assembly:GCF_000346465.:	NW_006760208.1	+	24518932	24519300	368	-	-	-	-
ppe-miR3627-5p	18773796	PRUPE_ppa013671mg	NCBI_Assembly:GCF_000346465.:	NW_006760208.1	-	23861036	23861746	710	-	-	GO:0016491: oxidoreductase activity	-
ppe-miR3627-5p	18773846	PRUPE_ppa007621mg	NCBI_Assembly:GCF_000346465.:	NW_006760208.1	+	28552209	28553650	1441	-	GO:0009555: pollen development; GO:0010252: auxin homeostasis; GO:0080162:intracellular auxin transport	GO:0080161: auxin transmembrane transporter activity	GO:0005783:endoplasmic reticulum;GO:0016021:integral component of membrane
ppe-miR3627-5p	18773902	PRUPE_ppa023557mg	NCBI_Assembly:GCF_000346465.:	NW_006760208.1	+	26340042	26340665	623	-	-	-	-
ppe-miR3627-5p	18773928	PRUPE_ppa007006mg	NCBI_Assembly:GCF_000346465.:	NW_006760208.1	+	22321963	22324058	2095	pper04712:Circadian rhythm - plant	-	-	-
ppe-miR3627-5p	18773963	PRUPE_ppa017613mg	NCBI_Assembly:GCF_000346465.:	NW_006760208.1	-	15711009	15711362	353	-	-	-	-
ppe-miR3627-5p	18774050	PRUPE_ppa014163mg	NCBI_Assembly:GCF_000346465.:	NW_006760208.1	+	3279785	3280573	788	-	-	-	-

ppe-miR3627-5p	18774230	PRUPE_ppa005889mg	NCBI_Assembly:GCF_000346465.:	NW_006760208.1	+	25428887	25433106	4219	-	GO:0006486:protein glycosylation;GO:0006810:transport	-	GO:0016021:integral component of membrane
ppe-miR3627-5p	18774278	PRUPE_ppa000249mg	NCBI_Assembly:GCF_000346465.:	NW_006760208.1	+	25754018	25761408	7390	-	-	GO:0005524:ATP binding;GO:0016887:ATPase activity	GO:0016020:membrane
ppe-miR3627-5p	18774429	PRUPE_ppb016053mg	NCBI_Assembly:GCF_000346465.:	NW_006760208.1	-	15738723	15739151	428	-	-	-	-
ppe-miR3627-5p	18774495	PRUPE_ppa008066mg	NCBI_Assembly:GCF_000346465.:	NW_006760208.1	+	5486574	5491478	4904	-	-	GO:0003824:catalytic activity;GO:0050662:coenzyme binding	-
ppe-miR3627-5p	18774542	PRUPE_ppa023119mg	NCBI_Assembly:GCF_000346465.:	NW_006760208.1	-	10182479	10183818	1339	-	-	-	-
ppe-miR3627-5p	18774564	PRUPE_ppa014550mg	NCBI_Assembly:GCF_000346465.:	NW_006760208.1	+	22790674	22791904	1230	-	-	-	-
ppe-miR3627-5p	18774569	PRUPE_ppa008090mg	NCBI_Assembly:GCF_000346465.:	NW_006760208.1	-	11143968	11149067	5099	pper01100:Metabolic pathways;pper00460:Cyanoamino acid metabolism	GO:0006807:nitrogen compound metabolic process	GO:0016810:hydrolase activity, acting on carbon-nitrogen (but not peptide) bonds	-
ppe-miR3627-5p	18774727	PRUPE_ppa000518mg	NCBI_Assembly:GCF_000346465.:	NW_006760208.1	+	28834266	28841989	7723	-	GO:0046856:phosphatidylinositol dephosphorylation	-	-
ppe-miR3627-5p	18774767	PRUPE_ppa023379mg	NCBI_Assembly:GCF_000346465.:	NW_006760208.1	-	6899754	6900584	830	-	-	-	-
ppe-miR3627-5p	18774788	PRUPE_ppa019213mg	NCBI_Assembly:GCF_000346465.:	NW_006760208.1	-	13244909	13246009	1100	-	-	-	-
ppe-miR3627-5p	18774804	PRUPE_ppa022897mg	NCBI_Assembly:GCF_000346465.:	NW_006760208.1	+	7489425	7489928	503	-	-	-	-
ppe-miR3627-5p	18774842	PRUPE_ppb013373mg	NCBI_Assembly:GCF_000346465.:	NW_006760208.1	-	5697869	5698623	754	-	-	-	-
ppe-miR3627-5p	18775000	PRUPE_ppa002406mg	NCBI_Assembly:GCF_000346465.:	NW_006760208.1	-	26597166	26603335	6169	-	-	GO:0000062:fatty-acyl-CoA binding	-
ppe-miR3627-5p	18775038	PRUPE_ppa023709mg	NCBI_Assembly:GCF_000346465.:	NW_006760208.1	+	6653434	6654696	1262	-	-	-	-

ppe-miR3627-5p	18775122	PRUPE_ppa009132mg	NCBI_Assembly:GCF_000346465.:	NW_006760208.1	+	1165700	1171648	5948	-	GO:0006816: calcium ion transport;GO:0006833:water transport;GO:0007030:Golgi organization; GO:0007033: vacuole organization; GO:0009651:response to salt stress;GO:0009750:response to fructose;GO:0048767:root hair elongation	-	GO:0005737:cytoplasm
ppe-miR3627-5p	18775126	PRUPE_ppa009621mg	NCBI_Assembly:GCF_000346465.:	NW_006760208.1	-	20800639	20804889	4250	pper01100:Metabolic pathways;pper00230:Purine metabolism;pper00920:Sulfur metabolism	GO:0000103: sulfate assimilation;GO:0070814:hydrogen sulfide biosynthetic process	GO:0004020: adenylylsulfate kinase activity;GO:0005524:ATP binding	-
ppe-miR3627-5p	18775129	PRUPE_ppa026862mg	NCBI_Assembly:GCF_000346465.:	NW_006760208.1	-	23630546	23632924	2378	-	-	-	-
ppe-miR3627-5p	18775209	PRUPE_ppa006991mg	NCBI_Assembly:GCF_000346465.:	NW_006760208.1	-	20044484	20048124	3640	-	-	GO:0008270: zinc ion binding	-
ppe-miR3627-5p	18775213	PRUPE_ppa016881mg	NCBI_Assembly:GCF_000346465.:	NW_006760208.1	-	25955971	25957900	1929	-	GO:0006352: DNA-templated transcription, initiation;GO:0016570:histone modification	GO:0003677: DNA binding	GO:0005634:nucleus

ppe-miR3627-5p	18775260	PRUPE_ppa007684mg	NCBI_Assembly:GCF_000346465.:	NW_006760208.1	+	27960297	27964411	4114	pper01100:Metabolic pathways;pper01110:Biosynthesis of secondary metabolites;pper01230:Biosynthesis of amino acids;pper00260:Glycine, serine and threonine metabolism	GO:0006520: cellular amino acid metabolic process	GO:0016829:lyase activity;GO:0030170:pyridoxal phosphate binding	-
ppe-miR3627-5p	18775356	PRUPE_ppa009102mg	NCBI_Assembly:GCF_000346465.:	NW_006760208.1	+	23754229	23757433	3204	-	GO:0006007: glucose catabolic process;GO:0006839:mitochondrial transport;GO:0009744:response to sucrose;GO:0009749:response to glucose;GO:0009750:response to fructose;GO:0009853:photorespiration	GO:0017077: oxidative phosphorylation uncoupler activity	GO:0005774:vacuolar membrane;GO:0009506:plasmodesma;GO:0009507:chloroplast;GO:0016021:integral component of membrane;GO:0031966:mitochondrial membrane
ppe-miR3627-5p	18775442	PRUPE_ppa006390mg	NCBI_Assembly:GCF_000346465.:	NW_006760208.1	-	5217109	5220287	3178	-	-	GO:0004674: protein serine/threonine kinase activity;GO:0005524:ATP binding	-
ppe-miR3627-5p	18775483	PRUPE_ppa016524mg	NCBI_Assembly:GCF_000346465.:	NW_006760208.1	+	18799836	18802523	2687	pper04626:Plant-pathogen interaction	-	GO:0043531: ADP binding	-

ppe-miR3627-5p	18775492	PRUPE_ppa023027mg	NCBI_Assembly:GCF_000346465.:	NW_006760208.1	-	27741945	27743622	1677	-	-	GO:0004190:aspartic-type endopeptidase activity	-
ppe-miR3627-5p	18775585	PRUPE_ppa009109mg	NCBI_Assembly:GCF_000346465.:	NW_006760208.1	-	28744479	28749803	5324	pper01100:Metabolic pathways;pper01110:Biosynthesis of secondary metabolites;pper00630:Glyoxylate and dicarboxylate metabolism	-	GO:0016791:phosphatase activity	-
ppe-miR3627-5p	18775600	PRUPE_ppa022443mg	NCBI_Assembly:GCF_000346465.:	NW_006760208.1	-	22554009	22557757	3748	-	-	GO:0004674:protein serine/threonine kinase activity;GO:0005524:ATP binding	GO:0016021:integral component of membrane
ppe-miR3627-5p	18775664	PRUPE_ppa019986mg	NCBI_Assembly:GCF_000346465.:	NW_006760212.1	-	14045979	14048379	2400	-	-	GO:0046872:metal ion binding	-
ppe-miR3627-5p	18775687	PRUPE_ppa016745mg	NCBI_Assembly:GCF_000346465.:	NW_006760212.1	+	6097784	6098926	1142	-	-	GO:0008234:cysteine-type peptidase activity	-
ppe-miR3627-5p	18775746	PRUPE_ppa009816mg	NCBI_Assembly:GCF_000346465.:	NW_006760212.1	-	2021724	2027890	6166	-	GO:0000278:mitotic cell cycle;GO:0006310:DNA recombination;GO:0006396:RNA processing;GO:0010413:glucuronoxylan metabolic process;GO:0045492:xylan biosynthetic process	GO:0003676:nucleic acid binding;GO:0005524:ATP binding;GO:0008026:ATP-dependent helicase activity;GO:0009378:four-way junction helicase activity;GO:0043138:3'-5' DNA helicase activity	GO:0016592:mediator complex

ppe-miR3627-5p	18775751	PRUPE_ppa017546mg	NCBI_Assembly:GCF_000346465.:	NW_006760212.1	-	6524421	6526042	1621	-	-	GO:0016706: oxidoreductase activity, acting on paired donors, with incorporation or reduction of molecular oxygen, 2-oxoglutarate as one donor, and incorporation of one atom each of oxygen into both donors;GO:0046872:metal ion binding	-
ppe-miR3627-5p	18775805	PRUPE_ppa015669mg	NCBI_Assembly:GCF_000346465.:	NW_006760212.1	+	11359439	11360035	596	-	-	-	-
ppe-miR3627-5p	18775828	PRUPE_ppa023749mg	NCBI_Assembly:GCF_000346465.:	NW_006760212.1	+	11398268	11398864	596	-	-	-	-
ppe-miR3627-5p	18775856	PRUPE_ppa018383mg	NCBI_Assembly:GCF_000346465.:	NW_006760212.1	+	6023632	6026118	2486	-	-	GO:0008234: cysteine-type peptidase activity	-
ppe-miR3627-5p	18775874	PRUPE_ppa021485mg	NCBI_Assembly:GCF_000346465.:	NW_006760212.1	+	11380363	11380887	524	-	-	-	-
ppe-miR3627-5p	18775970	PRUPE_ppa003802mg	NCBI_Assembly:GCF_000346465.:	NW_006760212.1	-	5290622	5293312	2690	pper03008:Ribosome biogenesis in eukaryotes	GO:0001522: pseudouridine synthesis;GO:0006396:RNA processing	GO:0003723: RNA binding;GO:0009982:pseudouridine synthase activity	-
ppe-miR3627-5p	18776004	PRUPE_ppa001235mg	NCBI_Assembly:GCF_000346465.:	NW_006760212.1	-	10994361	11002339	7978	pper01100:Metabolic pathways;pper00480:Glutathione metabolism	-	GO:0008237: metallopeptidase activity;GO:0008270:zinc ion binding	-

ppe-miR3627-5p	18776013	PRUPE_ppa018792mg	NCBI_Assembly:GCF_000346465.:	NW_006760212.1	+	13454809	13456284	1475	-	GO:0008643: carbohydrate transport	-	GO:0016021:integral component of membrane
ppe-miR3627-5p	18776195	PRUPE_ppa013522mg	NCBI_Assembly:GCF_000346465.:	NW_006760212.1	+	13469822	13471882	2060	-	GO:0010286: heat acclimation	-	-
ppe-miR3627-5p	18776455	PRUPE_ppa013326mg	NCBI_Assembly:GCF_000346465.:	NW_006760212.1	-	18051182	18052249	1067	-	-	-	-
ppe-miR3627-5p	18776456	PRUPE_ppa002398mg	NCBI_Assembly:GCF_000346465.:	NW_006760212.1	+	16973490	16976190	2700	-	-	GO:0003677: DNA binding	-
ppe-miR3627-5p	18776478	PRUPE_ppa012437mg	NCBI_Assembly:GCF_000346465.:	NW_006760212.1	+	3469223	3470205	982	-	-	-	-
ppe-miR3627-5p	18776496	PRUPE_ppa006389mg	NCBI_Assembly:GCF_000346465.:	NW_006760212.1	-	15687887	15689324	1437	-	GO:0008283: cell proliferation;GO:0009735:response to cytokinin;GO:0009737:response to abscisic acid;GO:0009739:response to gibberellin;GO:0010229:inflorescence development; GO:0031347:regulation of defense response	GO:0003700: sequence-specific DNA binding transcription factor activity	-

ppe-miR3627-5p	18776551	PRUPE_ppa016142mg	NCBI_Assembly:GCF_000346465.:	NW_006760212.1	+	17909095	17910180	1085	-	GO:0001708: cell fate specification; GO:0010589:leaf proximal/distal pattern formation;GO:0045893:positive regulation of transcription, DNA-templated	GO:0003677: DNA binding	GO:0005634:nucleus;GO:0005737:cytoplasm
ppe-miR3627-5p	18776620	PRUPE_ppa014950mg	NCBI_Assembly:GCF_000346465.:	NW_006760212.1	+	17225096	17231264	6168	-	GO:0007018: microtubule-based movement	GO:0003777: microtubule motor activity;GO:0005524:ATP binding	GO:0005871:kinesin complex;GO:0005874:microtubule
ppe-miR3627-5p	18776725	PRUPE_ppa023983mg	NCBI_Assembly:GCF_000346465.:	NW_006760212.1	+	11151284	11153563	2279	-	-	-	-
ppe-miR3627-5p	18776753	PRUPE_ppa005138mg	NCBI_Assembly:GCF_000346465.:	NW_006760212.1	+	12988419	12990143	1724	-	-	GO:0004674: protein serine/threonine kinase activity;GO:0005524:ATP binding	-
ppe-miR3627-5p	18776756	PRUPE_ppa015467mg	NCBI_Assembly:GCF_000346465.:	NW_006760212.1	-	18124919	18125672	753	-	-	GO:0008270: zinc ion binding	-
ppe-miR3627-5p	18776824	PRUPE_ppa017658mg	NCBI_Assembly:GCF_000346465.:	NW_006760212.1	-	14932530	14933539	1009	pper03050:Proteasome;pper03440:Homologous recombination	-	-	-

ppe-miR3627-5p	18776899	PRUPE_ppa006999mg	NCBI_Assembly:GCF_000346465.:	NW_006760212.1	-	13958011	13960527	2516	pper03050:Proteasome	GO:0006635:fatty acid beta-oxidation;GO:0006888:ER to Golgi vesicle-mediated transport;GO:0009407:toxin catabolic process;GO:0009640:photomorphogenesis;GO:0009735:response to cytokinin;GO:0010090:trichome morphogenesis;GO:0010388:cullin deneddylation;GO:0042023:DNA endoreduplication;GO:0043090:amino acid import;GO:0043161:proteasome-mediated ubiquitin-dependent protein catabolic process;GO:0048767:root hair elongation;GO	-	GO:0005634:nucleus;GO:0005829:cytosol;GO:0005886:plasma membrane
ppe-miR3627-5p	18776902	PRUPE_ppa018275mg	NCBI_Assembly:GCF_000346465.:	NW_006760212.1	+	10443741	10445781	2040	-	-	-	-
ppe-miR3627-5p	18776912	PRUPE_ppa003460mg	NCBI_Assembly:GCF_000346465.:	NW_006760212.1	+	9078875	9082464	3589	-	-	-	-
ppe-miR3627-5p	18776931	PRUPE_ppa018587mg	NCBI_Assembly:GCF_000346465.:	NW_006760212.1	-	4660074	4660613	539	-	-	-	GO:0005615:extracellular space

ppe-miR3627-5p	18777012	PRUPE_ppa012414mg	NCBI_Assembly:GCF_000346465.:	NW_006760212.1	+	13581887	13583455	1568	-	-	GO:0003700: sequence-specific DNA binding transcription factor activity;GO:0043565:sequence-specific DNA binding	-
ppe-miR3627-5p	18777069	PRUPE_ppa026103mg	NCBI_Assembly:GCF_000346465.:	NW_006760212.1	+	14115267	14116544	1277	pper01100:Metabolic pathways;pper01110:Biosynthesis of secondary metabolites;pper00460:Cyanoamino acid metabolism;pper00500:Starch and sucrose metabolism;pper00940:Phenylpropanoid biosynthesis	GO:0005975: carbohydrate metabolic process	GO:0004553: hydrolase activity, hydrolyzing O-glycosyl compounds	-
ppe-miR3627-5p	18777227	PRUPE_ppa016857mg	NCBI_Assembly:GCF_000346465.:	NW_006760212.1	+	10678680	10680419	1739	-	-	-	-
ppe-miR3627-5p	18777339	PRUPE_ppa020915mg	NCBI_Assembly:GCF_000346465.:	NW_006760212.1	-	11448711	11449304	593	-	-	-	-
ppe-miR3627-5p	18777429	PRUPE_ppa021960mg	NCBI_Assembly:GCF_000346465.:	NW_006760212.1	+	11417831	11418469	638	-	-	-	-
ppe-miR3627-5p	18777557	PRUPE_ppa009476mg	NCBI_Assembly:GCF_000346465.:	NW_006760212.1	+	17679651	17681863	2212	-	GO:0006629:lipid metabolic process	GO:0016627: oxidoreductase activity, acting on the CH-CH group of donors	GO:0005737:cytoplasm;GO:0016021:integral component of membrane
ppe-miR3627-5p	18777592	PRUPE_ppa027018mg	NCBI_Assembly:GCF_000346465.:	NW_006760212.1	+	11389480	11390076	596	-	-	-	-

ppe-miR3627-5p	18777599	PRUPE_ppa002741mg	NCBI_Assembly:GCF_000346465.:	NW_006760212.1	+	14419772	14423660	3888	pper03040:Spliceosome	-	GO:0003676: nucleic acid binding;GO:0004386:helicase activity;GO:0005524:ATP binding	-
ppe-miR3627-5p	18777722	PRUPE_ppa015080mg	NCBI_Assembly:GCF_000346465.:	NW_006760212.1	+	13765518	13770790	5272	-	GO:0007030: Golgi organization; GO:0015031: protein transport	-	GO:0005773:vacuole;GO:0016020:membrane
ppe-miR3627-5p	18777889	PRUPE_ppa026530mg	NCBI_Assembly:GCF_000346465.:	NW_006760212.1	+	8660990	8663254	2264	-	-	-	-
ppe-miR3627-5p	18777896	PRUPE_ppa001981mg	NCBI_Assembly:GCF_000346465.:	NW_006760212.1	-	12352940	12358660	5720	pper01100:Metabolic pathways;pper00760:Nicotinate and nicotinamide metabolism	GO:0009435: NAD biosynthetic process;GO:0046686:response to cadmium ion	GO:0003952: NAD+ synthase (glutamine-hydrolyzing) activity;GO:0005524:ATP binding;GO:0016810:hydrolase activity, acting on carbon-nitrogen (but not peptide) bonds	GO:0005829:cytosol
ppe-miR3627-5p	18777906	PRUPE_ppb019466mg	NCBI_Assembly:GCF_000346465.:	NW_006760212.1	-	15668515	15670171	1656	-	-	-	-
ppe-miR3627-5p	18777937	PRUPE_ppa027140mg	NCBI_Assembly:GCF_000346465.:	NW_006760212.1	+	13546531	13548150	1619	-	-	GO:0016491: oxidoreductase activity	-
ppe-miR3627-5p	18777989	PRUPE_ppa025782m2g	NCBI_Assembly:GCF_000346465.:	NW_006760212.1	+	13227835	13230487	2652	pper04144:Endocytosis	-	-	GO:0005737:cytoplasm
ppe-miR3627-5p	18777994	PRUPE_ppa022884mg	NCBI_Assembly:GCF_000346465.:	NW_006760212.1	-	10779889	10783412	3523	pper01100:Metabolic pathways;pper00480:Glutathione metabolism	GO:0006750: glutathione biosynthetic process	GO:0004357: glutamate-cysteine ligase activity	-

ppe-miR3627-5p	18778048	PRUPE_ppa019095mg	NCBI_Assembly:GCF_000346465.:	NW_006760212.1	-	13478027	13479893	1866	-	GO:0001709: cell fate determination; GO:0048653: anther development	-	-
ppe-miR3627-5p	18778080	PRUPE_ppa015265mg	NCBI_Assembly:GCF_000346465.:	NW_006760212.1	-	15422631	15423905	1274	-	-	-	-
ppe-miR3627-5p	18778135	PRUPE_ppa021893mg	NCBI_Assembly:GCF_000346465.:	NW_006760219.1	+	280	837	557	-	-	-	-
ppe-miR3627-5p	18778158	PRUPE_ppa000063mg	NCBI_Assembly:GCF_000346465.:	NW_006760220.1	+	7488534	7500972	12438	-	GO:0000278: mitotic cell cycle;GO:0003002:regionalization;GO:0006338:chromatin remodeling;GO:0007155:cell adhesion;GO:0009793:embryo development ending in seed dormancy;GO:0009910:negative regulation of flower development; GO:0010090:trichome morphogenesis;GO:0010228:vegetative to reproductive phase transition of meristem;GO:0042742:defense response to bacterium;GO:0045010:actin nucleation;GO	GO:0003677: DNA binding;GO:0005524:ATP binding	GO:0005618:cell wall;GO:0016514:SWI/SNF complex

ppe-miR3627-5p	18778162	PRUPE_ppa003866mg	NCBI_Assembly:GCF_000346465.:	NW_006760220.1	+	4256156	4260678	4522	pper03013:RNA transport	-	-	-
ppe-miR3627-5p	18778202	PRUPE_ppa000762mg	NCBI_Assembly:GCF_000346465.:	NW_006760220.1	+	4548077	4551789	3712	-	-	GO:0004672: protein kinase activity;GO:0005524:ATP binding	GO:0016021:integral component of membrane
ppe-miR3627-5p	18778294	PRUPE_ppa024082mg	NCBI_Assembly:GCF_000346465.:	NW_006760220.1	+	15734805	15735291	486	-	-	-	-
ppe-miR3627-5p	18778353	PRUPE_ppa022422mg	NCBI_Assembly:GCF_000346465.:	NW_006760220.1	-	4867455	4868573	1118	-	-	GO:0008762: UDP-N-acetylmuramate dehydrogenase activity;GO:0050660:flavin adenine dinucleotide binding	-
ppe-miR3627-5p	18778438	PRUPE_ppa023450mg	NCBI_Assembly:GCF_000346465.:	NW_006760220.1	+	9884939	9886570	1631	-	-	-	-
ppe-miR3627-5p	18778476	PRUPE_ppa018799mg	NCBI_Assembly:GCF_000346465.:	NW_006760220.1	+	14759312	14760403	1091	-	GO:0006351:transcription, DNA-templated;GO:0006355:regulation of transcription, DNA-templated	GO:0003677: DNA binding	GO:0005634:nucleus
ppe-miR3627-5p	18778552	PRUPE_ppa027108mg	NCBI_Assembly:GCF_000346465.:	NW_006760220.1	-	3839122	3840591	1469	-	GO:0055114: oxidation-reduction process	-	GO:0016021:integral component of membrane
ppe-miR3627-5p	18778614	PRUPE_ppa020065mg	NCBI_Assembly:GCF_000346465.:	NW_006760220.1	+	5802166	5805539	3373	pper04626:Plant-pathogen interaction	-	GO:0043531: ADP binding	-

ppe-miR3627-5p	18778653	PRUPE_ppa016878mg	NCBI_Assembly:GCF_000346465.:	NW_006760220.1	+	14142445	14148380	5935	pper03022:Basal transcription factors	GO:0006352:DNA-templated transcription, initiation;GO:0051090:regulation of sequence-specific DNA binding transcription factor activity	-	GO:0005634:nucleus
ppe-miR3627-5p	18778759	PRUPE_ppa006647mg	NCBI_Assembly:GCF_000346465.:	NW_006760220.1	-	13884905	13887127	2222	pper01100:Metabolic pathways;pper00240:Pyrimidine metabolism;pper00250:Alanine, aspartate and glutamate metabolism	GO:0006164:purine nucleotide biosynthetic process;GO:0006207:'de novo' pyrimidine nucleobase biosynthetic process;GO:0006520:cellular amino acid metabolic process;GO:0009220:pyrimidine ribonucleotide biosynthetic process	GO:0004070:aspartate carbamoyltransferase activity;GO:0016597:amino acid binding	GO:0005829:cytosol;GO:0009570:chloroplast stroma
ppe-miR3627-5p	18778768	PRUPE_ppb010594mg	NCBI_Assembly:GCF_000346465.:	NW_006760220.1	+	29593008	29594799	1791	pper01100:Metabolic pathways;pper01110:Biosynthesis of secondary metabolites;pper00940:Phenylpropanoid biosynthesis	-	GO:0008270:zinc ion binding;GO:0016491:oxidoreductase activity	-

ppe-miR3627-5p	18778833	PRUPE_ppa010564mg	NCBI_Assembly:GCF_000346465.:	NW_006760220.1	+	514035	515906	1871	-	-	GO:0005094: Rho GDP-dissociation inhibitor activity	GO:0005737:cytoplasm
ppe-miR3627-5p	18778962	PRUPE_ppa009361mg	NCBI_Assembly:GCF_000346465.:	NW_006760220.1	-	5230934	5234898	3964	-	GO:0006886:intracellular protein transport	-	GO:0005622:intracellular;GO:0005886:plasma membrane
ppe-miR3627-5p	18779050	PRUPE_ppa018063mg	NCBI_Assembly:GCF_000346465.:	NW_006760220.1	-	8433749	8434486	737	pper00480:Glutathione metabolism	-	-	-
ppe-miR3627-5p	18779056	PRUPE_ppa021071mg	NCBI_Assembly:GCF_000346465.:	NW_006760220.1	-	1472076	1476124	4048	-	GO:0048544:recognition of pollen	GO:0004674: protein serine/threonine kinase activity;GO:0005524:ATP binding	-
ppe-miR3627-5p	18779065	PRUPE_ppa012541mg	NCBI_Assembly:GCF_000346465.:	NW_006760220.1	-	1010659	1011518	859	-	-	-	-

ppe-miR3627-5p	18779066	PRUPE_ppa009952mg	NCBI_Assembly:GCF_000346465.:	NW_006760220.1	-	443762	446650	2888	pper01100:Metabolic pathways;pper01110:Biosynthesis of secondary metabolites;pper00562:Inositol phosphate metabolism;pper00053:Ascorbate and aldarate metabolism;pper04070:Phosphatidylinositol signaling system	GO:0009409:response to cold;GO:0010264:myo-inositol hexakisphosphate biosynthetic process;GO:0019243:methylglyoxal catabolic process to D-lactate;GO:0019761:glucosinolate biosynthetic process;GO:0019853:L-ascorbic acid biosynthetic process;GO:0046854:phosphatidylinositol phosphorylation	GO:0010347:L-galactose-1-phosphate phosphatase activity	GO:0005829:cytosol;GO:0005886:plasma membrane
ppe-miR3627-5p	18779121	PRUPE_ppa026035mg	NCBI_Assembly:GCF_000346465.:	NW_006760220.1	-	18284228	18286865	2637	-	-	-	-
ppe-miR3627-5p	18779148	PRUPE_ppa011749mg	NCBI_Assembly:GCF_000346465.:	NW_006760220.1	+	10609391	10610186	795	-	-	GO:0003676:nucleic acid binding;GO:0008408:3'-5' exonuclease activity	-
ppe-miR3627-5p	18779182	PRUPE_ppa001384mg	NCBI_Assembly:GCF_000346465.:	NW_006760220.1	+	461879	464711	2832	-	-	GO:0005524:ATP binding;GO:0043531:ADP binding	-
ppe-miR3627-5p	18779297	PRUPE_ppa022346mg	NCBI_Assembly:GCF_000346465.:	NW_006760220.1	+	20203741	20206199	2458	-	-	-	-
ppe-miR3627-5p	18779301	PRUPE_ppa021968mg	NCBI_Assembly:GCF_000346465.:	NW_006760220.1	+	13713781	13714272	491	-	-	-	-

ppe-miR3627-5p	18779533	PRUPE_ppa014502mg	NCBI_Assembly:GCF_000346465.:	NW_006760220.1	+	30192609	30193116	507	-	GO:0005975: carbohydrate metabolic process	GO:0004650: polygalacturonase activity	-
ppe-miR3627-5p	18779737	PRUPE_ppa003409mg	NCBI_Assembly:GCF_000346465.:	NW_006760220.1	+	17917804	17924929	7125	-	-	GO:0003677: DNA binding	-
ppe-miR3627-5p	18779746	PRUPE_ppa006512mg	NCBI_Assembly:GCF_000346465.:	NW_006760220.1	-	855911	858863	2952	pper01100:Metabolic pathways;pper00260:Glycine, serine and threonine metabolism;pper01200:Carbon metabolism;pper00670:One carbon pool by folate	GO:0006546: glycine catabolic process;GO:0007389:pattern specification process;GO:0008361:regulation of cell size;GO:0009658:chloroplast organization; GO:0009926: auxin polar transport;GO:0010015:root morphogenesis;GO:0010075:regulation of meristem growth;GO:0016117:carotenoid biosynthetic process;GO:0019344:cysteine biosynthetic process;GO:0040007:growth;GO:0042744:hydrogen peroxide catabolic process;GO:0048481:ovule development	GO:0004047: aminomethyltransferase activity;GO:0008483:transaminase activity	GO:0009534:chloroplast thylakoid;GO:0009570:chloroplast stroma;GO:0009941:chloroplast envelope;GO:0016020:membrane;GO:0022626:cytosolic ribosome;GO:0048046:apoplast
ppe-miR3627-5p	18779837	PRUPE_ppa023160mg	NCBI_Assembly:GCF_000346465.:	NW_006760220.1	-	12706120	12707509	1389	-	-	-	-
ppe-miR3627-5p	18779917	PRUPE_ppb017674mg	NCBI_Assembly:GCF_000346465.:	NW_006760220.1	+	11941734	11942033	299	-	-	-	-

ppe-miR3627-5p	18780020	PRUPE_ppa003262mg	NCBI_Assembly:GCF_000346465.:	NW_006760220.1	+	5601440	5605940	4500	-	-	-	-
ppe-miR3627-5p	18780092	PRUPE_ppa007122mg	NCBI_Assembly:GCF_000346465.:	NW_006760220.1	-	18378421	18379972	1551	-	-	-	-
ppe-miR3627-5p	18780532	PRUPE_ppa015462mg	NCBI_Assembly:GCF_000346465.:	NW_006760220.1	-	2611850	2613380	1530	-	-	-	-
ppe-miR3627-5p	18781402	PRUPE_ppa025787mg	NCBI_Assembly:GCF_000346465.:	NW_006760220.1	+	22631190	22632960	1770	pper01100:Metabolic pathways;pper00500:Starch and sucrose metabolism;pper00040:Pentose and glucuronate interconversions	GO:0005975: carbohydrate metabolic process;GO:0071555:cell wall organization	GO:0004650: polygalacturonase activity	GO:0005576:extracellular region
ppe-miR3627-5p	18781443	PRUPE_ppa018240mg	NCBI_Assembly:GCF_000346465.:	NW_006760220.1	-	4120456	4122546	2090	-	-	-	-
ppe-miR3627-5p	18781633	PRUPE_ppa004721mg	NCBI_Assembly:GCF_000346465.:	NW_006760268.1	+	6125913	6127919	2006	-	GO:0006865: amino acid transport	-	GO:0016021:integral component of membrane
ppe-miR3627-5p	18782562	PRUPE_ppa026495mg	NCBI_Assembly:GCF_000346465.:	NW_006760268.1	-	1405747	1409348	3601	-	-	-	-
ppe-miR3627-5p	18782926	PRUPE_ppa011716mg	NCBI_Assembly:GCF_000346465.:	NW_006760268.1	+	6138977	6139869	892	-	-	-	-
ppe-miR3627-5p	18782983	PRUPE_ppa014469mg	NCBI_Assembly:GCF_000346465.:	NW_006760268.1	+	21288904	21289681	777	-	-	-	-
ppe-miR3627-5p	18782985	PRUPE_ppb011343mg	NCBI_Assembly:GCF_000346465.:	NW_006760268.1	+	1301574	1302364	790	-	-	-	-
ppe-miR3627-5p	18783100	PRUPE_ppa023073mg	NCBI_Assembly:GCF_000346465.:	NW_006760268.1	+	8559961	8560469	508	-	-	GO:0004672: protein kinase activity;GO:0005524:ATP binding	-
ppe-miR3627-5p	18783102	PRUPE_ppa001593mg	NCBI_Assembly:GCF_000346465.:	NW_006760268.1	-	17166435	17170295	3860	-	GO:0048544:recognition of pollen	GO:0004672: protein kinase activity;GO:0005524:ATP binding	-
ppe-miR3627-5p	18783130	PRUPE_ppa011883mg	NCBI_Assembly:GCF_000346465.:	NW_006760268.1	-	11640840	11642748	1908	-	-	-	-
ppe-miR3627-5p	18783165	PRUPE_ppb022456mg	NCBI_Assembly:GCF_000346465.:	NW_006760268.1	-	7110374	7110595	221	-	-	-	-
ppe-miR3627-5p	18783220	PRUPE_ppa027198mg	NCBI_Assembly:GCF_000346465.:	NW_006760268.1	+	17934073	17936802	2729	-	-	GO:0003723: RNA binding	-
ppe-miR3627-5p	18783268	PRUPE_ppb020721mg	NCBI_Assembly:GCF_000346465.:	NW_006760268.1	-	18704742	18706234	1492	-	-	-	-
ppe-miR3627-5p	18783301	PRUPE_ppa026724mg	NCBI_Assembly:GCF_000346465.:	NW_006760268.1	+	6190370	6191437	1067	-	-	-	-

ppe-miR3627-5p	18783400	PRUPE_ppa008959mg	NCBI_Assembly:GCF_000346465.:	NW_006760268.1	+	4908825	4911501	2676	-	GO:0006606:protein import into nucleus;GO:0006626:protein targeting to mitochondrion;GO:0019750:chloroplast localization;GO:0045036:protein targeting to chloroplast	GO:0003924:GTPase activity;GO:0005525:GTP binding;GO:0015450:P-P-bond-hydrolysis-driven protein transmembrane transporter activity	GO:0009707:chloroplast outer membrane;GO:0016021:integral component of membrane
ppe-miR3627-5p	18783490	PRUPE_ppa013552mg	NCBI_Assembly:GCF_000346465.:	NW_006760268.1	+	15170248	15171175	927	-	GO:0030001:metal ion transport	GO:0046872:metal ion binding	-
ppe-miR3627-5p	18783526	PRUPE_ppa013164mg	NCBI_Assembly:GCF_000346465.:	NW_006760268.1	-	21108665	21109712	1047	-	-	-	-
ppe-miR3627-5p	18783564	PRUPE_ppa002874mg	NCBI_Assembly:GCF_000346465.:	NW_006760268.1	-	2101506	2103730	2224	-	-	GO:0043531:ADP binding	-
ppe-miR3627-5p	18783612	PRUPE_ppa005899mg	NCBI_Assembly:GCF_000346465.:	NW_006760268.1	+	10809693	10812242	2549	pper03013:RNA transport	-	GO:0003743:translation initiation factor activity	-
ppe-miR3627-5p	18783642	PRUPE_ppa026633mg	NCBI_Assembly:GCF_000346465.:	NW_006760268.1	-	17302783	17303508	725	-	GO:0006950:response to stress	-	-
ppe-miR3627-5p	18783649	PRUPE_ppa003330mg	NCBI_Assembly:GCF_000346465.:	NW_006760268.1	-	19430187	19433082	2895	-	-	-	-
ppe-miR3627-5p	18783654	PRUPE_ppa006437mg	NCBI_Assembly:GCF_000346465.:	NW_006760268.1	+	19246165	19248673	2508	pper00562:Inositol phosphate metabolism;pper04070:Phosphatidylinositol signaling system	GO:0009555:pollen development;GO:0009860:pollen tube growth;GO:0030048:actin filament-based movement	GO:0004725:protein tyrosine phosphatase activity;GO:0008138:protein tyrosine/serine/threonine phosphatase activity	-
ppe-miR3627-5p	18783688	PRUPE_ppa020904mg	NCBI_Assembly:GCF_000346465.:	NW_006760268.1	+	21105725	21107867	2142	-	-	GO:0046872:metal ion binding	-

ppe-miR3627-5p	18783787	PRUPE_ppa008193mg	NCBI_Assembly:GCF_000346465.:	NW_006760268.1	-	1671128	1673802	2674	pper03010:Ribosome	GO:0000028:ribosomal small subunit assembly;GO:0006412:translation	GO:0003735: structural constituent of ribosome	GO:0022627:cytosolic small ribosomal subunit
ppe-miR3627-5p	18783809	PRUPE_ppa019604mg	NCBI_Assembly:GCF_000346465.:	NW_006760268.1	-	15651594	15651935	341	-	-	-	-
ppe-miR3627-5p	18783841	PRUPE_ppa007623mg	NCBI_Assembly:GCF_000346465.:	NW_006760268.1	+	20903152	20905042	1890	pper01100:Metabolic pathways;pper00053:Ascorbate and aldarate metabolism;pper00520:Amino sugar and nucleotide sugar metabolism;pper00040:Pentose and glucuronate interconversions	-	GO:0005524: ATP binding;GO:0016301:kinase activity;GO:0016773:phosphotransferase activity, alcohol group as acceptor	GO:0005737:cytoplasm
ppe-miR3627-5p	18783955	PRUPE_ppa013601mg	NCBI_Assembly:GCF_000346465.:	NW_006760268.1	+	18251172	18253330	2158	pper03013:RNA transport	-	GO:0003743:translation initiation factor activity	-

ppe-miR3627-5p	18783973	PRUPE_ppa009667mg	NCBI_Assembly:GCF_000346465.:	NW_006760268.1	-	19848924	19852023	3099	pper01100:Metabolic pathways;pper00430:Taurine and hypotaurine metabolism	GO:0001666:response to hypoxia;GO:0009862:systemic acquired resistance, salicylic acid mediated signaling pathway;GO:0010089:xylem development; GO:0010310:regulation of hydrogen peroxide metabolic process;GO:0044036:cell wall macromolecule metabolic process	GO:0047800: cysteamine dioxygenase activity	-

ppe-miR3627-5p	18783993	PRUPE_ppa007934mg	NCBI_Assembly:GCF_000346465.:	NW_006760268.1	+	21064285	21066617	2332	pper01100:Metabolic pathways;pper01110:Biosynthesis of secondary metabolites;pper01200:Carbon metabolism;pper00190:Oxidative phosphorylation;pper00020:Citrate cycle (TCA cycle)	GO:0006099:tricarboxylic acid cycle	GO:0008177:succinate dehydrogenase (ubiquinone) activity;GO:0009055:electron carrier activity;GO:0046872:metal ion binding;GO:0051537:2 iron, 2 sulfur cluster binding;GO:0051538:3 iron, 4 sulfur cluster binding;GO:0051539:4 iron, 4 sulfur cluster binding	GO:0005743:mitochondrial inner membrane
ppe-miR3627-5p	18784039	PRUPE_ppa015414mg	NCBI_Assembly:GCF_000346465.:	NW_006760268.1	+	13167236	13168573	1337	-	-	GO:0005215:transporter activity	GO:0016020:membrane
ppe-miR3627-5p	18784054	PRUPE_ppa004003mg	NCBI_Assembly:GCF_000346465.:	NW_006760268.1	+	20907158	20910618	3460	-	GO:0001522:pseudouridine synthesis;GO:0006396:RNA processing;GO:0019288:isopentenyl diphosphate biosynthetic process, methylerythritol 4-phosphate pathway	GO:0003723:RNA binding;GO:0009982:pseudouridine synthase activity	-
ppe-miR3627-5p	18784194	PRUPE_ppa017921mg	NCBI_Assembly:GCF_000346465.:	NW_006760268.1	+	21079579	21081198	1619	-	-	-	-

ppe-miR3627-5p	18784206	PRUPE_ppa016949mg	NCBI_Assembly:GCF_000346465.:	NW_006760268.1	+	18957619	18959010	1391	-	-	GO:0016747:transferase activity, transferring acyl groups other than amino-acyl groups	-
ppe-miR3627-5p	18784253	PRUPE_ppa013486mg	NCBI_Assembly:GCF_000346465.:	NW_006760268.1	-	20533386	20535048	1662	-	GO:0009062:fatty acid catabolic process	-	-
ppe-miR3627-5p	18784330	PRUPE_ppa002530mg	NCBI_Assembly:GCF_000346465.:	NW_006760268.1	+	20635345	20638079	2734	-	-	GO:0004674: protein serine/threonine kinase activity;GO:0005524:ATP binding	-
ppe-miR3627-5p	18784379	PRUPE_ppa005796mg	NCBI_Assembly:GCF_000346465.:	NW_006760268.1	+	21549550	21552878	3328	-	GO:0019408: dolichol biosynthetic process	GO:0016597: amino acid binding	-
ppe-miR3627-5p	18784607	PRUPE_ppa008629mg	NCBI_Assembly:GCF_000346465.:	NW_006760324.1	+	20340439	20341425	986	-	GO:0010413: glucuronoxylan metabolic process;GO:0045492:xylan biosynthetic process	-	-
ppe-miR3627-5p	18784704	PRUPE_ppa027146mg	NCBI_Assembly:GCF_000346465.:	NW_006760324.1	-	2025468	2027319	1851	-	-	GO:0003676: nucleic acid binding	-
ppe-miR3627-5p	18784749	PRUPE_ppa022677mg	NCBI_Assembly:GCF_000346465.:	NW_006760324.1	-	22159337	22160433	1096	-	GO:0006826:iron ion transport;GO:0010106:cellular response to iron ion starvation;GO:0010167:response to nitrate;GO:0015706:nitrate transport	-	-

ppe-miR3627-5p	18784780	PRUPE_ppa002702mg	NCBI_Assembly:GCF_000346465.:	NW_006760324.1	+	24640919	24643035	2116	-	-	GO:0004674: protein serine/threonine kinase activity;GO:0005524:ATP binding	-
ppe-miR3627-5p	18784826	PRUPE_ppb022117mg	NCBI_Assembly:GCF_000346465.:	NW_006760324.1	-	8222803	8223427	624	-	-	-	-
ppe-miR3627-5p	18784857	PRUPE_ppa013105mg	NCBI_Assembly:GCF_000346465.:	NW_006760324.1	-	21897885	21899536	1651	-	GO:0030042: actin filament depolymerization	-	GO:0015629:actin cytoskeleton
ppe-miR3627-5p	18784859	PRUPE_ppa000654mg	NCBI_Assembly:GCF_000346465.:	NW_006760324.1	+	19610286	19614023	3737	-	-	GO:0005524: ATP binding;GO:0016787:hydrolase activity;GO:0046872:metal ion binding	GO:0005783:endoplasmic reticulum;GO:0016021:integral component of membrane
ppe-miR3627-5p	18784875	PRUPE_ppa008506mg	NCBI_Assembly:GCF_000346465.:	NW_006760324.1	+	86348	88893	2545	-	GO:0045454: cell redox homeostasis	-	GO:0005623:cell
ppe-miR3627-5p	18784953	PRUPE_ppa024194mg	NCBI_Assembly:GCF_000346465.:	NW_006760324.1	-	26134332	26134805	473	-	-	-	-
ppe-miR3627-5p	18784990	PRUPE_ppa019777mg	NCBI_Assembly:GCF_000346465.:	NW_006760324.1	+	26375752	26378000	2248	pper04120:Ubiquitin mediated proteolysis	-	-	-
ppe-miR3627-5p	18785010	PRUPE_ppa026782mg	NCBI_Assembly:GCF_000346465.:	NW_006760324.1	-	24963203	24964267	1064	-	-	-	-
ppe-miR3627-5p	18785011	PRUPE_ppa013167mg	NCBI_Assembly:GCF_000346465.:	NW_006760324.1	-	24951504	24952210	706	-	-	-	-
ppe-miR3627-5p	18785040	PRUPE_ppa002291mg	NCBI_Assembly:GCF_000346465.:	NW_006760324.1	+	26274315	26276741	2426	-	-	GO:0004672: protein kinase activity;GO:0005524:ATP binding;GO:0030246:carbohydrate binding	-
ppe-miR3627-5p	18785129	PRUPE_ppa013414mg	NCBI_Assembly:GCF_000346465.:	NW_006760324.1	+	16564743	16566676	1933	pper03010:Ribosome	GO:0006412:translation	GO:0003735: structural constituent of ribosome	GO:0005840:ribosome

ppe-miR3627-5p	18785337	PRUPE_ppa009506mg	NCBI_Assembly:GCF_000346465.:	NW_006760324.1	-	22777485	22779328	1843	-	-	GO:0005215:transporter activity	GO:0016021:integral component of membrane
ppe-miR3627-5p	18785342	PRUPE_ppa013379mg	NCBI_Assembly:GCF_000346465.:	NW_006760324.1	-	21042498	21046231	3733	pper01100:Metabolic pathways;pper01110:Biosynthesis of secondary metabolites;pper00630:Glyoxylate and dicarboxylate metabolism	-	-	-
ppe-miR3627-5p	18785417	PRUPE_ppa003188mg	NCBI_Assembly:GCF_000346465.:	NW_006760324.1	+	20641729	20648812	7083	-	GO:0006623:protein targeting to vacuole;GO:0006904:vesicle docking involved in exocytosis;GO:0007030:Golgi organization;GO:0007033:vacuole organization;GO:0048193:Golgi vesicle transport	-	GO:0005829:cytosol;GO:0009705:plant-type vacuole membrane
ppe-miR3627-5p	18785457	PRUPE_ppa009680mg	NCBI_Assembly:GCF_000346465.:	NW_006760324.1	+	23914211	23915735	1524	-	GO:0006397:mRNA processing;GO:0008380:RNA splicing;GO:0034477:U6 snRNA 3'-end processing	GO:0004518:nuclease activity	GO:0005634:nucleus

ppe-miR3627-5p	18785525	PRUPE_ppa026456mg	NCBI_Assembly:GCF_000346465.:	NW_006760324.1	-		22737085	22739353	2268	pper03060:Protein export	GO:0006465: signal peptide processing	GO:0008233: peptidase activity	GO:0005787:signal peptidase complex;GO:0016021:integral component of membrane
ppe-miR3627-5p	18785699	PRUPE_ppa023150mg	NCBI_Assembly:GCF_000346465.:	NW_006760324.1	+		21835120	21836198	1078	-	-	-	-
ppe-miR3627-5p	18785717	PRUPE_ppa018863mg	NCBI_Assembly:GCF_000346465.:	NW_006760324.1	-		19085007	19087894	2887	-	GO:0000165: MAPK cascade;GO:0006364:rRNA processing;GO:0006546:glycine catabolic process;GO:0006612:protein targeting to membrane;GO:0006636:unsaturated fatty acid biosynthetic process;GO:0006733:oxidoreduction coenzyme metabolic process;GO:0006766:vitamin metabolic process;GO:0009072:aromatic amino acid family metabolic process;GO:0009106:lipoate metabolic process;GO:0009108:coenzyme biosynthetic process;GO:0009117:nucleotide	-	GO:0009535:chloroplast thylakoid membrane

ppe-miR3627-5p	18785731	PRUPE_ppa002374mg	NCBI_Assembly:GCF_000346465.:	NW_006760324.1	+	22258389	22262208	3819	-	-	GO:0003677: DNA binding;GO:0003682:chromatin binding	-
ppe-miR3627-5p	18785733	PRUPE_ppa009786mg	NCBI_Assembly:GCF_000346465.:	NW_006760324.1	+	24093494	24094822	1328	-	GO:0019953: sexual reproduction	-	GO:0005576:extracellular region
ppe-miR3627-5p	18785741	PRUPE_ppa012866mg	NCBI_Assembly:GCF_000346465.:	NW_006760324.1	+	15530021	15533108	3087	pper03013:RNA transport;pper03015:mRNA surveillance pathway;pper03040:Spliceosome	GO:0007067: mitotic nuclear division;GO:0009793:embryo development ending in seed dormancy;GO:0010183:pollen tube guidance	-	GO:0005730:nucleolus;GO:0009507:chloroplast;GO:0016607:nuclear speck;GO:0035145:exon-exon junction complex
ppe-miR3627-5p	18785799	PRUPE_ppa004494mg	NCBI_Assembly:GCF_000346465.:	NW_006760324.1	-	15549822	15553881	4059	-	-	-	-
ppe-miR3627-5p	18785802	PRUPE_ppa021549mg	NCBI_Assembly:GCF_000346465.:	NW_006760324.1	+	23222353	23227676	5323	-	-	GO:0004672: protein kinase activity;GO:0005524:ATP binding	-
ppe-miR3627-5p	18785817	PRUPE_ppa025650mg	NCBI_Assembly:GCF_000346465.:	NW_006760324.1	-	20136843	20140465	3622	-	-	GO:0003676: nucleic acid binding;GO:0008270:zinc ion binding	-
ppe-miR3627-5p	18785865	PRUPE_ppa026116mg	NCBI_Assembly:GCF_000346465.:	NW_006760324.1	-	17980194	17980757	563	-	-	-	-
ppe-miR3627-5p	18785885	PRUPE_ppa023953mg	NCBI_Assembly:GCF_000346465.:	NW_006760324.1	-	26325905	26330508	4603	pper02010:ABC transporters	-	GO:0005524: ATP binding;GO:0042626:ATPase activity, coupled to transmembrane movement of substances	GO:0016021:integral component of membrane
ppe-miR3627-5p	18785946	PRUPE_ppb016461mg	NCBI_Assembly:GCF_000346465.:	NW_006760324.1	+	1837466	1843276	5810	-	-	-	-

ppe-miR3627-5p	18786032	PRUPE_ppa011679mg	NCBI_Assembly:GCF_000346465.:	NW_006760324.1	+	24984144	24987037	2893	pper03015:mRNA surveillance pathway	GO:0006378:mRNA polyadenylation	GO:0003729:mRNA binding;GO:0016787:hydrolase activity	GO:0005849:mRNA cleavage factor complex
ppe-miR3627-5p	18786110	PRUPE_ppa010502mg	NCBI_Assembly:GCF_000346465.:	NW_006760324.1	-	24861461	24862540	1079	-	-	GO:0030246:carbohydrate binding	-
ppe-miR3627-5p	18786112	PRUPE_ppa024761mg	NCBI_Assembly:GCF_000346465.:	NW_006760324.1	-	25188321	25190182	1861	-	GO:0055085:transmembrane transport	-	GO:0016021:integral component of membrane
ppe-miR3627-5p	18786182	PRUPE_ppa007130mg	NCBI_Assembly:GCF_000346465.:	NW_006760324.1	-	26459492	26461283	1791	pper03040:Spliceosome	GO:0000398:mRNA splicing, via spliceosome	-	GO:0005634:nucleus
ppe-miR3627-5p	18786190	PRUPE_ppa012419mg	NCBI_Assembly:GCF_000346465.:	NW_006760324.1	+	25386961	25388719	1758	pper01100:Metabolic pathways;pper00190:Oxidative phosphorylation	GO:0022904:respiratory electron transport chain	GO:0016651:oxidoreductase activity, acting on NAD(P)H	GO:0005743:mitochondrial inner membrane
ppe-miR3627-5p	18786209	PRUPE_ppa021240mg	NCBI_Assembly:GCF_000346465.:	NW_006760324.1	+	17099060	17103257	4197	-	-	GO:0004252:serine-type endopeptidase activity	GO:0009505:plant-type cell wall
ppe-miR3627-5p	18786496	PRUPE_ppa015951mg	NCBI_Assembly:GCF_000346465.:	NW_006760324.1	-	12530226	12531191	965	-	-	-	-
ppe-miR3627-5p	18786549	PRUPE_ppa017777mg	NCBI_Assembly:GCF_000346465.:	NW_006760324.1	-	20394484	20397390	2906	-	-	GO:0004222:metalloendopeptidase activity;GO:0008270:zinc ion binding	GO:0031012:extracellular matrix
ppe-miR3627-5p	18786553	PRUPE_ppb022527mg	NCBI_Assembly:GCF_000346465.:	NW_006760324.1	+	12039497	12039865	368	-	-	-	-
ppe-miR3627-5p	18786775	PRUPE_ppb019389mg	NCBI_Assembly:GCF_000346465.:	NW_006760324.1	+	4746762	4752203	5441	-	-	GO:0003676:nucleic acid binding;GO:0008270:zinc ion binding	-
ppe-miR3627-5p	18786816	PRUPE_ppa005866mg	NCBI_Assembly:GCF_000346465.:	NW_006760324.1	+	19035113	19038117	3004	-	-	-	-

ppe-miR3627-5p	18786834	PRUPE_ppa012623mg	NCBI_Assembly:GCF_000346465.:	NW_006760324.1	+	25517927	25519712	1785	-	GO:0009073: aromatic amino acid family biosynthetic process;GO:0010207:photosystem II assembly;GO:0016226:iron-sulfur cluster assembly;GO:0045893:positive regulation of transcription, DNA-templated	-	-
ppe-miR3627-5p	18786902	PRUPE_ppa013547mg	NCBI_Assembly:GCF_000346465.:	NW_006760324.1	+	17147208	17149783	2575	pper04120:Ubiquitin mediated proteolysis;pper03420:Nucleotide excision repair;pper04141:Protein processing in endoplasmic reticulum	-	GO:0008270: zinc ion binding	-
ppe-miR3627-5p	18786982	PRUPE_ppa014330mg	NCBI_Assembly:GCF_000346465.:	NW_006760324.1	-	9844956	9845820	864	-	-	-	-
ppe-miR3627-5p	18787021	PRUPE_ppb025125mg	NCBI_Assembly:GCF_000346465.:	NW_006760324.1	-	6605323	6606720	1397	-	-	-	-
ppe-miR3627-5p	18787030	PRUPE_ppa017732mg	NCBI_Assembly:GCF_000346465.:	NW_006760324.1	-	25269343	25272170	2827	-	-	GO:0016491: oxidoreductase activity	GO:0016021:integral component of membrane
ppe-miR3627-5p	18787125	PRUPE_ppa018991mg	NCBI_Assembly:GCF_000346465.:	NW_006760324.1	-	26246015	26246802	787	-	GO:0030001: metal ion transport	GO:0046872: metal ion binding	-
ppe-miR3627-5p	18787134	PRUPE_ppa013899mg	NCBI_Assembly:GCF_000346465.:	NW_006760324.1	-	25268312	25268969	657	-	-	-	-
ppe-miR3627-5p	18787425	PRUPE_ppa012839mg	NCBI_Assembly:GCF_000346465.:	NW_006760324.1	+	14687720	14689537	1817	-	-	-	-

ppe-miR3627-5p	18787513	PRUPE_ppa020128mg	NCBI_Assembly:GCF_000346465.:	NW_006760324.1	+	26559153	26561327	2174	pper04145:Phagosome;pper04130:SNARE interactions in vesicular transport	GO:0006623:protein targeting to vacuole;GO:0006896:Golgi to vacuole transport;GO:0009556:microsporogenesis;GO:0010200:response to chitin;GO:0045324:late endosome to vacuole transport;GO:0052543:callose deposition in cell wall;GO:0061025:membrane fusion	-	GO:0005770:late endosome;GO:0016020:membrane;GO:0030140:trans-Golgi network transport vesicle
ppe-miR3627-5p	18787545	PRUPE_ppa011012mg	NCBI_Assembly:GCF_000346465.:	NW_006760324.1	-	25546597	25548376	1779	-	-	-	-
ppe-miR3627-5p	18787629	PRUPE_ppa000649mg	NCBI_Assembly:GCF_000346465.:	NW_006760324.1	-	9026764	9037776	11012	-	-	-	-
ppe-miR3627-5p	18787817	PRUPE_ppa021645mg	NCBI_Assembly:GCF_000346465.:	NW_006760359.1	-	245449	246873	1424	-	-	GO:0016758:transferase activity, transferring hexosyl groups	-
ppe-miR3627-5p	18787832	PRUPE_ppa015315mg	NCBI_Assembly:GCF_000346465.:	NW_006760359.1	-	91167	92521	1354	-	-	-	-
ppe-miR3627-5p	18787870	PRUPE_ppa016809mg	NCBI_Assembly:GCF_000346465.:	NW_006760367.1	+	325035	326533	1498	-	-	GO:0003677:DNA binding	-
ppe-miR3627-5p	18787927	PRUPE_ppa001725mg	NCBI_Assembly:GCF_000346465.:	NW_006760376.1	-	639845	645217	5372	-	-	-	-
ppe-miR3627-5p	18787942	PRUPE_ppb024212mg	NCBI_Assembly:GCF_000346465.:	NW_006760376.1	+	476011	478599	2588	-	-	-	-
ppe-miR3627-5p	18787986	PRUPE_ppa007184mg	NCBI_Assembly:GCF_000346465.:	NW_006760380.1	+	304542	306061	1519	-	-	-	GO:0005886:plasma membrane
ppe-miR3627-5p	18788015	PRUPE_ppa003192mg	NCBI_Assembly:GCF_000346465.:	NW_006760384.1	+	516653	524945	8292	-	GO:0000398:mRNA splicing, via spliceosome	GO:0000166:nucleotide binding;GO:0003676:nucleic acid binding	-

ppe-miR3627-5p	18788077	PRUPE_ppa012754mg	NCBI_Assembly:GCF_000346465.:	NW_006760384.1	+	289311	290031	720	pper03010:Ribosome	GO:0006412:translation	GO:0003735: structural constituent of ribosome	GO:0005840:ribosome
ppe-miR3627-5p	18788303	PRUPE_ppa026926mg	NCBI_Assembly:GCF_000346465.:	NW_006760385.1	+	8530432	8531975	1543	-	-	-	-
ppe-miR3627-5p	18788320	PRUPE_ppb022140mg	NCBI_Assembly:GCF_000346465.:	NW_006760385.1	-	32985396	32985789	393	-	-	-	-
ppe-miR3627-5p	18788403	PRUPE_ppa000674mg	NCBI_Assembly:GCF_000346465.:	NW_006760385.1	-	8441158	8454812	13654	pper04120:Ubiquitin mediated proteolysis	-	GO:0004842: ubiquitin-protein transferase activity;GO:0016874:ligase activity	-
ppe-miR3627-5p	18788404	PRUPE_ppa001264mg	NCBI_Assembly:GCF_000346465.:	NW_006760385.1	-	7738374	7747711	9337	-	-	-	-
ppe-miR3627-5p	18788505	PRUPE_ppa007423mg	NCBI_Assembly:GCF_000346465.:	NW_006760385.1	+	9658612	9660301	1689	-	-	GO:0003824: catalytic activity	GO:0005634:nucleus;GO:0005737:cytoplasm
ppe-miR3627-5p	18788509	PRUPE_ppa018021mg	NCBI_Assembly:GCF_000346465.:	NW_006760385.1	-	28105319	28105591	272	-	-	-	-
ppe-miR3627-5p	18788533	PRUPE_ppb002474mg	NCBI_Assembly:GCF_000346465.:	NW_006760385.1	-	31080991	31084553	3562	-	-	GO:0003676: nucleic acid binding;GO:0046872:metal ion binding	-
ppe-miR3627-5p	18788577	PRUPE_ppa000696mg	NCBI_Assembly:GCF_000346465.:	NW_006760385.1	-	3254874	3258683	3809	-	-	-	-
ppe-miR3627-5p	18788727	PRUPE_ppa019796mg	NCBI_Assembly:GCF_000346465.:	NW_006760385.1	+	34480304	34483789	3485	-	-	-	-
ppe-miR3627-5p	18788734	PRUPE_ppa000838mg	NCBI_Assembly:GCF_000346465.:	NW_006760385.1	+	14102982	14106048	3066	-	GO:0002237:response to molecule of bacterial origin;GO:0007165:signal transduction; GO:0010103: stomatal complex morphogenesis;GO:0048443:stamen development	GO:0004672: protein kinase activity;GO:0005524:ATP binding	GO:0016021:integral component of membrane
ppe-miR3627-5p	18788786	PRUPE_ppa026749mg	NCBI_Assembly:GCF_000346465.:	NW_006760385.1	+	46057197	46058963	1766	-	GO:0006606: protein import into nucleus	-	GO:0005622:intracellular

ppe-miR3627-5p	18788856	PRUPE_ppa002827mg	NCBI_Assembly:GCF_000346465.:	NW_006760385.1	-	9613794	9618828	5034	pper03008:Ribosome biogenesis in eukaryotes	GO:0000478:endonucleolytic cleavage involved in rRNA processing;GO:0009220:pyrimidine ribonucleotide biosynthetic process;GO:0009909:regulation of flower development	-	-
ppe-miR3627-5p	18788887	PRUPE_ppb024283mg	NCBI_Assembly:GCF_000346465.:	NW_006760385.1	-	36314798	36318511	3713	-	GO:0006351:transcription, DNA-templated;GO:0006355:regulation of transcription, DNA-templated	GO:0003677:DNA binding	GO:0005634:nucleus
ppe-miR3627-5p	18788893	PRUPE_ppa013749mg	NCBI_Assembly:GCF_000346465.:	NW_006760385.1	+	1680907	1684569	3662	-	GO:0007186:G-protein coupled receptor signaling pathway;GO:0009845:seed germination;GO:0010540:basipetal auxin transport;GO:0018342:protein prenylation;GO:0018345:protein palmitoylation;GO:0048527:lateral root development	GO:0004871:signal transducer activity	GO:0005834:heterotrimeric G-protein complex

ppe-miR3627-5p	18789007	PRUPE_ppa002669mg	NCBI_Assembly:GCF_000346465.:	NW_006760385.1	-	2748383	2753025	4642	pper04712:Circadian rhythm - plant	GO:0006281:DNA repair;GO:0006338:chromatin remodeling;GO:0009414:response to water deprivation;GO:0009638:phototropism;GO:0009911:positive regulation of flower development;GO:0010075:regulation of meristem growth;GO:0010118:stomatal movement;GO:0010617:circadian regulation of calcium ion oscillation;GO:0042744:hydrogen peroxide catabolic process	GO:0003913:DNA photolyase activity;GO:0009882:blue light photoreceptor activity	GO:0005773:vacuole;GO:0005829:cytosol

ppe-miR3627-5p	18789024	PRUPE_ppa004079mg	NCBI_Assembly:GCF_000346465.:	NW_006760385.1	+	37047480	37051363	3883	-	GO:0001731:formation of translation preinitiation complex;GO:0006446:regulation of translational initiation	GO:0003743:translation initiation factor activity	GO:0005852:eukaryotic translation initiation factor 3 complex;GO:0016282:eukaryotic 43S preinitiation complex;GO:0033290:eukaryotic 48S preinitiation complex
ppe-miR3627-5p	18789164	PRUPE_ppa011392mg	NCBI_Assembly:GCF_000346465.:	NW_006760385.1	-	39729066	39730055	989	-	GO:0010258: NADH dehydrogenase complex (plastoquinone) assembly	GO:0016655: oxidoreductase activity, acting on NAD(P)H, quinone or similar compound as acceptor	GO:0010598:NAD(P)H dehydrogenase complex (plastoquinone)
ppe-miR3627-5p	18789236	PRUPE_ppa002379mg	NCBI_Assembly:GCF_000346465.:	NW_006760385.1	+	42467125	42470565	3440	-	-	GO:0008270: zinc ion binding	-
ppe-miR3627-5p	18789246	PRUPE_ppa1027199mg	NCBI_Assembly:GCF_000346465.:	NW_006760385.1	-	29532487	29534201	1714	-	GO:0006541: glutamine metabolic process	GO:0016787: hydrolase activity	-
ppe-miR3627-5p	18789254	PRUPE_ppa013405mg	NCBI_Assembly:GCF_000346465.:	NW_006760385.1	-	1734825	1735222	397	pper03040:Spliceosome	-	GO:0000166: nucleotide binding;GO:0003676:nucleic acid binding	-
ppe-miR3627-5p	18789302	PRUPE_ppa008965mg	NCBI_Assembly:GCF_000346465.:	NW_006760385.1	-	36394584	36397530	2946	pper03050:Proteasome	-	-	-
ppe-miR3627-5p	18789348	PRUPE_ppa021895mg	NCBI_Assembly:GCF_000346465.:	NW_006760385.1	+	29836077	29837105	1028	-	-	-	-

ppe-miR3627-5p	18789685	PRUPE_ppa004420mg	NCBI_Assembly:GCF_000346465.:	NW_006760385.1	+	11915952	11917893	1941	-	-	GO:0022891: substrate-specific transmembrane transporter activity	GO:0016021:integral component of membrane
ppe-miR3627-5p	18789875	PRUPE_ppa026955mg	NCBI_Assembly:GCF_000346465.:	NW_006760385.1	+	33853255	33853783	528	-	-	GO:0009055: electron carrier activity	-
ppe-miR3627-5p	18789999	PRUPE_ppa015046mg	NCBI_Assembly:GCF_000346465.:	NW_006760385.1	+	6422414	6422923	509	-	GO:0048765:root hair cell differentiation	-	GO:0005576:extracellular region
ppe-miR3627-5p	18790123	PRUPE_ppa008028mg	NCBI_Assembly:GCF_000346465.:	NW_006760385.1	-	24773412	24775592	2180	-	GO:0015996: chlorophyll catabolic process	-	GO:0009535:chloroplast thylakoid membrane
ppe-miR3627-5p	18790182	PRUPE_ppa016917mg	NCBI_Assembly:GCF_000346465.:	NW_006760385.1	+	43634196	43636388	2192	pper00040:Pentose and glucuronate interconversions	GO:0045490: pectin catabolic process	GO:0030570: pectate lyase activity;GO:0046872:metal ion binding	-
ppe-miR3627-5p	18790191	PRUPE_ppa011195mg	NCBI_Assembly:GCF_000346465.:	NW_006760385.1	-	8269998	8271069	1071	pper00360:Phenylalanine metabolism;pper01100:Metabolic pathways;pper01110:Biosynthesis of secondary metabolites;pper00940:Phenylpropanoid biosynthesis	GO:0009269:response to desiccation;GO:0010231:maintenance of seed dormancy	GO:0008379:thioredoxin peroxidase activity	-
ppe-miR3627-5p	18790218	PRUPE_ppa008690mg	NCBI_Assembly:GCF_000346465.:	NW_006760385.1	+	40493805	40497727	3922	-	-	GO:0002161: aminoacyl-tRNA editing activity	GO:0005829:cytosol
ppe-miR3627-5p	18790281	PRUPE_ppa005300mg	NCBI_Assembly:GCF_000346465.:	NW_006760385.1	+	26360705	26364644	3939	-	-	-	-

ppe-miR3627-5p	18790332	PRUPE_ppa011014mg	NCBI_Assembly:GCF_000346465.:	NW_006760385.1	+	27134558	27136178	1620	-	-	GO:0003723:RNA binding;GO:0033897:ribonuclease T2 activity	-
ppe-miR3627-5p	18790418	PRUPE_ppa009338mg	NCBI_Assembly:GCF_000346465.:	NW_006760385.1	+	32176940	32179341	2401	-	GO:0000165:MAPK cascade;GO:0006333:chromatin assembly or disassembly;GO:0006612:protein targeting to membrane;GO:0009617:response to bacterium;GO:0009862:systemic acquired resistance, salicylic acid mediated signaling pathway;GO:0009867:jasmonic acid mediated signaling pathway;GO:0009965:leaf morphogenesis;GO:0010310:regulation of hydrogen peroxide metabolic process;GO:0010363:regulation of plant-type hypersensitive response;GO:	GO:0003677:DNA binding;GO:0003700:sequence-specific DNA binding transcription factor activity	GO:0005634:nucleus

ppe-miR3627-5p	18790563	PRUPE_ppa009486mg	NCBI_Assembly:GCF_000346465.:	NW_006760385.1	-	37822094	37825641	3547	pper04120:Ubiquitin mediated proteolysis	-	GO:0008270:zinc ion binding	-
ppe-miR3627-5p	18790627	PRUPE_ppa019311mg	NCBI_Assembly:GCF_000346465.:	NW_006760385.1	-	45712753	45714033	1280	-	GO:0006351:transcription, DNA-templated;GO:0006355:regulation of transcription, DNA-templated	GO:0003677:DNA binding	GO:0005634:nucleus
ppe-miR3627-5p	18790805	PRUPE_ppa021860mg	NCBI_Assembly:GCF_000346465.:	NW_006760385.1	+	43640304	43642373	2069	pper00040:Pentose and glucuronate interconversions	GO:0045490:pectin catabolic process	GO:0030570:pectate lyase activity;GO:0046872:metal ion binding	-
ppe-miR3627-5p	18790812	PRUPE_ppa006218mg	NCBI_Assembly:GCF_000346465.:	NW_006760385.1	+	42590792	42597221	6429	-	-	GO:0004672:protein kinase activity;GO:0005524:ATP binding	-
ppe-miR3627-5p	18790815	PRUPE_ppa005656mg	NCBI_Assembly:GCF_000346465.:	NW_006760385.1	+	32619665	32623160	3495	-	GO:0000278:mitotic cell cycle;GO:0006606:protein import into nucleus;GO:0009220:pyrimidine ribonucleotide biosynthetic process;GO:0030488:tRNA methylation	-	GO:0031515:tRNA (m1A) methyltransferase complex
ppe-miR3627-5p	18790829	PRUPE_ppa006081mg	NCBI_Assembly:GCF_000346465.:	NW_006760385.1	-	36963334	36968122	4788	-	-	GO:0004674:protein serine/threonine kinase activity;GO:0005524:ATP binding	-

ppe-miR3627-5p	18790853	PRUPE_ppa003953mg	NCBI_Assembly:GCF_000346465.:	NW_006760385.1	+	32424448	32428492	4044	-	GO:0009306: protein secretion	-	GO:0005623:cell
ppe-miR3627-5p	18790855	PRUPE_ppa001141mg	NCBI_Assembly:GCF_000346465.:	NW_006760385.1	-	44638502	44649769	11267	-	GO:0006487: protein N-linked glycosylation; GO:0007033: vacuole organization; GO:0007155: cell adhesion;GO:0009826:unidimensional cell growth;GO:0009832:plant-type cell wall biogenesis;GO:0010090:trichome morphogenesis;GO:0010413:glucuronoxylan metabolic process;GO:0032957:inositol trisphosphate metabolic process;GO:0045010:actin nucleation;GO:0045492:xylan biosynthetic process;GO:0046855:inositol phosphate dephosphorylation;GO:0048765:root hair cell	GO:0043813: phosphatidylinositol-3,5-bisphosphate 5-phosphatase activity	-
ppe-miR3627-5p	18790860	PRUPE_ppa008950mg	NCBI_Assembly:GCF_000346465.:	NW_006760385.1	+	33304911	33307843	2932	-	-	-	-

ppe-miR3627-5p	18790989	PRUPE_ppa019787mg	NCBI_Assembly:GCF_000346465.:	NW_006760385.1	-	34879415	34882016	2601	pper01100:Metabolic pathways;pper00591:Linoleic acid metabolism	-	GO:0004497: monooxygenase activity;GO:0005506:iron ion binding;GO:0016705:oxidoreductase activity, acting on paired donors, with incorporation or reduction of molecular oxygen;GO:0020037:heme binding	-
ppe-miR3627-5p	18791024	PRUPE_ppa025315mg	NCBI_Assembly:GCF_000346465.:	NW_006760385.1	-	6924475	6924906	431	-	-	-	-
ppe-miR3627-5p	18791051	PRUPE_ppa020774mg	NCBI_Assembly:GCF_000346465.:	NW_006760385.1	-	13433132	13439500	6368	-	-	-	-
ppe-miR3627-5p	18791096	PRUPE_ppa021804mg	NCBI_Assembly:GCF_000346465.:	NW_006760385.1	+	27015910	27017664	1754	-	-	-	-
ppe-miR3627-5p	18791193	PRUPE_ppa018784mg	NCBI_Assembly:GCF_000346465.:	NW_006760385.1	-	36646371	36646706	335	-	-	-	GO:0012511:monolayer-surrounded lipid storage body;GO:0016021:integral component of membrane
ppe-miR3627-5p	18791198	PRUPE_ppa024413mg	NCBI_Assembly:GCF_000346465.:	NW_006760385.1	+	43990695	43991615	920	-	-	-	-
ppe-miR3627-5p	18791275	PRUPE_ppa025635mg	NCBI_Assembly:GCF_000346465.:	NW_006760385.1	+	28049572	28050674	1102	-	-	-	-
ppe-miR3627-5p	18791286	PRUPE_ppa005996mg	NCBI_Assembly:GCF_000346465.:	NW_006760385.1	+	25342080	25345325	3245	pper01100:Metabolic pathways;pper00770:Pantothenate and CoA biosynthesis	GO:0015937: coenzyme A biosynthetic process	GO:0004594: pantothenate kinase activity;GO:0005524:ATP binding	-

ppe-miR3627-5p	18791329	PRUPE_ppa015518mg	NCBI_Assembly:GCF_000346465.:	NW_006760385.1	+	2546914	2548414	1500	-	-	GO:0005506:iron ion binding;GO:0016706:oxidoreductase activity, acting on paired donors, with incorporation or reduction of molecular oxygen, 2-oxoglutarate as one donor, and incorporation of one atom each of oxygen into both donors	-
ppe-miR3627-5p	18791362	PRUPE_ppb018005mg	NCBI_Assembly:GCF_000346465.:	NW_006760385.1	+	14715648	14716239	591	-	GO:0000723:telomere maintenance;GO:0006281:DNA repair	GO:0016887:ATPase activity	GO:0030870:Mre11 complex
ppe-miR3627-5p	18791417	PRUPE_ppb012900mg	NCBI_Assembly:GCF_000346465.:	NW_006760385.1	-	11668024	11670213	2189	pper01100:Metabolic pathways;pper01110:Biosynthesis of secondary metabolites;pper00500:Starch and sucrose metabolism;pper00520:Amino sugar and nucleotide sugar metabolism	GO:0005978:glycogen biosynthetic process;GO:0019252:starch biosynthetic process	GO:0008878:glucose-1-phosphate adenylyltransferase activity	GO:0009507:chloroplast

ppe-miR3627-5p	18791436	PRUPE_ppa000861mg	NCBI_Assembly:GCF_000346465.:	NW_006760385.1	+	5520989	5529996	9007	-	GO:0051017: actin filament bundle assembly	-	-
ppe-miR3627-5p	18791451	PRUPE_ppa011820mg	NCBI_Assembly:GCF_000346465.:	NW_006760385.1	+	35874450	35876756	2306	-	-	-	GO:0005634:nucleus
ppe-miR3627-5p	18791456	PRUPE_ppa000165mg	NCBI_Assembly:GCF_000346465.:	NW_006760385.1	-	34484342	34494286	9944	-	GO:0006396: RNA processing	GO:0003723: RNA binding;GO:0004525:ribonuclease III activity;GO:0005524:ATP binding	-
ppe-miR3627-5p	18791520	PRUPE_ppa004366mg	NCBI_Assembly:GCF_000346465.:	NW_006760385.1	+	26370161	26374199	4038	-	-	-	-
ppe-miR3627-5p	18791525	PRUPE_ppa003844mg	NCBI_Assembly:GCF_000346465.:	NW_006760385.1	+	33397729	33401141	3412	-	-	GO:0003676: nucleic acid binding;GO:0046872:metal ion binding	-
ppe-miR3627-5p	18791540	PRUPE_ppa010880mg	NCBI_Assembly:GCF_000346465.:	NW_006760385.1	+	35982026	35984734	2708	-	GO:0006457: protein folding;GO:0009408:response to heat;GO:0009644:response to high light intensity;GO:0042542:response to hydrogen peroxide	-	-
ppe-miR3627-5p	18791590	PRUPE_ppa014856mg	NCBI_Assembly:GCF_000346465.:	NW_006760385.1	-	29858367	29860726	2359	-	-	-	-
ppe-miR3627-5p	18791592	PRUPE_ppa009896mg	NCBI_Assembly:GCF_000346465.:	NW_006760385.1	-	23252758	23255748	2990	pper03050:Proteasome	GO:0051603: proteolysis involved in cellular protein catabolic process	GO:0004298:threonine-type endopeptidase activity	GO:0005634:nucleus;GO:0005737:cytoplasm;GO:0005839:proteasome core complex
ppe-miR3627-5p	18791639	PRUPE_ppa014280mg	NCBI_Assembly:GCF_000346465.:	NW_006760385.1	+	22874155	22875511	1356	-	-	-	GO:0005783:endoplasmic reticulum

ppe-miR3627-5p	18791703	PRUPE_ppa004739mg	NCBI_Assembly:GCF_000346465.:	NW_006760385.1	-	42797851	42801888	4037	-	-	-	-
ppe-miR3627-5p	18791758	PRUPE_ppa007937mg	NCBI_Assembly:GCF_000346465.:	NW_006760385.1	+	5536662	5542444	5782	pper01100:Metabolic pathways;pper01110:Biosynthesis of secondary metabolites;pper00010:Glycolysis / Gluconeogenesis	GO:0005975: carbohydrate metabolic process	GO:0016853:isomerase activity;GO:0030246:carbohydrate binding	-
ppe-miR3627-5p	18791861	PRUPE_ppa012145mg	NCBI_Assembly:GCF_000346465.:	NW_006760385.1	-	28681154	28682607	1453	-	-	GO:0008270: zinc ion binding	-

ppe-miR3627-5p	18791879	PRUPE_ppa002437mg	NCBI_Assembly:GCF_000346465.:	NW_006760385.1	-	33992452	33997486	5034	pper03022:Basal transcription factors	GO:0000394:RNA splicing, via endonucleolytic cleavage and ligation;GO:0006355:regulation of transcription, DNA-templated;GO:0006366:transcription from RNA polymerase II promoter;GO:0007062:sister chromatid cohesion;GO:0007131:reciprocal meiotic recombination;GO:0033044:regulation of chromosome organization;GO:0042138:meiotic DNA double-strand break formation;GO:0045132:meiotic chromosome segregation	-	GO:0005634:nucleus
ppe-miR3627-5p	18791885	PRUPE_ppa008231mg	NCBI_Assembly:GCF_000346465.:	NW_006760385.1	-	36300916	36301938	1022	pper04075:Plant hormone signal transduction	GO:0010423:negative regulation of brassinosteroid biosynthetic process	-	GO:0005829:cytosol
ppe-miR3627-5p	18791897	PRUPE_ppa014156mg	NCBI_Assembly:GCF_000346465.:	NW_006760385.1	-	41226362	41226984	622	-	-	-	-

ppe-miR3627-5p	18792083	PRUPE_ppa003277mg	NCBI_Assembly:GCF_000346465.:	NW_006760385.1	+		35042581	35048940	6359	-	GO:0006814: sodium ion transport;GO: 0010351:lithium ion transport	GO:0015299: solute:proton antiporter activity	GO:0016021:integral component of membrane
ppe-miR3627-5p	18792148	PRUPE_ppa000748mg	NCBI_Assembly:GCF_000346465.:	NW_006760385.1	-		34758812	34762296	3484	-	-	-	-
ppe-miR3627-5p	18792161	PRUPE_ppa005842mg	NCBI_Assembly:GCF_000346465.:	NW_006760385.1	-		32742295	32747092	4797	-	-	GO:0046872: metal ion binding	-

ppe-miR3627-5p	18792164	PRUPE_ppa000605mg	NCBI_Assembly:GCF_000346465.:	NW_006760385.1	-	32666249	32675535	9286	pper03008:Ribosome biogenesis in eukaryotes;pper03018:RNA degradation	GO:0006635:fatty acid beta-oxidation;GO:0007062:sister chromatid cohesion;GO:0007131:reciprocal meiotic recombination;GO:0009887:organ morphogenesis;GO:0009888:tissue development;GO:0010332:response to gamma radiation;GO:0010587:miRNA catabolic process;GO:0010638:positive regulation of organelle organization;GO:0016558:protein import into peroxisome matrix;GO:0031048:chromatin silencing by small RNA;GO:0032204:regulation of telomere maintenance;GO:0042138:	GO:0003676:nucleic acid binding;GO:0004534:5'-3' exoribonuclease activity;GO:0008270:zinc ion binding	GO:0005634:nucleus
ppe-miR3627-5p	18792217	PRUPE_ppa011723mg	NCBI_Assembly:GCF_000346465.:	NW_006760385.1	+	27235548	27236861	1313	-	-	-	-
ppe-miR3627-5p	18792253	PRUPE_ppa001493mg	NCBI_Assembly:GCF_000346465.:	NW_006760385.1	-	9174337	9182385	8048	-	-	GO:0003677:DNA binding	GO:0005634:nucleus
ppe-miR3627-5p	18792268	PRUPE_ppa025233mg	NCBI_Assembly:GCF_000346465.:	NW_006760385.1	-	8663333	8663932	599	-	-	-	-
ppe-miR3627-5p	18792281	PRUPE_ppa024231mg	NCBI_Assembly:GCF_000346465.:	NW_006760385.1	-	12795066	12797665	2599	-	-	-	-
ppe-miR3627-5p	18792659	PRUPE_ppa023525mg	NCBI_Assembly:GCF_000346465.:	NW_006760385.1	-	38712607	38713608	1001	-	-	-	-

ppe-miR3627-5p	18792671	PRUPE_ppa000527mg	NCBI_Assembly:GCF_000346465.:	NW_006760385.1	-	46477711	46485085	7374	-	GO:0000956: nuclear-transcribed mRNA catabolic process;GO:0006487:protein N-linked glycosylation; GO:0006511: ubiquitin-dependent protein catabolic process;GO:0008283:cell proliferation;GO:0009908:flower development; GO:0016579: protein deubiquitination;GO:0048364:root development; GO:0048366:leaf development; GO:1901000:regulation of response to salt stress	GO:0036459: ubiquitinyl hydrolase activity;GO:0046872:metal ion binding	-
ppe-miR3627-5p	18792683	PRUPE_ppa021787mg	NCBI_Assembly:GCF_000346465.:	NW_006760385.1	-	14797629	14798291	662	-	-	-	-
ppe-miR3627-5p	18792758	PRUPE_ppa012903mg	NCBI_Assembly:GCF_000346465.:	NW_006760385.1	-	32710459	32711025	566	pper04075:Plant hormone signal transduction	-	-	-

ppe-miR3627-5p	18792822	PRUPE_ppa013136mg	NCBI_Assembly:GCF_000346465.:	NW_006760385.1	-	45077348	45079614	2266	-	GO:0045036: protein targeting to chloroplast	-	GO:0009535:chloroplast thylakoid membrane;GO:0009941:chloroplast envelope
ppe-miR3627-5p	18793074	PRUPE_ppa013170mg	NCBI_Assembly:GCF_000346465.:	NW_006760385.1	+	32553886	32554449	563	-	-	-	-
ppe-miR3627-5p	18793099	PRUPE_ppa025373mg	NCBI_Assembly:GCF_000346465.:	NW_006760385.1	+	25790323	25792420	2097	-	-	GO:0022857:transmembrane transporter activity	GO:0016021:integral component of membrane
ppe-miR3627-5p	18793118	PRUPE_ppa023232mg	NCBI_Assembly:GCF_000346465.:	NW_006760385.1	+	12097963	12098870	907	-	-	-	-
ppe-miR3627-5p	18793130	PRUPE_ppa002057mg	NCBI_Assembly:GCF_000346465.:	NW_006760385.1	+	4526454	4533022	6568	pper03450:Non-homologous end-joining	GO:0006310: DNA recombination;GO:0051103: DNA ligation involved in DNA repair	GO:0003677: DNA binding;GO:0003910:DNA ligase (ATP) activity;GO:0005524:ATP binding	-
ppe-miR3627-5p	18793345	PRUPE_ppa008679mg	NCBI_Assembly:GCF_000346465.:	NW_006760385.1	-	8695063	8698489	3426	-	GO:0006470: protein dephosphorylation	GO:0004722: protein serine/threonine phosphatase activity;GO:0046872:metal ion binding	-
ppe-miR3627-5p	18793350	PRUPE_ppa009961mg	NCBI_Assembly:GCF_000346465.:	NW_006760385.1	+	37118692	37121814	3122	-	GO:0000917: barrier septum assembly;GO:0006364:rRNA processing;GO:0006399:tRNA metabolic process;GO:0009658:chloroplast organization	GO:0005525: GTP binding	-
ppe-miR3627-5p	18793438	PRUPE_ppa025377mg	NCBI_Assembly:GCF_000346465.:	NW_006760385.1	-	39018821	39020551	1730	-	-	-	-

ppe-miR3627-5p	18793499	PRUPE_ppa024593mg	NCBI_Assembly:GCF_000346465.:	NW_006760385.1	+	43926049	43927846	1797	-	-	-	-
ppe-miR3627-5p	18793609	PRUPE_ppa016563mg	NCBI_Assembly:GCF_000346465.:	NW_006760385.1	-	36508257	36511249	2992	-	-	GO:0003676: nucleic acid binding	-
ppe-miR3627-5p	18793614	PRUPE_ppa021685mg	NCBI_Assembly:GCF_000346465.:	NW_006760385.1	-	33714750	33717233	2483	-	GO:0080060:integument development	GO:0003682: chromatin binding;GO:0003700:sequence-specific DNA binding transcription factor activity;GO:0044212:transcription regulatory region DNA binding	GO:0005618:cell wall
ppe-miR3627-5p	18793617	PRUPE_ppa019389mg	NCBI_Assembly:GCF_000346465.:	NW_006760385.1	-	33363433	33364781	1348	-	GO:0048868: pollen tube development	-	-
ppe-miR3627-5p	18793629	PRUPE_ppa011623mg	NCBI_Assembly:GCF_000346465.:	NW_006760385.1	-	35105407	35108453	3046	-	-	-	-
ppe-miR3627-5p	18793733	PRUPE_ppa022389mg	NCBI_Assembly:GCF_000346465.:	NW_006760385.1	+	13024467	13026277	1810	-	-	GO:0003676: nucleic acid binding	-
ppe-miR3627-5p	18793794	PRUPE_ppa010875mg	NCBI_Assembly:GCF_000346465.:	NW_006760385.1	-	16588039	16590462	2423	-	-	-	-
ppe-miR3627-5p	18793825	PRUPE_ppa002160mg	NCBI_Assembly:GCF_000346465.:	NW_006760385.1	-	43574616	43591392	16776	-	-	-	-
ppe-miR3627-5p	18793963	PRUPE_ppa002452mg	NCBI_Assembly:GCF_000346465.:	NW_006760385.1	-	29450610	29453390	2780	-	-	GO:0004672: protein kinase activity;GO:0005524:ATP binding	GO:0016021:integral component of membrane
ppe-miR3627-5p	18794051	PRUPE_ppa025732mg	NCBI_Assembly:GCF_000346465.:	NW_006760385.1	-	26645918	26649506	3588	-	-	GO:0003676: nucleic acid binding;GO:0008270:zinc ion binding	-
ppe-miR395d	18765973	PRUPE_ppa009429mg	NCBI_Assembly:GCF_000346465.:	NW_006760186.1	-	601147	607633	6486	-	-	GO:0008270: zinc ion binding	-

ppe-miR395d	18766004	PRUPE_ppa002790mg	NCBI_Assembly:GCF_000346465.:	NW_006760186.1	+	568707	573299	4592	-	GO:0007067: mitotic nuclear division	-	GO:0005819:spindle;GO:0005874:microtubule
ppe-miR395d	18766083	PRUPE_ppa012918mg	NCBI_Assembly:GCF_000346465.:	NW_006760186.1	-	1267515	1268921	1406	pper03030:DNA replication;pper03420:Nucleotide excision repair;pper03430:Mismatch repair;pper03440:Homologous recombination	-	-	-
ppe-miR395d	18766242	PRUPE_ppa008037mg	NCBI_Assembly:GCF_000346465.:	NW_006760194.1	+	4336639	4340550	3911	pper01100:Metabolic pathways;pper00564:Glycerophospholipid metabolism	GO:0046474: glycerophospholipid biosynthetic process	GO:0016301: kinase activity	-
ppe-miR395d	18766279	PRUPE_ppa024159mg	NCBI_Assembly:GCF_000346465.:	NW_006760194.1	-	15109425	15111343	1918	-	-	-	-
ppe-miR395d	18766395	PRUPE_ppa004526mg	NCBI_Assembly:GCF_000346465.:	NW_006760194.1	-	15163721	15166055	2334	-	GO:0015996: chlorophyll catabolic process	GO:0015171: amino acid transmembrane transporter activity;GO:0015203:polyamine transmembrane transporter activity	GO:0016021:integral component of membrane
ppe-miR395d	18766411	PRUPE_ppa024841mg	NCBI_Assembly:GCF_000346465.:	NW_006760194.1	+	15576031	15577650	1619	-	-	-	-

ppe-miR395d	18766436	PRUPE_ppa004090mg	NCBI_Assembly:GCF_000346465.:	NW_006760194.1	-	1073236	1077570	4334	pper01100:Metabolic pathways;pper01110:Biosynthesis of secondary metabolites;pper01230:Biosynthesis of amino acids;pper00460:Cyanoamino acid metabolism;pper00260:Glycine, serine and threonine metabolism;pper01200:Carbon metabolism;pper00630:Glyoxylate and dicarboxylate metabolism;pper00670:One carbon pool by folate	GO:0006544: glycine metabolic process;GO:0006563:L-serine metabolic process;GO:0035999:tetrahydrofolate interconversion	GO:0004372: glycine hydroxymethyltransferase activity;GO:0030170:pyridoxal phosphate binding	-
ppe-miR395d	18766622	PRUPE_ppa026489mg	NCBI_Assembly:GCF_000346465.:	NW_006760194.1	+	17738551	17742009	3458	pper01100:Metabolic pathways;pper00591:Linoleic acid metabolism;pper00592:alpha-Linolenic acid metabolism	GO:0031408: oxylipin biosynthetic process	GO:0016702: oxidoreductase activity, acting on single donors with incorporation of molecular oxygen, incorporation of two atoms of oxygen;GO:0046872:metal ion binding	-

ppe-miR395d	18766905	PRUPE_ppa010238mg	NCBI_Assembly:GCF_000346465.:	NW_006760194.1	-	17710283	17713864	3581	-	GO:0006626: protein targeting to mitochondrion	-	GO:0005622:intracellular
ppe-miR395d	18766984	PRUPE_ppa002521mg	NCBI_Assembly:GCF_000346465.:	NW_006760194.1	+	15189170	15194284	5114	-	-	-	-
ppe-miR395d	18767040	PRUPE_ppa003275mg	NCBI_Assembly:GCF_000346465.:	NW_006760194.1	-	17767541	17771857	4316	-	-	GO:0005507: copper ion binding;GO:0016491:oxidoreductase activity	-
ppe-miR395d	18767124	PRUPE_ppa009911mg	NCBI_Assembly:GCF_000346465.:	NW_006760194.1	-	16124715	16127302	2587	-	GO:0006621: protein retention in ER lumen	GO:0046923: ER retention sequence binding	GO:0005783:endoplasmic reticulum;GO:0016021:integral component of membrane
ppe-miR395d	18767201	PRUPE_ppa001167mg	NCBI_Assembly:GCF_000346465.:	NW_006760194.1	-	14516906	14520665	3759	-	GO:0006612: protein targeting to membrane;GO:0009963:positive regulation of flavonoid biosynthetic process;GO:0010363:regulation of plant-type hypersensitive response	-	GO:0005622:intracellular;GO:0005886:plasma membrane
ppe-miR395d	18767277	PRUPE_ppa014595mg	NCBI_Assembly:GCF_000346465.:	NW_006760194.1	+	12311873	12312839	966	-	-	-	-
ppe-miR395d	18767294	PRUPE_ppa021363mg	NCBI_Assembly:GCF_000346465.:	NW_006760194.1	+	15369063	15371337	2274	-	GO:0005975: carbohydrate metabolic process	GO:0004553: hydrolase activity, hydrolyzing O-glycosyl compounds	-
ppe-miR395d	18767361	PRUPE_ppa022741mg	NCBI_Assembly:GCF_000346465.:	NW_006760194.1	+	18362540	18363295	755	-	-	-	-

ppe-miR395d	18767547	PRUPE_ppa019221mg	NCBI_Assembly:GCF_000346465.:	NW_006760194.1	+	14405645	14408015	2370	-	GO:0006629:lipid metabolic process	GO:0008081:phosphoric diester hydrolase activity	-
ppe-miR395d	18767822	PRUPE_ppa000722m1g	NCBI_Assembly:GCF_000346465.:	NW_006760194.1	+	13464962	13469159	4197	-	-	-	GO:0005829:cytosol
ppe-miR395d	18768061	PRUPE_ppa012240mg	NCBI_Assembly:GCF_000346465.:	NW_006760194.1	-	14806669	14808745	2076	-	-	-	-
ppe-miR395d	18768202	PRUPE_ppa001622mg	NCBI_Assembly:GCF_000346465.:	NW_006760194.1	+	279145	284899	5754	-	GO:0006265:DNA topological change	GO:0003677:DNA binding;GO:0003917:DNA topoisomerase type I activity;GO:0003918:DNA topoisomerase type II (ATP-hydrolyzing) activity	GO:0005694:chromosome
ppe-miR395d	18768237	PRUPE_ppa011000mg	NCBI_Assembly:GCF_000346465.:	NW_006760194.1	+	20173614	20176339	2725	-	-	GO:0004674:protein serine/threonine kinase activity;GO:0005524:ATP binding	-
ppe-miR395d	18768342	PRUPE_ppb024531mg	NCBI_Assembly:GCF_000346465.:	NW_006760194.1	+	3139028	3139845	817	pper01100:Metabolic pathways;pper01110:Biosynthesis of secondary metabolites;pper00520:Amino sugar and nucleotide sugar metabolism;pper00051:Fructose and mannose metabolism	GO:0009298:GDP-mannose biosynthetic process;GO:0019307:mannose biosynthetic process	GO:0004615:phosphomannomutase activity	GO:0005737:cytoplasm

ppe-miR395d	18768354	PRUPE_ppa006970mg	NCBI_Assembly:GCF_000346465.:	NW_006760194.1	-	2189686	2191207	1521	-	GO:0006826:iron ion transport	-	GO:0009941:chloroplast envelope;GO:0016021:integral component of membrane
ppe-miR395d	18768585	PRUPE_ppa001449mg	NCBI_Assembly:GCF_000346465.:	NW_006760194.1	+	15075595	15078075	2480	-	-	-	-
ppe-miR395d	18768676	PRUPE_ppa009275mg	NCBI_Assembly:GCF_000346465.:	NW_006760194.1	-	19816717	19818077	1360	-	GO:0006334:nucleosome assembly	GO:0003677:DNA binding	GO:0000786:nucleosome;GO:0005634:nucleus
ppe-miR395d	18768855	PRUPE_ppa017519mg	NCBI_Assembly:GCF_000346465.:	NW_006760194.1	+	6131193	6132599	1406	-	GO:0006351:transcription, DNA-templated;GO:0006355:regulation of transcription, DNA-templated	-	-
ppe-miR395d	18768881	PRUPE_ppa010784mg	NCBI_Assembly:GCF_000346465.:	NW_006760194.1	-	12279538	12280787	1249	-	-	-	-
ppe-miR395d	18768995	PRUPE_ppa012821mg	NCBI_Assembly:GCF_000346465.:	NW_006760194.1	+	2798301	2799216	915	-	-	-	-
ppe-miR395d	18769161	PRUPE_ppa012454mg	NCBI_Assembly:GCF_000346465.:	NW_006760201.1	+	15608087	15609932	1845	pper04626:Plant-pathogen interaction	-	GO:0005509:calcium ion binding	-
ppe-miR395d	18769231	PRUPE_ppa007628mg	NCBI_Assembly:GCF_000346465.:	NW_006760201.1	+	16930198	16933784	3586	pper01100:Metabolic pathways;pper01110:Biosynthesis of secondary metabolites;pper00010:Glycolysis / Gluconeogenesis	GO:0005975:carbohydrate metabolic process	GO:0016853:isomerase activity;GO:0030246:carbohydrate binding	GO:0009570:chloroplast stroma;GO:0048046:apoplast
ppe-miR395d	18769274	PRUPE_ppa015779mg	NCBI_Assembly:GCF_000346465.:	NW_006760201.1	+	17155191	17155656	465	-	-	-	-
ppe-miR395d	18769344	PRUPE_ppa011052mg	NCBI_Assembly:GCF_000346465.:	NW_006760201.1	-	16461498	16463069	1571	pper04141:Protein processing in endoplasmic reticulum	GO:0006661:phosphatidylinositol biosynthetic process	GO:0008270:zinc ion binding	-
ppe-miR395d	18769621	PRUPE_ppa007474mg	NCBI_Assembly:GCF_000346465.:	NW_006760201.1	+	20207325	20208672	1347	-	-	-	-

ppe-miR395d	18769648	PRUPE_ppa014386mg	NCBI_Assembly:GCF_000346465.:	NW_006760201.1	-	4511844	4512658	814	-	-	-	-
ppe-miR395d	18769953	PRUPE_ppa026994mg	NCBI_Assembly:GCF_000346465.:	NW_006760201.1	-	13330674	13331185	511	-	GO:0006098: pentose-phosphate shunt;GO:0009073:aromatic amino acid family biosynthetic process;GO:0016226:iron-sulfur cluster assembly;GO:0045893:positive regulation of transcription, DNA-templated	-	GO:0009570:chloroplast stroma

ppe-miR395d	18770083	PRUPE_ppa000647mg	NCBI_Assembly:GCF_000346465.:	NW_006760201.1	-	20582610	20588923	6313	pper03430:Mismatch repair	GO:0000911:cytokinesis by cell plate formation;GO:0006270:DNA replication initiation;GO:0006275:regulation of DNA replication;GO:0006298:mismatch repair;GO:0006306:DNA methylation;GO:0007067:mitotic nuclear division;GO:0007129:synapsis;GO:0008283:cell proliferation;GO:0009909:regulation of flower development;GO:0010389:regulation of G2/M transition of mitotic cell cycle;GO:0016458:gene silencing;GO:0051567:histone H3-K9 methylation	GO:0005524:ATP binding;GO:0030983:mismatched DNA binding	-
ppe-miR395d	18770098	PRUPE_ppa018741mg	NCBI_Assembly:GCF_000346465.:	NW_006760201.1	+	13234455	13235180	725	-	-	-	-
ppe-miR395d	18770112	PRUPE_ppb013602mg	NCBI_Assembly:GCF_000346465.:	NW_006760201.1	+	12290746	12291666	920	-	-	-	-
ppe-miR395d	18770122	PRUPE_ppa005800mg	NCBI_Assembly:GCF_000346465.:	NW_006760201.1	+	11900066	11901882	1816	pper04120:Ubiquitin mediated proteolysis	-	-	-

ppe-miR395d	18770144	PRUPE_ppa022931mg	NCBI_Assembly:GCF_000346465.:	NW_006760201.1	-	20577784	20582043	4259	pper04144:Endocytosis	GO:0009827:plant-type cell wall modification;GO:0009846:pollen germination;GO:0009860:pollen tube growth	GO:0005086:ARF guanyl-nucleotide exchange factor activity	GO:0090406:pollen tube
ppe-miR395d	18770528	PRUPE_ppa019571mg	NCBI_Assembly:GCF_000346465.:	NW_006760201.1	-	20109536	20112716	3180	-	-	GO:0004674:protein serine/threonine kinase activity;GO:0005524:ATP binding	GO:0016021:integral component of membrane
ppe-miR395d	18770741	PRUPE_ppa016201mg	NCBI_Assembly:GCF_000346465.:	NW_006760201.1	+	2223778	2224323	545	-	-	-	-
ppe-miR395d	18770788	PRUPE_ppa024680mg	NCBI_Assembly:GCF_000346465.:	NW_006760201.1	+	3281893	3283162	1269	-	-	-	-
ppe-miR395d	18771598	PRUPE_ppa009437mg	NCBI_Assembly:GCF_000346465.:	NW_006760201.1	-	15261753	15263731	1978	-	GO:0006073:cellular glucan metabolic process;GO:0016998:cell wall macromolecule catabolic process;GO:0042546:cell wall biogenesis;GO:0071555:cell wall organization	GO:0004553:hydrolase activity, hydrolyzing O-glycosyl compounds;GO:0016762:xyloglucan:xyloglucosyl transferase activity	GO:0005618:cell wall;GO:0048046:apoplast
ppe-miR395d	18771682	PRUPE_ppa007189mg	NCBI_Assembly:GCF_000346465.:	NW_006760201.1	+	18223854	18225877	2023	-	-	-	-
ppe-miR395d	18771775	PRUPE_ppb010572mg	NCBI_Assembly:GCF_000346465.:	NW_006760201.1	+	2358432	2359456	1024	-	-	-	-

ppe-miR395d	18771839	PRUPE_ppa024442mg	NCBI_Assembly:GCF_000346465.:	NW_006760201.1	+	8659977	8662300	2323	pper00908:Zeatin biosynthesis	GO:0009823: cytokinin catabolic process;GO:0010089:xylem development; GO:0044036: cell wall macromolecule metabolic process;GO:0048507:meristem development	GO:0008762: UDP-N-acetylmuramate dehydrogenase activity;GO:0019139:cytokinin dehydrogenase activity;GO:0050660:flavin adenine dinucleotide binding	-
ppe-miR395d	18771880	PRUPE_ppb014730mg	NCBI_Assembly:GCF_000346465.:	NW_006760201.1	+	11058590	11059846	1256	-	-	GO:0043531: ADP binding	-

ppe-miR395d	18771926	PRUPE_ppa000944mg	NCBI_Assembly:GCF_000346465.:	NW_006760208.1	-	27412741	27419682	6941	-	GO:0002229:defense response to oomycetes;GO:0006487:protein N-linked glycosylation;GO:0008219:cell death;GO:0009414:response to water deprivation;GO:0009617:response to bacterium;GO:0009620:response to fungus;GO:0009723:response to ethylene;GO:0009788:negative regulation of abscisic acid-activated signaling pathway;GO:0046777:protein autophosphorylation;GO:1900150:regulation of defense response to fungus;GO:1900424:regulation of defense response to	GO:0004709:MAP kinase kinase kinase activity;GO:0004712:protein serine/threonine/tyrosine kinase activity;GO:0005524:ATP binding	GO:0005802:trans-Golgi network;GO:0005829:cytosol
ppe-miR395d	18772137	PRUPE_ppa001677mg	NCBI_Assembly:GCF_000346465.:	NW_006760208.1	-	22763017	22767119	4102	-	-	GO:0015299:solute:proton antiporter activity	GO:0016021:integral component of membrane
ppe-miR395d	18772217	PRUPE_ppa002217mg	NCBI_Assembly:GCF_000346465.:	NW_006760208.1	-	22910610	22913200	2590	-	-	GO:0003824:catalytic activity	-

ppe-miR395d	18772284	PRUPE_ppa004870mg	NCBI_Assembly:GCF_000346465.:	NW_006760208.1	+	25189032	25193057	4025	-	-	GO:0004674:protein serine/threonine kinase activity;GO:0005524:ATP binding	-
ppe-miR395d	18772312	PRUPE_ppa005524mg	NCBI_Assembly:GCF_000346465.:	NW_006760208.1	+	23087122	23091454	4332	-	-	-	-
ppe-miR395d	18772361	PRUPE_ppa019688mg	NCBI_Assembly:GCF_000346465.:	NW_006760208.1	+	284301	287525	3224	-	GO:0000165:MAPK cascade;GO:0006612:protein targeting to membrane;GO:0009595:detection of biotic stimulus;GO:0009697:salicylic acid biosynthetic process;GO:0009862:systemic acquired resistance, salicylic acid mediated signaling pathway;GO:0009867:jasmonic acid mediated signaling pathway;GO:0010200:response to chitin;GO:0010310:regulation of hydrogen peroxide metabolic process;GO:0010363:regulation of plant-type hypersensitive response;GO:	GO:0005315:inorganic phosphate transmembrane transporter activity;GO:0030504:inorganic diphosphate transmembrane transporter activity	GO:0005622:intracellular;GO:0016021:integral component of membrane

ppe-miR395d	18772362	PRUPE_ppa021634mg	NCBI_Assembly:GCF_000346465.:	NW_006760208.1	-	15802525	15803631	1106	-	-	-	-
ppe-miR395d	18772491	PRUPE_ppa008835mg	NCBI_Assembly:GCF_000346465.:	NW_006760208.1	-	21511337	21512335	998	-	-	-	-
ppe-miR395d	18772599	PRUPE_ppa025305mg	NCBI_Assembly:GCF_000346465.:	NW_006760208.1	+	21827888	21831934	4046	-	-	-	-
ppe-miR395d	18772604	PRUPE_ppa022064mg	NCBI_Assembly:GCF_000346465.:	NW_006760208.1	+	2661441	2661890	449	-	-	-	-
ppe-miR395d	18772628	PRUPE_ppa1027138mg	NCBI_Assembly:GCF_000346465.:	NW_006760208.1	-	26588870	26589472	602	-	-	-	-
ppe-miR395d	18772752	PRUPE_ppa004767mg	NCBI_Assembly:GCF_000346465.:	NW_006760208.1	-	7188788	7194025	5237	-	-	GO:0008146: sulfotransferase activity	GO:0016021:integral component of membrane
ppe-miR395d	18773101	PRUPE_ppa014548mg	NCBI_Assembly:GCF_000346465.:	NW_006760208.1	+	25654669	25655829	1160	-	GO:0009409:response to cold	-	GO:0016021:integral component of membrane
ppe-miR395d	18773211	PRUPE_ppa026614mg	NCBI_Assembly:GCF_000346465.:	NW_006760208.1	+	20122964	20123629	665	-	-	GO:0016758:transferase activity, transferring hexosyl groups	-
ppe-miR395d	18773323	PRUPE_ppa013158mg	NCBI_Assembly:GCF_000346465.:	NW_006760208.1	-	27507837	27509796	1959	pper03010:Ribosome	GO:0006412:translation	GO:0000166: nucleotide binding;GO:0003735:structural constituent of ribosome	GO:0005840:ribosome
ppe-miR395d	18773416	PRUPE_ppa009910mg	NCBI_Assembly:GCF_000346465.:	NW_006760208.1	-	13246594	13250421	3827	pper01100:Metabolic pathways;pper01110:Biosynthesis of secondary metabolites;pper01230:Biosynthesis of amino acids;pper00300:Lysine biosynthesis	GO:0009089:lysine biosynthetic process via diaminopimelate	GO:0008839: 4-hydroxy-tetrahydrodipicolinate reductase;GO:0070402:NADPH binding	-

ppe-miR395d	18773486	PRUPE_ppa001740mg	NCBI_Assembly:GCF_000346465.:	NW_006760208.1	-	13580380	13587267	6887	-	GO:0006486: protein glycosylation; GO:0006635:fatty acid beta-oxidation;GO:0016558:protein import into peroxisome matrix	-	GO:0005622:intracellular;GO:0005886:plasma membrane;GO:0009506:plasmodesma
ppe-miR395d	18773640	PRUPE_ppa005989mg	NCBI_Assembly:GCF_000346465.:	NW_006760208.1	-	9811755	9817089	5334	pper04144:Endocytosis	GO:0006623: protein targeting to vacuole;GO:0006635:fatty acid beta-oxidation;GO:0006869:lipid transport;GO:0006891:intra-Golgi vesicle-mediated transport;GO:0007032:endosome organization; GO:0007033: vacuole organization; GO:0010091:trichome branching;GO:0010351:lithium ion transport;GO:0016197:endosomal transport;GO:0016558:protein import into peroxisome matrix	GO:0005524: ATP binding	GO:0005634:nucleus;GO:0005771:multivesicular body;GO:0009506:plasmodesma

ppe-miR395d	18773734	PRUPE_ppa020769mg	NCBI_Assembly:GCF_000346465.:	NW_006760208.1	-	23154560	23157970	3410	-	-	-	GO:0016021:integral component of membrane
ppe-miR395d	18773813	PRUPE_ppa015954mg	NCBI_Assembly:GCF_000346465.:	NW_006760208.1	+	5716921	5717980	1059	-	-	GO:0003677: DNA binding;GO:0003682:chromatin binding	-
ppe-miR395d	18773836	PRUPE_ppb024045mg	NCBI_Assembly:GCF_000346465.:	NW_006760208.1	+	11564154	11564696	542	-	-	-	-
ppe-miR395d	18774282	PRUPE_ppa004341mg	NCBI_Assembly:GCF_000346465.:	NW_006760208.1	-	2340406	2343839	3433	-	-	GO:0004497: monooxygenase activity;GO:0005506:iron ion binding;GO:0016705:oxidoreductase activity, acting on paired donors, with incorporation or reduction of molecular oxygen;GO:0020037:heme binding	-
ppe-miR395d	18774358	PRUPE_ppa007468mg	NCBI_Assembly:GCF_000346465.:	NW_006760208.1	+	6986156	6988676	2520	-	-	GO:0008270: zinc ion binding	-
ppe-miR395d	18774458	PRUPE_ppa001009mg	NCBI_Assembly:GCF_000346465.:	NW_006760208.1	-	6402794	6408935	6141	-	-	GO:0005215:transporter activity;GO:0005524:ATP binding;GO:0016887:ATPase activity	GO:0016021:integral component of membrane
ppe-miR395d	18774493	PRUPE_ppa023974mg	NCBI_Assembly:GCF_000346465.:	NW_006760208.1	-	16028125	16032496	4371	-	-	-	-
ppe-miR395d	18774602	PRUPE_ppa017999mg	NCBI_Assembly:GCF_000346465.:	NW_006760208.1	-	13327555	13330212	2657	-	-	GO:0043531: ADP binding	-
ppe-miR395d	18774660	PRUPE_ppa025652mg	NCBI_Assembly:GCF_000346465.:	NW_006760208.1	-	22592625	22593497	872	-	-	-	-

ppe-miR395d	18774705	PRUPE_ppa023075mg	NCBI_Assembly:GCF_000346465.:	NW_006760208.1	+	24932917	24933419	502	-	-	GO:0009055: electron carrier activity	-
ppe-miR395d	18774744	PRUPE_ppa006015mg	NCBI_Assembly:GCF_000346465.:	NW_006760208.1	+	1703242	1705171	1929	-	-	GO:0016758:transferase activity, transferring hexosyl groups	-
ppe-miR395d	18774828	PRUPE_ppa022793mg	NCBI_Assembly:GCF_000346465.:	NW_006760208.1	-	2786738	2787258	520	-	-	-	-
ppe-miR395d	18774833	PRUPE_ppa019730mg	NCBI_Assembly:GCF_000346465.:	NW_006760208.1	-	26547337	26551592	4255	pper04626:Plant-pathogen interaction	GO:0006826:iron ion transport;GO:0009408:response to heat;GO:0009845:seed germination;GO:0010106:cellular response to iron ion starvation;GO:0010167:response to nitrate;GO:0015706:nitrate transport	GO:0004601: peroxidase activity;GO:0005509:calcium ion binding;GO:0016174:NAD(P)H oxidase activity	GO:0016021:integral component of membrane
ppe-miR395d	18774879	PRUPE_ppa003773mg	NCBI_Assembly:GCF_000346465.:	NW_006760208.1	+	8438554	8441609	3055	-	-	GO:0004674: protein serine/threonine kinase activity;GO:0005524:ATP binding	-
ppe-miR395d	18774882	PRUPE_ppa019085mg	NCBI_Assembly:GCF_000346465.:	NW_006760208.1	+	18963484	18967054	3570	-	-	GO:0043531: ADP binding	-
ppe-miR395d	18775018	PRUPE_ppa003731mg	NCBI_Assembly:GCF_000346465.:	NW_006760208.1	+	21803813	21807645	3832	-	-	-	-
ppe-miR395d	18775033	PRUPE_ppa010197mg	NCBI_Assembly:GCF_000346465.:	NW_006760208.1	-	20429509	20432189	2680	-	-	-	-

ppe-miR395d	18775244	PRUPE_ppa010719mg	NCBI_Assembly:GCF_000346465.:	NW_006760208.1	+	25111136	25114030	2894	-	GO:0009793:embryo development ending in seed dormancy;GO:0016226:iron-sulfur cluster assembly;GO:0043043:peptide biosynthetic process;GO:0045036:protein targeting to chloroplast	GO:0003746:translation elongation factor activity	GO:0009570:chloroplast stroma
ppe-miR395d	18775333	PRUPE_ppa014520mg	NCBI_Assembly:GCF_000346465.:	NW_006760208.1	-	24120752	24121319	567	-	-	-	-
ppe-miR395d	18775728	PRUPE_ppa004996mg	NCBI_Assembly:GCF_000346465.:	NW_006760212.1	+	11188218	11190931	2713	-	GO:0005975:carbohydrate metabolic process;GO:0071555:cell wall organization	GO:0004650:polygalacturonase activity	GO:0005576:extracellular region
ppe-miR395d	18775841	PRUPE_ppa021263mg	NCBI_Assembly:GCF_000346465.:	NW_006760212.1	+	7708826	7710769	1943	pper04120:Ubiquitin mediated proteolysis;pper04141:Protein processing in endoplasmic reticulum	GO:0006511:ubiquitin-dependent protein catabolic process	-	GO:0031461:cullin-RING ubiquitin ligase complex
ppe-miR395d	18775871	PRUPE_ppa020456mg	NCBI_Assembly:GCF_000346465.:	NW_006760212.1	-	16834633	16836753	2120	-	GO:0007067:mitotic nuclear division	-	GO:0000775:chromosome, centromeric region;GO:0005634:nucleus

ppe-miR395d	18775884	PRUPE_ppa019280mg	NCBI_Assembly:GCF_000346465.:	NW_006760212.1	+	3644150	3645239	1089	-	-	GO:0003677:DNA binding;GO:0003682:chromatin binding;GO:0003700:sequence-specific DNA binding transcription factor activity	-
ppe-miR395d	18775934	PRUPE_ppa023247mg	NCBI_Assembly:GCF_000346465.:	NW_006760212.1	-	11986550	11986915	365	-	GO:0006869:lipid transport	GO:0008289:lipid binding	-
ppe-miR395d	18775996	PRUPE_ppa003575mg	NCBI_Assembly:GCF_000346465.:	NW_006760212.1	-	13288009	13293379	5370	-	-	-	-
ppe-miR395d	18776147	PRUPE_ppa017571mg	NCBI_Assembly:GCF_000346465.:	NW_006760212.1	-	15274651	15276452	1801	-	-	GO:0003677:DNA binding;GO:0003682:chromatin binding	-
ppe-miR395d	18776155	PRUPE_ppa013028mg	NCBI_Assembly:GCF_000346465.:	NW_006760212.1	+	6505985	6508109	2124	-	-	-	-
ppe-miR395d	18776171	PRUPE_ppa001787mg	NCBI_Assembly:GCF_000346465.:	NW_006760212.1	+	12885277	12890862	5585	pper03013:RNA transport	-	-	-
ppe-miR395d	18776221	PRUPE_ppa002101mg	NCBI_Assembly:GCF_000346465.:	NW_006760212.1	-	16418437	16422940	4503	-	-	GO:0004601:peroxidase activity;GO:0005509:calcium ion binding;GO:0050664:oxidoreductase activity, acting on NAD(P)H, oxygen as acceptor	GO:0016021:integral component of membrane
ppe-miR395d	18776337	PRUPE_ppa015583mg	NCBI_Assembly:GCF_000346465.:	NW_006760212.1	-	17115300	17117309	2009	-	-	-	-
ppe-miR395d	18776382	PRUPE_ppa007507mg	NCBI_Assembly:GCF_000346465.:	NW_006760212.1	+	11257461	11260583	3122	-	-	GO:0003676:nucleic acid binding	-
ppe-miR395d	18776478	PRUPE_ppa012437mg	NCBI_Assembly:GCF_000346465.:	NW_006760212.1	+	3469223	3470205	982	-	-	-	-

ppe-miR395d	18776559	PRUPE_ppa016601mg	NCBI_Assembly:GCF_000346465.:	NW_006760212.1	+	5002067	5005113	3046	-	-	GO:0004497: monooxygenase activity;GO:0005506:iron ion binding;GO:0016705:oxidoreductase activity, acting on paired donors, with incorporation or reduction of molecular oxygen;GO:0020037:heme binding	-
ppe-miR395d	18776649	PRUPE_ppa005059mg	NCBI_Assembly:GCF_000346465.:	NW_006760212.1	+	1402343	1405240	2897	pper00906:Carotenoid biosynthesis	-	GO:0004497: monooxygenase activity;GO:0005506:iron ion binding;GO:0016705:oxidoreductase activity, acting on paired donors, with incorporation or reduction of molecular oxygen;GO:0020037:heme binding	-
ppe-miR395d	18776673	PRUPE_ppa006955mg	NCBI_Assembly:GCF_000346465.:	NW_006760212.1	+	5849713	5854095	4382	-	GO:0006457: protein folding;GO:0006979:response to oxidative stress	GO:0003755: peptidyl-prolyl cis-trans isomerase activity	-

ppe-miR395d	18776736	PRUPE_ppa013288mg	NCBI_Assembly:GCF_000346465.:	NW_006760212.1	-	11950685	11951535	850	-	-	-	-
ppe-miR395d	18776798	PRUPE_ppa004611mg	NCBI_Assembly:GCF_000346465.:	NW_006760212.1	+	11826137	11828782	2645	-	-	-	-
ppe-miR395d	18776851	PRUPE_ppa006018mg	NCBI_Assembly:GCF_000346465.:	NW_006760212.1	-	13524131	13525691	1560	pper00073:Cutin, suberine and wax biosynthesis	-	GO:0016747:transferase activity, transferring acyl groups other than amino-acyl groups	-
ppe-miR395d	18777099	PRUPE_ppa003155mg	NCBI_Assembly:GCF_000346465.:	NW_006760212.1	-	15383687	15386393	2706	-	-	GO:0005215:transporter activity	GO:0016020:membrane
ppe-miR395d	18777147	PRUPE_ppa005023mg	NCBI_Assembly:GCF_000346465.:	NW_006760212.1	+	11821973	11825409	3436	-	-	-	-
ppe-miR395d	18777190	PRUPE_ppa015124mg	NCBI_Assembly:GCF_000346465.:	NW_006760212.1	-	882246	884401	2155	-	-	-	-
ppe-miR395d	18777324	PRUPE_ppa018016mg	NCBI_Assembly:GCF_000346465.:	NW_006760212.1	-	9681214	9681612	398	-	GO:0006662:glycerol ether metabolic process;GO:0045454:cell redox homeostasis	GO:0015035:protein disulfide oxidoreductase activity	GO:0005623:cell
ppe-miR395d	18777331	PRUPE_ppa008400mg	NCBI_Assembly:GCF_000346465.:	NW_006760212.1	-	7773985	7777822	3837	-	GO:0043407:negative regulation of MAP kinase activity	GO:0004725:protein tyrosine phosphatase activity;GO:0008138:protein tyrosine/serine/threonine phosphatase activity	-

ppe-miR395d	18777444	PRUPE_ppa009898mg	NCBI_Assembly:GCF_000346465.:	NW_006760212.1	-	2655889	2657874	1985	-	-	GO:0003677: DNA binding;GO:0003682:chromatin binding;GO:0003700:sequence-specific DNA binding transcription factor activity	-
ppe-miR395d	18777496	PRUPE_ppa009609mg	NCBI_Assembly:GCF_000346465.:	NW_006760212.1	-	15671370	15674389	3019	-	-	-	-
ppe-miR395d	18777712	PRUPE_ppa006936mg	NCBI_Assembly:GCF_000346465.:	NW_006760212.1	+	5321105	5328026	6921	-	GO:0016571: histone methylation;GO:0048451:petal formation;GO:0048453:sepal formation	-	-
ppe-miR395d	18777880	PRUPE_ppa002015mg	NCBI_Assembly:GCF_000346465.:	NW_006760212.1	-	12506374	12512286	5912	-	-	GO:0004672: protein kinase activity;GO:0005524:ATP binding	-
ppe-miR395d	18777991	PRUPE_ppa011001mg	NCBI_Assembly:GCF_000346465.:	NW_006760212.1	+	6736793	6742010	5217	-	-	-	-
ppe-miR395d	18778057	PRUPE_ppa011954mg	NCBI_Assembly:GCF_000346465.:	NW_006760212.1	+	17622024	17623067	1043	-	-	GO:0003677: DNA binding;GO:0003682:chromatin binding	-
ppe-miR395d	18778201	PRUPE_ppa013681mg	NCBI_Assembly:GCF_000346465.:	NW_006760220.1	-	1769292	1769886	594	-	GO:0007017: microtubule-based process	-	GO:0005875:microtubule associated complex

ppe-miR395d	18778250	PRUPE_ppa010162mg	NCBI_Assembly:GCF_000346465.:	NW_006760220.1	+	2902954	2906436	3482	-	GO:0006096: glycolytic process;GO:0006833:water transport;GO:0006972:hyperosmotic response;GO:0007030:Golgi organization; GO:0009266:response to temperature stimulus;GO:0009651:response to salt stress;GO:0009750:response to fructose;GO:0019288:isopentenyl diphosphate biosynthetic process, methylerythritol 4-phosphate pathway;GO:0019344:cysteine biosynthetic process;GO:0032880:regulation of protein localization;GO:0042744:hydrogen [illegible]	GO:0005509: calcium ion binding	GO:0005634:nucleus;GO:0005829:cytosol;GO:0005886:plasma membrane
ppe-miR395d	18778305	PRUPE_ppa016168mg	NCBI_Assembly:GCF_000346465.:	NW_006760220.1	+	4305742	4309784	4042	-	-	-	-
ppe-miR395d	18778324	PRUPE_ppa007058mg	NCBI_Assembly:GCF_000346465.:	NW_006760220.1	-	8482652	8485600	2948	-	-	-	-

ppe-miR395d	18778417	PRUPE_ppa005278mg	NCBI_Assembly:GCF_000346465.:	NW_006760220.1	+	13875390	13884446	9056	pper04122:Sulfur relay system	GO:0002143:tRNA wobble position uridine thiolation;GO:0006777:Mo-molybdopterin cofactor biosynthetic process;GO:0018192:enzyme active site formation via L-cysteine persulfide	GO:0004792:thiosulfate sulfurtransferase activity;GO:0005524:ATP binding;GO:0008265:Mo-molybdopterin cofactor sulfurase activity;GO:0008641:small protein activating enzyme activity;GO:0016779:nucleotidyltransferase activity;GO:0046872:metal ion binding	GO:0005829:cytosol
ppe-miR395d	18778429	PRUPE_ppa005033mg	NCBI_Assembly:GCF_000346465.:	NW_006760220.1	+	8462021	8467187	5166	pper01100:Metabolic pathways;pper00510:N-Glycan biosynthesis	GO:0016126:sterol biosynthetic process;GO:0030244:cellulose biosynthetic process;GO:0046520:sphingoid biosynthetic process;GO:0048193:Golgi vesicle transport	GO:0016757:transferase activity, transferring glycosyl groups	GO:0005622:intracellular
ppe-miR395d	18778640	PRUPE_ppa022486mg	NCBI_Assembly:GCF_000346465.:	NW_006760220.1	-	2187892	2189560	1668	-	-	-	-
ppe-miR395d	18778687	PRUPE_ppa022500mg	NCBI_Assembly:GCF_000346465.:	NW_006760220.1	-	13111873	13112571	698	-	-	-	-
ppe-miR395d	18778738	PRUPE_ppa016336mg	NCBI_Assembly:GCF_000346465.:	NW_006760220.1	-	10488889	10489611	722	-	-	-	-

ppe-miR395d	18778944	PRUPE_ppa000567mg	NCBI_Assembly:GCF_000346465.:	NW_006760220.1	+	29673435	29680787	7352	-	GO:0030244:cellulose biosynthetic process;GO:0071555:cell wall organization	GO:0008270:zinc ion binding;GO:0016760:cellulose synthase (UDP-forming) activity	GO:0005886:plasma membrane;GO:0016021:integral component of membrane
ppe-miR395d	18779075	PRUPE_ppa014055mg	NCBI_Assembly:GCF_000346465.:	NW_006760220.1	-	11513534	11514029	495	-	-	-	-
ppe-miR395d	18779097	PRUPE_ppa019045mg	NCBI_Assembly:GCF_000346465.:	NW_006760220.1	+	8379912	8380797	885	pper00480:Glutathione metabolism	-	-	-
ppe-miR395d	18779219	PRUPE_ppa002371mg	NCBI_Assembly:GCF_000346465.:	NW_006760220.1	-	15937740	15940017	2277	-	-	-	-
ppe-miR395d	18779332	PRUPE_ppa016555mg	NCBI_Assembly:GCF_000346465.:	NW_006760220.1	+	10384024	10385982	1958	-	-	GO:0004674:protein serine/threonine kinase activity;GO:0005524:ATP binding;GO:0030246:carbohydrate binding	-
ppe-miR395d	18779383	PRUPE_ppa001166mg	NCBI_Assembly:GCF_000346465.:	NW_006760220.1	+	6194205	6198347	4142	-	-	GO:0008270:zinc ion binding	-
ppe-miR395d	18779419	PRUPE_ppa004121mg	NCBI_Assembly:GCF_000346465.:	NW_006760220.1	-	2132531	2134568	2037	-	-	-	-
ppe-miR395d	18779489	PRUPE_ppa025550mg	NCBI_Assembly:GCF_000346465.:	NW_006760220.1	-	9223298	9224657	1359	-	-	-	-

ppe-miR395d	18779569	PRUPE_ppa009670mg	NCBI_Assembly:GCF_000346465.:	NW_006760220.1	+	13283745	13286914	3169	-	GO:0009965:leaf morphogenesis;GO:0010027:thylakoid membrane organization;GO:0015979:photosynthesis;GO:0030154:cell differentiation	GO:0005509:calcium ion binding	GO:0009534:chloroplast thylakoid;GO:0009570:chloroplast stroma;GO:0009654:photosystem II oxygen evolving complex;GO:0019898:extrinsic component of membrane;GO:0031977:thylakoid lumen
ppe-miR395d	18779678	PRUPE_ppa023453mg	NCBI_Assembly:GCF_000346465.:	NW_006760220.1	-	28354121	28355432	1311	-	-	GO:0004672:protein kinase activity;GO:0005524:ATP binding	-
ppe-miR395d	18779739	PRUPE_ppa021722mg	NCBI_Assembly:GCF_000346465.:	NW_006760220.1	-	4705853	4710386	4533	-	-	GO:0004672:protein kinase activity;GO:0005509:calcium ion binding;GO:0005524:ATP binding;GO:0030247:polysaccharide binding	-
ppe-miR395d	18779787	PRUPE_ppa014555mg	NCBI_Assembly:GCF_000346465.:	NW_006760220.1	+	8375736	8376625	889	pper00480:Glutathione metabolism	-	-	-
ppe-miR395d	18779841	PRUPE_ppa024549mg	NCBI_Assembly:GCF_000346465.:	NW_006760220.1	-	131827	132477	650	-	-	GO:0004857:enzyme inhibitor activity;GO:0030599:pectinesterase activity	-
ppe-miR395d	18779843	PRUPE_ppa017340mg	NCBI_Assembly:GCF_000346465.:	NW_006760220.1	-	13589425	13589709	284	-	GO:0006869:lipid transport	GO:0008289:lipid binding	-

ppe-miR395d	18780148	PRUPE_ppa026458mg	NCBI_Assembly:GCF_000346465.:	NW_006760220.1	-	10591493	10594680	3187	-	-	GO:0015238: drug transmembrane transporter activity;GO:0015297:antiporter activity	GO:0016020:membrane
ppe-miR395d	18780303	PRUPE_ppa023993mg	NCBI_Assembly:GCF_000346465.:	NW_006760220.1	-	17800797	17802832	2035	-	-	GO:0004674: protein serine/threonine kinase activity;GO:0005524:ATP binding;GO:0030246:carbohydrate binding	-
ppe-miR395d	18780894	PRUPE_ppa015941mg	NCBI_Assembly:GCF_000346465.:	NW_006760220.1	+	10347778	10350422	2644	-	-	GO:0004672: protein kinase activity;GO:0005524:ATP binding;GO:0030246:carbohydrate binding	-
ppe-miR395d	18781233	PRUPE_ppa009038mg	NCBI_Assembly:GCF_000346465.:	NW_006760220.1	-	9508052	9509218	1166	-	GO:0005975: carbohydrate metabolic process	GO:0004553: hydrolase activity, hydrolyzing O-glycosyl compounds	-
ppe-miR395d	18781525	PRUPE_ppa019606mg	NCBI_Assembly:GCF_000346465.:	NW_006760220.1	+	7367813	7369080	1267	-	-	-	-
ppe-miR395d	18781581	PRUPE_ppa012119mg	NCBI_Assembly:GCF_000346465.:	NW_006760268.1	-	8681723	8684270	2547	pper03010:Ribosome	GO:0006412:translation	GO:0003735: structural constituent of ribosome	GO:0005840:ribosome
ppe-miR395d	18781663	PRUPE_ppa023239mg	NCBI_Assembly:GCF_000346465.:	NW_006760268.1	+	10494335	10497401	3066	-	-	GO:0004672: protein kinase activity;GO:0005524:ATP binding	GO:0016021:integral component of membrane
ppe-miR395d	18781694	PRUPE_ppa020648mg	NCBI_Assembly:GCF_000346465.:	NW_006760268.1	-	19633174	19633872	698	-	-	-	-

ppe-miR395d	18782884	PRUPE_ppa007979mg	NCBI_Assembly:GCF_000346465.:	NW_006760268.1	+	14103636	14105181	1545	-	-	GO:0016788: hydrolase activity, acting on ester bonds	-
ppe-miR395d	18782887	PRUPE_ppa000084mg	NCBI_Assembly:GCF_000346465.:	NW_006760268.1	-	10735617	10755622	20005	pper03013:RNA transport;pper03040:Spliceosome	-	GO:0000155: phosphorelay sensor kinase activity	GO:0005622:intracellular;GO:0016020:membrane
ppe-miR395d	18782922	PRUPE_ppb021823mg	NCBI_Assembly:GCF_000346465.:	NW_006760268.1	+	8417859	8418059	200	-	GO:0006351:transcription, DNA-templated;GO:0006355:regulation of transcription, DNA-templated	GO:0003677: DNA binding	GO:0005634:nucleus
ppe-miR395d	18783049	PRUPE_ppa023191mg	NCBI_Assembly:GCF_000346465.:	NW_006760268.1	-	15141364	15141746	382	-	-	-	-
ppe-miR395d	18783136	PRUPE_ppa027005mg	NCBI_Assembly:GCF_000346465.:	NW_006760268.1	+	10916107	10917864	1757	-	-	-	GO:0016021:integral component of membrane
ppe-miR395d	18783257	PRUPE_ppa019394mg	NCBI_Assembly:GCF_000346465.:	NW_006760268.1	-	518061	518282	221	-	-	-	-
ppe-miR395d	18783352	PRUPE_ppa010252mg	NCBI_Assembly:GCF_000346465.:	NW_006760268.1	-	2149391	2150644	1253	-	-	GO:0003677: DNA binding;GO:0003682:chromatin binding	-
ppe-miR395d	18783546	PRUPE_ppa013605mg	NCBI_Assembly:GCF_000346465.:	NW_006760268.1	+	18357758	18358496	738	-	-	-	-
ppe-miR395d	18783754	PRUPE_ppa009608mg	NCBI_Assembly:GCF_000346465.:	NW_006760268.1	-	13672078	13673293	1215	-	GO:0006073: cellular glucan metabolic process;GO:0071555:cell wall organization	GO:0004553: hydrolase activity, hydrolyzing O-glycosyl compounds;GO:0016762:xyloglucan:xyloglucosyl transferase activity	GO:0005618:cell wall;GO:0048046:apoplast

ppe-miR395d	18783829	PRUPE_ppa018520mg	NCBI_Assembly:GCF_000346465.:	NW_006760268.1	-	1732527	1733536	1009	-	GO:0006633:fatty acid biosynthetic process	-	-
ppe-miR395d	18783831	PRUPE_ppa002420mg	NCBI_Assembly:GCF_000346465.:	NW_006760268.1	+	20838318	20841102	2784	-	-	GO:0004672: protein kinase activity;GO:0005524:ATP binding	-
ppe-miR395d	18783864	PRUPE_ppa021381mg	NCBI_Assembly:GCF_000346465.:	NW_006760268.1	-	7303609	7303890	281	-	-	GO:0003676: nucleic acid binding	-
ppe-miR395d	18783889	PRUPE_ppa013551mg	NCBI_Assembly:GCF_000346465.:	NW_006760268.1	+	19199756	19202490	2734	-	-	-	-
ppe-miR395d	18784101	PRUPE_ppa025523mg	NCBI_Assembly:GCF_000346465.:	NW_006760268.1	-	9057162	9057467	305	-	-	-	-
ppe-miR395d	18784364	PRUPE_ppa007883mg	NCBI_Assembly:GCF_000346465.:	NW_006760268.1	-	19548346	19549836	1490	-	GO:0006351:transcription, DNA-templated;GO:0006355:regulation of transcription, DNA-templated	GO:0003677: DNA binding	GO:0005634:nucleus
ppe-miR395d	18784376	PRUPE_ppa026806mg	NCBI_Assembly:GCF_000346465.:	NW_006760268.1	+	20610313	20612423	2110	-	-	-	-
ppe-miR395d	18784552	PRUPE_ppa022509mg	NCBI_Assembly:GCF_000346465.:	NW_006760324.1	+	4978029	4980145	2116	-	-	-	-
ppe-miR395d	18784729	PRUPE_ppa026051mg	NCBI_Assembly:GCF_000346465.:	NW_006760324.1	-	19281205	19282339	1134	-	-	-	-
ppe-miR395d	18784739	PRUPE_ppa018480mg	NCBI_Assembly:GCF_000346465.:	NW_006760324.1	+	9570543	9571153	610	-	-	-	-
ppe-miR395d	18784747	PRUPE_ppa000247mg	NCBI_Assembly:GCF_000346465.:	NW_006760324.1	-	439117	443666	4549	-	-	GO:0043531: ADP binding	-
ppe-miR395d	18784803	PRUPE_ppa023853mg	NCBI_Assembly:GCF_000346465.:	NW_006760324.1	-	19069154	19070052	898	-	-	GO:0003677: DNA binding;GO:0003682:chromatin binding	-
ppe-miR395d	18784853	PRUPE_ppa008042mg	NCBI_Assembly:GCF_000346465.:	NW_006760324.1	-	25669715	25673674	3959	-	GO:0006470: protein dephosphorylation;GO:0048767:root hair elongation	GO:0004722: protein serine/threonine phosphatase activity;GO:0046872:metal ion binding	-

ppe-miR395d	18784886	PRUPE_ppa011359mg	NCBI_Assembly:GCF_000346465.:	NW_006760324.1	-	21998093	21999918	1825	-	-	GO:0003676: nucleic acid binding;GO:0008270:zinc ion binding	-
ppe-miR395d	18785001	PRUPE_ppa024230mg	NCBI_Assembly:GCF_000346465.:	NW_006760324.1	+	14525962	14526949	987	-	-	-	-
ppe-miR395d	18785011	PRUPE_ppa013167mg	NCBI_Assembly:GCF_000346465.:	NW_006760324.1	-	24951504	24952210	706	-	-	-	-
ppe-miR395d	18785058	PRUPE_ppa000113mg	NCBI_Assembly:GCF_000346465.:	NW_006760324.1	+	25747437	25755998	8561	-	-	GO:0003677: DNA binding;GO:0008270:zinc ion binding	-
ppe-miR395d	18785081	PRUPE_ppa010386mg	NCBI_Assembly:GCF_000346465.:	NW_006760324.1	-	16158063	16160063	2000	-	-	-	-
ppe-miR395d	18785086	PRUPE_ppa001496mg	NCBI_Assembly:GCF_000346465.:	NW_006760324.1	-	25388663	25391454	2791	-	-	-	-
ppe-miR395d	18785127	PRUPE_ppa003890mg	NCBI_Assembly:GCF_000346465.:	NW_006760324.1	+	22929782	22932643	2861	pper01100:Metabolic pathways;pper00562:Inositol phosphate metabolism;pper04070:Phosphatidylinositol signaling system	GO:0016042:lipid catabolic process;GO:0035556:intracellular signal transduction	GO:0004435: phosphatidylinositol phospholipase C activity;GO:0004871:signal transducer activity	GO:0005622:intracellular
ppe-miR395d	18785188	PRUPE_ppa011410mg	NCBI_Assembly:GCF_000346465.:	NW_006760324.1	-	8273826	8280710	6884	-	-	-	GO:0005829:cytosol
ppe-miR395d	18785226	PRUPE_ppa003202mg	NCBI_Assembly:GCF_000346465.:	NW_006760324.1	+	22028333	22032770	4437	pper00970:Aminoacyl-tRNA biosynthesis;pper00450:Selenocompound metabolism	GO:0006431: methionyl-tRNA aminoacylation;GO:0048481:ovule development	GO:0004825: methionine-tRNA ligase activity;GO:0005524:ATP binding	GO:0005739:mitochondrion;GO:0009570:chloroplast stroma
ppe-miR395d	18785377	PRUPE_ppa009435mg	NCBI_Assembly:GCF_000346465.:	NW_006760324.1	-	25422966	25425021	2055	-	-	-	-

ppe-miR395d	18785423	PRUPE_ppa025412mg	NCBI_Assembly:GCF_000346465.:	NW_006760324.1	-	20658793	20659218	425	pper04120:Ubiquitin mediated proteolysis;pper04141:Protein processing in endoplasmic reticulum	GO:0006511: ubiquitin-dependent protein catabolic process	-	-
ppe-miR395d	18785468	PRUPE_ppa010803mg	NCBI_Assembly:GCF_000346465.:	NW_006760324.1	+	17396793	17399861	3068	-	GO:0016559: peroxisome fission	-	GO:0005779:integral component of peroxisomal membrane
ppe-miR395d	18785492	PRUPE_ppa003289mg	NCBI_Assembly:GCF_000346465.:	NW_006760324.1	-	23198695	23202687	3992	-	-	GO:0008168: methyltransferase activity	-
ppe-miR395d	18785605	PRUPE_ppa016627mg	NCBI_Assembly:GCF_000346465.:	NW_006760324.1	-	18641896	18644638	2742	-	-	GO:0008270: zinc ion binding	-
ppe-miR395d	18785677	PRUPE_ppa018600mg	NCBI_Assembly:GCF_000346465.:	NW_006760324.1	+	18099703	18101691	1988	-	-	-	-

ppe-miR395d	18785682	PRUPE_ppa011130mg	NCBI_Assembly:GCF_000346465.:	NW_006760324.1	-	13708533	13709230	697	pper03010:Ribosome	GO:0006364:rRNA processing;GO:0006412:translation;GO:0010207:photosystem II assembly;GO:0015995:chlorophyll biosynthetic process;GO:0019288:isopentenyl diphosphate biosynthetic process, methylerythritol 4-phosphate pathway;GO:0045036:protein targeting to chloroplast	GO:0003735:structural constituent of ribosome	GO:0005840:ribosome;GO:0009570:chloroplast stroma;GO:0009941:chloroplast envelope;GO:0016020:membrane
ppe-miR395d	18785745	PRUPE_ppa015741mg	NCBI_Assembly:GCF_000346465.:	NW_006760324.1	+	2490159	2490425	266	-	-	-	-
ppe-miR395d	18785790	PRUPE_ppa010737mg	NCBI_Assembly:GCF_000346465.:	NW_006760324.1	-	8312798	8317814	5016	-	GO:0006623:protein targeting to vacuole;GO:0007033:vacuole organization;GO:0016192:vesicle-mediated transport	-	GO:0005768:endosome;GO:0016021:integral component of membrane;GO:0031201:SNARE complex
ppe-miR395d	18785838	PRUPE_ppa013726mg	NCBI_Assembly:GCF_000346465.:	NW_006760324.1	-	26004055	26005205	1150	-	-	-	-
ppe-miR395d	18785944	PRUPE_ppa022813mg	NCBI_Assembly:GCF_000346465.:	NW_006760324.1	-	1802711	1804831	2120	-	-	GO:0004252:serine-type endopeptidase activity	-

ppe-miR395d	18786011	PRUPE_ppa021814mg	NCBI_Assembly:GCF_000346465.:	NW_006760324.1	+	13697799	13702325	4526	pper00360:Phenylalanine metabolism;pper01100:Metabolic pathways;pper01110:Biosynthesis of secondary metabolites;pper00940:Phenylpropanoid biosynthesis;pper00941:Flavonoid biosynthesis;pper00945:Stilbenoid, diarylheptanoid and gingerol biosynthesis	-	GO:0008171: O-methyltransferase activity	-
ppe-miR395d	18786098	PRUPE_ppa015805mg	NCBI_Assembly:GCF_000346465.:	NW_006760324.1	+	12823566	12823991	425	pper04120:Ubiquitin mediated proteolysis;pper04141:Protein processing in endoplasmic reticulum	GO:0006511: ubiquitin-dependent protein catabolic process	-	-
ppe-miR395d	18786124	PRUPE_ppa016360mg	NCBI_Assembly:GCF_000346465.:	NW_006760324.1	+	4017891	4018541	650	-	-	GO:0003676: nucleic acid binding	-
ppe-miR395d	18786192	PRUPE_ppa011500mg	NCBI_Assembly:GCF_000346465.:	NW_006760324.1	+	9657770	9658575	805	-	-	-	GO:0005739:mitochondrion
ppe-miR395d	18786197	PRUPE_ppa015461mg	NCBI_Assembly:GCF_000346465.:	NW_006760324.1	+	556851	561032	4181	-	-	GO:0043531: ADP binding	-
ppe-miR395d	18786304	PRUPE_ppa000274mg	NCBI_Assembly:GCF_000346465.:	NW_006760324.1	-	472157	477921	5764	-	-	GO:0043531: ADP binding	-

ppe-miR395d	18786544	PRUPE_ppa021740mg	NCBI_Assembly:GCF_000346465.:	NW_006760324.1	-	20290490	20292256	1766	-	GO:0006351:transcription, DNA-templated;GO:0006355:regulation of transcription, DNA-templated	GO:0003677: DNA binding	GO:0005634:nucleus
ppe-miR395d	18786695	PRUPE_ppa006786mg	NCBI_Assembly:GCF_000346465.:	NW_006760324.1	-	20357554	20360409	2855	-	-	GO:0016787: hydrolase activity	-
ppe-miR395d	18786713	PRUPE_ppa008765mg	NCBI_Assembly:GCF_000346465.:	NW_006760324.1	+	23842589	23845231	2642	-	-	GO:0004784: superoxide dismutase activity;GO:0005507:copper ion binding;GO:0046872:metal ion binding	GO:0005739:mitochondrion;GO:0005886:plasma membrane;GO:0009579:thylakoid;GO:0009941:chloroplast envelope
ppe-miR395d	18786729	PRUPE_ppa025228mg	NCBI_Assembly:GCF_000346465.:	NW_006760324.1	-	18021496	18024036	2540	-	-	-	-
ppe-miR395d	18786760	PRUPE_ppa004425mg	NCBI_Assembly:GCF_000346465.:	NW_006760324.1	-	25844232	25849372	5140	pper03040:Spliceosome	-	GO:0003676: nucleic acid binding;GO:0008270:zinc ion binding	GO:0005634:nucleus
ppe-miR395d	18787168	PRUPE_ppa005723mg	NCBI_Assembly:GCF_000346465.:	NW_006760324.1	+	10152771	10157648	4877	-	-	-	-
ppe-miR395d	18787216	PRUPE_ppa016239mg	NCBI_Assembly:GCF_000346465.:	NW_006760324.1	+	8139069	8140763	1694	pper01100:Metabolic pathways;pper00240:Pyrimidine metabolism	GO:0006221: pyrimidine nucleotide biosynthetic process	GO:0003883: CTP synthase activity	-

ppe-miR395d	18787253	PRUPE_ppa009184mg	NCBI_Assembly:GCF_000346465.:	NW_006760324.1	-	22184393	22186450	2057	-	GO:0002679:respiratory burst involved in defense response;GO:0009611:response to wounding;GO:0009612:response to mechanical stimulus;GO:0009693:ethylene biosynthetic process;GO:0010200:response to chitin;GO:0030968:endoplasmic reticulum unfolded protein response;GO:0035556:intracellular signal transduction; GO:0052542: defense response by callose deposition	-	GO:0005622:intracellular

ppe-miR395d	18787543	PRUPE_ppa007731mg	NCBI_Assembly:GCF_000346465.:	NW_006760324.1	+	26347312	26349788	2476	-	GO:0006338:chromatin remodeling;GO:0006355:regulation of transcription, DNA-templated;GO:0008284:positive regulation of cell proliferation;GO:0009909:regulation of flower development;GO:0042742:defense response to bacterium	-	GO:0005634:nucleus
ppe-miR395d	18787619	PRUPE_ppa026568mg	NCBI_Assembly:GCF_000346465.:	NW_006760324.1	-	613905	614144	239	-	-	-	-

ppe-miR395d	18787656	PRUPE_ppa025392mg	NCBI_Assembly:GCF_000346465.:	NW_006760324.1	-	26307434	26309277	1843	-	GO:0000278:mitotic cell cycle;GO:0003002:regionalization;GO:0006325:chromatin organization;GO:0007062:sister chromatid cohesion;GO:0007131:reciprocal meiotic recombination;GO:0007155:cell adhesion;GO:0008284:positive regulation of cell proliferation;GO:0009410:response to xenobiotic stimulus;GO:0009913:epidermal cell differentiation;GO:0010090:trichome morphogenesis;GO:0030307:positive regulation of cell growth;GO:0033044:regulation of	GO:0003690:double-stranded DNA binding	GO:0009330:DNA topoisomerase complex (ATP-hydrolyzing)

ppe-miR395d	18788206	PRUPE_ppa001184mg	NCBI_Assembly:GCF_000346465.:	NW_006760385.1	-	45498552	45501691	3139	-	GO:0002237:response to molecule of bacterial origin;GO:0006612:protein targeting to membrane;GO:0007165:signal transduction;GO:0009963:positive regulation of flavonoid biosynthetic process;GO:0010051:xylem and phloem pattern formation;GO:0010103:stomatal complex morphogenesis;GO:0010363:regulation of plant-type hypersensitive response;GO:0048443:stamen development	GO:0004674:protein serine/threonine kinase activity;GO:0005524:ATP binding	GO:0005622:intracellular
ppe-miR395d	18788452	PRUPE_ppa001811mg	NCBI_Assembly:GCF_000346465.:	NW_006760385.1	-	39741082	39745109	4027	-	-	GO:0004674:protein serine/threonine kinase activity;GO:0005524:ATP binding	-
ppe-miR395d	18788497	PRUPE_ppa010741mg	NCBI_Assembly:GCF_000346465.:	NW_006760385.1	+	29926223	29927821	1598	-	-	-	-
ppe-miR395d	18788617	PRUPE_ppa004032mg	NCBI_Assembly:GCF_000346465.:	NW_006760385.1	+	20252596	20258405	5809	-	-	-	-
ppe-miR395d	18788901	PRUPE_ppa013963mg	NCBI_Assembly:GCF_000346465.:	NW_006760385.1	-	35670562	35672619	2057	-	-	-	-

ppe-miR395d	18788922	PRUPE_ppa021372mg	NCBI_Assembly:GCF_000346465.:	NW_006760385.1	+	23717589	23720651	3062	-	-	-	-
ppe-miR395d	18788928	PRUPE_ppb022800mg	NCBI_Assembly:GCF_000346465.:	NW_006760385.1	+	46240281	46242449	2168	-	GO:0015074: DNA integration	GO:0003676: nucleic acid binding	GO:0005634:nucleus
ppe-miR395d	18789333	PRUPE_ppa009225mg	NCBI_Assembly:GCF_000346465.:	NW_006760385.1	+	34637405	34638801	1396	-	-	-	-
ppe-miR395d	18789337	PRUPE_ppa026534mg	NCBI_Assembly:GCF_000346465.:	NW_006760385.1	-	23227246	23228133	887	-	-	-	-
ppe-miR395d	18789348	PRUPE_ppa021895mg	NCBI_Assembly:GCF_000346465.:	NW_006760385.1	+	29836077	29837105	1028	-	-	-	-
ppe-miR395d	18789368	PRUPE_ppa008274mg	NCBI_Assembly:GCF_000346465.:	NW_006760385.1	-	35320464	35321936	1472	pper03015:mRNA surveillance pathway	GO:0008283: cell proliferation	GO:0000166: nucleotide binding;GO:0003727:single-stranded RNA binding	-
ppe-miR395d	18789561	PRUPE_ppa002152mg	NCBI_Assembly:GCF_000346465.:	NW_006760385.1	-	33653546	33657734	4188	-	-	GO:0004672: protein kinase activity;GO:0005524:ATP binding	-
ppe-miR395d	18789581	PRUPE_ppa023705mg	NCBI_Assembly:GCF_000346465.:	NW_006760385.1	-	12836048	12838881	2833	-	-	GO:0003676: nucleic acid binding;GO:0008408:3'-5' exonuclease activity	-
ppe-miR395d	18789594	PRUPE_ppa024271mg	NCBI_Assembly:GCF_000346465.:	NW_006760385.1	-	28245756	28247186	1430	-	-	GO:0016758:transferase activity, transferring hexosyl groups	-
ppe-miR395d	18789624	PRUPE_ppa016599mg	NCBI_Assembly:GCF_000346465.:	NW_006760385.1	+	42273170	42275476	2306	-	-	-	-
ppe-miR395d	18789967	PRUPE_ppa010227mg	NCBI_Assembly:GCF_000346465.:	NW_006760385.1	-	31644107	31647916	3809	-	GO:0055085:transmembrane transport	-	GO:0005741:mitochondrial outer membrane
ppe-miR395d	18790024	PRUPE_ppa009071mg	NCBI_Assembly:GCF_000346465.:	NW_006760385.1	+	38368466	38369389	923	-	-	-	-

ppe-miR395d	18790186	PRUPE_ppa000679mg	NCBI_Assembly:GCF_000346465.:	NW_006760385.1	-	22816990	22822324	5334	pper04075:Plant hormone signal transduction	GO:0000303:response to superoxide;GO:0006635:fatty acid beta-oxidation;GO:0006970:response to osmotic stress;GO:0007623:circadian rhythm;GO:0008219:cell death;GO:0009863:salicylic acid mediated signaling pathway;GO:0009873:ethylene-activated signaling pathway;GO:0009909:regulation of flower development;GO:0010029:regulation of seed germination;GO:0010087:phloem or xylem histogenesis;GO:0010150:leaf senescence;GO:0010271:regulation of chlorophyll	GO:0000155:phosphorelay sensor kinase activity;GO:0005034:osmosensor activity;GO:0009884:cytokinin receptor activity	GO:0005622:intracellular;GO:0005886:plasma membrane
ppe-miR395d	18790574	PRUPE_ppa015883mg	NCBI_Assembly:GCF_000346465.:	NW_006760385.1	-	34655413	34656502	1089	-	-	GO:0003677:DNA binding;GO:0003682:chromatin binding	-
ppe-miR395d	18790614	PRUPE_ppa026149mg	NCBI_Assembly:GCF_000346465.:	NW_006760385.1	-	781938	783156	1218	-	-	-	-

ppe-miR395d	18790645	PRUPE_ppa003065mg	NCBI_Assembly:GCF_000346465.:	NW_006760385.1	+	21693380	21695571	2191	pper04075:Plant hormone signal transduction	-	-	-
ppe-miR395d	18790656	PRUPE_ppa017915mg	NCBI_Assembly:GCF_000346465.:	NW_006760385.1	+	45967661	45970112	2451	-	-	GO:0004497:monooxygenase activity;GO:0005506:iron ion binding;GO:0016705:oxidoreductase activity, acting on paired donors, with incorporation or reduction of molecular oxygen;GO:0020037:heme binding	-
ppe-miR395d	18790818	PRUPE_ppa018370mg	NCBI_Assembly:GCF_000346465.:	NW_006760385.1	+	210547	212298	1751	-	-	-	-
ppe-miR395d	18790851	PRUPE_ppa013479mg	NCBI_Assembly:GCF_000346465.:	NW_006760385.1	+	8564114	8566744	2630	-	GO:0030244:cellulose biosynthetic process;GO:0048193:Golgi vesicle transport	-	GO:0072546:ER membrane protein complex
ppe-miR395d	18790894	PRUPE_ppa009591mg	NCBI_Assembly:GCF_000346465.:	NW_006760385.1	+	1013580	1016203	2623	pper01100:Metabolic pathways;pper01110:Biosynthesis of secondary metabolites;pper01230:Biosynthesis of amino acids;pper00340:Histidine metabolism	GO:0000105:histidine biosynthetic process	GO:0004424:imidazoleglycerol-phosphate dehydratase activity	-

ppe-miR395d	18790984	PRUPE_ppa002895mg	NCBI_Assembly:GCF_000346465.:	NW_006760385.1	+	25192376	25195005	2629	-	GO:0009627:systemic acquired resistance;GO:0031347:regulation of defense response	GO:0004674:protein serine/threonine kinase activity;GO:0005524:ATP binding	GO:0005886:plasma membrane
ppe-miR395d	18791020	PRUPE_ppa011087mg	NCBI_Assembly:GCF_000346465.:	NW_006760385.1	+	2842401	2843683	1282	pper00480:Glutathione metabolism	-	-	-
ppe-miR395d	18791026	PRUPE_ppa004044mg	NCBI_Assembly:GCF_000346465.:	NW_006760385.1	+	25854449	25858687	4238	-	GO:0055085:transmembrane transport	GO:0005215:transporter activity	GO:0016020:membrane
ppe-miR395d	18791150	PRUPE_ppa000982mg	NCBI_Assembly:GCF_000346465.:	NW_006760385.1	-	30749960	30753856	3896	-	-	GO:0004674:protein serine/threonine kinase activity;GO:0005524:ATP binding	GO:0016021:integral component of membrane
ppe-miR395d	18791220	PRUPE_ppa004917mg	NCBI_Assembly:GCF_000346465.:	NW_006760385.1	-	34796409	34798210	1801	-	-	-	-
ppe-miR395d	18791239	PRUPE_ppa004764mg	NCBI_Assembly:GCF_000346465.:	NW_006760385.1	+	2856939	2862040	5101	-	-	GO:0008270:zinc ion binding	-
ppe-miR395d	18791456	PRUPE_ppa000165mg	NCBI_Assembly:GCF_000346465.:	NW_006760385.1	-	34484342	34494286	9944	-	GO:0006396:RNA processing	GO:0003723:RNA binding;GO:0004525:ribonuclease III activity;GO:0005524:ATP binding	-
ppe-miR395d	18791492	PRUPE_ppa017671mg	NCBI_Assembly:GCF_000346465.:	NW_006760385.1	+	44542032	44545042	3010	-	-	-	-

ppe-miR395d	18791508	PRUPE_ppa021958mg	NCBI_Assembly:GCF_000346465.:	NW_006760385.1	+	37725820	37733439	7619	-	GO:0007062:sister chromatid cohesion;GO:0009640:photomorphogenesis;GO:0009845:seed germination;GO:0009880:embryonic pattern specification;GO:0009908:flower development;GO:0009909:regulation of flower development;GO:0010072:primary shoot apical meristem specification;GO:0010162:seed dormancy process;GO:0010182:sugar mediated signaling pathway;GO:0010228:vegetative to reproductive phase transition of meristem;GO:	-	GO:0080008:Cul4-RING E3 ubiquitin ligase complex
ppe-miR395d	18791568	PRUPE_ppa017473mg	NCBI_Assembly:GCF_000346465.:	NW_006760385.1	-	39283009	39284696	1687	-	-	-	-
ppe-miR395d	18791578	PRUPE_ppa011250mg	NCBI_Assembly:GCF_000346465.:	NW_006760385.1	+	41645943	41648408	2465	-	-	-	-
ppe-miR395d	18791650	PRUPE_ppa016412mg	NCBI_Assembly:GCF_000346465.:	NW_006760385.1	+	3056759	3057893	1134	-	-	-	-
ppe-miR395d	18791727	PRUPE_ppa010221mg	NCBI_Assembly:GCF_000346465.:	NW_006760385.1	+	35002426	35003750	1324	-	GO:0015996:chlorophyll catabolic process	-	-

ppe-miR395d	18791779	PRUPE_ppa020266mg	NCBI_Assembly:GCF_000346465.1	NW_006760385.1	-	27391378	27393500	2122	-	GO:0006457: protein folding	GO:0001106: RNA polymerase II transcription corepressor activity	GO:0016272:prefoldin complex
ppe-miR395d	18791890	PRUPE_ppb008326mg	NCBI_Assembly:GCF_000346465.1	NW_006760385.1	-	368596	369949	1353	-	-	-	-
ppe-miR395d	18791900	PRUPE_ppa006841mg	NCBI_Assembly:GCF_000346465.1	NW_006760385.1	-	8141239	8143517	2278	pper00270:Cysteine and methionine metabolism;pper01100:Metabolic pathways;pper01110:Biosynthesis of secondary metabolites;pper01230:Biosynthesis of amino acids	GO:0006556: S-adenosylmethionine biosynthetic process;GO:0006730:one-carbon metabolic process	GO:0004478: methionine adenosyltransferase activity;GO:0005524:ATP binding;GO:0046872:metal ion binding	-

ppe-miR395d	18791945	PRUPE_ppa011862mg	NCBI_Assembly:GCF_000346465.:	NW_006760385.1	-	27526854	27527908	1054	-	GO:0000023: maltose metabolic process;GO:0010103:stomatal complex morphogenesis;GO:0019252:starch biosynthetic process;GO:0019288:isopentenyl diphosphate biosynthetic process, methylerythritol 4-phosphate pathway;GO:0045893:positive regulation of transcription, DNA-templated	-	-
ppe-miR395d	18791971	PRUPE_ppa007249mg	NCBI_Assembly:GCF_000346465.:	NW_006760385.1	-	10528540	10530578	2038	-	-	GO:0004672: protein kinase activity;GO:0005524:ATP binding	-
ppe-miR395d	18792000	PRUPE_ppa012735mg	NCBI_Assembly:GCF_000346465.:	NW_006760385.1	-	2458167	2460388	2221	-	-	-	-
ppe-miR395d	18792055	PRUPE_ppa003585mg	NCBI_Assembly:GCF_000346465.:	NW_006760385.1	+	41165262	41168730	3468	-	GO:0006400:tRNA modification;GO:0006897:endocytosis	GO:0003924: GTPase activity;GO:0005525:GTP binding	GO:0005622:intracellular

ppe-miR395d	18792059	PRUPE_ppa006099mg	NCBI_Assembly:GCF_000346465.:	NW_006760385.1	-	24452651	24456565	3914	-	GO:0009825:multidimensional cell growth;GO:0010090:trichome morphogenesis;GO:0034314:Arp2/3 complex-mediated actin nucleation	GO:0005524:ATP binding	GO:0005829:cytosol;GO:0005885:Arp2/3 protein complex
ppe-miR395d	18792274	PRUPE_ppa010941mg	NCBI_Assembly:GCF_000346465.:	NW_006760385.1	+	1473042	1476175	3133	pper00270:Cysteine and methionine metabolism;pper01100:Metabolic pathways	GO:0019509:L-methionine biosynthetic process from methylthioadenosine	GO:0005506:iron ion binding;GO:0010309:acireductone dioxygenase [iron(II)-requiring] activity	GO:0005634:nucleus;GO:0005737:cytoplasm
ppe-miR395d	18792276	PRUPE_ppa014400mg	NCBI_Assembly:GCF_000346465.:	NW_006760385.1	-	12224759	12225723	964	-	-	-	-
ppe-miR395d	18792316	PRUPE_ppa015102mg	NCBI_Assembly:GCF_000346465.:	NW_006760385.1	+	25513844	25515951	2107	-	-	-	-
ppe-miR395d	18792928	PRUPE_ppa026794mg	NCBI_Assembly:GCF_000346465.:	NW_006760385.1	+	28762074	28762671	597	-	-	GO:0016787:hydrolase activity	-
ppe-miR395d	18792941	PRUPE_ppa022492mg	NCBI_Assembly:GCF_000346465.:	NW_006760385.1	-	46742027	46743934	1907	-	-	GO:0005215:transporter activity	GO:0016021:integral component of membrane
ppe-miR395d	18792954	PRUPE_ppa025631mg	NCBI_Assembly:GCF_000346465.:	NW_006760385.1	+	9291920	9293738	1818	pper01100:Metabolic pathways;pper00500:Starch and sucrose metabolism;pper00040:Pentose and glucuronate interconversions	GO:0042545:cell wall modification;GO:0045490:pectin catabolic process	GO:0004857:enzyme inhibitor activity;GO:0030599:pectinesterase activity;GO:0045330:aspartyl esterase activity	GO:0005618:cell wall

ppe-miR395d	18793102	PRUPE_ppa017542mg	NCBI_Assembly:GCF_000346465.:	NW_006760385.1	+	3427794	3428324	530	-	GO:0006351:transcription, DNA-templated;GO:0006355:regulation of transcription, DNA-templated	GO:0003677: DNA binding	GO:0005634:nucleus
ppe-miR395d	18793230	PRUPE_ppb018168mg	NCBI_Assembly:GCF_000346465.:	NW_006760385.1	+	35854553	35856821	2268	-	-	GO:0005506:iron ion binding;GO:0016705:oxidoreductase activity, acting on paired donors, with incorporation or reduction of molecular oxygen;GO:0020037:heme binding	-
ppe-miR395d	18793318	PRUPE_ppa021028mg	NCBI_Assembly:GCF_000346465.:	NW_006760385.1	+	37376898	37379922	3024	-	GO:0048527:lateral root development; GO:0080147:root hair cell development	GO:0005459: UDP-galactose transmembrane transporter activity;GO:0005460:UDP-glucose transmembrane transporter activity	GO:0005794:Golgi apparatus;GO:0016021:integral component of membrane
ppe-miR395d	18793325	PRUPE_ppa005844mg	NCBI_Assembly:GCF_000346465.:	NW_006760385.1	-	40349992	40356435	6443	-	-	-	-
ppe-miR395d	18793498	PRUPE_ppa002971mg	NCBI_Assembly:GCF_000346465.:	NW_006760385.1	-	27002959	27007696	4737	-	-	GO:0008168: methyltransferase activity	GO:0005768:endosome;GO:0005802:trans-Golgi network
ppe-miR395d	18793534	PRUPE_ppa015312mg	NCBI_Assembly:GCF_000346465.:	NW_006760385.1	-	41522175	41528153	5978	-	-	-	-
ppe-miR395d	18793620	PRUPE_ppa011867mg	NCBI_Assembly:GCF_000346465.:	NW_006760385.1	+	11047435	11048013	578	-	-	-	-

ppe-miR395d	18793896	PRUPE_ppa010822mg	NCBI_Assembly:GCF_000346465.:	NW_006760385.1	-	46344191	46351743	7552	-	GO:0006351:transcription, DNA-templated	GO:0003677:DNA binding;GO:0003700:sequence-specific DNA binding transcription factor activity	GO:0005634:nucleus
ppe-miR395d	18793970	PRUPE_ppa022249mg	NCBI_Assembly:GCF_000346465.:	NW_006760385.1	-	34321536	34322132	596	-	-	-	-
ppe-miR395d	18794040	PRUPE_ppa006856mg	NCBI_Assembly:GCF_000346465.:	NW_006760385.1	-	29731860	29734304	2444	-	GO:0051726:regulation of cell cycle	-	-
ppe-miR395e	18765973	PRUPE_ppa009429mg	NCBI_Assembly:GCF_000346465.:	NW_006760186.1	-	601147	607633	6486	-	-	GO:0008270:zinc ion binding	-
ppe-miR395e	18766004	PRUPE_ppa002790mg	NCBI_Assembly:GCF_000346465.:	NW_006760186.1	+	568707	573299	4592	-	GO:0007067:mitotic nuclear division	-	GO:0005819:spindle;GO:0005874:microtubule
ppe-miR395e	18766083	PRUPE_ppa012918mg	NCBI_Assembly:GCF_000346465.:	NW_006760186.1	-	1267515	1268921	1406	pper03030:DNA replication;pper03420:Nucleotide excision repair;pper03430:Mismatch repair;pper03440:Homologous recombination	-	-	-
ppe-miR395e	18766242	PRUPE_ppa008037mg	NCBI_Assembly:GCF_000346465.:	NW_006760194.1	+	4336639	4340550	3911	pper01100:Metabolic pathways;pper00564:Glycerophospholipid metabolism	GO:0046474:glycerophospholipid biosynthetic process	GO:0016301:kinase activity	-
ppe-miR395e	18766279	PRUPE_ppa024159mg	NCBI_Assembly:GCF_000346465.:	NW_006760194.1	-	15109425	15111343	1918	-	-	-	-

ppe-miR395e	18766395	PRUPE_ppa004526mg	NCBI_Assembly:GCF_000346465.:	NW_006760194.1	-	15163721	15166055	2334	-	GO:0015996: chlorophyll catabolic process	GO:0015171: amino acid transmembrane transporter activity;GO:0015203:polyamine transmembrane transporter activity	GO:0016021:integral component of membrane
ppe-miR395e	18766411	PRUPE_ppa024841mg	NCBI_Assembly:GCF_000346465.:	NW_006760194.1	+	15576031	15577650	1619	-	-	-	-
ppe-miR395e	18766436	PRUPE_ppa004090mg	NCBI_Assembly:GCF_000346465.:	NW_006760194.1	-	1073236	1077570	4334	pper01100:Metabolic pathways;pper01110:Biosynthesis of secondary metabolites;pper01230:Biosynthesis of amino acids;pper00460:Cyanoamino acid metabolism;pper00260:Glycine, serine and threonine metabolism;pper01200:Carbon metabolism;pper00630:Glyoxylate and dicarboxylate metabolism;pper00670:One carbon pool by folate	GO:0006544: glycine metabolic process;GO:0006563:L-serine metabolic process;GO:0035999:tetrahydrofolate interconversion	GO:0004372: glycine hydroxymethyltransferase activity;GO:0030170:pyridoxal phosphate binding	-

ppe-miR395e	18766622	PRUPE_ppa026489mg	NCBI_Assembly:GCF_000346465.:	NW_006760194.1	+	17738551	17742009	3458	pper01100:Metabolic pathways;pper00591:Linoleic acid metabolism;pper00592:alpha-Linolenic acid metabolism	GO:0031408: oxylipin biosynthetic process	GO:0016702: oxidoreductase activity, acting on single donors with incorporation of molecular oxygen, incorporation of two atoms of oxygen;GO:0046872:metal ion binding	-
ppe-miR395e	18766905	PRUPE_ppa010238mg	NCBI_Assembly:GCF_000346465.:	NW_006760194.1	-	17710283	17713864	3581	-	GO:0006626: protein targeting to mitochondrion	-	GO:0005622:intracellular
ppe-miR395e	18766984	PRUPE_ppa002521mg	NCBI_Assembly:GCF_000346465.:	NW_006760194.1	+	15189170	15194284	5114	-	-	-	-
ppe-miR395e	18767040	PRUPE_ppa003275mg	NCBI_Assembly:GCF_000346465.:	NW_006760194.1	-	17767541	17771857	4316	-	-	GO:0005507: copper ion binding;GO:0016491:oxidoreductase activity	-
ppe-miR395e	18767124	PRUPE_ppa009911mg	NCBI_Assembly:GCF_000346465.:	NW_006760194.1	-	16124715	16127302	2587	-	GO:0006621: protein retention in ER lumen	GO:0046923: ER retention sequence binding	GO:0005783:endoplasmic reticulum;GO:0016021:integral component of membrane

ppe-miR395e	18767201	PRUPE_ppa001167mg	NCBI_Assembly:GCF_000346465.:	NW_006760194.1	-	14516906	14520665	3759	-	GO:0006612: protein targeting to membrane;GO:0009963:positive regulation of flavonoid biosynthetic process;GO:0010363:regulation of plant-type hypersensitive response	-	GO:0005622:intracellular;GO:0005886:plasma membrane
ppe-miR395e	18767277	PRUPE_ppa014595mg	NCBI_Assembly:GCF_000346465.:	NW_006760194.1	+	12311873	12312839	966	-	-	-	-
ppe-miR395e	18767294	PRUPE_ppa021363mg	NCBI_Assembly:GCF_000346465.:	NW_006760194.1	+	15369063	15371337	2274	-	GO:0005975: carbohydrate metabolic process	GO:0004553: hydrolase activity, hydrolyzing O-glycosyl compounds	-
ppe-miR395e	18767361	PRUPE_ppa022741mg	NCBI_Assembly:GCF_000346465.:	NW_006760194.1	+	18362540	18363295	755	-	-	-	-
ppe-miR395e	18767547	PRUPE_ppa019221mg	NCBI_Assembly:GCF_000346465.:	NW_006760194.1	+	14405645	14408015	2370	-	GO:0006629:lipid metabolic process	GO:0008081: phosphoric diester hydrolase activity	-
ppe-miR395e	18767822	PRUPE_ppa000722m1g	NCBI_Assembly:GCF_000346465.:	NW_006760194.1	+	13464962	13469159	4197	-	-	-	GO:0005829:cytosol
ppe-miR395e	18768061	PRUPE_ppa012240mg	NCBI_Assembly:GCF_000346465.:	NW_006760194.1	-	14806669	14808745	2076	-	-	-	-
ppe-miR395e	18768202	PRUPE_ppa001622mg	NCBI_Assembly:GCF_000346465.:	NW_006760194.1	+	279145	284899	5754	-	GO:0006265: DNA topological change	GO:0003677: DNA binding;GO:0003917:DNA topoisomerase type I activity;GO:0003918:DNA topoisomerase type II (ATP-hydrolyzing) activity	GO:0005694:chromosome

ppe-miR395e	18768237	PRUPE_ppa011000mg	NCBI_Assembly:GCF_000346465.:	NW_006760194.1	+	20173614	20176339	2725	-	-	GO:0004674:protein serine/threonine kinase activity;GO:0005524:ATP binding	-
ppe-miR395e	18768342	PRUPE_ppb024531mg	NCBI_Assembly:GCF_000346465.:	NW_006760194.1	+	3139028	3139845	817	pper01100:Metabolic pathways;pper01110:Biosynthesis of secondary metabolites;pper00520:Amino sugar and nucleotide sugar metabolism;pper00051:Fructose and mannose metabolism	GO:0009298:GDP-mannose biosynthetic process;GO:0019307:mannose biosynthetic process	GO:0004615:phosphomannomutase activity	GO:0005737:cytoplasm
ppe-miR395e	18768354	PRUPE_ppa006970mg	NCBI_Assembly:GCF_000346465.:	NW_006760194.1	-	2189686	2191207	1521	-	GO:0006826:iron ion transport	-	GO:0009941:chloroplast envelope;GO:0016021:integral component of membrane
ppe-miR395e	18768585	PRUPE_ppa001449mg	NCBI_Assembly:GCF_000346465.:	NW_006760194.1	+	15075595	15078075	2480	-	-	-	-
ppe-miR395e	18768676	PRUPE_ppa009275mg	NCBI_Assembly:GCF_000346465.:	NW_006760194.1	-	19816717	19818077	1360	-	GO:0006334:nucleosome assembly	GO:0003677:DNA binding	GO:0000786:nucleosome;GO:0005634:nucleus
ppe-miR395e	18768855	PRUPE_ppa017519mg	NCBI_Assembly:GCF_000346465.:	NW_006760194.1	+	6131193	6132599	1406	-	GO:0006351:transcription, DNA-templated;GO:0006355:regulation of transcription, DNA-templated	-	-
ppe-miR395e	18768881	PRUPE_ppa010784mg	NCBI_Assembly:GCF_000346465.:	NW_006760194.1	-	12279538	12280787	1249	-	-	-	-

ppe-miR395e	18768995	PRUPE_ppa012821mg	NCBI_Assembly:GCF_000346465.:	NW_006760194.1	+	2798301	2799216	915	-	-	-	-
ppe-miR395e	18769161	PRUPE_ppa012454mg	NCBI_Assembly:GCF_000346465.:	NW_006760201.1	+	15608087	15609932	1845	pper04626:Plant-pathogen interaction	-	GO:0005509: calcium ion binding	-
ppe-miR395e	18769231	PRUPE_ppa007628mg	NCBI_Assembly:GCF_000346465.:	NW_006760201.1	+	16930198	16933784	3586	pper01100:Metabolic pathways;pper01110:Biosynthesis of secondary metabolites;pper00010:Glycolysis / Gluconeogenesis	GO:0005975: carbohydrate metabolic process	GO:0016853:isomerase activity;GO:0030246:carbohydrate binding	GO:0009570:chloroplast stroma;GO:0048046:apoplast
ppe-miR395e	18769274	PRUPE_ppa015779mg	NCBI_Assembly:GCF_000346465.:	NW_006760201.1	+	17155191	17155656	465	-	-	-	-
ppe-miR395e	18769344	PRUPE_ppa011052mg	NCBI_Assembly:GCF_000346465.:	NW_006760201.1	-	16461498	16463069	1571	pper04141:Protein processing in endoplasmic reticulum	GO:0006661: phosphatidylinositol biosynthetic process	GO:0008270: zinc ion binding	-
ppe-miR395e	18769621	PRUPE_ppa007474mg	NCBI_Assembly:GCF_000346465.:	NW_006760201.1	+	20207325	20208672	1347	-	-	-	-
ppe-miR395e	18769648	PRUPE_ppa014386mg	NCBI_Assembly:GCF_000346465.:	NW_006760201.1	-	4511844	4512658	814	-	-	-	-
ppe-miR395e	18769953	PRUPE_ppa026994mg	NCBI_Assembly:GCF_000346465.:	NW_006760201.1	-	13330674	13331185	511	-	GO:0006098: pentose-phosphate shunt;GO:0009073:aromatic amino acid family biosynthetic process;GO:0016226:iron-sulfur cluster assembly;GO:0045893:positive regulation of transcription, DNA-templated	-	GO:0009570:chloroplast stroma

ppe-miR395e	18770083	PRUPE_ppa000647mg	NCBI_Assembly:GCF_000346465.:	NW_006760201.1	-	20582610	20588923	6313	pper03430:Mismatch repair	GO:0000911:cytokinesis by cell plate formation;GO:0006270:DNA replication initiation;GO:0006275:regulation of DNA replication;GO:0006298:mismatch repair;GO:0006306:DNA methylation;GO:0007067:mitotic nuclear division;GO:0007129:synapsis;GO:0008283:cell proliferation;GO:0009909:regulation of flower development;GO:0010389:regulation of G2/M transition of mitotic cell cycle;GO:0016458:gene silencing;GO:0051567:histone H3-K9 methylation	GO:0005524:ATP binding;GO:0030983:mismatched DNA binding	-
ppe-miR395e	18770098	PRUPE_ppa018741mg	NCBI_Assembly:GCF_000346465.:	NW_006760201.1	+	13234455	13235180	725	-	-	-	-
ppe-miR395e	18770112	PRUPE_ppb013602mg	NCBI_Assembly:GCF_000346465.:	NW_006760201.1	+	12290746	12291666	920	-	-	-	-
ppe-miR395e	18770122	PRUPE_ppa005800mg	NCBI_Assembly:GCF_000346465.:	NW_006760201.1	+	11900066	11901882	1816	pper04120:Ubiquitin mediated proteolysis	-	-	-

ppe-miR395e	18770144	PRUPE_ppa022931mg	NCBI_Assembly:GCF_000346465.:	NW_006760201.1	-	20577784	20582043	4259	pper04144:Endocytosis	GO:0009827:plant-type cell wall modification;GO:0009846:pollen germination;GO:0009860:pollen tube growth	GO:0005086:ARF guanyl-nucleotide exchange factor activity	GO:0090406:pollen tube
ppe-miR395e	18770528	PRUPE_ppa019571mg	NCBI_Assembly:GCF_000346465.:	NW_006760201.1	-	20109536	20112716	3180	-	-	GO:0004674:protein serine/threonine kinase activity;GO:0005524:ATP binding	GO:0016021:integral component of membrane
ppe-miR395e	18770741	PRUPE_ppa016201mg	NCBI_Assembly:GCF_000346465.:	NW_006760201.1	+	2223778	2224323	545	-	-	-	-
ppe-miR395e	18770788	PRUPE_ppa024680mg	NCBI_Assembly:GCF_000346465.:	NW_006760201.1	+	3281893	3283162	1269	-	-	-	-
ppe-miR395e	18771598	PRUPE_ppa009437mg	NCBI_Assembly:GCF_000346465.:	NW_006760201.1	-	15261753	15263731	1978	-	GO:0006073:cellular glucan metabolic process;GO:0016998:cell wall macromolecule catabolic process;GO:0042546:cell wall biogenesis;GO:0071555:cell wall organization	GO:0004553:hydrolase activity, hydrolyzing O-glycosyl compounds;GO:0016762:xyloglucan:xyloglucosyl transferase activity	GO:0005618:cell wall;GO:0048046:apoplast
ppe-miR395e	18771682	PRUPE_ppa007189mg	NCBI_Assembly:GCF_000346465.:	NW_006760201.1	+	18223854	18225877	2023	-	-	-	-
ppe-miR395e	18771775	PRUPE_ppb010572mg	NCBI_Assembly:GCF_000346465.:	NW_006760201.1	+	2358432	2359456	1024	-	-	-	-

ppe-miR395e	18771839	PRUPE_ppa024442mg	NCBI_Assembly:GCF_000346465.:	NW_006760201.1	+	8659977	8662300	2323	pper00908:Zeatin biosynthesis	GO:0009823: cytokinin catabolic process;GO:0010089:xylem development; GO:0044036: cell wall macromolecule metabolic process;GO:0048507:meristem development	GO:0008762: UDP-N-acetylmuramate dehydrogenase activity;GO:0019139:cytokinin dehydrogenase activity;GO:0050660:flavin adenine dinucleotide binding	-
ppe-miR395e	18771880	PRUPE_ppb014730mg	NCBI_Assembly:GCF_000346465.:	NW_006760201.1	+	11058590	11059846	1256	-	-	GO:0043531: ADP binding	-

ppe-miR395e	18771926	PRUPE_ppa000944mg	NCBI_Assembly:GCF_000346465.:	NW_006760208.1	-	27412741	27419682	6941	-	GO:0002229:defense response to oomycetes;GO:0006487:protein N-linked glycosylation;GO:0008219:cell death;GO:0009414:response to water deprivation;GO:0009617:response to bacterium;GO:0009620:response to fungus;GO:0009723:response to ethylene;GO:0009788:negative regulation of abscisic acid-activated signaling pathway;GO:0046777:protein autophosphorylation;GO:1900150:regulation of defense response to fungus;GO:1900424:regulation of defense response to	GO:0004709:MAP kinase kinase kinase activity;GO:0004712:protein serine/threonine/tyrosine kinase activity;GO:0005524:ATP binding	GO:0005802:trans-Golgi network;GO:0005829:cytosol
ppe-miR395e	18772137	PRUPE_ppa001677mg	NCBI_Assembly:GCF_000346465.:	NW_006760208.1	-	22763017	22767119	4102	-	-	GO:0015299:solute:proton antiporter activity	GO:0016021:integral component of membrane
ppe-miR395e	18772217	PRUPE_ppa002217mg	NCBI_Assembly:GCF_000346465.:	NW_006760208.1	-	22910610	22913200	2590	-	-	GO:0003824:catalytic activity	-

ppe-miR395e	18772284	PRUPE_ppa004870mg	NCBI_Assembly:GCF_000346465.:	NW_006760208.1	+	25189032	25193057	4025	-	-	GO:0004674:protein serine/threonine kinase activity;GO:0005524:ATP binding	-
ppe-miR395e	18772312	PRUPE_ppa005524mg	NCBI_Assembly:GCF_000346465.:	NW_006760208.1	+	23087122	23091454	4332	-	-	-	-
ppe-miR395e	18772361	PRUPE_ppa019688mg	NCBI_Assembly:GCF_000346465.:	NW_006760208.1	+	284301	287525	3224	-	GO:0000165:MAPK cascade;GO:0006612:protein targeting to membrane;GO:0009595:detection of biotic stimulus;GO:0009697:salicylic acid biosynthetic process;GO:0009862:systemic acquired resistance, salicylic acid mediated signaling pathway;GO:0009867:jasmonic acid mediated signaling pathway;GO:0010200:response to chitin;GO:0010310:regulation of hydrogen peroxide metabolic process;GO:0010363:regulation of plant-type hypersensitive response;GO:	GO:0005315:inorganic phosphate transmembrane transporter activity;GO:0030504:inorganic diphosphate transmembrane transporter activity	GO:0005622:intracellular;GO:0016021:integral component of membrane

ppe-miR395e	18772362	PRUPE_ppa021634mg	NCBI_Assembly:GCF_000346465.:	NW_006760208.1	-	15802525	15803631	1106	-	-	-	-
ppe-miR395e	18772491	PRUPE_ppa008835mg	NCBI_Assembly:GCF_000346465.:	NW_006760208.1	-	21511337	21512335	998	-	-	-	-
ppe-miR395e	18772599	PRUPE_ppa025305mg	NCBI_Assembly:GCF_000346465.:	NW_006760208.1	+	21827888	21831934	4046	-	-	-	-
ppe-miR395e	18772604	PRUPE_ppa022064mg	NCBI_Assembly:GCF_000346465.:	NW_006760208.1	+	2661441	2661890	449	-	-	-	-
ppe-miR395e	18772628	PRUPE_ppa1027138mg	NCBI_Assembly:GCF_000346465.:	NW_006760208.1	-	26588870	26589472	602	-	-	-	-
ppe-miR395e	18772752	PRUPE_ppa004767mg	NCBI_Assembly:GCF_000346465.:	NW_006760208.1	-	7188788	7194025	5237	-	-	GO:0008146: sulfotransferase activity	GO:0016021:integral component of membrane
ppe-miR395e	18773101	PRUPE_ppa014548mg	NCBI_Assembly:GCF_000346465.:	NW_006760208.1	+	25654669	25655829	1160	-	GO:0009409:response to cold	-	GO:0016021:integral component of membrane
ppe-miR395e	18773211	PRUPE_ppa026614mg	NCBI_Assembly:GCF_000346465.:	NW_006760208.1	+	20122964	20123629	665	-	-	GO:0016758:transferase activity, transferring hexosyl groups	-
ppe-miR395e	18773323	PRUPE_ppa013158mg	NCBI_Assembly:GCF_000346465.:	NW_006760208.1	-	27507837	27509796	1959	pper03010:Ribosome	GO:0006412:translation	GO:0000166: nucleotide binding;GO:0003735:structural constituent of ribosome	GO:0005840:ribosome
ppe-miR395e	18773416	PRUPE_ppa009910mg	NCBI_Assembly:GCF_000346465.:	NW_006760208.1	-	13246594	13250421	3827	pper01100:Metabolic pathways;pper01110:Biosynthesis of secondary metabolites;pper01230:Biosynthesis of amino acids;pper00300:Lysine biosynthesis	GO:0009089:lysine biosynthetic process via diaminopimelate	GO:0008839: 4-hydroxy-tetrahydrodipicolinate reductase;GO:0070402:NADPH binding	-

ppe-miR395e	18773486	PRUPE_ppa001740mg	NCBI_Assembly:GCF_000346465.:	NW_006760208.1	-	13580380	13587267	6887	-	GO:0006486:protein glycosylation;GO:0006635:fatty acid beta-oxidation;GO:0016558:protein import into peroxisome matrix	-	GO:0005622:intracellular;GO:0005886:plasma membrane;GO:0009506:plasmodesma
ppe-miR395e	18773640	PRUPE_ppa005989mg	NCBI_Assembly:GCF_000346465.:	NW_006760208.1	-	9811755	9817089	5334	pper04144:Endocytosis	GO:0006623:protein targeting to vacuole;GO:0006635:fatty acid beta-oxidation;GO:0006869:lipid transport;GO:0006891:intra-Golgi vesicle-mediated transport;GO:0007032:endosome organization;GO:0007033:vacuole organization;GO:0010091:trichome branching;GO:0010351:lithium ion transport;GO:0016197:endosomal transport;GO:0016558:protein import into peroxisome matrix	GO:0005524:ATP binding	GO:0005634:nucleus;GO:0005771:multivesicular body;GO:0009506:plasmodesma

ppe-miR395e	18773734	PRUPE_ppa020769mg	NCBI_Assembly:GCF_000346465.:	NW_006760208.1	-	23154560	23157970	3410	-	-	-	GO:0016021:integral component of membrane
ppe-miR395e	18773813	PRUPE_ppa015954mg	NCBI_Assembly:GCF_000346465.:	NW_006760208.1	+	5716921	5717980	1059	-	-	GO:0003677: DNA binding;GO:0003682:chromatin binding	-
ppe-miR395e	18773836	PRUPE_ppb024045mg	NCBI_Assembly:GCF_000346465.:	NW_006760208.1	+	11564154	11564696	542	-	-	-	-
ppe-miR395e	18774282	PRUPE_ppa004341mg	NCBI_Assembly:GCF_000346465.:	NW_006760208.1	-	2340406	2343839	3433	-	-	GO:0004497: monooxygenase activity;GO:0005506:iron ion binding;GO:0016705:oxidoreductase activity, acting on paired donors, with incorporation or reduction of molecular oxygen;GO:0020037:heme binding	-
ppe-miR395e	18774358	PRUPE_ppa007468mg	NCBI_Assembly:GCF_000346465.:	NW_006760208.1	+	6986156	6988676	2520	-	-	GO:0008270: zinc ion binding	-
ppe-miR395e	18774458	PRUPE_ppa001009mg	NCBI_Assembly:GCF_000346465.:	NW_006760208.1	-	6402794	6408935	6141	-	-	GO:0005215:transporter activity;GO:0005524:ATP binding;GO:0016887:ATPase activity	GO:0016021:integral component of membrane
ppe-miR395e	18774493	PRUPE_ppa023974mg	NCBI_Assembly:GCF_000346465.:	NW_006760208.1	-	16028125	16032496	4371	-	-	-	-
ppe-miR395e	18774602	PRUPE_ppa017999mg	NCBI_Assembly:GCF_000346465.:	NW_006760208.1	-	13327555	13330212	2657	-	-	GO:0043531: ADP binding	-
ppe-miR395e	18774660	PRUPE_ppa025652mg	NCBI_Assembly:GCF_000346465.:	NW_006760208.1	-	22592625	22593497	872	-	-	-	-

ppe-miR395e	18774705	PRUPE_ppa023075mg	NCBI_Assembly:GCF_000346465.:	NW_006760208.1	+	24932917	24933419	502	-	-	GO:0009055: electron carrier activity	-
ppe-miR395e	18774744	PRUPE_ppa006015mg	NCBI_Assembly:GCF_000346465.:	NW_006760208.1	+	1703242	1705171	1929	-	-	GO:0016758:transferase activity, transferring hexosyl groups	-
ppe-miR395e	18774828	PRUPE_ppa022793mg	NCBI_Assembly:GCF_000346465.:	NW_006760208.1	-	2786738	2787258	520	-	-	-	-
ppe-miR395e	18774833	PRUPE_ppa019730mg	NCBI_Assembly:GCF_000346465.:	NW_006760208.1	-	26547337	26551592	4255	pper04626:Plant-pathogen interaction	GO:0006826:iron ion transport;GO:0009408:response to heat;GO:0009845:seed germination;GO:0010106:cellular response to iron ion starvation;GO:0010167:response to nitrate;GO:0015706:nitrate transport	GO:0004601: peroxidase activity;GO:0005509:calcium ion binding;GO:0016174:NAD(P)H oxidase activity	GO:0016021:integral component of membrane
ppe-miR395e	18774879	PRUPE_ppa003773mg	NCBI_Assembly:GCF_000346465.:	NW_006760208.1	+	8438554	8441609	3055	-	-	GO:0004674: protein serine/threonine kinase activity;GO:0005524:ATP binding	-
ppe-miR395e	18774882	PRUPE_ppa019085mg	NCBI_Assembly:GCF_000346465.:	NW_006760208.1	+	18963484	18967054	3570	-	-	GO:0043531: ADP binding	-
ppe-miR395e	18775018	PRUPE_ppa003731mg	NCBI_Assembly:GCF_000346465.:	NW_006760208.1	+	21803813	21807645	3832	-	-	-	-
ppe-miR395e	18775033	PRUPE_ppa010197mg	NCBI_Assembly:GCF_000346465.:	NW_006760208.1	-	20429509	20432189	2680	-	-	-	-

ppe-miR395e	18775244	PRUPE_ppa010719mg	NCBI_Assembly:GCF_000346465.:	NW_006760208.1	+	25111136	25114030	2894	-	GO:0009793:embryo development ending in seed dormancy;GO:0016226:iron-sulfur cluster assembly;GO:0043043:peptide biosynthetic process;GO:0045036:protein targeting to chloroplast	GO:0003746:translation elongation factor activity	GO:0009570:chloroplast stroma
ppe-miR395e	18775333	PRUPE_ppa014520mg	NCBI_Assembly:GCF_000346465.:	NW_006760208.1	-	24120752	24121319	567	-	-	-	-
ppe-miR395e	18775728	PRUPE_ppa004996mg	NCBI_Assembly:GCF_000346465.:	NW_006760212.1	+	11188218	11190931	2713	-	GO:0005975:carbohydrate metabolic process;GO:0071555:cell wall organization	GO:0004650:polygalacturonase activity	GO:0005576:extracellular region
ppe-miR395e	18775841	PRUPE_ppa021263mg	NCBI_Assembly:GCF_000346465.:	NW_006760212.1	+	7708826	7710769	1943	pper04120:Ubiquitin mediated proteolysis;pper04141:Protein processing in endoplasmic reticulum	GO:0006511:ubiquitin-dependent protein catabolic process	-	GO:0031461:cullin-RING ubiquitin ligase complex
ppe-miR395e	18775871	PRUPE_ppa020456mg	NCBI_Assembly:GCF_000346465.:	NW_006760212.1	-	16834633	16836753	2120	-	GO:0007067:mitotic nuclear division	-	GO:0000775:chromosome, centromeric region;GO:0005634:nucleus

ppe-miR395e	18775884	PRUPE_ppa019280mg	NCBI_Assembly:GCF_000346465.:	NW_006760212.1	+	3644150	3645239	1089	-	-	GO:0003677: DNA binding;GO:0003682:chromatin binding;GO:0003700:sequence-specific DNA binding transcription factor activity	-
ppe-miR395e	18775934	PRUPE_ppa023247mg	NCBI_Assembly:GCF_000346465.:	NW_006760212.1	-	11986550	11986915	365	-	GO:0006869:lipid transport	GO:0008289:lipid binding	-
ppe-miR395e	18775996	PRUPE_ppa003575mg	NCBI_Assembly:GCF_000346465.:	NW_006760212.1	-	13288009	13293379	5370	-	-	-	-
ppe-miR395e	18776147	PRUPE_ppa017571mg	NCBI_Assembly:GCF_000346465.:	NW_006760212.1	-	15274651	15276452	1801	-	-	GO:0003677: DNA binding;GO:0003682:chromatin binding	-
ppe-miR395e	18776155	PRUPE_ppa013028mg	NCBI_Assembly:GCF_000346465.:	NW_006760212.1	+	6505985	6508109	2124	-	-	-	-
ppe-miR395e	18776171	PRUPE_ppa001787mg	NCBI_Assembly:GCF_000346465.:	NW_006760212.1	+	12885277	12890862	5585	pper03013:RNA transport	-	-	-
ppe-miR395e	18776221	PRUPE_ppa002101mg	NCBI_Assembly:GCF_000346465.:	NW_006760212.1	-	16418437	16422940	4503	-	-	GO:0004601: peroxidase activity;GO:0005509:calcium ion binding;GO:0050664:oxidoreductase activity, acting on NAD(P)H, oxygen as acceptor	GO:0016021:integral component of membrane
ppe-miR395e	18776337	PRUPE_ppa015583mg	NCBI_Assembly:GCF_000346465.:	NW_006760212.1	-	17115300	17117309	2009	-	-	-	-
ppe-miR395e	18776382	PRUPE_ppa007507mg	NCBI_Assembly:GCF_000346465.:	NW_006760212.1	+	11257461	11260583	3122	-	-	GO:0003676: nucleic acid binding	-
ppe-miR395e	18776478	PRUPE_ppa012437mg	NCBI_Assembly:GCF_000346465.:	NW_006760212.1	+	3469223	3470205	982	-	-	-	-

ppe-miR395e	18776559	PRUPE_ppa016601mg	NCBI_Assembly:GCF_000346465.:	NW_006760212.1	+	5002067	5005113	3046	-	-	GO:0004497: monooxygenase activity;GO:0005506:iron ion binding;GO:0016705:oxidoreductase activity, acting on paired donors, with incorporation or reduction of molecular oxygen;GO:0020037:heme binding	-
ppe-miR395e	18776649	PRUPE_ppa005059mg	NCBI_Assembly:GCF_000346465.:	NW_006760212.1	+	1402343	1405240	2897	pper00906:Carotenoid biosynthesis	-	GO:0004497: monooxygenase activity;GO:0005506:iron ion binding;GO:0016705:oxidoreductase activity, acting on paired donors, with incorporation or reduction of molecular oxygen;GO:0020037:heme binding	-
ppe-miR395e	18776673	PRUPE_ppa006955mg	NCBI_Assembly:GCF_000346465.:	NW_006760212.1	+	5849713	5854095	4382	-	GO:0006457: protein folding;GO:0006979:response to oxidative stress	GO:0003755: peptidyl-prolyl cis-trans isomerase activity	-

ppe-miR395e	18776736	PRUPE_ppa013288mg	NCBI_Assembly:GCF_000346465.:	NW_006760212.1	-	11950685	11951535	850	-	-	-	-
ppe-miR395e	18776798	PRUPE_ppa004611mg	NCBI_Assembly:GCF_000346465.:	NW_006760212.1	+	11826137	11828782	2645	-	-	-	-
ppe-miR395e	18776851	PRUPE_ppa006018mg	NCBI_Assembly:GCF_000346465.:	NW_006760212.1	-	13524131	13525691	1560	pper00073:Cutin, suberine and wax biosynthesis	-	GO:0016747:transferase activity, transferring acyl groups other than amino-acyl groups	-
ppe-miR395e	18777099	PRUPE_ppa003155mg	NCBI_Assembly:GCF_000346465.:	NW_006760212.1	-	15383687	15386393	2706	-	-	GO:0005215:transporter activity	GO:0016020:membrane
ppe-miR395e	18777147	PRUPE_ppa005023mg	NCBI_Assembly:GCF_000346465.:	NW_006760212.1	+	11821973	11825409	3436	-	-	-	-
ppe-miR395e	18777190	PRUPE_ppa015124mg	NCBI_Assembly:GCF_000346465.:	NW_006760212.1	-	882246	884401	2155	-	-	-	-
ppe-miR395e	18777324	PRUPE_ppa018016mg	NCBI_Assembly:GCF_000346465.:	NW_006760212.1	-	9681214	9681612	398	-	GO:0006662:glycerol ether metabolic process;GO:0045454:cell redox homeostasis	GO:0015035:protein disulfide oxidoreductase activity	GO:0005623:cell
ppe-miR395e	18777331	PRUPE_ppa008400mg	NCBI_Assembly:GCF_000346465.:	NW_006760212.1	-	7773985	7777822	3837	-	GO:0043407:negative regulation of MAP kinase activity	GO:0004725:protein tyrosine phosphatase activity;GO:0008138:protein tyrosine/serine/threonine phosphatase activity	-

ppe-miR395e	18777444	PRUPE_ppa009898mg	NCBI_Assembly:GCF_000346465.:	NW_006760212.1	-	2655889	2657874	1985	-	-	GO:0003677: DNA binding;GO:0003682:chromatin binding;GO:0003700:sequence-specific DNA binding transcription factor activity	-
ppe-miR395e	18777496	PRUPE_ppa009609mg	NCBI_Assembly:GCF_000346465.:	NW_006760212.1	-	15671370	15674389	3019	-	-	-	-
ppe-miR395e	18777712	PRUPE_ppa006936mg	NCBI_Assembly:GCF_000346465.:	NW_006760212.1	+	5321105	5328026	6921	-	GO:0016571: histone methylation;GO:0048451:petal formation;GO:0048453:sepal formation	-	-
ppe-miR395e	18777880	PRUPE_ppa002015mg	NCBI_Assembly:GCF_000346465.:	NW_006760212.1	-	12506374	12512286	5912	-	-	GO:0004672: protein kinase activity;GO:0005524:ATP binding	-
ppe-miR395e	18777991	PRUPE_ppa011001mg	NCBI_Assembly:GCF_000346465.:	NW_006760212.1	+	6736793	6742010	5217	-	-	-	-
ppe-miR395e	18778057	PRUPE_ppa011954mg	NCBI_Assembly:GCF_000346465.:	NW_006760212.1	+	17622024	17623067	1043	-	-	GO:0003677: DNA binding;GO:0003682:chromatin binding	-
ppe-miR395e	18778201	PRUPE_ppa013681mg	NCBI_Assembly:GCF_000346465.:	NW_006760220.1	-	1769292	1769886	594	-	GO:0007017: microtubule-based process	-	GO:0005875:microtubule associated complex

ppe-miR395e	18778250	PRUPE_ppa010162mg	NCBI_Assembly:GCF_000346465.:	NW_006760220.1	+	2902954	2906436	3482	-	GO:0006096:glycolytic process;GO:0006833:water transport;GO:0006972:hyperosmotic response;GO:0007030:Golgi organization;GO:0009266:response to temperature stimulus;GO:0009651:response to salt stress;GO:0009750:response to fructose;GO:0019288:isopentenyl diphosphate biosynthetic process, methylerythritol 4-phosphate pathway;GO:0019344:cysteine biosynthetic process;GO:0032880:regulation of protein localization;GO:0042744:hydrogen	GO:0005509:calcium ion binding	GO:0005634:nucleus;GO:0005829:cytosol;GO:0005886:plasma membrane
ppe-miR395e	18778305	PRUPE_ppa016168mg	NCBI_Assembly:GCF_000346465.:	NW_006760220.1	+	4305742	4309784	4042	-	-	-	-
ppe-miR395e	18778324	PRUPE_ppa007058mg	NCBI_Assembly:GCF_000346465.:	NW_006760220.1	-	8482652	8485600	2948	-	-	-	-

ppe-miR395e	18778417	PRUPE_ppa005278mg	NCBI_Assembly:GCF_000346465.:	NW_006760220.1	+	13875390	13884446	9056	pper04122:Sulfur relay system	GO:0002143:tRNA wobble position uridine thiolation;GO:0006777:Mo-molybdopterin cofactor biosynthetic process;GO:0018192:enzyme active site formation via L-cysteine persulfide	GO:0004792:thiosulfate sulfurtransferase activity;GO:0005524:ATP binding;GO:0008265:Mo-molybdopterin cofactor sulfurase activity;GO:0008641:small protein activating enzyme activity;GO:0016779:nucleotidyltransferase activity;GO:0046872:metal ion binding	GO:0005829:cytosol
ppe-miR395e	18778429	PRUPE_ppa005033mg	NCBI_Assembly:GCF_000346465.:	NW_006760220.1	+	8462021	8467187	5166	pper01100:Metabolic pathways;pper00510:N-Glycan biosynthesis	GO:0016126:sterol biosynthetic process;GO:0030244:cellulose biosynthetic process;GO:0046520:sphingoid biosynthetic process;GO:0048193:Golgi vesicle transport	GO:0016757:transferase activity, transferring glycosyl groups	GO:0005622:intracellular
ppe-miR395e	18778640	PRUPE_ppa022486mg	NCBI_Assembly:GCF_000346465.:	NW_006760220.1	-	2187892	2189560	1668	-	-	-	-
ppe-miR395e	18778687	PRUPE_ppa022500mg	NCBI_Assembly:GCF_000346465.:	NW_006760220.1	-	13111873	13112571	698	-	-	-	-
ppe-miR395e	18778738	PRUPE_ppa016336mg	NCBI_Assembly:GCF_000346465.:	NW_006760220.1	-	10488889	10489611	722	-	-	-	-

ppe-miR395e	18778944	PRUPE_ppa000567mg	NCBI_Assembly:GCF_000346465.:	NW_006760220.1	+	29673435	29680787	7352	-	GO:0030244:cellulose biosynthetic process;GO:0071555:cell wall organization	GO:0008270:zinc ion binding;GO:0016760:cellulose synthase (UDP-forming) activity	GO:0005886:plasma membrane;GO:0016021:integral component of membrane
ppe-miR395e	18779075	PRUPE_ppa014055mg	NCBI_Assembly:GCF_000346465.:	NW_006760220.1	-	11513534	11514029	495	-	-	-	-
ppe-miR395e	18779097	PRUPE_ppa019045mg	NCBI_Assembly:GCF_000346465.:	NW_006760220.1	+	8379912	8380797	885	pper00480:Glutathione metabolism	-	-	-
ppe-miR395e	18779219	PRUPE_ppa002371mg	NCBI_Assembly:GCF_000346465.:	NW_006760220.1	-	15937740	15940017	2277	-	-	-	-
ppe-miR395e	18779332	PRUPE_ppa016555mg	NCBI_Assembly:GCF_000346465.:	NW_006760220.1	+	10384024	10385982	1958	-	-	GO:0004674:protein serine/threonine kinase activity;GO:0005524:ATP binding;GO:0030246:carbohydrate binding	-
ppe-miR395e	18779383	PRUPE_ppa001166mg	NCBI_Assembly:GCF_000346465.:	NW_006760220.1	+	6194205	6198347	4142	-	-	GO:0008270:zinc ion binding	-
ppe-miR395e	18779419	PRUPE_ppa004121mg	NCBI_Assembly:GCF_000346465.:	NW_006760220.1	-	2132531	2134568	2037	-	-	-	-
ppe-miR395e	18779489	PRUPE_ppa025550mg	NCBI_Assembly:GCF_000346465.:	NW_006760220.1	-	9223298	9224657	1359	-	-	-	-

ppe-miR395e	18779569	PRUPE_ppa009670mg	NCBI_Assembly:GCF_000346465.:	NW_006760220.1	+	13283745	13286914	3169	-	GO:0009965:leaf morphogenesis;GO:0010027:thylakoid membrane organization;GO:0015979:photosynthesis;GO:0030154:cell differentiation	GO:0005509:calcium ion binding	GO:0009534:chloroplast thylakoid;GO:0009570:chloroplast stroma;GO:0009654:photosystem II oxygen evolving complex;GO:0019898:extrinsic component of membrane;GO:0031977:thylakoid lumen
ppe-miR395e	18779678	PRUPE_ppa023453mg	NCBI_Assembly:GCF_000346465.:	NW_006760220.1	-	28354121	28355432	1311	-	-	GO:0004672:protein kinase activity;GO:0005524:ATP binding	-
ppe-miR395e	18779739	PRUPE_ppa021722mg	NCBI_Assembly:GCF_000346465.:	NW_006760220.1	-	4705853	4710386	4533	-	-	GO:0004672:protein kinase activity;GO:0005509:calcium ion binding;GO:0005524:ATP binding;GO:0030247:polysaccharide binding	-
ppe-miR395e	18779787	PRUPE_ppa014555mg	NCBI_Assembly:GCF_000346465.:	NW_006760220.1	+	8375736	8376625	889	pper00480:Glutathione metabolism	-	-	-
ppe-miR395e	18779841	PRUPE_ppa024549mg	NCBI_Assembly:GCF_000346465.:	NW_006760220.1	-	131827	132477	650	-	-	GO:0004857:enzyme inhibitor activity;GO:0030599:pectinesterase activity	-
ppe-miR395e	18779843	PRUPE_ppa017340mg	NCBI_Assembly:GCF_000346465.:	NW_006760220.1	-	13589425	13589709	284	-	GO:0006869:lipid transport	GO:0008289:lipid binding	-

ppe-miR395e	18780148	PRUPE_ppa026458mg	NCBI_Assembly:GCF_000346465.:	NW_006760220.1	-	10591493	10594680	3187	-	-	GO:0015238: drug transmembrane transporter activity;GO:0015297:antiporter activity	GO:0016020:membrane
ppe-miR395e	18780303	PRUPE_ppa023993mg	NCBI_Assembly:GCF_000346465.:	NW_006760220.1	-	17800797	17802832	2035	-	-	GO:0004674: protein serine/threonine kinase activity;GO:0005524:ATP binding;GO:0030246:carbohydrate binding	-
ppe-miR395e	18780894	PRUPE_ppa015941mg	NCBI_Assembly:GCF_000346465.:	NW_006760220.1	+	10347778	10350422	2644	-	-	GO:0004672: protein kinase activity;GO:0005524:ATP binding;GO:0030246:carbohydrate binding	-
ppe-miR395e	18781233	PRUPE_ppa009038mg	NCBI_Assembly:GCF_000346465.:	NW_006760220.1	-	9508052	9509218	1166	-	GO:0005975: carbohydrate metabolic process	GO:0004553: hydrolase activity, hydrolyzing O-glycosyl compounds	-
ppe-miR395e	18781525	PRUPE_ppa019606mg	NCBI_Assembly:GCF_000346465.:	NW_006760220.1	+	7367813	7369080	1267	-	-	-	-
ppe-miR395e	18781581	PRUPE_ppa012119mg	NCBI_Assembly:GCF_000346465.:	NW_006760268.1	-	8681723	8684270	2547	pper03010:Ribosome	GO:0006412:translation	GO:0003735: structural constituent of ribosome	GO:0005840:ribosome
ppe-miR395e	18781663	PRUPE_ppa023239mg	NCBI_Assembly:GCF_000346465.:	NW_006760268.1	+	10494335	10497401	3066	-	-	GO:0004672: protein kinase activity;GO:0005524:ATP binding	GO:0016021:integral component of membrane
ppe-miR395e	18781694	PRUPE_ppa020648mg	NCBI_Assembly:GCF_000346465.:	NW_006760268.1	-	19633174	19633872	698	-	-	-	-

ppe-miR395e	18782884	PRUPE_ppa007979mg	NCBI_Assembly:GCF_000346465.:	NW_006760268.1	+	14103636	14105181	1545	-	-	GO:0016788: hydrolase activity, acting on ester bonds	-
ppe-miR395e	18782887	PRUPE_ppa000084mg	NCBI_Assembly:GCF_000346465.:	NW_006760268.1	-	10735617	10755622	20005	pper03013:RNA transport;pper03040:Spliceosome	-	GO:0000155: phosphorelay sensor kinase activity	GO:0005622:intracellular;GO:0016020:membrane
ppe-miR395e	18782922	PRUPE_ppb021823mg	NCBI_Assembly:GCF_000346465.:	NW_006760268.1	+	8417859	8418059	200	-	GO:0006351:transcription, DNA-templated;GO:0006355:regulation of transcription, DNA-templated	GO:0003677: DNA binding	GO:0005634:nucleus
ppe-miR395e	18783049	PRUPE_ppa023191mg	NCBI_Assembly:GCF_000346465.:	NW_006760268.1	-	15141364	15141746	382	-	-	-	-
ppe-miR395e	18783136	PRUPE_ppa027005mg	NCBI_Assembly:GCF_000346465.:	NW_006760268.1	+	10916107	10917864	1757	-	-	-	GO:0016021:integral component of membrane
ppe-miR395e	18783257	PRUPE_ppa019394mg	NCBI_Assembly:GCF_000346465.:	NW_006760268.1	-	518061	518282	221	-	-	-	-
ppe-miR395e	18783352	PRUPE_ppa010252mg	NCBI_Assembly:GCF_000346465.:	NW_006760268.1	-	2149391	2150644	1253	-	-	GO:0003677: DNA binding;GO:0003682:chromatin binding	-
ppe-miR395e	18783546	PRUPE_ppa013605mg	NCBI_Assembly:GCF_000346465.:	NW_006760268.1	+	18357758	18358496	738	-	-	-	-
ppe-miR395e	18783754	PRUPE_ppa009608mg	NCBI_Assembly:GCF_000346465.:	NW_006760268.1	-	13672078	13673293	1215	-	GO:0006073: cellular glucan metabolic process;GO:0071555:cell wall organization	GO:0004553: hydrolase activity, hydrolyzing O-glycosyl compounds;GO:0016762:xyloglucan:xyloglucosyl transferase activity	GO:0005618:cell wall;GO:0048046:apoplast

ppe-miR395e	18783829	PRUPE_ppa018520mg	NCBI_Assembly:GCF_000346465.:	NW_006760268.1	-	1732527	1733536	1009	-	GO:0006633:fatty acid biosynthetic process	-	-
ppe-miR395e	18783831	PRUPE_ppa002420mg	NCBI_Assembly:GCF_000346465.:	NW_006760268.1	+	20838318	20841102	2784	-	-	GO:0004672: protein kinase activity;GO:0005524:ATP binding	-
ppe-miR395e	18783864	PRUPE_ppa021381mg	NCBI_Assembly:GCF_000346465.:	NW_006760268.1	-	7303609	7303890	281	-	-	GO:0003676: nucleic acid binding	-
ppe-miR395e	18783889	PRUPE_ppa013551mg	NCBI_Assembly:GCF_000346465.:	NW_006760268.1	+	19199756	19202490	2734	-	-	-	-
ppe-miR395e	18784101	PRUPE_ppa025523mg	NCBI_Assembly:GCF_000346465.:	NW_006760268.1	-	9057162	9057467	305	-	-	-	-
ppe-miR395e	18784364	PRUPE_ppa007883mg	NCBI_Assembly:GCF_000346465.:	NW_006760268.1	-	19548346	19549836	1490	-	GO:0006351:transcription, DNA-templated;GO:0006355:regulation of transcription, DNA-templated	GO:0003677: DNA binding	GO:0005634:nucleus
ppe-miR395e	18784376	PRUPE_ppa026806mg	NCBI_Assembly:GCF_000346465.:	NW_006760268.1	+	20610313	20612423	2110	-	-	-	-
ppe-miR395e	18784552	PRUPE_ppa022509mg	NCBI_Assembly:GCF_000346465.:	NW_006760324.1	+	4978029	4980145	2116	-	-	-	-
ppe-miR395e	18784729	PRUPE_ppa026051mg	NCBI_Assembly:GCF_000346465.:	NW_006760324.1	-	19281205	19282339	1134	-	-	-	-
ppe-miR395e	18784739	PRUPE_ppa018480mg	NCBI_Assembly:GCF_000346465.:	NW_006760324.1	+	9570543	9571153	610	-	-	-	-
ppe-miR395e	18784747	PRUPE_ppa000247mg	NCBI_Assembly:GCF_000346465.:	NW_006760324.1	-	439117	443666	4549	-	-	GO:0043531: ADP binding	-
ppe-miR395e	18784803	PRUPE_ppa023853mg	NCBI_Assembly:GCF_000346465.:	NW_006760324.1	-	19069154	19070052	898	-	-	GO:0003677: DNA binding;GO:0003682:chromatin binding	-
ppe-miR395e	18784853	PRUPE_ppa008042mg	NCBI_Assembly:GCF_000346465.:	NW_006760324.1	-	25669715	25673674	3959	-	GO:0006470: protein dephosphorylation;GO:0048767:root hair elongation	GO:0004722: protein serine/threonine phosphatase activity;GO:0046872:metal ion binding	-

ppe-miR395e	18784886	PRUPE_ppa011359mg	NCBI_Assembly:GCF_000346465.:	NW_006760324.1	-	21998093	21999918	1825	-	-	GO:0003676:nucleic acid binding;GO:0008270:zinc ion binding	-
ppe-miR395e	18785001	PRUPE_ppa024230mg	NCBI_Assembly:GCF_000346465.:	NW_006760324.1	+	14525962	14526949	987	-	-	-	-
ppe-miR395e	18785011	PRUPE_ppa013167mg	NCBI_Assembly:GCF_000346465.:	NW_006760324.1	-	24951504	24952210	706	-	-	-	-
ppe-miR395e	18785058	PRUPE_ppa000113mg	NCBI_Assembly:GCF_000346465.:	NW_006760324.1	+	25747437	25755998	8561	-	-	GO:0003677:DNA binding;GO:0008270:zinc ion binding	-
ppe-miR395e	18785081	PRUPE_ppa010386mg	NCBI_Assembly:GCF_000346465.:	NW_006760324.1	-	16158063	16160063	2000	-	-	-	-
ppe-miR395e	18785086	PRUPE_ppa001496mg	NCBI_Assembly:GCF_000346465.:	NW_006760324.1	-	25388663	25391454	2791	-	-	-	-
ppe-miR395e	18785127	PRUPE_ppa003890mg	NCBI_Assembly:GCF_000346465.:	NW_006760324.1	+	22929782	22932643	2861	pper01100:Metabolic pathways;pper00562:Inositol phosphate metabolism;pper04070:Phosphatidylinositol signaling system	GO:0016042:lipid catabolic process;GO:0035556:intracellular signal transduction	GO:0004435:phosphatidylinositol phospholipase C activity;GO:0004871:signal transducer activity	GO:0005622:intracellular
ppe-miR395e	18785188	PRUPE_ppa011410mg	NCBI_Assembly:GCF_000346465.:	NW_006760324.1	-	8273826	8280710	6884	-	-	-	GO:0005829:cytosol
ppe-miR395e	18785226	PRUPE_ppa003202mg	NCBI_Assembly:GCF_000346465.:	NW_006760324.1	+	22028333	22032770	4437	pper00970:Aminoacyl-tRNA biosynthesis;pper00450:Selenocompound metabolism	GO:0006431:methionyl-tRNA aminoacylation;GO:0048481:ovule development	GO:0004825:methionine-tRNA ligase activity;GO:0005524:ATP binding	GO:0005739:mitochondrion;GO:0009570:chloroplast stroma
ppe-miR395e	18785377	PRUPE_ppa009435mg	NCBI_Assembly:GCF_000346465.:	NW_006760324.1	-	25422966	25425021	2055	-	-	-	-

ppe-miR395e	18785423	PRUPE_ppa025412mg	NCBI_Assembly:GCF_000346465.:	NW_006760324.1	-	20658793	20659218	425	pper04120:Ubiquitin mediated proteolysis;pper04141:Protein processing in endoplasmic reticulum	GO:0006511: ubiquitin-dependent protein catabolic process	-	-
ppe-miR395e	18785468	PRUPE_ppa010803mg	NCBI_Assembly:GCF_000346465.:	NW_006760324.1	+	17396793	17399861	3068	-	GO:0016559: peroxisome fission	-	GO:0005779:integral component of peroxisomal membrane
ppe-miR395e	18785492	PRUPE_ppa003289mg	NCBI_Assembly:GCF_000346465.:	NW_006760324.1	-	23198695	23202687	3992	-	-	GO:0008168: methyltransferase activity	-
ppe-miR395e	18785605	PRUPE_ppa016627mg	NCBI_Assembly:GCF_000346465.:	NW_006760324.1	-	18641896	18644638	2742	-	-	GO:0008270: zinc ion binding	-
ppe-miR395e	18785677	PRUPE_ppa018600mg	NCBI_Assembly:GCF_000346465.:	NW_006760324.1	+	18099703	18101691	1988	-	-	-	-

ppe-miR395e	18785682	PRUPE_ppa011130mg	NCBI_Assembly:GCF_000346465.:	NW_006760324.1	-	13708533	13709230	697	pper03010:Ribosome	GO:0006364:rRNA processing;GO:0006412:translation;GO:0010207:photosystem II assembly;GO:0015995:chlorophyll biosynthetic process;GO:0019288:isopentenyl diphosphate biosynthetic process, methylerythritol 4-phosphate pathway;GO:0045036:protein targeting to chloroplast	GO:0003735:structural constituent of ribosome	GO:0005840:ribosome;GO:0009570:chloroplast stroma;GO:0009941:chloroplast envelope;GO:0016020:membrane
ppe-miR395e	18785745	PRUPE_ppa015741mg	NCBI_Assembly:GCF_000346465.:	NW_006760324.1	+	2490159	2490425	266	-	-	-	-
ppe-miR395e	18785790	PRUPE_ppa010737mg	NCBI_Assembly:GCF_000346465.:	NW_006760324.1	-	8312798	8317814	5016	-	GO:0006623:protein targeting to vacuole;GO:0007033:vacuole organization;GO:0016192:vesicle-mediated transport	-	GO:0005768:endosome;GO:0016021:integral component of membrane;GO:0031201:SNARE complex
ppe-miR395e	18785838	PRUPE_ppa013726mg	NCBI_Assembly:GCF_000346465.:	NW_006760324.1	-	26004055	26005205	1150	-	-	-	-
ppe-miR395e	18785944	PRUPE_ppa022813mg	NCBI_Assembly:GCF_000346465.:	NW_006760324.1	-	1802711	1804831	2120	-	-	GO:0004252:serine-type endopeptidase activity	-

ppe-miR395e	18786011	PRUPE_ppa021814mg	NCBI_Assembly:GCF_000346465.:	NW_006760324.1	+	13697799	13702325	4526	pper00360:Phenylalanine metabolism;pper01100:Metabolic pathways;pper01110:Biosynthesis of secondary metabolites;pper00940:Phenylpropanoid biosynthesis;pper00941:Flavonoid biosynthesis;pper00945:Stilbenoid, diarylheptanoid and gingerol biosynthesis	-	GO:0008171: O-methyltransferase activity	-
ppe-miR395e	18786098	PRUPE_ppa015805mg	NCBI_Assembly:GCF_000346465.:	NW_006760324.1	+	12823566	12823991	425	pper04120:Ubiquitin mediated proteolysis;pper04141:Protein processing in endoplasmic reticulum	GO:0006511: ubiquitin-dependent protein catabolic process	-	-
ppe-miR395e	18786124	PRUPE_ppa016360mg	NCBI_Assembly:GCF_000346465.:	NW_006760324.1	+	4017891	4018541	650	-	-	GO:0003676: nucleic acid binding	-
ppe-miR395e	18786192	PRUPE_ppa011500mg	NCBI_Assembly:GCF_000346465.:	NW_006760324.1	+	9657770	9658575	805	-	-	-	GO:0005739:mitochondrion
ppe-miR395e	18786197	PRUPE_ppa015461mg	NCBI_Assembly:GCF_000346465.:	NW_006760324.1	+	556851	561032	4181	-	-	GO:0043531: ADP binding	-
ppe-miR395e	18786304	PRUPE_ppa000274mg	NCBI_Assembly:GCF_000346465.:	NW_006760324.1	-	472157	477921	5764	-	-	GO:0043531: ADP binding	-

ppe-miR395e	18786544	PRUPE_ppa021740mg	NCBI_Assembly:GCF_000346465.:	NW_006760324.1	-	20290490	20292256	1766	-	GO:0006351:transcription, DNA-templated;GO:0006355:regulation of transcription, DNA-templated	GO:0003677: DNA binding	GO:0005634:nucleus
ppe-miR395e	18786695	PRUPE_ppa006786mg	NCBI_Assembly:GCF_000346465.:	NW_006760324.1	-	20357554	20360409	2855	-	-	GO:0016787: hydrolase activity	-
ppe-miR395e	18786713	PRUPE_ppa008765mg	NCBI_Assembly:GCF_000346465.:	NW_006760324.1	+	23842589	23845231	2642	-	-	GO:0004784: superoxide dismutase activity;GO:0005507:copper ion binding;GO:0046872:metal ion binding	GO:0005739:mitochondrion;GO:0005886:plasma membrane;GO:0009579:thylakoid;GO:0009941:chloroplast envelope
ppe-miR395e	18786729	PRUPE_ppa025228mg	NCBI_Assembly:GCF_000346465.:	NW_006760324.1	-	18021496	18024036	2540	-	-	-	-
ppe-miR395e	18786760	PRUPE_ppa004425mg	NCBI_Assembly:GCF_000346465.:	NW_006760324.1	-	25844232	25849372	5140	pper03040:Spliceosome	-	GO:0003676: nucleic acid binding;GO:0008270:zinc ion binding	GO:0005634:nucleus
ppe-miR395e	18787168	PRUPE_ppa005723mg	NCBI_Assembly:GCF_000346465.:	NW_006760324.1	+	10152771	10157648	4877	-	-	-	-
ppe-miR395e	18787216	PRUPE_ppa016239mg	NCBI_Assembly:GCF_000346465.:	NW_006760324.1	+	8139069	8140763	1694	pper01100:Metabolic pathways;pper00240:Pyrimidine metabolism	GO:0006221: pyrimidine nucleotide biosynthetic process	GO:0003883: CTP synthase activity	-

ppe-miR395e	18787253	PRUPE_ppa009184mg	NCBI_Assembly:GCF_000346465.:	NW_006760324.1	-	22184393	22186450	2057	-	GO:0002679:respiratory burst involved in defense response;GO:0009611:response to wounding;GO:0009612:response to mechanical stimulus;GO:0009693:ethylene biosynthetic process;GO:0010200:response to chitin;GO:0030968:endoplasmic reticulum unfolded protein response;GO:0035556:intracellular signal transduction;GO:0052542:defense response by callose deposition	-	GO:0005622:intracellular

ppe-miR395e	18787543	PRUPE_ppa007731mg	NCBI_Assembly:GCF_000346465.:	NW_006760324.1	+	26347312	26349788	2476	-	GO:0006338:chromatin remodeling;GO:0006355:regulation of transcription, DNA-templated;GO:0008284:positive regulation of cell proliferation;GO:0009909:regulation of flower development;GO:0042742:defense response to bacterium	-	GO:0005634:nucleus
ppe-miR395e	18787619	PRUPE_ppa026568mg	NCBI_Assembly:GCF_000346465.:	NW_006760324.1	-	613905	614144	239	-	-	-	-

ppe-miR395e	18787656	PRUPE_ppa025392mg	NCBI_Assembly:GCF_000346465.:	NW_006760324.1	-	26307434	26309277	1843	-	GO:0000278: mitotic cell cycle;GO:0003002:regionalization;GO:0006325:chromatin organization; GO:0007062: sister chromatid cohesion;GO:0007131:reciprocal meiotic recombination;GO:0007155: cell adhesion;GO:0008284:positive regulation of cell proliferation;GO:0009410:response to xenobiotic stimulus;GO:0009913:epidermal cell differentiation;GO:0010090:trichome morphogenesis;GO:0030307:positive regulation of cell growth;GO:0033044:regulation of	GO:0003690: double-stranded DNA binding	GO:0009330:DNA topoisomerase complex (ATP-hydrolyzing)

ppe-miR395e	18788206	PRUPE_ppa001184mg	NCBI_Assembly:GCF_000346465.:	NW_006760385.1	-	45498552	45501691	3139	-	GO:0002237:response to molecule of bacterial origin;GO:0006612:protein targeting to membrane;GO:0007165:signal transduction;GO:0009963:positive regulation of flavonoid biosynthetic process;GO:0010051:xylem and phloem pattern formation;GO:0010103:stomatal complex morphogenesis;GO:0010363:regulation of plant-type hypersensitive response;GO:0048443:stamen development	GO:0004674:protein serine/threonine kinase activity;GO:0005524:ATP binding	GO:0005622:intracellular
ppe-miR395e	18788452	PRUPE_ppa001811mg	NCBI_Assembly:GCF_000346465.:	NW_006760385.1	-	39741082	39745109	4027	-	-	GO:0004674:protein serine/threonine kinase activity;GO:0005524:ATP binding	-
ppe-miR395e	18788497	PRUPE_ppa010741mg	NCBI_Assembly:GCF_000346465.:	NW_006760385.1	+	29926223	29927821	1598	-	-	-	-
ppe-miR395e	18788617	PRUPE_ppa004032mg	NCBI_Assembly:GCF_000346465.:	NW_006760385.1	+	20252596	20258405	5809	-	-	-	-
ppe-miR395e	18788901	PRUPE_ppa013963mg	NCBI_Assembly:GCF_000346465.:	NW_006760385.1	-	35670562	35672619	2057	-	-	-	-

ppe-miR395e	18788922	PRUPE_ppa021372mg	NCBI_Assembly:GCF_000346465.:	NW_006760385.1	+	23717589	23720651	3062	-	-	-	-
ppe-miR395e	18788928	PRUPE_ppb022800mg	NCBI_Assembly:GCF_000346465.:	NW_006760385.1	+	46240281	46242449	2168	-	GO:0015074: DNA integration	GO:0003676: nucleic acid binding	GO:0005634:nucleus
ppe-miR395e	18789333	PRUPE_ppa009225mg	NCBI_Assembly:GCF_000346465.:	NW_006760385.1	+	34637405	34638801	1396	-	-	-	-
ppe-miR395e	18789337	PRUPE_ppa026534mg	NCBI_Assembly:GCF_000346465.:	NW_006760385.1	-	23227246	23228133	887	-	-	-	-
ppe-miR395e	18789348	PRUPE_ppa021895mg	NCBI_Assembly:GCF_000346465.:	NW_006760385.1	+	29836077	29837105	1028	-	-	-	-
ppe-miR395e	18789368	PRUPE_ppa008274mg	NCBI_Assembly:GCF_000346465.:	NW_006760385.1	-	35320464	35321936	1472	pper03015:mRNA surveillance pathway	GO:0008283: cell proliferation	GO:0000166: nucleotide binding;GO:0003727:single-stranded RNA binding	-
ppe-miR395e	18789561	PRUPE_ppa002152mg	NCBI_Assembly:GCF_000346465.:	NW_006760385.1	-	33653546	33657734	4188	-	-	GO:0004672: protein kinase activity;GO:0005524:ATP binding	-
ppe-miR395e	18789581	PRUPE_ppa023705mg	NCBI_Assembly:GCF_000346465.:	NW_006760385.1	-	12836048	12838881	2833	-	-	GO:0003676: nucleic acid binding;GO:0008408:3'-5' exonuclease activity	-
ppe-miR395e	18789594	PRUPE_ppa024271mg	NCBI_Assembly:GCF_000346465.:	NW_006760385.1	-	28245756	28247186	1430	-	-	GO:0016758:transferase activity, transferring hexosyl groups	-
ppe-miR395e	18789624	PRUPE_ppa016599mg	NCBI_Assembly:GCF_000346465.:	NW_006760385.1	+	42273170	42275476	2306	-	-	-	-
ppe-miR395e	18789907	PRUPE_ppa018287mg	NCBI_Assembly:GCF_000346465.:	NW_006760385.1	+	23038942	23039874	932	-	-	GO:0003700: sequence-specific DNA binding transcription factor activity;GO:0043565:sequence-specific DNA binding	GO:0005634:nucleus

ppe-miR395e	18789967	PRUPE_ppa010227mg	NCBI_Assembly:GCF_000346465.:	NW_006760385.1	-	31644107	31647916	3809	-	GO:0055085:transmembrane transport	-	GO:0005741:mitochondrial outer membrane
ppe-miR395e	18790024	PRUPE_ppa009071mg	NCBI_Assembly:GCF_000346465.:	NW_006760385.1	+	38368466	38369389	923	-	-	-	-
ppe-miR395e	18790186	PRUPE_ppa000679mg	NCBI_Assembly:GCF_000346465.:	NW_006760385.1	-	22816990	22822324	5334	pper04075:Plant hormone signal transduction	GO:0000303:response to superoxide;GO:0006635:fatty acid beta-oxidation;GO:0006970:response to osmotic stress;GO:0007623:circadian rhythm;GO:0008219:cell death;GO:0009863:salicylic acid mediated signaling pathway;GO:0009873:ethylene-activated signaling pathway;GO:0009909:regulation of flower development;GO:0010029:regulation of seed germination;GO:0010087:phloem or xylem histogenesis;GO:0010150:leaf senescence;GO:0010271:regulation of chlorophyll	GO:0000155:phosphorelay sensor kinase activity;GO:0005034:osmosensor activity;GO:0009884:cytokinin receptor activity	GO:0005622:intracellular;GO:0005886:plasma membrane

ppe-miR395e	18790574	PRUPE_ppa015883mg	NCBI_Assembly:GCF_000346465.:	NW_006760385.1	-	34655413	34656502	1089	-	-	GO:0003677: DNA binding;GO:0003682:chromatin binding	-
ppe-miR395e	18790614	PRUPE_ppa026149mg	NCBI_Assembly:GCF_000346465.:	NW_006760385.1	-	781938	783156	1218	-	-	-	-
ppe-miR395e	18790645	PRUPE_ppa003065mg	NCBI_Assembly:GCF_000346465.:	NW_006760385.1	+	21693380	21695571	2191	pper04075:Plant hormone signal transduction	-	-	-
ppe-miR395e	18790656	PRUPE_ppa017915mg	NCBI_Assembly:GCF_000346465.:	NW_006760385.1	+	45967661	45970112	2451	-	-	GO:0004497: monooxygenase activity;GO:0005506:iron ion binding;GO:0016705:oxidoreductase activity, acting on paired donors, with incorporation or reduction of molecular oxygen;GO:0020037:heme binding	-
ppe-miR395e	18790818	PRUPE_ppa018370mg	NCBI_Assembly:GCF_000346465.:	NW_006760385.1	+	210547	212298	1751	-	-	-	-
ppe-miR395e	18790851	PRUPE_ppa013479mg	NCBI_Assembly:GCF_000346465.:	NW_006760385.1	+	8564114	8566744	2630	-	GO:0030244: cellulose biosynthetic process;GO:0048193:Golgi vesicle transport	-	GO:0072546:ER membrane protein complex

ppe-miR395e	18790894	PRUPE_ppa009591mg	NCBI_Assembly:GCF_000346465.:	NW_006760385.1	+	1013580	1016203	2623	pper01100:Metabolic pathways;pper01110:Biosynthesis of secondary metabolites;pper01230:Biosynthesis of amino acids;pper00340:Histidine metabolism	GO:0000105: histidine biosynthetic process	GO:0004424:imidazoleglycerol-phosphate dehydratase activity	-
ppe-miR395e	18790984	PRUPE_ppa002895mg	NCBI_Assembly:GCF_000346465.:	NW_006760385.1	+	25192376	25195005	2629	-	GO:0009627: systemic acquired resistance;GO:0031347:regulation of defense response	GO:0004674: protein serine/threonine kinase activity;GO:0005524:ATP binding	GO:0005886:plasma membrane
ppe-miR395e	18791020	PRUPE_ppa011087mg	NCBI_Assembly:GCF_000346465.:	NW_006760385.1	+	2842401	2843683	1282	pper00480:Glutathione metabolism	-	-	-
ppe-miR395e	18791026	PRUPE_ppa004044mg	NCBI_Assembly:GCF_000346465.:	NW_006760385.1	+	25854449	25858687	4238	-	GO:0055085:transmembrane transport	GO:0005215:transporter activity	GO:0016020:membrane
ppe-miR395e	18791150	PRUPE_ppa000982mg	NCBI_Assembly:GCF_000346465.:	NW_006760385.1	-	30749960	30753856	3896	-	-	GO:0004674: protein serine/threonine kinase activity;GO:0005524:ATP binding	GO:0016021:integral component of membrane
ppe-miR395e	18791220	PRUPE_ppa004917mg	NCBI_Assembly:GCF_000346465.:	NW_006760385.1	-	34796409	34798210	1801	-	-	-	-
ppe-miR395e	18791239	PRUPE_ppa004764mg	NCBI_Assembly:GCF_000346465.:	NW_006760385.1	+	2856939	2862040	5101	-	-	GO:0008270: zinc ion binding	-

ppe-miR395e	18791456	PRUPE_ppa000165mg	NCBI_Assembly:GCF_000346465.:	NW_006760385.1	-	34484342	34494286	9944	-	GO:0006396: RNA processing	GO:0003723: RNA binding;GO:0004525:ribonuclease III activity;GO:0005524:ATP binding	-
ppe-miR395e	18791492	PRUPE_ppa017671mg	NCBI_Assembly:GCF_000346465.:	NW_006760385.1	+	44542032	44545042	3010	-	-	-	-

ppe-miR395e	18791508	PRUPE_ppa021958mg	NCBI_Assembly:GCF_000346465.:	NW_006760385.1	+	37725820	37733439	7619	-	GO:0007062: sister chromatid cohesion;GO:0009640:photomorphogenesis;GO:0009845:seed germination;GO:0009880:embryonic pattern specification;GO:0009908:flower development;GO:0009909:regulation of flower development;GO:0010072:primary shoot apical meristem specification;GO:0010162:seed dormancy process;GO:0010182:sugar mediated signaling pathway;GO:0010228:vegetative to reproductive phase transition of meristem;GO:	-	GO:0080008:Cul4-RING E3 ubiquitin ligase complex
ppe-miR395e	18791568	PRUPE_ppa017473mg	NCBI_Assembly:GCF_000346465.:	NW_006760385.1	-	39283009	39284696	1687	-	-	-	-
ppe-miR395e	18791578	PRUPE_ppa011250mg	NCBI_Assembly:GCF_000346465.:	NW_006760385.1	+	41645943	41648408	2465	-	-	-	-
ppe-miR395e	18791650	PRUPE_ppa016412mg	NCBI_Assembly:GCF_000346465.:	NW_006760385.1	+	3056759	3057893	1134	-	-	-	-
ppe-miR395e	18791727	PRUPE_ppa010221mg	NCBI_Assembly:GCF_000346465.:	NW_006760385.1	+	35002426	35003750	1324	-	GO:0015996: chlorophyll catabolic process	-	-

ppe-miR395e	18791779	PRUPE_ppa020266mg	NCBI_Assembly:GCF_000346465.:	NW_006760385.1	-	27391378	27393500	2122	-	GO:0006457: protein folding	GO:0001106: RNA polymerase II transcription corepressor activity	GO:0016272:prefoldin complex
ppe-miR395e	18791890	PRUPE_ppb008326mg	NCBI_Assembly:GCF_000346465.:	NW_006760385.1	-	368596	369949	1353	-	-	-	-
ppe-miR395e	18791900	PRUPE_ppa006841mg	NCBI_Assembly:GCF_000346465.:	NW_006760385.1	-	8141239	8143517	2278	pper00270:Cysteine and methionine metabolism;pper01100:Metabolic pathways;pper01110:Biosynthesis of secondary metabolites;pper01230:Biosynthesis of amino acids	GO:0006556: S-adenosylmethionine biosynthetic process;GO:0006730:one-carbon metabolic process	GO:0004478: methionine adenosyltransferase activity;GO:0005524:ATP binding;GO:0046872:metal ion binding	-

ppe-miR395e	18791945	PRUPE_ppa011862mg	NCBI_Assembly:GCF_000346465.:	NW_006760385.1	-	27526854	27527908	1054	-	GO:0000023:maltose metabolic process;GO:0010103:stomatal complex morphogenesis;GO:0019252:starch biosynthetic process;GO:0019288:isopentenyl diphosphate biosynthetic process, methylerythritol 4-phosphate pathway;GO:0045893:positive regulation of transcription, DNA-templated	-	-
ppe-miR395e	18791971	PRUPE_ppa007249mg	NCBI_Assembly:GCF_000346465.:	NW_006760385.1	-	10528540	10530578	2038	-	-	GO:0004672:protein kinase activity;GO:0005524:ATP binding	-
ppe-miR395e	18792000	PRUPE_ppa012735mg	NCBI_Assembly:GCF_000346465.:	NW_006760385.1	-	2458167	2460388	2221	-	-	-	-
ppe-miR395e	18792055	PRUPE_ppa003585mg	NCBI_Assembly:GCF_000346465.:	NW_006760385.1	+	41165262	41168730	3468	-	GO:0006400:tRNA modification;GO:0006897:endocytosis	GO:0003924:GTPase activity;GO:0005525:GTP binding	GO:0005622:intracellular

ppe-miR395e	18792059	PRUPE_ppa006099mg	NCBI_Assembly:GCF_000346465.:	NW_006760385.1	-	24452651	24456565	3914	-	GO:0009825:multidimensional cell growth;GO:0010090:trichome morphogenesis;GO:0034314:Arp2/3 complex-mediated actin nucleation	GO:0005524:ATP binding	GO:0005829:cytosol;GO:0005885:Arp2/3 protein complex
ppe-miR395e	18792274	PRUPE_ppa010941mg	NCBI_Assembly:GCF_000346465.:	NW_006760385.1	+	1473042	1476175	3133	pper00270:Cysteine and methionine metabolism;pper01100:Metabolic pathways	GO:0019509:L-methionine biosynthetic process from methylthioadenosine	GO:0005506:iron ion binding;GO:0010309:acireductone dioxygenase [iron(II)-requiring] activity	GO:0005634:nucleus;GO:0005737:cytoplasm
ppe-miR395e	18792276	PRUPE_ppa014400mg	NCBI_Assembly:GCF_000346465.:	NW_006760385.1	-	12224759	12225723	964	-	-	-	-
ppe-miR395e	18792316	PRUPE_ppa015102mg	NCBI_Assembly:GCF_000346465.:	NW_006760385.1	+	25513844	25515951	2107	-	-	-	-
ppe-miR395e	18792928	PRUPE_ppa026794mg	NCBI_Assembly:GCF_000346465.:	NW_006760385.1	+	28762074	28762671	597	-	-	GO:0016787:hydrolase activity	-
ppe-miR395e	18792941	PRUPE_ppa022492mg	NCBI_Assembly:GCF_000346465.:	NW_006760385.1	-	46742027	46743934	1907	-	-	GO:0005215:transporter activity	GO:0016021:integral component of membrane
ppe-miR395e	18792954	PRUPE_ppa025631mg	NCBI_Assembly:GCF_000346465.:	NW_006760385.1	+	9291920	9293738	1818	pper01100:Metabolic pathways;pper00500:Starch and sucrose metabolism;pper00040:Pentose and glucuronate interconversions	GO:0042545:cell wall modification;GO:0045490:pectin catabolic process	GO:0004857:enzyme inhibitor activity;GO:0030599:pectinesterase activity;GO:0045330:aspartyl esterase activity	GO:0005618:cell wall

ppe-miR395e	18793102	PRUPE_ppa017542mg	NCBI_Assembly:GCF_000346465.:	NW_006760385.1	+	3427794	3428324	530	-	GO:0006351:transcription, DNA-templated;GO:0006355:regulation of transcription, DNA-templated	GO:0003677: DNA binding	GO:0005634:nucleus
ppe-miR395e	18793230	PRUPE_ppb018168mg	NCBI_Assembly:GCF_000346465.:	NW_006760385.1	+	35854553	35856821	2268	-	-	GO:0005506:iron ion binding;GO:0016705:oxidoreductase activity, acting on paired donors, with incorporation or reduction of molecular oxygen;GO:0020037:heme binding	-
ppe-miR395e	18793318	PRUPE_ppa021028mg	NCBI_Assembly:GCF_000346465.:	NW_006760385.1	+	37376898	37379922	3024	-	GO:0048527:lateral root development; GO:0080147:root hair cell development	GO:0005459: UDP-galactose transmembrane transporter activity;GO:0005460:UDP-glucose transmembrane transporter activity	GO:0005794:Golgi apparatus;GO:0016021:integral component of membrane
ppe-miR395e	18793325	PRUPE_ppa005844mg	NCBI_Assembly:GCF_000346465.:	NW_006760385.1	-	40349992	40356435	6443	-	-	-	-
ppe-miR395e	18793498	PRUPE_ppa002971mg	NCBI_Assembly:GCF_000346465.:	NW_006760385.1	-	27002959	27007696	4737	-	-	GO:0008168: methyltransferase activity	GO:0005768:endosome;GO:0005802:trans-Golgi network
ppe-miR395e	18793534	PRUPE_ppa015312mg	NCBI_Assembly:GCF_000346465.:	NW_006760385.1	-	41522175	41528153	5978	-	-	-	-
ppe-miR395e	18793620	PRUPE_ppa011867mg	NCBI_Assembly:GCF_000346465.:	NW_006760385.1	+	11047435	11048013	578	-	-	-	-

ppe-miR395e	18793896	PRUPE_ppa010822mg	NCBI_Assembly:GCF_000346465.:	NW_006760385.1	-	46344191	46351743	7552	-	GO:0006351:transcription, DNA-templated	GO:0003677:DNA binding;GO:0003700:sequence-specific DNA binding transcription factor activity	GO:0005634:nucleus
ppe-miR395e	18793970	PRUPE_ppa022249mg	NCBI_Assembly:GCF_000346465.:	NW_006760385.1	-	34321536	34322132	596	-	-	-	-
ppe-miR395e	18794040	PRUPE_ppa006856mg	NCBI_Assembly:GCF_000346465.:	NW_006760385.1	-	29731860	29734304	2444	-	GO:0051726:regulation of cell cycle	-	-
ppe-miR397	18766056	PRUPE_ppa021871mg	NCBI_Assembly:GCF_000346465.:	NW_006760186.1	-	77484	77858	374	-	-	-	-
ppe-miR397	18767133	PRUPE_ppa025105mg	NCBI_Assembly:GCF_000346465.:	NW_006760194.1	-	15176977	15178824	1847	-	-	-	-
ppe-miR397	18767238	PRUPE_ppa000339mg	NCBI_Assembly:GCF_000346465.:	NW_006760194.1	+	8519548	8523359	3811	pper03040:Spliceosome	-	-	GO:0009507:chloroplast
ppe-miR397	18767361	PRUPE_ppa022741mg	NCBI_Assembly:GCF_000346465.:	NW_006760194.1	+	18362540	18363295	755	-	-	-	-
ppe-miR397	18767545	PRUPE_ppa016630mg	NCBI_Assembly:GCF_000346465.:	NW_006760194.1	+	17104774	17110599	5825	-	GO:0007165:signal transduction	GO:0043531:ADP binding	-
ppe-miR397	18767709	PRUPE_ppa010834mg	NCBI_Assembly:GCF_000346465.:	NW_006760194.1	+	14537748	14541484	3736	-	GO:0042744:hydrogen peroxide catabolic process;GO:0045454:cell redox homeostasis	-	GO:0005623:cell
ppe-miR397	18767730	PRUPE_ppb011856mg	NCBI_Assembly:GCF_000346465.:	NW_006760194.1	+	13457523	13458365	842	-	-	-	-
ppe-miR397	18767883	PRUPE_ppa027155mg	NCBI_Assembly:GCF_000346465.:	NW_006760194.1	+	3034310	3039276	4966	-	GO:0007165:signal transduction	GO:0043531:ADP binding	-
ppe-miR397	18767905	PRUPE_ppa019628mg	NCBI_Assembly:GCF_000346465.:	NW_006760194.1	-	3045239	3049250	4011	-	GO:0007165:signal transduction	GO:0043531:ADP binding	-
ppe-miR397	18768189	PRUPE_ppa011562mg	NCBI_Assembly:GCF_000346465.:	NW_006760194.1	+	19735177	19736005	828	-	-	GO:0003676:nucleic acid binding;GO:0008270:zinc ion binding	-

ppe-miR397	18768360	PRUPE_ppa011362mg	NCBI_Assembly:GCF_000346465.:	NW_006760194.1	+	17668381	17669502	1121	pper04140:Regulation of autophagy	GO:0006914: autophagy	GO:0004839: ubiquitin activating enzyme activity	-
ppe-miR397	18768383	PRUPE_ppa021879mg	NCBI_Assembly:GCF_000346465.:	NW_006760194.1	-	3007122	3009387	2265	-	-	GO:0003676: nucleic acid binding	-
ppe-miR397	18768409	PRUPE_ppa006838mg	NCBI_Assembly:GCF_000346465.:	NW_006760194.1	+	15774477	15776076	1599	-	-	-	-
ppe-miR397	18768440	PRUPE_ppb018196mg	NCBI_Assembly:GCF_000346465.:	NW_006760194.1	+	850915	854177	3262	-	-	GO:0003676: nucleic acid binding	-
ppe-miR397	18768541	PRUPE_ppa019393mg	NCBI_Assembly:GCF_000346465.:	NW_006760194.1	-	11170897	11171270	373	-	GO:0006810:transport	-	GO:0016020:membrane
ppe-miR397	18768718	PRUPE_ppa009920mg	NCBI_Assembly:GCF_000346465.:	NW_006760194.1	+	10757487	10760361	2874	-	GO:0006355:regulation of transcription, DNA-templated;GO:0010228:vegetative to reproductive phase transition of meristem;GO:0048510:regulation of timing of transition from vegetative to reproductive phase;GO:0090239:regulation of histone H4 acetylation	-	-
ppe-miR397	18769001	PRUPE_ppa018060mg	NCBI_Assembly:GCF_000346465.:	NW_006760194.1	+	3006180	3013775	7595	-	GO:0007165: signal transduction	GO:0043531: ADP binding	-

ppe-miR397	18769129	PRUPE_ppa012191mg	NCBI_Assembly:GCF_000346465.:	NW_006760201.1	-	21147500	21150651	3151	pper03060:Protein export	GO:0006465: signal peptide processing	GO:0008233: peptidase activity	GO:0005774:vacuolar membrane;GO:0005783:endoplasmic reticulum;GO:0005886:plasma membrane;GO:0016021:integral component of membrane
ppe-miR397	18769295	PRUPE_ppa005779mg	NCBI_Assembly:GCF_000346465.:	NW_006760201.1	-	14710426	14714250	3824	pper01100:Metabolic pathways;pper01110:Biosynthesis of secondary metabolites;pper01230:Biosynthesis of amino acids;pper01200:Carbon metabolism;pper00010:Glycolysis / Gluconeogenesis;pper03018:RNA degradation	GO:0006096: glycolytic process	GO:0000287: magnesium ion binding;GO:0004634:phosphopyruvate hydratase activity	GO:0000015:phosphopyruvate hydratase complex
ppe-miR397	18769366	PRUPE_ppa005688mg	NCBI_Assembly:GCF_000346465.:	NW_006760201.1	-	21756434	21759114	2680	-	-	GO:0022891: substrate-specific transmembrane transporter activity	GO:0005886:plasma membrane;GO:0016021:integral component of membrane
ppe-miR397	18769517	PRUPE_ppa025803mg	NCBI_Assembly:GCF_000346465.:	NW_006760201.1	+	4474905	4476323	1418	-	GO:0009451: RNA modification	-	-
ppe-miR397	18769598	PRUPE_ppa025374mg	NCBI_Assembly:GCF_000346465.:	NW_006760201.1	+	7784939	7787078	2139	-	-	GO:0022857:transmembrane transporter activity	GO:0016021:integral component of membrane

ppe-miR397	18769841	PRUPE_ppa014409mg	NCBI_Assembly:GCF_000346465.:	NW_006760201.1	-	19001745	19002367	622	-	-	-	-
ppe-miR397	18770019	PRUPE_ppa009354mg	NCBI_Assembly:GCF_000346465.:	NW_006760201.1	+	21805612	21806590	978	-	GO:0006457: protein folding	GO:0003755: peptidyl-prolyl cis-trans isomerase activity	GO:0009507:chloroplast;GO:0031977:thylakoid lumen
ppe-miR397	18770042	PRUPE_ppa002266mg	NCBI_Assembly:GCF_000346465.:	NW_006760201.1	+	18396737	18399039	2302	pper00310:Lysine degradation	-	GO:0008270: zinc ion binding;GO:0018024:histone-lysine N-methyltransferase activity	GO:0005634:nucleus;GO:0005694:chromosome
ppe-miR397	18770388	PRUPE_ppa016884mg	NCBI_Assembly:GCF_000346465.:	NW_006760201.1	-	19808201	19810591	2390	-	GO:0009909:regulation of flower development; GO:0016570: histone modification;GO:0048449:floral organ formation	-	-
ppe-miR397	18770435	PRUPE_ppa011596mg	NCBI_Assembly:GCF_000346465.:	NW_006760201.1	+	18173457	18178288	4831	-	-	-	-
ppe-miR397	18771591	PRUPE_ppa026309mg	NCBI_Assembly:GCF_000346465.:	NW_006760201.1	+	11511118	11513340	2222	-	-	GO:0003723: RNA binding	-
ppe-miR397	18771853	PRUPE_ppa018017mg	NCBI_Assembly:GCF_000346465.:	NW_006760201.1	+	3642913	3643379	466	-	-	GO:0009055: electron carrier activity	-
ppe-miR397	18771875	PRUPE_ppa020978mg	NCBI_Assembly:GCF_000346465.:	NW_006760201.1	+	21493602	21498807	5205	-	-	-	-
ppe-miR397	18772091	PRUPE_ppa022399mg	NCBI_Assembly:GCF_000346465.:	NW_006760208.1	-	22792375	22796107	3732	-	GO:0006810:transport	-	GO:0005886:plasma membrane
ppe-miR397	18772190	PRUPE_ppa017391mg	NCBI_Assembly:GCF_000346465.:	NW_006760208.1	-	20667281	20668381	1100	-	-	GO:0016758:transferase activity, transferring hexosyl groups	GO:0016021:integral component of membrane
ppe-miR397	18772386	PRUPE_ppa009701mg	NCBI_Assembly:GCF_000346465.:	NW_006760208.1	-	25317500	25321749	4249	-	-	GO:0003723: RNA binding	-

ppe-miR397	18772440	PRUPE_ppa007952mg	NCBI_Assembly:GCF_000346465.:	NW_006760208.1	-	27646630	27648606	1976	-	-	GO:0016491: oxidoreductase activity	-
ppe-miR397	18772448	PRUPE_ppa008584mg	NCBI_Assembly:GCF_000346465.:	NW_006760208.1	+	24230296	24231786	1490	-	GO:0006457: protein folding	-	-
ppe-miR397	18772459	PRUPE_ppa008150mg	NCBI_Assembly:GCF_000346465.:	NW_006760208.1	-	3010744	3012233	1489	-	-	GO:0008270: zinc ion binding	-
ppe-miR397	18772851	PRUPE_ppa003306mg	NCBI_Assembly:GCF_000346465.:	NW_006760208.1	-	6474068	6475828	1760	-	-	-	-

ppe-miR397	18773046	PRUPE_ppa012933mg	NCBI_Assembly:GCF_000346465.:	NW_006760208.1	-	25215189	25217285	2096	pper04120:Ubiquitin mediated proteolysis;pper04141:Protein processing in endoplasmic reticulum	GO:0006301:postreplication repair;GO:0006635:fatty acid beta-oxidation;GO:0016558:protein import into peroxisome matrix;GO:0042023:DNA endoreduplication;GO:0043161:proteasome-mediated ubiquitin-dependent protein catabolic process;GO:0043248:proteasome assembly;GO:0048193:Golgi vesicle transport;GO:0051510:regulation of unidimensional cell growth;GO:0051788:response to misfolded protein	GO:0004842:ubiquitin-protein transferase activity;GO:0016874:ligase activity	GO:0005622:intracellular;GO:0005886:plasma membrane
ppe-miR397	18773070	PRUPE_ppa016080mg	NCBI_Assembly:GCF_000346465.:	NW_006760208.1	-	4416056	4416361	305	-	-	-	-
ppe-miR397	18773156	PRUPE_ppa013534mg	NCBI_Assembly:GCF_000346465.:	NW_006760208.1	+	7622521	7628016	5495	-	-	-	-
ppe-miR397	18773180	PRUPE_ppb019035mg	NCBI_Assembly:GCF_000346465.:	NW_006760208.1	+	19034600	19035829	1229	-	-	GO:0003676:nucleic acid binding;GO:0008270:zinc ion binding	-
ppe-miR397	18773865	PRUPE_ppa016955mg	NCBI_Assembly:GCF_000346465.:	NW_006760208.1	+	11055984	11057942	1958	-	-	-	-

ppe-miR397	18774102	PRUPE_ppa012560mg	NCBI_Assembly:GCF_000346465.:	NW_006760208.1	-	23526651	23527870	1219	-	GO:0006950:response to stress	-	GO:0005886:plasma membrane;GO:0009506:plasmodesma
ppe-miR397	18774183	PRUPE_ppa022795mg	NCBI_Assembly:GCF_000346465.:	NW_006760208.1	+	1699336	1701513	2177	-	-	GO:0016758:transferase activity, transferring hexosyl groups	-
ppe-miR397	18774312	PRUPE_ppa007335mg	NCBI_Assembly:GCF_000346465.:	NW_006760208.1	-	1071849	1073675	1826	-	-	GO:0008270: zinc ion binding	-
ppe-miR397	18774587	PRUPE_ppa026045mg	NCBI_Assembly:GCF_000346465.:	NW_006760208.1	-	5182423	5183394	971	-	-	-	-
ppe-miR397	18774759	PRUPE_ppa020750mg	NCBI_Assembly:GCF_000346465.:	NW_006760208.1	-	829829	834001	4172	-	-	-	-
ppe-miR397	18775136	PRUPE_ppa013590mg	NCBI_Assembly:GCF_000346465.:	NW_006760208.1	-	19875656	19876141	485	-	-	-	-
ppe-miR397	18775179	PRUPE_ppa009345mg	NCBI_Assembly:GCF_000346465.:	NW_006760208.1	-	23489190	23490214	1024	-	-	-	-
ppe-miR397	18775189	PRUPE_ppa021558mg	NCBI_Assembly:GCF_000346465.:	NW_006760208.1	-	21176172	21176690	518	-	-	GO:0046872: metal ion binding	-
ppe-miR397	18775206	PRUPE_ppa003454mg	NCBI_Assembly:GCF_000346465.:	NW_006760208.1	-	21136282	21139673	3391	-	-	-	-
ppe-miR397	18775207	PRUPE_ppa014762mg	NCBI_Assembly:GCF_000346465.:	NW_006760208.1	-	20521663	20522508	845	-	-	GO:0016491: oxidoreductase activity	-
ppe-miR397	18775232	PRUPE_ppa024349mg	NCBI_Assembly:GCF_000346465.:	NW_006760208.1	-	23795718	23796497	779	pper01100:Metabolic pathways;pper01110:Biosynthesis of secondary metabolites;pper00905:Brassinosteroid biosynthesis	GO:0008202: steroid metabolic process	GO:0003865: 3-oxo-5-alpha-steroid 4-dehydrogenase activity;GO:0009917:sterol 5-alpha reductase activity;GO:0050213:progesterone 5-alpha-reductase activity	GO:0005737:cytoplasm;GO:0016021:integral component of membrane

ppe-miR397	18775280	PRUPE_ppa012595mg	NCBI_Assembly:GCF_000346465.:	NW_006760208.1	+	27087670	27089498	1828	pper04075:Plant hormone signal transduction	GO:0000160:phosphorelay signal transduction system	GO:0004871:signal transducer activity	GO:0005622:intracellular
ppe-miR397	18775916	PRUPE_ppa004038mg	NCBI_Assembly:GCF_000346465.:	NW_006760212.1	-	10874365	10877018	2653	pper01100:Metabolic pathways;pper00230:Purine metabolism	GO:0001510:RNA methylation;GO:0006177:GMP biosynthetic process;GO:0006529:asparagine biosynthetic process;GO:0006606:protein import into nucleus;GO:0009220:pyrimidine ribonucleotide biosynthetic process	GO:0003922:GMP synthase (glutamine-hydrolyzing) activity;GO:0004066:asparagine synthase (glutamine-hydrolyzing) activity;GO:0005524:ATP binding;GO:0016462:pyrophosphatase activity	GO:0005829:cytosol
ppe-miR397	18775969	PRUPE_ppa026105mg	NCBI_Assembly:GCF_000346465.:	NW_006760212.1	+	13542339	13545981	3642	-	GO:0000724:double-strand break repair via homologous recombination;GO:0007126:meiotic nuclear division;GO:0048451:petal formation;GO:0048453:sepal formation	-	-
ppe-miR397	18775978	PRUPE_ppa000184mg	NCBI_Assembly:GCF_000346465.:	NW_006760212.1	-	11283418	11290657	7239	-	GO:0006486:protein glycosylation	-	GO:0005829:cytosol
ppe-miR397	18776013	PRUPE_ppa018792mg	NCBI_Assembly:GCF_000346465.:	NW_006760212.1	+	13454809	13456284	1475	-	GO:0008643:carbohydrate transport	-	GO:0016021:integral component of membrane

ppe-miR397	18776029	PRUPE_ppa023181mg	NCBI_Assembly:GCF_000346465.:	NW_006760212.1	-	11969044	11969388	344	-	GO:0006869:lipid transport	GO:0008289:lipid binding	-
ppe-miR397	18776515	PRUPE_ppa018079mg	NCBI_Assembly:GCF_000346465.:	NW_006760212.1	-	11433341	11433976	635	-	-	-	-
ppe-miR397	18776524	PRUPE_ppa024616mg	NCBI_Assembly:GCF_000346465.:	NW_006760212.1	-	16985632	16986006	374	-	-	-	-
ppe-miR397	18776567	PRUPE_ppa010976mg	NCBI_Assembly:GCF_000346465.:	NW_006760212.1	-	1490048	1490874	826	-	GO:0006886:intracellular protein transport	GO:0015450:P-P-bond-hydrolysis-driven protein transmembrane transporter activity	GO:0005744:mitochondrial inner membrane presequence translocase complex;GO:0016021:integral component of membrane
ppe-miR397	18776577	PRUPE_ppa018672mg	NCBI_Assembly:GCF_000346465.:	NW_006760212.1	-	5783307	5784402	1095	pper00562:Inositol phosphate metabolism;pper00053:Ascorbate and aldarate metabolism	GO:0019310:inositol catabolic process	GO:0005506:iron ion binding;GO:0050113:inositol oxygenase activity	GO:0005737:cytoplasm
ppe-miR397	18776721	PRUPE_ppa012293mg	NCBI_Assembly:GCF_000346465.:	NW_006760212.1	+	4427337	4428083	746	-	-	-	GO:0009507:chloroplast
ppe-miR397	18776858	PRUPE_ppa015224mg	NCBI_Assembly:GCF_000346465.:	NW_006760212.1	+	9655936	9656570	634	-	-	GO:0009055:electron carrier activity	-
ppe-miR397	18777026	PRUPE_ppa019480mg	NCBI_Assembly:GCF_000346465.:	NW_006760212.1	+	10401689	10402762	1073	-	-	-	-
ppe-miR397	18777320	PRUPE_ppa008299mg	NCBI_Assembly:GCF_000346465.:	NW_006760212.1	+	12382291	12384979	2688	-	-	GO:0016491:oxidoreductase activity	-

ppe-miR397	18777434	PRUPE_ppa026791mg	NCBI_Assembly:GCF_000346465.:	NW_006760212.1	-	14697073	14698853	1780	-	GO:0006863: purine nucleobase transport;GO: 0009553:embryo sac development; GO:0010183: pollen tube guidance;GO: 0045697:regulation of synergid differentiation	GO:0003677: DNA binding;GO:0003682:chromatin binding;GO:0003700:sequence-specific DNA binding transcription factor activity	-
ppe-miR397	18777460	PRUPE_ppa026659mg	NCBI_Assembly:GCF_000346465.:	NW_006760212.1	+	15744371	15745373	1002	pper00480:Glutathione metabolism	-	-	-
ppe-miR397	18777597	PRUPE_ppa020229mg	NCBI_Assembly:GCF_000346465.:	NW_006760212.1	-	5792226	5793321	1095	pper00562:Inositol phosphate metabolism;pper00053:Ascorbate and aldarate metabolism	GO:0019310:inositol catabolic process	GO:0005506:iron ion binding;GO:0050113:inositol oxygenase activity	GO:0005737:cytoplasm
ppe-miR397	18777767	PRUPE_ppa012101mg	NCBI_Assembly:GCF_000346465.:	NW_006760212.1	-	5182606	5183329	723	-	-	GO:0004857: enzyme inhibitor activity;GO:0030599:pectinesterase activity	-
ppe-miR397	18777856	PRUPE_ppa020374mg	NCBI_Assembly:GCF_000346465.:	NW_006760212.1	-	2823801	2824180	379	-	-	GO:0000166: nucleotide binding;GO:0003676:nucleic acid binding	-
ppe-miR397	18778121	PRUPE_ppa025907mg	NCBI_Assembly:GCF_000346465.:	NW_006760212.1	+	10484479	10486684	2205	-	-	-	-
ppe-miR397	18778289	PRUPE_ppa011051mg	NCBI_Assembly:GCF_000346465.:	NW_006760220.1	+	29560033	29561786	1753	-	-	-	-
ppe-miR397	18778511	PRUPE_ppa013202mg	NCBI_Assembly:GCF_000346465.:	NW_006760220.1	+	12467624	12469210	1586	-	-	GO:0003677: DNA binding	GO:0000786:nucleosome;GO:0005634:nucleus
ppe-miR397	18778558	PRUPE_ppa026155mg	NCBI_Assembly:GCF_000346465.:	NW_006760220.1	-	13297957	13298682	725	-	-	-	-

ppe-miR397	18778572	PRUPE_ppa000747mg	NCBI_Assembly:GCF_000346465.:	NW_006760220.1	-	189623	195322	5699	-	-	-	-
ppe-miR397	18778852	PRUPE_ppa023295mg	NCBI_Assembly:GCF_000346465.:	NW_006760220.1	+	10610909	10611784	875	-	-	GO:0005215:transporter activity	GO:0016021:integral component of membrane
ppe-miR397	18778953	PRUPE_ppa016086mg	NCBI_Assembly:GCF_000346465.:	NW_006760220.1	-	27723366	27725439	2073	-	-	GO:0008270: zinc ion binding	-
ppe-miR397	18778954	PRUPE_ppa003033mg	NCBI_Assembly:GCF_000346465.:	NW_006760220.1	-	23714765	23722063	7298	-	-	-	-
ppe-miR397	18779102	PRUPE_ppa007390mg	NCBI_Assembly:GCF_000346465.:	NW_006760220.1	+	8454390	8458372	3982	pper01100:Metabolic pathways;pper01110:Biosynthesis of secondary metabolites;pper00630:Glyoxylate and dicarboxylate metabolism;pper04146:Peroxisome	-	GO:0010181: FMN binding;GO:0016491:oxidoreductase activity	-
ppe-miR397	18779576	PRUPE_ppa020840mg	NCBI_Assembly:GCF_000346465.:	NW_006760220.1	-	25063343	25067182	3839	-	GO:0015074: DNA integration	GO:0003676: nucleic acid binding	-

ppe-miR397	18779711	PRUPE_ppa021601mg	NCBI_Assembly:GCF_000346465.:	NW_006760220.1	-	26272328	26275757	3429	pper01100:Metabolic pathways;pper00230:Purine metabolism;pper00240:Pyrimidine metabolism;pper03030:DNA replication;pper03420:Nucleotide excision repair;pper03430:Mismatch repair;pper03440:Homologous recombination;pper03410:Base excision repair	-	GO:0000166: nucleotide binding;GO:0003677:DNA binding	-
ppe-miR397	18779842	PRUPE_ppa006626mg	NCBI_Assembly:GCF_000346465.:	NW_006760220.1	-	926547	929833	3286	-	GO:0006486: protein glycosylation	GO:0008378: galactosyltransferase activity	GO:0005794:Golgi apparatus;GO:0016021:integral component of membrane
ppe-miR397	18779888	PRUPE_ppa004360mg	NCBI_Assembly:GCF_000346465.:	NW_006760220.1	+	18541069	18545104	4035	-	GO:0019761: glucosinolate biosynthetic process	GO:0015238: drug transmembrane transporter activity;GO:0015297:antiporter activity	GO:0016021:integral component of membrane
ppe-miR397	18780037	PRUPE_ppa016417mg	NCBI_Assembly:GCF_000346465.:	NW_006760220.1	-	8695948	8702926	6978	-	-	-	-

ppe-miR397	18781705	PRUPE_ppa007319mg	NCBI_Assembly:GCF_000346465.:	NW_006760268.1	-	16260549	16264200	3651	-	GO:0007020: microtubule nucleation;GO:0007067:mitotic nuclear division;GO:0031023:microtubule organizing center organization; GO:0051225: spindle assembly	-	-
ppe-miR397	18783013	PRUPE_ppa020470mg	NCBI_Assembly:GCF_000346465.:	NW_006760268.1	+	19107803	19109506	1703	-	-	GO:0016758:transferase activity, transferring hexosyl groups	-
ppe-miR397	18783613	PRUPE_ppa006798mg	NCBI_Assembly:GCF_000346465.:	NW_006760268.1	-	15957988	15960982	2994	-	GO:0030048: actin filament-based movement;GO:0051645:Golgi localization;GO:0051646:mitochondrion localization;GO:0060151:peroxisome localization	-	-

ppe-miR397	18783736	PRUPE_ppa004050mg	NCBI_Assembly:GCF_000346465.:	NW_006760268.1	+	2324057	2325683	1626	pper01100:Metabolic pathways;pper01230:Biosynthesis of amino acids;pper00260:Glycine, serine and threonine metabolism;pper00750:Vitamin B6 metabolism	GO:0006520: cellular amino acid metabolic process	GO:0030170: pyridoxal phosphate binding	-
ppe-miR397	18783739	PRUPE_ppa001641mg	NCBI_Assembly:GCF_000346465.:	NW_006760268.1	+	21903273	21910069	6796	pper01100:Metabolic pathways;pper01110:Biosynthesis of secondary metabolites;pper01200:Carbon metabolism;pper00010:Glycolysis / Gluconeogenesis;pper00620:Pyruvate metabolism;pper00640:Propanoate metabolism	GO:0006094: gluconeogenesis;GO:0007010:cytoskeleton organization; GO:0010498: proteasomal protein catabolic process;GO:0019427:acetyl-CoA biosynthetic process from acetate;GO:0031348:negative regulation of defense response	GO:0003987: acetate-CoA ligase activity;GO:0005524:ATP binding;GO:0016208:AMP binding	GO:0005829:cytosol;GO:0009570:chloroplast stroma
ppe-miR397	18783869	PRUPE_ppa013074mg	NCBI_Assembly:GCF_000346465.:	NW_006760268.1	-	9026891	9029666	2775	-	-	GO:0043565: sequence-specific DNA binding	-

ppe-miR397	18783972	PRUPE_ppa008465mg	NCBI_Assembly:GCF_000346465.:	NW_006760268.1	+	3528765	3531308	2543	pper03013:RNA transport	GO:0015031:protein transport;GO:0051028:mRNA transport;GO:0055085:transmembrane transport	-	GO:0005643:nuclear pore;GO:0031965:nuclear membrane
ppe-miR397	18784195	PRUPE_ppa000231mg	NCBI_Assembly:GCF_000346465.:	NW_006760268.1	+	4755200	4762077	6877	-	-	GO:0005524:ATP binding;GO:0016887:ATPase activity	GO:0016020:membrane
ppe-miR397	18784272	PRUPE_ppb020170mg	NCBI_Assembly:GCF_000346465.:	NW_006760268.1	-	10288266	10290689	2423	-	-	GO:0030247:polysaccharide binding	-
ppe-miR397	18784418	PRUPE_ppa007007mg	NCBI_Assembly:GCF_000346465.:	NW_006760268.1	+	18343501	18345686	2185	-	-	GO:0008270:zinc ion binding	GO:0005622:intracellular
ppe-miR397	18784557	PRUPE_ppa008319mg	NCBI_Assembly:GCF_000346465.:	NW_006760324.1	+	22799376	22801945	2569	pper04130:SNARE interactions in vesicular transport	GO:0006886:intracellular protein transport;GO:0006891:intra-Golgi vesicle-mediated transport;GO:0061025:membrane fusion	-	GO:0009504:cell plate;GO:0016020:membrane;GO:0043231:intracellular membrane-bounded organelle
ppe-miR397	18784604	PRUPE_ppa023956mg	NCBI_Assembly:GCF_000346465.:	NW_006760324.1	-	22623551	22624992	1441	-	-	GO:0016758:transferase activity, transferring hexosyl groups	-
ppe-miR397	18784859	PRUPE_ppa000654mg	NCBI_Assembly:GCF_000346465.:	NW_006760324.1	+	19610286	19614023	3737	-	-	GO:0005524:ATP binding;GO:0016787:hydrolase activity;GO:0046872:metal ion binding	GO:0005783:endoplasmic reticulum;GO:0016021:integral component of membrane

ppe-miR397	18784864	PRUPE_ppa024830mg	NCBI_Assembly:GCF_000346465.:	NW_006760324.1	-	18049020	18052072	3052	-	GO:0009966:regulation of signal transduction	GO:0004674:protein serine/threonine kinase activity;GO:0005524:ATP binding	-
ppe-miR397	18784897	PRUPE_ppa002304mg	NCBI_Assembly:GCF_000346465.:	NW_006760324.1	+	24568306	24573106	4800	-	GO:0001510:RNA methylation;GO:0051604:protein maturation	GO:0003723:RNA binding;GO:0004386:helicase activity;GO:0005524:ATP binding	GO:0005730:nucleolus
ppe-miR397	18784956	PRUPE_ppb021274mg	NCBI_Assembly:GCF_000346465.:	NW_006760324.1	-	12971302	12972082	780	-	-	-	-
ppe-miR397	18785532	PRUPE_ppa008941mg	NCBI_Assembly:GCF_000346465.:	NW_006760324.1	-	26082188	26084321	2133	pper01100:Metabolic pathways;pper01110:Biosynthesis of secondary metabolites;pper01200:Carbon metabolism;pper00030:Pentose phosphate pathway	GO:0002229:defense response to oomycetes;GO:0005975:carbohydrate metabolic process;GO:0006098:pentose-phosphate shunt;GO:0042742:defense response to bacterium;GO:0071461:cellular response to redox state	GO:0017057:6-phosphogluconolactonase activity	GO:0005777:peroxisome;GO:0009570:chloroplast stroma
ppe-miR397	18786224	PRUPE_ppa023710mg	NCBI_Assembly:GCF_000346465.:	NW_006760324.1	+	17983066	17983623	557	-	-	-	-
ppe-miR397	18786604	PRUPE_ppa014834mg	NCBI_Assembly:GCF_000346465.:	NW_006760324.1	-	13255033	13256157	1124	-	GO:0006633:fatty acid biosynthetic process	GO:0016747:transferase activity, transferring acyl groups other than amino-acyl groups	GO:0016020:membrane

ppe-miR397	18786808	PRUPE_ppa001538mg	NCBI_Assembly:GCF_000346465.:	NW_006760324.1	-	22947996	22950548	2552	-	GO:0006612:protein targeting to membrane;GO:0009723:response to ethylene;GO:0009738:abscisic acid-activated signaling pathway;GO:0010363:regulation of plant-type hypersensitive response;GO:0035556:intracellular signal transduction;GO:0043069:negative regulation of programmed cell death	GO:0004674:protein serine/threonine kinase activity;GO:0005524:ATP binding	GO:0005622:intracellular
ppe-miR397	18786810	PRUPE_ppa003988mg	NCBI_Assembly:GCF_000346465.:	NW_006760324.1	+	18237915	18244748	6833	-	-	GO:0004190:aspartic-type endopeptidase activity	GO:0016021:integral component of membrane

ppe-miR397	18786834	PRUPE_ppa012623mg	NCBI_Assembly:GCF_000346465.:	NW_006760324.1	+	25517927	25519712	1785	-	GO:0009073:aromatic amino acid family biosynthetic process;GO:0010207:photosystem II assembly;GO:0016226:iron-sulfur cluster assembly;GO:0045893:positive regulation of transcription, DNA-templated	-	-
ppe-miR397	18786919	PRUPE_ppa001323mg	NCBI_Assembly:GCF_000346465.:	NW_006760324.1	-	16762797	16770148	7351	-	GO:0007018:microtubule-based movement;GO:0048364:root development	GO:0003777:microtubule motor activity;GO:0005524:ATP binding	GO:0005871:kinesin complex;GO:0005874:microtubule
ppe-miR397	18787068	PRUPE_ppa011512mg	NCBI_Assembly:GCF_000346465.:	NW_006760324.1	-	26354089	26355891	1802	pper03010:Ribosome	GO:0006412:translation	GO:0003735:structural constituent of ribosome	GO:0005840:ribosome
ppe-miR397	18787310	PRUPE_ppa007832mg	NCBI_Assembly:GCF_000346465.:	NW_006760324.1	-	24134260	24138004	3744	-	-	-	-
ppe-miR397	18787654	PRUPE_ppa009463mg	NCBI_Assembly:GCF_000346465.:	NW_006760324.1	+	24431661	24434162	2501	-	-	-	GO:0005774:vacuolar membrane;GO:0005794:Golgi apparatus;GO:0009506:plasmodesma
ppe-miR397	18788141	PRUPE_ppa007739mg	NCBI_Assembly:GCF_000346465.:	NW_006760385.1	-	22684613	22687122	2509	-	-	-	-
ppe-miR397	18788236	PRUPE_ppa023469mg	NCBI_Assembly:GCF_000346465.:	NW_006760385.1	-	11263089	11265869	2780	-	-	GO:0016787:hydrolase activity	-
ppe-miR397	18788883	PRUPE_ppa003420mg	NCBI_Assembly:GCF_000346465.:	NW_006760385.1	+	11216968	11224454	7486	-	-	-	-

ppe-miR397	18788950	PRUPE_ppa015770mg	NCBI_Assembly:GCF_000346465.:	NW_006760385.1	-	32363297	32364397	1100	-	-	-	-
ppe-miR397	18788991	PRUPE_ppa025869mg	NCBI_Assembly:GCF_000346465.:	NW_006760385.1	-	41804656	41805631	975	pper01100:Metabolic pathways;pper00430:Taurine and hypotaurine metabolism	-	GO:0047800: cysteamine dioxygenase activity	-
ppe-miR397	18789047	PRUPE_ppa015753mg	NCBI_Assembly:GCF_000346465.:	NW_006760385.1	+	36845238	36846653	1415	-	-	GO:0003700: sequence-specific DNA binding transcription factor activity;GO:0008270:zinc ion binding;GO:0043565:sequence-specific DNA binding	-
ppe-miR397	18789128	PRUPE_ppa003089mg	NCBI_Assembly:GCF_000346465.:	NW_006760385.1	+	26156402	26158338	1936	-	-	GO:0004672: protein kinase activity;GO:0005524:ATP binding	-
ppe-miR397	18789143	PRUPE_ppa015357mg	NCBI_Assembly:GCF_000346465.:	NW_006760385.1	-	41711536	41713126	1590	-	-	GO:0016758:transferase activity, transferring hexosyl groups	-
ppe-miR397	18789201	PRUPE_ppa023103mg	NCBI_Assembly:GCF_000346465.:	NW_006760385.1	-	5337545	5338171	626	-	GO:0006351:transcription, DNA-templated;GO:0006355:regulation of transcription, DNA-templated	GO:0003677: DNA binding	GO:0005634:nucleus
ppe-miR397	18789501	PRUPE_ppa009136mg	NCBI_Assembly:GCF_000346465.:	NW_006760385.1	+	34530537	34532193	1656	-	-	-	-

ppe-miR397	18789869	PRUPE_ppa010885mg	NCBI_Assembly:GCF_000346465.:	NW_006760385.1	-	29363547	29364923	1376	-	GO:0048441: petal development; GO:0048443: stamen development; GO:2000488: positive regulation of brassinosteroid biosynthetic process	GO:0003700: sequence-specific DNA binding transcription factor activity	-
ppe-miR397	18790229	PRUPE_ppa024375mg	NCBI_Assembly:GCF_000346465.:	NW_006760385.1	-	33250625	33251091	466	-	-	GO:0003677: DNA binding;GO:0003682:chromatin binding	GO:0005634:nucleus
ppe-miR397	18790253	PRUPE_ppb022998mg	NCBI_Assembly:GCF_000346465.:	NW_006760385.1	+	776108	778080	1972	-	-	-	-
ppe-miR397	18790629	PRUPE_ppa006302mg	NCBI_Assembly:GCF_000346465.:	NW_006760385.1	-	27762710	27766402	3692	-	-	GO:0015095: magnesium ion transmembrane transporter activity	GO:0016020:membrane
ppe-miR397	18790763	PRUPE_ppa006233mg	NCBI_Assembly:GCF_000346465.:	NW_006760385.1	-	44905019	44908079	3060	-	-	GO:0003746:translation elongation factor activity	-
ppe-miR397	18790766	PRUPE_ppa022821mg	NCBI_Assembly:GCF_000346465.:	NW_006760385.1	-	29520712	29521497	785	-	-	GO:0030247: polysaccharide binding	-
ppe-miR397	18791053	PRUPE_ppa000529mg	NCBI_Assembly:GCF_000346465.:	NW_006760385.1	-	10029358	10035695	6337	-	-	-	GO:0005887:integral component of plasma membrane
ppe-miR397	18791102	PRUPE_ppa022190mg	NCBI_Assembly:GCF_000346465.:	NW_006760385.1	+	28780448	28780926	478	-	-	GO:0016787: hydrolase activity	-
ppe-miR397	18791629	PRUPE_ppa022763mg	NCBI_Assembly:GCF_000346465.:	NW_006760385.1	+	1004683	1011375	6692	-	-	GO:0008270: zinc ion binding	-

ppe-miR397	18792051	PRUPE_ppa1027228mg	NCBI_Assembly:GCF_000346465.:	NW_006760385.1	-	15025159	15025942	783	-	-	-	-
ppe-miR397	18792241	PRUPE_ppa022764mg	NCBI_Assembly:GCF_000346465.:	NW_006760385.1	+	32308658	32312789	4131	-	-	GO:0004252: serine-type endopeptidase activity	GO:0048046:apoplast
ppe-miR397	18792393	PRUPE_ppa000092mg	NCBI_Assembly:GCF_000346465.:	NW_006760385.1	+	44168866	44185959	17093	pper03022:Basal transcription factors	-	GO:0003676: nucleic acid binding;GO:0008270:zinc ion binding	-
ppe-miR397	18792667	PRUPE_ppa006384mg	NCBI_Assembly:GCF_000346465.:	NW_006760385.1	-	29725971	29729415	3444	-	GO:0006260: DNA replication	GO:0003677: DNA binding	GO:0000808:origin recognition complex;GO:0005634:nucleus
ppe-miR397	18792699	PRUPE_ppa024578mg	NCBI_Assembly:GCF_000346465.:	NW_006760385.1	+	32231561	32232215	654	-	-	-	-
ppe-miR397	18793423	PRUPE_ppa003359mg	NCBI_Assembly:GCF_000346465.:	NW_006760385.1	+	44737406	44740663	3257	-	-	GO:0005524: ATP binding;GO:0019136:deoxynucleoside kinase activity	GO:0005634:nucleus
ppe-miR397	18793616	PRUPE_ppa023187mg	NCBI_Assembly:GCF_000346465.:	NW_006760385.1	-	16669356	16670243	887	pper03018:RNA degradation	-	GO:0003676: nucleic acid binding	GO:0005634:nucleus
ppe-miR397	18793774	PRUPE_ppa001963mg	NCBI_Assembly:GCF_000346465.:	NW_006760385.1	+	997016	1000395	3379	-	-	GO:0004672: protein kinase activity;GO:0005524:ATP binding	-
ppe-miR397	18793870	PRUPE_ppa011926mg	NCBI_Assembly:GCF_000346465.:	NW_006760385.1	-	29257143	29260189	3046	-	-	-	-
ppe-miR397	18793980	PRUPE_ppa021517mg	NCBI_Assembly:GCF_000346465.:	NW_006760385.1	-	38177207	38177992	785	-	-	-	-
ppe-miR399a	18765973	PRUPE_ppa009429mg	NCBI_Assembly:GCF_000346465.:	NW_006760186.1	-	601147	607633	6486	-	-	GO:0008270: zinc ion binding	-
ppe-miR399a	18766075	PRUPE_ppa017434mg	NCBI_Assembly:GCF_000346465.:	NW_006760186.1	+	1103593	1106721	3128	-	-	-	-

ppe-miR399a	18766308	PRUPE_ppa006426mg	NCBI_Assembly:GCF_000346465.:	NW_006760194.1	+	18625929	18628209	2280	pper00350:Tyrosine metabolism;pper01100:Metabolic pathways;pper01110:Biosynthesis of secondary metabolites;pper00010:Glycolysis / Gluconeogenesis;pper00071:Fatty acid degradation;pper01220:Degradation of aromatic compounds	-	GO:0008270:zinc ion binding;GO:0016491:oxidoreductase activity	-
ppe-miR399a	18766389	PRUPE_ppa002285mg	NCBI_Assembly:GCF_000346465.:	NW_006760194.1	+	19344238	19351517	7279	pper01100:Metabolic pathways;pper04141:Protein processing in endoplasmic reticulum;pper00510:N-Glycan biosynthesis	GO:0006364:rRNA processing;GO:0006487:protein N-linked glycosylation;GO:0030244:cellulose biosynthetic process	-	GO:0005739:mitochondrion;GO:0005774:vacuolar membrane;GO:0005794:Golgi apparatus;GO:0005886:plasma membrane;GO:0008250:oligosaccharyltransferase complex;GO:0009505:plant-type cell wall;GO:0009506:plasmodesma;GO:0016021:integral component of membrane

ppe-miR399a	18766653	PRUPE_ppa009062mg	NCBI_Assembly:GCF_000346465.:	NW_006760194.1	-	15231741	15235050	3309	-	GO:0006816:calcium ion transport;GO:0007030:Golgi organization;GO:0009651:response to salt stress	GO:0004252:serine-type endopeptidase activity	GO:0005794:Golgi apparatus;GO:0016021:integral component of membrane
ppe-miR399a	18766785	PRUPE_ppa025549mg	NCBI_Assembly:GCF_000346465.:	NW_006760194.1	-	20951099	20952172	1073	-	GO:0006355:regulation of transcription, DNA-templated	-	-
ppe-miR399a	18767096	PRUPE_ppb011560mg	NCBI_Assembly:GCF_000346465.:	NW_006760194.1	-	11598167	11598825	658	-	-	-	-
ppe-miR399a	18767103	PRUPE_ppa019020mg	NCBI_Assembly:GCF_000346465.:	NW_006760194.1	+	18618387	18620939	2552	pper00350:Tyrosine metabolism;pper01100:Metabolic pathways;pper00010:Glycolysis / Gluconeogenesis;pper00071:Fatty acid degradation;pper00592:alpha-Linolenic acid metabolism	-	GO:0008270:zinc ion binding;GO:0016491:oxidoreductase activity	-
ppe-miR399a	18767107	PRUPE_ppa004632mg	NCBI_Assembly:GCF_000346465.:	NW_006760194.1	-	10370539	10373057	2518	-	-	GO:0003676:nucleic acid binding	-
ppe-miR399a	18767210	PRUPE_ppb012289mg	NCBI_Assembly:GCF_000346465.:	NW_006760194.1	-	14020978	14021361	383	-	-	-	-
ppe-miR399a	18767217	PRUPE_ppa020853mg	NCBI_Assembly:GCF_000346465.:	NW_006760194.1	-	13320021	13320245	224	-	GO:0006412:translation	GO:0003735:structural constituent of ribosome;GO:0019843:rRNA binding	GO:0005840:ribosome;GO:0009507:chloroplast
ppe-miR399a	18767266	PRUPE_ppa020872mg	NCBI_Assembly:GCF_000346465.:	NW_006760194.1	+	21164815	21165931	1116	-	GO:0030001:metal ion transport	GO:0046872:metal ion binding	-

ppe-miR399a	18767339	PRUPE_ppa009150mg	NCBI_Assembly:GCF_000346465.:	NW_006760194.1	-	14006728	14007806	1078	-	-	GO:0016787: hydrolase activity	-
ppe-miR399a	18767466	PRUPE_ppa020894mg	NCBI_Assembly:GCF_000346465.:	NW_006760194.1	-	18876698	18879653	2955	-	-	-	-
ppe-miR399a	18767497	PRUPE_ppa006873mg	NCBI_Assembly:GCF_000346465.:	NW_006760194.1	+	15273505	15277643	4138	-	-	GO:0004252: serine-type endopeptidase activity	GO:0009534:chloroplast thylakoid;GO:0009840:chloroplastic endopeptidase Clp complex;GO:0009941:chloroplast envelope
ppe-miR399a	18767571	PRUPE_ppa023089mg	NCBI_Assembly:GCF_000346465.:	NW_006760194.1	-	2192287	2193330	1043	pper04120:Ubiquitin mediated proteolysis	-	-	-
ppe-miR399a	18767596	PRUPE_ppa022586mg	NCBI_Assembly:GCF_000346465.:	NW_006760194.1	+	21108414	21108944	530	-	GO:0006355:regulation of transcription, DNA-templated	GO:0043565: sequence-specific DNA binding	GO:0005634:nucleus
ppe-miR399a	18767790	PRUPE_ppa015171mg	NCBI_Assembly:GCF_000346465.:	NW_006760194.1	+	5615149	5618910	3761	-	-	GO:0016788: hydrolase activity, acting on ester bonds	-
ppe-miR399a	18768048	PRUPE_ppa003473mg	NCBI_Assembly:GCF_000346465.:	NW_006760194.1	+	1567423	1572066	4643	pper01100:Metabolic pathways;pper01110:Biosynthesis of secondary metabolites;pper00970:Aminoacyl-tRNA biosynthesis;pper00860:Porphyrin and chlorophyll metabolism	GO:0006424: glutamyl-tRNA aminoacylation;GO:0007005:mitochondrion organization; GO:0009658: chloroplast organization; GO:0048481: ovule development	GO:0000049:tRNA binding;GO:0004818:glutamate-tRNA ligase activity;GO:0005524:ATP binding	GO:0009570:chloroplast stroma
ppe-miR399a	18768137	PRUPE_ppa003618mg	NCBI_Assembly:GCF_000346465.:	NW_006760194.1	+	18885680	18887637	1957	-	-	-	-
ppe-miR399a	18768269	PRUPE_ppa004599mg	NCBI_Assembly:GCF_000346465.:	NW_006760194.1	+	11518748	11522333	3585	-	-	-	-

ppe-miR399a	18768281	PRUPE_ppa017236mg	NCBI_Assembly:GCF_000346465.:	NW_006760194.1	+	20891901	20892881	980	-	-	-	-
ppe-miR399a	18768284	PRUPE_ppa017290mg	NCBI_Assembly:GCF_000346465.:	NW_006760194.1	+	1273373	1275009	1636	-	-	-	-
ppe-miR399a	18768358	PRUPE_ppa015290mg	NCBI_Assembly:GCF_000346465.:	NW_006760194.1	+	18031923	18033401	1478	-	-	GO:0008270: zinc ion binding;GO:0016491:oxidoreductase activity	-
ppe-miR399a	18768434	PRUPE_ppa007262mg	NCBI_Assembly:GCF_000346465.:	NW_006760194.1	+	17120745	17124625	3880	pper01100:Metabolic pathways;pper00564:Glycerophospholipid metabolism;pper00561:Glycerolipid metabolism	GO:0006301: postreplication repair;GO:0006651:diacylglycerol biosynthetic process;GO:0006655:phosphatidylglycerol biosynthetic process;GO:0019432:triglyceride biosynthetic process	GO:0016746:transferase activity, transferring acyl groups	GO:0005783:endoplasmic reticulum;GO:0005886:plasma membrane
ppe-miR399a	18768449	PRUPE_ppa004626mg	NCBI_Assembly:GCF_000346465.:	NW_006760194.1	+	21326906	21330676	3770	-	-	GO:0003824: catalytic activity;GO:0050662:coenzyme binding	-
ppe-miR399a	18768474	PRUPE_ppa009160mg	NCBI_Assembly:GCF_000346465.:	NW_006760194.1	+	13991484	13992436	952	-	-	GO:0016787: hydrolase activity	-

ppe-miR399a	18768495	PRUPE_ppa003344mg	NCBI_Assembly:GCF_000346465.:	NW_006760194.1	+	20944811	20948669	3858	pper04075:Plant hormone signal transduction	GO:0006511:ubiquitin-dependent protein catabolic process;GO:0007165:signal transduction; GO:0009733:response to auxin;GO:0010103:stomatal complex morphogenesis;GO:0010152:pollen maturation;GO:0010311:lateral root formation;GO:0042752:regulation of circadian rhythm;GO:0048443:stamen development	GO:0000822:inositol hexakisphosphate binding;GO:0004842:ubiquitin-protein transferase activity;GO:0010011:auxin binding	GO:0019005:SCF ubiquitin ligase complex
ppe-miR399a	18768791	PRUPE_ppa023238mg	NCBI_Assembly:GCF_000346465.:	NW_006760194.1	+	5973319	5973759	440	-	-	-	-
ppe-miR399a	18768843	PRUPE_ppa026667mg	NCBI_Assembly:GCF_000346465.:	NW_006760194.1	+	7121654	7123480	1826	-	-	-	-
ppe-miR399a	18768890	PRUPE_ppa026845mg	NCBI_Assembly:GCF_000346465.:	NW_006760194.1	+	21128796	21130033	1237	-	-	-	-
ppe-miR399a	18768929	PRUPE_ppa008027mg	NCBI_Assembly:GCF_000346465.:	NW_006760194.1	-	1775362	1778511	3149	-	-	GO:0004185:serine-type carboxypeptidase activity	-
ppe-miR399a	18768930	PRUPE_ppa026648m2g	NCBI_Assembly:GCF_000346465.:	NW_006760194.1	+	13700091	13701897	1806	-	-	GO:0003677:DNA binding	-
ppe-miR399a	18768970	PRUPE_ppa007244mg	NCBI_Assembly:GCF_000346465.:	NW_006760194.1	+	18013509	18015830	2321	-	-	-	-
ppe-miR399a	18769009	PRUPE_ppa014941mg	NCBI_Assembly:GCF_000346465.:	NW_006760195.1	-	6054	8703	2649	pper03008:Ribosome biogenesis in eukaryotes	-	-	-

ppe-miR399a	18769045	PRUPE_ppa013406mg	NCBI_Assembly:GCF_000346465.:	NW_006760201.1	-	19236557	19237094	537	-	GO:0015979: photosynthesis	-	GO:0009523:photosystem II;GO:0009535: chloroplast thylakoid membrane
ppe-miR399a	18769245	PRUPE_ppa003577mg	NCBI_Assembly:GCF_000346465.:	NW_006760201.1	+	16924748	16926920	2172	-	GO:0046274:lignin catabolic process	GO:0005507: copper ion binding;GO:0052716:hydroquinone:oxygen oxidoreductase activity	GO:0048046:apoplast
ppe-miR399a	18769290	PRUPE_ppa021928mg	NCBI_Assembly:GCF_000346465.:	NW_006760201.1	-	11099194	11099826	632	-	-	-	-
ppe-miR399a	18769345	PRUPE_ppa002566mg	NCBI_Assembly:GCF_000346465.:	NW_006760201.1	+	10469406	10474286	4880	-	-	GO:0004672: protein kinase activity;GO:0005524:ATP binding	GO:0016021:integral component of membrane
ppe-miR399a	18769599	PRUPE_ppa013916mg	NCBI_Assembly:GCF_000346465.:	NW_006760201.1	+	19725099	19726882	1783	-	-	-	-
ppe-miR399a	18769642	PRUPE_ppa003398mg	NCBI_Assembly:GCF_000346465.:	NW_006760201.1	+	21907250	21910147	2897	-	-	-	-
ppe-miR399a	18769765	PRUPE_ppa011922mg	NCBI_Assembly:GCF_000346465.:	NW_006760201.1	+	18179453	18180885	1432	pper01100:Metabolic pathways;pper00230:Purine metabolism;pper00730:Thiamine metabolism	-	GO:0005524: ATP binding;GO:0098519:nucleotide phosphatase activity, acting on free nucleotides	-
ppe-miR399a	18769852	PRUPE_ppa011588mg	NCBI_Assembly:GCF_000346465.:	NW_006760201.1	-	7121138	7125887	4749	-	-	GO:0016787: hydrolase activity	-
ppe-miR399a	18769854	PRUPE_ppa011027mg	NCBI_Assembly:GCF_000346465.:	NW_006760201.1	-	20614754	20616156	1402	-	GO:0000902: cell morphogenesis;GO:0016049:cell growth;GO:0048193:Golgi vesicle transport	-	GO:0005622:intracellular

ppe-miR399a	18770365	PRUPE_ppa006453mg	NCBI_Assembly:GCF_000346465.:	NW_006760201.1	+	21656976	21659541	2565	pper01100:Metabolic pathways;pper00561:Glycerolipid metabolism	GO:0009247:glycolipid biosynthetic process;GO:0030259:lipid glycosylation	GO:0016758:transferase activity, transferring hexosyl groups;GO:0030246:carbohydrate binding	-
ppe-miR399a	18770440	PRUPE_ppa026685mg	NCBI_Assembly:GCF_000346465.:	NW_006760201.1	+	11079278	11080984	1706	-	-	-	-
ppe-miR399a	18771679	PRUPE_ppa007084mg	NCBI_Assembly:GCF_000346465.:	NW_006760201.1	+	19304552	19307367	2815	-	-	-	-
ppe-miR399a	18771691	PRUPE_ppa011442mg	NCBI_Assembly:GCF_000346465.:	NW_006760201.1	-	21229994	21232404	2410	-	-	-	-
ppe-miR399a	18771775	PRUPE_ppb010572mg	NCBI_Assembly:GCF_000346465.:	NW_006760201.1	+	2358432	2359456	1024	-	-	-	-
ppe-miR399a	18771873	PRUPE_ppa021278mg	NCBI_Assembly:GCF_000346465.:	NW_006760201.1	-	12864874	12866558	1684	-	-	-	-
ppe-miR399a	18771908	PRUPE_ppa018518mg	NCBI_Assembly:GCF_000346465.:	NW_006760201.1	-	22282558	22286045	3487	-	GO:0000212:meiotic spindle organization;GO:0007140:male meiosis;GO:0009553:embryo sac development;GO:0009555:pollen development;GO:0042138:meiotic DNA double-strand break formation;GO:0048236:plant-type spore development;GO:0051026:chiasma assembly	-	-
ppe-miR399a	18771913	PRUPE_ppa000802mg	NCBI_Assembly:GCF_000346465.:	NW_006760201.1	+	7509053	7515515	6462	-	-	GO:0004672:protein kinase activity;GO:0005524:ATP binding	-

ppe-miR399a	18771977	PRUPE_ppa004703mg	NCBI_Assembly:GCF_000346465.:	NW_006760208.1	+	1068561	1071315	2754	pper01100:Metabolic pathways;pper01110:Biosynthesis of secondary metabolites;pper01230:Biosynthesis of amino acids;pper00230:Purine metabolism;pper01200:Carbon metabolism;pper00010:Glycolysis / Gluconeogenesis;pper00620:Pyruvate metabolism	GO:0006096: glycolytic process	GO:0000287: magnesium ion binding;GO:0004743:pyruvate kinase activity;GO:0030955:potassium ion binding	-
ppe-miR399a	18771980	PRUPE_ppa021292mg	NCBI_Assembly:GCF_000346465.:	NW_006760208.1	-	20897171	20898733	1562	-	-	GO:0008080: N-acetyltransferase activity	-
ppe-miR399a	18772015	PRUPE_ppa020283mg	NCBI_Assembly:GCF_000346465.:	NW_006760208.1	-	94062	97247	3185	pper01100:Metabolic pathways;pper00562:Inositol phosphate metabolism;pper04070:Phosphatidylinositol signaling system	GO:0016042:lipid catabolic process;GO:0035556:intracellular signal transduction	GO:0004435: phosphatidylinositol phospholipase C activity;GO:0004871:signal transducer activity	GO:0005622:intracellular
ppe-miR399a	18772171	PRUPE_ppa008068mg	NCBI_Assembly:GCF_000346465.:	NW_006760208.1	-	27629821	27631484	1663	-	-	-	-

ppe-miR399a	18772283	PRUPE_ppa018984mg	NCBI_Assembly:GCF_000346465.:	NW_006760208.1	-	4712771	4714461	1690	pper00960:Tropane, piperidine and pyridine alkaloid biosynthesis;pper01100:Metabolic pathways;pper01110:Biosynthesis of secondary metabolites	-	GO:0016491: oxidoreductase activity	-
ppe-miR399a	18772284	PRUPE_ppa004870mg	NCBI_Assembly:GCF_000346465.:	NW_006760208.1	+	25189032	25193057	4025	-	-	GO:0004674: protein serine/threonine kinase activity;GO:0005524:ATP binding	-
ppe-miR399a	18772291	PRUPE_ppa020056mg	NCBI_Assembly:GCF_000346465.:	NW_006760208.1	-	24346831	24347302	471	-	-	-	-
ppe-miR399a	18772340	PRUPE_ppa014434mg	NCBI_Assembly:GCF_000346465.:	NW_006760208.1	+	28701796	28703240	1444	pper03010:Ribosome	GO:0006412:translation	GO:0003735: structural constituent of ribosome	GO:0005840:ribosome
ppe-miR399a	18772437	PRUPE_ppb024320mg	NCBI_Assembly:GCF_000346465.:	NW_006760208.1	-	7909830	7911032	1202	pper01100:Metabolic pathways;pper01110:Biosynthesis of secondary metabolites;pper00020:Citrate cycle (TCA cycle)	-	GO:0005524: ATP binding	-
ppe-miR399a	18772470	PRUPE_ppa004966mg	NCBI_Assembly:GCF_000346465.:	NW_006760208.1	+	26654665	26659360	4695	-	GO:0042149: cellular response to glucose starvation;GO:0046777:protein autophosphorylation	GO:0004674: protein serine/threonine kinase activity;GO:0030295:protein kinase activator activity	-

ppe-miR399a	18772589	PRUPE_ppb012338mg	NCBI_Assembly:GCF_000346465.:	NW_006760208.1	-	17624453	17625651	1198	-	-	GO:0005509: calcium ion binding;GO:0005544:calcium-dependent phospholipid binding	-
ppe-miR399a	18772713	PRUPE_ppa026856mg	NCBI_Assembly:GCF_000346465.:	NW_006760208.1	+	4756263	4760831	4568	-	GO:0015074: DNA integration	GO:0003676: nucleic acid binding;GO:0008270:zinc ion binding	-
ppe-miR399a	18772717	PRUPE_ppa011013mg	NCBI_Assembly:GCF_000346465.:	NW_006760208.1	+	3083440	3086470	3030	pper03040:Spliceosome	GO:0000398: mRNA splicing, via spliceosome	-	GO:0005681:spliceosomal complex
ppe-miR399a	18772848	PRUPE_ppa012512mg	NCBI_Assembly:GCF_000346465.:	NW_006760208.1	-	28597961	28600262	2301	-	GO:0051252:regulation of RNA metabolic process	GO:0008428:ribonuclease inhibitor activity;GO:0008948:oxaloacetate decarboxylase activity;GO:0046872:metal ion binding;GO:0047443:4-hydroxy-4-methyl-2-oxoglutarate aldolase activity	-

ppe-miR399a	18772854	PRUPE_ppa009739mg	NCBI_Assembly:GCF_000346465.:	NW_006760208.1	-	27613927	27614972	1045	pper01100:Metabolic pathways;pper01110:Biosynthesis of secondary metabolites;pper01230:Biosynthesis of amino acids;pper01200:Carbon metabolism;pper00710:Carbon fixation in photosynthetic organisms;pper00030:Pentose phosphate pathway	GO:0000165:MAPK cascade;GO:0006546:glycine catabolic process;GO:0006569:tryptophan catabolic process;GO:0006612:protein targeting to membrane;GO:0006636:unsaturated fatty acid biosynthetic process;GO:0006766:vitamin metabolic process;GO:0009052:pentose-phosphate shunt, non-oxidative branch;GO:0009106:lipoate metabolic process;GO:0009108:coenzyme biosynthetic process;GO:0009409:response to cold;GO:0009595:detection of biotic stimulus;GO:0009684:indole	GO:0004751:ribose-5-phosphate isomerase activity	GO:0009535:chloroplast thylakoid membrane;GO:0009570:chloroplast stroma;GO:0009941:chloroplast envelope

ppe-miR399a	18772857	PRUPE_ppa015172mg	NCBI_Assembly:GCF_000346465.:	NW_006760208.1	-	4788590	4790475	1885	pper00960:Tropane, piperidine and pyridine alkaloid biosynthesis;pper01100:Metabolic pathways;pper01110:Biosynthesis of secondary metabolites	-	-	-
ppe-miR399a	18772890	PRUPE_ppa005628mg	NCBI_Assembly:GCF_000346465.:	NW_006760208.1	+	28797761	28803086	5325	-	GO:0000184:nuclear-transcribed mRNA catabolic process, nonsense-mediated decay;GO:0006346:methylation-dependent chromatin silencing;GO:0009910:negative regulation of flower development;GO:0016246:RNA interference	GO:0000166:nucleotide binding;GO:0003729:mRNA binding	GO:0005634:nucleus;GO:0005829:cytosol
ppe-miR399a	18772894	PRUPE_ppa016122mg	NCBI_Assembly:GCF_000346465.:	NW_006760208.1	-	17139540	17140477	937	-	-	-	-

ppe-miR399a	18772908	PRUPE_ppa008848mg	NCBI_Assembly:GCF_000346465.:	NW_006760208.1	-	16892942	16895991	3049	pper03018:RNA degradation	GO:0009910: negative regulation of flower development; GO:0010452: histone H3-K36 methylation;GO:0051568:histone H3-K4 methylation	-	GO:0005829:cytosol;GO:0080008:Cul4-RING E3 ubiquitin ligase complex
ppe-miR399a	18772965	PRUPE_ppa003133mg	NCBI_Assembly:GCF_000346465.:	NW_006760208.1	+	979212	984709	5497	pper00970:Aminoacyl-tRNA biosynthesis	GO:0006364:rRNA processing;GO:0006430:lysyl-tRNA aminoacylation;GO:0009658:chloroplast organization; GO:0009793: embryo development ending in seed dormancy;GO:0010027:thylakoid membrane organization; GO:0010228: vegetative to reproductive phase transition of meristem;GO:0016226:iron-sulfur cluster assembly;GO:0048481:ovule development	GO:0003676: nucleic acid binding;GO:0004824:lysine-tRNA ligase activity;GO:0005524:ATP binding	GO:0005739:mitochondrion;GO:0009507:chloroplast

ppe-miR399a	18773023	PRUPE_ppa000481mg	NCBI_Assembly:GCF_000346465.:	NW_006760208.1	-	2734047	2740737	6690	pper03018:RNA degradation	-	-	-
ppe-miR399a	18773064	PRUPE_ppa026542mg	NCBI_Assembly:GCF_000346465.:	NW_006760208.1	+	19097088	19100921	3833	-	-	-	-
ppe-miR399a	18773109	PRUPE_ppa002400mg	NCBI_Assembly:GCF_000346465.:	NW_006760208.1	-	3282932	3284965	2033	-	GO:0000226: microtubule cytoskeleton organization;GO:0000911: cytokinesis by cell plate formation	GO:0004674: protein serine/threonine kinase activity;GO:0005524:ATP binding;GO:0030246:carbohydrate binding	GO:0005886:plasma membrane
ppe-miR399a	18773200	PRUPE_ppa015129mg	NCBI_Assembly:GCF_000346465.:	NW_006760208.1	+	24389492	24392166	2674	-	-	-	-
ppe-miR399a	18773259	PRUPE_ppa006223mg	NCBI_Assembly:GCF_000346465.:	NW_006760208.1	-	28525751	28528504	2753	-	-	-	-
ppe-miR399a	18773397	PRUPE_ppa003394mg	NCBI_Assembly:GCF_000346465.:	NW_006760208.1	-	27065063	27066929	1866	-	-	GO:0004674: protein serine/threonine kinase activity;GO:0005524:ATP binding	-
ppe-miR399a	18773404	PRUPE_ppa013372mg	NCBI_Assembly:GCF_000346465.:	NW_006760208.1	+	28268097	28269061	964	-	-	-	-
ppe-miR399a	18773668	PRUPE_ppa011071mg	NCBI_Assembly:GCF_000346465.:	NW_006760208.1	-	11522776	11523683	907	-	-	-	GO:0009506:plasmodesma
ppe-miR399a	18773730	PRUPE_ppa027184mg	NCBI_Assembly:GCF_000346465.:	NW_006760208.1	+	11245198	11249694	4496	-	-	GO:0016491: oxidoreductase activity	-
ppe-miR399a	18773826	PRUPE_ppa017358mg	NCBI_Assembly:GCF_000346465.:	NW_006760208.1	+	28259234	28260154	920	-	-	-	-
ppe-miR399a	18773944	PRUPE_ppa016194mg	NCBI_Assembly:GCF_000346465.:	NW_006760208.1	-	23197844	23199790	1946	-	-	GO:0004674: protein serine/threonine kinase activity;GO:0005524:ATP binding;GO:0030246:carbohydrate binding	-

ppe-miR399a	18774027	PRUPE_ppa013881mg	NCBI_Assembly:GCF_000346465.:	NW_006760208.1	-	4662737	4664429	1692	pper04120:Ubiquitin mediated proteolysis	GO:0000394:RNA splicing, via endonucleolytic cleavage and ligation;GO:0006366:transcription from RNA polymerase II promoter;GO:0006511:ubiquitin-dependent protein catabolic process;GO:0009062:fatty acid catabolic process	-	GO:0005829:cytosol
ppe-miR399a	18774163	PRUPE_ppa022695m1g	NCBI_Assembly:GCF_000346465.:	NW_006760208.1	+	23979327	23979917	590	-	-	-	-
ppe-miR399a	18774332	PRUPE_ppa001694mg	NCBI_Assembly:GCF_000346465.:	NW_006760208.1	-	484594	490050	5456	pper01100:Metabolic pathways;pper00600:Sphingolipid metabolism	-	-	-
ppe-miR399a	18774351	PRUPE_ppa019859mg	NCBI_Assembly:GCF_000346465.:	NW_006760208.1	-	22852430	22853473	1043	-	-	-	-
ppe-miR399a	18774428	PRUPE_ppa026651mg	NCBI_Assembly:GCF_000346465.:	NW_006760208.1	-	4119490	4120669	1179	-	GO:0006351:transcription, DNA-templated	GO:0003677:DNA binding;GO:0003700:sequence-specific DNA binding transcription factor activity	GO:0005634:nucleus
ppe-miR399a	18774432	PRUPE_ppa015458mg	NCBI_Assembly:GCF_000346465.:	NW_006760208.1	+	13911905	13913007	1102	-	-	-	-
ppe-miR399a	18774435	PRUPE_ppa023074mg	NCBI_Assembly:GCF_000346465.:	NW_006760208.1	+	6095840	6096748	908	-	GO:0005975:carbohydrate metabolic process	GO:0004553:hydrolase activity, hydrolyzing O-glycosyl compounds	-

ppe-miR399a	18774504	PRUPE_ppa001899mg	NCBI_Assembly:GCF_000346465.:	NW_006760208.1	-	25628843	25632596	3753	pper04712:Circadian rhythm - plant;pper04075:Plant hormone signal transduction	GO:0007623:circadian rhythm;GO:0009630:gravitropism;GO:0009704:de-etiolation;GO:0009740:gibberellic acid mediated signaling pathway;GO:0031539:positive regulation of anthocyanin metabolic process	GO:0003700:sequence-specific DNA binding transcription factor activity	-
ppe-miR399a	18774649	PRUPE_ppa006076mg	NCBI_Assembly:GCF_000346465.:	NW_006760208.1	-	25424097	25428158	4061	pper01100:Metabolic pathways;pper00564:Glycerophospholipid metabolism	GO:0009058:biosynthetic process	GO:0003824:catalytic activity	GO:0031307:integral component of mitochondrial outer membrane
ppe-miR399a	18774666	PRUPE_ppa022021mg	NCBI_Assembly:GCF_000346465.:	NW_006760208.1	-	19820736	19824274	3538	-	GO:0005975:carbohydrate metabolic process	GO:0003824:catalytic activity;GO:0030246:carbohydrate binding	-
ppe-miR399a	18774762	PRUPE_ppa021055mg	NCBI_Assembly:GCF_000346465.:	NW_006760208.1	+	17374905	17376117	1212	-	-	-	-
ppe-miR399a	18774790	PRUPE_ppa015372mg	NCBI_Assembly:GCF_000346465.:	NW_006760208.1	-	22546621	22549793	3172	-	-	GO:0004674:protein serine/threonine kinase activity;GO:0005524:ATP binding	-
ppe-miR399a	18774939	PRUPE_ppa007778mg	NCBI_Assembly:GCF_000346465.:	NW_006760208.1	+	3852281	3856859	4578	-	-	GO:0016491:oxidoreductase activity	GO:0005829:cytosol
ppe-miR399a	18774988	PRUPE_ppa000364mg	NCBI_Assembly:GCF_000346465.:	NW_006760208.1	+	22390475	22398870	8395	-	-	-	-
ppe-miR399a	18775113	PRUPE_ppb017334mg	NCBI_Assembly:GCF_000346465.:	NW_006760208.1	-	12095488	12097332	1844	-	-	-	-

ppe-miR399a	18775209	PRUPE_ppa006991mg	NCBI_Assembly:GCF_000346465.:	NW_006760208.1	-	20044484	20048124	3640	-	-	GO:0008270: zinc ion binding	-
ppe-miR399a	18775296	PRUPE_ppa008597mg	NCBI_Assembly:GCF_000346465.:	NW_006760208.1	+	9069082	9070387	1305	-	-	GO:0004197: cysteine-type endopeptidase activity	-
ppe-miR399a	18775480	PRUPE_ppa012666mg	NCBI_Assembly:GCF_000346465.:	NW_006760208.1	+	419346	421239	1893	-	-	-	-
ppe-miR399a	18775490	PRUPE_ppa011828mg	NCBI_Assembly:GCF_000346465.:	NW_006760208.1	-	4422659	4423412	753	-	-	-	-
ppe-miR399a	18775496	PRUPE_ppa018731mg	NCBI_Assembly:GCF_000346465.:	NW_006760208.1	+	10450138	10450767	629	-	-	GO:0030145: manganese ion binding;GO:0045735:nutrient reservoir activity	GO:0005576:extracellular region
ppe-miR399a	18775568	PRUPE_ppa000045mg	NCBI_Assembly:GCF_000346465.:	NW_006760208.1	-	936998	956575	19577	-	-	GO:0004198: calcium-dependent cysteine-type endopeptidase activity	GO:0005622:intracellular
ppe-miR399a	18775570	PRUPE_ppa002436mg	NCBI_Assembly:GCF_000346465.:	NW_006760208.1	-	26809480	26814571	5091	-	-	GO:0004672: protein kinase activity;GO:0005524:ATP binding	-
ppe-miR399a	18775644	PRUPE_ppa015865mg	NCBI_Assembly:GCF_000346465.:	NW_006760212.1	+	4172077	4173474	1397	-	-	-	-
ppe-miR399a	18775693	PRUPE_ppa000133mg	NCBI_Assembly:GCF_000346465.:	NW_006760212.1	-	16118504	16126374	7870	-	-	-	-
ppe-miR399a	18775782	PRUPE_ppa008892mg	NCBI_Assembly:GCF_000346465.:	NW_006760212.1	+	483479	485358	1879	-	-	GO:0004672: protein kinase activity;GO:0005524:ATP binding	-
ppe-miR399a	18775951	PRUPE_ppa005259mg	NCBI_Assembly:GCF_000346465.:	NW_006760212.1	+	17045245	17050330	5085	-	-	GO:0008270: zinc ion binding	-

ppe-miR399a	18775983	PRUPE_ppa027074mg	NCBI_Assembly:GCF_000346465.:	NW_006760212.1	+	13950417	13953697	3280	pper01100:Metabolic pathways;pper01110:Biosynthesis of secondary metabolites;pper00460:Cyanoamino acid metabolism;pper00500:Starch and sucrose metabolism;pper00940:Phenylpropanoid biosynthesis	GO:0005975: carbohydrate metabolic process	GO:0004553: hydrolase activity, hydrolyzing O-glycosyl compounds	-
ppe-miR399a	18776170	PRUPE_ppa009958mg	NCBI_Assembly:GCF_000346465.:	NW_006760212.1	-	3457003	3458077	1074	-	-	GO:0016491: oxidoreductase activity	-
ppe-miR399a	18776179	PRUPE_ppa009208mg	NCBI_Assembly:GCF_000346465.:	NW_006760212.1	+	3752496	3753516	1020	-	-	GO:0008270: zinc ion binding	-
ppe-miR399a	18776282	PRUPE_ppa026808mg	NCBI_Assembly:GCF_000346465.:	NW_006760212.1	+	15251676	15252302	626	-	-	GO:0030145: manganese ion binding;GO:0045735:nutrient reservoir activity	GO:0005576:extracellular region
ppe-miR399a	18776296	PRUPE_ppa010475mg	NCBI_Assembly:GCF_000346465.:	NW_006760212.1	-	8169268	8170621	1353	-	-	GO:0005215:transporter activity	GO:0016021:integral component of membrane
ppe-miR399a	18776338	PRUPE_ppa021800mg	NCBI_Assembly:GCF_000346465.:	NW_006760212.1	+	12879034	12879549	515	-	-	-	-
ppe-miR399a	18776348	PRUPE_ppa014389mg	NCBI_Assembly:GCF_000346465.:	NW_006760212.1	+	9787181	9787984	803	-	-	-	-
ppe-miR399a	18776354	PRUPE_ppa009207mg	NCBI_Assembly:GCF_000346465.:	NW_006760212.1	-	17579509	17582224	2715	pper04146:Peroxisome	GO:0007031: peroxisome organization	-	GO:0005779:integral component of peroxisomal membrane
ppe-miR399a	18776481	PRUPE_ppa004570mg	NCBI_Assembly:GCF_000346465.:	NW_006760212.1	+	17974913	17978235	3322	-	-	-	-

ppe-miR399a	18776519	PRUPE_ppa005346mg	NCBI_Assembly:GCF_000346465.:	NW_006760212.1	-	16469317	16473788	4471	-	GO:0006810:transport;GO:0009737:response to abscisic acid	GO:0000166:nucleotide binding;GO:0003676:nucleic acid binding	GO:0005622:intracellular
ppe-miR399a	18776610	PRUPE_ppa024573mg	NCBI_Assembly:GCF_000346465.:	NW_006760212.1	+	14378850	14380949	2099	-	-	-	-
ppe-miR399a	18776623	PRUPE_ppa017021mg	NCBI_Assembly:GCF_000346465.:	NW_006760212.1	-	3990578	3992189	1611	-	-	-	-
ppe-miR399a	18776669	PRUPE_ppa010909mg	NCBI_Assembly:GCF_000346465.:	NW_006760212.1	-	10054488	10055353	865	-	GO:0006351:transcription, DNA-templated	GO:0003677:DNA binding;GO:0003700:sequence-specific DNA binding transcription factor activity	GO:0005634:nucleus
ppe-miR399a	18776755	PRUPE_ppa004485mg	NCBI_Assembly:GCF_000346465.:	NW_006760212.1	+	15126674	15130650	3976	pper01100:Metabolic pathways;pper01110:Biosynthesis of secondary metabolites;pper00520:Amino sugar and nucleotide sugar metabolism;pper00051:Fructose and mannose metabolism	GO:0009058:biosynthetic process	GO:0016779:nucleotidyltransferase activity	GO:0005777:peroxisome;GO:0005829:cytosol
ppe-miR399a	18776883	PRUPE_ppa023135mg	NCBI_Assembly:GCF_000346465.:	NW_006760212.1	+	17433502	17433816	314	-	-	-	-
ppe-miR399a	18776954	PRUPE_ppa020095mg	NCBI_Assembly:GCF_000346465.:	NW_006760212.1	+	9427609	9429005	1396	-	-	-	-
ppe-miR399a	18776980	PRUPE_ppa026241mg	NCBI_Assembly:GCF_000346465.:	NW_006760212.1	+	16734996	16738161	3165	-	-	-	-

ppe-miR399a	18777155	PRUPE_ppa014547mg	NCBI_Assembly:GCF_000346465.:	NW_006760212.1	-	18323041	18325086	2045	pper01100:Metabolic pathways;pper00230:Purine metabolism;pper00240:Pyrimidine metabolism;pper03020:RNA polymerase	GO:0006351:transcription, DNA-templated	GO:0003677:DNA binding;GO:0003899:DNA-directed RNA polymerase activity	-
ppe-miR399a	18777185	PRUPE_ppa008757mg	NCBI_Assembly:GCF_000346465.:	NW_006760212.1	-	11307803	11312183	4380	pper03015:mRNA surveillance pathway	-	GO:0004721:phosphoprotein phosphatase activity	-
ppe-miR399a	18777217	PRUPE_ppa015306mg	NCBI_Assembly:GCF_000346465.:	NW_006760212.1	-	273485	274878	1393	-	GO:0006886:intracellular protein transport;GO:0016192:vesicle-mediated transport	GO:0008565:protein transporter activity	GO:0030117:membrane coat
ppe-miR399a	18777238	PRUPE_ppa001170mg	NCBI_Assembly:GCF_000346465.:	NW_006760212.1	+	12737702	12745290	7588	-	GO:0006511:ubiquitin-dependent protein catabolic process	GO:0004843:ubiquitin-specific protease activity	-
ppe-miR399a	18777245	PRUPE_ppa019629mg	NCBI_Assembly:GCF_000346465.:	NW_006760212.1	+	399657	400754	1097	-	-	-	-
ppe-miR399a	18777246	PRUPE_ppa006929mg	NCBI_Assembly:GCF_000346465.:	NW_006760212.1	+	17800856	17804219	3363	-	-	-	-
ppe-miR399a	18777410	PRUPE_ppa019736mg	NCBI_Assembly:GCF_000346465.:	NW_006760212.1	-	13964471	13965279	808	-	GO:0006412:translation	GO:0003735:structural constituent of ribosome	GO:0005840:ribosome

ppe-miR399a	18777515	PRUPE_ppa006372mg	NCBI_Assembly:GCF_000346465.:	NW_006760212.1	+	13116676	13119151	2475	-	GO:0009740: gibberellic acid mediated signaling pathway;GO:0010077:maintenance of inflorescence meristem identity;GO:0010582:floral meristem determinacy	GO:0003700: sequence-specific DNA binding transcription factor activity;GO:0031490:chromatin DNA binding;GO:0043565:sequence-specific DNA binding	-
ppe-miR399a	18777540	PRUPE_ppa005332mg	NCBI_Assembly:GCF_000346465.:	NW_006760212.1	-	13943720	13948088	4368	-	-	GO:0004672: protein kinase activity;GO:0005524:ATP binding	-
ppe-miR399a	18777640	PRUPE_ppa019151mg	NCBI_Assembly:GCF_000346465.:	NW_006760212.1	-	15793129	15795926	2797	-	-	GO:0008270: zinc ion binding	-
ppe-miR399a	18777650	PRUPE_ppa025025mg	NCBI_Assembly:GCF_000346465.:	NW_006760212.1	-	4607045	4607743	698	-	-	-	GO:0005615:extracellular space
ppe-miR399a	18777654	PRUPE_ppa016860mg	NCBI_Assembly:GCF_000346465.:	NW_006760212.1	-	3006577	3009415	2838	-	GO:0006281: DNA repair	GO:0003677: DNA binding;GO:0004519:endonuclease activity	GO:0005622:intracellular
ppe-miR399a	18777712	PRUPE_ppa006936mg	NCBI_Assembly:GCF_000346465.:	NW_006760212.1	+	5321105	5328026	6921	-	GO:0016571: histone methylation;GO:0048451:petal formation;GO:0048453:sepal formation	-	-
ppe-miR399a	18777715	PRUPE_ppa007046mg	NCBI_Assembly:GCF_000346465.:	NW_006760212.1	+	10848903	10853296	4393	pper00970:Aminoacyl-tRNA biosynthesis	GO:0006418:tRNA aminoacylation for protein translation	GO:0004831:tyrosine-tRNA ligase activity;GO:0005524:ATP binding	-

ppe-miR399a	18777914	PRUPE_ppa018401mg	NCBI_Assembly:GCF_000346465.:	NW_006760212.1	-	13075355	13075843	488	-	GO:0006457: protein folding;GO:0009408:response to heat;GO:0009644:response to high light intensity;GO:0042542:response to hydrogen peroxide	-	-
ppe-miR399a	18777918	PRUPE_ppb023334mg	NCBI_Assembly:GCF_000346465.:	NW_006760212.1	+	1815081	1816997	1916	-	-	-	-
ppe-miR399a	18777935	PRUPE_ppa004805mg	NCBI_Assembly:GCF_000346465.:	NW_006760212.1	+	17510063	17513103	3040	-	-	GO:0015238: drug transmembrane transporter activity;GO:0015297:antiporter activity	GO:0016021:integral component of membrane
ppe-miR399a	18777977	PRUPE_ppa018992mg	NCBI_Assembly:GCF_000346465.:	NW_006760212.1	-	12343935	12344243	308	-	-	GO:0003676: nucleic acid binding	-
ppe-miR399a	18778176	PRUPE_ppa009430mg	NCBI_Assembly:GCF_000346465.:	NW_006760220.1	+	2306567	2308181	1614	-	-	-	-
ppe-miR399a	18778305	PRUPE_ppa016168mg	NCBI_Assembly:GCF_000346465.:	NW_006760220.1	+	4305742	4309784	4042	-	-	-	-
ppe-miR399a	18778507	PRUPE_ppa020500mg	NCBI_Assembly:GCF_000346465.:	NW_006760220.1	-	14405724	14407874	2150	-	-	-	-

ppe-miR399a	18778617	PRUPE_ppa000701mg	NCBI_Assembly:GCF_000346465.:	NW_006760220.1	+	8274643	8281598	6955	-	GO:0009902:chloroplast relocation;GO:0010027:thylakoid membrane organization;GO:0010155:regulation of proton transport;GO:0019288:isopentenyl diphosphate biosynthetic process, methylerythritol 4-phosphate pathway;GO:0034660:ncRNA metabolic process;GO:0042793:transcription from plastid promoter;GO:0046777:protein autophosphorylation	GO:0003743:translation initiation factor activity;GO:0003924:GTPase activity;GO:0005525:GTP binding	GO:0009570:chloroplast stroma;GO:0009941:chloroplast envelope
ppe-miR399a	18778624	PRUPE_ppa010619mg	NCBI_Assembly:GCF_000346465.:	NW_006760220.1	+	5317466	5320763	3297	pper01100:Metabolic pathways;pper00760:Nicotinate and nicotinamide metabolism	GO:0009435:NAD biosynthetic process	GO:0000309:nicotinamide-nucleotide adenylyltransferase activity;GO:0004515:nicotinate-nucleotide adenylyltransferase activity;GO:0005524:ATP binding	-

ppe-miR399a	18778625	PRUPE_ppa026628mg	NCBI_Assembly:GCF_000346465.:	NW_006760220.1	-	17183768	17185556	1788	-	-	GO:0004497: monooxygenase activity;GO:0005506:iron ion binding;GO:0016705:oxidoreductase activity, acting on paired donors, with incorporation or reduction of molecular oxygen;GO:0020037:heme binding	-
ppe-miR399a	18778695	PRUPE_ppa004903mg	NCBI_Assembly:GCF_000346465.:	NW_006760220.1	-	6203915	6210952	7037	pper01100:Metabolic pathways;pper01110:Biosynthesis of secondary metabolites;pper00520:Amino sugar and nucleotide sugar metabolism	-	GO:0070569: uridylyltransferase activity	-
ppe-miR399a	18778704	PRUPE_ppa012099mg	NCBI_Assembly:GCF_000346465.:	NW_006760220.1	-	13415929	13421283	5354	-	-	GO:0008137: NADH dehydrogenase (ubiquinone) activity;GO:0009055:electron carrier activity	GO:0016020:membrane

ppe-miR399a	18778793	PRUPE_ppa006022mg	NCBI_Assembly:GCF_000346465.:	NW_006760220.1	+	6214403	6220603	6200	pper00970:Aminoacyl-tRNA biosynthesis	GO:0006432:phenylalanyl-tRNA aminoacylation;GO:0008033:tRNA processing;GO:0009658:chloroplast organization;GO:0019288:isopentenyl diphosphate biosynthetic process, methylerythritol 4-phosphate pathway;GO:0048481:ovule development	GO:0000049:tRNA binding;GO:0000287:magnesium ion binding;GO:0004826:phenylalanine-tRNA ligase activity;GO:0005524:ATP binding	GO:0005737:cytoplasm;GO:0016020:membrane
ppe-miR399a	18778921	PRUPE_ppa013364mg	NCBI_Assembly:GCF_000346465.:	NW_006760220.1	-	2454336	2457059	2723	-	-	-	-

ppe-miR399a	18778963	PRUPE_ppa020652mg	NCBI_Assembly:GCF_000346465.:	NW_006760220.1	-	25019621	25030946	11325	-	GO:0007155: cell adhesion;GO: 0009887:organ morphogenesis;GO:0010090:trichome morphogenesis;GO:0010228:vegetative to reproductive phase transition of meristem;GO: 0016926:protein desumoylation;GO:0019375: galactolipid biosynthetic process;GO:0033044:regulation of chromosome organization; GO:0045010: actin nucleation;GO:0048765:root hair cell differentiation; GO:0050665: hydrogen peroxide biosynthetic process;GO:0071555:cell	-	GO:0005741:mitochondrial outer membrane;GO: 0043234:protein complex
ppe-miR399a	18779154	PRUPE_ppa001954mg	NCBI_Assembly:GCF_000346465.:	NW_006760220.1	-	8712703	8718586	5883	-	-	-	-

ppe-miR399a	18779254	PRUPE_ppa012670mg	NCBI_Assembly:GCF_000346465.:	NW_006760220.1	-	10295899	10298498	2599	pper03030:DNA replication;pper03420:Nucleotide excision repair;pper03430:Mismatch repair;pper03440:Homologous recombination	-	-	-
ppe-miR399a	18779410	PRUPE_ppa008293mg	NCBI_Assembly:GCF_000346465.:	NW_006760220.1	+	13837265	13841725	4460	-	-	-	GO:0016020:membrane
ppe-miR399a	18779450	PRUPE_ppa018537mg	NCBI_Assembly:GCF_000346465.:	NW_006760220.1	+	7449667	7452431	2764	-	-	GO:0004672:protein kinase activity;GO:0005524:ATP binding	-
ppe-miR399a	18779452	PRUPE_ppa024643mg	NCBI_Assembly:GCF_000346465.:	NW_006760220.1	-	5112924	5114337	1413	-	-	GO:0022857:transmembrane transporter activity	GO:0016021:integral component of membrane
ppe-miR399a	18779471	PRUPE_ppb013698mg	NCBI_Assembly:GCF_000346465.:	NW_006760220.1	+	24362120	24364001	1881	pper01100:Metabolic pathways;pper01110:Biosynthesis of secondary metabolites;pper00860:Porphyrin and chlorophyll metabolism	-	GO:0004659:prenyltransferase activity	GO:0016021:integral component of membrane
ppe-miR399a	18779583	PRUPE_ppa018959mg	NCBI_Assembly:GCF_000346465.:	NW_006760220.1	+	10345044	10346978	1934	-	-	GO:0004674:protein serine/threonine kinase activity;GO:0005524:ATP binding;GO:0030246:carbohydrate binding	-

ppe-miR399a	18779740	PRUPE_ppa018958mg	NCBI_Assembly:GCF_000346465.:	NW_006760220.1	-	18184139	18184519	380	-	-	-	-
ppe-miR399a	18779913	PRUPE_ppa025030mg	NCBI_Assembly:GCF_000346465.:	NW_006760220.1	-	15881222	15883971	2749	-	-	GO:0003676: nucleic acid binding;GO:0008270:zinc ion binding	-
ppe-miR399a	18779991	PRUPE_ppa008046mg	NCBI_Assembly:GCF_000346465.:	NW_006760220.1	+	20920376	20923256	2880	-	-	-	-
ppe-miR399a	18780079	PRUPE_ppa015082mg	NCBI_Assembly:GCF_000346465.:	NW_006760220.1	-	24810888	24811947	1059	pper01100:Metabolic pathways;pper01110:Biosynthesis of secondary metabolites;pper00860:Porphyrin and chlorophyll metabolism	-	GO:0004659: prenyltransferase activity	GO:0016021:integral component of membrane
ppe-miR399a	18780103	PRUPE_ppa023148mg	NCBI_Assembly:GCF_000346465.:	NW_006760220.1	-	9365859	9367794	1935	-	-	-	-
ppe-miR399a	18780108	PRUPE_ppa016234mg	NCBI_Assembly:GCF_000346465.:	NW_006760220.1	-	5105620	5106582	962	-	-	GO:0022857:transmembrane transporter activity	GO:0016021:integral component of membrane
ppe-miR399a	18781481	PRUPE_ppa022572mg	NCBI_Assembly:GCF_000346465.:	NW_006760220.1	-	4701199	4704171	2972	-	-	GO:0004674: protein serine/threonine kinase activity;GO:0005509:calcium ion binding;GO:0005524:ATP binding;GO:0030247:polysaccharide binding	-
ppe-miR399a	18782115	PRUPE_ppa016771mg	NCBI_Assembly:GCF_000346465.:	NW_006760268.1	+	19647980	19650114	2134	-	-	GO:0005215:transporter activity	GO:0016021:integral component of membrane
ppe-miR399a	18782928	PRUPE_ppa001317mg	NCBI_Assembly:GCF_000346465.:	NW_006760268.1	+	4261864	4266973	5109	-	-	GO:0005524: ATP binding	-

ppe-miR399a	18782948	PRUPE_ppa010211mg	NCBI_Assembly:GCF_000346465.:	NW_006760268.1	-	15700209	15701775	1566	pper01100:Metabolic pathways;pper00240:Pyrimidine metabolism	GO:0071897:DNA biosynthetic process	GO:0004797:thymidine kinase activity;GO:0005524:ATP binding	-
ppe-miR399a	18782972	PRUPE_ppb019240mg	NCBI_Assembly:GCF_000346465.:	NW_006760268.1	+	6542704	6543256	552	-	GO:0006694:steroid biosynthetic process	GO:0003854:3-beta-hydroxy-delta5-steroid dehydrogenase activity	-
ppe-miR399a	18783081	PRUPE_ppa006640mg	NCBI_Assembly:GCF_000346465.:	NW_006760268.1	+	6029077	6030548	1471	pper00400:Phenylalanine, tyrosine and tryptophan biosynthesis;pper01100:Metabolic pathways;pper01230:Biosynthesis of amino acids	GO:0006567:threonine catabolic process;GO:0006571:tyrosine biosynthetic process	GO:0004665:prephenate dehydrogenase (NADP+) activity;GO:0008977:prephenate dehydrogenase activity	-
ppe-miR399a	18783095	PRUPE_ppa019948mg	NCBI_Assembly:GCF_000346465.:	NW_006760268.1	+	6264607	6266070	1463	pper01100:Metabolic pathways;pper01110:Biosynthesis of secondary metabolites;pper00940:Phenylpropanoid biosynthesis;pper00941:Flavonoid biosynthesis;pper00945:Stilbenoid, diarylheptanoid and gingerol biosynthesis	-	GO:0016747:transferase activity, transferring acyl groups other than amino-acyl groups	-

ppe-miR399a	18783111	PRUPE_ppa027044mg	NCBI_Assembly:GCF_000346465.:	NW_006760268.1	-	3373731	3381346	7615	-	-	GO:0005524: ATP binding;GO:0016887:ATPase activity	GO:0016020:membrane
ppe-miR399a	18783112	PRUPE_ppa026527mg	NCBI_Assembly:GCF_000346465.:	NW_006760268.1	-	2143200	2144460	1260	pper04140:Regulation of autophagy	GO:0000045: autophagosome assembly	-	GO:0005737:cytoplasm
ppe-miR399a	18783181	PRUPE_ppa015446mg	NCBI_Assembly:GCF_000346465.:	NW_006760268.1	+	2963349	2964677	1328	-	-	GO:0003700: sequence-specific DNA binding transcription factor activity	-
ppe-miR399a	18783278	PRUPE_ppa025611mg	NCBI_Assembly:GCF_000346465.:	NW_006760268.1	-	16129350	16131571	2221	-	GO:0016042:lipid catabolic process	GO:0016788: hydrolase activity, acting on ester bonds	-
ppe-miR399a	18783409	PRUPE_ppa007776mg	NCBI_Assembly:GCF_000346465.:	NW_006760268.1	-	17737673	17739720	2047	-	-	GO:0008171: O-methyltransferase activity	-
ppe-miR399a	18783468	PRUPE_ppa002875mg	NCBI_Assembly:GCF_000346465.:	NW_006760268.1	-	950340	956010	5670	-	GO:0015992: proton transport	GO:0004427:inorganic diphosphatase activity;GO:0009678:hydrogen-translocating pyrophosphatase activity	GO:0016020:membrane
ppe-miR399a	18783512	PRUPE_ppa012953mg	NCBI_Assembly:GCF_000346465.:	NW_006760268.1	-	5157084	5158957	1873	-	GO:0006412:translation	GO:0003735: structural constituent of ribosome;GO:0019843:rRNA binding	GO:0005840:ribosome;GO:0009507:chloroplast
ppe-miR399a	18783607	PRUPE_ppa024395mg	NCBI_Assembly:GCF_000346465.:	NW_006760268.1	+	13169570	13171966	2396	-	-	-	-
ppe-miR399a	18783854	PRUPE_ppa007817mg	NCBI_Assembly:GCF_000346465.:	NW_006760268.1	+	19047717	19051663	3946	-	-	GO:0003824: catalytic activity	-
ppe-miR399a	18783876	PRUPE_ppa016125mg	NCBI_Assembly:GCF_000346465.:	NW_006760268.1	-	16475354	16476272	918	-	-	-	-

ppe-miR399a	18783908	PRUPE_ppa013978mg	NCBI_Assembly:GCF_000346465.:	NW_006760268.1	-	3522490	3523281	791	-	-	-	-
ppe-miR399a	18784047	PRUPE_ppb021358mg	NCBI_Assembly:GCF_000346465.:	NW_006760268.1	+	14949585	14950096	511	-	-	GO:0016758:transferase activity, transferring hexosyl groups	-
ppe-miR399a	18784144	PRUPE_ppa014864mg	NCBI_Assembly:GCF_000346465.:	NW_006760268.1	+	3477645	3479488	1843	-	GO:0006306:DNA methylation;GO:0006346:methylation-dependent chromatin silencing;GO:0033562:co-transcriptional gene silencing by RNA interference machinery	-	-
ppe-miR399a	18784159	PRUPE_ppa020823mg	NCBI_Assembly:GCF_000346465.:	NW_006760268.1	-	14649691	14649912	221	-	-	-	-
ppe-miR399a	18784161	PRUPE_ppa022634mg	NCBI_Assembly:GCF_000346465.:	NW_006760268.1	-	16924369	16925538	1169	-	-	-	-
ppe-miR399a	18784207	PRUPE_ppa007792mg	NCBI_Assembly:GCF_000346465.:	NW_006760268.1	-	4440498	4441622	1124	-	-	-	-
ppe-miR399a	18784259	PRUPE_ppb012503mg	NCBI_Assembly:GCF_000346465.:	NW_006760268.1	-	726336	729625	3289	-	-	-	-
ppe-miR399a	18784292	PRUPE_ppa024929mg	NCBI_Assembly:GCF_000346465.:	NW_006760268.1	+	3465850	3467610	1760	-	-	-	-
ppe-miR399a	18784382	PRUPE_ppa009023mg	NCBI_Assembly:GCF_000346465.:	NW_006760268.1	+	3000627	3003430	2803	pper00562:Inositol phosphate metabolism;pper00053:Ascorbate and aldarate metabolism	GO:0019310:inositol catabolic process	GO:0005506:iron ion binding;GO:0050113:inositol oxygenase activity	GO:0005737:cytoplasm

ppe-miR399a	18784392	PRUPE_ppa014443mg	NCBI_Assembly:GCF_000346465.:	NW_006760268.1	-	21468808	21469557	749	pper04141:Protein processing in endoplasmic reticulum;pper03060:Protein export;pper04145:Phagosome	GO:0006605: protein targeting;GO:0015824:proline transport	GO:0015450: P-P-bond-hydrolysis-driven protein transmembrane transporter activity	GO:0005622:intracellular;GO:0005886:plasma membrane
ppe-miR399a	18784575	PRUPE_ppa002045mg	NCBI_Assembly:GCF_000346465.:	NW_006760324.1	-	22272262	22276834	4572	-	GO:0006281: DNA repair;GO:0006355:regulation of transcription, DNA-templated	-	GO:0031011:Ino80 complex
ppe-miR399a	18784613	PRUPE_ppa005987mg	NCBI_Assembly:GCF_000346465.:	NW_006760324.1	-	23634170	23637502	3332	-	-	-	GO:0016021:integral component of membrane
ppe-miR399a	18784637	PRUPE_ppa002306mg	NCBI_Assembly:GCF_000346465.:	NW_006760324.1	+	10754049	10760153	6104	-	-	-	-
ppe-miR399a	18784939	PRUPE_ppa026025mg	NCBI_Assembly:GCF_000346465.:	NW_006760324.1	-	25300916	25305264	4348	-	GO:0009058: biosynthetic process	GO:0005524: ATP binding;GO:0008763:UDP-N-acetylmuramate-L-alanine ligase activity	-

ppe-miR399a	18785087	PRUPE_ppa004577mg	NCBI_Assembly:GCF_000346465.:	NW_006760324.1	-	22056015	22058580	2565	-	GO:0006487: protein N-linked glycosylation;GO:0009408:response to heat;GO:0009644:response to high light intensity;GO:0042542:response to hydrogen peroxide	-	GO:0009941:chloroplast envelope
ppe-miR399a	18785109	PRUPE_ppa002451mg	NCBI_Assembly:GCF_000346465.:	NW_006760324.1	-	16799282	16803235	3953	-	GO:0009630: gravitropism	GO:0046872: metal ion binding	-
ppe-miR399a	18785130	PRUPE_ppa002373mg	NCBI_Assembly:GCF_000346465.:	NW_006760324.1	+	24422692	24426108	3416	-	GO:0010075:regulation of meristem growth	GO:0004672: protein kinase activity;GO:0005524:ATP binding	GO:0016021:integral component of membrane
ppe-miR399a	18785186	PRUPE_ppa019273mg	NCBI_Assembly:GCF_000346465.:	NW_006760324.1	+	4731013	4732968	1955	-	-	-	-
ppe-miR399a	18785225	PRUPE_ppa000519mg	NCBI_Assembly:GCF_000346465.:	NW_006760324.1	-	2892000	2897834	5834	-	GO:0009909:regulation of flower development;GO:0010228: vegetative to reproductive phase transition of meristem;GO:0010452:histone H3-K36 methylation;GO:0051568:histone H3-K4 methylation	GO:0018024: histone-lysine N-methyltransferase activity	GO:0005634:nucleus;GO:0005737:cytoplasm
ppe-miR399a	18785337	PRUPE_ppa009506mg	NCBI_Assembly:GCF_000346465.:	NW_006760324.1	-	22777485	22779328	1843	-	-	GO:0005215:transporter activity	GO:0016021:integral component of membrane

ppe-miR399a	18785348	PRUPE_ppa004573mg	NCBI_Assembly:GCF_000346465.:	NW_006760324.1	-	23505584	23509707	4123	-	-	GO:0005471:ATP:ADP antiporter activity;GO:0005524:ATP binding	GO:0016021:integral component of membrane
ppe-miR399a	18785375	PRUPE_ppa011632mg	NCBI_Assembly:GCF_000346465.:	NW_006760324.1	+	23855695	23858239	2544	-	-	GO:0001104:RNA polymerase II transcription cofactor activity	GO:0016592:mediator complex
ppe-miR399a	18785540	PRUPE_ppa004040mg	NCBI_Assembly:GCF_000346465.:	NW_006760324.1	-	3994120	4002085	7965	pper04120:Ubiquitin mediated proteolysis	GO:0006281:DNA repair;GO:0010100:negative regulation of photomorphogenesis;GO:0048608:reproductive structure development	GO:0003824:catalytic activity	-
ppe-miR399a	18785559	PRUPE_ppa003293mg	NCBI_Assembly:GCF_000346465.:	NW_006760324.1	+	24238991	24244021	5030	pper04141:Protein processing in endoplasmic reticulum	-	GO:0008270:zinc ion binding	-
ppe-miR399a	18785593	PRUPE_ppa026670mg	NCBI_Assembly:GCF_000346465.:	NW_006760324.1	+	21180140	21181818	1678	-	-	-	-
ppe-miR399a	18785660	PRUPE_ppa003661mg	NCBI_Assembly:GCF_000346465.:	NW_006760324.1	-	25595443	25597590	2147	-	GO:0006635:fatty acid beta-oxidation;GO:0016558:protein import into peroxisome matrix	-	GO:0005622:intracellular
ppe-miR399a	18785807	PRUPE_ppa021165mg	NCBI_Assembly:GCF_000346465.:	NW_006760324.1	-	19286872	19291655	4783	-	-	GO:0003924:GTPase activity;GO:0005525:GTP binding	-

ppe-miR399a	18785911	PRUPE_ppa019337mg	NCBI_Assembly:GCF_000346465.:	NW_006760324.1	+	2778225	2779334	1109	-	GO:0006355:regulation of transcription, DNA-templated	GO:0003690: double-stranded DNA binding	GO:0005739:mitochondrion
ppe-miR399a	18786035	PRUPE_ppa016717mg	NCBI_Assembly:GCF_000346465.:	NW_006760324.1	-	7439646	7441436	1790	-	GO:0006629:lipid metabolic process	GO:0016627: oxidoreductase activity, acting on the CH-CH group of donors	GO:0005737:cytoplasm;GO:0016021:integral component of membrane
ppe-miR399a	18786155	PRUPE_ppa011741mg	NCBI_Assembly:GCF_000346465.:	NW_006760324.1	-	2384047	2385405	1358	-	-	GO:0016491: oxidoreductase activity	-
ppe-miR399a	18786313	PRUPE_ppa004292mg	NCBI_Assembly:GCF_000346465.:	NW_006760324.1	+	25992485	25996225	3740	-	-	GO:0003676: nucleic acid binding;GO:0008408:3'-5' exonuclease activity	-
ppe-miR399a	18786333	PRUPE_ppa003681mg	NCBI_Assembly:GCF_000346465.:	NW_006760324.1	+	25477115	25481676	4561	-	-	-	-
ppe-miR399a	18786407	PRUPE_ppa019822mg	NCBI_Assembly:GCF_000346465.:	NW_006760324.1	+	12490051	12491311	1260	-	-	-	-
ppe-miR399a	18786649	PRUPE_ppa002407mg	NCBI_Assembly:GCF_000346465.:	NW_006760324.1	+	1204329	1217078	12749	pper03440:Homologous recombination	GO:0006259: DNA metabolic process	GO:0003677: DNA binding;GO:0004518:nuclease activity	-
ppe-miR399a	18786680	PRUPE_ppa000665mg	NCBI_Assembly:GCF_000346465.:	NW_006760324.1	+	697082	707398	10316	pper04141:Protein processing in endoplasmic reticulum	-	-	-
ppe-miR399a	18786683	PRUPE_ppa001153m2g	NCBI_Assembly:GCF_000346465.:	NW_006760324.1	+	4202330	4203255	925	-	-	-	-
ppe-miR399a	18786739	PRUPE_ppa023113mg	NCBI_Assembly:GCF_000346465.:	NW_006760324.1	-	5898576	5903275	4699	-	-	GO:0003676: nucleic acid binding	-
ppe-miR399a	18787313	PRUPE_ppa009190mg	NCBI_Assembly:GCF_000346465.:	NW_006760324.1	-	24964812	24968070	3258	-	-	-	-
ppe-miR399a	18787356	PRUPE_ppa002945mg	NCBI_Assembly:GCF_000346465.:	NW_006760324.1	+	20913904	20919665	5761	-	GO:0005975: carbohydrate metabolic process	GO:0016868:intramolecular transferase activity, phosphotransferases	-

ppe-miR399a	18787417	PRUPE_ppa002592mg	NCBI_Assembly:GCF_000346465.:	NW_006760324.1	+	25494310	25498933	4623	pper01100:Metabolic pathways;pper01110:Biosynthesis of secondary metabolites;pper01230:Biosynthesis of amino acids;pper00230:Purine metabolism;pper01200:Carbon metabolism;pper00010:Glycolysis / Gluconeogenesis;pper00620:Pyruvate metabolism	GO:0006096:glycolytic process;GO:0006633:fatty acid biosynthetic process;GO:0010431:seed maturation	GO:0000287:magnesium ion binding;GO:0004743:pyruvate kinase activity;GO:0030955:potassium ion binding	-
ppe-miR399a	18787471	PRUPE_ppa009244mg	NCBI_Assembly:GCF_000346465.:	NW_006760324.1	+	18279896	18281743	1847	-	GO:0006109:regulation of carbohydrate metabolic process	-	-
ppe-miR399a	18787533	PRUPE_ppa005325mg	NCBI_Assembly:GCF_000346465.:	NW_006760324.1	+	26282617	26284194	1577	-	-	-	-
ppe-miR399a	18788040	PRUPE_ppa000841mg	NCBI_Assembly:GCF_000346465.:	NW_006760384.1	-	178324	185505	7181	pper03060:Protein export	GO:0006605:protein targeting;GO:0017038:protein import	GO:0005524:ATP binding	GO:0005622:intracellular;GO:0016020:membrane

ppe-miR399a	18788054	PRUPE_ppa008829mg	NCBI_Assembly:GCF_000346465.:	NW_006760384.1	+	389307	396965	7658	pper01100:Metabolic pathways;pper00061:Fatty acid biosynthesis;pper01212:Fatty acid metabolism;pper00780:Biotin metabolism;pper01040:Biosynthesis of unsaturated fatty acids	GO:0006633:fatty acid biosynthetic process	GO:0004316:3-oxoacyl-[acyl-carrier-protein] reductase (NADPH) activity;GO:0051287:NAD binding	-
ppe-miR399a	18788117	PRUPE_ppa003906mg	NCBI_Assembly:GCF_000346465.:	NW_006760385.1	+	36733550	36737291	3741	-	GO:0000956:nuclear-transcribed mRNA catabolic process	GO:0004176:ATP-dependent peptidase activity	-
ppe-miR399a	18788159	PRUPE_ppa022414mg	NCBI_Assembly:GCF_000346465.:	NW_006760385.1	-	33690398	33690652	254	-	-	GO:0005506:iron ion binding;GO:0016705:oxidoreductase activity, acting on paired donors, with incorporation or reduction of molecular oxygen;GO:0020037:heme binding	-
ppe-miR399a	18788302	PRUPE_ppa017874mg	NCBI_Assembly:GCF_000346465.:	NW_006760385.1	+	3098959	3099875	916	-	-	-	-

ppe-miR399a	18788365	PRUPE_ppa020838mg	NCBI_Assembly:GCF_000346465.:	NW_006760385.1	+	12867476	12871449	3973	pper03440:Homologous recombination;pper03450:Non-homologous end-joining	GO:0000723:telomere maintenance;GO:0006281:DNA repair	GO:0016887:ATPase activity	GO:0030870:Mre11 complex
ppe-miR399a	18788367	PRUPE_ppa003919mg	NCBI_Assembly:GCF_000346465.:	NW_006760385.1	-	44587241	44593596	6355	-	-	-	-
ppe-miR399a	18788393	PRUPE_ppa001230mg	NCBI_Assembly:GCF_000346465.:	NW_006760385.1	+	2474700	2479979	5279	pper04120:Ubiquitin mediated proteolysis	GO:0006511:ubiquitin-dependent protein catabolic process;GO:0007267:cell-cell signaling;GO:0007276:gamete generation;GO:0009561:megagametogenesis;GO:0009616:virus induced gene silencing;GO:0010267:production of ta-siRNAs involved in RNA interference;GO:0032875:regulation of DNA endoreduplication;GO:0035196:production of miRNAs involved in gene silencing by miRNA;GO:0051302:regulation of cell division;GO:0051510:regula	-	GO:0005819:spindle

ppe-miR399a	18788401	PRUPE_ppa004685mg	NCBI_Assembly:GCF_000346465.:	NW_006760385.1	-	10036791	10039515	2724	pper01100:Metabolic pathways;pper01110:Biosynthesis of secondary metabolites;pper00280:Valine, leucine and isoleucine degradation	GO:0009750:response to fructose	GO:0004147:dihydrolipoamide branched chain acyltransferase activity;GO:0008270:zinc ion binding;GO:0016407:acetyltransferase activity	-
ppe-miR399a	18788464	PRUPE_ppa024080mg	NCBI_Assembly:GCF_000346465.:	NW_006760385.1	-	27083790	27084839	1049	-	-	GO:0003677:DNA binding	-
ppe-miR399a	18788468	PRUPE_ppa025170mg	NCBI_Assembly:GCF_000346465.:	NW_006760385.1	-	26600360	26602194	1834	-	-	GO:0003676:nucleic acid binding	-
ppe-miR399a	18788532	PRUPE_ppa001556mg	NCBI_Assembly:GCF_000346465.:	NW_006760385.1	-	30673687	30676962	3275	pper01100:Metabolic pathways;pper00500:Starch and sucrose metabolism	GO:0005992:trehalose biosynthetic process	GO:0003825:alpha,alpha-trehalose-phosphate synthase (UDP-forming) activity;GO:0004805:trehalose-phosphatase activity	-
ppe-miR399a	18788543	PRUPE_ppa013194mg	NCBI_Assembly:GCF_000346465.:	NW_006760385.1	-	40822038	40823961	1923	-	GO:0006661:phosphatidylinositol biosynthetic process	-	-
ppe-miR399a	18788599	PRUPE_ppa024089mg	NCBI_Assembly:GCF_000346465.:	NW_006760385.1	-	16436803	16438448	1645	-	-	GO:0005351:sugar:proton symporter activity	GO:0000139:Golgi membrane;GO:0016021:integral component of membrane

ppe-miR399a	18788684	PRUPE_ppa023077mg	NCBI_Assembly:GCF_000346465.:	NW_006760385.1	-	35308331	35311476	3145	-	GO:0006351:transcription, DNA-templated	GO:0003677:DNA binding;GO:0003700:sequence-specific DNA binding transcription factor activity	GO:0005634:nucleus
ppe-miR399a	18788842	PRUPE_ppa018888mg	NCBI_Assembly:GCF_000346465.:	NW_006760385.1	-	15097060	15097413	353	-	-	-	-
ppe-miR399a	18788907	PRUPE_ppa000500mg	NCBI_Assembly:GCF_000346465.:	NW_006760385.1	+	1541620	1548811	7191	-	-	GO:0004672:protein kinase activity;GO:0005524:ATP binding	-
ppe-miR399a	18789001	PRUPE_ppa011898mg	NCBI_Assembly:GCF_000346465.:	NW_006760385.1	-	6020541	6022780	2239	pper03010:Ribosome	GO:0006412:translation	GO:0003735:structural constituent of ribosome	GO:0005840:ribosome
ppe-miR399a	18789073	PRUPE_ppa001428mg	NCBI_Assembly:GCF_000346465.:	NW_006760385.1	-	37359061	37367671	8610	-	GO:0006265:DNA topological change	GO:0003677:DNA binding;GO:0003917:DNA topoisomerase type I activity	GO:0005694:chromosome
ppe-miR399a	18789298	PRUPE_ppa022234mg	NCBI_Assembly:GCF_000346465.:	NW_006760385.1	+	46468639	46469611	972	-	-	-	-
ppe-miR399a	18789429	PRUPE_ppa007861mg	NCBI_Assembly:GCF_000346465.:	NW_006760385.1	+	29875423	29877566	2143	-	-	-	-
ppe-miR399a	18789629	PRUPE_ppa001869mg	NCBI_Assembly:GCF_000346465.:	NW_006760385.1	+	39783923	39791108	7185	-	-	-	-
ppe-miR399a	18789716	PRUPE_ppa004472mg	NCBI_Assembly:GCF_000346465.:	NW_006760385.1	+	33406639	33409102	2463	-	-	GO:0003824:catalytic activity	-
ppe-miR399a	18789911	PRUPE_ppa017762mg	NCBI_Assembly:GCF_000346465.:	NW_006760385.1	+	34833777	34835208	1431	-	-	-	-
ppe-miR399a	18789935	PRUPE_ppa018234mg	NCBI_Assembly:GCF_000346465.:	NW_006760385.1	-	7140182	7141572	1390	-	-	GO:0016788:hydrolase activity, acting on ester bonds	-
ppe-miR399a	18789940	PRUPE_ppa008967mg	NCBI_Assembly:GCF_000346465.:	NW_006760385.1	+	26921892	26923669	1777	-	-	-	-
ppe-miR399a	18789966	PRUPE_ppa002376mg	NCBI_Assembly:GCF_000346465.:	NW_006760385.1	-	35247920	35254015	6095	-	-	-	GO:0016020:membrane
ppe-miR399a	18790009	PRUPE_ppa025518mg	NCBI_Assembly:GCF_000346465.:	NW_006760385.1	-	5838336	5839922	1586	-	-	-	-

ppe-miR399a	18790242	PRUPE_ppa005391mg	NCBI_Assembly:GCF_000346465.:	NW_006760385.1	-	8329296	8331805	2509	-	GO:0005975:carbohydrate metabolic process;GO:0071555:cell wall organization	GO:0004650:polygalacturonase activity	GO:0005576:extracellular region
ppe-miR399a	18790338	PRUPE_ppa000691mg	NCBI_Assembly:GCF_000346465.:	NW_006760385.1	-	42839038	42846724	7686	pper00970:Aminoacyl-tRNA biosynthesis	GO:0006438:valyl-tRNA aminoacylation	GO:0002161:aminoacyl-tRNA editing activity;GO:0004832:valine-tRNA ligase activity;GO:0005524:ATP binding	-
ppe-miR399a	18790341	PRUPE_ppa006995mg	NCBI_Assembly:GCF_000346465.:	NW_006760385.1	+	14208855	14211238	2383	pper01100:Metabolic pathways;pper01110:Biosynthesis of secondary metabolites;pper00906:Carotenoid biosynthesis	GO:0016120:carotene biosynthetic process	GO:0090471:9,15,9'-tri-cis-zeta-carotene isomerase activity	-
ppe-miR399a	18790439	PRUPE_ppa003505mg	NCBI_Assembly:GCF_000346465.:	NW_006760385.1	+	44713126	44720164	7038	-	-	-	-
ppe-miR399a	18790480	PRUPE_ppa010453mg	NCBI_Assembly:GCF_000346465.:	NW_006760385.1	-	36952563	36953549	986	-	GO:0009736:cytokinin-activated signaling pathway	GO:0005215:transporter activity	GO:0009705:plant-type vacuole membrane;GO:0016021:integral component of membrane;GO:0042807:central vacuole
ppe-miR399a	18790491	PRUPE_ppa010592mg	NCBI_Assembly:GCF_000346465.:	NW_006760385.1	-	18458042	18466798	8756	-	-	GO:0003677:DNA binding;GO:0003682:chromatin binding	-

ppe-miR399a	18790606	PRUPE_ppa003541mg	NCBI_Assembly:GCF_000346465.:	NW_006760385.1	-	33734171	33737797	3626	-	GO:0006351:transcription, DNA-templated;GO:0006355:regulation of transcription, DNA-templated	GO:0003677: DNA binding	GO:0005634:nucleus
ppe-miR399a	18790677	PRUPE_ppa013789mg	NCBI_Assembly:GCF_000346465.:	NW_006760385.1	+	9067543	9068055	512	-	-	-	-
ppe-miR399a	18790782	PRUPE_ppa026515mg	NCBI_Assembly:GCF_000346465.:	NW_006760385.1	+	30997427	30997633	206	-	-	-	-
ppe-miR399a	18790788	PRUPE_ppa020634mg	NCBI_Assembly:GCF_000346465.:	NW_006760385.1	-	40664093	40665852	1759	-	-	GO:0003950: NAD+ ADP-ribosyltransferase activity;GO:0046872:metal ion binding	-
ppe-miR399a	18790824	PRUPE_ppa004754mg	NCBI_Assembly:GCF_000346465.:	NW_006760385.1	+	46775224	46777901	2677	-	-	GO:0015238: drug transmembrane transporter activity;GO:0015297:antiporter activity	GO:0016021:integral component of membrane
ppe-miR399a	18790861	PRUPE_ppa016648mg	NCBI_Assembly:GCF_000346465.:	NW_006760385.1	+	46708606	46709014	408	-	-	-	-
ppe-miR399a	18790863	PRUPE_ppa002677mg	NCBI_Assembly:GCF_000346465.:	NW_006760385.1	+	249809	256506	6697	-	-	-	-
ppe-miR399a	18790913	PRUPE_ppa003015mg	NCBI_Assembly:GCF_000346465.:	NW_006760385.1	+	13754849	13761403	6554	-	-	-	-
ppe-miR399a	18790959	PRUPE_ppa017683mg	NCBI_Assembly:GCF_000346465.:	NW_006760385.1	-	36371502	36375932	4430	-	-	GO:0004674: protein serine/threonine kinase activity;GO:0005524:ATP binding	-

ppe-miR399a	18790971	PRUPE_ppa014659mg	NCBI_Assembly:GCF_000346465.:	NW_006760385.1	+	34410399	34413972	3573	-	-	GO:0004930:G-protein coupled receptor activity;GO:0004970:ionotropic glutamate receptor activity	GO:0016021:integral component of membrane
ppe-miR399a	18791056	PRUPE_ppa018418mg	NCBI_Assembly:GCF_000346465.:	NW_006760385.1	-	4133469	4133906	437	-	-	-	-
ppe-miR399a	18791084	PRUPE_ppa024067mg	NCBI_Assembly:GCF_000346465.:	NW_006760385.1	+	12022815	12024340	1525	-	-	-	-
ppe-miR399a	18791097	PRUPE_ppa003140mg	NCBI_Assembly:GCF_000346465.:	NW_006760385.1	-	3339719	3342360	2641	-	-	-	-
ppe-miR399a	18791099	PRUPE_ppa027076mg	NCBI_Assembly:GCF_000346465.:	NW_006760385.1	+	34223590	34225449	1859	-	-	-	-

ppe-miR399a	18791101	PRUPE_ppa000475mg	NCBI_Assembly:GCF_000346465.:	NW_006760385.1	+	3514047	3527066	13019	-	GO:0000278:mitotic cell cycle;GO:0000724:double-strand break repair via homologous recombination;GO:0006261:DNA-dependent DNA replication;GO:0006275:regulation of DNA replication;GO:0006298:mismatch repair;GO:0006306:DNA methylation;GO:0006342:chromatin silencing;GO:0009408:response to heat;GO:0009555:pollen development;GO:0016444:somatic cell DNA recombination;GO:0016572:histone phosphorylation;GO:0031047:gene silencing by	GO:0005524:ATP binding;GO:0030983:mismatched DNA binding	GO:0005739:mitochondrion
ppe-miR399a	18791112	PRUPE_ppa003189mg	NCBI_Assembly:GCF_000346465.:	NW_006760385.1	-	44118136	44120327	2191	-	-	-	-

ppe-miR399a	18791177	PRUPE_ppa015860mg	NCBI_Assembly:GCF_000346465.:	NW_006760385.1	+	8877282	8878607	1325	pper04075:Plant hormone signal transduction	GO:0006351:transcription, DNA-templated;GO:0006355:regulation of transcription, DNA-templated	-	-
ppe-miR399a	18791202	PRUPE_ppa005424mg	NCBI_Assembly:GCF_000346465.:	NW_006760385.1	-	4619511	4622941	3430	-	-	-	-
ppe-miR399a	18791222	PRUPE_ppa005767mg	NCBI_Assembly:GCF_000346465.:	NW_006760385.1	+	38724616	38726517	1901	-	-	-	-
ppe-miR399a	18791466	PRUPE_ppa017016mg	NCBI_Assembly:GCF_000346465.:	NW_006760385.1	-	41433396	41433701	305	-	-	-	-
ppe-miR399a	18791496	PRUPE_ppa025864mg	NCBI_Assembly:GCF_000346465.:	NW_006760385.1	-	18557774	18561747	3973	-	-	GO:0003676: nucleic acid binding	-
ppe-miR399a	18791594	PRUPE_ppa021918mg	NCBI_Assembly:GCF_000346465.:	NW_006760385.1	+	36067677	36069915	2238	-	GO:0010047:fruit dehiscence	GO:0003700: sequence-specific DNA binding transcription factor activity	-
ppe-miR399a	18791618	PRUPE_ppa022573mg	NCBI_Assembly:GCF_000346465.:	NW_006760385.1	+	26726666	26727094	428	-	-	-	-
ppe-miR399a	18791760	PRUPE_ppa006790mg	NCBI_Assembly:GCF_000346465.:	NW_006760385.1	+	33956580	33958440	1860	-	-	-	-
ppe-miR399a	18792013	PRUPE_ppa010030mg	NCBI_Assembly:GCF_000346465.:	NW_006760385.1	-	5875523	5880359	4836	pper03030:DNA replication;pper03420:Nucleotide excision repair;pper03430:Mismatch repair;pper03440:Homologous recombination	GO:0006260: DNA replication;GO:0006281:DNA repair;GO:0006310:DNA recombination	GO:0003677: DNA binding	GO:0005634:nucleus

ppe-miR399a	18792035	PRUPE_ppa009028mg	NCBI_Assembly:GCF_000346465.:	NW_006760385.1	+	32103751	32107253	3502	-	GO:0007031: peroxisome organization; GO:0009407:toxin catabolic process;GO:0010260:organ senescence;GO:0033542:fatty acid beta-oxidation, unsaturated, even number;GO:0043161:proteasome-mediated ubiquitin-dependent protein catabolic process;GO:0051788:response to misfolded protein;GO:0080129:proteasome core complex assembly	GO:0080023: 3R-hydroxyacyl-CoA dehydratase activity	-
ppe-miR399a	18792036	PRUPE_ppa024963mg	NCBI_Assembly:GCF_000346465.:	NW_006760385.1	-	44312622	44317310	4688	-	GO:0007165: signal transduction	GO:0043531: ADP binding	-
ppe-miR399a	18792060	PRUPE_ppa005599mg	NCBI_Assembly:GCF_000346465.:	NW_006760385.1	-	9620826	9623551	2725	-	GO:0005975: carbohydrate metabolic process;GO:0071555:cell wall organization	GO:0004650: polygalacturonase activity	GO:0005576:extracellular region
ppe-miR399a	18792062	PRUPE_ppa016319mg	NCBI_Assembly:GCF_000346465.:	NW_006760385.1	-	11693617	11694684	1067	-	-	-	-

ppe-miR399a	18792067	PRUPE_ppa001698mg	NCBI_Assembly:GCF_000346465.:	NW_006760385.1	-	24750067	24755835	5768	pper00350:Tyrosine metabolism;pper00360:Phenylalanine metabolism;pper00950:Isoquinoline alkaloid biosynthesis;pper00960:Tropane, piperidine and pyridine alkaloid biosynthesis;pper01100:Metabolic pathways;pper01110:Biosynthesis of secondary metabolites;pper00260:Glycine, serine and threonine metabolism;pper00410:beta-Alanine metabolism	GO:0009308: amine metabolic process	GO:0005507: copper ion binding;GO:0008131:primary amine oxidase activity;GO:0048038:quinone binding	-
ppe-miR399a	18792080	PRUPE_ppa018375mg	NCBI_Assembly:GCF_000346465.:	NW_006760385.1	+	12997348	13000030	2682	-	-	-	-
ppe-miR399a	18792100	PRUPE_ppa023243mg	NCBI_Assembly:GCF_000346465.:	NW_006760385.1	+	39007925	39009219	1294	-	-	-	-
ppe-miR399a	18792203	PRUPE_ppa018087mg	NCBI_Assembly:GCF_000346465.:	NW_006760385.1	-	18342427	18343505	1078	-	-	-	-
ppe-miR399a	18792301	PRUPE_ppa013309mg	NCBI_Assembly:GCF_000346465.:	NW_006760385.1	+	36339974	36340716	742	-	-	-	-
ppe-miR399a	18792677	PRUPE_ppa011114mg	NCBI_Assembly:GCF_000346465.:	NW_006760385.1	-	38206915	38208341	1426	-	-	-	-
ppe-miR399a	18792694	PRUPE_ppa015719mg	NCBI_Assembly:GCF_000346465.:	NW_006760385.1	+	25760642	25762397	1755	-	-	GO:0022857:transmembrane transporter activity	GO:0016021:integral component of membrane
ppe-miR399a	18792695	PRUPE_ppa020849mg	NCBI_Assembly:GCF_000346465.:	NW_006760385.1	-	29516759	29517559	800	-	-	-	-

ppe-miR399a	18792765	PRUPE_ppa018001mg	NCBI_Assembly:GCF_000346465.:	NW_006760385.1	+	19038888	19040659	1771	-	-	GO:0003676: nucleic acid binding;GO:0004523:RNA-DNA hybrid ribonuclease activity	-
ppe-miR399a	18792792	PRUPE_ppa020612mg	NCBI_Assembly:GCF_000346465.:	NW_006760385.1	+	38709527	38712125	2598	-	-	GO:0003676: nucleic acid binding	-
ppe-miR399a	18792844	PRUPE_ppa011688mg	NCBI_Assembly:GCF_000346465.:	NW_006760385.1	-	38232261	38233165	904	-	-	-	-
ppe-miR399a	18792951	PRUPE_ppa000234mg	NCBI_Assembly:GCF_000346465.:	NW_006760385.1	-	38257482	38264376	6894	-	-	GO:0005524: ATP binding;GO:0016887:ATPase activity	GO:0016020:membrane
ppe-miR399a	18793041	PRUPE_ppa001871mg	NCBI_Assembly:GCF_000346465.:	NW_006760385.1	-	23767436	23770997	3561	-	GO:0000956: nuclear-transcribed mRNA catabolic process;GO:0009887:organ morphogenesis;GO:0009888:tissue development;GO:0010638: positive regulation of organelle organization;GO:0033044:regulation of chromosome organization	GO:0004672: protein kinase activity;GO:0005524:ATP binding	GO:0005829:cytosol
ppe-miR399a	18793062	PRUPE_ppa026517mg	NCBI_Assembly:GCF_000346465.:	NW_006760385.1	-	5153876	5155194	1318	-	-	-	-

ppe-miR399a	18793151	PRUPE_ppa020400mg	NCBI_Assembly:GCF_000346465.:	NW_006760385.1	+	8590084	8590878	794	-	-	GO:0004857: enzyme inhibitor activity;GO:0030599:pectinesterase activity	-
ppe-miR399a	18793153	PRUPE_ppb016944mg	NCBI_Assembly:GCF_000346465.:	NW_006760385.1	-	33687777	33688046	269	-	-	-	-
ppe-miR399a	18793172	PRUPE_ppa002197mg	NCBI_Assembly:GCF_000346465.:	NW_006760385.1	-	44434918	44447942	13024	pper03430:Mismatch repair	GO:0000278: mitotic cell cycle;GO:0000724:double-strand break repair via homologous recombination;GO:0006261: DNA-dependent DNA replication;GO:0006275:regulation of DNA replication;GO:0006298:mismatch repair;GO:0006306:DNA methylation;GO:0006312:mitotic recombination;GO:0007062: sister chromatid cohesion;GO:0007129:synapsis;GO:0009555:pollen development;GO:0009560: embryo sac egg cell differentiation;GO:0009737:response to abscisic	GO:0005524: ATP binding;GO:0030983:mismatched DNA binding	GO:0000790:nuclear chromatin;GO:0032300:mismatch repair complex

ppe-miR399a	18793242	PRUPE_ppb024512mg	NCBI_Assembly:GCF_000346465.:	NW_006760385.1	-	3060756	3061791	1035	-	-	-	-
ppe-miR399a	18793251	PRUPE_ppa016338mg	NCBI_Assembly:GCF_000346465.:	NW_006760385.1	-	7369988	7370474	486	-	-	-	-
ppe-miR399a	18793314	PRUPE_ppa003848mg	NCBI_Assembly:GCF_000346465.:	NW_006760385.1	+	45611355	45614316	2961	pper01100:Metabolic pathways;pper01110:Biosynthesis of secondary metabolites;pper00860:Porphyrin and chlorophyll metabolism	GO:0006782: protoporphyrinogen IX biosynthetic process	GO:0008883: glutamyl-tRNA reductase activity;GO:0050661:NADP binding	-
ppe-miR399a	18793423	PRUPE_ppa003359mg	NCBI_Assembly:GCF_000346465.:	NW_006760385.1	+	44737406	44740663	3257	-	-	GO:0005524: ATP binding;GO:0019136:deoxynucleoside kinase activity	GO:0005634:nucleus
ppe-miR399a	18793490	PRUPE_ppa011790mg	NCBI_Assembly:GCF_000346465.:	NW_006760385.1	-	30794823	30795810	987	-	GO:0055114: oxidation-reduction process	-	GO:0016021:integral component of membrane
ppe-miR399a	18793609	PRUPE_ppa016563mg	NCBI_Assembly:GCF_000346465.:	NW_006760385.1	-	36508257	36511249	2992	-	-	GO:0003676: nucleic acid binding	-
ppe-miR399a	18793635	PRUPE_ppa022966mg	NCBI_Assembly:GCF_000346465.:	NW_006760385.1	-	21718488	21719918	1430	-	-	-	-
ppe-miR399a	18793710	PRUPE_ppa015844mg	NCBI_Assembly:GCF_000346465.:	NW_006760385.1	+	36403828	36405769	1941	-	GO:0016042:lipid catabolic process	GO:0016787: hydrolase activity	-
ppe-miR399a	18793735	PRUPE_ppa004116mg	NCBI_Assembly:GCF_000346465.:	NW_006760385.1	+	10744696	10747638	2942	-	GO:0000272: polysaccharide catabolic process	GO:0016161: beta-amylase activity	-
ppe-miR399a	18793740	PRUPE_ppa020641mg	NCBI_Assembly:GCF_000346465.:	NW_006760385.1	+	26453464	26453872	408	-	-	-	-
ppe-miR399a	18793850	PRUPE_ppa010429mg	NCBI_Assembly:GCF_000346465.:	NW_006760385.1	+	36196774	36199340	2566	-	-	-	-
ppe-miR399a	18793871	PRUPE_ppa013143mg	NCBI_Assembly:GCF_000346465.:	NW_006760385.1	+	30301278	30302565	1287	-	-	-	-

ppe-miR399a	18793885	PRUPE_ppa008849mg	NCBI_Assembly:GCF_000346465.:	NW_006760385.1	-	41798530	41799645	1115	-	GO:0009827: plant-type cell wall modification;GO:0009860:pollen tube growth;GO:0030048:actin filament-based movement	GO:0005509: calcium ion binding;GO:0005544:calcium-dependent phospholipid binding	-
ppe-miR399a	18793931	PRUPE_ppa006751mg	NCBI_Assembly:GCF_000346465.:	NW_006760385.1	-	31408171	31410540	2369	pper00130:Ubiquinone and other terpenoid-quinone biosynthesis;pper01100:Metabolic pathways;pper01110:Biosynthesis of secondary metabolites	GO:0006744: ubiquinone biosynthetic process	GO:0004659: prenyltransferase activity	GO:0016021:integral component of membrane
ppe-miR399a	18793969	PRUPE_ppa002916mg	NCBI_Assembly:GCF_000346465.:	NW_006760385.1	-	32521063	32525475	4412	-	-	-	-
ppe-miR399a	18794004	PRUPE_ppa001332mg	NCBI_Assembly:GCF_000346465.:	NW_006760385.1	+	30333415	30337496	4081	-	-	-	GO:0009570:chloroplast stroma
ppe-miR399a	18794009	PRUPE_ppa015515mg	NCBI_Assembly:GCF_000346465.:	NW_006760385.1	+	14500303	14501572	1269	-	-	GO:0003676: nucleic acid binding	-
ppe-miR399a	18794025	PRUPE_ppa010120mg	NCBI_Assembly:GCF_000346465.:	NW_006760385.1	-	1238017	1238975	958	-	-	-	-
ppe-miR399b	18766091	PRUPE_ppa017187mg	NCBI_Assembly:GCF_000346465.:	NW_006760186.1	+	1860450	1860692	242	-	-	-	-
ppe-miR399b	18766096	PRUPE_ppa020671mg	NCBI_Assembly:GCF_000346465.:	NW_006760186.1	-	1738757	1743688	4931	-	GO:0015074: DNA integration	GO:0003676: nucleic acid binding;GO:0008270:zinc ion binding	GO:0005634:nucleus
ppe-miR399b	18766337	PRUPE_ppa011090mg	NCBI_Assembly:GCF_000346465.:	NW_006760194.1	+	16341926	16345567	3641	-	GO:0006457: protein folding	GO:0003755: peptidyl-prolyl cis-trans isomerase activity	GO:0005768:endosome;GO:0005802:trans-Golgi network

ppe-miR399b	18766515	PRUPE_ppa012371mg	NCBI_Assembly:GCF_000346465.:	NW_006760194.1	+	12424589	12425930	1341	pper01100:Metabolic pathways;pper00230:Purine metabolism;pper03008:Ribosome biogenesis in eukaryotes	-	GO:0004017:adenylate kinase activity;GO:0005524:ATP binding;GO:0016887:ATPase activity	GO:0005634:nucleus
ppe-miR399b	18766527	PRUPE_ppa015566mg	NCBI_Assembly:GCF_000346465.:	NW_006760194.1	-	14931785	14933569	1784	-	-	-	-
ppe-miR399b	18766643	PRUPE_ppa002034mg	NCBI_Assembly:GCF_000346465.:	NW_006760194.1	-	10890815	10893524	2709	pper01100:Metabolic pathways;pper00330:Arginine and proline metabolism	GO:0006527:arginine catabolic process;GO:0008295:spermidine biosynthetic process	GO:0008792:arginine decarboxylase activity	-
ppe-miR399b	18767147	PRUPE_ppa002883mg	NCBI_Assembly:GCF_000346465.:	NW_006760194.1	-	19519468	19521697	2229	-	GO:0006457:protein folding;GO:0009220:pyrimidine ribonucleotide biosynthetic process;GO:0009408:response to heat;GO:0009644:response to high light intensity;GO:0034976:response to endoplasmic reticulum stress;GO:0042542:response to hydrogen peroxide	-	-
ppe-miR399b	18767187	PRUPE_ppa011712mg	NCBI_Assembly:GCF_000346465.:	NW_006760194.1	+	19855629	19856383	754	-	-	-	-

ppe-miR399b	18767217	PRUPE_ppa020853mg	NCBI_Assembly:GCF_000346465.:	NW_006760194.1	-	13320021	13320245	224	-	GO:0006412:translation	GO:0003735:structural constituent of ribosome;GO:0019843:rRNA binding	GO:0005840:ribosome;GO:0009507:chloroplast
ppe-miR399b	18767959	PRUPE_ppa009713mg	NCBI_Assembly:GCF_000346465.:	NW_006760194.1	-	13246149	13248933	2784	-	GO:0006635:fatty acid beta-oxidation	-	-
ppe-miR399b	18768048	PRUPE_ppa003473mg	NCBI_Assembly:GCF_000346465.:	NW_006760194.1	+	1567423	1572066	4643	pper01100:Metabolic pathways;pper01110:Biosynthesis of secondary metabolites;pper00970:Aminoacyl-tRNA biosynthesis;pper00860:Porphyrin and chlorophyll metabolism	GO:0006424:glutamyl-tRNA aminoacylation;GO:0007005:mitochondrion organization;GO:0009658:chloroplast organization;GO:0048481:ovule development	GO:0000049:tRNA binding;GO:0004818:glutamate-tRNA ligase activity;GO:0005524:ATP binding	GO:0009570:chloroplast stroma
ppe-miR399b	18768186	PRUPE_ppa017453mg	NCBI_Assembly:GCF_000346465.:	NW_006760194.1	-	8500470	8502333	1863	-	-	GO:0003676:nucleic acid binding	-
ppe-miR399b	18768337	PRUPE_ppa001597mg	NCBI_Assembly:GCF_000346465.:	NW_006760194.1	+	17480655	17483482	2827	-	-	GO:0008270:zinc ion binding	-
ppe-miR399b	18768343	PRUPE_ppa009673mg	NCBI_Assembly:GCF_000346465.:	NW_006760194.1	+	21310994	21313159	2165	-	-	-	-

ppe-miR399b	18768528	PRUPE_ppa015646mg	NCBI_Assembly:GCF_000346465.:	NW_006760194.1	+	7728756	7731632	2876	-	GO:0000023: maltose metabolic process;GO:0006655:phosphatidylglycerol biosynthetic process;GO:0009902:chloroplast relocation;GO:0010027:thylakoid membrane organization; GO:0019252: starch biosynthetic process;GO:0019288:isopentenyl diphosphate biosynthetic process, methylerythritol 4-phosphate pathway;GO:0034660:ncRNA metabolic process	GO:0005525: GTP binding	GO:0005623:cell

ppe-miR399b	18768579	PRUPE_ppa008035mg	NCBI_Assembly:GCF_000346465.:	NW_006760194.1	+	16315289	16316870	1581	-	GO:0009664: plant-type cell wall organization; GO:0010075:regulation of meristem growth;GO:0042545:cell wall modification;GO:0048653:anther development	-	GO:0031225:anchored component of membrane
ppe-miR399b	18768595	PRUPE_ppa006074mg	NCBI_Assembly:GCF_000346465.:	NW_006760194.1	+	15952911	15957113	4202	-	-	-	-
ppe-miR399b	18768647	PRUPE_ppa017756mg	NCBI_Assembly:GCF_000346465.:	NW_006760194.1	-	21099469	21102398	2929	-	-	GO:0003677: DNA binding;GO:0003682:chromatin binding	GO:0005634:nucleus
ppe-miR399b	18768843	PRUPE_ppa026667mg	NCBI_Assembly:GCF_000346465.:	NW_006760194.1	+	7121654	7123480	1826	-	-	-	-
ppe-miR399b	18768929	PRUPE_ppa008027mg	NCBI_Assembly:GCF_000346465.:	NW_006760194.1	-	1775362	1778511	3149	-	-	GO:0004185: serine-type carboxypeptidase activity	-
ppe-miR399b	18769379	PRUPE_ppa005408mg	NCBI_Assembly:GCF_000346465.:	NW_006760201.1	-	17966371	17969174	2803	-	-	-	GO:0016021:integral component of membrane
ppe-miR399b	18769391	PRUPE_ppa009428mg	NCBI_Assembly:GCF_000346465.:	NW_006760201.1	+	22012232	22015165	2933	-	-	-	-
ppe-miR399b	18769525	PRUPE_ppa000956mg	NCBI_Assembly:GCF_000346465.:	NW_006760201.1	+	13135139	13139090	3951	-	-	GO:0004674: protein serine/threonine kinase activity;GO:0005524:ATP binding	GO:0016021:integral component of membrane
ppe-miR399b	18770164	PRUPE_ppa025430mg	NCBI_Assembly:GCF_000346465.:	NW_006760201.1	-	8581255	8582563	1308	-	GO:0005975: carbohydrate metabolic process	GO:0004553: hydrolase activity, hydrolyzing O-glycosyl compounds	-

ppe-miR399b	18770335	PRUPE_ppa014780mg	NCBI_Assembly:GCF_000346465.:	NW_006760201.1	-	17925799	17929103	3304	-	-	-	-
ppe-miR399b	18770361	PRUPE_ppa002594mg	NCBI_Assembly:GCF_000346465.:	NW_006760201.1	+	21110945	21113870	2925	-	-	GO:0001104: RNA polymerase II transcription cofactor activity	GO:0016592:mediator complex
ppe-miR399b	18770803	PRUPE_ppa002389mg	NCBI_Assembly:GCF_000346465.:	NW_006760201.1	+	17146994	17149546	2552	-	-	GO:0008270: zinc ion binding	-
ppe-miR399b	18771691	PRUPE_ppa011442mg	NCBI_Assembly:GCF_000346465.:	NW_006760201.1	-	21229994	21232404	2410	-	-	-	-
ppe-miR399b	18771867	PRUPE_ppa008217mg	NCBI_Assembly:GCF_000346465.:	NW_006760201.1	-	10345815	10348485	2670	-	-	-	-
ppe-miR399b	18772296	PRUPE_ppa000848mg	NCBI_Assembly:GCF_000346465.:	NW_006760208.1	+	27077220	27083449	6229	-	GO:0007033: vacuole organization; GO:0016036: cellular response to phosphate starvation;GO:0019375:galactolipid biosynthetic process;GO:0042631:cellular response to water deprivation	-	-
ppe-miR399b	18772351	PRUPE_ppa011183mg	NCBI_Assembly:GCF_000346465.:	NW_006760208.1	+	22370372	22372629	2257	-	GO:0016973: poly(A)+ mRNA export from nucleus	-	GO:0005634:nucleus
ppe-miR399b	18772432	PRUPE_ppa002341mg	NCBI_Assembly:GCF_000346465.:	NW_006760208.1	-	23833004	23835119	2115	-	-	-	-
ppe-miR399b	18772437	PRUPE_ppb024320mg	NCBI_Assembly:GCF_000346465.:	NW_006760208.1	-	7909830	7911032	1202	pper01100:Metabolic pathways;pper01110:Biosynthesis of secondary metabolites;pper00020:Citrate cycle (TCA cycle)	-	GO:0005524: ATP binding	-

ppe-miR399b	18772553	PRUPE_ppa011788mg	NCBI_Assembly:GCF_000346465.:	NW_006760208.1	+	23368045	23368880	835	-	GO:0001510:RNA methylation;GO:0009220:pyrimidine ribonucleotide biosynthetic process	-	GO:0005759:mitochondrial matrix
ppe-miR399b	18772625	PRUPE_ppa013236mg	NCBI_Assembly:GCF_000346465.:	NW_006760208.1	-	26460661	26462211	1550	-	GO:0009741:response to brassinosteroid;GO:0032880:regulation of protein localization	-	-
ppe-miR399b	18772703	PRUPE_ppa013294mg	NCBI_Assembly:GCF_000346465.:	NW_006760208.1	-	800076	802075	1999	pper01100:Metabolic pathways;pper04145:Phagosome;pper00190:Oxidative phosphorylation	GO:0015991:ATP hydrolysis coupled proton transport	GO:0046961:proton-transporting ATPase activity, rotational mechanism	GO:0033180:proton-transporting V-type ATPase, V1 domain
ppe-miR399b	18772713	PRUPE_ppa026856mg	NCBI_Assembly:GCF_000346465.:	NW_006760208.1	+	4756263	4760831	4568	-	GO:0015074:DNA integration	GO:0003676:nucleic acid binding;GO:0008270:zinc ion binding	-
ppe-miR399b	18772741	PRUPE_ppa025142mg	NCBI_Assembly:GCF_000346465.:	NW_006760208.1	-	28342941	28347177	4236	-	-	GO:0004672:protein kinase activity;GO:0005524:ATP binding	-
ppe-miR399b	18772769	PRUPE_ppa018142mg	NCBI_Assembly:GCF_000346465.:	NW_006760208.1	-	2365695	2366985	1290	pper03010:Ribosome	GO:0006412:translation	GO:0003735:structural constituent of ribosome	GO:0005840:ribosome

ppe-miR399b	18773097	PRUPE_ppa005585mg	NCBI_Assembly:GCF_000346465.:	NW_006760208.1	+	27680735	27684276	3541	-	GO:0009410:response to xenobiotic stimulus;GO:0030968:endoplasmic reticulum unfolded protein response	GO:0003700: sequence-specific DNA binding transcription factor activity;GO:0043565:sequence-specific DNA binding	-
ppe-miR399b	18773202	PRUPE_ppa006309mg	NCBI_Assembly:GCF_000346465.:	NW_006760208.1	+	20346296	20351709	5413	pper01100:Metabolic pathways;pper01110:Biosynthesis of secondary metabolites;pper01230:Biosynthesis of amino acids;pper01210:2-Oxocarboxylic acid metabolism;pper00280:Valine, leucine and isoleucine degradation;pper00290:Valine, leucine and isoleucine biosynthesis;pper00770:Pantothenate and CoA biosynthesis	GO:0009081: branched-chain amino acid metabolic process;GO:0009407:toxin catabolic process;GO:0019932:second-messenger-mediated signaling	GO:0052654: L-leucine transaminase activity;GO:0052655:L-valine transaminase activity;GO:0052656:L-isoleucine transaminase activity	GO:0009570:chloroplast stroma
ppe-miR399b	18773206	PRUPE_ppa000715mg	NCBI_Assembly:GCF_000346465.:	NW_006760208.1	+	15591507	15604682	13175	pper03008:Ribosome biogenesis in eukaryotes	-	GO:0008080: N-acetyltransferase activity	-

ppe-miR399b	18773246	PRUPE_ppa000199mg	NCBI_Assembly:GCF_000346465.:	NW_006760208.1	-	27846859	27857130	10271	-	-	GO:0003774:motor activity;GO:0005524:ATP binding	GO:0016459:myosin complex
ppe-miR399b	18773397	PRUPE_ppa003394mg	NCBI_Assembly:GCF_000346465.:	NW_006760208.1	-	27065063	27066929	1866	-	-	GO:0004674:protein serine/threonine kinase activity;GO:0005524:ATP binding	-

ppe-miR399b	18773570	PRUPE_ppa016557mg	NCBI_Assembly:GCF_000346465.:	NW_006760208.1	-	11720905	11728341	7436	-	GO:0006342: chromatin silencing;GO: 0009855:determination of bilateral symmetry;GO:0009965:leaf morphogenesis;GO:0010014:meristem initiation;GO:0010051:xylem and phloem pattern formation;GO: 0010078:maintenance of root meristem identity;GO:0010479:stele development; GO:0016569: covalent chromatin modification;GO:0031047:gene silencing by RNA;GO:0045893:positive regulation of transcription, DNA-templated;GO:0048439:flower morphogenesis	GO:0003700: sequence-specific DNA binding transcription factor activity	-
ppe-miR399b	18773612	PRUPE_ppa017005mg	NCBI_Assembly:GCF_000346465.:	NW_006760208.1	-	26386678	26387261	583	-	-	-	-
ppe-miR399b	18773668	PRUPE_ppa011071mg	NCBI_Assembly:GCF_000346465.:	NW_006760208.1	-	11522776	11523683	907	-	-	-	GO:0009506:plasmodesma
ppe-miR399b	18773760	PRUPE_ppa013652mg	NCBI_Assembly:GCF_000346465.:	NW_006760208.1	-	24305750	24306208	458	pper03040:Spliceosome	-	-	-

ppe-miR399b	18773829	PRUPE_ppa016855mg	NCBI_Assembly:GCF_000346465.:	NW_006760208.1	-	15227164	15230550	3386	pper01100:Metabolic pathways;pper01110:Biosynthesis of secondary metabolites;pper01230:Biosynthesis of amino acids;pper00460:Cyanoamino acid metabolism;pper00260:Glycine, serine and threonine metabolism;pper01200:Carbon metabolism;pper00630:Glyoxylate and dicarboxylate metabolism;pper00670:One carbon pool by folate	GO:0006544: glycine metabolic process;GO:0006563:L-serine metabolic process	GO:0004372: glycine hydroxymethyltransferase activity;GO:0030170:pyridoxal phosphate binding	-
ppe-miR399b	18773917	PRUPE_ppb012667mg	NCBI_Assembly:GCF_000346465.:	NW_006760208.1	-	23245698	23246342	644	-	-	GO:0003676: nucleic acid binding;GO:0046872:metal ion binding	-
ppe-miR399b	18774065	PRUPE_ppa000408mg	NCBI_Assembly:GCF_000346465.:	NW_006760208.1	-	8592060	8597778	5718	-	-	GO:0008270: zinc ion binding	-
ppe-miR399b	18774171	PRUPE_ppa026526m1g	NCBI_Assembly:GCF_000346465.:	NW_006760208.1	-	4240270	4240919	649	-	-	-	-
ppe-miR399b	18774275	PRUPE_ppa023542mg	NCBI_Assembly:GCF_000346465.:	NW_006760208.1	-	22107881	22108465	584	-	-	GO:0016788: hydrolase activity, acting on ester bonds	-
ppe-miR399b	18774293	PRUPE_ppa014401mg	NCBI_Assembly:GCF_000346465.:	NW_006760208.1	+	17536153	17536731	578	-	-	-	-

ppe-miR399b	18774416	PRUPE_ppa001201mg	NCBI_Assembly:GCF_000346465.:	NW_006760208.1	-	8192136	8199559	7423	-	-	GO:0008270: zinc ion binding	-
ppe-miR399b	18774504	PRUPE_ppa001899mg	NCBI_Assembly:GCF_000346465.:	NW_006760208.1	-	25628843	25632596	3753	pper04712:Circadian rhythm - plant;pper04075:Plant hormone signal transduction	GO:0007623: circadian rhythm;GO:0009630:gravitropism;GO:0009704:de-etiolation;GO:0009740:gibberellic acid mediated signaling pathway;GO:0031539:positive regulation of anthocyanin metabolic process	GO:0003700: sequence-specific DNA binding transcription factor activity	-
ppe-miR399b	18774666	PRUPE_ppa022021mg	NCBI_Assembly:GCF_000346465.:	NW_006760208.1	-	19820736	19824274	3538	-	GO:0005975: carbohydrate metabolic process	GO:0003824: catalytic activity;GO:0030246:carbohydrate binding	-
ppe-miR399b	18774724	PRUPE_ppa016872mg	NCBI_Assembly:GCF_000346465.:	NW_006760208.1	-	5674822	5675382	560	-	-	-	-
ppe-miR399b	18774790	PRUPE_ppa015372mg	NCBI_Assembly:GCF_000346465.:	NW_006760208.1	-	22546621	22549793	3172	-	-	GO:0004674: protein serine/threonine kinase activity;GO:0005524:ATP binding	-

ppe-miR399b	18774969	PRUPE_ppa018581mg	NCBI_Assembly:GCF_000346465.:	NW_006760208.1	-	19172953	19176552	3599	-	-	GO:0004672:protein kinase activity;GO:0004842:ubiquitin-protein transferase activity;GO:0005524:ATP binding;GO:0016874:ligase activity	-
ppe-miR399b	18775169	PRUPE_ppa003733mg	NCBI_Assembly:GCF_000346465.:	NW_006760208.1	-	1196389	1198575	2186	-	-	GO:0005507:copper ion binding;GO:0016491:oxidoreductase activity	-
ppe-miR399b	18775261	PRUPE_ppa012626mg	NCBI_Assembly:GCF_000346465.:	NW_006760208.1	+	21536036	21538805	2769	pper04120:Ubiquitin mediated proteolysis;pper03013:RNA transport	GO:0009737:response to abscisic acid;GO:0009793:embryo development ending in seed dormancy	GO:0016874:ligase activity;GO:0019789:SUMO transferase activity	GO:0005634:nucleus
ppe-miR399b	18775393	PRUPE_ppa013875mg	NCBI_Assembly:GCF_000346465.:	NW_006760208.1	+	9322129	9324357	2228	pper03010:Ribosome	GO:0006412:translation	GO:0003735:structural constituent of ribosome	GO:0005840:ribosome
ppe-miR399b	18775399	PRUPE_ppa005612mg	NCBI_Assembly:GCF_000346465.:	NW_006760208.1	+	25690341	25692601	2260	-	-	GO:0003824:catalytic activity;GO:0030170:pyridoxal phosphate binding	-
ppe-miR399b	18775419	PRUPE_ppa019435mg	NCBI_Assembly:GCF_000346465.:	NW_006760208.1	-	16231660	16232844	1184	-	-	GO:0003700:sequence-specific DNA binding transcription factor activity	-
ppe-miR399b	18775468	PRUPE_ppa009945mg	NCBI_Assembly:GCF_000346465.:	NW_006760208.1	-	24570029	24570988	959	-	-	-	-

ppe-miR399b	18775550	PRUPE_ppa027120mg	NCBI_Assembly:GCF_000346465.:	NW_006760208.1	-	2822212	2822713	501	-	-	-	-
ppe-miR399b	18775632	PRUPE_ppa027222mg	NCBI_Assembly:GCF_000346465.:	NW_006760208.1	+	13468907	13471200	2293	-	-	GO:0009055: electron carrier activity	-
ppe-miR399b	18775998	PRUPE_ppa003882mg	NCBI_Assembly:GCF_000346465.:	NW_006760212.1	-	12562194	12564053	1859	-	-	GO:0004497: monooxygenase activity;GO:0005506:iron ion binding;GO:0016705:oxidoreductase activity, acting on paired donors, with incorporation or reduction of molecular oxygen;GO:0020037:heme binding	-
ppe-miR399b	18776028	PRUPE_ppa001299mg	NCBI_Assembly:GCF_000346465.:	NW_006760212.1	-	3389487	3394340	4853	-	-	-	-
ppe-miR399b	18776082	PRUPE_ppa010100mg	NCBI_Assembly:GCF_000346465.:	NW_006760212.1	-	18031646	18034260	2614	-	-	GO:0008080: N-acetyltransferase activity	-
ppe-miR399b	18776219	PRUPE_ppa000755mg	NCBI_Assembly:GCF_000346465.:	NW_006760212.1	+	13213133	13219770	6637	pper00511:Other glycan degradation	GO:0006013: mannose metabolic process	GO:0004559: alpha-mannosidase activity;GO:0008270:zinc ion binding;GO:0030246:carbohydrate binding	-
ppe-miR399b	18776274	PRUPE_ppa014574mg	NCBI_Assembly:GCF_000346465.:	NW_006760212.1	+	6385614	6386161	547	-	-	-	-
ppe-miR399b	18776296	PRUPE_ppa010475mg	NCBI_Assembly:GCF_000346465.:	NW_006760212.1	-	8169268	8170621	1353	-	-	GO:0005215:transporter activity	GO:0016021:integral component of membrane

ppe-miR399b	18776394	PRUPE_ppa006897mg	NCBI_Assembly:GCF_000346465.:	NW_006760212.1	-	14003605	14005071	1466	-	-	GO:0016788: hydrolase activity, acting on ester bonds	-
ppe-miR399b	18776460	PRUPE_ppa017247mg	NCBI_Assembly:GCF_000346465.:	NW_006760212.1	+	3719840	3722850	3010	pper00970:Aminoacyl-tRNA biosynthesis	GO:0006418:tRNA aminoacylation for protein translation	GO:0004812: aminoacyl-tRNA ligase activity;GO:0005524:ATP binding	-
ppe-miR399b	18776613	PRUPE_ppa009580mg	NCBI_Assembly:GCF_000346465.:	NW_006760212.1	-	1890399	1891438	1039	-	GO:0006351:transcription, DNA-templated	GO:0003682: chromatin binding;GO:0003700:sequence-specific DNA binding transcription factor activity;GO:0043565:sequence-specific DNA binding	GO:0005634:nucleus
ppe-miR399b	18776637	PRUPE_ppa007751mg	NCBI_Assembly:GCF_000346465.:	NW_006760212.1	-	4109360	4113264	3904	-	GO:0007264: small GTPase mediated signal transduction	GO:0005525: GTP binding	GO:0005622:intracellular
ppe-miR399b	18776755	PRUPE_ppa004485mg	NCBI_Assembly:GCF_000346465.:	NW_006760212.1	+	15126674	15130650	3976	pper01100:Metabolic pathways;pper01110:Biosynthesis of secondary metabolites;pper00520:Amino sugar and nucleotide sugar metabolism;pper00051:Fructose and mannose metabolism	GO:0009058: biosynthetic process	GO:0016779: nucleotidyltransferase activity	GO:0005777:peroxisome;GO:0005829:cytosol

ppe-miR399b	18776912	PRUPE_ppa003460mg	NCBI_Assembly:GCF_000346465.:	NW_006760212.1	+	9078875	9082464	3589	-	-	-	-
ppe-miR399b	18777061	PRUPE_ppa020890mg	NCBI_Assembly:GCF_000346465.:	NW_006760212.1	-	15425077	15426824	1747	-	-	-	-
ppe-miR399b	18777185	PRUPE_ppa008757mg	NCBI_Assembly:GCF_000346465.:	NW_006760212.1	-	11307803	11312183	4380	pper03015:mRNA surveillance pathway	-	GO:0004721: phosphoprotein phosphatase activity	-
ppe-miR399b	18777245	PRUPE_ppa019629mg	NCBI_Assembly:GCF_000346465.:	NW_006760212.1	+	399657	400754	1097	-	-	-	-
ppe-miR399b	18777270	PRUPE_ppa009214mg	NCBI_Assembly:GCF_000346465.:	NW_006760212.1	-	14891980	14893875	1895	-	GO:0009965:leaf morphogenesis;GO:0016556:mRNA modification;GO:0030154:cell differentiation	-	-
ppe-miR399b	18777469	PRUPE_ppa004958mg	NCBI_Assembly:GCF_000346465.:	NW_006760212.1	+	630596	634001	3405	-	-	GO:0003677: DNA binding;GO:0003682:chromatin binding	GO:0005634:nucleus
ppe-miR399b	18777528	PRUPE_ppa022165mg	NCBI_Assembly:GCF_000346465.:	NW_006760212.1	-	9894510	9896108	1598	-	-	-	-
ppe-miR399b	18777566	PRUPE_ppa010235mg	NCBI_Assembly:GCF_000346465.:	NW_006760212.1	-	18069230	18073792	4562	-	-	-	-
ppe-miR399b	18777630	PRUPE_ppa000750mg	NCBI_Assembly:GCF_000346465.:	NW_006760212.1	-	2415750	2426878	11128	pper03013:RNA transport	-	-	-
ppe-miR399b	18777654	PRUPE_ppa016860mg	NCBI_Assembly:GCF_000346465.:	NW_006760212.1	-	3006577	3009415	2838	-	GO:0006281: DNA repair	GO:0003677: DNA binding;GO:0004519:endonuclease activity	GO:0005622:intracellular

ppe-miR399b	18777655	PRUPE_ppa020898mg	NCBI_Assembly:GCF_000346465.:	NW_006760212.1	-	10952680	10957733	5053	-	GO:0007155: cell adhesion;GO:0010090:trichome morphogenesis;GO:0010098:suspensor development;GO:0010103: stomatal complex morphogenesis;GO:0010229:inflorescence development;GO:0045010: actin nucleation;GO:0048765:root hair cell differentiation;GO:0071555: cell wall organization	GO:0004672: protein kinase activity;GO:0005524:ATP binding	-
ppe-miR399b	18777677	PRUPE_ppa022955mg	NCBI_Assembly:GCF_000346465.:	NW_006760212.1	-	16403484	16405948	2464	-	-	GO:0046872: metal ion binding	-
ppe-miR399b	18777715	PRUPE_ppa007046mg	NCBI_Assembly:GCF_000346465.:	NW_006760212.1	+	10848903	10853296	4393	pper00970:Aminoacyl-tRNA biosynthesis	GO:0006418:tRNA aminoacylation for protein translation	GO:0004831:tyrosine-tRNA ligase activity;GO:0005524:ATP binding	-
ppe-miR399b	18777918	PRUPE_ppb023334mg	NCBI_Assembly:GCF_000346465.:	NW_006760212.1	+	1815081	1816997	1916	-	-	-	-
ppe-miR399b	18778176	PRUPE_ppa009430mg	NCBI_Assembly:GCF_000346465.:	NW_006760220.1	+	2306567	2308181	1614	-	-	-	-

ppe-miR399b	18778342	PRUPE_ppa004336mg	NCBI_Assembly:GCF_000346465.:	NW_006760220.1	-	5076836	5078837	2001	-	GO:0006810:transport;GO:0016126:sterol biosynthetic process;GO:0052541:plant-type cell wall cellulose metabolic process;GO:0052546:cell wall pectin metabolic process	-	GO:0005634:nucleus;GO:0005829:cytosol;GO:0005886:plasma membrane;GO:0016021:integral component of membrane
ppe-miR399b	18778360	PRUPE_ppa021959mg	NCBI_Assembly:GCF_000346465.:	NW_006760220.1	+	11119202	11120234	1032	-	-	-	-
ppe-miR399b	18778372	PRUPE_ppb018056mg	NCBI_Assembly:GCF_000346465.:	NW_006760220.1	-	23355092	23356970	1878	-	-	-	-
ppe-miR399b	18778443	PRUPE_ppa017415mg	NCBI_Assembly:GCF_000346465.:	NW_006760220.1	+	1128116	1129997	1881	-	-	GO:0004674: protein serine/threonine kinase activity;GO:0005524:ATP binding	-
ppe-miR399b	18778509	PRUPE_ppa006635mg	NCBI_Assembly:GCF_000346465.:	NW_006760220.1	+	29521995	29524635	2640	-	-	GO:0016491: oxidoreductase activity	-
ppe-miR399b	18778521	PRUPE_ppa005108mg	NCBI_Assembly:GCF_000346465.:	NW_006760220.1	-	4188105	4190878	2773	-	-	-	-
ppe-miR399b	18778704	PRUPE_ppa012099mg	NCBI_Assembly:GCF_000346465.:	NW_006760220.1	-	13415929	13421283	5354	-	-	GO:0008137: NADH dehydrogenase (ubiquinone) activity;GO:0009055:electron carrier activity	GO:0016020:membrane
ppe-miR399b	18778827	PRUPE_ppa008267mg	NCBI_Assembly:GCF_000346465.:	NW_006760220.1	-	2843853	2845181	1328	-	-	GO:0008234: cysteine-type peptidase activity	-
ppe-miR399b	18778854	PRUPE_ppa001748mg	NCBI_Assembly:GCF_000346465.:	NW_006760220.1	+	639096	641744	2648	-	-	-	GO:0005829:cytosol

ppe-miR399b	18778880	PRUPE_ppa012362mg	NCBI_Assembly:GCF_000346465.:	NW_006760220.1	+	25691145	25692421	1276	pper01100:Metabolic pathways;pper00195:Photosynthesis	GO:0006098:pentose-phosphate shunt;GO:0009773:photosynthetic electron transport in photosystem I;GO:0019344:cysteine biosynthetic process;GO:0035304:regulation of protein dephosphorylation	-	GO:0009522:photosystem I;GO:0009535:chloroplast thylakoid membrane
ppe-miR399b	18778903	PRUPE_ppa022240mg	NCBI_Assembly:GCF_000346465.:	NW_006760220.1	-	20008775	20011714	2939	-	-	-	-
ppe-miR399b	18779215	PRUPE_ppa024717mg	NCBI_Assembly:GCF_000346465.:	NW_006760220.1	-	19357139	19362118	4979	pper01100:Metabolic pathways;pper00760:Nicotinate and nicotinamide metabolism	GO:0006741:NADP biosynthetic process;GO:0019674:NAD metabolic process	GO:0003951:NAD+ kinase activity;GO:0042736:NADH kinase activity	-
ppe-miR399b	18779223	PRUPE_ppa017095mg	NCBI_Assembly:GCF_000346465.:	NW_006760220.1	+	13899708	13900418	710	-	-	-	-
ppe-miR399b	18779406	PRUPE_ppa019372mg	NCBI_Assembly:GCF_000346465.:	NW_006760220.1	-	7326238	7326771	533	pper04120:Ubiquitin mediated proteolysis;pper04141:Protein processing in endoplasmic reticulum	GO:0006511:ubiquitin-dependent protein catabolic process	-	-
ppe-miR399b	18779450	PRUPE_ppa018537mg	NCBI_Assembly:GCF_000346465.:	NW_006760220.1	+	7449667	7452431	2764	-	-	GO:0004672:protein kinase activity;GO:0005524:ATP binding	-

ppe-miR399b	18779452	PRUPE_ppa024643mg	NCBI_Assembly:GCF_000346465.:	NW_006760220.1	-	5112924	5114337	1413	-	-	GO:0022857:transmembrane transporter activity	GO:0016021:integral component of membrane
ppe-miR399b	18779603	PRUPE_ppa002355mg	NCBI_Assembly:GCF_000346465.:	NW_006760220.1	+	15716296	15721927	5631	-	GO:0000956:nuclear-transcribed mRNA catabolic process;GO:0009294:DNA mediated transformation;GO:0045931:positive regulation of mitotic cell cycle;GO:0048364:root development	-	-
ppe-miR399b	18779605	PRUPE_ppa012912mg	NCBI_Assembly:GCF_000346465.:	NW_006760220.1	-	25011636	25014071	2435	pper04626:Plant-pathogen interaction;pper04070:Phosphatidylinositol signaling system	-	GO:0005509:calcium ion binding	-

ppe-miR399b	18779621	PRUPE_ppa000929mg	NCBI_Assembly:GCF_000346465.:	NW_006760220.1	-	26278327	26293608	15281	pper01100:Metabolic pathways;pper00230:Purine metabolism;pper00240:Pyrimidine metabolism;pper03030:DNA replication;pper03420:Nucleotide excision repair;pper03430:Mismatch repair;pper03440:Homologous recombination;pper03410:Base excision repair	GO:0006260:DNA replication	GO:0000166:nucleotide binding;GO:0003677:DNA binding;GO:0003887:DNA-directed DNA polymerase activity;GO:0008408:3'-5' exonuclease activity	-
ppe-miR399b	18779797	PRUPE_ppa021626mg	NCBI_Assembly:GCF_000346465.:	NW_006760220.1	+	15362309	15363340	1031	-	-	-	GO:0042170:plastid membrane
ppe-miR399b	18779857	PRUPE_ppa015582mg	NCBI_Assembly:GCF_000346465.:	NW_006760220.1	-	25370914	25372098	1184	-	-	-	-
ppe-miR399b	18779895	PRUPE_ppa022280mg	NCBI_Assembly:GCF_000346465.:	NW_006760220.1	+	9537167	9538049	882	-	GO:0006351:transcription, DNA-templated	GO:0043565:sequence-specific DNA binding	-
ppe-miR399b	18779967	PRUPE_ppa002309mg	NCBI_Assembly:GCF_000346465.:	NW_006760220.1	+	1344438	1347113	2675	-	-	GO:0004674:protein serine/threonine kinase activity;GO:0005524:ATP binding	-
ppe-miR399b	18780108	PRUPE_ppa016234mg	NCBI_Assembly:GCF_000346465.:	NW_006760220.1	-	5105620	5106582	962	-	-	GO:0022857:transmembrane transporter activity	GO:0016021:integral component of membrane

ppe-miR399b	18783081	PRUPE_ppa006640mg	NCBI_Assembly:GCF_000346465.:	NW_006760268.1	+	6029077	6030548	1471	pper00400:Phenylalanine, tyrosine and tryptophan biosynthesis;pper01100:Metabolic pathways;pper01230:Biosynthesis of amino acids	GO:0006567:threonine catabolic process;GO:0006571:tyrosine biosynthetic process	GO:0004665:prephenate dehydrogenase (NADP+) activity;GO:0008977:prephenate dehydrogenase activity	-

ppe-miR399b	18783250	PRUPE_ppa001405mg	NCBI_Assembly:GCF_000346465.:	NW_006760268.1	-	3503422	3509619	6197	-	GO:0006995:cellular response to nitrogen starvation;GO:0007155:cell adhesion;GO:0009944:polarity specification of adaxial/abaxial axis;GO:0009965:leaf morphogenesis;GO:0010014:meristem initiation;GO:0010051:xylem and phloem pattern formation;GO:0010075:regulation of meristem growth;GO:0010089:xylem development;GO:0010090:trichome morphogenesis;GO:0045010:actin nucleation;GO:0048263:determination of dorsal identity;GO:00	GO:0003700:sequence-specific DNA binding transcription factor activity;GO:0008289:lipid binding;GO:0043565:sequence-specific DNA binding	GO:0005634:nucleus

ppe-miR399b	18783348	PRUPE_ppa008778mg	NCBI_Assembly:GCF_000346465.:	NW_006760268.1	+	7620937	7625768	4831	pper01100:Metabolic pathways;pper01110:Biosynthesis of secondary metabolites;pper00010:Glycolysis / Gluconeogenesis	GO:0005975: carbohydrate metabolic process	GO:0016853:isomerase activity;GO:0030246:carbohydrate binding	-
ppe-miR399b	18783512	PRUPE_ppa012953mg	NCBI_Assembly:GCF_000346465.:	NW_006760268.1	-	5157084	5158957	1873	-	GO:0006412:translation	GO:0003735: structural constituent of ribosome;GO:0019843:rRNA binding	GO:0005840:ribosome;GO:0009507:chloroplast
ppe-miR399b	18783570	PRUPE_ppa008451mg	NCBI_Assembly:GCF_000346465.:	NW_006760268.1	+	10781748	10787797	6049	-	-	GO:0008168: methyltransferase activity	GO:0005634:nucleus;GO:0005829:cytosol
ppe-miR399b	18783605	PRUPE_ppa016199mg	NCBI_Assembly:GCF_000346465.:	NW_006760268.1	+	2380072	2380909	837	-	-	-	GO:0016021:integral component of membrane
ppe-miR399b	18783654	PRUPE_ppa006437mg	NCBI_Assembly:GCF_000346465.:	NW_006760268.1	+	19246165	19248673	2508	pper00562:Inositol phosphate metabolism;pper04070:Phosphatidylinositol signaling system	GO:0009555: pollen development;GO:0009860: pollen tube growth;GO:0030048:actin filament-based movement	GO:0004725: protein tyrosine phosphatase activity;GO:0008138:protein tyrosine/serine/threonine phosphatase activity	-
ppe-miR399b	18783705	PRUPE_ppa017372mg	NCBI_Assembly:GCF_000346465.:	NW_006760268.1	-	20707687	20709492	1805	-	-	-	-

ppe-miR399b	18783794	PRUPE_ppa001344mg	NCBI_Assembly:GCF_000346465.:	NW_006760268.1	-	14714849	14718257	3408	-	GO:0000956: nuclear-transcribed mRNA catabolic process;GO:0006487:protein N-linked glycosylation; GO:0007623: circadian rhythm;GO:0008284:positive regulation of cell proliferation;GO:0009630:gravitropism;GO:0010218:response to far red light;GO:0042753:positive regulation of circadian rhythm;GO:0045893:positive regulation of transcription, DNA-templated	GO:0003700: sequence-specific DNA binding transcription factor activity;GO:0008270:zinc ion binding	-
ppe-miR399b	18783854	PRUPE_ppa007817mg	NCBI_Assembly:GCF_000346465.:	NW_006760268.1	+	19047717	19051663	3946	-	-	GO:0003824: catalytic activity	-
ppe-miR399b	18784159	PRUPE_ppa020823mg	NCBI_Assembly:GCF_000346465.:	NW_006760268.1	-	14649691	14649912	221	-	-	-	-

ppe-miR399b	18784227	PRUPE_ppa012238mg	NCBI_Assembly:GCF_000346465.:	NW_006760268.1	+	1736220	1738450	2230	-	-	GO:0009055: electron carrier activity;GO:0046872:metal ion binding;GO:0051537:2 iron, 2 sulfur cluster binding	-
ppe-miR399b	18784331	PRUPE_ppa004522mg	NCBI_Assembly:GCF_000346465.:	NW_006760268.1	+	19637658	19639175	1517	-	-	-	-
ppe-miR399b	18784361	PRUPE_ppa002721mg	NCBI_Assembly:GCF_000346465.:	NW_006760268.1	-	18498827	18500772	1945	-	-	GO:0016773: phosphotransferase activity, alcohol group as acceptor	-
ppe-miR399b	18784392	PRUPE_ppa014443mg	NCBI_Assembly:GCF_000346465.:	NW_006760268.1	-	21468808	21469557	749	pper04141:Protein processing in endoplasmic reticulum;pper03060:Protein export;pper04145:Phagosome	GO:0006605: protein targeting;GO:0015824:proline transport	GO:0015450: P-P-bond-hydrolysis-driven protein transmembrane transporter activity	GO:0005622:intracellular;GO:0005886:plasma membrane
ppe-miR399b	18784433	PRUPE_ppa016534mg	NCBI_Assembly:GCF_000346465.:	NW_006760268.1	-	4384986	4386220	1234	-	-	-	-
ppe-miR399b	18784567	PRUPE_ppa000036mg	NCBI_Assembly:GCF_000346465.:	NW_006760324.1	-	18180356	18193956	13600	-	-	GO:0001104: RNA polymerase II transcription cofactor activity	GO:0016592:mediator complex

ppe-miR399b	18784732	PRUPE_ppa019379mg	NCBI_Assembly:GCF_000346465.:	NW_006760324.1	-	21142029	21143722	1693	-	GO:0000398: mRNA splicing, via spliceosome; GO:0009560: embryo sac egg cell differentiation; GO:0010030: positive regulation of seed germination;GO:0045893:positive regulation of transcription, DNA-templated	-	-
ppe-miR399b	18785018	PRUPE_ppa006442mg	NCBI_Assembly:GCF_000346465.:	NW_006760324.1	+	23859618	23862800	3182	pper01100:Metabolic pathways;pper00330:Arginine and proline metabolism;pper00910:Nitrogen metabolism;pper00250:Alanine, aspartate and glutamate metabolism	GO:0006598: polyamine catabolic process;GO:0009698:phenylpropanoid metabolic process;GO:0042398:cellular modified amino acid biosynthetic process;GO:0046482:para-aminobenzoic acid metabolic process;GO:0048193:Golgi vesicle transport	GO:0004352: glutamate dehydrogenase (NAD+) activity;GO:0005507:copper ion binding;GO:0005524:ATP binding;GO:0008270:zinc ion binding;GO:0050897:cobalt ion binding	GO:0005774:vacuolar membrane

ppe-miR399b	18785192	PRUPE_ppa009021mg	NCBI_Assembly:GCF_000346465.:	NW_006760324.1	+	17133123	17135222	2099	pper04145:Phagosome;pper04130:SNARE interactions in vesicular transport	GO:0000226: microtubule cytoskeleton organization; GO:0016192: vesicle-mediated transport	-	GO:0016020:membrane
ppe-miR399b	18785324	PRUPE_ppa001731mg	NCBI_Assembly:GCF_000346465.:	NW_006760324.1	-	24439869	24442274	2405	-	-	GO:0003676: nucleic acid binding	-
ppe-miR399b	18785337	PRUPE_ppa009506mg	NCBI_Assembly:GCF_000346465.:	NW_006760324.1	-	22777485	22779328	1843	-	-	GO:0005215:transporter activity	GO:0016021:integral component of membrane
ppe-miR399b	18785371	PRUPE_ppa018509mg	NCBI_Assembly:GCF_000346465.:	NW_006760324.1	-	9879290	9879556	266	-	-	-	-
ppe-miR399b	18785386	PRUPE_ppb019262mg	NCBI_Assembly:GCF_000346465.:	NW_006760324.1	-	17378949	17379698	749	-	-	-	-
ppe-miR399b	18785682	PRUPE_ppa011130mg	NCBI_Assembly:GCF_000346465.:	NW_006760324.1	-	13708533	13709230	697	pper03010:Ribosome	GO:0006364:rRNA processing;GO:0006412:translation;GO:0010207:photosystem II assembly;GO:0015995:chlorophyll biosynthetic process;GO:0019288:isopentenyl diphosphate biosynthetic process, methylerythritol 4-phosphate pathway;GO:0045036:protein targeting to chloroplast	GO:0003735: structural constituent of ribosome	GO:0005840:ribosome;GO:0009570:chloroplast stroma;GO:0009941:chloroplast envelope;GO:0016020:membrane

ppe-miR399b	18786011	PRUPE_ppa021814mg	NCBI_Assembly:GCF_000346465.:	NW_006760324.1	+	13697799	13702325	4526	pper00360:Phenylalanine metabolism;pper01100:Metabolic pathways;pper01110:Biosynthesis of secondary metabolites;pper00940:Phenylpropanoid biosynthesis;pper00941:Flavonoid biosynthesis;pper00945:Stilbenoid, diarylheptanoid and gingerol biosynthesis	-	GO:0008171: O-methyltransferase activity	-
ppe-miR399b	18786333	PRUPE_ppa003681mg	NCBI_Assembly:GCF_000346465.:	NW_006760324.1	+	25477115	25481676	4561	-	-	-	-
ppe-miR399b	18786407	PRUPE_ppa019822mg	NCBI_Assembly:GCF_000346465.:	NW_006760324.1	+	12490051	12491311	1260	-	-	-	-
ppe-miR399b	18786411	PRUPE_ppa000445mg	NCBI_Assembly:GCF_000346465.:	NW_006760324.1	-	24190832	24197514	6682	-	GO:0007076: mitotic chromosome condensation	GO:0005524: ATP binding	GO:0000796:condensin complex;GO:0005634:nucleus
ppe-miR399b	18786418	PRUPE_ppa011423mg	NCBI_Assembly:GCF_000346465.:	NW_006760324.1	+	26200835	26202162	1327	-	-	-	-
ppe-miR399b	18786458	PRUPE_ppa014237mg	NCBI_Assembly:GCF_000346465.:	NW_006760324.1	-	2237936	2238383	447	-	-	-	-
ppe-miR399b	18786578	PRUPE_ppa018411mg	NCBI_Assembly:GCF_000346465.:	NW_006760324.1	-	14596048	14597509	1461	-	-	-	-
ppe-miR399b	18786898	PRUPE_ppa021725mg	NCBI_Assembly:GCF_000346465.:	NW_006760324.1	+	4120981	4123664	2683	-	-	-	-
ppe-miR399b	18786965	PRUPE_ppa020941mg	NCBI_Assembly:GCF_000346465.:	NW_006760324.1	-	15534547	15535258	711	-	-	GO:0003676: nucleic acid binding	-
ppe-miR399b	18787038	PRUPE_ppa002072mg	NCBI_Assembly:GCF_000346465.:	NW_006760324.1	-	25978206	25981397	3191	-	-	-	-
ppe-miR399b	18787308	PRUPE_ppa020296mg	NCBI_Assembly:GCF_000346465.:	NW_006760324.1	-	15759794	15761546	1752	-	-	-	-

ppe-miR399b	18787358	PRUPE_ppa009068mg	NCBI_Assembly:GCF_000346465.:	NW_006760324.1	-	12082619	12090092	7473	-	GO:0008380: RNA splicing;GO:0046719:regulation by virus of viral protein levels in host cell	GO:0003723: RNA binding;GO:0003727:single-stranded RNA binding	-
ppe-miR399b	18787608	PRUPE_ppa009450mg	NCBI_Assembly:GCF_000346465.:	NW_006760324.1	-	24078449	24082397	3948	-	GO:0006623: protein targeting to vacuole;GO:0007165:signal transduction; GO:0048193: Golgi vesicle transport	-	GO:0005622:intracellular
ppe-miR399b	18787754	PRUPE_ppa021732mg	NCBI_Assembly:GCF_000346465.:	NW_006760340.1	+	6	4235	4229	-	-	GO:0043531: ADP binding	-
ppe-miR399b	18787759	PRUPE_ppb022397m2g	NCBI_Assembly:GCF_000346465.:	NW_006760343.1	-	340306	341692	1386	-	-	-	-
ppe-miR399b	18787868	PRUPE_ppb014711mg	NCBI_Assembly:GCF_000346465.:	NW_006760367.1	+	463949	467551	3602	-	-	-	-
ppe-miR399b	18787900	PRUPE_ppa018589mg	NCBI_Assembly:GCF_000346465.:	NW_006760373.1	+	5684	6014	330	-	GO:0009611:response to wounding	GO:0004867: serine-type endopeptidase inhibitor activity	-
ppe-miR399b	18788068	PRUPE_ppa002104mg	NCBI_Assembly:GCF_000346465.:	NW_006760384.1	+	333822	338385	4563	-	-	GO:0005452:inorganic anion exchanger activity	GO:0016021:integral component of membrane
ppe-miR399b	18788080	PRUPE_ppa005383mg	NCBI_Assembly:GCF_000346465.:	NW_006760384.1	-	286377	289068	2691	-	GO:0008033:tRNA processing	GO:0008168: methyltransferase activity	-
ppe-miR399b	18788117	PRUPE_ppa003906mg	NCBI_Assembly:GCF_000346465.:	NW_006760385.1	+	36733550	36737291	3741	-	GO:0000956: nuclear-transcribed mRNA catabolic process	GO:0004176: ATP-dependent peptidase activity	-

ppe-miR399b	18788159	PRUPE_ppa022414mg	NCBI_Assembly:GCF_000346465.:	NW_006760385.1	-	33690398	33690652	254	-	-	GO:0005506:iron ion binding;GO:0016705:oxidoreductase activity, acting on paired donors, with incorporation or reduction of molecular oxygen;GO:0020037:heme binding	-
ppe-miR399b	18788401	PRUPE_ppa004685mg	NCBI_Assembly:GCF_000346465.:	NW_006760385.1	-	10036791	10039515	2724	pper01100:Metabolic pathways;pper01110:Biosynthesis of secondary metabolites;pper00280:Valine, leucine and isoleucine degradation	GO:0009750:response to fructose	GO:0004147:dihydrolipoamide branched chain acyltransferase activity;GO:0008270:zinc ion binding;GO:0016407:acetyltransferase activity	-
ppe-miR399b	18788464	PRUPE_ppa024080mg	NCBI_Assembly:GCF_000346465.:	NW_006760385.1	-	27083790	27084839	1049	-	-	GO:0003677:DNA binding	-
ppe-miR399b	18788581	PRUPE_ppa010943mg	NCBI_Assembly:GCF_000346465.:	NW_006760385.1	+	42726341	42727038	697	-	-	-	-
ppe-miR399b	18788599	PRUPE_ppa024089mg	NCBI_Assembly:GCF_000346465.:	NW_006760385.1	-	16436803	16438448	1645	-	-	GO:0005351:sugar:proton symporter activity	GO:0000139:Golgi membrane;GO:0016021:integral component of membrane

ppe-miR399b	18788649	PRUPE_ppa002482mg	NCBI_Assembly:GCF_000346465.:	NW_006760385.1	+	24622186	24625295	3109	-	GO:0006486: protein glycosylation;GO:0006816: calcium ion transport;GO:0009630:gravitropism	-	-
ppe-miR399b	18788740	PRUPE_ppa002909mg	NCBI_Assembly:GCF_000346465.:	NW_006760385.1	+	1904466	1913208	8742	pper04141:Protein processing in endoplasmic reticulum	-	GO:0004571: mannosyl-oligosaccharide 1,2-alpha-mannosidase activity;GO:0005509:calcium ion binding	GO:0016020:membrane
ppe-miR399b	18788904	PRUPE_ppa008145mg	NCBI_Assembly:GCF_000346465.:	NW_006760385.1	-	13844264	13847050	2786	-	-	-	-
ppe-miR399b	18788977	PRUPE_ppa007907mg	NCBI_Assembly:GCF_000346465.:	NW_006760385.1	-	29062609	29064394	1785	-	GO:0019288:isopentenyl diphosphate biosynthetic process, methylerythritol 4-phosphate pathway	-	-
ppe-miR399b	18789053	PRUPE_ppa003530mg	NCBI_Assembly:GCF_000346465.:	NW_006760385.1	-	25359301	25361906	2605	-	GO:0045454: cell redox homeostasis	GO:0047134: protein-disulfide reductase activity	GO:0005623:cell
ppe-miR399b	18789148	PRUPE_ppa012638mg	NCBI_Assembly:GCF_000346465.:	NW_006760385.1	+	36004896	36006401	1505	pper04120:Ubiquitin mediated proteolysis;pper03013:RNA transport	-	GO:0016874:ligase activity;GO:0019789:SUMO transferase activity	-
ppe-miR399b	18789432	PRUPE_ppb022567mg	NCBI_Assembly:GCF_000346465.:	NW_006760385.1	-	37583763	37584447	684	pper03015:mRNA surveillance pathway	-	GO:0005525: GTP binding	-
ppe-miR399b	18789566	PRUPE_ppb008955mg	NCBI_Assembly:GCF_000346465.:	NW_006760385.1	+	34438515	34439741	1226	-	-	-	-

ppe-miR399b	18789946	PRUPE_ppa020172mg	NCBI_Assembly:GCF_000346465.:	NW_006760385.1	+	43042853	43049167	6314	-	GO:0006952: defense response;GO: 0009607:resp onse to biotic stimulus	-	GO:0016021:in tegral component of membrane
ppe-miR399b	18790012	PRUPE_ppa002673mg	NCBI_Assembly:GCF_000346465.:	NW_006760385.1	+	39173782	39177492	3710	pper04075:Pl ant hormone signal transduction	-	-	-
ppe-miR399b	18790697	PRUPE_ppa023790mg	NCBI_Assembly:GCF_000346465.:	NW_006760385.1	+	20853155	20855656	2501	-	-	-	-
ppe-miR399b	18790754	PRUPE_ppa001790mg	NCBI_Assembly:GCF_000346465.:	NW_006760385.1	+	31819140	31822522	3382	-	GO:0009901: anther dehiscence;G O:0010584:po llen exine formation	GO:0016787: hydrolase activity	-
ppe-miR399b	18790913	PRUPE_ppa003015mg	NCBI_Assembly:GCF_000346465.:	NW_006760385.1	+	13754849	13761403	6554	-	-	-	-
ppe-miR399b	18790926	PRUPE_ppa010254mg	NCBI_Assembly:GCF_000346465.:	NW_006760385.1	-	17569154	17573314	4160	-	-	-	-
ppe-miR399b	18790955	PRUPE_ppa013995mg	NCBI_Assembly:GCF_000346465.:	NW_006760385.1	-	41860866	41861701	835	-	-	-	-
ppe-miR399b	18790961	PRUPE_ppa022592mg	NCBI_Assembly:GCF_000346465.:	NW_006760385.1	-	25127315	25127977	662	-	-	-	-
ppe-miR399b	18791047	PRUPE_ppa004329mg	NCBI_Assembly:GCF_000346465.:	NW_006760385.1	-	28294906	28296885	1979	pper00905:Br assinosteroid biosynthesis	-	GO:0004497: monooxygena se activity;GO:00 05506:iron ion binding;GO:00 16705:oxidore ductase activity, acting on paired donors, with incorporation or reduction of molecular oxygen;GO:00 20037:heme binding	-
ppe-miR399b	18791054	PRUPE_ppa014041mg	NCBI_Assembly:GCF_000346465.:	NW_006760385.1	+	21880360	21882024	1664	-	-	-	-
ppe-miR399b	18791097	PRUPE_ppa003140mg	NCBI_Assembly:GCF_000346465.:	NW_006760385.1	-	3339719	3342360	2641	-	-	-	-

ppe-miR399b	18791099	PRUPE_ppa027076mg	NCBI_Assembly:GCF_000346465.:	NW_006760385.1	+	34223590	34225449	1859	-	-	-	-
ppe-miR399b	18791100	PRUPE_ppa000724mg	NCBI_Assembly:GCF_000346465.:	NW_006760385.1	-	36446655	36453195	6540	-	-	GO:0016787: hydrolase activity	-
ppe-miR399b	18791106	PRUPE_ppa015567mg	NCBI_Assembly:GCF_000346465.:	NW_006760385.1	-	44704111	44705703	1592	pper03010:Ribosome	GO:0006412:translation	GO:0003735: structural constituent of ribosome	GO:0005840:ribosome
ppe-miR399b	18791112	PRUPE_ppa003189mg	NCBI_Assembly:GCF_000346465.:	NW_006760385.1	-	44118136	44120327	2191	-	-	-	-
ppe-miR399b	18791139	PRUPE_ppa018147mg	NCBI_Assembly:GCF_000346465.:	NW_006760385.1	+	19832252	19834227	1975	-	-	-	-
ppe-miR399b	18791732	PRUPE_ppa004372mg	NCBI_Assembly:GCF_000346465.:	NW_006760385.1	+	33752297	33755478	3181	pper01100:Metabolic pathways;pper01110:Biosynthesis of secondary metabolites;pper00904:Diterpenoid biosynthesis	-	GO:0004497: monooxygenase activity;GO:0005506:iron ion binding;GO:0016705:oxidoreductase activity, acting on paired donors, with incorporation or reduction of molecular oxygen;GO:0020037:heme binding	-
ppe-miR399b	18791750	PRUPE_ppa016970mg	NCBI_Assembly:GCF_000346465.:	NW_006760385.1	+	21325279	21326227	948	-	-	-	-
ppe-miR399b	18791934	PRUPE_ppa000005m1g	NCBI_Assembly:GCF_000346465.:	NW_006760385.1	+	8362457	8373750	11293	-	-	-	-
ppe-miR399b	18792036	PRUPE_ppa024963mg	NCBI_Assembly:GCF_000346465.:	NW_006760385.1	-	44312622	44317310	4688	-	GO:0007165: signal transduction	GO:0043531: ADP binding	-
ppe-miR399b	18792194	PRUPE_ppa014339mg	NCBI_Assembly:GCF_000346465.:	NW_006760385.1	+	5172209	5172794	585	-	GO:0030150: protein import into mitochondrial matrix	-	GO:0005742:mitochondrial outer membrane translocase complex

ppe-miR399b	18792764	PRUPE_ppa009133mg	NCBI_Assembly:GCF_000346465.:	NW_006760385.1	-	41927883	41931905	4022	-	GO:0000398: mRNA splicing, via spliceosome	GO:0000166: nucleotide binding;GO:0003676:nucleic acid binding	GO:0009570:chloroplast stroma
ppe-miR399b	18792903	PRUPE_ppa009396mg	NCBI_Assembly:GCF_000346465.:	NW_006760385.1	-	31619016	31622752	3736	-	-	GO:0003677: DNA binding;GO:0003682:chromatin binding;GO:0003700:sequence-specific DNA binding transcription factor activity	-
ppe-miR399b	18793012	PRUPE_ppa019562mg	NCBI_Assembly:GCF_000346465.:	NW_006760385.1	-	3938447	3938773	326	-	-	-	-
ppe-miR399b	18793142	PRUPE_ppb020678mg	NCBI_Assembly:GCF_000346465.:	NW_006760385.1	+	18820246	18820776	530	-	-	GO:0003676: nucleic acid binding;GO:0008270:zinc ion binding	-
ppe-miR399b	18793151	PRUPE_ppa020400mg	NCBI_Assembly:GCF_000346465.:	NW_006760385.1	+	8590084	8590878	794	-	-	GO:0004857: enzyme inhibitor activity;GO:0030599:pectinesterase activity	-
ppe-miR399b	18793265	PRUPE_ppa017041mg	NCBI_Assembly:GCF_000346465.:	NW_006760385.1	-	43950408	43955109	4701	-	GO:0007165: signal transduction	GO:0043531: ADP binding	-

ppe-miR399b	18793350	PRUPE_ppa009961mg	NCBI_Assembly:GCF_000346465.:	NW_006760385.1	+	37118692	37121814	3122	-	GO:0000917:barrier septum assembly;GO:0006364:rRNA processing;GO:0006399:tRNA metabolic process;GO:0009658:chloroplast organization	GO:0005525:GTP binding	-
ppe-miR399b	18793354	PRUPE_ppa005309mg	NCBI_Assembly:GCF_000346465.:	NW_006760385.1	-	3272193	3275209	3016	pper03015:mRNA surveillance pathway	-	GO:0000166:nucleotide binding;GO:0003676:nucleic acid binding	-
ppe-miR399b	18793564	PRUPE_ppa004735mg	NCBI_Assembly:GCF_000346465.:	NW_006760385.1	+	4984096	4989039	4943	-	-	-	-
ppe-miR399b	18793567	PRUPE_ppa017967mg	NCBI_Assembly:GCF_000346465.:	NW_006760385.1	-	41735661	41736497	836	-	-	-	-
ppe-miR399b	18793609	PRUPE_ppa016563mg	NCBI_Assembly:GCF_000346465.:	NW_006760385.1	-	36508257	36511249	2992	-	-	GO:0003676:nucleic acid binding	-
ppe-miR399b	18793833	PRUPE_ppa002209mg	NCBI_Assembly:GCF_000346465.:	NW_006760385.1	+	35292333	35295328	2995	-	-	-	GO:0009506:plasmodesma
ppe-miR399b	18793921	PRUPE_ppa025225mg	NCBI_Assembly:GCF_000346465.:	NW_006760385.1	+	32563831	32565785	1954	-	-	GO:0033926:glycopeptide alpha-N-acetylgalactosaminidase activity	-
ppe-miR399b	18793974	PRUPE_ppa008147mg	NCBI_Assembly:GCF_000346465.:	NW_006760385.1	-	30835909	30838099	2190	pper00740:Riboflavin metabolism	-	GO:0003993:acid phosphatase activity	-
ppe-miR7122b-5p	18766201	PRUPE_ppa020801mg	NCBI_Assembly:GCF_000346465.:	NW_006760194.1	-	6395785	6397333	1548	pper00970:Aminoacyl-tRNA biosynthesis	GO:0006422:aspartyl-tRNA aminoacylation	GO:0004815:aspartate-tRNA ligase activity;GO:0005524:ATP binding	GO:0005737:cytoplasm
ppe-miR7122b-5p	18769878	PRUPE_ppa023151mg	NCBI_Assembly:GCF_000346465.:	NW_006760201.1	+	19662152	19662805	653	-	-	-	-

ppe-miR7122b-5p	18769935	PRUPE_ppa013735mg	NCBI_Assembly:GCF_000346465.:	NW_006760201.1	-	17453450	17454015	565	pper04075:Plant hormone signal transduction	-	-	-
ppe-miR7122b-5p	18771686	PRUPE_ppa008196mg	NCBI_Assembly:GCF_000346465.:	NW_006760201.1	-	715756	719007	3251	-	-	-	-
ppe-miR7122b-5p	18772575	PRUPE_ppa004875mg	NCBI_Assembly:GCF_000346465.:	NW_006760208.1	+	28753884	28755732	1848	-	-	GO:0016747:transferase activity, transferring acyl groups other than amino-acyl groups	-
ppe-miR7122b-5p	18772997	PRUPE_ppa006964mg	NCBI_Assembly:GCF_000346465.:	NW_006760208.1	-	5747257	5750038	2781	pper01100:Metabolic pathways;pper00740:Riboflavin metabolism	GO:0009231:riboflavin biosynthetic process	GO:0003935: GTP cyclohydrolase II activity;GO:0008686:3,4-dihydroxy-2-butanone-4-phosphate synthase activity	-
ppe-miR7122b-5p	18773203	PRUPE_ppa018471mg	NCBI_Assembly:GCF_000346465.:	NW_006760208.1	-	27922175	27925154	2979	-	-	-	-

ppe-miR7122b-5p	18774044	PRUPE_ppa012763mg	NCBI_Assembly:GCF_000346465.:	NW_006760208.1	-	20717570	20720396	2826	pper01100:Metabolic pathways;pper00190:Oxidative phosphorylation	GO:0006096: glycolytic process;GO:0006511:ubiquitin-dependent protein catabolic process;GO:0006833:water transport;GO:0006972:hyperosmotic response;GO:0007030:Golgi organization; GO:0009631: cold acclimation;GO:0009651:response to salt stress;GO:0009853:photorespiration;GO:0022900:electron transport chain;GO:0046686:response to cadmium ion;GO:0051788:response to misfolded protein;GO:0080129:proteasome core complex assembly	GO:0008137: NADH dehydrogenase (ubiquinone) activity;GO:0050897:cobalt ion binding	GO:0005747:mitochondrial respiratory chain complex I
ppe-miR7122b-5p	18774122	PRUPE_ppa011070mg	NCBI_Assembly:GCF_000346465.:	NW_006760208.1	+	18008979	18011047	2068	pper04144:Endocytosis	GO:0007264: small GTPase mediated signal transduction; GO:0015031: protein transport	GO:0005525: GTP binding	GO:0005622:intracellular

ppe-miR7122b-5p	18774255	PRUPE_ppa003080mg	NCBI_Assembly:GCF_000346465.:	NW_006760208.1	-	18129628	18135355	5727	-	GO:0006351:transcription, DNA-templated	GO:0003714:transcription corepressor activity	-
ppe-miR7122b-5p	18774508	PRUPE_ppa018361mg	NCBI_Assembly:GCF_000346465.:	NW_006760208.1	+	28435994	28437650	1656	-	-	GO:0004672: protein kinase activity;GO:0005524:ATP binding	-
ppe-miR7122b-5p	18775543	PRUPE_ppb023165mg	NCBI_Assembly:GCF_000346465.:	NW_006760208.1	-	25000262	25002805	2543	-	-	GO:0033926: glycopeptide alpha-N-acetylgalactosaminidase activity	-
ppe-miR7122b-5p	18776725	PRUPE_ppa023983mg	NCBI_Assembly:GCF_000346465.:	NW_006760212.1	+	11151284	11153563	2279	-	-	-	-
ppe-miR7122b-5p	18776973	PRUPE_ppa019102m2g	NCBI_Assembly:GCF_000346465.:	NW_006760212.1	-	12010103	12010569	466	-	-	GO:0008270: zinc ion binding	-
ppe-miR7122b-5p	18778167	PRUPE_ppa007013mg	NCBI_Assembly:GCF_000346465.:	NW_006760220.1	-	15942619	15945529	2910	-	-	-	-

ppe-miR7122b-5p	18778250	PRUPE_ppa010162mg	NCBI_Assembly:GCF_000346465.:	NW_006760220.1	+	2902954	2906436	3482	-	GO:0006096: glycolytic process;GO:0006833:water transport;GO:0006972:hyperosmotic response;GO:0007030:Golgi organization; GO:0009266:response to temperature stimulus;GO:0009651:response to salt stress;GO:0009750:response to fructose;GO:0019288:isopentenyl diphosphate biosynthetic process, methylerythritol 4-phosphate pathway;GO:0019344:cysteine biosynthetic process;GO:0032880:regulation of protein localization;GO:0042744:hydrogen	GO:0005509: calcium ion binding	GO:0005634:nucleus;GO:0005829:cytosol;GO:0005886:plasma membrane
ppe-miR7122b-5p	18778366	PRUPE_ppa021398mg	NCBI_Assembly:GCF_000346465.:	NW_006760220.1	-	4514473	4514937	464	-	-	-	-
ppe-miR7122b-5p	18778647	PRUPE_ppa011437mg	NCBI_Assembly:GCF_000346465.:	NW_006760220.1	+	3729018	3729873	855	-	-	-	-

ppe-miR7122b-5p	18778695	PRUPE_ppa004903mg	NCBI_Assembly:GCF_000346465.:	NW_006760220.1	-	6203915	6210952	7037	pper01100:Metabolic pathways;pper01110:Biosynthesis of secondary metabolites;pper00520:Amino sugar and nucleotide sugar metabolism	-	GO:0070569: uridylyltransferase activity	-
ppe-miR7122b-5p	18783018	PRUPE_ppa026640mg	NCBI_Assembly:GCF_000346465.:	NW_006760268.1	-	12877574	12879431	1857	-	-	GO:0003677: DNA binding;GO:0003682:chromatin binding	-
ppe-miR7122b-5p	18783121	PRUPE_ppa025169mg	NCBI_Assembly:GCF_000346465.:	NW_006760268.1	-	5676848	5679135	2287	-	-	GO:0043531: ADP binding	-
ppe-miR7122b-5p	18783408	PRUPE_ppa010088mg	NCBI_Assembly:GCF_000346465.:	NW_006760268.1	+	20860426	20862309	1883	-	-	GO:0008168: methyltransferase activity	-
ppe-miR7122b-5p	18783489	PRUPE_ppa000531mg	NCBI_Assembly:GCF_000346465.:	NW_006760268.1	+	12470972	12489060	18088	-	GO:0006486: protein glycosylation; GO:0010205: photoinhibition	GO:0004252: serine-type endopeptidase activity	GO:0005829:cytosol;GO:0009507:chloroplast
ppe-miR7122b-5p	18783723	PRUPE_ppa012986mg	NCBI_Assembly:GCF_000346465.:	NW_006760268.1	+	20200036	20202138	2102	-	GO:0000023: maltose metabolic process;GO:0019252:starch biosynthetic process;GO:0043085:positive regulation of catalytic activity	GO:0009055: electron carrier activity;GO:0030385:ferredoxin:thioredoxin reductase activity;GO:0051539:4 iron, 4 sulfur cluster binding	GO:0009570:chloroplast stroma;GO:0009941:chloroplast envelope

ppe-miR7122b-5p	18784904	PRUPE_ppa012628mg	NCBI_Assembly:GCF_000346465.:	NW_006760324.1	+	25560108	25563302	3194	pper04120:Ubiquitin mediated proteolysis;pper04141:Protein processing in endoplasmic reticulum	GO:0006301:postreplication repair;GO:0006635:fatty acid beta-oxidation;GO:0009407:toxin catabolic process;GO:0010286:heat acclimation;GO:0042023:DNA endoreduplication;GO:0043161:proteasome-mediated ubiquitin-dependent protein catabolic process;GO:0051510:regulation of unidimensional cell growth;GO:0051788:response to misfolded protein;GO:0080129:proteasome core complex assembly	GO:0016874:ligase activity	-

ppe-miR7122b-5p	18786590	PRUPE_ppa018404mg	NCBI_Assembly:GCF_000346465.:	NW_006760324.1	-	19963740	19966574	2834	pper01100:Metabolic pathways;pper01110:Biosynthesis of secondary metabolites;pper00460:Cyanoamino acid metabolism;pper00500:Starch and sucrose metabolism;pper00940:Phenylpropanoid biosynthesis	GO:0005975: carbohydrate metabolic process	GO:0004553: hydrolase activity, hydrolyzing O-glycosyl compounds	-
ppe-miR7122b-5p	18786658	PRUPE_ppa022308mg	NCBI_Assembly:GCF_000346465.:	NW_006760324.1	-	24289466	24291023	1557	pper01100:Metabolic pathways;pper00500:Starch and sucrose metabolism;pper00040:Pentose and glucuronate interconversions	GO:0042545: cell wall modification;GO:0045490:pectin catabolic process	GO:0004857: enzyme inhibitor activity;GO:0030599:pectinesterase activity;GO:0045330:aspartyl esterase activity	GO:0005618:cell wall
ppe-miR7122b-5p	18788484	PRUPE_ppa015548mg	NCBI_Assembly:GCF_000346465.:	NW_006760385.1	-	32189550	32197047	7497	-	-	GO:0004713: protein tyrosine kinase activity;GO:0005524:ATP binding;GO:0016597:amino acid binding	-

ppe-miR7122b-5p	18788669	PRUPE_ppa009157mg	NCBI_Assembly:GCF_000346465.:	NW_006760385.1	+	42262608	42270789	8181	pper03040:Spliceosome	GO:0006397:mRNA processing;GO:0010413:glucuronoxylan metabolic process;GO:0045492:xylan biosynthetic process	GO:0003723:RNA binding	GO:0005634:nucleus;GO:0005737:cytoplasm
ppe-miR7122b-5p	18788914	PRUPE_ppa003357mg	NCBI_Assembly:GCF_000346465.:	NW_006760385.1	-	35166169	35170745	4576	pper04144:Endocytosis	GO:0006623:protein targeting to vacuole	-	GO:0005884:actin filament;GO:0009579:thylakoid
ppe-miR7122b-5p	18789476	PRUPE_ppa013518mg	NCBI_Assembly:GCF_000346465.:	NW_006760385.1	+	8148425	8150167	1742	pper04140:Regulation of autophagy	GO:0006914:autophagy;GO:0046482:para-aminobenzoic acid metabolic process	-	-
ppe-miR7122b-5p	18789898	PRUPE_ppa018096mg	NCBI_Assembly:GCF_000346465.:	NW_006760385.1	-	1205597	1206186	589	-	-	-	-
ppe-miR7122b-5p	18790027	PRUPE_ppa019344mg	NCBI_Assembly:GCF_000346465.:	NW_006760385.1	+	35398877	35402139	3262	-	GO:0006457:protein folding	-	-
ppe-miR7122b-5p	18790578	PRUPE_ppa024286mg	NCBI_Assembly:GCF_000346465.:	NW_006760385.1	-	36356538	36361326	4788	-	-	GO:0004674:protein serine/threonine kinase activity;GO:0005524:ATP binding	-
ppe-miR7122b-5p	18791066	PRUPE_ppa003031mg	NCBI_Assembly:GCF_000346465.:	NW_006760385.1	+	27755997	27760130	4133	-	-	GO:0003993:acid phosphatase activity;GO:0046872:metal ion binding	-
ppe-miR7122b-5p	18791090	PRUPE_ppa001362mg	NCBI_Assembly:GCF_000346465.:	NW_006760385.1	-	23698613	23703659	5046	-	GO:0048765:root hair cell differentiation	-	-
ppe-miR7122b-5p	18791114	PRUPE_ppa026546mg	NCBI_Assembly:GCF_000346465.:	NW_006760385.1	-	20425046	20426611	1565	-	-	-	-

ppe-miR7122b-5p	18793043	PRUPE_ppa014411mg	NCBI_Assembly:GCF_000346465.:	NW_006760385.1	-	41021209	41022565	1356	-	-	-	-
ppe-miR8133-3p	18766108	PRUPE_ppa023098mg	NCBI_Assembly:GCF_000346465.:	NW_006760186.1	-	368269	369827	1558	-	-	-	-
ppe-miR8133-3p	18766505	PRUPE_ppa017960mg	NCBI_Assembly:GCF_000346465.:	NW_006760194.1	+	19144620	19146860	2240	-	-	GO:0004672: protein kinase activity;GO:0005524:ATP binding	-
ppe-miR8133-3p	18767295	PRUPE_ppa020442mg	NCBI_Assembly:GCF_000346465.:	NW_006760194.1	-	13086418	13086855	437	-	-	-	-
ppe-miR8133-3p	18767327	PRUPE_ppa005336mg	NCBI_Assembly:GCF_000346465.:	NW_006760194.1	-	13627785	13631045	3260	-	-	-	-
ppe-miR8133-3p	18767934	PRUPE_ppa005313mg	NCBI_Assembly:GCF_000346465.:	NW_006760194.1	-	18065840	18068507	2667	pper01100:Metabolic pathways;pper00790:Folate biosynthesis	GO:0046654:tetrahydrofolate biosynthetic process	GO:0003934: GTP cyclohydrolase I activity	GO:0005737:cytoplasm
ppe-miR8133-3p	18768203	PRUPE_ppa023695mg	NCBI_Assembly:GCF_000346465.:	NW_006760194.1	+	319382	320371	989	-	-	GO:0016787: hydrolase activity	-
ppe-miR8133-3p	18768512	PRUPE_ppa023105mg	NCBI_Assembly:GCF_000346465.:	NW_006760194.1	-	3958897	3962368	3471	-	-	-	-
ppe-miR8133-3p	18769137	PRUPE_ppa011232mg	NCBI_Assembly:GCF_000346465.:	NW_006760201.1	+	19919190	19922228	3038	pper04144:Endocytosis	GO:0007034: vacuolar transport	-	GO:0005622:intracellular
ppe-miR8133-3p	18769562	PRUPE_ppa011524mg	NCBI_Assembly:GCF_000346465.:	NW_006760201.1	+	21362513	21363679	1166	-	-	GO:0009055: electron carrier activity	-
ppe-miR8133-3p	18769642	PRUPE_ppa003398mg	NCBI_Assembly:GCF_000346465.:	NW_006760201.1	+	21907250	21910147	2897	-	-	-	-
ppe-miR8133-3p	18769856	PRUPE_ppa023464mg	NCBI_Assembly:GCF_000346465.:	NW_006760201.1	+	16798886	16800040	1154	-	-	-	-
ppe-miR8133-3p	18769889	PRUPE_ppa020819mg	NCBI_Assembly:GCF_000346465.:	NW_006760201.1	+	8182897	8183254	357	-	-	-	-
ppe-miR8133-3p	18770154	PRUPE_ppb022069mg	NCBI_Assembly:GCF_000346465.:	NW_006760201.1	+	2734355	2736205	1850	-	-	GO:0003676: nucleic acid binding;GO:0008270:zinc ion binding	-
ppe-miR8133-3p	18770415	PRUPE_ppa021441mg	NCBI_Assembly:GCF_000346465.:	NW_006760201.1	-	9997587	10002233	4646	-	GO:0007165: signal transduction	GO:0005524: ATP binding;GO:0043531:ADP binding	-
ppe-miR8133-3p	18770507	PRUPE_ppa023129mg	NCBI_Assembly:GCF_000346465.:	NW_006760201.1	-	12042014	12043625	1611	-	-	-	-

ppe-miR8133-3p	18771885	PRUPE_ppa024770mg	NCBI_Assembly:GCF_000346465.:	NW_006760201.1	+	1746961	1747406	445	pper04120:Ubiquitin mediated proteolysis;pper04141:Protein processing in endoplasmic reticulum	GO:0006511: ubiquitin-dependent protein catabolic process	-	-
ppe-miR8133-3p	18772262	PRUPE_ppa015995mg	NCBI_Assembly:GCF_000346465.:	NW_006760208.1	-	24617226	24618398	1172	-	-	-	-
ppe-miR8133-3p	18772295	PRUPE_ppa018594mg	NCBI_Assembly:GCF_000346465.:	NW_006760208.1	-	1901111	1901431	320	-	-	GO:0004672: protein kinase activity;GO:0005524:ATP binding	-
ppe-miR8133-3p	18772700	PRUPE_ppa001602mg	NCBI_Assembly:GCF_000346465.:	NW_006760208.1	-	23074357	23080451	6094	-	GO:0006399:tRNA metabolic process;GO:0009902:chloroplast relocation;GO:0010027:thylakoid membrane organization	GO:0000175: 3'-5'-exoribonuclease activity	GO:0005623:cell

ppe-miR8133-3p	18772871	PRUPE_ppa009156mg	NCBI_Assembly:GCF_000346465.:	NW_006760208.1	-	1052688	1055813	3125	-	GO:0006353: DNA-templated transcription, termination;GO:0009073:aromatic amino acid family biosynthetic process;GO:0010103:stomatal complex morphogenesis;GO:0016226:iron-sulfur cluster assembly;GO:0042793:transcription from plastid promoter;GO:0045893:positive regulation of transcription, DNA-templated	GO:0003723: RNA binding	GO:0009507:chloroplast
ppe-miR8133-3p	18774286	PRUPE_ppa007298mg	NCBI_Assembly:GCF_000346465.:	NW_006760208.1	-	13893331	13897325	3994	-	-	GO:0004672: protein kinase activity;GO:0005524:ATP binding	-
ppe-miR8133-3p	18774412	PRUPE_ppa026062mg	NCBI_Assembly:GCF_000346465.:	NW_006760208.1	+	22067135	22067493	358	-	-	-	-
ppe-miR8133-3p	18774585	PRUPE_ppa024060mg	NCBI_Assembly:GCF_000346465.:	NW_006760208.1	-	5168719	5170017	1298	-	-	-	-
ppe-miR8133-3p	18774821	PRUPE_ppa008870mg	NCBI_Assembly:GCF_000346465.:	NW_006760208.1	-	13330802	13334753	3951	-	GO:0006281: DNA repair	-	GO:0030915:Smc5-Smc6 complex
ppe-miR8133-3p	18775347	PRUPE_ppa008565mg	NCBI_Assembly:GCF_000346465.:	NW_006760208.1	-	18537225	18539186	1961	-	-	-	GO:0005634:nucleus
ppe-miR8133-3p	18775903	PRUPE_ppa015862mg	NCBI_Assembly:GCF_000346465.:	NW_006760212.1	+	11156982	11158598	1616	-	-	-	-

ppe-miR8133-3p	18776167	PRUPE_ppa006896mg	NCBI_Assembly:GCF_000346465.:	NW_006760212.1	+	17250811	17253714	2903	-	GO:0000398: mRNA splicing, via spliceosome; GO:0006470: protein dephosphorylation;GO:0030422:production of siRNA involved in RNA interference;GO:0035196:production of miRNAs involved in gene silencing by miRNA	-	-
ppe-miR8133-3p	18776201	PRUPE_ppa001510mg	NCBI_Assembly:GCF_000346465.:	NW_006760212.1	+	18445436	18453726	8290	-	-	-	GO:0016021:integral component of membrane

ppe-miR8133-3p	18776908	PRUPE_ppa006801mg	NCBI_Assembly:GCF_000346465.:	NW_006760212.1	-	12695757	12697169	1412	-	GO:0006351:transcription, DNA-templated;GO:0009809:lignin biosynthetic process;GO:0009834:plant-type secondary cell wall biogenesis;GO:0010047:fruit dehiscence;GO:0010413:glucuronoxylan metabolic process;GO:0045492:xylan biosynthetic process;GO:0045893:positive regulation of transcription, DNA-templated	GO:0003700:sequence-specific DNA binding transcription factor activity;GO:0043565:sequence-specific DNA binding	GO:0005634:nucleus
ppe-miR8133-3p	18777299	PRUPE_ppa021585mg	NCBI_Assembly:GCF_000346465.:	NW_006760212.1	+	4146224	4146589	365	-	-	-	-
ppe-miR8133-3p	18777315	PRUPE_ppa023438mg	NCBI_Assembly:GCF_000346465.:	NW_006760212.1	+	1166303	1170007	3704	-	-	GO:0003677:DNA binding	-
ppe-miR8133-3p	18777708	PRUPE_ppa014792mg	NCBI_Assembly:GCF_000346465.:	NW_006760212.1	+	3416210	3420889	4679	-	GO:0006355:regulation of transcription, DNA-templated	GO:0008289:lipid binding;GO:0043565:sequence-specific DNA binding	GO:0005634:nucleus
ppe-miR8133-3p	18779240	PRUPE_ppa015395mg	NCBI_Assembly:GCF_000346465.:	NW_006760220.1	+	1620052	1620498	446	-	-	-	-

ppe-miR8133-3p	18779444	PRUPE_ppa010636mg	NCBI_Assembly:GCF_000346465.:	NW_006760220.1	-	27917465	27920784	3319	pper03050:Proteasome	GO:0051603:proteolysis involved in cellular protein catabolic process	GO:0004298:threonine-type endopeptidase activity	GO:0005634:nucleus;GO:0005737:cytoplasm;GO:0005839:proteasome core complex
ppe-miR8133-3p	18779459	PRUPE_ppa022523mg	NCBI_Assembly:GCF_000346465.:	NW_006760220.1	+	1038744	1041555	2811	-	-	GO:0015238:drug transmembrane transporter activity;GO:0015297:antiporter activity	GO:0016021:integral component of membrane
ppe-miR8133-3p	18780101	PRUPE_ppa026706mg	NCBI_Assembly:GCF_000346465.:	NW_006760220.1	+	1884731	1885678	947	-	-	-	-
ppe-miR8133-3p	18781530	PRUPE_ppa025063mg	NCBI_Assembly:GCF_000346465.:	NW_006760220.1	-	7818909	7825543	6634	-	-	-	-
ppe-miR8133-3p	18783089	PRUPE_ppa021818mg	NCBI_Assembly:GCF_000346465.:	NW_006760268.1	-	12413189	12413500	311	-	-	-	-
ppe-miR8133-3p	18783248	PRUPE_ppa019369mg	NCBI_Assembly:GCF_000346465.:	NW_006760268.1	-	7501106	7501694	588	-	-	GO:0003676:nucleic acid binding;GO:0008270:zinc ion binding	-
ppe-miR8133-3p	18783391	PRUPE_ppa013398mg	NCBI_Assembly:GCF_000346465.:	NW_006760268.1	+	17148798	17149326	528	pper03010:Ribosome	GO:0006412:translation	GO:0003735:structural constituent of ribosome	GO:0005840:ribosome
ppe-miR8133-3p	18783431	PRUPE_ppa019290mg	NCBI_Assembly:GCF_000346465.:	NW_006760268.1	+	2073232	2075289	2057	-	-	-	-
ppe-miR8133-3p	18783820	PRUPE_ppa001180mg	NCBI_Assembly:GCF_000346465.:	NW_006760268.1	+	14808062	14814233	6171	pper03018:RNA degradation	-	-	-
ppe-miR8133-3p	18784266	PRUPE_ppa000075mg	NCBI_Assembly:GCF_000346465.:	NW_006760268.1	+	20814066	20828873	14807	pper03013:RNA transport	-	-	-
ppe-miR8133-3p	18784449	PRUPE_ppa010471mg	NCBI_Assembly:GCF_000346465.:	NW_006760268.1	+	9873538	9874589	1051	-	-	-	-
ppe-miR8133-3p	18784839	PRUPE_ppa017483mg	NCBI_Assembly:GCF_000346465.:	NW_006760324.1	-	2465100	2465309	209	-	-	GO:0008137:NADH dehydrogenase (ubiquinone) activity	-

ppe-miR8133-3p	18784998	PRUPE_ppa011831mg	NCBI_Assembly:GCF_000346465.:	NW_006760324.1	-	24346319	24347190	871	-	-	GO:0004857: enzyme inhibitor activity;GO:0030599:pectinesterase activity	-
ppe-miR8133-3p	18785049	PRUPE_ppa001878mg	NCBI_Assembly:GCF_000346465.:	NW_006760324.1	-	21633041	21635528	2487	-	-	GO:0005524: ATP binding;GO:0016887:ATPase activity	GO:0016020:membrane
ppe-miR8133-3p	18785434	PRUPE_ppa009040mg	NCBI_Assembly:GCF_000346465.:	NW_006760324.1	-	15171070	15176978	5908	pper04140:Regulation of autophagy	GO:0000902: cell morphogenesis;GO:0006623:protein targeting to vacuole;GO:0006914:autophagy;GO:0016049:cell growth;GO:0048193:Golgi vesicle transport	-	GO:0005829:cytosol
ppe-miR8133-3p	18785435	PRUPE_ppb023590mg	NCBI_Assembly:GCF_000346465.:	NW_006760324.1	-	19446494	19448066	1572	-	GO:0006886:intracellular protein transport	-	GO:0005622:intracellular
ppe-miR8133-3p	18785502	PRUPE_ppa013025mg	NCBI_Assembly:GCF_000346465.:	NW_006760324.1	-	22833998	22834872	874	-	-	-	-
ppe-miR8133-3p	18785686	PRUPE_ppa023598mg	NCBI_Assembly:GCF_000346465.:	NW_006760324.1	+	12383632	12388101	4469	-	GO:0015074: DNA integration	GO:0003676: nucleic acid binding;GO:0008270:zinc ion binding	-

ppe-miR8133-3p	18785910	PRUPE_ppa015423mg	NCBI_Assembly:GCF_000346465.:	NW_006760324.1	-	20091809	20094922	3113	pper01100:Metabolic pathways;pper01110:Biosynthesis of secondary metabolites;pper00940:Phenylpropanoid biosynthesis	-	GO:0008171: O-methyltransferase activity	-
ppe-miR8133-3p	18786160	PRUPE_ppa026681mg	NCBI_Assembly:GCF_000346465.:	NW_006760324.1	-	5729441	5731291	1850	-	-	GO:0003676: nucleic acid binding;GO:0008270:zinc ion binding	-
ppe-miR8133-3p	18786233	PRUPE_ppa017325mg	NCBI_Assembly:GCF_000346465.:	NW_006760324.1	+	7649757	7651461	1704	-	-	-	-
ppe-miR8133-3p	18786379	PRUPE_ppa026491mg	NCBI_Assembly:GCF_000346465.:	NW_006760324.1	-	5734020	5735967	1947	-	-	GO:0003676: nucleic acid binding;GO:0008270:zinc ion binding	-
ppe-miR8133-3p	18786919	PRUPE_ppa001323mg	NCBI_Assembly:GCF_000346465.:	NW_006760324.1	-	16762797	16770148	7351	-	GO:0007018: microtubule-based movement;GO:0048364:root development	GO:0003777: microtubule motor activity;GO:0005524:ATP binding	GO:0005871:kinesin complex;GO:0005874:microtubule
ppe-miR8133-3p	18787059	PRUPE_ppa010898mg	NCBI_Assembly:GCF_000346465.:	NW_006760324.1	-	9850890	9853291	2401	-	-	GO:0003746:translation elongation factor activity	GO:0005853:eukaryotic translation elongation factor 1 complex
ppe-miR8133-3p	18787213	PRUPE_ppa021061mg	NCBI_Assembly:GCF_000346465.:	NW_006760324.1	-	1947168	1951535	4367	-	-	GO:0043531: ADP binding	-

ppe-miR8133-3p	18787309	PRUPE_ppa005193mg	NCBI_Assembly:GCF_000346465.:	NW_006760324.1	-	10279200	10287314	8114	pper01100:Metabolic pathways;pper01110:Biosynthesis of secondary metabolites;pper01230:Biosynthesis of amino acids;pper01210:2-Oxocarboxylic acid metabolism;pper01200:Carbon metabolism;pper00630:Glyoxylate and dicarboxylate metabolism;pper00020:Citrate cycle (TCA cycle)	GO:0006099:tricarboxylic acid cycle;GO:0044262:cellular carbohydrate metabolic process	GO:0004108: citrate (Si)-synthase activity	-
ppe-miR8133-3p	18787554	PRUPE_ppa006571m1g	NCBI_Assembly:GCF_000346465.:	NW_006760324.1	-	26382199	26385400	3201	-	-	-	-
ppe-miR8133-3p	18787693	PRUPE_ppa024369mg	NCBI_Assembly:GCF_000346465.:	NW_006760324.1	-	15762126	15763553	1427	-	-	-	-
ppe-miR8133-3p	18787710	PRUPE_ppa025564mg	NCBI_Assembly:GCF_000346465.:	NW_006760324.1	-	13205966	13206979	1013	-	-	-	-
ppe-miR8133-3p	18788817	PRUPE_ppa008750mg	NCBI_Assembly:GCF_000346465.:	NW_006760385.1	+	37913818	37917007	3189	-	-	-	-
ppe-miR8133-3p	18789255	PRUPE_ppa011676mg	NCBI_Assembly:GCF_000346465.:	NW_006760385.1	-	26496874	26497803	929	-	-	GO:0000166: nucleotide binding;GO:0003676:nucleic acid binding	-
ppe-miR8133-3p	18789429	PRUPE_ppa007861mg	NCBI_Assembly:GCF_000346465.:	NW_006760385.1	+	29875423	29877566	2143	-	-	-	-
ppe-miR8133-3p	18790003	PRUPE_ppa006508mg	NCBI_Assembly:GCF_000346465.:	NW_006760385.1	+	37215769	37219638	3869	-	GO:0045727: positive regulation of translation;GO:0046777:protein autophosphorylation	GO:0004672: protein kinase activity;GO:0005524:ATP binding	-

ppe-miR8133-3p	18790261	PRUPE_ppa024315mg	NCBI_Assembly:GCF_000346465.:	NW_006760385.1	-	46378836	46379480	644	pper04626:Plant-pathogen interaction	-	GO:0005509: calcium ion binding	-
ppe-miR8133-3p	18790992	PRUPE_ppa005113mg	NCBI_Assembly:GCF_000346465.:	NW_006760385.1	+	39460086	39462259	2173	-	-	-	-
ppe-miR8133-3p	18791055	PRUPE_ppa014337mg	NCBI_Assembly:GCF_000346465.:	NW_006760385.1	+	44513827	44514293	466	-	-	-	-
ppe-miR8133-3p	18791063	PRUPE_ppa024170mg	NCBI_Assembly:GCF_000346465.:	NW_006760385.1	+	46428807	46431059	2252	pper00910:Nitrogen metabolism	GO:0015976: carbon utilization	GO:0004089: carbonate dehydratase activity;GO:0008270:zinc ion binding	GO:0005739:mitochondrion
ppe-miR8133-3p	18791086	PRUPE_ppa008937mg	NCBI_Assembly:GCF_000346465.:	NW_006760385.1	-	41605101	41607997	2896	-	GO:0045454: cell redox homeostasis	-	GO:0005623:cell
ppe-miR8133-3p	18792482	PRUPE_ppa022136mg	NCBI_Assembly:GCF_000346465.:	NW_006760385.1	-	44547973	44550003	2030	-	-	-	-
ppe-miR8133-3p	18793118	PRUPE_ppa023232mg	NCBI_Assembly:GCF_000346465.:	NW_006760385.1	+	12097963	12098870	907	-	-	-	-
ppe-miR8133-3p	18793662	PRUPE_ppa023948mg	NCBI_Assembly:GCF_000346465.:	NW_006760385.1	+	15788796	15789071	275	-	-	GO:0004672: protein kinase activity;GO:0005524:ATP binding	-
ppe-miR8133-3p	18793907	PRUPE_ppa017723mg	NCBI_Assembly:GCF_000346465.:	NW_006760385.1	-	17752598	17753549	951	-	-	-	-
