# Supplementary material for: Identification and characterization of Prunus persica miRNAs in response to UVB radiation in greenhouse through high-throughput sequencing
Source: BMC Genomics. 2017 Dec 2;18:938. doi: 10.1186/s12864-017-4347-5 (PMC5712094; doi:10.1186/s12864-017-4347-5)
Supplement: Supplementary file 4 — Details of targets genes and their annotation, GO classification, and KEGG pathway for the known miRNAs. (PDF 2823 kb) [file 12864_2017_4347_MOESM4_ESM.pdf]

#microRNA	gene_id	gene_name	genome_version	scaffold_id	strand	start_position	end_position	length	Pathway - KEGG PATHWAY	GO - Biological Process	GO - Molecular Function	GO - Cellular Component
Pp03_19842-3p	18766057	PRUPE_ppa011719mg	NCBI_Assembly:GCF_000346465.1	NW_006760186.1	-	574283	574985	702	-	-	GO:0000166:nucleotide binding;GO:0003676:nucleic acid binding	-
Pp03_19842-3p	18766062	PRUPE_ppb015926mg	NCBI_Assembly:GCF_000346465.1	NW_006760186.1	+	487504	487725	221	-	-	-	-
Pp03_19842-3p	18766068	PRUPE_ppa019766mg	NCBI_Assembly:GCF_000346465.1	NW_006760186.1	-	263781	265739	1958	-	-	-	-
Pp03_19842-3p	18766130	PRUPE_ppa004741mg	NCBI_Assembly:GCF_000346465.1	NW_006760186.1	-	966987	970106	3119	-	-	-	-
Pp03_19842-3p	18766179	PRUPE_ppb009745mg	NCBI_Assembly:GCF_000346465.1	NW_006760194.1	-	4772535	4774941	2406	pper01100:Metabolic pathways;pper00600:Sphingolipid metabolism	GO:0006633:fatty acid biosynthetic process	GO:0005506:iron ion binding;GO:0016491:oxidoreductase activity	-
Pp03_19842-3p	18766275	PRUPE_ppa015287mg	NCBI_Assembly:GCF_000346465.1	NW_006760194.1	+	15451503	15456874	5371	-	GO:0055085:transmembrane transport	GO:0005215:transporter activity	GO:0016020:membrane
Pp03_19842-3p	18766505	PRUPE_ppa017960mg	NCBI_Assembly:GCF_000346465.1	NW_006760194.1	+	19144620	19146860	2240	-	-	GO:0004672:protein kinase activity;GO:0005524:ATP binding	-
Pp03_19842-3p	18766568	PRUPE_ppa023318mg	NCBI_Assembly:GCF_000346465.1	NW_006760194.1	-	8219531	8221782	2251	-	-	-	-

Pp03_19842-3p	18766630	PRUPE_ppa004697mg	NCBI_Assembly:GCF_000346465.1	NW_006760194.1	+	17529228	17533017	3789	-	GO:0006351:transcription, DNA-templated;GO:0009294:DNA mediated transformation;GO:0009405:pathogenesis;GO:0009861:jasmonic acid and ethylene-dependent systemic resistance;GO:0016573:histone acetylation;GO:0045892:negative regulation of transcription, DNA-templated;GO:2000026:regulation of multicellular organismal development	GO:0032041:NAD-dependent histone deacetylase activity (H3-K14 specific);GO:0046969:NAD-dependent histone deacetylase activity (H3-K9 specific);GO:0046970:NAD-dependent histone deacetylase activity (H4-K16 specific);GO:0097372:NAD-dependent histone deacetylase activity (H3-K18 specific)	GO:0005634:nucleus
Pp03_19842-3p	18766631	PRUPE_ppa024072mg	NCBI_Assembly:GCF_000346465.1	NW_006760194.1	-	20673352	20674006	654	-	-	-	-
Pp03_19842-3p	18766697	PRUPE_ppa018501mg	NCBI_Assembly:GCF_000346465.1	NW_006760194.1	-	20270757	20278479	7722	-	-	GO:0003677:DNA binding;GO:0008270:zinc ion binding	-
Pp03_19842-3p	18766725	PRUPE_ppa018793mg	NCBI_Assembly:GCF_000346465.1	NW_006760194.1	+	10415579	10416181	602	-	-	GO:0043531:ADP binding	-

Pp03_19842-3p	18766825	PRUPE_ppa010338mg	NCBI_Assembly:GCF_000346465.1	NW_006760194.1	+	21231002	21233234	2232	pper01100:Metabolic pathways;pper00730:Thiamine metabolism	GO:0006772:thiamine metabolic process;GO:0009229:thiamine diphosphate biosynthetic process	GO:0004788:thiamine diphosphokinase activity;GO:0005524:ATP binding;GO:0030975:thiamine binding	-
Pp03_19842-3p	18766889	PRUPE_ppa019971mg	NCBI_Assembly:GCF_000346465.1	NW_006760194.1	-	18823731	18829264	5533	-	GO:0006139:nucleobase-containing compound metabolic process;GO:0006357:regulation of transcription from RNA polymerase II promoter;GO:0032784:regulation of DNA-templated transcription, elongation	GO:0003676:nucleic acid binding	-
Pp03_19842-3p	18766955	PRUPE_ppa018278mg	NCBI_Assembly:GCF_000346465.1	NW_006760194.1	+	7257073	7258422	1349	-	-	GO:0005215:transporter activity	GO:0016020:membrane
Pp03_19842-3p	18767061	PRUPE_ppa011663mg	NCBI_Assembly:GCF_000346465.1	NW_006760194.1	-	13195698	13197462	1764	-	GO:0009269:response to desiccation;GO:0009409:response to cold;GO:0009651:response to salt stress	-	GO:0005773:vacuole

Pp03_19842-3p	18767265	PRUPE_ppa012572mg	NCBI_Assembly:GCF_000346465.1	NW_006760194.1	-	17737096	17737822	726	-	GO:0006511:ubiquitin-dependent protein catabolic process;GO:0009853:photorespiration;GO:0051788:response to misfolded protein;GO:0080129:proteasome core complex assembly	GO:0009055:electron carrier activity;GO:0051536:iron-sulfur cluster binding	GO:0005739:mitochondrion;GO:0009507:chloroplast
Pp03_19842-3p	18767274	PRUPE_ppa009717mg	NCBI_Assembly:GCF_000346465.1	NW_006760194.1	+	297446	301902	4456	-	-	-	-
Pp03_19842-3p	18767313	PRUPE_ppb009396mg	NCBI_Assembly:GCF_000346465.1	NW_006760194.1	+	17570442	17573539	3097	-	-	-	-
Pp03_19842-3p	18767671	PRUPE_ppb018208mg	NCBI_Assembly:GCF_000346465.1	NW_006760194.1	+	20745939	20746858	919	-	-	-	-
Pp03_19842-3p	18768822	PRUPE_ppa003116mg	NCBI_Assembly:GCF_000346465.1	NW_006760194.1	+	12218379	12221480	3101	pper04626:Plant-pathogen interaction	-	GO:0005216:ion channel activity	GO:0016021:integral component of membrane
Pp03_19842-3p	18768922	PRUPE_ppa016084mg	NCBI_Assembly:GCF_000346465.1	NW_006760194.1	-	9584784	9589700	4916	-	GO:0015074:DNA integration	GO:0003676:nucleic acid binding	GO:0005634:nucleus
Pp03_19842-3p	18768955	PRUPE_ppa026522mg	NCBI_Assembly:GCF_000346465.1	NW_006760194.1	-	7606110	7606472	362	pper04626:Plant-pathogen interaction;pper04075:Plant hormone signal transduction	-	-	GO:0005576:extracellular region
Pp03_19842-3p	18769174	PRUPE_ppa025136mg	NCBI_Assembly:GCF_000346465.1	NW_006760201.1	-	4682592	4684028	1436	-	-	-	-

Pp03_19842-3p	18769379	PRUPE_ppa005408mg	NCBI_Assembly:GCF_000346465.1	NW_006760201.1	-	17966371	17969174	2803	-	-	-	GO:0016021:integral component of membrane
Pp03_19842-3p	18769478	PRUPE_ppa011348mg	NCBI_Assembly:GCF_000346465.1	NW_006760201.1	+	18564222	18564953	731	-	-	GO:0008270:zinc ion binding	-
Pp03_19842-3p	18769519	PRUPE_ppa009651mg	NCBI_Assembly:GCF_000346465.1	NW_006760201.1	-	13383717	13384666	949	pper03018:RNA degradation	-	GO:0003676:nucleic acid binding	GO:0005634:nucleus
Pp03_19842-3p	18769525	PRUPE_ppa000956mg	NCBI_Assembly:GCF_000346465.1	NW_006760201.1	+	13135139	13139090	3951	-	-	GO:0004674:protein serine/threonine kinase activity;GO:0005524:ATP binding	GO:0016021:integral component of membrane
Pp03_19842-3p	18769596	PRUPE_ppa009030mg	NCBI_Assembly:GCF_000346465.1	NW_006760201.1	+	1388379	1393102	4723	pper04146:Peroxisome	-	-	GO:0009941:chloroplast envelope;GO:0016021:integral component of membrane
Pp03_19842-3p	18769705	PRUPE_ppa011155mg	NCBI_Assembly:GCF_000346465.1	NW_006760201.1	-	12560112	12561308	1196	-	GO:0006950:response to stress	-	-
Pp03_19842-3p	18769743	PRUPE_ppa024560mg	NCBI_Assembly:GCF_000346465.1	NW_006760201.1	-	12225955	12226530	575	-	-	-	-
Pp03_19842-3p	18769760	PRUPE_ppa009094mg	NCBI_Assembly:GCF_000346465.1	NW_006760201.1	+	21753887	21755939	2052	-	-	-	-

Pp03_19842-3p	18769805	PRUPE_ppa004813mg	NCBI_Assembly:GCF_000346465.1	NW_006760201.1	+	17678639	17680538	1899	-	GO:0006270:DNA replication initiation;GO:0006275:regulation of DNA replication;GO:0006306:DNA methylation; GO:0008283:cell proliferation; GO:0051567:histone H3-K9 methylation; GO:0051726:regulation of cell cycle	-	-
Pp03_19842-3p	18769836	PRUPE_ppa011498mg	NCBI_Assembly:GCF_000346465.1	NW_006760201.1	-	4338406	4343373	4967	pper01100:Metabolic pathways;pper00230:Purine metabolism;pper00920:Sulfur metabolism	GO:0000103:sulfate assimilation; GO:0048232:male gamete generation;GO:0070814:hydrogen sulfide biosynthetic process	GO:0004020:adenylylsulfate kinase activity;GO:0005524:ATP binding	GO:0005829:cytosol
Pp03_19842-3p	18769857	PRUPE_ppa009223mg	NCBI_Assembly:GCF_000346465.1	NW_006760201.1	+	19781231	19782354	1123	-	-	-	-

Pp03_19842-3p	18769873	PRUPE_ppa004832mg	NCBI_Assembly:GCF_000346465.1	NW_006760201.1	-	19041634	19043368	1734	-	-	GO:0004497:monooxygenase activity;GO:0005506:iron ion binding;GO:0016705:oxidoreductase activity, acting on paired donors, with incorporation or reduction of molecular oxygen;GO:0020037:heme binding	-
Pp03_19842-3p	18770207	PRUPE_ppa021998mg	NCBI_Assembly:GCF_000346465.1	NW_006760201.1	-	12229159	12229755	596	-	-	-	-
Pp03_19842-3p	18770243	PRUPE_ppa010925mg	NCBI_Assembly:GCF_000346465.1	NW_006760201.1	-	4267134	4268180	1046	-	-	-	-
Pp03_19842-3p	18770258	PRUPE_ppa019391mg	NCBI_Assembly:GCF_000346465.1	NW_006760201.1	-	22363069	22365369	2300	-	GO:0007049:cell cycle;GO:0016556:mRNA modification	-	GO:0005634:nucleus
Pp03_19842-3p	18770374	PRUPE_ppa006202mg	NCBI_Assembly:GCF_000346465.1	NW_006760201.1	-	9381376	9385282	3906	-	GO:0006351:transcription, DNA-templated	GO:0003677:DNA binding;GO:0003700:sequence-specific DNA binding transcription factor activity	GO:0005634:nucleus
Pp03_19842-3p	18770427	PRUPE_ppa021008mg	NCBI_Assembly:GCF_000346465.1	NW_006760201.1	+	8688340	8690157	1817	-	-	-	-

Pp03_19842-3p	18770456	PRUPE_ppa005635mg	NCBI_Assembly:GCF_000346465.1	NW_006760201.1	-	15392669	15394021	1352	-	-	GO:0016747: transferase activity, transferring acyl groups other than amino-acyl groups	-
Pp03_19842-3p	18770468	PRUPE_ppa010124mg	NCBI_Assembly:GCF_000346465.1	NW_006760201.1	-	12236297	12239517	3220	pper03040:Spliceosome	GO:0000398: mRNA splicing, via spliceosome; GO:0030422: production of siRNA involved in RNA interference; GO:0035196: production of miRNAs involved in gene silencing by miRNA	GO:0000166: nucleotide binding;GO:0003676:nucleic acid binding	GO:0005829: cytosol;GO:0016020:membrane;GO:0016607:nuclear speck
Pp03_19842-3p	18770659	PRUPE_ppa026980mg	NCBI_Assembly:GCF_000346465.1	NW_006760201.1	+	19429012	19429389	377	-	-	-	-
Pp03_19842-3p	18770902	PRUPE_ppa018690mg	NCBI_Assembly:GCF_000346465.1	NW_006760201.1	+	18904802	18906367	1565	-	-	-	-
Pp03_19842-3p	18770909	PRUPE_ppa003826mg	NCBI_Assembly:GCF_000346465.1	NW_006760201.1	-	15580301	15583711	3410	-	-	-	-
Pp03_19842-3p	18770956	PRUPE_ppa024186mg	NCBI_Assembly:GCF_000346465.1	NW_006760201.1	+	1534047	1538562	4515	-	-	GO:0008270: zinc ion binding	-
Pp03_19842-3p	18770988	PRUPE_ppa007215mg	NCBI_Assembly:GCF_000346465.1	NW_006760201.1	-	17521692	17523284	1592	-	-	-	-

Pp03_19842-3p	18771189	PRUPE_ppa001174mg	NCBI_Assembly:GCF_000346465.1	NW_006760201.1	+	9812105	9815333	3228	-	GO:0009733:response to auxin;GO:0009741:response to brassinosteroid	GO:0004672:protein kinase activity;GO:0005524:ATP binding	-
Pp03_19842-3p	18771272	PRUPE_ppa022131mg	NCBI_Assembly:GCF_000346465.1	NW_006760201.1	-	12018276	12018773	497	-	-	-	-
Pp03_19842-3p	18771335	PRUPE_ppa002608mg	NCBI_Assembly:GCF_000346465.1	NW_006760201.1	-	20559771	20563591	3820	-	-	GO:0008271:secondary active sulfate transmembrane transporter activity	GO:0016021:integral component of membrane
Pp03_19842-3p	18771336	PRUPE_ppb010366mg	NCBI_Assembly:GCF_000346465.1	NW_006760201.1	-	17780542	17781295	753	-	-	-	-
Pp03_19842-3p	18771350	PRUPE_ppa026273mg	NCBI_Assembly:GCF_000346465.1	NW_006760201.1	-	13353676	13355652	1976	-	-	-	GO:0016020:membrane
Pp03_19842-3p	18771376	PRUPE_ppa019400mg	NCBI_Assembly:GCF_000346465.1	NW_006760201.1	+	2173684	2175853	2169	-	-	-	-
Pp03_19842-3p	18771406	PRUPE_ppa007584mg	NCBI_Assembly:GCF_000346465.1	NW_006760201.1	+	15866102	15870803	4701	pper04075:Plant hormone signal transduction	-	GO:0004672:protein kinase activity;GO:0005524:ATP binding	-
Pp03_19842-3p	18771443	PRUPE_ppa024893mg	NCBI_Assembly:GCF_000346465.1	NW_006760201.1	-	10411124	10413697	2573	-	-	GO:0003676:nucleic acid binding;GO:0008270:zinc ion binding	-
Pp03_19842-3p	18771491	PRUPE_ppa024687mg	NCBI_Assembly:GCF_000346465.1	NW_006760201.1	+	16142834	16145927	3093	-	-	-	-
Pp03_19842-3p	18771551	PRUPE_ppa021357mg	NCBI_Assembly:GCF_000346465.1	NW_006760201.1	-	20299457	20299879	422	-	-	-	-

Pp03_19842-3p	18771686	PRUPE_ppa008196mg	NCBI_Assembly:GCF_000346465.1	NW_006760201.1	-		715756	719007	3251	-	-	-	-
Pp03_19842-3p	18771708	PRUPE_ppa006045mg	NCBI_Assembly:GCF_000346465.1	NW_006760201.1	-		20366760	20370535	3775	-	-	GO:0004674: protein serine/threonine kinase activity;GO:0005524:ATP binding	-
Pp03_19842-3p	18771750	PRUPE_ppa001635mg	NCBI_Assembly:GCF_000346465.1	NW_006760201.1	-		10363385	10367619	4234	-	-	-	-
Pp03_19842-3p	18771759	PRUPE_ppa008076mg	NCBI_Assembly:GCF_000346465.1	NW_006760201.1	+		21072481	21074670	2189	-	GO:0043687: post-translational protein modification; GO:0045893: positive regulation of transcription, DNA-templated	GO:0003700: sequence-specific DNA binding transcription factor activity;GO:0046872:metal ion binding	-
Pp03_19842-3p	18771785	PRUPE_ppa003063mg	NCBI_Assembly:GCF_000346465.1	NW_006760201.1	-		12839946	12844541	4595	-	-	-	-
Pp03_19842-3p	18771810	PRUPE_ppa024294mg	NCBI_Assembly:GCF_000346465.1	NW_006760201.1	-		12413919	12417324	3405	-	-	-	-
Pp03_19842-3p	18771868	PRUPE_ppa004069mg	NCBI_Assembly:GCF_000346465.1	NW_006760201.1	-		9889985	9894870	4885	pper04626:Plant-pathogen interaction	-	GO:0004672: protein kinase activity;GO:0005509:calcium ion binding;GO:0005524:ATP binding	-
Pp03_19842-3p	18771968	PRUPE_ppa002496mg	NCBI_Assembly:GCF_000346465.1	NW_006760208.1	+		28652156	28655388	3232	-	-	-	GO:0005829: cytosol

Pp03_19842-3p	18772002	PRUPE_ppa019654mg	NCBI_Assembly:GCF_000346465.1	NW_006760208.1	-	10817959	10821870	3911	-	GO:0009073:aromatic amino acid family biosynthetic process;GO:0016226:iron-sulfur cluster assembly	-	-
Pp03_19842-3p	18772101	PRUPE_ppa025531mg	NCBI_Assembly:GCF_000346465.1	NW_006760208.1	+	8375673	8378296	2623	-	-	-	-
Pp03_19842-3p	18772102	PRUPE_ppa014218mg	NCBI_Assembly:GCF_000346465.1	NW_006760208.1	+	4236409	4236977	568	-	-	-	-
Pp03_19842-3p	18772144	PRUPE_ppa009158mg	NCBI_Assembly:GCF_000346465.1	NW_006760208.1	-	26504544	26507768	3224	-	GO:0007015:actin filament organization;GO:0030048:actin filament-based movement;GO:0051016:barbed-end actin filament capping;GO:0051645:Golgi localization;GO:0051646:mitochondrion localization;GO:0060151:peroxisome localization	-	GO:0005634:nucleus;GO:0008290:F-actin capping protein complex

Pp03_19842-3p	18772273	PRUPE_ppa012044mg	NCBI_Assembly:GCF_000346465.1	NW_006760208.1	-	4338498	4341760	3262	-	GO:0006098: pentose-phosphate shunt;GO:0009561:megagametogenesis;GO:0009902:chloroplast relocation;GO:0010027:thylakoid membrane organization;GO:0010304:PSII associated light-harvesting complex II catabolic process;GO:0015995:chlorophyll biosynthetic process;GO:0016117:carotenoid biosynthetic process;GO:0019288:isopentenyl diphosphate biosynthetic process, methylerythritol 4-phosphate pathway;GO:	-	GO:0009535: chloroplast thylakoid membrane
Pp03_19842-3p	18772417	PRUPE_ppa023743mg	NCBI_Assembly:GCF_000346465.1	NW_006760208.1	-	4287306	4289421	2115	-	-	-	-

Pp03_19842-3p	18772600	PRUPE_ppa006696mg	NCBI_Assembly:GCF_000346465.1	NW_006760208.1	-	4698598	4700545	1947	pper04075:Plant hormone signal transduction	GO:0006470:protein dephosphorylation	GO:0004722:protein serine/threonine phosphatase activity;GO:0046872:metal ion binding	-
Pp03_19842-3p	18772791	PRUPE_ppa025514mg	NCBI_Assembly:GCF_000346465.1	NW_006760208.1	-	2760652	2764953	4301	-	-	GO:0004252:serine-type endopeptidase activity	-
Pp03_19842-3p	18772971	PRUPE_ppa011993mg	NCBI_Assembly:GCF_000346465.1	NW_006760208.1	+	4627193	4627935	742	-	-	-	-
Pp03_19842-3p	18773042	PRUPE_ppa001736mg	NCBI_Assembly:GCF_000346465.1	NW_006760208.1	+	22720101	22722419	2318	-	GO:0009793:embryo development ending in seed dormancy;GO:0010027:thylakoid membrane organization;GO:0010228:vegetative to reproductive phase transition of meristem;GO:0016226:iron-sulfur cluster assembly;GO:0048481:ovule development	-	-

Pp03_19842-3p	18774496	PRUPE_ppa004635mg	NCBI_Assembly:GCF_000346465.1	NW_006760208.1	+		21292415	21295206	2791	-	-	GO:0004497:monooxygenase activity;GO:0005506:iron ion binding;GO:0016705:oxidoreductase activity, acting on paired donors, with incorporation or reduction of molecular oxygen;GO:0020037:heme binding	-
Pp03_19842-3p	18774501	PRUPE_ppa025973mg	NCBI_Assembly:GCF_000346465.1	NW_006760208.1	+		20452259	20452567	308	-	-	-	-
Pp03_19842-3p	18774504	PRUPE_ppa001899mg	NCBI_Assembly:GCF_000346465.1	NW_006760208.1	-		25628843	25632596	3753	pper04712:Circadian rhythm - plant;pper04075:Plant hormone signal transduction	GO:0007623:circadian rhythm;GO:0009630:gravitropism;GO:0009704:de-etiolation;GO:0009740:gibberellic acid mediated signaling pathway;GO:0031539:positive regulation of anthocyanin metabolic process	GO:0003700:sequence-specific DNA binding transcription factor activity	-

Pp03_19842-3p	18775498	PRUPE_ppa024093mg	NCBI_Assembly:GCF_000346465.1	NW_006760208.1	-	11589328	11592711	3383	-	GO:0006313:transposition, DNA-mediated	GO:0003677:DNA binding;GO:0004803:transposase activity	-	
Pp03_19842-3p	18775818	PRUPE_ppa006286mg	NCBI_Assembly:GCF_000346465.1	NW_006760212.1	+	13848799	13851317	2518	-	-	-	-	
Pp03_19842-3p	18776100	PRUPE_ppa010157m2g	NCBI_Assembly:GCF_000346465.1	NW_006760212.1	-	5648909	5649436	527	-	-	-	-	
Pp03_19842-3p	18776723	PRUPE_ppa005561m2g	NCBI_Assembly:GCF_000346465.1	NW_006760212.1	-	10983914	10984029	115	-	-	-	-	
Pp03_19842-3p	18777216	PRUPE_ppa010414mg	NCBI_Assembly:GCF_000346465.1	NW_006760212.1	-	10668503	10672359	3856	pper00480:Glutathione metabolism	-	-	-	
Pp03_19842-3p	18777249	PRUPE_ppa002234mg	NCBI_Assembly:GCF_000346465.1	NW_006760212.1	-	16039648	16044046	4398	-	-	GO:0004672:protein kinase activity;GO:0005524:ATP binding	-	
Pp03_19842-3p	18777315	PRUPE_ppa023438mg	NCBI_Assembly:GCF_000346465.1	NW_006760212.1	+	1166303	1170007	3704	-	-	GO:0003677:DNA binding	-	
Pp03_19842-3p	18777321	PRUPE_ppa011569mg	NCBI_Assembly:GCF_000346465.1	NW_006760212.1	-	13979405	13981724	2319	-	-	GO:0046872:metal ion binding	-	
Pp03_19842-3p	18777338	PRUPE_ppa015858mg	NCBI_Assembly:GCF_000346465.1	NW_006760212.1	+	13928558	13930881	2323	-	GO:0006355:regulation of transcription, DNA-templated	GO:0003690:double-stranded DNA binding	GO:0005739:mitochondrion	
Pp03_19842-3p	18777399	PRUPE_ppa004675mg	NCBI_Assembly:GCF_000346465.1	NW_006760212.1	-	15049078	15051467	2389	-	-	-	-	
Pp03_19842-3p	18777483	PRUPE_ppa020525mg	NCBI_Assembly:GCF_000346465.1	NW_006760212.1	-	8850248	8854932	4684	-	-	-	-	

Pp03_19842-3p	18777509	PRUPE_ppa026240mg	NCBI_Assembly:GCF_000346465.1	NW_006760212.1	+	5997581	6001496	3915	-	-	GO:0003676:nucleic acid binding;GO:0008270:zinc ion binding	-
Pp03_19842-3p	18777575	PRUPE_ppa011938mg	NCBI_Assembly:GCF_000346465.1	NW_006760212.1	-	15545125	15547597	2472	-	GO:0001881:receptor recycling;GO:0006511:ubiquitin-dependent protein catabolic process;GO:0006623:protein targeting to vacuole;GO:0009853:photorespiration;GO:0016192:vesicle-mediated transport;GO:0016197:endosomal transport;GO:0051788:response to misfolded protein;GO:0080129:proteasome core complex assembly	-	GO:0005771:multivesicular body;GO:0030904:retromer complex
Pp03_19842-3p	18777721	PRUPE_ppa022818mg	NCBI_Assembly:GCF_000346465.1	NW_006760212.1	+	17899514	17903700	4186	-	-	GO:0015079:potassium ion transmembrane transporter activity	GO:0016021:integral component of membrane

Pp03_19842-3p	18777781	PRUPE_ppa007315mg	NCBI_Assembly:GCF_000346465.1	NW_006760212.1	+	6791012	6794632	3620	-	-	GO:0016787:hydrolase activity	GO:0005829:cytosol
Pp03_19842-3p	18777805	PRUPE_ppa008226mg	NCBI_Assembly:GCF_000346465.1	NW_006760212.1	-	8317904	8321733	3829	-	-	-	-
Pp03_19842-3p	18777814	PRUPE_ppa015317mg	NCBI_Assembly:GCF_000346465.1	NW_006760212.1	-	11856279	11858349	2070	-	-	GO:0004674:protein serine/threonine kinase activity;GO:0005524:ATP binding	-
Pp03_19842-3p	18777826	PRUPE_ppa020908mg	NCBI_Assembly:GCF_000346465.1	NW_006760212.1	-	4992196	4992471	275	-	-	-	GO:0009941:chloroplast envelope
Pp03_19842-3p	18777917	PRUPE_ppa001291mg	NCBI_Assembly:GCF_000346465.1	NW_006760212.1	-	12865267	12868928	3661	-	GO:0006351:transcription, DNA-templated;GO:0006355:regulation of transcription, DNA-templated	GO:0003677:DNA binding	GO:0005634:nucleus
Pp03_19842-3p	18777951	PRUPE_ppa008851mg	NCBI_Assembly:GCF_000346465.1	NW_006760212.1	+	14125120	14127419	2299	-	-	GO:0016787:hydrolase activity	-
Pp03_19842-3p	18778061	PRUPE_ppa000953mg	NCBI_Assembly:GCF_000346465.1	NW_006760212.1	+	2876588	2879451	2863	pper04626:Plant-pathogen interaction	-	GO:0043531:ADP binding	-
Pp03_19842-3p	18778079	PRUPE_ppa015406mg	NCBI_Assembly:GCF_000346465.1	NW_006760212.1	+	13130914	13135287	4373	-	-	GO:0008270:zinc ion binding	-
Pp03_19842-3p	18778122	PRUPE_ppa001411mg	NCBI_Assembly:GCF_000346465.1	NW_006760212.1	-	13235330	13239084	3754	-	-	-	-
Pp03_19842-3p	18778123	PRUPE_ppa025782m1g	NCBI_Assembly:GCF_000346465.1	NW_006760212.1	+	13231052	13234619	3567	pper04144:Endocytosis	-	GO:0005096:GTPase activator activity	-
Pp03_19842-3p	18778215	PRUPE_ppa011626mg	NCBI_Assembly:GCF_000346465.1	NW_006760220.1	-	16565410	16566274	864	-	-	-	-

Pp03_19842-3p	18778259	PRUPE_ppa001961mg	NCBI_Assembly:GCF_000346465.1	NW_006760220.1	+	12580095	12589549	9454	-	-	-	-
Pp03_19842-3p	18778347	PRUPE_ppa002345mg	NCBI_Assembly:GCF_000346465.1	NW_006760220.1	+	4241233	4246220	4987	-	GO:0010405: arabinogalactan protein metabolic process;GO:0018258:protein O-linked glycosylation via hydroxyproline	GO:0030246: carbohydrate binding;GO:0035250:UDP-galactosyltransferase activity	GO:0005794: Golgi apparatus;GO:0016021:integral component of membrane
Pp03_19842-3p	18778375	PRUPE_ppa027114mg	NCBI_Assembly:GCF_000346465.1	NW_006760220.1	+	5843875	5845275	1400	-	-	GO:0005385: zinc ion transmembrane transporter activity	GO:0016021: integral component of membrane
Pp03_19842-3p	18778416	PRUPE_ppa004823mg	NCBI_Assembly:GCF_000346465.1	NW_006760220.1	-	10108872	10113030	4158	-	GO:0055085: transmembrane transport	GO:0005509: calcium ion binding	GO:0016021: integral component of membrane
Pp03_19842-3p	18778478	PRUPE_ppa025681mg	NCBI_Assembly:GCF_000346465.1	NW_006760220.1	-	3950628	3953050	2422	-	-	-	-
Pp03_19842-3p	18778615	PRUPE_ppa003607mg	NCBI_Assembly:GCF_000346465.1	NW_006760220.1	-	5163287	5168196	4909	-	-	GO:0004674: protein serine/threonine kinase activity;GO:0005524:ATP binding	-

Pp03_19842-3p	18778627	PRUPE_ppa000550mg	NCBI_Assembly:GCF_000346465.1	NW_006760220.1	-	8359249	8363448	4199	-	GO:0002237:response to molecule of bacterial origin;GO:0007165:signal transduction;GO:0010103:stomatal complex morphogenesis;GO:0048443:stamen development	GO:0004672:protein kinase activity;GO:0005524:ATP binding	GO:0016021:integral component of membrane
Pp03_19842-3p	18778634	PRUPE_ppa022868mg	NCBI_Assembly:GCF_000346465.1	NW_006760220.1	-	10974197	10975795	1598	-	-	-	-
Pp03_19842-3p	18778652	PRUPE_ppa010081mg	NCBI_Assembly:GCF_000346465.1	NW_006760220.1	+	3727962	3728810	848	pper01100:Metabolic pathways;pper00196:Photosynthesis - antenna proteins	GO:0009765:photosynthesis, light harvesting;GO:0018298:protein-chromophore linkage	GO:0016168:chlorophyll binding;GO:0046872:metal ion binding	GO:0009507:chloroplast;GO:0009522:photosystem I;GO:0009523:photosystem II;GO:0016021:integral component of membrane
Pp03_19842-3p	18778659	PRUPE_ppa014235mg	NCBI_Assembly:GCF_000346465.1	NW_006760220.1	-	11510233	11510757	524	-	-	-	-
Pp03_19842-3p	18778662	PRUPE_ppa015874mg	NCBI_Assembly:GCF_000346465.1	NW_006760220.1	+	680859	682291	1432	-	-	-	-
Pp03_19842-3p	18778678	PRUPE_ppa011648mg	NCBI_Assembly:GCF_000346465.1	NW_006760220.1	-	234153	237287	3134	-	-	-	GO:0005773:vacuole;GO:0005886:plasma membrane
Pp03_19842-3p	18778752	PRUPE_ppa013893mg	NCBI_Assembly:GCF_000346465.1	NW_006760220.1	-	2481043	2484185	3142	-	-	-	-

Pp03_19842-3p	18778821	PRUPE_ppb014490mg	NCBI_Assembly:GCF_000346465.1	NW_006760220.1	+	1340088	1342853	2765	-	-	GO:0016772: transferase activity, transferring phosphorus-containing groups	-
Pp03_19842-3p	18778840	PRUPE_ppa007232mg	NCBI_Assembly:GCF_000346465.1	NW_006760220.1	+	4502602	4506118	3516	pper01100:Metabolic pathways;pper00195:Photosynthesis	-	GO:0016491: oxidoreductase activity	-
Pp03_19842-3p	18779089	PRUPE_ppa022792mg	NCBI_Assembly:GCF_000346465.1	NW_006760220.1	+	535814	536083	269	-	-	-	-
Pp03_19842-3p	18779506	PRUPE_ppa025131mg	NCBI_Assembly:GCF_000346465.1	NW_006760220.1	+	12050428	12050909	481	-	-	-	-

Pp03_19842-3p	18779574	PRUPE_ppa000402mg	NCBI_Assembly:GCF_000346465.1	NW_006760220.1	-	19789740	19803415	13675	-	GO:0007155:cell adhesion;GO:0010090:trichome morphogenesis;GO:0010228:vegetative to reproductive phase transition of meristem;GO:0016926:protein desumoylation;GO:0045010:actin nucleation;GO:0048765:root hair cell differentiation;GO:0050665:hydrogen peroxide biosynthetic process;GO:0071555:cell wall organization	-	-
Pp03_19842-3p	18779637	PRUPE_ppa016012mg	NCBI_Assembly:GCF_000346465.1	NW_006760220.1	-	964517	965863	1346	pper00040:Pentose and glucuronate interconversions	GO:0045490:pectin catabolic process	GO:0030570:pectate lyase activity;GO:0046872:metal ion binding	-

Pp03_19842-3p	18779680	PRUPE_ppa012245mg	NCBI_Assembly:GCF_000346465.1	NW_006760220.1	+	30102546	30108327	5781	-	GO:0000394:RNA splicing, via endonucleolytic cleavage and ligation;GO:0006661:phosphatidylinositol biosynthetic process;GO:0009086:methionine biosynthetic process;GO:0016192:vesicle-mediated transport	-	GO:0016021:integral component of membrane
Pp03_19842-3p	18779694	PRUPE_ppa004725mg	NCBI_Assembly:GCF_000346465.1	NW_006760220.1	+	6389487	6390980	1493	-	-	GO:0016758:transferase activity, transferring hexosyl groups	-
Pp03_19842-3p	18779819	PRUPE_ppb001416mg	NCBI_Assembly:GCF_000346465.1	NW_006760220.1	+	29745034	29748657	3623	-	-	-	-

Pp03_19842-3p	18779838	PRUPE_ppa000071mg	NCBI_Assembly:GCF_000346465.1	NW_006760220.1	+	25610527	25626706	16179	-	GO:0006075:(1->3)-beta-D-glucan biosynthetic process;GO:0009556:microsporogenesis;GO:0009827:plant-type cell wall modification;GO:0009846:pollen germination;GO:0009860:pollen tube growth;GO:0010584:pollen exine formation;GO:0080092:regulation of pollen tube growth	GO:0003843:1,3-beta-D-glucan synthase activity	GO:0000148:1,3-beta-D-glucan synthase complex
Pp03_19842-3p	18779916	PRUPE_ppa001615mg	NCBI_Assembly:GCF_000346465.1	NW_006760220.1	-	1461647	1465052	3405	-	GO:0048544:recognition of pollen	GO:0004674:protein serine/threonine kinase activity;GO:0005524:ATP binding	-
Pp03_19842-3p	18779931	PRUPE_ppa002948mg	NCBI_Assembly:GCF_000346465.1	NW_006760220.1	+	5581765	5585393	3628	-	-	-	-

Pp03_19842-3p	18780233	PRUPE_ppa010411mg	NCBI_Assembly:GCF_000346465.1	NW_006760220.1	+	6398845	6402220	3375	pper03050:Proteasome	GO:0006511:ubiquitin-dependent protein catabolic process	GO:0004298:threonine-type endopeptidase activity	GO:0005634:nucleus;GO:0005737:cytoplasm;GO:0019773:proteasome core complex, alpha-subunit complex
Pp03_19842-3p	18780255	PRUPE_ppa024298m2g	NCBI_Assembly:GCF_000346465.1	NW_006760220.1	+	3049591	3053063	3472	-	-	-	-
Pp03_19842-3p	18780294	PRUPE_ppa017479mg	NCBI_Assembly:GCF_000346465.1	NW_006760220.1	-	17442892	17446069	3177	-	-	GO:0004497:monooxygenase activity;GO:0005506:iron ion binding;GO:0016705:oxidoreductase activity, acting on paired donors, with incorporation or reduction of molecular oxygen;GO:0020037:heme binding	-
Pp03_19842-3p	18780387	PRUPE_ppa011078mg	NCBI_Assembly:GCF_000346465.1	NW_006760220.1	-	3514299	3515942	1643	-	-	-	-
Pp03_19842-3p	18780442	PRUPE_ppa014139mg	NCBI_Assembly:GCF_000346465.1	NW_006760220.1	-	2588371	2588691	320	-	GO:0045454:cell redox homeostasis	GO:0009055:electron carrier activity;GO:0015035:protein disulfide oxidoreductase activity	GO:0005623:cell

Pp03_19842-3p	18780468	PRUPE_ppa009230mg	NCBI_Assembly:GCF_000346465.1	NW_006760220.1	-	11168222	11169155	933	-	-	GO:0008168:methyltransferase activity	-
Pp03_19842-3p	18780480	PRUPE_ppa001834mg	NCBI_Assembly:GCF_000346465.1	NW_006760220.1	+	3349202	3357353	8151	-	-	GO:0004672:protein kinase activity;GO:0005524:ATP binding	-
Pp03_19842-3p	18780505	PRUPE_ppa015975mg	NCBI_Assembly:GCF_000346465.1	NW_006760220.1	+	3830890	3834599	3709	-	-	GO:0003968:RNA-directed RNA polymerase activity	-
Pp03_19842-3p	18780538	PRUPE_ppa025583mg	NCBI_Assembly:GCF_000346465.1	NW_006760220.1	+	1087887	1088543	656	pper04141:Protein processing in endoplasmic reticulum	GO:0009408:response to heat;GO:0009644:response to high light intensity;GO:0042542:response to hydrogen peroxide	-	-
Pp03_19842-3p	18780568	PRUPE_ppa006315mg	NCBI_Assembly:GCF_000346465.1	NW_006760220.1	+	5199428	5204798	5370	pper01100:Metabolic pathways;pper01110:Biosynthesis of secondary metabolites;pper00520:Amino sugar and nucleotide sugar metabolism	GO:0006012:galactose metabolic process	GO:0003978:UDP-glucose 4-epimerase activity;GO:0050662:coenzyme binding	-

Pp03_19842-3p	18780576	PRUPE_ppa008675mg	NCBI_Assembly:GCF_000346465.1	NW_006760220.1	+	3313373	3316798	3425	-	GO:0006355:regulation of transcription, DNA-templated;GO:0009414:response to water deprivation;GO:0009737:response to abscisic acid;GO:0042538:hyperosmotic salinity response	-	-	
Pp03_19842-3p	18780601	PRUPE_ppa013514mg	NCBI_Assembly:GCF_000346465.1	NW_006760220.1	-	13158336	13161036	2700	-	-	-	GO:0016020:membrane	
Pp03_19842-3p	18781530	PRUPE_ppa025063mg	NCBI_Assembly:GCF_000346465.1	NW_006760220.1	-	7818909	7825543	6634	-	-	-	-	
Pp03_19842-3p	18781640	PRUPE_ppa020057mg	NCBI_Assembly:GCF_000346465.1	NW_006760268.1	+	19270904	19272288	1384	-	-	GO:0004672:protein kinase activity;GO:0005524:ATP binding	-	
Pp03_19842-3p	18782862	PRUPE_ppa019597mg	NCBI_Assembly:GCF_000346465.1	NW_006760268.1	+	1367665	1372041	4376	-	GO:0015074:DNA integration	GO:0003676:nucleic acid binding	GO:0005634:nucleus	

Pp03_19842-3p	18782962	PRUPE_ppa009083mg	NCBI_Assembly:GCF_000346465.1	NW_006760268.1	+	6450391	6452867	2476	-	GO:0010264:myo-inositol hexakisphosphate biosynthetic process	GO:0015168:glycerol transmembrane transporter activity;GO:0015204:urea transmembrane transporter activity;GO:0046715:borate transmembrane transporter activity	GO:0005886:plasma membrane;GO:0016021:integral component of membrane
Pp03_19842-3p	18782981	PRUPE_ppa017224mg	NCBI_Assembly:GCF_000346465.1	NW_006760268.1	+	19412641	19413914	1273	-	GO:0032955:regulation of barrier septum assembly;GO:0051301:cell division	-	-
Pp03_19842-3p	18783019	PRUPE_ppa007567mg	NCBI_Assembly:GCF_000346465.1	NW_006760268.1	-	20287558	20290017	2459	-	-	GO:0022857:transmembrane transporter activity	GO:0016021:integral component of membrane
Pp03_19842-3p	18783025	PRUPE_ppa025208mg	NCBI_Assembly:GCF_000346465.1	NW_006760268.1	+	16909026	16913170	4144	-	-	-	-
Pp03_19842-3p	18783100	PRUPE_ppa023073mg	NCBI_Assembly:GCF_000346465.1	NW_006760268.1	+	8559961	8560469	508	-	-	GO:0004672:protein kinase activity;GO:0005524:ATP binding	-
Pp03_19842-3p	18783152	PRUPE_ppa020987mg	NCBI_Assembly:GCF_000346465.1	NW_006760268.1	+	19821038	19823654	2616	-	-	-	-
Pp03_19842-3p	18783175	PRUPE_ppa010059mg	NCBI_Assembly:GCF_000346465.1	NW_006760268.1	+	1413967	1414998	1031	-	-	GO:0008270:zinc ion binding	-

Pp03_19842-3p	18783176	PRUPE_ppa002575mg	NCBI_Assembly:GCF_000346465.1	NW_006760268.1	+	21642024	21646827	4803	-	-	-	GO:0005737: cytoplasm
Pp03_19842-3p	18783307	PRUPE_ppa005040mg	NCBI_Assembly:GCF_000346465.1	NW_006760268.1	+	7320061	7322318	2257	-	-	GO:0004190: aspartic-type endopeptidase activity	GO:0048046: apoplast
Pp03_19842-3p	18783436	PRUPE_ppa002558mg	NCBI_Assembly:GCF_000346465.1	NW_006760268.1	-	21824770	21828546	3776	-	-	GO:0003676: nucleic acid binding;GO:0008270:zinc ion binding	-
Pp03_19842-3p	18783452	PRUPE_ppa009567mg	NCBI_Assembly:GCF_000346465.1	NW_006760268.1	-	5624837	5627233	2396	-	-	-	-
Pp03_19842-3p	18783605	PRUPE_ppa016199mg	NCBI_Assembly:GCF_000346465.1	NW_006760268.1	+	2380072	2380909	837	-	-	-	GO:0016021: integral component of membrane
Pp03_19842-3p	18783635	PRUPE_ppa018231mg	NCBI_Assembly:GCF_000346465.1	NW_006760268.1	+	4885037	4886194	1157	-	-	GO:0003682: chromatin binding	-
Pp03_19842-3p	18783665	PRUPE_ppa019057mg	NCBI_Assembly:GCF_000346465.1	NW_006760268.1	-	18172325	18174355	2030	-	GO:0016998: cell wall macromolecule catabolic process	GO:0052861: glucan endo-1,3-beta-glucanase activity, C-3 substituted reducing group;GO:0052862:glucan endo-1,4-beta-glucanase activity, C-3 substituted reducing group	-

Pp03_19842-3p	18783760	PRUPE_ppa009917mg	NCBI_Assembly:GCF_000346465.1	NW_006760268.1	+	10102928	10108264	5336	pper01100:Metabolic pathways;pper01110:Biosynthesis of secondary metabolites;pper00100:Steroid biosynthesis	GO:0006633:fatty acid biosynthetic process	GO:0005506:iron ion binding;GO:0016491:oxidoreductase activity	GO:0016021:integral component of membrane
Pp03_19842-3p	18783787	PRUPE_ppa008193mg	NCBI_Assembly:GCF_000346465.1	NW_006760268.1	-	1671128	1673802	2674	pper03010:Ribosome	GO:0000028:ribosomal small subunit assembly;GO:0006412:translation	GO:0003735:structural constituent of ribosome	GO:0022627:cytosolic small ribosomal subunit
Pp03_19842-3p	18783796	PRUPE_ppa026283mg	NCBI_Assembly:GCF_000346465.1	NW_006760268.1	+	1951010	1951378	368	-	-	-	-
Pp03_19842-3p	18783884	PRUPE_ppa023037mg	NCBI_Assembly:GCF_000346465.1	NW_006760268.1	-	17691970	17692362	392	-	-	-	-
Pp03_19842-3p	18783965	PRUPE_ppa004876mg	NCBI_Assembly:GCF_000346465.1	NW_006760268.1	+	19914830	19917316	2486	-	GO:0015979:photosynthesis;GO:0045333:cellular respiration	GO:0008270:zinc ion binding	-
Pp03_19842-3p	18784039	PRUPE_ppa015414mg	NCBI_Assembly:GCF_000346465.1	NW_006760268.1	+	13167236	13168573	1337	-	-	GO:0005215:transporter activity	GO:0016020:membrane
Pp03_19842-3p	18784057	PRUPE_ppa011418mg	NCBI_Assembly:GCF_000346465.1	NW_006760268.1	+	7293624	7296420	2796	-	-	-	-
Pp03_19842-3p	18784187	PRUPE_ppa011673mg	NCBI_Assembly:GCF_000346465.1	NW_006760268.1	+	2754521	2755994	1473	-	GO:0016192:vesicle-mediated transport	-	GO:0005783:endoplasmic reticulum
Pp03_19842-3p	18784244	PRUPE_ppa026501mg	NCBI_Assembly:GCF_000346465.1	NW_006760268.1	+	7595447	7597264	1817	-	-	-	-
Pp03_19842-3p	18784283	PRUPE_ppa022074mg	NCBI_Assembly:GCF_000346465.1	NW_006760268.1	-	12452404	12455202	2798	-	-	-	-

Pp03_19842-3p	18784568	PRUPE_ppa011162mg	NCBI_Assembly:GCF_000346465.1	NW_006760324.1	-	13752300	13753142	842	-	-	-	-
Pp03_19842-3p	18784606	PRUPE_ppa019864mg	NCBI_Assembly:GCF_000346465.1	NW_006760324.1	-	21695188	21696612	1424	-	GO:0006633: fatty acid biosynthetic process	GO:0016747: transferase activity, transferring acyl groups other than amino-acyl groups	GO:0016020: membrane
Pp03_19842-3p	18784608	PRUPE_ppa010351mg	NCBI_Assembly:GCF_000346465.1	NW_006760324.1	+	26051329	26053080	1751	-	GO:0000023: maltose metabolic process;GO:0006098:pentose-phosphate shunt;GO:0019252:starch biosynthetic process;GO:0043085:positive regulation of catalytic activity	-	GO:0009535: chloroplast thylakoid membrane;GO:0009941:chloroplast envelope

Pp03_19842-3p	18784678	PRUPE_ppa017352mg	NCBI_Assembly:GCF_000346465.1	NW_006760324.1	+	21764643	21766886	2243	pper01100:Metabolic pathways;pper01110:Biosynthesis of secondary metabolites;pper00511:Other glycan degradation;pper00520:Amino sugar and nucleotide sugar metabolism;pper00531:Glycosaminoglycan degradation;pper00603:Glycosphingolipid biosynthesis - globo series;pper00604:Glycosphingolipid biosynthesis - ganglio series	GO:0005975:carbohydrate metabolic process	GO:0004563:beta-N-acetylhexosaminidase activity	-
Pp03_19842-3p	18784698	PRUPE_ppa008157m2g	NCBI_Assembly:GCF_000346465.1	NW_006760324.1	+	16927225	16927644	419	-	-	GO:0004672:protein kinase activity;GO:0005524:ATP binding	-

Pp03_19842-3p	18784742	PRUPE_ppa008453mg	NCBI_Assembly:GCF_000346465.1	NW_006760324.1	+	13989674	13992935	3261	pper01100:Metabolic pathways;pper00230:Purine metabolism	GO:0001560:regulation of cell growth by extracellular stimulus;GO:0006144:purine nucleobase metabolic process;GO:0006499:N-terminal protein myristoylation;GO:0009627:systemic acquired resistance;GO:0009742:brassinosteroid mediated signaling pathway;GO:0019428:allantoin biosynthetic process;GO:0031347:regulation of defense response;GO:0051289:protein homotetramerization	GO:0033971:hydroxyisourate hydrolase activity;GO:0051997:2-oxo-4-hydroxy-4-carboxy-5-ureidoimidazoline decarboxylase activity	GO:0005777:peroxisome;GO:0005829:cytosol;GO:0031234:extrinsic component of cytoplasmic side of plasma membrane

Pp03_19842-3p	18784764	PRUPE_ppa001928mg	NCBI_Assembly:GCF_000346465.1	NW_006760324.1	+	25103928	25108655	4727	pper03015:mRNA surveillance pathway	GO:0000278:mitotic cell cycle;GO:0006306:DNA methylation;GO:0006342:chromatin silencing;GO:0006378:mRNA polyadenylation;GO:0006379:mRNA cleavage;GO:0007267:cell-cell signaling;GO:0009220:pyrimidine ribonucleotide biosynthetic process;GO:0009616:virus induced gene silencing;GO:0009640:photomorphogenesis;GO:0010267:production of ta-siRNAs involved in RNA interference;GO:0010388:cullin deneddylatio	-	GO:0005847:mRNA cleavage and polyadenylation specificity factor complex;GO:0009506:plasmodesma

Pp03_19842-3p	18784902	PRUPE_ppa010401mg	NCBI_Assembly:GCF_000346465.1	NW_006760324.1	+	9924457	9927369	2912	pper03013:RNA transport;pper03015:mRNA surveillance pathway;pper03018:RNA degradation	GO:0009773:photosynthetic electron transport in photosystem I;GO:0019288:isopentenyl diphosphate biosynthetic process, methylerythritol 4-phosphate pathway;GO:0045036:protein targeting to chloroplast	GO:0000166:nucleotide binding;GO:0003676:nucleic acid binding	GO:0009535:chloroplast thylakoid membrane;GO:0009570:chloroplast stroma;GO:0009941:chloroplast envelope
Pp03_19842-3p	18784915	PRUPE_ppa014173mg	NCBI_Assembly:GCF_000346465.1	NW_006760324.1	+	21701305	21702412	1107	-	-	-	-
Pp03_19842-3p	18785020	PRUPE_ppa011969mg	NCBI_Assembly:GCF_000346465.1	NW_006760324.1	-	13654479	13656705	2226	-	-	-	-
Pp03_19842-3p	18785027	PRUPE_ppa013788mg	NCBI_Assembly:GCF_000346465.1	NW_006760324.1	+	14870650	14871581	931	-	-	-	-
Pp03_19842-3p	18785047	PRUPE_ppa002366mg	NCBI_Assembly:GCF_000346465.1	NW_006760324.1	+	16539611	16542751	3140	-	GO:0010075:regulation of meristem growth	GO:0004672:protein kinase activity;GO:0005524:ATP binding	GO:0016021:integral component of membrane
Pp03_19842-3p	18785110	PRUPE_ppa025152mg	NCBI_Assembly:GCF_000346465.1	NW_006760324.1	-	16635551	16635778	227	-	-	-	-
Pp03_19842-3p	18785112	PRUPE_ppa003039mg	NCBI_Assembly:GCF_000346465.1	NW_006760324.1	-	26502156	26505894	3738	-	-	GO:0005524:ATP binding	-

Pp03_19842-3p	18785134	PRUPE_ppa021305mg	NCBI_Assembly:GCF_000346465.1	NW_006760324.1	+	13687709	13689728	2019	-	GO:0000956:nuclear-transcribed mRNA catabolic process;GO:0006346:methylation-dependent chromatin silencing;GO:0007346:regulation of mitotic cell cycle;GO:0009825:multidimensional cell growth;GO:0009910:negative regulation of flower development;GO:0010016:shoot system morphogenesis;GO:0010048:vernalization response;GO:0016246:RNA interference;GO:0031048:chromatin silencing by small	GO:0003677:DNA binding;GO:0003935:GTP cyclohydrolase II activity	GO:0000791:euchromatin;GO:0005720:nuclear heterochromatin;GO:0009507:chloroplast
Pp03_19842-3p	18785152	PRUPE_ppa023600mg	NCBI_Assembly:GCF_000346465.1	NW_006760324.1	-	9134862	9136872	2010	-	-	-	-
Pp03_19842-3p	18785214	PRUPE_ppa013450mg	NCBI_Assembly:GCF_000346465.1	NW_006760324.1	+	9686283	9687856	1573	pper03010:Ribosome	GO:0006412:translation	GO:0003735:structural constituent of ribosome	GO:0005840:ribosome

Pp03_19842-3p	18785236	PRUPE_ppa010525mg	NCBI_Assembly:GCF_000346465.1	NW_006760324.1	+	22303363	22306151	2788	-	GO:0006457:protein folding	GO:0003755:peptidyl-prolyl cis-trans isomerase activity	-
Pp03_19842-3p	18785361	PRUPE_ppa010092mg	NCBI_Assembly:GCF_000346465.1	NW_006760324.1	-	23952501	23954459	1958	-	-	GO:0003677:DNA binding;GO:0003682:chromatin binding;GO:0003700:sequence-specific DNA binding transcription factor activity;GO:0008270:zinc ion binding	-
Pp03_19842-3p	18785546	PRUPE_ppa023233mg	NCBI_Assembly:GCF_000346465.1	NW_006760324.1	+	4006579	4006797	218	-	-	-	-
Pp03_19842-3p	18785557	PRUPE_ppa000101m2g	NCBI_Assembly:GCF_000346465.1	NW_006760324.1	-	23031314	23040552	9238	pper04120:Ubiquitin mediated proteolysis	-	-	GO:0005680:anaphase-promoting complex
Pp03_19842-3p	18785589	PRUPE_ppa013270mg	NCBI_Assembly:GCF_000346465.1	NW_006760324.1	+	26483165	26484109	944	-	-	-	-
Pp03_19842-3p	18785639	PRUPE_ppa011710mg	NCBI_Assembly:GCF_000346465.1	NW_006760324.1	-	14233314	14234493	1179	-	-	GO:0016787:hydrolase activity	-
Pp03_19842-3p	18785655	PRUPE_ppa016978mg	NCBI_Assembly:GCF_000346465.1	NW_006760324.1	+	17158918	17162528	3610	-	-	GO:0008324:cation transmembrane transporter activity	GO:0016021:integral component of membrane
Pp03_19842-3p	18785810	PRUPE_ppa023497mg	NCBI_Assembly:GCF_000346465.1	NW_006760324.1	+	18702013	18705700	3687	-	-	GO:0008270:zinc ion binding	GO:0005886:plasma membrane

Pp03_19842-3p	18785870	PRUPE_ppa001671mg	NCBI_Assembly:GCF_000346465.1	NW_006760324.1	-	6830498	6836546	6048	-	-	GO:0004672:protein kinase activity;GO:0005524:ATP binding	-
Pp03_19842-3p	18786065	PRUPE_ppa007819mg	NCBI_Assembly:GCF_000346465.1	NW_006760324.1	+	24210052	24213172	3120	-	GO:0006376:mRNA splice site selection	GO:0003729:mRNA binding	GO:0005685:U1 snRNP
Pp03_19842-3p	18786081	PRUPE_ppa011367mg	NCBI_Assembly:GCF_000346465.1	NW_006760324.1	-	24997064	24999001	1937	-	-	-	-
Pp03_19842-3p	18786165	PRUPE_ppb021167mg	NCBI_Assembly:GCF_000346465.1	NW_006760324.1	-	14662266	14662502	236	-	-	-	-
Pp03_19842-3p	18786169	PRUPE_ppa008326m2g	NCBI_Assembly:GCF_000346465.1	NW_006760324.1	+	19046071	19046418	347	-	-	GO:0016758:transferase activity, transferring hexosyl groups	-
Pp03_19842-3p	18786176	PRUPE_ppa025968mg	NCBI_Assembly:GCF_000346465.1	NW_006760324.1	+	332336	334660	2324	-	-	GO:0003676:nucleic acid binding;GO:0008270:zinc ion binding	-
Pp03_19842-3p	18786187	PRUPE_ppa016841mg	NCBI_Assembly:GCF_000346465.1	NW_006760324.1	-	14738516	14738943	427	-	-	-	-
Pp03_19842-3p	18786193	PRUPE_ppa014160mg	NCBI_Assembly:GCF_000346465.1	NW_006760324.1	+	2472256	2472877	621	-	-	-	-
Pp03_19842-3p	18786330	PRUPE_ppa005993mg	NCBI_Assembly:GCF_000346465.1	NW_006760324.1	+	24820981	24825176	4195	pper03040:Spliceosome	-	GO:0000166:nucleotide binding;GO:0003676:nucleic acid binding	-
Pp03_19842-3p	18786334	PRUPE_ppa008985mg	NCBI_Assembly:GCF_000346465.1	NW_006760324.1	+	4383605	4385810	2205	-	-	-	-
Pp03_19842-3p	18786417	PRUPE_ppb014191mg	NCBI_Assembly:GCF_000346465.1	NW_006760324.1	-	10133688	10134241	553	-	-	-	-

Pp03_19842-3p	18786459	PRUPE_ppb017095mg	NCBI_Assembly:GCF_000346465.1	NW_006760324.1	-	7585123	7589486	4363	-	-	GO:0003676:nucleic acid binding;GO:0008270:zinc ion binding	-
Pp03_19842-3p	18786610	PRUPE_ppa024290mg	NCBI_Assembly:GCF_000346465.1	NW_006760324.1	-	25029599	25031236	1637	-	GO:0030001:metal ion transport	GO:0046872:metal ion binding	-
Pp03_19842-3p	18786613	PRUPE_ppa018908mg	NCBI_Assembly:GCF_000346465.1	NW_006760324.1	-	12594859	12597087	2228	-	-	-	-
Pp03_19842-3p	18786702	PRUPE_ppa006808mg	NCBI_Assembly:GCF_000346465.1	NW_006760324.1	-	26163258	26166569	3311	-	-	GO:0000166:nucleotide binding;GO:0003676:nucleic acid binding	-
Pp03_19842-3p	18786737	PRUPE_ppa021192mg	NCBI_Assembly:GCF_000346465.1	NW_006760324.1	+	13027862	13029019	1157	-	-	-	-
Pp03_19842-3p	18786883	PRUPE_ppa005831mg	NCBI_Assembly:GCF_000346465.1	NW_006760324.1	+	5469641	5472791	3150	-	GO:0032957:inositol trisphosphate metabolic process;GO:0046855:inositol phosphate dephosphorylation;GO:0046856:phosphatidylinositol dephosphorylation	-	-
Pp03_19842-3p	18786888	PRUPE_ppa000286mg	NCBI_Assembly:GCF_000346465.1	NW_006760324.1	+	26397562	26405536	7974	-	-	GO:0004672:protein kinase activity;GO:0005524:ATP binding	-

Pp03_19842-3p	18786916	PRUPE_ppa025397mg	NCBI_Assembly:GCF_000346465.1	NW_006760324.1	-	23116887	23120230	3343	-	-	GO:0003677:DNA binding;GO:0003682:chromatin binding;GO:0003700:sequence-specific DNA binding transcription factor activity	-
Pp03_19842-3p	18786919	PRUPE_ppa001323mg	NCBI_Assembly:GCF_000346465.1	NW_006760324.1	-	16762797	16770148	7351	-	GO:0007018:microtubule-based movement;GO:0048364:root development	GO:0003777:microtubule motor activity;GO:0005524:ATP binding	GO:0005871:kinesin complex;GO:0005874:microtubule
Pp03_19842-3p	18786960	PRUPE_ppa010566mg	NCBI_Assembly:GCF_000346465.1	NW_006760324.1	-	20588843	20590531	1688	-	GO:0000398:mRNA splicing, via spliceosome;GO:0032502:developmental process;GO:0051302:regulation of cell division	GO:0000166:nucleotide binding;GO:0003676:nucleic acid binding;GO:0008270:zinc ion binding	GO:0005634:nucleus
Pp03_19842-3p	18786974	PRUPE_ppa010870mg	NCBI_Assembly:GCF_000346465.1	NW_006760324.1	-	25557080	25559323	2243	-	GO:0006623:protein targeting to vacuole;GO:0010200:response to chitin;GO:0048193:Golgi vesicle transport	GO:0003774:motor activity	GO:0005886:plasma membrane;GO:0016459:myosin complex
Pp03_19842-3p	18786985	PRUPE_ppa025257mg	NCBI_Assembly:GCF_000346465.1	NW_006760324.1	-	14861565	14862817	1252	-	-	-	-

Pp03_19842-3p	18787038	PRUPE_ppa002072mg	NCBI_Assembly:GCF_000346465.1	NW_006760324.1	-		25978206	25981397	3191	-	-	-	-
Pp03_19842-3p	18787132	PRUPE_ppa024567mg	NCBI_Assembly:GCF_000346465.1	NW_006760324.1	+		2115851	2123681	7830	-	GO:0009308:amine metabolic process	GO:0005507:copper ion binding;GO:0048038:quinone binding	-
Pp03_19842-3p	18787196	PRUPE_ppa009051mg	NCBI_Assembly:GCF_000346465.1	NW_006760324.1	-		17047525	17050233	2708	-	GO:0000373:Group II intron splicing;GO:0000956:nuclear-transcribed mRNA catabolic process;GO:0009793:embryo development ending in seed dormancy;GO:0010048:vernalization response;GO:0048573:photoperiodism, flowering;GO:0097031:mitochondrial respiratory chain complex I biogenesis;GO:0097034:mitochondrial respiratory chain complex IV biogenesis	GO:0003723:RNA binding	-

Pp03_19842-3p	18787243	PRUPE_ppa014727mg	NCBI_Assembly:GCF_000346465.1	NW_006760324.1	-	2693936	2695131	1195	-	GO:0006355:regulation of transcription, DNA-templated	GO:0003690:double-stranded DNA binding	GO:0005739:mitochondrion
Pp03_19842-3p	18787292	PRUPE_ppa019396mg	NCBI_Assembly:GCF_000346465.1	NW_006760324.1	+	16961517	16962788	1271	-	-	GO:0016616:oxidoreductase activity, acting on the CH-OH group of donors, NAD or NADP as acceptor;GO:0051287:NAD binding	-
Pp03_19842-3p	18788499	PRUPE_ppa002408mg	NCBI_Assembly:GCF_000346465.1	NW_006760385.1	-	9496961	9500728	3767	pper04075:Plant hormone signal transduction	GO:0000160:phosphorelay signal transduction system	GO:0003677:DNA binding;GO:0003682:chromatin binding	GO:0005622:intracellular
Pp03_19842-3p	18788553	PRUPE_ppa005965mg	NCBI_Assembly:GCF_000346465.1	NW_006760385.1	+	37973077	37977545	4468	-	-	-	-
Pp03_19842-3p	18788563	PRUPE_ppa019750mg	NCBI_Assembly:GCF_000346465.1	NW_006760385.1	-	14304135	14307619	3484	-	-	-	-
Pp03_19842-3p	18788586	PRUPE_ppa023308mg	NCBI_Assembly:GCF_000346465.1	NW_006760385.1	-	21300206	21302410	2204	-	-	GO:0005215:transporter activity	GO:0016020:membrane
Pp03_19842-3p	18788608	PRUPE_ppa016186mg	NCBI_Assembly:GCF_000346465.1	NW_006760385.1	+	32288926	32292516	3590	-	-	GO:0003676:nucleic acid binding;GO:0008270:zinc ion binding	-
Pp03_19842-3p	18788609	PRUPE_ppa010032mg	NCBI_Assembly:GCF_000346465.1	NW_006760385.1	-	22653728	22655127	1399	-	-	-	-
Pp03_19842-3p	18788613	PRUPE_ppa025111mg	NCBI_Assembly:GCF_000346465.1	NW_006760385.1	-	30042216	30043069	853	-	-	GO:0004185:serine-type carboxypeptidase activity	-

Pp03_19842-3p	18788654	PRUPE_ppa004410mg	NCBI_Assembly:GCF_000346465.1	NW_006760385.1	+	10045186	10046893	1707	-	-	GO:0015238:drug transmembrane transporter activity;GO:0015297:antiporter activity	GO:0016021:integral component of membrane
Pp03_19842-3p	18788875	PRUPE_ppa002814mg	NCBI_Assembly:GCF_000346465.1	NW_006760385.1	-	24440241	24442715	2474	pper03015:mRNA surveillance pathway	-	GO:0000166:nucleotide binding;GO:0003676:nucleic acid binding	-
Pp03_19842-3p	18788925	PRUPE_ppa003665mg	NCBI_Assembly:GCF_000346465.1	NW_006760385.1	-	27697689	27700123	2434	-	GO:0009220:pyrimidine ribonucleotide biosynthetic process	-	-
Pp03_19842-3p	18789000	PRUPE_ppa009550mg	NCBI_Assembly:GCF_000346465.1	NW_006760385.1	+	29346818	29348548	1730	-	GO:0006308:DNA catabolic process	GO:0003676:nucleic acid binding;GO:0004519:endonuclease activity	-
Pp03_19842-3p	18789023	PRUPE_ppa001231mg	NCBI_Assembly:GCF_000346465.1	NW_006760385.1	+	25693253	25703178	9925	-	GO:0006886:intracellular protein transport;GO:0016192:vesicle-mediated transport	GO:0008565:protein transporter activity	GO:0005794:Golgi apparatus;GO:0030131:clathrin adaptor complex

Pp03_19842-3p	18789091	PRUPE_ppa010830mg	NCBI_Assembly:GCF_000346465.1	NW_006760385.1	-	5019913	5021002	1089	-	GO:0006457:protein folding;GO:0009408:response to heat;GO:0009644:response to high light intensity;GO:0042542:response to hydrogen peroxide	-	-
Pp03_19842-3p	18789108	PRUPE_ppa021067mg	NCBI_Assembly:GCF_000346465.1	NW_006760385.1	+	27284302	27285822	1520	-	GO:0055085:transmembrane transport	-	GO:0016021:integral component of membrane
Pp03_19842-3p	18789116	PRUPE_ppa008777mg	NCBI_Assembly:GCF_000346465.1	NW_006760385.1	-	29194751	29196273	1522	-	-	GO:0003824:catalytic activity;GO:0050662:coenzyme binding	-
Pp03_19842-3p	18789126	PRUPE_ppa000749mg	NCBI_Assembly:GCF_000346465.1	NW_006760385.1	-	31047136	31056373	9237	pper04141:Protein processing in endoplasmic reticulum	GO:0006886:intracellular protein transport;GO:0006888:ER to Golgi vesicle-mediated transport	GO:0008270:zinc ion binding	GO:0030127:COPII vesicle coat
Pp03_19842-3p	18789150	PRUPE_ppa001454mg	NCBI_Assembly:GCF_000346465.1	NW_006760385.1	+	45452225	45460523	8298	-	-	-	-
Pp03_19842-3p	18789151	PRUPE_ppa011965mg	NCBI_Assembly:GCF_000346465.1	NW_006760385.1	-	31027464	31028268	804	-	GO:0010584:pollen exine formation;GO:0019290:siderophore biosynthetic process	GO:0008667:2,3-dihydro-2,3-dihydroxybenzoate dehydrogenase activity	-

Pp03_19842-3p	18789166	PRUPE_ppa020558mg	NCBI_Assembly:GCF_000346465.1	NW_006760385.1	-	33198740	33200325	1585	-	-	-	-
Pp03_19842-3p	18789630	PRUPE_ppa007313mg	NCBI_Assembly:GCF_000346465.1	NW_006760385.1	+	33503229	33504351	1122	-	-	GO:0016757: transferase activity, transferring glycosyl groups	-
Pp03_19842-3p	18789924	PRUPE_ppa006313mg	NCBI_Assembly:GCF_000346465.1	NW_006760385.1	+	10314881	10316802	1921	-	-	GO:0003824: catalytic activity	-
Pp03_19842-3p	18789946	PRUPE_ppa020172mg	NCBI_Assembly:GCF_000346465.1	NW_006760385.1	+	43042853	43049167	6314	-	GO:0006952: defense response;GO:0009607:response to biotic stimulus	-	GO:0016021: integral component of membrane
Pp03_19842-3p	18790020	PRUPE_ppa012209mg	NCBI_Assembly:GCF_000346465.1	NW_006760385.1	-	39544645	39545750	1105	-	-	-	-
Pp03_19842-3p	18790032	PRUPE_ppa013335mg	NCBI_Assembly:GCF_000346465.1	NW_006760385.1	+	32206554	32207449	895	-	-	-	-
Pp03_19842-3p	18790067	PRUPE_ppa000420mg	NCBI_Assembly:GCF_000346465.1	NW_006760385.1	-	28661391	28666431	5040	-	-	GO:0000287: magnesium ion binding;GO:0004012:phospholipid-translocating ATPase activity;GO:0005524:ATP binding	GO:0016021: integral component of membrane
Pp03_19842-3p	18790219	PRUPE_ppa012457mg	NCBI_Assembly:GCF_000346465.1	NW_006760385.1	-	38155414	38157297	1883	-	-	-	-

Pp03_19842-3p	18790291	PRUPE_ppa024900mg	NCBI_Assembly:GCF_000346465.1	NW_006760385.1	-	38001148	38003311	2163	-	-	GO:0016706:oxidoreductase activity, acting on paired donors, with incorporation or reduction of molecular oxygen, 2-oxoglutarate as one donor, and incorporation of one atom each of oxygen into both donors;GO:0046872:metal ion binding	-
Pp03_19842-3p	18790304	PRUPE_ppa022845mg	NCBI_Assembly:GCF_000346465.1	NW_006760385.1	+	31122118	31122494	376	-	GO:0006952:defense response	-	-
Pp03_19842-3p	18790307	PRUPE_ppa025279mg	NCBI_Assembly:GCF_000346465.1	NW_006760385.1	-	5122562	5123779	1217	-	-	-	-
Pp03_19842-3p	18790341	PRUPE_ppa006995mg	NCBI_Assembly:GCF_000346465.1	NW_006760385.1	+	14208855	14211238	2383	pper01100:Metabolic pathways;pper01110:Biosynthesis of secondary metabolites;pper00906:Carotenoid biosynthesis	GO:0016120:carotene biosynthetic process	GO:0090471:9,15,9'-tri-cis-zeta-carotene isomerase activity	-
Pp03_19842-3p	18790438	PRUPE_ppa012564mg	NCBI_Assembly:GCF_000346465.1	NW_006760385.1	-	21454177	21454804	627	pper03010:Ribosome	GO:0006412:translation	GO:0003735:structural constituent of ribosome	GO:0005840:ribosome

Pp03_19842-3p	18790598	PRUPE_ppa017438mg	NCBI_Assembly:GCF_000346465.1	NW_006760385.1	+	32013752	32016019	2267	-	-	-	-
Pp03_19842-3p	18790612	PRUPE_ppa012573mg	NCBI_Assembly:GCF_000346465.1	NW_006760385.1	+	9606195	9606977	782	-	GO:0006952:defense response;GO:0009607:response to biotic stimulus	-	-
Pp03_19842-3p	18790681	PRUPE_ppa005886mg	NCBI_Assembly:GCF_000346465.1	NW_006760385.1	-	23833349	23835333	1984	-	-	-	GO:0016021:integral component of membrane
Pp03_19842-3p	18790769	PRUPE_ppa009759mg	NCBI_Assembly:GCF_000346465.1	NW_006760385.1	+	9611402	9612346	944	-	GO:0030001:metal ion transport	GO:0046872:metal ion binding	-
Pp03_19842-3p	18790816	PRUPE_ppa026072mg	NCBI_Assembly:GCF_000346465.1	NW_006760385.1	+	23305090	23306828	1738	-	-	-	-
Pp03_19842-3p	18790828	PRUPE_ppa011037mg	NCBI_Assembly:GCF_000346465.1	NW_006760385.1	+	27146897	27147786	889	-	-	GO:0008270:zinc ion binding	-
Pp03_19842-3p	18790837	PRUPE_ppa017595mg	NCBI_Assembly:GCF_000346465.1	NW_006760385.1	-	18801106	18804751	3645	-	-	GO:0008234:cysteine-type peptidase activity	-
Pp03_19842-3p	18790863	PRUPE_ppa002677mg	NCBI_Assembly:GCF_000346465.1	NW_006760385.1	+	249809	256506	6697	-	-	-	-
Pp03_19842-3p	18790967	PRUPE_ppa003215mg	NCBI_Assembly:GCF_000346465.1	NW_006760385.1	+	43215593	43217371	1778	-	-	-	-
Pp03_19842-3p	18790970	PRUPE_ppb016405mg	NCBI_Assembly:GCF_000346465.1	NW_006760385.1	+	39615185	39616564	1379	-	-	-	-
Pp03_19842-3p	18790994	PRUPE_ppb018331mg	NCBI_Assembly:GCF_000346465.1	NW_006760385.1	+	38073191	38073640	449	-	-	-	-

Pp03_19842-3p	18791101	PRUPE_ppa000475mg	NCBI_Assembly:GCF_000346465.1	NW_006760385.1	+	3514047	3527066	13019	-	GO:0000278:mitotic cell cycle;GO:0000724:double-strand break repair via homologous recombination;GO:0006261:DNA-dependent DNA replication;GO:0006275:regulation of DNA replication;GO:0006298:mismatch repair;GO:0006306:DNA methylation;GO:0006342:chromatin silencing;GO:0009408:response to heat;GO:0009555:pollen development;GO:0016444:somatic cell DNA recombination;GO:0016572:histone phosphorylation;GO:00310[illegible]	GO:0005524:ATP binding;GO:0030983:mismatched DNA binding	GO:0005739:mitochondrion
Pp03_19842-3p	18791185	PRUPE_ppa015712mg	NCBI_Assembly:GCF_000346465.1	NW_006760385.1	+	23631293	23632681	1388	-	-	GO:0003676:nucleic acid binding;GO:0008270:zinc ion binding	-
Pp03_19842-3p	18791226	PRUPE_ppa013095mg	NCBI_Assembly:GCF_000346465.1	NW_006760385.1	-	15750004	15750867	863	-	-	-	-

Pp03_19842-3p	18791423	PRUPE_ppa000442mg	NCBI_Assembly:GCF_000346465.1	NW_006760385.1	+		16007987	16019372	11385	pper00970:Aminoacyl-tRNA biosynthesis	GO:0006094:gluconeogenesis;GO:0006096:glycolytic process;GO:0006428:isoleucyl-tRNA aminoacylation;GO:0007010:cytoskeleton organization;GO:0009651:response to salt stress;GO:0010498:proteasomal protein catabolic process	GO:0002161:aminoacyl-tRNA editing activity;GO:0004822:isoleucine-tRNA ligase activity;GO:0005524:ATP binding	GO:0005737:cytoplasm;GO:0005829:cytosol
Pp03_19842-3p	18791439	PRUPE_ppa004923mg	NCBI_Assembly:GCF_000346465.1	NW_006760385.1	+		42572708	42580610	7902	pper03013:RNA transport;pper03015:mRNA surveillance pathway	-	GO:0000166:nucleotide binding	-
Pp03_19842-3p	18791521	PRUPE_ppa005440mg	NCBI_Assembly:GCF_000346465.1	NW_006760385.1	-		39179282	39183920	4638	-	-	-	-
Pp03_19842-3p	18791677	PRUPE_ppa002388mg	NCBI_Assembly:GCF_000346465.1	NW_006760385.1	+		37371713	37374804	3091	-	GO:0006396:RNA processing	GO:0003723:RNA binding	GO:0048046:apoplast

Pp03_19842-3p	18791704	PRUPE_ppa011586mg	NCBI_Assembly:GCF_000346465.1	NW_006760385.1	+	4281164	4285639	4475	pper01100:Metabolic pathways;pper00230:Purine metabolism;pper00240:Pyrimidine metabolism	GO:0009117:nucleotide metabolic process;GO:0009204:deoxyribonucleoside triphosphate catabolic process	GO:0000166:nucleotide binding;GO:0046872:metal ion binding;GO:0047429:nucleoside-triphosphate diphosphatase activity	GO:0005737:cytoplasm
Pp03_19842-3p	18791722	PRUPE_ppa008102mg	NCBI_Assembly:GCF_000346465.1	NW_006760385.1	-	40779614	40782319	2705	-	-	-	-
Pp03_19842-3p	18791749	PRUPE_ppa022126mg	NCBI_Assembly:GCF_000346465.1	NW_006760385.1	+	32438839	32442705	3866	-	GO:0007018:microtubule-based movement	GO:0003777:microtubule motor activity;GO:0005524:ATP binding	GO:0005871:kinesin complex;GO:0005874:microtubule
Pp03_19842-3p	18791761	PRUPE_ppa001206mg	NCBI_Assembly:GCF_000346465.1	NW_006760385.1	-	34584685	34590550	5865	-	-	GO:0005375:copper ion transmembrane transporter activity;GO:0005524:ATP binding;GO:0019829:cation-transporting ATPase activity;GO:0046872:metal ion binding	GO:0016021:integral component of membrane
Pp03_19842-3p	18791781	PRUPE_ppa023748mg	NCBI_Assembly:GCF_000346465.1	NW_006760385.1	-	35554083	35555211	1128	-	-	-	-
Pp03_19842-3p	18791816	PRUPE_ppa021710mg	NCBI_Assembly:GCF_000346465.1	NW_006760385.1	+	1549481	1551763	2282	-	-	GO:0016491:oxidoreductase activity	-
Pp03_19842-3p	18791876	PRUPE_ppa009064mg	NCBI_Assembly:GCF_000346465.1	NW_006760385.1	+	24351664	24354669	3005	-	-	-	GO:0016020:membrane

Pp03_19842-3p	18791883	PRUPE_ppa014300mg	NCBI_Assembly:GCF_000346465.1	NW_006760385.1	+	27974733	27975757	1024	-	-	-	-
Pp03_19842-3p	18792136	PRUPE_ppa014196mg	NCBI_Assembly:GCF_000346465.1	NW_006760385.1	-	35389493	35390038	545	-	-	-	-
Pp03_19842-3p	18792234	PRUPE_ppa000819mg	NCBI_Assembly:GCF_000346465.1	NW_006760385.1	-	32689854	32694015	4161	-	-	-	-
Pp03_19842-3p	18792268	PRUPE_ppa025233mg	NCBI_Assembly:GCF_000346465.1	NW_006760385.1	-	8663333	8663932	599	-	-	-	-

Pp03_19842-3p	18792407	PRUPE_ppa004257mg	NCBI_Assembly:GCF_000346465.1	NW_006760385.1	+	41829094	41832969	3875	pper00970:Aminoacyl-tRNA biosynthesis	GO:0000096:sulfur amino acid metabolic process;GO:0006364:rRNA processing;GO:0006434:seryl-tRNA aminoacylation;GO:0007005:mitochondrion organization;GO:0008652:cellular amino acid biosynthetic process;GO:0009069:serine family amino acid metabolic process;GO:0009793:embryo development ending in seed dormancy;GO:0009902:chloroplast relocation;GO:0009965:leaf morphogenesis;GO:0010027:thylakoid	GO:0004828:serine-tRNA ligase activity;GO:0005524:ATP binding	GO:0005739:mitochondrion;GO:0009507:chloroplast
Pp03_19842-3p	18792660	PRUPE_ppa013163mg	NCBI_Assembly:GCF_000346465.1	NW_006760385.1	-	46285077	46287878	2801	-	-	-	-
Pp03_19842-3p	18792698	PRUPE_ppa002933mg	NCBI_Assembly:GCF_000346465.1	NW_006760385.1	+	26739627	26743279	3652	-	-	-	-

Pp03_19842-3p	18793667	PRUPE_ppa001416mg	NCBI_Assembly:GCF_000346465.1	NW_006760385.1	-	35355380	35360766	5386	-	GO:0000280:nuclear division;GO:0000911:cytokinesis by cell plate formation;GO:0006270:DNA replication initiation;GO:0006275:regulation of DNA replication;GO:0006306:DNA methylation;GO:0006346:methylation-dependent chromatin silencing;GO:0007018:microtubule-based movement;GO:0008283:cell proliferation;GO:0009909:regulation of flower development;GO:0010389:regulation of G2/M transition of mitotic cell	GO:0003777:microtubule motor activity;GO:0005524:ATP binding	GO:0005871:kinesin complex;GO:0005874:microtubule
Pp03_22312-3p(vvi-miR2950-5p)	18766048	PRUPE_ppa023642mg	NCBI_Assembly:GCF_000346465.1	NW_006760186.1	+	208729	213280	4551	-	-	GO:0043531:ADP binding	-

Pp03_22312-3p(vvi-miR2950-5p)	18766280	PRUPE_ppa008929mg	NCBI_Assembly:GCF_000346465.1	NW_006760194.1	+		21575002	21578412	3410	-	GO:0055085:transmembrane transport	GO:0051724:NAD transporter activity	GO:0005739:mitochondrion;GO:0016021:integral component of membrane
Pp03_22312-3p(vvi-miR2950-5p)	18766370	PRUPE_ppa001025mg	NCBI_Assembly:GCF_000346465.1	NW_006760194.1	+		12131307	12137485	6178	-	-	GO:0004672:protein kinase activity;GO:0005524:ATP binding	-
Pp03_22312-3p(vvi-miR2950-5p)	18766506	PRUPE_ppa009218mg	NCBI_Assembly:GCF_000346465.1	NW_006760194.1	-		19328898	19331052	2154	pper03018:RNA degradation	-	GO:0005524:ATP binding;GO:0016887:ATPase activity	-
Pp03_22312-3p(vvi-miR2950-5p)	18766609	PRUPE_ppa013743mg	NCBI_Assembly:GCF_000346465.1	NW_006760194.1	+		18175589	18176390	801	pper01100:Metabolic pathways;pper00510:N-Glycan biosynthesis	GO:0009853:photorespiration	-	GO:0005783:endoplasmic reticulum;GO:0033185:dolichol-phosphate-mannose synthase complex
Pp03_22312-3p(vvi-miR2950-5p)	18766702	PRUPE_ppa020331mg	NCBI_Assembly:GCF_000346465.1	NW_006760194.1	-		17669750	17672135	2385	-	-	GO:0003723:RNA binding;GO:0008168:methyltransferase activity	-

Pp03_22312-3p(vvi-miR2950-5p)	18766740	PRUPE_ppa002766mg	NCBI_Assembly:GCF_000346465.1	NW_006760194.1	-		4488714	4496250	7536	-	GO:0006399:tRNA metabolic process;GO:0009658:chloroplast organization; GO:0080158:chloroplast ribulose bisphosphate carboxylase complex biogenesis;GO:1901259:chloroplast rRNA processing	GO:0004386:helicase activity;GO:0005524:ATP binding;GO:0016887:ATPase activity;GO:0070180:large ribosomal subunit rRNA binding	GO:0009507:chloroplast
Pp03_22312-3p(vvi-miR2950-5p)	18766896	PRUPE_ppa022797mg	NCBI_Assembly:GCF_000346465.1	NW_006760194.1	+		1691595	1696306	4711	-	GO:0009855:determination of bilateral symmetry;GO:0009926:auxin polar transport;GO:0010014:meristem initiation;GO:0010073:meristem maintenance;GO:0048451:petal formation;GO:0048453:sepal formation;GO:0055085:transmembrane transport	-	GO:0005886:plasma membrane;GO:0016021:integral component of membrane
Pp03_22312-3p(vvi-miR2950-5p)	18767090	PRUPE_ppa020353mg	NCBI_Assembly:GCF_000346465.1	NW_006760194.1	+		21193658	21193969	311	-	-	-	-

Pp03_22312-3p(vvi-miR2950-5p)	18767163	PRUPE_ppa005303mg	NCBI_Assembly:GCF_000346465.1	NW_006760194.1	-	18577075	18580400	3325	pper01100:Metabolic pathways;pper01110:Biosynthesis of secondary metabolites;pper00071:Fatty acid degradation;pper00280:Valine, leucine and isoleucine degradation;pper01212:Fatty acid metabolism;pper04146:Peroxisome;pper00592:alpha-Linolenic acid metabolism;pper01040:Biosynthesis of unsaturated fatty acids	-	GO:0016747:transferase activity, transferring acyl groups other than amino-acyl groups	-
Pp03_22312-3p(vvi-miR2950-5p)	18767196	PRUPE_ppa000851mg	NCBI_Assembly:GCF_000346465.1	NW_006760194.1	+	3129187	3132135	2948	pper03040:Spliceosome	GO:0000398:mRNA splicing, via spliceosome	-	GO:0005634:nucleus
Pp03_22312-3p(vvi-miR2950-5p)	18767418	PRUPE_ppa003245mg	NCBI_Assembly:GCF_000346465.1	NW_006760194.1	+	616747	619447	2700	-	-	-	-
Pp03_22312-3p(vvi-miR2950-5p)	18767422	PRUPE_ppa020192mg	NCBI_Assembly:GCF_000346465.1	NW_006760194.1	+	13040476	13045350	4874	-	-	-	-

Pp03_22312-3p(vvi-miR2950-5p)	18767506	PRUPE_ppa004972mg	NCBI_Assembly:GCF_000346465.1	NW_006760194.1	+	14552719	14554366	1647	-	-	GO:0016758: transferase activity, transferring hexosyl groups	-	
Pp03_22312-3p(vvi-miR2950-5p)	18767513	PRUPE_ppa005420mg	NCBI_Assembly:GCF_000346465.1	NW_006760194.1	-	15559500	15564806	5306	-	-	GO:0004185: serine-type carboxypeptidase activity	-	
Pp03_22312-3p(vvi-miR2950-5p)	18767537	PRUPE_ppa023265mg	NCBI_Assembly:GCF_000346465.1	NW_006760194.1	+	1743435	1744832	1397	pper00061:Fatty acid biosynthesis; pper01212:Fatty acid metabolism;pper01040:Biosynthesis of unsaturated fatty acids	GO:0006631: fatty acid metabolic process	GO:0045300: acyl-[acyl-carrier-protein] desaturase activity	-	
Pp03_22312-3p(vvi-miR2950-5p)	18767579	PRUPE_ppa025972mg	NCBI_Assembly:GCF_000346465.1	NW_006760194.1	-	11759786	11763562	3776	-	-	GO:0005215: transporter activity	GO:0005622: intracellular	

Pp03_22312-3p(vvi-miR2950-5p)	18767691	PRUPE_ppa021570mg	NCBI_Assembly:GCF_000346465.1	NW_006760194.1	+	15692832	15699571	6739	pper01100:Metabolic pathways;pper00562:Inositol phosphate metabolism;pper04070:Phosphatidylinositol signaling system	GO:0032957:inositol trisphosphate metabolic process	GO:0000287:magnesium ion binding;GO:0005524:ATP binding;GO:0047325:inositol tetrakisphosphate 1-kinase activity;GO:0052725:inositol-1,3,4-trisphosphate 6-kinase activity;GO:0052726:inositol-1,3,4-trisphosphate 5-kinase activity	GO:0005622:intracellular
Pp03_22312-3p(vvi-miR2950-5p)	18767693	PRUPE_ppa012772mg	NCBI_Assembly:GCF_000346465.1	NW_006760194.1	+	14316181	14316697	516	pper03010:Ribosome	GO:0006412:translation	GO:0003735:structural constituent of ribosome	GO:0005840:ribosome
Pp03_22312-3p(vvi-miR2950-5p)	18767703	PRUPE_ppa021097mg	NCBI_Assembly:GCF_000346465.1	NW_006760194.1	-	2614200	2616702	2502	-	-	GO:0003682:chromatin binding;GO:0003700:sequence-specific DNA binding transcription factor activity	-
Pp03_22312-3p(vvi-miR2950-5p)	18768608	PRUPE_ppa009538mg	NCBI_Assembly:GCF_000346465.1	NW_006760194.1	-	16391219	16395787	4568	pper00480:Glutathione metabolism;pper00053:Ascorbate and aldarate metabolism	GO:0006979:response to oxidative stress	GO:0004601:peroxidase activity;GO:0020037:heme binding	-

Pp03_22312-3p(vvi-miR2950-5p)	18768866	PRUPE_ppa001002mg	NCBI_Assembly:GCF_000346465.1	NW_006760194.1	+	355877	367230	11353	pper04144:Endocytosis	-	GO:0003924:GTPase activity;GO:0005525:GTP binding	-
Pp03_22312-3p(vvi-miR2950-5p)	18768945	PRUPE_ppa005333mg	NCBI_Assembly:GCF_000346465.1	NW_006760194.1	+	14894503	14900257	5754	pper00270:Cysteine and methionine metabolism;pper01100:Metabolic pathways	GO:0009086:methionine biosynthetic process	GO:0046522:S-methyl-5-thioribose kinase activity	-
Pp03_22312-3p(vvi-miR2950-5p)	18768975	PRUPE_ppa020367mg	NCBI_Assembly:GCF_000346465.1	NW_006760194.1	-	15908576	15908971	395	-	-	-	GO:0005576:extracellular region
Pp03_22312-3p(vvi-miR2950-5p)	18769029	PRUPE_ppa009585mg	NCBI_Assembly:GCF_000346465.1	NW_006760201.1	-	21844141	21845830	1689	-	GO:0006470:protein dephosphorylation	GO:0004722:protein serine/threonine phosphatase activity;GO:0046872:metal ion binding	-
Pp03_22312-3p(vvi-miR2950-5p)	18769033	PRUPE_ppa003533mg	NCBI_Assembly:GCF_000346465.1	NW_006760201.1	-	17031049	17033787	2738	-	GO:0006661:phosphatidylinositol biosynthetic process	-	-
Pp03_22312-3p(vvi-miR2950-5p)	18769210	PRUPE_ppa007064mg	NCBI_Assembly:GCF_000346465.1	NW_006760201.1	-	21122996	21126829	3833	pper01100:Metabolic pathways;pper00564:Glycerophospholipid metabolism	-	GO:0016772:transferase activity, transferring phosphorus-containing groups	-
Pp03_22312-3p(vvi-miR2950-5p)	18769289	PRUPE_ppa014814mg	NCBI_Assembly:GCF_000346465.1	NW_006760201.1	-	462475	463452	977	-	-	GO:0003677:DNA binding	-
Pp03_22312-3p(vvi-miR2950-5p)	18769694	PRUPE_ppa026757mg	NCBI_Assembly:GCF_000346465.1	NW_006760201.1	-	3446563	3451783	5220	-	-	-	-

Pp03_22312-3p(vvi-miR2950-5p)	18769749	PRUPE_ppa013193mg	NCBI_Assembly:GCF_000346465.1	NW_006760201.1	+		12895366	12896784	1418	pper03010:Ribosome	GO:0006354:DNA-templated transcription, elongation;GO:0006412:translation	GO:0003735:structural constituent of ribosome;GO:0019843:rRNA binding	GO:0005840:ribosome
Pp03_22312-3p(vvi-miR2950-5p)	18769783	PRUPE_ppa022289mg	NCBI_Assembly:GCF_000346465.1	NW_006760201.1	+		11767578	11769368	1790	-	-	-	-
Pp03_22312-3p(vvi-miR2950-5p)	18769813	PRUPE_ppa019096mg	NCBI_Assembly:GCF_000346465.1	NW_006760201.1	-		21094776	21095915	1139	-	GO:0007017:microtubule-based process	-	GO:0005875:microtubule associated complex
Pp03_22312-3p(vvi-miR2950-5p)	18769848	PRUPE_ppa009206mg	NCBI_Assembly:GCF_000346465.1	NW_006760201.1	+		20623541	20626156	2615	pper04144:Endocytosis;pper04145:Phagosome	GO:0007264:small GTPase mediated signal transduction;GO:0015031:protein transport	GO:0005525:GTP binding	GO:0005622:intracellular
Pp03_22312-3p(vvi-miR2950-5p)	18769857	PRUPE_ppa009223mg	NCBI_Assembly:GCF_000346465.1	NW_006760201.1	+		19781231	19782354	1123	-	-	-	-
Pp03_22312-3p(vvi-miR2950-5p)	18769898	PRUPE_ppa025058mg	NCBI_Assembly:GCF_000346465.1	NW_006760201.1	-		14235398	14236024	626	-	-	-	-
Pp03_22312-3p(vvi-miR2950-5p)	18769944	PRUPE_ppb015516mg	NCBI_Assembly:GCF_000346465.1	NW_006760201.1	+		6986615	6988378	1763	-	-	-	-
Pp03_22312-3p(vvi-miR2950-5p)	18770184	PRUPE_ppa014123mg	NCBI_Assembly:GCF_000346465.1	NW_006760201.1	+		19647249	19649507	2258	-	-	-	-
Pp03_22312-3p(vvi-miR2950-5p)	18770259	PRUPE_ppa002030mg	NCBI_Assembly:GCF_000346465.1	NW_006760201.1	-		21743785	21750308	6523	-	GO:0019344:cysteine biosynthetic process	GO:0008289:lipid binding	GO:0005886:plasma membrane

Pp03_22312-3p(vvi-miR2950-5p)	18770263	PRUPE_ppa000940mg	NCBI_Assembly:GCF_000346465.1	NW_006760201.1	+	16161368	16164235	2867	-	-	GO:0003824:catalytic activity;GO:0030170:pyridoxal phosphate binding	-	
Pp03_22312-3p(vvi-miR2950-5p)	18770349	PRUPE_ppa013378mg	NCBI_Assembly:GCF_000346465.1	NW_006760201.1	+	8087404	8089004	1600	-	-	-	-	
Pp03_22312-3p(vvi-miR2950-5p)	18770380	PRUPE_ppa017355mg	NCBI_Assembly:GCF_000346465.1	NW_006760201.1	-	10870330	10870590	260	-	-	-	-	
Pp03_22312-3p(vvi-miR2950-5p)	18770535	PRUPE_ppa016232mg	NCBI_Assembly:GCF_000346465.1	NW_006760201.1	-	15932107	15935203	3096	-	-	GO:0043531:ADP binding	-	
Pp03_22312-3p(vvi-miR2950-5p)	18770536	PRUPE_ppa023427mg	NCBI_Assembly:GCF_000346465.1	NW_006760201.1	-	22151699	22153818	2119	-	-	GO:0004672:protein kinase activity;GO:0005524:ATP binding	-	
Pp03_22312-3p(vvi-miR2950-5p)	18770703	PRUPE_ppa010582mg	NCBI_Assembly:GCF_000346465.1	NW_006760201.1	+	13170197	13171591	1394	-	-	-	-	
Pp03_22312-3p(vvi-miR2950-5p)	18770793	PRUPE_ppa002924mg	NCBI_Assembly:GCF_000346465.1	NW_006760201.1	-	8342599	8344788	2189	-	-	-	-	
Pp03_22312-3p(vvi-miR2950-5p)	18770893	PRUPE_ppa024619mg	NCBI_Assembly:GCF_000346465.1	NW_006760201.1	-	16100323	16101735	1412	-	-	-	-	
Pp03_22312-3p(vvi-miR2950-5p)	18770973	PRUPE_ppa002792mg	NCBI_Assembly:GCF_000346465.1	NW_006760201.1	+	13202198	13207088	4890	-	GO:0006887:exocytosis	-	GO:0000145:exocyst	
Pp03_22312-3p(vvi-miR2950-5p)	18771270	PRUPE_ppa026645mg	NCBI_Assembly:GCF_000346465.1	NW_006760201.1	+	13109026	13111144	2118	-	GO:0015743:malate transport	-	-	
Pp03_22312-3p(vvi-miR2950-5p)	18771406	PRUPE_ppa007584mg	NCBI_Assembly:GCF_000346465.1	NW_006760201.1	+	15866102	15870803	4701	pper04075:Plant hormone signal transduction	-	GO:0004672:protein kinase activity;GO:0005524:ATP binding	-	

Pp03_22312-3p(vvi-miR2950-5p)	18771427	PRUPE_ppa009153mg	NCBI_Assembly:GCF_000346465.1	NW_006760201.1	-	7708171	7711579	3408	-	GO:0046685: response to arsenic-containing substance;GO:0048573:photoperiodism, flowering	GO:0016787: hydrolase activity	-
Pp03_22312-3p(vvi-miR2950-5p)	18771789	PRUPE_ppa025276mg	NCBI_Assembly:GCF_000346465.1	NW_006760201.1	+	18161805	18164122	2317	-	-	-	-
Pp03_22312-3p(vvi-miR2950-5p)	18771909	PRUPE_ppa017543mg	NCBI_Assembly:GCF_000346465.1	NW_006760201.1	+	16263904	16264215	311	-	-	-	-
Pp03_22312-3p(vvi-miR2950-5p)	18772023	PRUPE_ppa012517mg	NCBI_Assembly:GCF_000346465.1	NW_006760208.1	-	2446550	2448638	2088	-	GO:0051252: regulation of RNA metabolic process	GO:0008428: ribonuclease inhibitor activity;GO:0008948:oxaloacetate decarboxylase activity;GO:0046872:metal ion binding;GO:0047443:4-hydroxy-4-methyl-2-oxoglutarate aldolase activity	-
Pp03_22312-3p(vvi-miR2950-5p)	18772032	PRUPE_ppa022354mg	NCBI_Assembly:GCF_000346465.1	NW_006760208.1	-	823994	826925	2931	-	-	-	-

Pp03_22312-3p(vvi-miR2950-5p)	18772061	PRUPE_ppa000777mg	NCBI_Assembly:GCF_000346465.1	NW_006760208.1	-	6131023	6140274	9251	-	GO:0009638:phototropism;GO:0009644:response to high light intensity;GO:0009744:response to sucrose;GO:0009903:chloroplast avoidance movement;GO:0009904:chloroplast accumulation movement;GO:0010114:response to red light;GO:0010119:regulation of stomatal movement;GO:0010155:regulation of proton transport;GO:0010218:response to far red light;GO:0010362:negative regulation of anion channel activity by blue	GO:0000155:phosphorelay sensor kinase activity;GO:0005524:ATP binding;GO:0009882:blue light photoreceptor activity;GO:0010181:FMN binding	GO:0005773:vacuole;GO:0009898:cytoplasmic side of plasma membrane;GO:0009986:cell surface
Pp03_22312-3p(vvi-miR2950-5p)	18772086	PRUPE_ppa014249mg	NCBI_Assembly:GCF_000346465.1	NW_006760208.1	+	17533894	17534340	446	-	-	-	-
Pp03_22312-3p(vvi-miR2950-5p)	18772288	PRUPE_ppa011211mg	NCBI_Assembly:GCF_000346465.1	NW_006760208.1	-	848505	850410	1905	-	GO:0006783:heme biosynthetic process	GO:0004045:aminoacyl-tRNA hydrolase activity	-

Pp03_22312-3p(vvi-miR2950-5p)	18772298	PRUPE_ppa011615mg	NCBI_Assembly:GCF_000346465.1	NW_006760208.1	-	3163541	3165606	2065	-	-	-	-
Pp03_22312-3p(vvi-miR2950-5p)	18772586	PRUPE_ppa020985mg	NCBI_Assembly:GCF_000346465.1	NW_006760208.1	+	27583823	27589905	6082	-	GO:0001522:pseudouridine synthesis	GO:0003723:RNA binding;GO:0009982:pseudouridine synthase activity	-
Pp03_22312-3p(vvi-miR2950-5p)	18772613	PRUPE_ppa006979mg	NCBI_Assembly:GCF_000346465.1	NW_006760208.1	-	26866817	26869716	2899	pper01100:Metabolic pathways;pper01110:Biosynthesis of secondary metabolites;pper04146:Peroxisome;pper00900:Terpenoid backbone biosynthesis	GO:0000280:nuclear division;GO:0007000:nucleolus organization;GO:0008299:isoprenoid biosynthetic process	GO:0004496:mevalonate kinase activity;GO:0005524:ATP binding	GO:0005829:cytosol

Pp03_22312-3p(vvi-miR2950-5p)	18772818	PRUPE_ppa004544mg	NCBI_Assembly:GCF_000346465.1	NW_006760208.1	-	2186962	2190064	3102	pper00130:Ubiquinone and other terpenoid-quinone biosynthesis;pper00360:Phenylalanine metabolism;pper01100:Metabolic pathways;pper01110:Biosynthesis of secondary metabolites;pper00940:Phenylpropanoid biosynthesis;pper00941:Flavonoid biosynthesis;pper01220:Degradation of aromatic compounds;pper00945:Stilbenoid, diarylheptanoid and gingerol biosynthesis	GO:0009555:pollen development;GO:0009611:response to wounding;GO:0009805:coumarin biosynthetic process;GO:0009808:lignin metabolic process;GO:0009963:positive regulation of flavonoid biosynthetic process;GO:0040007:growth	GO:0004497:monooxygenase activity;GO:0005506:iron ion binding;GO:0016705:oxidoreductase activity, acting on paired donors, with incorporation or reduction of molecular oxygen;GO:0020037:heme binding	GO:0005774:vacuolar membrane;GO:0005783:endoplasmic reticulum;GO:0005794:Golgi apparatus;GO:0005886:plasma membrane;GO:0009505:plant-type cell wall;GO:0009506:plasmodesma
Pp03_22312-3p(vvi-miR2950-5p)	18772869	PRUPE_ppa002959mg	NCBI_Assembly:GCF_000346465.1	NW_006760208.1	-	721654	725527	3873	-	-	GO:0003676:nucleic acid binding;GO:0004386:helicase activity;GO:0005524:ATP binding	-

Pp03_22312-3p(vvi-miR2950-5p)	18772916	PRUPE_ppa009532mg	NCBI_Assembly:GCF_000346465.1	NW_006760208.1	-	26457803	26459840	2037	-	-	-	-
Pp03_22312-3p(vvi-miR2950-5p)	18774406	PRUPE_ppa003208mg	NCBI_Assembly:GCF_000346465.1	NW_006760208.1	+	22955313	22960433	5120	-	-	GO:0003677:DNA binding;GO:0003682:chromatin binding;GO:0003700:sequence-specific DNA binding transcription factor activity	-
Pp03_22312-3p(vvi-miR2950-5p)	18774415	PRUPE_ppa000706mg	NCBI_Assembly:GCF_000346465.1	NW_006760208.1	-	21520169	21529519	9350	-	GO:0007018:microtubule-based movement	GO:0003777:microtubule motor activity;GO:0005524:ATP binding	GO:0005871:kinesin complex;GO:0005874:microtubule
Pp03_22312-3p(vvi-miR2950-5p)	18774537	PRUPE_ppa018851mg	NCBI_Assembly:GCF_000346465.1	NW_006760208.1	-	17907214	17908025	811	-	-	GO:0004672:protein kinase activity;GO:0005524:ATP binding	-
Pp03_22312-3p(vvi-miR2950-5p)	18775867	PRUPE_ppa017209mg	NCBI_Assembly:GCF_000346465.1	NW_006760212.1	-	5525811	5527412	1601	-	-	-	-
Pp03_22312-3p(vvi-miR2950-5p)	18775946	PRUPE_ppa018176mg	NCBI_Assembly:GCF_000346465.1	NW_006760212.1	+	8243925	8245004	1079	-	-	GO:0004222:metalloendopeptidase activity;GO:0008270:zinc ion binding	GO:0031012:extracellular matrix
Pp03_22312-3p(vvi-miR2950-5p)	18775989	PRUPE_ppa021506mg	NCBI_Assembly:GCF_000346465.1	NW_006760212.1	+	12649801	12651659	1858	-	-	GO:0016788:hydrolase activity, acting on ester bonds	-
Pp03_22312-3p(vvi-miR2950-5p)	18777239	PRUPE_ppa007867mg	NCBI_Assembly:GCF_000346465.1	NW_006760212.1	-	17822009	17824633	2624	-	-	-	GO:0016020:membrane

Pp03_22312-3p(vvi-miR2950-5p)	18777337	PRUPE_ppa016054mg	NCBI_Assembly:GCF_000346465.1	NW_006760212.1	-	15542082	15542737	655	-	-	GO:0008270:zinc ion binding	-
Pp03_22312-3p(vvi-miR2950-5p)	18777373	PRUPE_ppa019937mg	NCBI_Assembly:GCF_000346465.1	NW_006760212.1	-	12282901	12283311	410	-	-	-	-
Pp03_22312-3p(vvi-miR2950-5p)	18777392	PRUPE_ppa019709mg	NCBI_Assembly:GCF_000346465.1	NW_006760212.1	-	13774270	13775565	1295	pper04626:Plant-pathogen interaction	-	GO:0005509:calcium ion binding	-
Pp03_22312-3p(vvi-miR2950-5p)	18777679	PRUPE_ppa001777mg	NCBI_Assembly:GCF_000346465.1	NW_006760212.1	-	8784793	8793005	8212	-	-	-	-
Pp03_22312-3p(vvi-miR2950-5p)	18777706	PRUPE_ppa020153mg	NCBI_Assembly:GCF_000346465.1	NW_006760212.1	+	17269333	17270046	713	-	-	-	-
Pp03_22312-3p(vvi-miR2950-5p)	18777713	PRUPE_ppa026612mg	NCBI_Assembly:GCF_000346465.1	NW_006760212.1	-	13023054	13027542	4488	-	-	-	-
Pp03_22312-3p(vvi-miR2950-5p)	18777799	PRUPE_ppa024742mg	NCBI_Assembly:GCF_000346465.1	NW_006760212.1	+	8155625	8156335	710	pper01100:Metabolic pathways;pper00230:Purine metabolism;pper00240:Pyrimidine metabolism;pper03020:RNA polymerase	GO:0006351:transcription, DNA-templated	GO:0003677:DNA binding;GO:0003899:DNA-directed RNA polymerase activity	-
Pp03_22312-3p(vvi-miR2950-5p)	18777894	PRUPE_ppa014838mg	NCBI_Assembly:GCF_000346465.1	NW_006760212.1	-	16588065	16590429	2364	-	GO:0010215:cellulose microfibril organization;GO:0016049:cell growth	-	GO:0031225:anchored component of membrane
Pp03_22312-3p(vvi-miR2950-5p)	18778050	PRUPE_ppa007231mg	NCBI_Assembly:GCF_000346465.1	NW_006760212.1	+	4176405	4181870	5465	-	-	GO:0003677:DNA binding	-
Pp03_22312-3p(vvi-miR2950-5p)	18778117	PRUPE_ppa010653mg	NCBI_Assembly:GCF_000346465.1	NW_006760212.1	-	10327790	10329216	1426	-	-	-	-

Pp03_22312-3p(vvi-miR2950-5p)	18778177	PRUPE_ppa002754mg	NCBI_Assembly:GCF_000346465.1	NW_006760220.1	+		4389399	4393316	3917	-	-	-	GO:0005886:plasma membrane
Pp03_22312-3p(vvi-miR2950-5p)	18778364	PRUPE_ppa024259mg	NCBI_Assembly:GCF_000346465.1	NW_006760220.1	+		4132264	4133506	1242	-	-	GO:0016772:transferase activity, transferring phosphorus-containing groups	-
Pp03_22312-3p(vvi-miR2950-5p)	18778400	PRUPE_ppa020218mg	NCBI_Assembly:GCF_000346465.1	NW_006760220.1	+		12713489	12718628	5139	pper02010:ABC transporters	-	GO:0005524:ATP binding;GO:0042626:ATPase activity, coupled to transmembrane movement of substances	GO:0016021:integral component of membrane
Pp03_22312-3p(vvi-miR2950-5p)	18778463	PRUPE_ppa021803mg	NCBI_Assembly:GCF_000346465.1	NW_006760220.1	+		8900632	8901434	802	-	-	-	-
Pp03_22312-3p(vvi-miR2950-5p)	18778517	PRUPE_ppa012836mg	NCBI_Assembly:GCF_000346465.1	NW_006760220.1	-		17909158	17911127	1969	-	-	-	-
Pp03_22312-3p(vvi-miR2950-5p)	18778547	PRUPE_ppa024609mg	NCBI_Assembly:GCF_000346465.1	NW_006760220.1	-		18415021	18415916	895	-	GO:0006886:intracellular protein transport;GO:0016192:vesicle-mediated transport	-	GO:0030131:clathrin adaptor complex
Pp03_22312-3p(vvi-miR2950-5p)	18778641	PRUPE_ppa013009mg	NCBI_Assembly:GCF_000346465.1	NW_006760220.1	+		10752022	10753441	1419	-	-	-	-
Pp03_22312-3p(vvi-miR2950-5p)	18778687	PRUPE_ppa022500mg	NCBI_Assembly:GCF_000346465.1	NW_006760220.1	-		13111873	13112571	698	-	-	-	-

Pp03_22312-3p(vvi-miR2950-5p)	18778742	PRUPE_ppa004317mg	NCBI_Assembly:GCF_000346465.1	NW_006760220.1	-	4996978	4998552	1574	-	-	GO:0008762:UDP-N-acetylmuramate dehydrogenase activity;GO:0050660:flavin adenine dinucleotide binding	-
Pp03_22312-3p(vvi-miR2950-5p)	18778805	PRUPE_ppa020588mg	NCBI_Assembly:GCF_000346465.1	NW_006760220.1	+	29048801	29049049	248	-	-	-	-
Pp03_22312-3p(vvi-miR2950-5p)	18778869	PRUPE_ppa010079mg	NCBI_Assembly:GCF_000346465.1	NW_006760220.1	-	2833406	2836477	3071	-	-	-	-
Pp03_22312-3p(vvi-miR2950-5p)	18778938	PRUPE_ppa009183mg	NCBI_Assembly:GCF_000346465.1	NW_006760220.1	-	2413424	2415581	2157	-	-	GO:0000064:L-ornithine transmembrane transporter activity;GO:0005290:L-histidine transmembrane transporter activity;GO:0015181:arginine transmembrane transporter activity;GO:0015189:L-lysine transmembrane transporter activity	GO:0016021:integral component of membrane

Pp03_22312-3p(vvi-miR2950-5p)	18779020	PRUPE_ppa017208mg	NCBI_Assembly:GCF_000346465.1	NW_006760220.1	-	13891181	13891627	446	-	GO:0045893: positive regulation of transcription, DNA-templated	-	-
Pp03_22312-3p(vvi-miR2950-5p)	18779234	PRUPE_ppa012872mg	NCBI_Assembly:GCF_000346465.1	NW_006760220.1	-	29832490	29833059	569	-	-	-	-
Pp03_22312-3p(vvi-miR2950-5p)	18779368	PRUPE_ppa027110mg	NCBI_Assembly:GCF_000346465.1	NW_006760220.1	-	3742905	3744120	1215	-	-	GO:0004672: protein kinase activity;GO:0005524:ATP binding	-
Pp03_22312-3p(vvi-miR2950-5p)	18779546	PRUPE_ppa018117mg	NCBI_Assembly:GCF_000346465.1	NW_006760220.1	-	7185525	7189718	4193	-	-	GO:0004672: protein kinase activity;GO:0005524:ATP binding	-
Pp03_22312-3p(vvi-miR2950-5p)	18779589	PRUPE_ppa025938mg	NCBI_Assembly:GCF_000346465.1	NW_006760220.1	+	4826042	4827177	1135	-	-	GO:0003824: catalytic activity	-
Pp03_22312-3p(vvi-miR2950-5p)	18779647	PRUPE_ppa018602mg	NCBI_Assembly:GCF_000346465.1	NW_006760220.1	+	9680158	9681663	1505	-	-	GO:0005524: ATP binding	-
Pp03_22312-3p(vvi-miR2950-5p)	18779770	PRUPE_ppa015170mg	NCBI_Assembly:GCF_000346465.1	NW_006760220.1	-	27188060	27189536	1476	-	-	-	-
Pp03_22312-3p(vvi-miR2950-5p)	18779807	PRUPE_ppa017622mg	NCBI_Assembly:GCF_000346465.1	NW_006760220.1	-	560189	560887	698	-	-	-	-
Pp03_22312-3p(vvi-miR2950-5p)	18779828	PRUPE_ppa018356mg	NCBI_Assembly:GCF_000346465.1	NW_006760220.1	+	21973079	21973486	407	-	-	GO:0008270: zinc ion binding	-

Pp03_22312-3p(vvi-miR2950-5p)	18779892	PRUPE_ppa018681mg	NCBI_Assembly:GCF_000346465.1	NW_006760220.1	-	134229	137722	3493	pper01110:Biosynthesis of secondary metabolites;pper00500:Starch and sucrose metabolism;pper00520:Amino sugar and nucleotide sugar metabolism	GO:0045489:pectin biosynthetic process;GO:0071555:cell wall organization	GO:0047262:polygalacturonate 4-alpha-galacturonosyltransferase activity	GO:0000139:Golgi membrane
Pp03_22312-3p(vvi-miR2950-5p)	18780184	PRUPE_ppa013577mg	NCBI_Assembly:GCF_000346465.1	NW_006760220.1	+	1559981	1560580	599	-	-	GO:0005509:calcium ion binding	-
Pp03_22312-3p(vvi-miR2950-5p)	18780188	PRUPE_ppa024485mg	NCBI_Assembly:GCF_000346465.1	NW_006760220.1	+	1333191	1333967	776	-	-	-	-
Pp03_22312-3p(vvi-miR2950-5p)	18780220	PRUPE_ppa019446mg	NCBI_Assembly:GCF_000346465.1	NW_006760220.1	+	4106164	4106490	326	-	GO:0045454:cell redox homeostasis	GO:0009055:electron carrier activity;GO:0015035:protein disulfide oxidoreductase activity	GO:0005623:cell

Pp03_22312-3p(vvi-miR2950-5p)	18780321	PRUPE_ppa003872mg	NCBI_Assembly:GCF_000346465.1	NW_006760220.1	-	864784	868963	4179	-	GO:0000271:polysaccharide biosynthetic process;GO:0009825:multidimensional cell growth;GO:0009932:cell tip growth;GO:0010075:regulation of meristem growth;GO:0010817:regulation of hormone levels;GO:0016126:sterol biosynthetic process;GO:0043481:anthocyanin accumulation in tissues in response to UV light;GO:0048653:anther development;GO:0048767:root hair elongation;GO:0071555:cell wall organization	GO:0005507:copper ion binding;GO:0016491:oxidoreductase activity	GO:0009505:plant-type cell wall;GO:0009506:plasmodesma;GO:0016020:membrane
Pp03_22312-3p(vvi-miR2950-5p)	18780577	PRUPE_ppa018233mg	NCBI_Assembly:GCF_000346465.1	NW_006760220.1	+	14720984	14721285	301	-	-	-	-

Pp03_22312-3p(vvi-miR2950-5p)	18780604	PRUPE_ppa000416mg	NCBI_Assembly:GCF_000346465.1	NW_006760220.1	-	25730188	25741974	11786	pper03440:Homologous recombination	GO:0006260:DNA replication;GO:0006281:DNA repair;GO:0006310:DNA recombination	GO:0003676:nucleic acid binding;GO:0005524:ATP binding;GO:0043140:ATP-dependent 3'-5' DNA helicase activity	GO:0005622:intracellular
Pp03_22312-3p(vvi-miR2950-5p)	18780605	PRUPE_ppa016487mg	NCBI_Assembly:GCF_000346465.1	NW_006760220.1	-	140417	142612	2195	-	-	-	-
Pp03_22312-3p(vvi-miR2950-5p)	18781530	PRUPE_ppa025063mg	NCBI_Assembly:GCF_000346465.1	NW_006760220.1	-	7818909	7825543	6634	-	-	-	-
Pp03_22312-3p(vvi-miR2950-5p)	18783384	PRUPE_ppa010701mg	NCBI_Assembly:GCF_000346465.1	NW_006760268.1	+	9063980	9071156	7176	pper01100:Metabolic pathways;pper00430:Taurine and hypotaurine metabolism	-	GO:0047800:cysteamine dioxygenase activity	-
Pp03_22312-3p(vvi-miR2950-5p)	18783645	PRUPE_ppa019507mg	NCBI_Assembly:GCF_000346465.1	NW_006760268.1	+	20600315	20603433	3118	-	-	GO:0003677:DNA binding;GO:0003682:chromatin binding	-
Pp03_22312-3p(vvi-miR2950-5p)	18783866	PRUPE_ppa022787mg	NCBI_Assembly:GCF_000346465.1	NW_006760268.1	-	1233670	1234539	869	-	GO:0009965:leaf morphogenesis;GO:0030154:cell differentiation;GO:0045893:positive regulation of transcription, DNA-templated	-	-

Pp03_22312-3p(vvi-miR2950-5p)	18783916	PRUPE_ppa024908mg	NCBI_Assembly:GCF_000346465.1	NW_006760268.1	-	6780668	6781669	1001	-	-	GO:0004672:protein kinase activity;GO:0005524:ATP binding	-
Pp03_22312-3p(vvi-miR2950-5p)	18784072	PRUPE_ppb024238mg	NCBI_Assembly:GCF_000346465.1	NW_006760268.1	+	9492901	9495185	2284	-	-	-	-
Pp03_22312-3p(vvi-miR2950-5p)	18784084	PRUPE_ppa020224mg	NCBI_Assembly:GCF_000346465.1	NW_006760268.1	+	7566139	7568501	2362	-	-	-	-
Pp03_22312-3p(vvi-miR2950-5p)	18784547	PRUPE_ppa025200mg	NCBI_Assembly:GCF_000346465.1	NW_006760322.1	-	1025	3032	2007	-	-	-	-
Pp03_22312-3p(vvi-miR2950-5p)	18784600	PRUPE_ppb013270mg	NCBI_Assembly:GCF_000346465.1	NW_006760324.1	+	23096613	23097213	600	-	-	-	-
Pp03_22312-3p(vvi-miR2950-5p)	18784631	PRUPE_ppa016139mg	NCBI_Assembly:GCF_000346465.1	NW_006760324.1	+	25643840	25645538	1698	-	-	-	-
Pp03_22312-3p(vvi-miR2950-5p)	18784640	PRUPE_ppa022057mg	NCBI_Assembly:GCF_000346465.1	NW_006760324.1	+	11692532	11694703	2171	pper01100:Metabolic pathways;pper00592:alpha-Linolenic acid metabolism	-	GO:0003824:catalytic activity	-
Pp03_22312-3p(vvi-miR2950-5p)	18784756	PRUPE_ppa013617mg	NCBI_Assembly:GCF_000346465.1	NW_006760324.1	-	12215846	12216539	693	-	-	-	-

Pp03_22312-3p(vvi-miR2950-5p)	18784809	PRUPE_ppa003388mg	NCBI_Assembly:GCF_000346465.1	NW_006760324.1	+	19014344	19019200	4856	pper04075:Plant hormone signal transduction	GO:0009864:induced systemic resistance, jasmonic acid mediated signaling pathway;GO:0010046:response to mycotoxin;GO:0010224:response to UV-B;GO:2000030:regulation of response to red or far red light	-	-
Pp03_22312-3p(vvi-miR2950-5p)	18784833	PRUPE_ppa023079mg	NCBI_Assembly:GCF_000346465.1	NW_006760324.1	-	25967384	25969064	1680	-	GO:0005975:carbohydrate metabolic process	GO:0004553:hydrolase activity, hydrolyzing O-glycosyl compounds	-
Pp03_22312-3p(vvi-miR2950-5p)	18784962	PRUPE_ppa021915mg	NCBI_Assembly:GCF_000346465.1	NW_006760324.1	+	3204873	3208022	3149	-	-	GO:0003676:nucleic acid binding	-
Pp03_22312-3p(vvi-miR2950-5p)	18785326	PRUPE_ppa007690mg	NCBI_Assembly:GCF_000346465.1	NW_006760324.1	+	20418777	20422205	3428	pper03015:mRNA surveillance pathway	-	-	-

Pp03_22312-3p(vvi-miR2950-5p)	18785452	PRUPE_ppa005774mg	NCBI_Assembly:GCF_000346465.1	NW_006760324.1	-	25210572	25212408	1836	-	GO:0010090:trichome morphogenesis;GO:0010143:cutin biosynthetic process;GO:0048730:epidermis morphogenesis;GO:0051179:localization	GO:0016747:transferase activity, transferring acyl groups other than amino-acyl groups	-
Pp03_22312-3p(vvi-miR2950-5p)	18785457	PRUPE_ppa009680mg	NCBI_Assembly:GCF_000346465.1	NW_006760324.1	+	23914211	23915735	1524	-	GO:0006397:mRNA processing;GO:0008380:RNA splicing;GO:0034477:U6 snRNA 3'-end processing	GO:0004518:nuclease activity	GO:0005634:nucleus
Pp03_22312-3p(vvi-miR2950-5p)	18785476	PRUPE_ppa025256mg	NCBI_Assembly:GCF_000346465.1	NW_006760324.1	-	22895201	22896826	1625	-	-	-	-
Pp03_22312-3p(vvi-miR2950-5p)	18785492	PRUPE_ppa003289mg	NCBI_Assembly:GCF_000346465.1	NW_006760324.1	-	23198695	23202687	3992	-	-	GO:0008168:methyltransferase activity	-
Pp03_22312-3p(vvi-miR2950-5p)	18785576	PRUPE_ppa015960mg	NCBI_Assembly:GCF_000346465.1	NW_006760324.1	+	24512182	24513587	1405	-	-	-	-
Pp03_22312-3p(vvi-miR2950-5p)	18785615	PRUPE_ppa019691mg	NCBI_Assembly:GCF_000346465.1	NW_006760324.1	+	24308594	24311131	2537	-	GO:0030245:cellulose catabolic process	GO:0008810:cellulase activity	-
Pp03_22312-3p(vvi-miR2950-5p)	18785640	PRUPE_ppa015318mg	NCBI_Assembly:GCF_000346465.1	NW_006760324.1	-	25694188	25695235	1047	-	-	-	-

Pp03_22312-3p(vvi-miR2950-5p)	18785669	PRUPE_ppa025386mg	NCBI_Assembly:GCF_000346465.1	NW_006760324.1	-		12828889	12829686	797	-	GO:0006351:transcription, DNA-templated;GO:0006355:regulation of transcription, DNA-templated	GO:0003677:DNA binding	GO:0005634:nucleus
Pp03_22312-3p(vvi-miR2950-5p)	18785685	PRUPE_ppa001015mg	NCBI_Assembly:GCF_000346465.1	NW_006760324.1	+		24515516	24519029	3513	pper04626:Plant-pathogen interaction	-	GO:0043531:ADP binding	-
Pp03_22312-3p(vvi-miR2950-5p)	18785877	PRUPE_ppa026875mg	NCBI_Assembly:GCF_000346465.1	NW_006760324.1	+		25066253	25068375	2122	-	-	GO:0004497:monooxygenase activity;GO:0005506:iron ion binding;GO:0016705:oxidoreductase activity, acting on paired donors, with incorporation or reduction of molecular oxygen;GO:0020037:heme binding	-
Pp03_22312-3p(vvi-miR2950-5p)	18785978	PRUPE_ppa005854mg	NCBI_Assembly:GCF_000346465.1	NW_006760324.1	+		14474641	14480704	6063	pper03008:Ribosome biogenesis in eukaryotes	-	GO:0005525:GTP binding;GO:0015093:ferrous iron transmembrane transporter activity	GO:0016021:integral component of membrane

Pp03_22312-3p(vvi-miR2950-5p)	18786174	PRUPE_ppa008820mg	NCBI_Assembly:GCF_000346465.1	NW_006760324.1	-	23442793	23444082	1289	pper00360:Phenylalanine metabolism;pper01100:Metabolic pathways;pper01110:Biosynthesis of secondary metabolites;pper00940:Phenylpropanoid biosynthesis	GO:0006979:response to oxidative stress	GO:0004601:peroxidase activity;GO:0020037:heme binding;GO:0046872:metal ion binding	GO:0005576:extracellular region
Pp03_22312-3p(vvi-miR2950-5p)	18786222	PRUPE_ppa017818mg	NCBI_Assembly:GCF_000346465.1	NW_006760324.1	+	6044926	6046873	1947	pper03015:mRNA surveillance pathway	GO:0000491:small nucleolar ribonucleoprotein complex assembly	-	GO:0005634:nucleus;GO:0048046:apoplast
Pp03_22312-3p(vvi-miR2950-5p)	18786274	PRUPE_ppa014956mg	NCBI_Assembly:GCF_000346465.1	NW_006760324.1	+	335705	336052	347	-	-	-	-
Pp03_22312-3p(vvi-miR2950-5p)	18786301	PRUPE_ppa011542mg	NCBI_Assembly:GCF_000346465.1	NW_006760324.1	+	15084919	15086434	1515	pper01100:Metabolic pathways;pper00230:Purine metabolism;pper00240:Pyrimidine metabolism;pper03020:RNA polymerase	GO:0006351:transcription, DNA-templated	GO:0003677:DNA binding;GO:0003899:DNA-directed RNA polymerase activity	GO:0005634:nucleus
Pp03_22312-3p(vvi-miR2950-5p)	18786438	PRUPE_ppa016315mg	NCBI_Assembly:GCF_000346465.1	NW_006760324.1	-	21490811	21491864	1053	-	-	GO:0004672:protein kinase activity;GO:0005524:ATP binding	-

Pp03_22312-3p(vvi-miR2950-5p)	18786535	PRUPE_ppa005463mg	NCBI_Assembly:GCF_000346465.1	NW_006760324.1	-	26045761	26050063	4302	-	-	GO:0015098:molybdate ion transmembrane transporter activity	GO:0005768:endosome;GO:0005802:trans-Golgi network;GO:0005886:plasma membrane;GO:0016021:integral component of membrane
Pp03_22312-3p(vvi-miR2950-5p)	18786676	PRUPE_ppa020295mg	NCBI_Assembly:GCF_000346465.1	NW_006760324.1	+	20002942	20006529	3587	-	-	GO:0008234:cysteine-type peptidase activity	-
Pp03_22312-3p(vvi-miR2950-5p)	18786755	PRUPE_ppa022401mg	NCBI_Assembly:GCF_000346465.1	NW_006760324.1	-	26511661	26514829	3168	pper00130:Ubiquinone and other terpenoid-quinone biosynthesis;pper00360:Phenylalanine metabolism;pper01100:Metabolic pathways;pper01110:Biosynthesis of secondary metabolites;pper00940:Phenylpropanoid biosynthesis	GO:0009718:anthocyanin-containing compound biosynthetic process;GO:0009744:response to sucrose;GO:0010224:response to UV-B;GO:0010584:pollen exine formation	GO:0003824:catalytic activity	-

Pp03_22312-3p(vvi-miR2950-5p)	18786757	PRUPE_ppa020232mg	NCBI_Assembly:GCF_000346465.1	NW_006760324.1	+	22083684	22084931	1247	-	GO:0006355:regulation of transcription, DNA-templated	GO:0003677:DNA binding	-
Pp03_22312-3p(vvi-miR2950-5p)	18786922	PRUPE_ppa015047mg	NCBI_Assembly:GCF_000346465.1	NW_006760324.1	+	15017215	15017862	647	-	-	-	-
Pp03_22312-3p(vvi-miR2950-5p)	18786936	PRUPE_ppa001523mg	NCBI_Assembly:GCF_000346465.1	NW_006760324.1	-	22885615	22892733	7118	pper03013:RNA transport	GO:0000226:microtubule cytoskeleton organization;GO:0000911:cytokinesis by cell plate formation;GO:0006611:protein export from nucleus;GO:0009627:systemic acquired resistance	-	GO:0005643:nuclear pore
Pp03_22312-3p(vvi-miR2950-5p)	18786940	PRUPE_ppa021878mg	NCBI_Assembly:GCF_000346465.1	NW_006760324.1	+	16220203	16221171	968	-	-	-	GO:0009507:chloroplast
Pp03_22312-3p(vvi-miR2950-5p)	18786945	PRUPE_ppa022909mg	NCBI_Assembly:GCF_000346465.1	NW_006760324.1	-	12351376	12352068	692	-	-	-	-
Pp03_22312-3p(vvi-miR2950-5p)	18786987	PRUPE_ppa018253mg	NCBI_Assembly:GCF_000346465.1	NW_006760324.1	+	11940876	11941307	431	-	-	GO:0004672:protein kinase activity;GO:0005524:ATP binding	-
Pp03_22312-3p(vvi-miR2950-5p)	18786993	PRUPE_ppa006134mg	NCBI_Assembly:GCF_000346465.1	NW_006760324.1	+	9814855	9817780	2925	-	-	-	-
Pp03_22312-3p(vvi-miR2950-5p)	18787152	PRUPE_ppa024599mg	NCBI_Assembly:GCF_000346465.1	NW_006760324.1	+	22298713	22299003	290	-	-	-	-

Pp03_22312-3p(vvi-miR2950-5p)	18787156	PRUPE_ppa022998mg	NCBI_Assembly:GCF_000346465.1	NW_006760324.1	-		11569970	11572643	2673	-	GO:0006221:pyrimidine nucleotide biosynthetic process	GO:0003682:chromatin binding;GO:0033862:UMP kinase activity	GO:0005737:cytoplasm
Pp03_22312-3p(vvi-miR2950-5p)	18787161	PRUPE_ppa011033mg	NCBI_Assembly:GCF_000346465.1	NW_006760324.1	+		17180597	17182696	2099	pper04130:SNARE interactions in vesicular transport	GO:0006810:transport;GO:0061025:membrane fusion	-	GO:0005794:Golgi apparatus
Pp03_22312-3p(vvi-miR2950-5p)	18787313	PRUPE_ppa009190mg	NCBI_Assembly:GCF_000346465.1	NW_006760324.1	-		24964812	24968070	3258	-	-	-	-
Pp03_22312-3p(vvi-miR2950-5p)	18787347	PRUPE_ppa026020mg	NCBI_Assembly:GCF_000346465.1	NW_006760324.1	-		7416374	7416649	275	-	-	-	-
Pp03_22312-3p(vvi-miR2950-5p)	18787487	PRUPE_ppa025304mg	NCBI_Assembly:GCF_000346465.1	NW_006760324.1	+		17981418	17981906	488	-	-	-	-

Pp03_22312-3p(vvi-miR2950-5p)	18788585	PRUPE_ppa003324mg	NCBI_Assembly:GCF_000346465.1	NW_006760385.1	+	31755342	31759540	4198	-	GO:0007062:sister chromatid cohesion;GO:0007131:reciprocal meiotic recombination;GO:0009630:gravitropism;GO:0009887:organ morphogenesis;GO:0009888:tissue development;GO:0010228:vegetative to reproductive phase transition of meristem;GO:0010359:regulation of anion channel activity;GO:0010413:glucuronoxylan metabolic process;GO:0010638:positive regulation of organelle organization;GO:0016926:protein desumoylatio	-	-
Pp03_22312-3p(vvi-miR2950-5p)	18788681	PRUPE_ppa022547mg	NCBI_Assembly:GCF_000346465.1	NW_006760385.1	+	45400109	45400312	203	-	GO:0006412:translation	GO:0003735:structural constituent of ribosome;GO:0019843:rRNA binding	GO:0005840:ribosome;GO:0009507:chloroplast

Pp03_22312-3p(vvi-miR2950-5p)	18788705	PRUPE_ppa008416mg	NCBI_Assembly:GCF_000346465.1	NW_006760385.1	-	11605772	11609287	3515	-	GO:0046496:nicotinamide nucleotide metabolic process;GO:0048573:photoperiodism, flowering	GO:0005524:ATP binding;GO:0047453:ATP-dependent NAD(P)H-hydrate dehydratase activity;GO:0052855:ADP-dependent NAD(P)H-hydrate dehydratase activity	-
Pp03_22312-3p(vvi-miR2950-5p)	18788716	PRUPE_ppa009817mg	NCBI_Assembly:GCF_000346465.1	NW_006760385.1	-	6011392	6016622	5230	-	-	GO:0008308:voltage-gated anion channel activity	GO:0005741:mitochondrial outer membrane;GO:0005886:plasma membrane
Pp03_22312-3p(vvi-miR2950-5p)	18788863	PRUPE_ppa017521mg	NCBI_Assembly:GCF_000346465.1	NW_006760385.1	+	11010187	11010540	353	-	GO:0009860:pollen tube growth	-	-
Pp03_22312-3p(vvi-miR2950-5p)	18788881	PRUPE_ppa019091mg	NCBI_Assembly:GCF_000346465.1	NW_006760385.1	-	40513520	40515124	1604	-	GO:0006817:phosphate ion transport	GO:0005315:inorganic phosphate transmembrane transporter activity	GO:0016021:integral component of membrane
Pp03_22312-3p(vvi-miR2950-5p)	18788914	PRUPE_ppa003357mg	NCBI_Assembly:GCF_000346465.1	NW_006760385.1	-	35166169	35170745	4576	pper04144:Endocytosis	GO:0006623:protein targeting to vacuole	-	GO:0005884:actin filament;GO:0009579:thylakoid

Pp03_22312-3p(vvi-miR2950-5p)	18788967	PRUPE_ppa017854mg	NCBI_Assembly:GCF_000346465.1	NW_006760385.1	-		31259753	31260213	460	pper01100:Metabolic pathways;pper00195:Photosynthesis	GO:0015979:photosynthesis	GO:0005509:calcium ion binding	GO:0009654:photosystem II oxygen evolving complex;GO:0019898:extrinsic component of membrane
Pp03_22312-3p(vvi-miR2950-5p)	18788999	PRUPE_ppa002630mg	NCBI_Assembly:GCF_000346465.1	NW_006760385.1	+		30828156	30832580	4424	-	-	-	GO:0005829:cytosol
Pp03_22312-3p(vvi-miR2950-5p)	18789096	PRUPE_ppa003234mg	NCBI_Assembly:GCF_000346465.1	NW_006760385.1	-		25365053	25369429	4376	-	GO:0045454:cell redox homeostasis	-	GO:0005623:cell
Pp03_22312-3p(vvi-miR2950-5p)	18789178	PRUPE_ppa009461mg	NCBI_Assembly:GCF_000346465.1	NW_006760385.1	+		46825567	46827433	1866	-	-	-	-
Pp03_22312-3p(vvi-miR2950-5p)	18789210	PRUPE_ppa022157mg	NCBI_Assembly:GCF_000346465.1	NW_006760385.1	-		234247	236385	2138	-	GO:0006979:response to oxidative stress;GO:0009651:response to salt stress;GO:0009737:response to abscisic acid;GO:0042775:mitochondrial ATP synthesis coupled electron transport	-	GO:0005739:mitochondrion

Pp03_22312-3p(vvi-miR2950-5p)	18789840	PRUPE_ppa011357mg	NCBI_Assembly:GCF_000346465.1	NW_006760385.1	-	3298459	3302077	3618	pper03013:RNA transport	GO:0009615:response to virus;GO:0050687:negative regulation of defense response to virus	GO:0000340:RNA 7-methylguanosine cap binding;GO:0003743:translation initiation factor activity	GO:0005634:nucleus;GO:0005829:cytosol;GO:0005845:mRNA cap binding complex

Pp03_22312-3p(vvi-miR2950-5p)	18789908	PRUPE_ppa024137mg	NCBI_Assembly:GCF_000346465.1	NW_006760385.1	-	11648843	11652144	3301	-	GO:0000165:MAPK cascade;GO:0006355:regulation of transcription, DNA-templated;GO:0006612:protein targeting to membrane;GO:0009561:megagametogenesis;GO:0009617:response to bacterium;GO:0009862:systemic acquired resistance, salicylic acid mediated signaling pathway;GO:0009867:jasmonic acid mediated signaling pathway;GO:0010310:regulation of hydrogen peroxide metabolic process;GO:0010363:regulation of	GO:0004842:ubiquitin-protein transferase activity;GO:0008270:zinc ion binding	GO:0005622:intracellular;GO:0016020:membrane
Pp03_22312-3p(vvi-miR2950-5p)	18789955	PRUPE_ppa007754mg	NCBI_Assembly:GCF_000346465.1	NW_006760385.1	+	46608599	46610648	2049	-	-	-	GO:0005634:nucleus

Pp03_22312-3p(vvi-miR2950-5p)	18790061	PRUPE_ppa012646mg	NCBI_Assembly:GCF_000346465.1	NW_006760385.1	+	9565714	9566578	864	-	GO:0006952:defense response;GO:0009607:response to biotic stimulus	-	-
Pp03_22312-3p(vvi-miR2950-5p)	18790201	PRUPE_ppa020938mg	NCBI_Assembly:GCF_000346465.1	NW_006760385.1	+	28444962	28448597	3635	pper03018:RNA degradation	GO:0000023:maltose metabolic process;GO:0006655:phosphatidylglycerol biosynthetic process;GO:0016556:mRNA modification; GO:0019252:starch biosynthetic process;GO:0019288:isopentenyl diphosphate biosynthetic process, methylerythritol 4-phosphate pathway;GO:0042026:protein refolding;GO:0043085:positive regulation of catalytic activity	GO:0005524:ATP binding	GO:0009941:chloroplast envelope
Pp03_22312-3p(vvi-miR2950-5p)	18790223	PRUPE_ppa013147mg	NCBI_Assembly:GCF_000346465.1	NW_006760385.1	+	22783952	22786797	2845	-	-	-	-

Pp03_22312-3p(vvi-miR2950-5p)	18790259	PRUPE_ppa011652mg	NCBI_Assembly:GCF_000346465.1	NW_006760385.1	-	5589815	5591633	1818	-	GO:0030003:cellular cation homeostasis;GO:0070838:divalent metal ion transport	-	GO:0005623:cell
Pp03_22312-3p(vvi-miR2950-5p)	18790372	PRUPE_ppa000670mg	NCBI_Assembly:GCF_000346465.1	NW_006760385.1	+	4690362	4703126	12764	-	-	GO:0005388:calcium-transporting ATPase activity;GO:0005524:ATP binding;GO:0046872:metal ion binding	GO:0016021:integral component of membrane
Pp03_22312-3p(vvi-miR2950-5p)	18790421	PRUPE_ppa002112mg	NCBI_Assembly:GCF_000346465.1	NW_006760385.1	-	35508120	35510862	2742	-	-	-	GO:0005886:plasma membrane
Pp03_22312-3p(vvi-miR2950-5p)	18790580	PRUPE_ppa005918mg	NCBI_Assembly:GCF_000346465.1	NW_006760385.1	-	45900438	45902646	2208	-	-	GO:0004497:monooxygenase activity;GO:0005506:iron ion binding;GO:0016705:oxidoreductase activity, acting on paired donors, with incorporation or reduction of molecular oxygen;GO:0020037:heme binding	-
Pp03_22312-3p(vvi-miR2950-5p)	18790683	PRUPE_ppa026590mg	NCBI_Assembly:GCF_000346465.1	NW_006760385.1	-	27601510	27603368	1858	-	-	-	-

Pp03_22312-3p(vvi-miR2950-5p)	18790783	PRUPE_ppa013802mg	NCBI_Assembly:GCF_000346465.1	NW_006760385.1	+	4073323	4074211	888	-	-	-	-	
Pp03_22312-3p(vvi-miR2950-5p)	18790968	PRUPE_ppa017218mg	NCBI_Assembly:GCF_000346465.1	NW_006760385.1	-	3980038	3981176	1138	pper00480:Glutathione metabolism	-	-	-	
Pp03_22312-3p(vvi-miR2950-5p)	18790974	PRUPE_ppa014996mg	NCBI_Assembly:GCF_000346465.1	NW_006760385.1	+	10877248	10878796	1548	pper00360:Phenylalanine metabolism;pper01100:Metabolic pathways;pper01110:Biosynthesis of secondary metabolites;pper00940:Phenylpropanoid biosynthesis	GO:0006979:response to oxidative stress	GO:0004601:peroxidase activity;GO:0020037:heme binding;GO:0046872:metal ion binding	-	
Pp03_22312-3p(vvi-miR2950-5p)	18791046	PRUPE_ppa017359mg	NCBI_Assembly:GCF_000346465.1	NW_006760385.1	+	10249086	10251148	2062	-	-	-	-	
Pp03_22312-3p(vvi-miR2950-5p)	18791240	PRUPE_ppa012125mg	NCBI_Assembly:GCF_000346465.1	NW_006760385.1	-	44007596	44010935	3339	-	GO:0007030:Golgi organization;GO:0009306:protein secretion;GO:0009826:unidimensional cell growth	-	GO:0000139:Golgi membrane;GO:0005802:trans-Golgi network;GO:0016021:integral component of membrane	
Pp03_22312-3p(vvi-miR2950-5p)	18791310	PRUPE_ppa017884mg	NCBI_Assembly:GCF_000346465.1	NW_006760385.1	-	16351689	16352695	1006	-	-	GO:0003677:DNA binding	-	
Pp03_22312-3p(vvi-miR2950-5p)	18791591	PRUPE_ppa011747mg	NCBI_Assembly:GCF_000346465.1	NW_006760385.1	-	1492603	1493684	1081	-	-	-	-	
Pp03_22312-3p(vvi-miR2950-5p)	18791664	PRUPE_ppa019422mg	NCBI_Assembly:GCF_000346465.1	NW_006760385.1	+	299037	300453	1416	-	-	-	-	

Pp03_22312-3p(vvi-miR2950-5p)	18791724	PRUPE_ppa009629mg	NCBI_Assembly:GCF_000346465.1	NW_006760385.1	-	3012284	3015093	2809	-	GO:0009658:chloroplast organization;GO:0010193:response to ozone;GO:0042742:defense response to bacterium	-	GO:0005634:nucleus;GO:0005886:plasma membrane;GO:0009535:chloroplast thylakoid membrane;GO:0009941:chloroplast envelope;GO:0031977:thylakoid lumen
Pp03_22312-3p(vvi-miR2950-5p)	18791771	PRUPE_ppa026343mg	NCBI_Assembly:GCF_000346465.1	NW_006760385.1	-	44510454	44511331	877	-	-	GO:0022857:transmembrane transporter activity	GO:0016021:integral component of membrane
Pp03_22312-3p(vvi-miR2950-5p)	18792007	PRUPE_ppb024585mg	NCBI_Assembly:GCF_000346465.1	NW_006760385.1	-	28489245	28489565	320	-	-	-	-
Pp03_22312-3p(vvi-miR2950-5p)	18792191	PRUPE_ppa016212mg	NCBI_Assembly:GCF_000346465.1	NW_006760385.1	-	38578459	38578743	284	-	-	-	-
Pp03_22312-3p(vvi-miR2950-5p)	18792196	PRUPE_ppa026474mg	NCBI_Assembly:GCF_000346465.1	NW_006760385.1	-	36674308	36677849	3541	-	GO:0006364:rRNA processing	GO:0004222:metalloendopeptidase activity	-
Pp03_22312-3p(vvi-miR2950-5p)	18792279	PRUPE_ppa000894mg	NCBI_Assembly:GCF_000346465.1	NW_006760385.1	+	34136647	34143640	6993	pper03040:Spliceosome	GO:0006396:RNA processing	GO:0000166:nucleotide binding;GO:0003723:RNA binding	-
Pp03_22312-3p(vvi-miR2950-5p)	18792452	PRUPE_ppa002461mg	NCBI_Assembly:GCF_000346465.1	NW_006760385.1	-	39643034	39647465	4431	-	-	GO:0008270:zinc ion binding	-
Pp03_22312-3p(vvi-miR2950-5p)	18792642	PRUPE_ppa024126mg	NCBI_Assembly:GCF_000346465.1	NW_006760385.1	-	40996858	40999127	2269	-	-	GO:0003676:nucleic acid binding	-

Pp03_22312-5p	18766027	PRUPE_ppa017620mg	NCBI_Assembly:GCF_000346465.1	NW_006760186.1	-	1537056	1539907	2851	-	-	GO:0008234:cysteine-type peptidase activity	-
Pp03_22312-5p	18766044	PRUPE_ppa026620mg	NCBI_Assembly:GCF_000346465.1	NW_006760186.1	-	123114	124493	1379	-	-	-	-
Pp03_22312-5p	18766072	PRUPE_ppa016792mg	NCBI_Assembly:GCF_000346465.1	NW_006760186.1	+	670573	671856	1283	-	-	GO:0016209:antioxidant activity;GO:0016491:oxidoreductase activity	-
Pp03_22312-5p	18766073	PRUPE_ppa018583mg	NCBI_Assembly:GCF_000346465.1	NW_006760186.1	-	788033	791067	3034	-	-	-	-
Pp03_22312-5p	18766099	PRUPE_ppa021870mg	NCBI_Assembly:GCF_000346465.1	NW_006760186.1	+	1328652	1330909	2257	-	-	GO:0008270:zinc ion binding	-
Pp03_22312-5p	18766103	PRUPE_ppa014632mg	NCBI_Assembly:GCF_000346465.1	NW_006760186.1	-	1936980	1937288	308	-	-	-	-
Pp03_22312-5p	18766152	PRUPE_ppa025265mg	NCBI_Assembly:GCF_000346465.1	NW_006760194.1	+	13727715	13731854	4139	-	-	GO:0043531:ADP binding	-
Pp03_22312-5p	18766164	PRUPE_ppa016093mg	NCBI_Assembly:GCF_000346465.1	NW_006760194.1	+	5983601	5988590	4989	-	-	GO:0003676:nucleic acid binding	-
Pp03_22312-5p	18766165	PRUPE_ppa012386mg	NCBI_Assembly:GCF_000346465.1	NW_006760194.1	+	6087466	6089524	2058	-	-	-	-
Pp03_22312-5p	18766193	PRUPE_ppa004587mg	NCBI_Assembly:GCF_000346465.1	NW_006760194.1	-	17075308	17079258	3950	pper04075:Plant hormone signal transduction	-	GO:0004672:protein kinase activity;GO:0005524:ATP binding	-
Pp03_22312-5p	18766205	PRUPE_ppb019151mg	NCBI_Assembly:GCF_000346465.1	NW_006760194.1	+	21077971	21079017	1046	pper01100:Metabolic pathways;pper00430:Taurine and hypotaurine metabolism	-	-	-

Pp03_22312-5p	18766218	PRUPE_ppa008342mg	NCBI_Assembly:GCF_000346465.1	NW_006760194.1	-	368172	369629	1457	-	-	-	-
Pp03_22312-5p	18766243	PRUPE_ppa015782mg	NCBI_Assembly:GCF_000346465.1	NW_006760194.1	+	8079782	8080078	296	-	-	-	-
Pp03_22312-5p	18766250	PRUPE_ppa025882mg	NCBI_Assembly:GCF_000346465.1	NW_006760194.1	+	899866	901794	1928	-	-	GO:0016706:oxidoreductase activity, acting on paired donors, with incorporation or reduction of molecular oxygen, 2-oxoglutarate as one donor, and incorporation of one atom each of oxygen into both donors	-
Pp03_22312-5p	18766283	PRUPE_ppa013630mg	NCBI_Assembly:GCF_000346465.1	NW_006760194.1	+	11794059	11794582	523	-	-	-	-
Pp03_22312-5p	18766376	PRUPE_ppa000433mg	NCBI_Assembly:GCF_000346465.1	NW_006760194.1	+	17301185	17311573	10388	-	-	GO:0005509:calcium ion binding	-
Pp03_22312-5p	18766405	PRUPE_ppa025270mg	NCBI_Assembly:GCF_000346465.1	NW_006760194.1	+	12715012	12717959	2947	pper03040:Spliceosome	-	GO:0003676:nucleic acid binding;GO:0005524:ATP binding;GO:0008026:ATP-dependent helicase activity	-
Pp03_22312-5p	18766412	PRUPE_ppa015182mg	NCBI_Assembly:GCF_000346465.1	NW_006760194.1	+	18751736	18752747	1011	pper00480:Glutathione metabolism	-	-	-

Pp03_22312-5p	18766432	PRUPE_ppa011316mg	NCBI_Assembly:GCF_000346465.1	NW_006760194.1	+	1987209	1991960	4751	-		GO:0010027:thylakoid membrane organization	GO:0016209:antioxidant activity;GO:0016491:oxidoreductase activity	GO:0009507:chloroplast
Pp03_22312-5p	18766459	PRUPE_ppa022491mg	NCBI_Assembly:GCF_000346465.1	NW_006760194.1	+	19889697	19893018	3321	-		GO:0006261:DNA-dependent DNA replication;GO:0009555:pollen development;GO:0009790:embryo development;GO:0031146:SCF-dependent proteasomal ubiquitin-dependent protein catabolic process;GO:0048316:seed development;GO:0055047:generative cell mitosis	-	GO:0001673:male germ cell nucleus;GO:0019005:SCF ubiquitin ligase complex
Pp03_22312-5p	18766516	PRUPE_ppb025199mg	NCBI_Assembly:GCF_000346465.1	NW_006760194.1	-	6351595	6352525	930	-		-	-	-
Pp03_22312-5p	18766605	PRUPE_ppb025593mg	NCBI_Assembly:GCF_000346465.1	NW_006760194.1	-	17184516	17186988	2472	-		-	-	-
Pp03_22312-5p	18766614	PRUPE_ppa007168mg	NCBI_Assembly:GCF_000346465.1	NW_006760194.1	+	16214960	16217451	2491	-		-	-	-
Pp03_22312-5p	18766635	PRUPE_ppa017223mg	NCBI_Assembly:GCF_000346465.1	NW_006760194.1	-	3304086	3304705	619	-		-	GO:0003676:nucleic acid binding	-

Pp03_22312-5p	18766703	PRUPE_ppa004400mg	NCBI_Assembly:GCF_000346465.1	NW_006760194.1	-	16521942	16526241	4299	-	-	-	-
Pp03_22312-5p	18766718	PRUPE_ppa026432mg	NCBI_Assembly:GCF_000346465.1	NW_006760194.1	+	8934725	8936059	1334	-	-	-	-
Pp03_22312-5p	18766758	PRUPE_ppa010199mg	NCBI_Assembly:GCF_000346465.1	NW_006760194.1	-	21588154	21589338	1184	-	-	-	-
Pp03_22312-5p	18766789	PRUPE_ppa010750mg	NCBI_Assembly:GCF_000346465.1	NW_006760194.1	-	17831289	17833782	2493	-	-	-	-
Pp03_22312-5p	18766914	PRUPE_ppa021698mg	NCBI_Assembly:GCF_000346465.1	NW_006760194.1	-	1895988	1898141	2153	-	-	GO:0017089:glycolipid transporter activity;GO:0051861:glycolipid binding	GO:0005737:cytoplasm
Pp03_22312-5p	18766925	PRUPE_ppa004418mg	NCBI_Assembly:GCF_000346465.1	NW_006760194.1	+	19437659	19439804	2145	-	-	GO:0045735:nutrient reservoir activity	-
Pp03_22312-5p	18766957	PRUPE_ppa004061mg	NCBI_Assembly:GCF_000346465.1	NW_006760194.1	+	6465236	6469033	3797	pper01100:Metabolic pathways;pper00230:Purine metabolism;pper00240:Pyrimidine metabolism;pper03030:DNA replication;pper03420:Nucleotide excision repair;pper03410:Base excision repair	GO:0006261:DNA-dependent DNA replication	GO:0003677:DNA binding;GO:0003887:DNA-directed DNA polymerase activity	GO:0005634:nucleus
Pp03_22312-5p	18767019	PRUPE_ppa018280mg	NCBI_Assembly:GCF_000346465.1	NW_006760194.1	-	18926348	18926578	230	-	-	-	-

Pp03_22312-5p	18767082	PRUPE_ppa022063mg	NCBI_Assembly:GCF_000346465.1	NW_006760194.1	+	16245243	16245809	566	-	-	-	-
Pp03_22312-5p	18767093	PRUPE_ppb022444mg	NCBI_Assembly:GCF_000346465.1	NW_006760194.1	-	17622657	17626416	3759	-	-	GO:0043531: ADP binding	-
Pp03_22312-5p	18767124	PRUPE_ppa009911mg	NCBI_Assembly:GCF_000346465.1	NW_006760194.1	-	16124715	16127302	2587	-	GO:0006621: protein retention in ER lumen	GO:0046923: ER retention sequence binding	GO:0005783: endoplasmic reticulum;GO:0016021:integral component of membrane
Pp03_22312-5p	18767127	PRUPE_ppa014001mg	NCBI_Assembly:GCF_000346465.1	NW_006760194.1	-	15909644	15910344	700	-	-	-	GO:0005576: extracellular region
Pp03_22312-5p	18767145	PRUPE_ppa021884mg	NCBI_Assembly:GCF_000346465.1	NW_006760194.1	+	5107996	5108598	602	pper01100:Metabolic pathways;pper01110:Biosynthesis of secondary metabolites;pper00330:Arginine and proline metabolism;pper00480:Glutathione metabolism	GO:0006596: polyamine biosynthetic process	GO:0003824: catalytic activity	-
Pp03_22312-5p	18767214	PRUPE_ppa023397mg	NCBI_Assembly:GCF_000346465.1	NW_006760194.1	-	14277477	14278923	1446	pper01100:Metabolic pathways;pper00600:Sphingolipid metabolism	-	-	GO:0005783: endoplasmic reticulum;GO:0016021:integral component of membrane

Pp03_22312-5p	18767223	PRUPE_ppa002723mg	NCBI_Assembly:GCF_000346465.1	NW_006760194.1	-	21434978	21439332	4354	pper01100:Metabolic pathways;pper00500:Starch and sucrose metabolism	GO:0001666:response to hypoxia;GO:0005982:starch metabolic process;GO:0005985:sucrose metabolic process;GO:0010431:seed maturation;GO:0019375:galactolipid biosynthetic process	GO:0016157:sucrose synthase activity	-
Pp03_22312-5p	18767238	PRUPE_ppa000339mg	NCBI_Assembly:GCF_000346465.1	NW_006760194.1	+	8519548	8523359	3811	pper03040:Spliceosome	-	-	GO:0009507:chloroplast
Pp03_22312-5p	18767293	PRUPE_ppa021443mg	NCBI_Assembly:GCF_000346465.1	NW_006760194.1	+	2396085	2398345	2260	-	-	GO:0004497:monooxygenase activity;GO:0005506:iron ion binding;GO:0016705:oxidoreductase activity, acting on paired donors, with incorporation or reduction of molecular oxygen;GO:0020037:heme binding	-

Pp03_22312-5p	18767329	PRUPE_ppa004882m2g	NCBI_Assembly:GCF_000346465.1	NW_006760194.1	-	12144846	12149809	4963	pper00970:Aminoacyl-tRNA biosynthesis	GO:0006432:phenylalanyl-tRNA aminoacylation	GO:0000049:tRNA binding;GO:0004826:phenylalanine-tRNA ligase activity;GO:0005524:ATP binding	GO:0005737:cytoplasm
Pp03_22312-5p	18767355	PRUPE_ppa001458mg	NCBI_Assembly:GCF_000346465.1	NW_006760194.1	-	19055065	19063047	7982	-	GO:0006397:mRNA processing	-	GO:0005634:nucleus
Pp03_22312-5p	18767442	PRUPE_ppa016824mg	NCBI_Assembly:GCF_000346465.1	NW_006760194.1	+	18112081	18113972	1891	-	GO:0006621:protein retention in ER lumen	GO:0046923:ER retention sequence binding	GO:0005783:endoplasmic reticulum;GO:0016021:integral component of membrane
Pp03_22312-5p	18767460	PRUPE_ppa022297mg	NCBI_Assembly:GCF_000346465.1	NW_006760194.1	-	628896	631354	2458	-	-	GO:0008168:methyltransferase activity	-
Pp03_22312-5p	18767556	PRUPE_ppa003272mg	NCBI_Assembly:GCF_000346465.1	NW_006760194.1	-	15971865	15974528	2663	-	-	GO:0003676:nucleic acid binding;GO:0008270:zinc ion binding	-
Pp03_22312-5p	18767561	PRUPE_ppa024894mg	NCBI_Assembly:GCF_000346465.1	NW_006760194.1	+	13601670	13604710	3040	-	GO:0006396:RNA processing	GO:0000166:nucleotide binding;GO:0003723:RNA binding	GO:0005634:nucleus;GO:0030529:ribonucleoprotein complex

Pp03_22312-5p	18767568	PRUPE_ppb020445mg	NCBI_Assembly:GCF_000346465.1	NW_006760194.1	-	17559294	17560265	971	-	-	GO:0003700:sequence-specific DNA binding transcription factor activity;GO:0043565:sequence-specific DNA binding	-
Pp03_22312-5p	18767585	PRUPE_ppa008730mg	NCBI_Assembly:GCF_000346465.1	NW_006760194.1	+	21465216	21466550	1334	-	GO:0006351:transcription, DNA-templated	GO:0003677:DNA binding;GO:0003700:sequence-specific DNA binding transcription factor activity	GO:0005634:nucleus
Pp03_22312-5p	18767588	PRUPE_ppb012210mg	NCBI_Assembly:GCF_000346465.1	NW_006760194.1	+	10667967	10668135	168	-	-	-	-
Pp03_22312-5p	18768797	PRUPE_ppa021730mg	NCBI_Assembly:GCF_000346465.1	NW_006760194.1	-	18496564	18497659	1095	-	-	GO:0003676:nucleic acid binding	-
Pp03_22312-5p	18768809	PRUPE_ppa015305mg	NCBI_Assembly:GCF_000346465.1	NW_006760194.1	+	16082290	16083229	939	-	-	-	GO:0016021:integral component of membrane
Pp03_22312-5p	18768834	PRUPE_ppa014095mg	NCBI_Assembly:GCF_000346465.1	NW_006760194.1	+	14853771	14854689	918	-	-	-	-
Pp03_22312-5p	18768866	PRUPE_ppa001002mg	NCBI_Assembly:GCF_000346465.1	NW_006760194.1	+	355877	367230	11353	pper04144:Endocytosis	-	GO:0003924:GTPase activity;GO:0005525:GTP binding	-
Pp03_22312-5p	18768981	PRUPE_ppa002822mg	NCBI_Assembly:GCF_000346465.1	NW_006760194.1	-	15309204	15315339	6135	-	-	GO:0005509:calcium ion binding	-

Pp03_22312-5p	18768991	PRUPE_ppa017362mg	NCBI_Assembly:GCF_000346465.1	NW_006760194.1	+		13493135	13494701	1566	-	-	-	-
Pp03_22312-5p	18769042	PRUPE_ppa010444mg	NCBI_Assembly:GCF_000346465.1	NW_006760201.1	+		17672590	17674565	1975	pper03050:Proteasome	GO:0006511:ubiquitin-dependent protein catabolic process	GO:0004298:threonine-type endopeptidase activity	GO:0005634:nucleus;GO:0005737:cytoplasm;GO:0019773:proteasome core complex, alpha-subunit complex
Pp03_22312-5p	18769062	PRUPE_ppa023344mg	NCBI_Assembly:GCF_000346465.1	NW_006760201.1	-		21400061	21401836	1775	-	-	-	-
Pp03_22312-5p	18769212	PRUPE_ppa001346mg	NCBI_Assembly:GCF_000346465.1	NW_006760201.1	+		17073933	17076479	2546	-	-	GO:0043531:ADP binding	-
Pp03_22312-5p	18769221	PRUPE_ppa005721mg	NCBI_Assembly:GCF_000346465.1	NW_006760201.1	+		12806403	12809812	3409	-	-	-	-
Pp03_22312-5p	18769243	PRUPE_ppa004707mg	NCBI_Assembly:GCF_000346465.1	NW_006760201.1	+		19718523	19720073	1550	pper01100:Metabolic pathways;pper00740:Riboflavin metabolism;pper00230:Purine metabolism;pper00500:Starch and sucrose metabolism;pper00770:Pantothenate and CoA biosynthesis;pper00760:Nicotinate and nicotinamide metabolism	GO:0000394:RNA splicing, via endonucleolytic cleavage and ligation;GO:0009086:methionine biosynthetic process	GO:0003824:catalytic activity	GO:0005773:vacuole

Pp03_22312-5p	18769293	PRUPE_ppa010251mg	NCBI_Assembly:GCF_000346465.1	NW_006760201.1	-	19786201	19788526	2325	pper01100:Metabolic pathways;pper00750:Vitamin B6 metabolism	GO:0051262:protein tetramerization	GO:0016791:phosphatase activity	-
Pp03_22312-5p	18769466	PRUPE_ppa012365mg	NCBI_Assembly:GCF_000346465.1	NW_006760201.1	+	10316103	10317965	1862	-	-	GO:0003677:DNA binding;GO:0008270:zinc ion binding	-
Pp03_22312-5p	18769474	PRUPE_ppa002711mg	NCBI_Assembly:GCF_000346465.1	NW_006760201.1	-	10102622	10109169	6547	-	-	GO:0003677:DNA binding;GO:0005524:ATP binding	-
Pp03_22312-5p	18769539	PRUPE_ppa006267mg	NCBI_Assembly:GCF_000346465.1	NW_006760201.1	-	20010351	20012863	2512	-	-	-	GO:0009505:plant-type cell wall
Pp03_22312-5p	18769545	PRUPE_ppa018687mg	NCBI_Assembly:GCF_000346465.1	NW_006760201.1	+	10529623	10532352	2729	-	-	GO:0004497:monooxygenase activity;GO:0005506:iron ion binding;GO:0016705:oxidoreductase activity, acting on paired donors, with incorporation or reduction of molecular oxygen;GO:0020037:heme binding	-
Pp03_22312-5p	18769556	PRUPE_ppa026210mg	NCBI_Assembly:GCF_000346465.1	NW_006760201.1	+	19078780	19079622	842	-	-	-	-
Pp03_22312-5p	18769618	PRUPE_ppa001284mg	NCBI_Assembly:GCF_000346465.1	NW_006760201.1	+	8960023	8968325	8302	pper03013:RNA transport	GO:0006810:transport	-	GO:0005643:nuclear pore

Pp03_22312-5p	18769703	PRUPE_ppa016738mg	NCBI_Assembly:GCF_000346465.1	NW_006760201.1	+		21868897	21869447	550	-	-	GO:0008270: zinc ion binding	-
Pp03_22312-5p	18769706	PRUPE_ppa019287mg	NCBI_Assembly:GCF_000346465.1	NW_006760201.1	-		20214947	20217992	3045	-	GO:0006355: regulation of transcription, DNA-templated	GO:0003690: double-stranded DNA binding	GO:0005739: mitochondrion
Pp03_22312-5p	18769714	PRUPE_ppa000360mg	NCBI_Assembly:GCF_000346465.1	NW_006760201.1	+		18822368	18829774	7406	-	-	-	-
Pp03_22312-5p	18769735	PRUPE_ppa007711mg	NCBI_Assembly:GCF_000346465.1	NW_006760201.1	-		22193637	22194715	1078	-	-	GO:0005345: purine nucleobase transmembrane transporter activity	GO:0016021: integral component of membrane
Pp03_22312-5p	18769845	PRUPE_ppa013262mg	NCBI_Assembly:GCF_000346465.1	NW_006760201.1	-		15242552	15244155	1603	-	GO:0006468: protein phosphorylation;GO:0046685:response to arsenic-containing substance	GO:0030611: arsenate reductase activity	GO:0005739: mitochondrion;GO:0009507:chloroplast
Pp03_22312-5p	18769865	PRUPE_ppa014587mg	NCBI_Assembly:GCF_000346465.1	NW_006760201.1	-		7313079	7313665	586	-	-	-	-
Pp03_22312-5p	18769881	PRUPE_ppa021795mg	NCBI_Assembly:GCF_000346465.1	NW_006760201.1	-		1037056	1040520	3464	-	-	-	-

Pp03_22312-5p	18769951	PRUPE_ppa023335mg	NCBI_Assembly:GCF_000346465.1	NW_006760201.1	+	19026328	19028081	1753	-	-	GO:0004497:monooxygenase activity;GO:0005506:iron ion binding;GO:0016705:oxidoreductase activity, acting on paired donors, with incorporation or reduction of molecular oxygen;GO:0020037:heme binding	-
Pp03_22312-5p	18770008	PRUPE_ppa026915mg	NCBI_Assembly:GCF_000346465.1	NW_006760201.1	+	6112449	6114105	1656	-	-	-	-
Pp03_22312-5p	18770142	PRUPE_ppa023290mg	NCBI_Assembly:GCF_000346465.1	NW_006760201.1	-	2513368	2513924	556	-	-	-	-
Pp03_22312-5p	18770207	PRUPE_ppa021998mg	NCBI_Assembly:GCF_000346465.1	NW_006760201.1	-	12229159	12229755	596	-	-	-	-
Pp03_22312-5p	18770340	PRUPE_ppa012070mg	NCBI_Assembly:GCF_000346465.1	NW_006760201.1	+	1025641	1028056	2415	pper04626:Plant-pathogen interaction;pper04070:Phosphatidylinositol signaling system	-	GO:0005509:calcium ion binding	-

Pp03_22312-5p	18770385	PRUPE_ppa015817mg	NCBI_Assembly:GCF_000346465.1	NW_006760201.1	+	14074262	14076067	1805	pper03040:Spliceosome;pper04141:Protein processing in endoplasmic reticulum;pper04144:Endocytosis	-	GO:0005524:ATP binding	-	
Pp03_22312-5p	18770432	PRUPE_ppa024423mg	NCBI_Assembly:GCF_000346465.1	NW_006760201.1	-	12263619	12264072	453	-	-	-	-	
Pp03_22312-5p	18770463	PRUPE_ppa019743mg	NCBI_Assembly:GCF_000346465.1	NW_006760201.1	-	15998866	15999719	853	-	-	GO:0003676:nucleic acid binding;GO:0008270:zinc ion binding	-	
Pp03_22312-5p	18770566	PRUPE_ppa009801mg	NCBI_Assembly:GCF_000346465.1	NW_006760201.1	-	2259168	2263493	4325	pper01110:Biosynthesis of secondary metabolites;pper00520:Amino sugar and nucleotide sugar metabolism	GO:0006499:N-terminal protein myristoylation;GO:0022900:electron transport chain;GO:0042732:D-xylose metabolic process	GO:0004128:cytochrome-b5 reductase activity, acting on NAD(P)H	GO:0005783:endoplasmic reticulum;GO:0005886:plasma membrane;GO:0009505:plant-type cell wall	
Pp03_22312-5p	18770640	PRUPE_ppb019068mg	NCBI_Assembly:GCF_000346465.1	NW_006760201.1	+	12162769	12163717	948	-	-	-	-	
Pp03_22312-5p	18770666	PRUPE_ppa004446mg	NCBI_Assembly:GCF_000346465.1	NW_006760201.1	+	19983905	19988064	4159	-	-	GO:0004222:metalloendopeptidase activity;GO:0046872:metal ion binding	-	
Pp03_22312-5p	18770739	PRUPE_ppa021376mg	NCBI_Assembly:GCF_000346465.1	NW_006760201.1	+	11071849	11073069	1220	-	GO:0035556:intracellular signal transduction	GO:0047134:protein-disulfide reductase activity	GO:0005622:intracellular	

Pp03_22312-5p	18770775	PRUPE_ppa000206mg	NCBI_Assembly:GCF_000346465.1	NW_006760201.1	-	21814608	21820314	5706	-	GO:0009644:response to high light intensity;GO:0042542:response to hydrogen peroxide	GO:0004712:protein serine/threonine/tyrosine kinase activity;GO:0005524:ATP binding	-
Pp03_22312-5p	18770799	PRUPE_ppa012368mg	NCBI_Assembly:GCF_000346465.1	NW_006760201.1	-	22026954	22028935	1981	-	-	-	-
Pp03_22312-5p	18770862	PRUPE_ppa000497m1g	NCBI_Assembly:GCF_000346465.1	NW_006760201.1	-	22686904	22699176	12272	-	-	GO:0003824:catalytic activity;GO:0046872:metal ion binding	-
Pp03_22312-5p	18771131	PRUPE_ppa001699mg	NCBI_Assembly:GCF_000346465.1	NW_006760201.1	-	21569248	21573115	3867	-	-	GO:0005247:voltage-gated chloride channel activity	GO:0016021:integral component of membrane
Pp03_22312-5p	18771175	PRUPE_ppa001423mg	NCBI_Assembly:GCF_000346465.1	NW_006760201.1	+	21500877	21506744	5867	pper03018:RNA degradation	GO:0006007:glucose catabolic process	-	-
Pp03_22312-5p	18771186	PRUPE_ppa012046mg	NCBI_Assembly:GCF_000346465.1	NW_006760201.1	-	16265088	16267042	1954	-	GO:0009560:embryo sac egg cell differentiation	-	-
Pp03_22312-5p	18771194	PRUPE_ppa010959mg	NCBI_Assembly:GCF_000346465.1	NW_006760201.1	-	9933049	9934144	1095	-	-	-	-

Pp03_22312-5p	18771263	PRUPE_ppa004154mg	NCBI_Assembly:GCF_000346465.1	NW_006760201.1	-	14801573	14804571	2998	-	GO:0000956:nuclear-transcribed mRNA catabolic process;GO:0006221:pyrimidine nucleotide biosynthetic process;GO:0048573:photoperiodism, flowering	GO:0003682:chromatin binding;GO:0033862:UMP kinase activity	GO:0005737:cytoplasm
Pp03_22312-5p	18771354	PRUPE_ppa011637mg	NCBI_Assembly:GCF_000346465.1	NW_006760201.1	+	17143214	17144411	1197	-	GO:0006950:response to stress;GO:0009415:response to water	-	-

Pp03_22312-5p	18771391	PRUPE_ppa005950mg	NCBI_Assembly:GCF_000346465.1	NW_006760201.1	-	16490755	16494212	3457	pper01100:Metabolic pathways;pper04141:Protein processing in endoplasmic reticulum;pper00510:N-Glycan biosynthesis	GO:0001510:RNA methylation;GO:0009664:plant-type cell wall organization;GO:0009826:unidimensional cell growth;GO:0018279:protein N-linked glycosylation via asparagine;GO:0030244:cellulose biosynthetic process;GO:0042545:cell wall modification;GO:0048193:Golgi vesicle transport	GO:0004579:dolichyl-diphosphooligosaccharide-protein glycotransferase activity	GO:0005730:nucleolus;GO:0005774:vacuolar membrane;GO:0005794:Golgi apparatus;GO:0008250:oligosaccharyltransferase complex;GO:0009505:plant-type cell wall;GO:0009506:plasmodesma;GO:0016021:integral component of membrane
Pp03_22312-5p	18771403	PRUPE_ppa005951mg	NCBI_Assembly:GCF_000346465.1	NW_006760201.1	+	13460554	13462935	2381	-	GO:0009850:auxin metabolic process;GO:0046482:para-aminobenzoic acid metabolic process	GO:0010210:IAA-Phe conjugate hydrolase activity;GO:0010211:IAA-Leu conjugate hydrolase activity	GO:0005783:endoplasmic reticulum
Pp03_22312-5p	18771537	PRUPE_ppa021020mg	NCBI_Assembly:GCF_000346465.1	NW_006760201.1	-	6263625	6264089	464	-	-	-	-

Pp03_22312-5p	18771541	PRUPE_ppa008990mg	NCBI_Assembly:GCF_000346465.1	NW_006760201.1	-	20478281	20481046	2765	-	GO:0009909:regulation of flower development;GO:0016570:histone modification;GO:0048449:floral organ formation	GO:0016740:transferase activity	GO:0005829:cytosol
Pp03_22312-5p	18771575	PRUPE_ppa020010mg	NCBI_Assembly:GCF_000346465.1	NW_006760201.1	+	15287878	15288578	700	-	GO:0006662:glycerol ether metabolic process;GO:0045454:cell redox homeostasis	GO:0015035:protein disulfide oxidoreductase activity	GO:0005623:cell
Pp03_22312-5p	18771580	PRUPE_ppa018092mg	NCBI_Assembly:GCF_000346465.1	NW_006760201.1	+	10698271	10699190	919	-	-	-	-
Pp03_22312-5p	18771607	PRUPE_ppa006708mg	NCBI_Assembly:GCF_000346465.1	NW_006760201.1	-	21139148	21141016	1868	-	GO:0055085:transmembrane transport	-	GO:0016021:integral component of membrane
Pp03_22312-5p	18771668	PRUPE_ppa014233mg	NCBI_Assembly:GCF_000346465.1	NW_006760201.1	-	9815789	9817454	1665	-	-	-	-

Pp03_22312-5p	18771683	PRUPE_ppa007050mg	NCBI_Assembly:GCF_000346465.1	NW_006760201.1	-	21387741	21389652	1911	-	GO:0006338:chromatin remodeling;GO:0006355:regulation of transcription, DNA-templated;GO:0008283:cell proliferation;GO:0009266:response to temperature stimulus;GO:0009910:negative regulation of flower development;GO:0030029:actin filament-based process;GO:0042742:defense response to bacterium	GO:0005200:structural constituent of cytoskeleton	GO:0005634:nucleus
Pp03_22312-5p	18771697	PRUPE_ppa013037mg	NCBI_Assembly:GCF_000346465.1	NW_006760201.1	-	2948705	2951617	2912	pper01100:Metabolic pathways;pper00230:Purine metabolism;pper00240:Pyrimidine metabolism;pper03020:RNA polymerase	GO:0006351:transcription, DNA-templated	GO:0003677:DNA binding;GO:0003899:DNA-directed RNA polymerase activity	GO:0005665:DNA-directed RNA polymerase II, core complex
Pp03_22312-5p	18771715	PRUPE_ppa025204mg	NCBI_Assembly:GCF_000346465.1	NW_006760201.1	-	19215056	19215631	575	-	-	GO:0000062:fatty-acyl-CoA binding	-

Pp03_22312-5p	18771721	PRUPE_ppa026457mg	NCBI_Assembly:GCF_000346465.1	NW_006760201.1	-	10016041	10016433	392	-	-	-	-
Pp03_22312-5p	18771792	PRUPE_ppa000190mg	NCBI_Assembly:GCF_000346465.1	NW_006760201.1	-	18189790	18194268	4478	pper00270:Cysteine and methionine metabolism;pper01100:Metabolic pathways	-	GO:0003677:DNA binding;GO:0003682:chromatin binding;GO:0003886:DNA (cytosine-5-)-methyltransferase activity	-
Pp03_22312-5p	18771796	PRUPE_ppa019594mg	NCBI_Assembly:GCF_000346465.1	NW_006760201.1	+	21342805	21343371	566	-	-	GO:0009055:electron carrier activity	-
Pp03_22312-5p	18771838	PRUPE_ppa023030mg	NCBI_Assembly:GCF_000346465.1	NW_006760201.1	+	12363890	12365424	1534	-	-	-	-
Pp03_22312-5p	18771881	PRUPE_ppa009379mg	NCBI_Assembly:GCF_000346465.1	NW_006760201.1	+	11226211	11230765	4554	pper04120:Ubiquitin mediated proteolysis	GO:0006511:ubiquitin-dependent protein catabolic process;GO:0007275:multicellular organismal development	GO:0004842:ubiquitin-protein transferase activity;GO:0008270:zinc ion binding;GO:0016874:ligase activity	GO:0005634:nucleus
Pp03_22312-5p	18771928	PRUPE_ppb022887mg	NCBI_Assembly:GCF_000346465.1	NW_006760208.1	-	5973018	5975072	2054	-	-	-	-
Pp03_22312-5p	18772009	PRUPE_ppa006559mg	NCBI_Assembly:GCF_000346465.1	NW_006760208.1	-	26971642	26974491	2849	-	-	-	GO:0005829:cytosol
Pp03_22312-5p	18772056	PRUPE_ppa002471mg	NCBI_Assembly:GCF_000346465.1	NW_006760208.1	-	23731153	23734669	3516	-	-	-	-

Pp03_22312-5p	18772061	PRUPE_ppa000777mg	NCBI_Assembly:GCF_000346465.1	NW_006760208.1	-	6131023	6140274	9251	-	GO:0009638:phototropism;GO:0009644:response to high light intensity;GO:0009744:response to sucrose;GO:0009903:chloroplast avoidance movement;GO:0009904:chloroplast accumulation movement;GO:0010114:response to red light;GO:0010119:regulation of stomatal movement;GO:0010155:regulation of proton transport;GO:0010218:response to far red light;GO:0010362:negative regulation of anion channel activity by blue	GO:0000155:phosphorelay sensor kinase activity;GO:0005524:ATP binding;GO:0009882:blue light photoreceptor activity;GO:0010181:FMN binding	GO:0005773:vacuole;GO:0009898:cytoplasmic side of plasma membrane;GO:0009986:cell surface
Pp03_22312-5p	18772068	PRUPE_ppa006584mg	NCBI_Assembly:GCF_000346465.1	NW_006760208.1	+	4335024	4337965	2941	-	-	GO:0008324:cation transmembrane transporter activity	GO:0016021:integral component of membrane

Pp03_22312-5p	18772079	PRUPE_ppa008999mg	NCBI_Assembly:GCF_000346465.1	NW_006760208.1	+	24326643	24328827	2184	-	GO:0006626:protein targeting to mitochondrion;GO:0009790:embryo development;GO:0019243:methylglyoxal catabolic process to D-lactate;GO:0033617:mitochondrial respiratory chain complex IV assembly	-	GO:0005739:mitochondrion
Pp03_22312-5p	18772171	PRUPE_ppa008068mg	NCBI_Assembly:GCF_000346465.1	NW_006760208.1	-	27629821	27631484	1663	-	-	-	-
Pp03_22312-5p	18772306	PRUPE_ppa015854mg	NCBI_Assembly:GCF_000346465.1	NW_006760208.1	+	4114693	4116797	2104	-	-	GO:0005215:transporter activity	GO:0016020:membrane
Pp03_22312-5p	18772312	PRUPE_ppa005524mg	NCBI_Assembly:GCF_000346465.1	NW_006760208.1	+	23087122	23091454	4332	-	-	-	-
Pp03_22312-5p	18772352	PRUPE_ppa000549mg	NCBI_Assembly:GCF_000346465.1	NW_006760208.1	-	1556773	1561912	5139	pper04120:Ubiquitin mediated proteolysis	GO:0006464:cellular protein modification process	GO:0005524:ATP binding;GO:0008641:small protein activating enzyme activity	-
Pp03_22312-5p	18772353	PRUPE_ppa013800mg	NCBI_Assembly:GCF_000346465.1	NW_006760208.1	+	27670179	27674177	3998	-	-	-	-

Pp03_22312-5p	18772414	PRUPE_ppa007429mg	NCBI_Assembly:GCF_000346465.1	NW_006760208.1	-	27874720	27877813	3093	pper00900:Terpenoid backbone biosynthesis	GO:0018343:protein farnesylation;GO:0042127:regulation of cell proliferation	GO:0003824:catalytic activity	GO:0005965:protein farnesyltransferase complex
Pp03_22312-5p	18772419	PRUPE_ppa006803mg	NCBI_Assembly:GCF_000346465.1	NW_006760208.1	+	23164222	23166494	2272	pper04141:Protein processing in endoplasmic reticulum	-	-	-
Pp03_22312-5p	18772495	PRUPE_ppa005717mg	NCBI_Assembly:GCF_000346465.1	NW_006760208.1	+	6620490	6623363	2873	pper03013:RNA transport	-	GO:0003746:translation elongation factor activity;GO:0003924:GTPase activity;GO:0005525:GTP binding	GO:0005737:cytoplasm
Pp03_22312-5p	18772593	PRUPE_ppa012761mg	NCBI_Assembly:GCF_000346465.1	NW_006760208.1	-	20621272	20622128	856	-	-	-	-
Pp03_22312-5p	18772637	PRUPE_ppa020578mg	NCBI_Assembly:GCF_000346465.1	NW_006760208.1	+	2213074	2214214	1140	-	-	-	-
Pp03_22312-5p	18772646	PRUPE_ppa010118mg	NCBI_Assembly:GCF_000346465.1	NW_006760208.1	-	2293929	2296069	2140	-	-	-	-
Pp03_22312-5p	18772688	PRUPE_ppa000158mg	NCBI_Assembly:GCF_000346465.1	NW_006760208.1	-	1084948	1098621	13673	-	-	-	-

Pp03_22312-5p	18772700	PRUPE_ppa001602mg	NCBI_Assembly:GCF_000346465.1	NW_006760208.1	-	23074357	23080451	6094	-	GO:0006399:tRNA metabolic process;GO:0009902:chloroplast relocation;GO:0010027:thylakoid membrane organization	GO:0000175:3'-5'-exoribonuclease activity	GO:0005623:cell
Pp03_22312-5p	18772729	PRUPE_ppa008251mg	NCBI_Assembly:GCF_000346465.1	NW_006760208.1	-	18078846	18081795	2949	pper04075:Plant hormone signal transduction	GO:0009651:response to salt stress	GO:0004672:protein kinase activity;GO:0005524:ATP binding	GO:0005634:nucleus;GO:0005829:cytosol
Pp03_22312-5p	18772802	PRUPE_ppa020782mg	NCBI_Assembly:GCF_000346465.1	NW_006760208.1	+	21112806	21117303	4497	-	-	-	-
Pp03_22312-5p	18772837	PRUPE_ppb023695mg	NCBI_Assembly:GCF_000346465.1	NW_006760208.1	-	23196406	23196913	507	-	-	GO:0030246:carbohydrate binding	-
Pp03_22312-5p	18772864	PRUPE_ppa018459mg	NCBI_Assembly:GCF_000346465.1	NW_006760208.1	-	26446961	26448303	1342	-	-	GO:0003723:RNA binding;GO:0033897:ribonuclease T2 activity	-
Pp03_22312-5p	18772946	PRUPE_ppa018192mg	NCBI_Assembly:GCF_000346465.1	NW_006760208.1	-	19082771	19085830	3059	-	-	-	-

Pp03_22312-5p	18773049	PRUPE_ppa016757mg	NCBI_Assembly:GCF_000346465.1	NW_006760208.1	+	3609173	3611775	2602	pper01100:Metabolic pathways;pper01110:Biosynthesis of secondary metabolites;pper00460:Cyanoamino acid metabolism;pper00500:Starch and sucrose metabolism;pper00940:Phenylpropanoid biosynthesis	GO:0005975:carbohydrate metabolic process	GO:0004553:hydrolase activity, hydrolyzing O-glycosyl compounds	-
Pp03_22312-5p	18774449	PRUPE_ppa005064mg	NCBI_Assembly:GCF_000346465.1	NW_006760208.1	-	630521	634193	3672	-	-	GO:0004672:protein kinase activity;GO:0005524:ATP binding	-
Pp03_22312-5p	18774487	PRUPE_ppa025912mg	NCBI_Assembly:GCF_000346465.1	NW_006760208.1	-	25157893	25159753	1860	-	-	GO:0003700:sequence-specific DNA binding transcription factor activity;GO:0043565:sequence-specific DNA binding	-
Pp03_22312-5p	18774500	PRUPE_ppb017342mg	NCBI_Assembly:GCF_000346465.1	NW_006760208.1	+	11455592	11456806	1214	-	-	GO:0030247:polysaccharide binding	-
Pp03_22312-5p	18774536	PRUPE_ppa008910mg	NCBI_Assembly:GCF_000346465.1	NW_006760208.1	-	7748308	7750219	1911	-	GO:0030001:metal ion transport	GO:0046872:metal ion binding	-

Pp03_22312-5p	18774564	PRUPE_ppa014550mg	NCBI_Assembly:GCF_000346465.1	NW_006760208.1	+	22790674	22791904	1230	-	-	-	-
Pp03_22312-5p	18775849	PRUPE_ppb020591mg	NCBI_Assembly:GCF_000346465.1	NW_006760212.1	+	4063730	4065532	1802	-	-	-	-
Pp03_22312-5p	18775856	PRUPE_ppa018383mg	NCBI_Assembly:GCF_000346465.1	NW_006760212.1	+	6023632	6026118	2486	-	-	GO:0008234: cysteine-type peptidase activity	-
Pp03_22312-5p	18775888	PRUPE_ppa022201mg	NCBI_Assembly:GCF_000346465.1	NW_006760212.1	+	12597330	12598918	1588	-	-	-	-
Pp03_22312-5p	18776035	PRUPE_ppa009615mg	NCBI_Assembly:GCF_000346465.1	NW_006760212.1	-	14497188	14499386	2198	pper03040:Spliceosome	-	GO:0003723: RNA binding	GO:0005634: nucleus;GO:0030529:ribonucleoprotein complex
Pp03_22312-5p	18776080	PRUPE_ppa012307mg	NCBI_Assembly:GCF_000346465.1	NW_006760212.1	+	13638431	13640407	1976	-	GO:0010103: stomatal complex morphogenesis;GO:0019761:glucosinolate biosynthetic process	-	GO:0031977: thylakoid lumen
Pp03_22312-5p	18777274	PRUPE_ppa019972mg	NCBI_Assembly:GCF_000346465.1	NW_006760212.1	+	5662613	5667405	4792	pper03440:Homologous recombination	-	GO:0003677: DNA binding;GO:0005524:ATP binding	-
Pp03_22312-5p	18777289	PRUPE_ppa020557m1g	NCBI_Assembly:GCF_000346465.1	NW_006760212.1	+	5379160	5381909	2749	-	GO:0006813: potassium ion transport;GO:0006814:sodium ion transport	GO:0005451: monovalent cation:proton antiporter activity	GO:0016021: integral component of membrane

Pp03_22312-5p	18777298	PRUPE_ppa001580mg	NCBI_Assembly:GCF_000346465.1	NW_006760212.1	+	2740518	2749583	9065	-	-	-	GO:0005794:Golgi apparatus;GO:0005886:plasma membrane;GO:0009506:plasmodesma
Pp03_22312-5p	18777326	PRUPE_ppa009893mg	NCBI_Assembly:GCF_000346465.1	NW_006760212.1	-	2766422	2773677	7255	-	GO:0015031:protein transport;GO:0016192:vesicle-mediated transport	-	GO:0005739:mitochondrion;GO:0005794:Golgi apparatus;GO:0016021:integral component of membrane
Pp03_22312-5p	18777339	PRUPE_ppa020915mg	NCBI_Assembly:GCF_000346465.1	NW_006760212.1	-	11448711	11449304	593	-	-	-	-
Pp03_22312-5p	18777360	PRUPE_ppa007800mg	NCBI_Assembly:GCF_000346465.1	NW_006760212.1	+	8261768	8264514	2746	-	-	GO:0022857:transmembrane transporter activity	GO:0016021:integral component of membrane
Pp03_22312-5p	18777377	PRUPE_ppa010508mg	NCBI_Assembly:GCF_000346465.1	NW_006760212.1	+	13817464	13820651	3187	pper01100:Metabolic pathways;pper01110:Biosynthesis of secondary metabolites;pper00230:Purine metabolism	-	GO:0004017:adenylate kinase activity;GO:0005524:ATP binding	-
Pp03_22312-5p	18777382	PRUPE_ppa010469mg	NCBI_Assembly:GCF_000346465.1	NW_006760212.1	-	5729215	5732911	3696	-	-	-	-
Pp03_22312-5p	18777403	PRUPE_ppa021078mg	NCBI_Assembly:GCF_000346465.1	NW_006760212.1	+	1128200	1129022	822	-	GO:0015979:photosynthesis	-	GO:0009507:chloroplast

Pp03_22312-5p	18777438	PRUPE_ppb020901mg	NCBI_Assembly:GCF_000346465.1	NW_006760212.1	+	9160979	9161546	567	-	-	GO:0005506:iron ion binding;GO:0016705:oxidoreductase activity, acting on paired donors, with incorporation or reduction of molecular oxygen;GO:0020037:heme binding	-
Pp03_22312-5p	18777604	PRUPE_ppa021984mg	NCBI_Assembly:GCF_000346465.1	NW_006760212.1	-	4649750	4651724	1974	-	-	-	GO:0005615:extracellular space

Pp03_22312-5p	18777687	PRUPE_ppa001337mg	NCBI_Assembly:GCF_000346465.1	NW_006760212.1	+	15206557	15210354	3797	-	GO:0006364:rRNA processing;GO:0006399:tRNA metabolic process;GO:0009902:chloroplast relocation;GO:0009965:leaf morphogenesis;GO:0010027:thylakoid membrane organization;GO:0010103:stomatal complex morphogenesis;GO:0010207:photosystem II assembly;GO:0030154:cell differentiation;GO:0035304:regulation of protein dephosphorylation;GO:0042793:transcription from plastid promoter;GO:0045036:protein targeting	-	GO:0009508:plastid chromosome

Pp03_22312-5p	18777704	PRUPE_ppa008803mg	NCBI_Assembly:GCF_000346465.1	NW_006760212.1	+	13883783	13886387	2604	pper01100:Metabolic pathways;pper00061:Fatty acid biosynthesis;pper01212:Fatty acid metabolism;pper00780:Biotin metabolism;pper01040:Biosynthesis of unsaturated fatty acids	GO:0006633:fatty acid biosynthetic process	GO:0004316:3-oxoacyl-[acyl-carrier-protein] reductase (NADPH) activity;GO:0051287:NAD binding	-
Pp03_22312-5p	18777706	PRUPE_ppa020153mg	NCBI_Assembly:GCF_000346465.1	NW_006760212.1	+	17269333	17270046	713	-	-	-	-
Pp03_22312-5p	18777724	PRUPE_ppa001401mg	NCBI_Assembly:GCF_000346465.1	NW_006760212.1	-	11291521	11298019	6498	pper04120:Ubiquitin mediated proteolysis	-	GO:0008270:zinc ion binding;GO:0019789:SUMO transferase activity	-
Pp03_22312-5p	18777748	PRUPE_ppa003933mg	NCBI_Assembly:GCF_000346465.1	NW_006760212.1	+	13731116	13732879	1763	-	GO:0006887:exocytosis;GO:0009644:response to high light intensity;GO:0042542:response to hydrogen peroxide	-	GO:0000145:exocyst;GO:0005829:cytosol;GO:0005886:plasma membrane
Pp03_22312-5p	18777777	PRUPE_ppa004401mg	NCBI_Assembly:GCF_000346465.1	NW_006760212.1	+	15968797	15971870	3073	-	-	GO:0003676:nucleic acid binding;GO:0004527:exonuclease activity	-

Pp03_22312-5p	18777893	PRUPE_ppa001070mg	NCBI_Assembly:GCF_000346465.1	NW_006760212.1	-	9561932	9571190	9258	-	GO:0007018:microtubule-based movement	GO:0003777:microtubule motor activity;GO:0005524:ATP binding	GO:0005871:kinesin complex;GO:0005874:microtubule
Pp03_22312-5p	18777900	PRUPE_ppa008487mg	NCBI_Assembly:GCF_000346465.1	NW_006760212.1	+	14331378	14332432	1054	pper00230:Purine metabolism;pper00564:Glycerophospholipid metabolism	-	GO:0016787:hydrolase activity	-
Pp03_22312-5p	18778031	PRUPE_ppa002540mg	NCBI_Assembly:GCF_000346465.1	NW_006760212.1	-	11954032	11960462	6430	pper01100:Metabolic pathways;pper00071:Fatty acid degradation;pper00061:Fatty acid biosynthesis;pper01212:Fatty acid metabolism;pper04146:Peroxisome	-	GO:0003824:catalytic activity	-
Pp03_22312-5p	18778042	PRUPE_ppa019022mg	NCBI_Assembly:GCF_000346465.1	NW_006760212.1	-	11538308	11538805	497	-	-	-	-
Pp03_22312-5p	18778065	PRUPE_ppa021772mg	NCBI_Assembly:GCF_000346465.1	NW_006760212.1	+	3232896	3236971	4075	-	GO:0030244:cellulose biosynthetic process	GO:0016760:cellulose synthase (UDP-forming) activity	GO:0016021:integral component of membrane
Pp03_22312-5p	18778102	PRUPE_ppa015539mg	NCBI_Assembly:GCF_000346465.1	NW_006760212.1	+	18311792	18315505	3713	-	-	-	-
Pp03_22312-5p	18778104	PRUPE_ppa003786mg	NCBI_Assembly:GCF_000346465.1	NW_006760212.1	+	13813325	13816644	3319	pper03008:Ribosome biogenesis in eukaryotes	-	-	-

Pp03_22312-5p	18778127	PRUPE_ppa020524mg	NCBI_Assembly:GCF_000346465.1	NW_006760212.1	+	11698624	11701254	2630	-	-	GO:0003700:sequence-specific DNA binding transcription factor activity;GO:0043565:sequence-specific DNA binding	-
Pp03_22312-5p	18778308	PRUPE_ppa022517mg	NCBI_Assembly:GCF_000346465.1	NW_006760220.1	+	13783864	13787937	4073	-	GO:0006396:RNA processing	GO:0003723:RNA binding;GO:0004525:ribonuclease III activity	-
Pp03_22312-5p	18778321	PRUPE_ppa018797mg	NCBI_Assembly:GCF_000346465.1	NW_006760220.1	-	1202660	1205814	3154	-	-	-	-
Pp03_22312-5p	18778325	PRUPE_ppa025243mg	NCBI_Assembly:GCF_000346465.1	NW_006760220.1	-	27790549	27793347	2798	-	-	-	-
Pp03_22312-5p	18778366	PRUPE_ppa021398mg	NCBI_Assembly:GCF_000346465.1	NW_006760220.1	-	4514473	4514937	464	-	-	-	-
Pp03_22312-5p	18778448	PRUPE_ppa022439mg	NCBI_Assembly:GCF_000346465.1	NW_006760220.1	-	28347468	28350514	3046	-	-	GO:0043531:ADP binding	-
Pp03_22312-5p	18778481	PRUPE_ppa011248mg	NCBI_Assembly:GCF_000346465.1	NW_006760220.1	+	580336	583632	3296	-	-	-	-
Pp03_22312-5p	18778483	PRUPE_ppa026799mg	NCBI_Assembly:GCF_000346465.1	NW_006760220.1	-	1229201	1231172	1971	-	GO:0009685:gibberellin metabolic process	GO:0046872:metal ion binding;GO:0052635:C-20 gibberellin 2-beta-dioxygenase activity	-
Pp03_22312-5p	18778499	PRUPE_ppb012387mg	NCBI_Assembly:GCF_000346465.1	NW_006760220.1	+	2238741	2240507	1766	-	-	-	-

Pp03_22312-5p	18778593	PRUPE_ppa008240mg	NCBI_Assembly:GCF_000346465.1	NW_006760220.1	-	447844	451877	4033	-	-	-	-
Pp03_22312-5p	18778614	PRUPE_ppa020065mg	NCBI_Assembly:GCF_000346465.1	NW_006760220.1	+	5802166	5805539	3373	pper04626:Plant-pathogen interaction	-	GO:0043531:ADP binding	-
Pp03_22312-5p	18778616	PRUPE_ppa014399mg	NCBI_Assembly:GCF_000346465.1	NW_006760220.1	+	16885833	16886879	1046	-	-	-	-
Pp03_22312-5p	18778630	PRUPE_ppa003664mg	NCBI_Assembly:GCF_000346465.1	NW_006760220.1	+	6819825	6823492	3667	-	GO:0009611:response to wounding;GO:0009805:coumarin biosynthetic process;GO:0009963:positive regulation of flavonoid biosynthetic process	-	-
Pp03_22312-5p	18778646	PRUPE_ppa011477mg	NCBI_Assembly:GCF_000346465.1	NW_006760220.1	+	2839646	2842103	2457	pper03010:Ribosome	GO:0006412:translation	GO:0003735:structural constituent of ribosome	GO:0005840:ribosome
Pp03_22312-5p	18778661	PRUPE_ppa003305mg	NCBI_Assembly:GCF_000346465.1	NW_006760220.1	+	14951801	14956955	5154	-	GO:0009616:virus induced gene silencing;GO:0010050:vegetative phase change;GO:0045893:positive regulation of transcription, DNA-templated	GO:0003700:sequence-specific DNA binding transcription factor activity;GO:0043565:sequence-specific DNA binding	-

Pp03_22312-5p	18778717	PRUPE_ppa020787mg	NCBI_Assembly:GCF_000346465.1	NW_006760220.1	+	4817812	4824534	6722	-	GO:0006312:mitotic recombination;GO:0006342:chromatin silencing;GO:0006406:mRNA export from nucleus;GO:0007062:sister chromatid cohesion;GO:0009560:embryo sac egg cell differentiation;GO:0009640:photomorphogenesis;GO:0009845:seed germination;GO:0009855:determination of bilateral symmetry;GO:0009880:embryonic pattern specification;GO:0009909:regulation of flower development;GO:0010072:primary shoot apical meristem	-	GO:0005622:intracellular
Pp03_22312-5p	18778718	PRUPE_ppa002800mg	NCBI_Assembly:GCF_000346465.1	NW_006760220.1	-	178415	181151	2736	-	-	-	-

Pp03_22312-5p	18778734	PRUPE_ppa007559mg	NCBI_Assembly:GCF_000346465.1	NW_006760220.1	-	17865514	17869992	4478	-	GO:0006626: protein targeting to mitochondrion;GO:0009220:pyrimidine ribonucleotide biosynthetic process;GO:0009640:photomorphogenesis;GO:0009853:photorespiration;GO:0010388:cullin deneddylation	-	GO:0005744: mitochondrial inner membrane presequence translocase complex
Pp03_22312-5p	18778756	PRUPE_ppa026718mg	NCBI_Assembly:GCF_000346465.1	NW_006760220.1	-	29907116	29908327	1211	-	GO:0006284: base-excision repair	GO:0003824: catalytic activity	-

Pp03_22312-5p	18778836	PRUPE_ppa005089mg	NCBI_Assembly:GCF_000346465.1	NW_006760220.1	+	371785	376087	4302	-	GO:0000278:mitotic cell cycle;GO:0006396:RNA processing;GO:0007129:synapsis;GO:0007131:reciprocal meiotic recombination;GO:0009410:response to xenobiotic stimulus;GO:0042138:meiotic DNA double-strand break formation;GO:0048522:positive regulation of cellular process;GO:0070482:response to oxygen levels	-	GO:0005634:nucleus;GO:0005829:cytosol
Pp03_22312-5p	18778895	PRUPE_ppa008940mg	NCBI_Assembly:GCF_000346465.1	NW_006760220.1	-	1123214	1125328	2114	pper01100:Metabolic pathways;pper00564:Glycerophospholipid metabolism	GO:0008654:phospholipid biosynthetic process;GO:0019722:calcium-mediated signaling	GO:0008808:cardiolipin synthase activity	GO:0005739:mitochondrion;GO:0016020:membrane
Pp03_22312-5p	18778962	PRUPE_ppa009361mg	NCBI_Assembly:GCF_000346465.1	NW_006760220.1	-	5230934	5234898	3964	-	GO:0006886:intracellular protein transport	-	GO:0005622:intracellular; GO:0005886:plasma membrane

Pp03_22312-5p	18779050	PRUPE_ppa018063mg	NCBI_Assembly:GCF_000346465.1	NW_006760220.1	-	8433749	8434486	737	pper00480:Glutathione metabolism	-	-	-
Pp03_22312-5p	18779054	PRUPE_ppb022006mg	NCBI_Assembly:GCF_000346465.1	NW_006760220.1	+	16438371	16438946	575	-	-	-	-
Pp03_22312-5p	18779091	PRUPE_ppa003523mg	NCBI_Assembly:GCF_000346465.1	NW_006760220.1	-	63451	67882	4431	-	-	-	-
Pp03_22312-5p	18779163	PRUPE_ppa023254mg	NCBI_Assembly:GCF_000346465.1	NW_006760220.1	-	142889	144580	1691	-	-	-	-
Pp03_22312-5p	18779205	PRUPE_ppa007088mg	NCBI_Assembly:GCF_000346465.1	NW_006760220.1	-	3959911	3961403	1492	-	GO:0005975: carbohydrate metabolic process	GO:0004553: hydrolase activity, hydrolyzing O-glycosyl compounds	-
Pp03_22312-5p	18779290	PRUPE_ppa014421mg	NCBI_Assembly:GCF_000346465.1	NW_006760220.1	+	532939	534543	1604	pper01100:Metabolic pathways;pper04145:Phagosome;pper00190:Oxidative phosphorylation	GO:0015991: ATP hydrolysis coupled proton transport	GO:0015078: hydrogen ion transmembrane transporter activity	GO:0033179: proton-transporting V-type ATPase, V0 domain
Pp03_22312-5p	18779370	PRUPE_ppa026747mg	NCBI_Assembly:GCF_000346465.1	NW_006760220.1	-	17227990	17229984	1994	-	-	GO:0043531: ADP binding	-
Pp03_22312-5p	18779373	PRUPE_ppa008276mg	NCBI_Assembly:GCF_000346465.1	NW_006760220.1	-	26922074	26930698	8624	-	GO:0010207: photosystem II assembly	-	-
Pp03_22312-5p	18779415	PRUPE_ppa008970mg	NCBI_Assembly:GCF_000346465.1	NW_006760220.1	+	3814257	3816525	2268	-	-	-	-
Pp03_22312-5p	18779466	PRUPE_ppa027161mg	NCBI_Assembly:GCF_000346465.1	NW_006760220.1	-	9358730	9361783	3053	-	-	-	-

Pp03_22312-5p	18779484	PRUPE_ppa004357mg	NCBI_Assembly:GCF_000346465.1	NW_006760220.1	-	6310792	6312820	2028	-	GO:0009556:microsporogenesis;GO:0009827:plant-type cell wall modification;GO:0009860:pollen tube growth;GO:0010584:pollen exine formation	-	-
Pp03_22312-5p	18779487	PRUPE_ppb013299mg	NCBI_Assembly:GCF_000346465.1	NW_006760220.1	+	11826632	11827271	639	-	-	GO:0000287:magnesium ion binding;GO:0010333:terpene synthase activity	-
Pp03_22312-5p	18779554	PRUPE_ppa004781mg	NCBI_Assembly:GCF_000346465.1	NW_006760220.1	-	1713119	1716755	3636	pper01100:Metabolic pathways;pper00600:Sphingolipid metabolism	-	GO:0016301:kinase activity	-
Pp03_22312-5p	18779568	PRUPE_ppa025091mg	NCBI_Assembly:GCF_000346465.1	NW_006760220.1	+	21280613	21280978	365	-	GO:0006032:chitin catabolic process;GO:0016998:cell wall macromolecule catabolic process	GO:0004568:chitinase activity	-

Pp03_22312-5p	18779585	PRUPE_ppa009987mg	NCBI_Assembly:GCF_000346465.1	NW_006760220.1	-	11357222	11358821	1599	pper01100:Metabolic pathways;pper00196:Photosynthesis - antenna proteins	GO:0009765:photosynthesis, light harvesting;GO:0018298:protein-chromophore linkage	GO:0005198:structural molecule activity;GO:0016168:chlorophyll binding;GO:0046872:metal ion binding	GO:0009522:photosystem I;GO:0009523:photosystem II;GO:0009535:chloroplast thylakoid membrane;GO:0016021:integral component of membrane
Pp03_22312-5p	18779613	PRUPE_ppa1027164mg	NCBI_Assembly:GCF_000346465.1	NW_006760220.1	-	2820107	2821422	1315	-	GO:0006629:lipid metabolic process	GO:0016787:hydrolase activity	-
Pp03_22312-5p	18779639	PRUPE_ppa021855mg	NCBI_Assembly:GCF_000346465.1	NW_006760220.1	-	8902793	8905237	2444	-	-	GO:0051119:sugar transmembrane transporter activity	GO:0005887:integral component of plasma membrane
Pp03_22312-5p	18779702	PRUPE_ppa010528mg	NCBI_Assembly:GCF_000346465.1	NW_006760220.1	-	797379	799395	2016	-	GO:0042744:hydrogen peroxide catabolic process	-	-

Pp03_22312-5p	18779919	PRUPE_ppa005063mg	NCBI_Assembly:GCF_000346465.1	NW_006760220.1	-	3245161	3252727	7566	pper01100:Metabolic pathways;pper01110:Biosynthesis of secondary metabolites;pper01230:Biosynthesis of amino acids;pper01210:2-Oxocarboxylic acid metabolism;pper00290:Valine, leucine and isoleucine biosynthesis;pper00770:Pantothenate and CoA biosynthesis;pper00650:Butanoate metabolism;pper00660:C5-Branched dibasic acid metabolism	GO:0006094:gluconeogenesis;GO:0006551:leucine metabolic process;GO:0006573:valine metabolic process;GO:0007010:cytoskeleton organization;GO:0009082:branched-chain amino acid biosynthetic process;GO:0010498:proteasomal protein catabolic process	GO:0003984:acetolactate synthase activity;GO:0016597:amino acid binding	GO:0005829:cytosol
Pp03_22312-5p	18779928	PRUPE_ppa026097mg	NCBI_Assembly:GCF_000346465.1	NW_006760220.1	+	23754358	23754744	386	-	-	-	-
Pp03_22312-5p	18779933	PRUPE_ppa017283mg	NCBI_Assembly:GCF_000346465.1	NW_006760220.1	-	16657974	16658725	751	-	-	-	-
Pp03_22312-5p	18779934	PRUPE_ppa003057mg	NCBI_Assembly:GCF_000346465.1	NW_006760220.1	+	8334925	8337255	2330	-	-	-	-
Pp03_22312-5p	18779954	PRUPE_ppa013505mg	NCBI_Assembly:GCF_000346465.1	NW_006760220.1	-	919547	921661	2114	pper04140:Regulation of autophagy	GO:0006914:autophagy	-	-

Pp03_22312-5p	18780066	PRUPE_ppa001879mg	NCBI_Assembly:GCF_000346465.1	NW_006760220.1	+	18668341	18672897	4556	pper01100:Metabolic pathways;pper00920:Sulfur metabolism	GO:0006096:glycolytic process;GO:0006275:regulation of DNA replication;GO:0006323:DNA packaging;GO:0006833:water transport;GO:0006972:hyperosmotic response;GO:0007030:Golgi organization;GO:0009266:response to temperature stimulus;GO:0009684:indoleacetic acid biosynthetic process;GO:0016126:sterol biosynthetic process;GO:0019344:cysteine biosynthetic process;GO:0019424:sulfide oxidation, using	GO:0003677:DNA binding;GO:0005507:copper ion binding;GO:0016002:sulfite reductase activity;GO:0020037:heme binding;GO:0050311:sulfite reductase (ferredoxin) activity;GO:0051539:4 iron, 4 sulfur cluster binding	GO:0009941:chloroplast envelope;GO:0010319:stromule;GO:0016020:membrane;GO:0042644:chloroplast nucleoid;GO:0048046:apoplast
Pp03_22312-5p	18780171	PRUPE_ppa015049mg	NCBI_Assembly:GCF_000346465.1	NW_006760220.1	-	24427112	24427684	572	-	-	-	-

Pp03_22312-5p	18780173	PRUPE_ppa006371mg	NCBI_Assembly:GCF_000346465.1	NW_006760220.1	-	226871	230338	3467	pper01100:Metabolic pathways;pper01110:Biosynthesis of secondary metabolites;pper01200:Carbon metabolism;pper00620:Pyruvate metabolism;pper00071:Fatty acid degradation; pper00280:Valine, leucine and isoleucine degradation; pper00630:Glyoxylate and dicarboxylate metabolism;pper01212:Fatty acid metabolism;pper00640:Propanoate metabolism;pper00900:Terpenoid backbone biosynthesis; pper00310:Lysine degradation; pper00380:Tr[illegible]	GO:0016126: sterol biosynthetic process;GO:0019745:pentacyclic triterpenoid biosynthetic process	GO:0016747: transferase activity, transferring acyl groups other than amino-acyl groups	-
Pp03_22312-5p	18780174	PRUPE_ppa013448mg	NCBI_Assembly:GCF_000346465.1	NW_006760220.1	-	4639904	4643419	3515	-	-	-	-
Pp03_22312-5p	18780256	PRUPE_ppa003626mg	NCBI_Assembly:GCF_000346465.1	NW_006760220.1	-	2007279	2012853	5574	pper03040:Spliceosome	-	-	GO:0005634: nucleus

Pp03_22312-5p	18780262	PRUPE_ppa008801mg	NCBI_Assembly:GCF_000346465.1	NW_006760220.1	+	8224220	8228164	3944	-	GO:0006351:transcription, DNA-templated;GO:0006355:regulation of transcription, DNA-templated	GO:0003677:DNA binding	GO:0005634:nucleus
Pp03_22312-5p	18780266	PRUPE_ppa024516m2g	NCBI_Assembly:GCF_000346465.1	NW_006760220.1	+	7748593	7748703	110	-	-	-	-
Pp03_22312-5p	18780275	PRUPE_ppa005194mg	NCBI_Assembly:GCF_000346465.1	NW_006760220.1	-	1147317	1150766	3449	pper01100:Metabolic pathways;pper01110:Biosynthesis of secondary metabolites;pper00460:Cyanoamino acid metabolism;pper00500:Starch and sucrose metabolism;pper00940:Phenylpropanoid biosynthesis	GO:0005975:carbohydrate metabolic process	GO:0004553:hydrolase activity, hydrolyzing O-glycosyl compounds	-
Pp03_22312-5p	18780295	PRUPE_ppa016371mg	NCBI_Assembly:GCF_000346465.1	NW_006760220.1	+	10604804	10606998	2194	-	-	GO:0005215:transporter activity	GO:0016021:integral component of membrane
Pp03_22312-5p	18780301	PRUPE_ppa023029mg	NCBI_Assembly:GCF_000346465.1	NW_006760220.1	-	11313326	11314492	1166	-	-	-	-
Pp03_22312-5p	18780333	PRUPE_ppa009296mg	NCBI_Assembly:GCF_000346465.1	NW_006760220.1	+	3124702	3126635	1933	-	-	GO:0008270:zinc ion binding	GO:0016020:membrane

Pp03_22312-5p	18780356	PRUPE_ppa012334mg	NCBI_Assembly:GCF_000346465.1	NW_006760220.1	-		16584437	16586139	1702	-	-	-	-
Pp03_22312-5p	18780376	PRUPE_ppa013712mg	NCBI_Assembly:GCF_000346465.1	NW_006760220.1	-		18678435	18678932	497	-	-	-	-
Pp03_22312-5p	18780430	PRUPE_ppa018994mg	NCBI_Assembly:GCF_000346465.1	NW_006760220.1	+		791176	794040	2864	-	-	GO:0008375:acetylglucosaminyltransferase activity	GO:0016020:membrane
Pp03_22312-5p	18780454	PRUPE_ppb019697mg	NCBI_Assembly:GCF_000346465.1	NW_006760220.1	+		29691221	29694100	2879	-	-	-	-
Pp03_22312-5p	18780495	PRUPE_ppa003651m1g	NCBI_Assembly:GCF_000346465.1	NW_006760220.1	+		8851880	8853887	2007	-	-	GO:0016772:transferase activity, transferring phosphorus-containing groups	-
Pp03_22312-5p	18780530	PRUPE_ppa026814mg	NCBI_Assembly:GCF_000346465.1	NW_006760220.1	+		15093394	15097382	3988	-	-	GO:0015238:drug transmembrane transporter activity;GO:0015297:antiporter activity	GO:0016020:membrane
Pp03_22312-5p	18780532	PRUPE_ppa015462mg	NCBI_Assembly:GCF_000346465.1	NW_006760220.1	-		2611850	2613380	1530	-	-	-	-
Pp03_22312-5p	18780557	PRUPE_ppa011879mg	NCBI_Assembly:GCF_000346465.1	NW_006760220.1	+		12440571	12443355	2784	pper03010:Ribosome	GO:0019288:isopentenyl diphosphate biosynthetic process, methylerythritol 4-phosphate pathway;GO:0032544:plastid translation	GO:0003735:structural constituent of ribosome	GO:0005840:ribosome;GO:0009570:chloroplast stroma;GO:0009941:chloroplast envelope

Pp03_22312-5p	18780562	PRUPE_ppa025444mg	NCBI_Assembly:GCF_000346465.1	NW_006760220.1	-	3325702	3327276	1574	-	-	GO:0016791:phosphatase activity	-
Pp03_22312-5p	18780573	PRUPE_ppa010773mg	NCBI_Assembly:GCF_000346465.1	NW_006760220.1	-	174576	177284	2708	pper01100:Metabolic pathways;pper00230:Purine metabolism;pper00240:Pyrimidine metabolism;pper03020:RNA polymerase	GO:0006351:transcription, DNA-templated	GO:0000166:nucleotide binding;GO:0003899:DNA-directed RNA polymerase activity	-
Pp03_22312-5p	18780584	PRUPE_ppa002367mg	NCBI_Assembly:GCF_000346465.1	NW_006760220.1	-	9700021	9703458	3437	-	GO:0045454:cell redox homeostasis	-	GO:0005623:cell
Pp03_22312-5p	18781400	PRUPE_ppa023515mg	NCBI_Assembly:GCF_000346465.1	NW_006760220.1	-	15364007	15365517	1510	-	-	GO:0008234:cysteine-type peptidase activity	GO:0048046:apoplast
Pp03_22312-5p	18781427	PRUPE_ppa026271mg	NCBI_Assembly:GCF_000346465.1	NW_006760220.1	-	3529765	3534938	5173	pper03430:Mismatch repair	GO:0006281:DNA repair;GO:0007062:sister chromatid cohesion;GO:0031048:chromatin silencing by small RNA;GO:0045132:meiotic chromosome segregation	GO:0003677:DNA binding;GO:0004518:nuclease activity	GO:0005634:nucleus
Pp03_22312-5p	18781430	PRUPE_ppa014874mg	NCBI_Assembly:GCF_000346465.1	NW_006760220.1	+	17412445	17413941	1496	-	-	-	-

Pp03_22312-5p	18781475	PRUPE_ppa006330mg	NCBI_Assembly:GCF_000346465.1	NW_006760220.1	+	22019831	22023469	3638	pper04141:Protein processing in endoplasmic reticulum	GO:0006457:protein folding;GO:0009408:response to heat	GO:0005524:ATP binding;GO:0046872:metal ion binding	-
Pp03_22312-5p	18781534	PRUPE_ppa020696mg	NCBI_Assembly:GCF_000346465.1	NW_006760220.1	+	7746193	7748349	2156	-	-	GO:0003924:GTPase activity;GO:0005525:GTP binding	-
Pp03_22312-5p	18781540	PRUPE_ppa004167m1g	NCBI_Assembly:GCF_000346465.1	NW_006760220.1	+	167610	168491	881	pper01100:Metabolic pathways;pper01110:Biosynthesis of secondary metabolites;pper01230:Biosynthesis of amino acids;pper01200:Carbon metabolism;pper00010:Glycolysis / Gluconeogenesis;pper00052:Galactose metabolism;pper03018:RNA degradation;pper00051:Fructose and mannose metabolism;pper00030:Pentose phosphate pathway	GO:0006096:glycolytic process	GO:0003872:6-phosphofructokinase activity	-

Pp03_22312-5p	18781600	PRUPE_ppa003326mg	NCBI_Assembly:GCF_000346465.1	NW_006760268.1	-	12971901	12974091	2190	-	GO:0009553:embryo sac development;GO:0009555:pollen development;GO:0009790:embryo development	-	GO:0005887:integral component of plasma membrane
Pp03_22312-5p	18781655	PRUPE_ppa009213mg	NCBI_Assembly:GCF_000346465.1	NW_006760268.1	-	17762905	17764642	1737	-	-	-	-
Pp03_22312-5p	18781668	PRUPE_ppa004502mg	NCBI_Assembly:GCF_000346465.1	NW_006760268.1	-	1888163	1890891	2728	-	GO:0007020:microtubule nucleation	-	GO:0005794:Golgi apparatus;GO:0016021:integral component of membrane
Pp03_22312-5p	18782886	PRUPE_ppa017150mg	NCBI_Assembly:GCF_000346465.1	NW_006760268.1	-	14401736	14402248	512	-	-	GO:0004672:protein kinase activity;GO:0005524:ATP binding	-
Pp03_22312-5p	18782896	PRUPE_ppa017518mg	NCBI_Assembly:GCF_000346465.1	NW_006760268.1	-	16897878	16899573	1695	-	-	-	-
Pp03_22312-5p	18782909	PRUPE_ppa018932mg	NCBI_Assembly:GCF_000346465.1	NW_006760268.1	-	8865132	8867201	2069	-	-	-	-
Pp03_22312-5p	18782976	PRUPE_ppa003996mg	NCBI_Assembly:GCF_000346465.1	NW_006760268.1	-	16286986	16291067	4081	-	-	GO:0022891:substrate-specific transmembrane transporter activity	GO:0016021:integral component of membrane
Pp03_22312-5p	18783036	PRUPE_ppa010115mg	NCBI_Assembly:GCF_000346465.1	NW_006760268.1	+	20928999	20930913	1914	-	-	-	-

Pp03_22312-5p	18783128	PRUPE_ppa001069mg	NCBI_Assembly:GCF_000346465.1	NW_006760268.1	-		14516366	14523001	6635	-	GO:0006351:transcription, DNA-templated;GO:0006355:regulation of transcription, DNA-templated;GO:0009734:auxin-activated signaling pathway	GO:0003677:DNA binding	GO:0005634:nucleus
Pp03_22312-5p	18783129	PRUPE_ppa008165mg	NCBI_Assembly:GCF_000346465.1	NW_006760268.1	+		12350218	12358599	8381	pper01100:Metabolic pathways;pper00750:Vitamin B6 metabolism	GO:0008615:pyridoxine biosynthetic process;GO:0009443:pyridoxal 5'-phosphate salvage;GO:0010054:trichoblast differentiation;GO:0042538:hyperosmotic salinity response	GO:0008478:pyridoxal kinase activity	GO:0005829:cytosol
Pp03_22312-5p	18783191	PRUPE_ppa000327mg	NCBI_Assembly:GCF_000346465.1	NW_006760268.1	-		14189720	14198128	8408	-	-	GO:0003676:nucleic acid binding;GO:0004386:helicase activity;GO:0005524:ATP binding	-
Pp03_22312-5p	18783208	PRUPE_ppb021687mg	NCBI_Assembly:GCF_000346465.1	NW_006760268.1	-		10715270	10716884	1614	-	-	-	-
Pp03_22312-5p	18783252	PRUPE_ppb020002mg	NCBI_Assembly:GCF_000346465.1	NW_006760268.1	+		8392996	8394977	1981	-	-	-	-

Pp03_22312-5p	18783263	PRUPE_ppa015845mg	NCBI_Assembly:GCF_000346465.1	NW_006760268.1	+	19304869	19306167	1298	-	-	GO:0016758: transferase activity, transferring hexosyl groups	-
Pp03_22312-5p	18783288	PRUPE_ppa006360mg	NCBI_Assembly:GCF_000346465.1	NW_006760268.1	+	10718998	10724586	5588	-	-	GO:0003746: translation elongation factor activity	GO:0005739: mitochondrion
Pp03_22312-5p	18783337	PRUPE_ppa016909mg	NCBI_Assembly:GCF_000346465.1	NW_006760268.1	-	14385838	14386253	415	-	-	GO:0030247: polysaccharide binding	-
Pp03_22312-5p	18783349	PRUPE_ppa016385mg	NCBI_Assembly:GCF_000346465.1	NW_006760268.1	-	18630946	18633249	2303	-	-	GO:0003677: DNA binding;GO:0003682:chromatin binding;GO:0003700:sequence-specific DNA binding transcription factor activity	-
Pp03_22312-5p	18783399	PRUPE_ppa024881mg	NCBI_Assembly:GCF_000346465.1	NW_006760268.1	+	12969608	12971568	1960	-	GO:0016556: mRNA modification	-	-
Pp03_22312-5p	18783428	PRUPE_ppa018288mg	NCBI_Assembly:GCF_000346465.1	NW_006760268.1	-	9114370	9116676	2306	-	-	GO:0003676: nucleic acid binding	-
Pp03_22312-5p	18783438	PRUPE_ppb012454mg	NCBI_Assembly:GCF_000346465.1	NW_006760268.1	-	7355749	7356280	531	-	-	GO:0003677: DNA binding	-
Pp03_22312-5p	18783450	PRUPE_ppa023158mg	NCBI_Assembly:GCF_000346465.1	NW_006760268.1	-	10148936	10150835	1899	-	-	GO:0004672: protein kinase activity;GO:0005524:ATP binding	-
Pp03_22312-5p	18783526	PRUPE_ppa013164mg	NCBI_Assembly:GCF_000346465.1	NW_006760268.1	-	21108665	21109712	1047	-	-	-	-

Pp03_22312-5p	18783579	PRUPE_ppa018693mg	NCBI_Assembly:GCF_000346465.1	NW_006760268.1	+	13418638	13421372	2734	-	-	-	-
Pp03_22312-5p	18783596	PRUPE_ppa008118mg	NCBI_Assembly:GCF_000346465.1	NW_006760268.1	-	19296139	19298487	2348	-	GO:0006396:RNA processing	GO:0003723:RNA binding;GO:0008173:RNA methyltransferase activity	-
Pp03_22312-5p	18783615	PRUPE_ppb012922mg	NCBI_Assembly:GCF_000346465.1	NW_006760268.1	+	20048116	20054175	6059	-	-	-	-
Pp03_22312-5p	18783700	PRUPE_ppa024541mg	NCBI_Assembly:GCF_000346465.1	NW_006760268.1	-	4983794	4985276	1482	-	GO:0045454:cell redox homeostasis	GO:0009055:electron carrier activity;GO:0015035:protein disulfide oxidoreductase activity	GO:0005623:cell
Pp03_22312-5p	18783764	PRUPE_ppa020667m1g	NCBI_Assembly:GCF_000346465.1	NW_006760268.1	+	20659292	20662357	3065	-	-	-	-

Pp03_22312-5p	18783775	PRUPE_ppa009747mg	NCBI_Assembly:GCF_000346465.1	NW_006760268.1	-	20399064	20401045	1981	-	GO:0006351:transcription, DNA-templated;GO:0009965:leaf morphogenesis;GO:0010167:response to nitrate;GO:0015706:nitrate transport;GO:0015996:chlorophyll catabolic process;GO:0045893:positive regulation of transcription, DNA-templated	GO:0003700:sequence-specific DNA binding transcription factor activity;GO:0043565:sequence-specific DNA binding	GO:0005634:nucleus
Pp03_22312-5p	18783885	PRUPE_ppa013904mg	NCBI_Assembly:GCF_000346465.1	NW_006760268.1	-	10843011	10845632	2621	-	GO:0006457:protein folding	-	GO:0005737:cytoplasm
Pp03_22312-5p	18784142	PRUPE_ppa024838mg	NCBI_Assembly:GCF_000346465.1	NW_006760268.1	+	12967860	12968897	1037	-	GO:0010264:myo-inositol hexakisphosphate biosynthetic process	GO:0016491:oxidoreductase activity	-
Pp03_22312-5p	18784175	PRUPE_ppa016678mg	NCBI_Assembly:GCF_000346465.1	NW_006760268.1	+	13787524	13788661	1137	-	-	-	-
Pp03_22312-5p	18784211	PRUPE_ppa001157mg	NCBI_Assembly:GCF_000346465.1	NW_006760268.1	+	18766568	18769249	2681	-	-	GO:0004674:protein serine/threonine kinase activity;GO:0005524:ATP binding	-

Pp03_22312-5p	18784249	PRUPE_ppa017379mg	NCBI_Assembly:GCF_000346465.1	NW_006760268.1	-	13354729	13357839	3110	-	-	GO:0000166:nucleotide binding;GO:0003676:nucleic acid binding	-
Pp03_22312-5p	18784250	PRUPE_ppa006461mg	NCBI_Assembly:GCF_000346465.1	NW_006760268.1	-	18834674	18838207	3533	-	GO:0001731:formation of translation preinitiation complex;GO:0006446:regulation of translational initiation	GO:0003743:translation initiation factor activity	GO:0005852:eukaryotic translation initiation factor 3 complex;GO:0016282:eukaryotic 43S preinitiation complex;GO:0033290:eukaryotic 48S preinitiation complex
Pp03_22312-5p	18784330	PRUPE_ppa002530mg	NCBI_Assembly:GCF_000346465.1	NW_006760268.1	+	20635345	20638079	2734	-	-	GO:0004674:protein serine/threonine kinase activity;GO:0005524:ATP binding	-
Pp03_22312-5p	18784442	PRUPE_ppa015471mg	NCBI_Assembly:GCF_000346465.1	NW_006760268.1	-	11178551	11178907	356	-	-	-	-
Pp03_22312-5p	18784493	PRUPE_ppa022327mg	NCBI_Assembly:GCF_000346465.1	NW_006760268.1	+	1584241	1585824	1583	-	-	GO:0016788:hydrolase activity, acting on ester bonds	-
Pp03_22312-5p	18784577	PRUPE_ppa000601mg	NCBI_Assembly:GCF_000346465.1	NW_006760324.1	-	26436646	26447351	10705	pper03013:RNA transport;pper03008:Ribosome biogenesis in eukaryotes	GO:0006886:intracellular protein transport	-	GO:0005622:intracellular

Pp03_22312-5p	18784616	PRUPE_ppa024897mg	NCBI_Assembly:GCF_000346465.1	NW_006760324.1	+	15827686	15828480	794	-	GO:0000079:regulation of cyclin-dependent protein serine/threonine kinase activity	-	-	
Pp03_22312-5p	18784686	PRUPE_ppa016475mg	NCBI_Assembly:GCF_000346465.1	NW_006760324.1	-	10222607	10224046	1439	-	-	GO:0047134:protein-disulfide reductase activity	-	
Pp03_22312-5p	18784705	PRUPE_ppa008009mg	NCBI_Assembly:GCF_000346465.1	NW_006760324.1	-	23745160	23748070	2910	-	-	GO:0008270:zinc ion binding;GO:0016491:oxidoreductase activity	GO:0005777:peroxisome	
Pp03_22312-5p	18784717	PRUPE_ppa005140mg	NCBI_Assembly:GCF_000346465.1	NW_006760324.1	+	2249647	2253110	3463	-	-	GO:0015238:drug transmembrane transporter activity;GO:0015297:antiporter activity	GO:0016021:integral component of membrane	
Pp03_22312-5p	18784775	PRUPE_ppa006573mg	NCBI_Assembly:GCF_000346465.1	NW_006760324.1	-	15668344	15673521	5177	pper01100:Metabolic pathways;pper01200:Carbon metabolism;pper00280:Valine, leucine and isoleucine degradation;pper00410:beta-Alanine metabolism;pper00640:Propanoate metabolism	-	GO:0003824:catalytic activity	-	

Pp03_22312-5p	18784804	PRUPE_ppa002415mg	NCBI_Assembly:GCF_000346465.1	NW_006760324.1	+	22991870	22994907	3037	-	-	GO:0046872: metal ion binding	-
Pp03_22312-5p	18784806	PRUPE_ppa000178mg	NCBI_Assembly:GCF_000346465.1	NW_006760324.1	-	20485707	20494195	8488	-	GO:0006306: DNA methylation; GO:0030422: production of siRNA involved in RNA interference	-	-
Pp03_22312-5p	18784826	PRUPE_ppb022117mg	NCBI_Assembly:GCF_000346465.1	NW_006760324.1	-	8222803	8223427	624	-	-	-	-
Pp03_22312-5p	18784859	PRUPE_ppa000654mg	NCBI_Assembly:GCF_000346465.1	NW_006760324.1	+	19610286	19614023	3737	-	-	GO:0005524: ATP binding;GO:0016787:hydrolase activity;GO:0046872:metal ion binding	GO:0005783: endoplasmic reticulum;GO:0016021:integral component of membrane
Pp03_22312-5p	18784910	PRUPE_ppa014292mg	NCBI_Assembly:GCF_000346465.1	NW_006760324.1	-	20216056	20216725	669	-	-	-	-
Pp03_22312-5p	18784925	PRUPE_ppa011624mg	NCBI_Assembly:GCF_000346465.1	NW_006760324.1	-	17258588	17260020	1432	-	-	GO:0008270: zinc ion binding	-
Pp03_22312-5p	18784994	PRUPE_ppa000070mg	NCBI_Assembly:GCF_000346465.1	NW_006760324.1	-	20186898	20196308	9410	-	GO:0006396: RNA processing	GO:0003723: RNA binding;GO:0004386:helicase activity;GO:0004525:ribonuclease III activity;GO:0005524:ATP binding	-
Pp03_22312-5p	18785010	PRUPE_ppa026782mg	NCBI_Assembly:GCF_000346465.1	NW_006760324.1	-	24963203	24964267	1064	-	-	-	-

Pp03_22312-5p	18785017	PRUPE_ppa014538mg	NCBI_Assembly:GCF_000346465.1	NW_006760324.1	-	19423955	19426039	2084	-	-	-	GO:0005739:mitochondrion;GO:0016020:membrane
Pp03_22312-5p	18785100	PRUPE_ppa025584mg	NCBI_Assembly:GCF_000346465.1	NW_006760324.1	-	4339291	4340882	1591	-	-	-	-
Pp03_22312-5p	18785234	PRUPE_ppa026205mg	NCBI_Assembly:GCF_000346465.1	NW_006760324.1	-	9961956	9963873	1917	-	-	-	-
Pp03_22312-5p	18785270	PRUPE_ppa008912mg	NCBI_Assembly:GCF_000346465.1	NW_006760324.1	-	1701684	1704264	2580	-	-	GO:0005509:calcium ion binding;GO:0005544:calcium-dependent phospholipid binding	-
Pp03_22312-5p	18785291	PRUPE_ppa016958mg	NCBI_Assembly:GCF_000346465.1	NW_006760324.1	+	16589749	16591386	1637	-	-	GO:0003824:catalytic activity;GO:0050662:coenzyme binding	-
Pp03_22312-5p	18785320	PRUPE_ppb017369mg	NCBI_Assembly:GCF_000346465.1	NW_006760324.1	+	25426680	25427829	1149	-	GO:0016192:vesicle-mediated transport	-	GO:0016021:integral component of membrane

Pp03_22312-5p	18785329	PRUPE_ppa008488mg	NCBI_Assembly:GCF_000346465.1	NW_006760324.1	+	22784179	22787716	3537	-	GO:0006024:glycosaminoglycan biosynthetic process;GO:0009617:response to bacterium;GO:0010087:phloem or xylem histogenesis;GO:0015012:heparan sulfate proteoglycan biosynthetic process;GO:0016337:single organismal cell-cell adhesion;GO:0045087:innate immune response	GO:0035251:UDP-glucosyltransferase activity	GO:0005768:endosome;GO:0005802:trans-Golgi network;GO:0016021:integral component of membrane
Pp03_22312-5p	18785343	PRUPE_ppa008981mg	NCBI_Assembly:GCF_000346465.1	NW_006760324.1	-	23915912	23918570	2658	-	-	-	-
Pp03_22312-5p	18785397	PRUPE_ppa018704mg	NCBI_Assembly:GCF_000346465.1	NW_006760324.1	-	21286241	21288915	2674	-	GO:0006351:transcription, DNA-templated	GO:0003677:DNA binding;GO:0003700:sequence-specific DNA binding transcription factor activity	GO:0005634:nucleus

Pp03_22312-5p	18785401	PRUPE_ppa006562mg	NCBI_Assembly:GCF_000346465.1	NW_006760324.1	+		18886525	18889364	2839	-	GO:0006869:lipid transport;GO:0048513:organ development	GO:0004842:ubiquitin-protein transferase activity	-
Pp03_22312-5p	18785491	PRUPE_ppa017469mg	NCBI_Assembly:GCF_000346465.1	NW_006760324.1	-		10763716	10769690	5974	-	-	GO:0008270:zinc ion binding	-
Pp03_22312-5p	18785537	PRUPE_ppa009785mg	NCBI_Assembly:GCF_000346465.1	NW_006760324.1	+		23697064	23698935	1871	-	GO:0033473:indoleacetic acid conjugate metabolic process;GO:0048367:shoot system development	GO:0080030:methyl indole-3-acetate esterase activity	-
Pp03_22312-5p	18785569	PRUPE_ppa006367mg	NCBI_Assembly:GCF_000346465.1	NW_006760324.1	-		10125057	10128957	3900	-	-	-	-
Pp03_22312-5p	18785713	PRUPE_ppa014949mg	NCBI_Assembly:GCF_000346465.1	NW_006760324.1	+		20812804	20813073	269	pper04075:Plant hormone signal transduction	-	-	-
Pp03_22312-5p	18785723	PRUPE_ppa021815mg	NCBI_Assembly:GCF_000346465.1	NW_006760324.1	-		26568718	26569346	628	-	GO:0006952:defense response;GO:0009607:response to biotic stimulus	-	-
Pp03_22312-5p	18785758	PRUPE_ppa016876mg	NCBI_Assembly:GCF_000346465.1	NW_006760324.1	-		20316948	20318260	1312	-	GO:0006351:transcription, DNA-templated;GO:0006355:regulation of transcription, DNA-templated	GO:0003677:DNA binding	GO:0005634:nucleus

Pp03_22312-5p	18785770	PRUPE_ppa013989mg	NCBI_Assembly:GCF_000346465.1	NW_006760324.1	+	14930273	14932313	2040	pper03010:Ribosome	GO:0006412:translation	GO:0003735:structural constituent of ribosome	GO:0022625:cytosolic large ribosomal subunit
Pp03_22312-5p	18785822	PRUPE_ppa026063mg	NCBI_Assembly:GCF_000346465.1	NW_006760324.1	-	21124577	21131554	6977	-	-	GO:0008270:zinc ion binding	-
Pp03_22312-5p	18785857	PRUPE_ppa007356mg	NCBI_Assembly:GCF_000346465.1	NW_006760324.1	-	4940582	4943444	2862	-	-	-	-
Pp03_22312-5p	18785910	PRUPE_ppa015423mg	NCBI_Assembly:GCF_000346465.1	NW_006760324.1	-	20091809	20094922	3113	pper01100:Metabolic pathways;pper01110:Biosynthesis of secondary metabolites;pper00940:Phenylpropanoid biosynthesis	-	GO:0008171:O-methyltransferase activity	-
Pp03_22312-5p	18785992	PRUPE_ppa019468mg	NCBI_Assembly:GCF_000346465.1	NW_006760324.1	+	3038170	3038370	200	-	GO:0006355:regulation of transcription, DNA-templated	GO:0003677:DNA binding	-
Pp03_22312-5p	18785993	PRUPE_ppa010645mg	NCBI_Assembly:GCF_000346465.1	NW_006760324.1	+	24736525	24737863	1338	-	-	-	-
Pp03_22312-5p	18786019	PRUPE_ppb022202mg	NCBI_Assembly:GCF_000346465.1	NW_006760324.1	+	6359854	6362609	2755	-	-	-	-
Pp03_22312-5p	18786025	PRUPE_ppa022875mg	NCBI_Assembly:GCF_000346465.1	NW_006760324.1	+	4246608	4249933	3325	-	-	GO:0003676:nucleic acid binding	-

Pp03_22312-5p	18786056	PRUPE_ppa022332mg	NCBI_Assembly:GCF_000346465.1	NW_006760324.1	-	9648689	9650128	1439	-	-	GO:0016747: transferase activity, transferring acyl groups other than amino-acyl groups	-
Pp03_22312-5p	18786078	PRUPE_ppa024455mg	NCBI_Assembly:GCF_000346465.1	NW_006760324.1	-	18734772	18736115	1343	-	GO:0006629: lipid metabolic process	GO:0016491: oxidoreductase activity	GO:0016021: integral component of membrane
Pp03_22312-5p	18786081	PRUPE_ppa011367mg	NCBI_Assembly:GCF_000346465.1	NW_006760324.1	-	24997064	24999001	1937	-	-	-	-
Pp03_22312-5p	18786133	PRUPE_ppa018146mg	NCBI_Assembly:GCF_000346465.1	NW_006760324.1	+	20779408	20780047	639	-	GO:0042545: cell wall modification	GO:0030599: pectinesterase activity;GO:0045330:aspartyl esterase activity	GO:0005618: cell wall
Pp03_22312-5p	18786146	PRUPE_ppa004462mg	NCBI_Assembly:GCF_000346465.1	NW_006760324.1	-	11699133	11706417	7284	-	GO:0006869: lipid transport	-	GO:0005634: nucleus;GO:0005829:cytosol
Pp03_22312-5p	18786168	PRUPE_ppa012544mg	NCBI_Assembly:GCF_000346465.1	NW_006760324.1	-	4338577	4339116	539	-	-	-	-
Pp03_22312-5p	18786212	PRUPE_ppa000384mg	NCBI_Assembly:GCF_000346465.1	NW_006760324.1	+	26332628	26341713	9085	-	-	-	-
Pp03_22312-5p	18786232	PRUPE_ppb014513mg	NCBI_Assembly:GCF_000346465.1	NW_006760324.1	-	14298977	14299858	881	-	-	-	-
Pp03_22312-5p	18786279	PRUPE_ppa023957mg	NCBI_Assembly:GCF_000346465.1	NW_006760324.1	+	24669930	24670862	932	-	-	GO:0016787: hydrolase activity	-

Pp03_22312-5p	18786286	PRUPE_ppa019267mg	NCBI_Assembly:GCF_000346465.1	NW_006760324.1	-	24722530	24722920	390	-	GO:0006351: transcription, DNA-templated;GO:0006355:regulation of transcription, DNA-templated	GO:0003677: DNA binding	GO:0005634: nucleus
Pp03_22312-5p	18786331	PRUPE_ppa023715mg	NCBI_Assembly:GCF_000346465.1	NW_006760324.1	+	22091931	22093496	1565	-	-	-	GO:0005886: plasma membrane;GO:0016021:integral component of membrane
Pp03_22312-5p	18786487	PRUPE_ppa000876mg	NCBI_Assembly:GCF_000346465.1	NW_006760324.1	+	24663397	24666853	3456	-	GO:0048445: carpel morphogenesis	GO:0016772: transferase activity, transferring phosphorus-containing groups	-
Pp03_22312-5p	18786502	PRUPE_ppa021828mg	NCBI_Assembly:GCF_000346465.1	NW_006760324.1	-	4759211	4759729	518	-	-	-	-
Pp03_22312-5p	18786620	PRUPE_ppa021336mg	NCBI_Assembly:GCF_000346465.1	NW_006760324.1	+	1887404	1888786	1382	-	-	-	-
Pp03_22312-5p	18786639	PRUPE_ppa009327mg	NCBI_Assembly:GCF_000346465.1	NW_006760324.1	+	8261498	8265486	3988	pper03030:DNA replication	-	GO:0003723: RNA binding;GO:0004523:RNA-DNA hybrid ribonuclease activity	-
Pp03_22312-5p	18786675	PRUPE_ppa021677mg	NCBI_Assembly:GCF_000346465.1	NW_006760324.1	-	15938099	15938374	275	-	-	-	-
Pp03_22312-5p	18786683	PRUPE_ppa001153m2g	NCBI_Assembly:GCF_000346465.1	NW_006760324.1	+	4202330	4203255	925	-	-	-	-

Pp03_22312-5p	18786759	PRUPE_ppa013766mg	NCBI_Assembly:GCF_000346465.1	NW_006760324.1	+	19596604	19596982	378	-	-	-	-
Pp03_22312-5p	18786762	PRUPE_ppa002700mg	NCBI_Assembly:GCF_000346465.1	NW_006760324.1	-	1220843	1227605	6762	-	-	GO:0003993:acid phosphatase activity;GO:0046872:metal ion binding	-
Pp03_22312-5p	18786771	PRUPE_ppa006189mg	NCBI_Assembly:GCF_000346465.1	NW_006760324.1	-	18148526	18151861	3335	pper01100:Metabolic pathways;pper00500:Starch and sucrose metabolism	GO:0000023:maltose metabolic process;GO:0000272:polysaccharide catabolic process;GO:0019252:starch biosynthetic process	GO:0016161:beta-amylase activity	GO:0009570:chloroplast stroma
Pp03_22312-5p	18786838	PRUPE_ppa005111mg	NCBI_Assembly:GCF_000346465.1	NW_006760324.1	+	18576923	18578834	1911	-	-	-	-
Pp03_22312-5p	18786903	PRUPE_ppb011814mg	NCBI_Assembly:GCF_000346465.1	NW_006760324.1	+	22965646	22966932	1286	-	-	-	-
Pp03_22312-5p	18786980	PRUPE_ppa018219mg	NCBI_Assembly:GCF_000346465.1	NW_006760324.1	+	23011740	23012997	1257	-	-	-	-
Pp03_22312-5p	18787043	PRUPE_ppa005449mg	NCBI_Assembly:GCF_000346465.1	NW_006760324.1	-	15802007	15805683	3676	-	-	-	-
Pp03_22312-5p	18787051	PRUPE_ppa019679mg	NCBI_Assembly:GCF_000346465.1	NW_006760324.1	-	19297135	19298789	1654	-	-	-	-

Pp03_22312-5p	18787060	PRUPE_ppa010887mg	NCBI_Assembly:GCF_000346465.1	NW_006760324.1	+	24727554	24729143	1589	-	-	GO:0005506:iron ion binding;GO:0016706:oxidoreductase activity, acting on paired donors, with incorporation or reduction of molecular oxygen, 2-oxoglutarate as one donor, and incorporation of one atom each of oxygen into both donors;GO:0031418:L-ascorbic acid binding	-
Pp03_22312-5p	18787133	PRUPE_ppa010851mg	NCBI_Assembly:GCF_000346465.1	NW_006760324.1	-	9921736	9923911	2175	pper03010:Ribosome	GO:0006412:translation	GO:0003735:structural constituent of ribosome	GO:0005840:ribosome
Pp03_22312-5p	18787139	PRUPE_ppb018756mg	NCBI_Assembly:GCF_000346465.1	NW_006760324.1	+	5654999	5655571	572	-	GO:0007165:signal transduction	-	-
Pp03_22312-5p	18787167	PRUPE_ppa005815mg	NCBI_Assembly:GCF_000346465.1	NW_006760324.1	+	6019663	6024299	4636	pper04144:Endocytosis	-	GO:0032266:phosphatidylinositol-3-phosphate binding	GO:0000814:ESCRT II complex
Pp03_22312-5p	18787226	PRUPE_ppa023280mg	NCBI_Assembly:GCF_000346465.1	NW_006760324.1	+	7981115	7988178	7063	-	-	-	-
Pp03_22312-5p	18787266	PRUPE_ppa025757mg	NCBI_Assembly:GCF_000346465.1	NW_006760324.1	-	7992090	7992810	720	-	-	-	-

Pp03_22312-5p	18787351	PRUPE_ppa006601mg	NCBI_Assembly:GCF_000346465.1	NW_006760324.1	-		12972399	12976543	4144	-	-	-	-
Pp03_22312-5p	18788553	PRUPE_ppa005965mg	NCBI_Assembly:GCF_000346465.1	NW_006760385.1	+		37973077	37977545	4468	-	-	-	-
Pp03_22312-5p	18788556	PRUPE_ppa024452mg	NCBI_Assembly:GCF_000346465.1	NW_006760385.1	-		41778787	41779251	464	-	-	GO:0003676:nucleic acid binding	-
Pp03_22312-5p	18788587	PRUPE_ppa026963mg	NCBI_Assembly:GCF_000346465.1	NW_006760385.1	+		2337870	2339087	1217	-	-	GO:0003677:DNA binding	-
Pp03_22312-5p	18788617	PRUPE_ppa004032mg	NCBI_Assembly:GCF_000346465.1	NW_006760385.1	+		20252596	20258405	5809	-	-	-	-
Pp03_22312-5p	18788682	PRUPE_ppa000690mg	NCBI_Assembly:GCF_000346465.1	NW_006760385.1	+		8093363	8099939	6576	-	-	GO:0003677:DNA binding	GO:0005634:nucleus
Pp03_22312-5p	18788698	PRUPE_ppa007933mg	NCBI_Assembly:GCF_000346465.1	NW_006760385.1	+		36230874	36232444	1570	-	-	-	-
Pp03_22312-5p	18788723	PRUPE_ppa021477mg	NCBI_Assembly:GCF_000346465.1	NW_006760385.1	-		40372852	40374427	1575	-	-	-	-
Pp03_22312-5p	18788745	PRUPE_ppa003930mg	NCBI_Assembly:GCF_000346465.1	NW_006760385.1	+		45330720	45340306	9586	-	GO:0009793:embryo development ending in seed dormancy	-	GO:0009507:chloroplast
Pp03_22312-5p	18788781	PRUPE_ppa024419mg	NCBI_Assembly:GCF_000346465.1	NW_006760385.1	-		39110153	39112555	2402	-	-	GO:0004674:protein serine/threonine kinase activity;GO:0005524:ATP binding;GO:0030246:carbohydrate binding	-

Pp03_22312-5p	18788800	PRUPE_ppa005208mg	NCBI_Assembly:GCF_000346465.1	NW_006760385.1	-	30386456	30388356	1900	pper01100:Metabolic pathways;pper00500:Starch and sucrose metabolism;pper00040:Pentose and glucuronate interconversions	GO:0042545:cell wall modification;GO:0045490:pectin catabolic process	GO:0004857:enzyme inhibitor activity;GO:0030599:pectinesterase activity;GO:0045330:aspartyl esterase activity	GO:0005618:cell wall
Pp03_22312-5p	18788856	PRUPE_ppa002827mg	NCBI_Assembly:GCF_000346465.1	NW_006760385.1	-	9613794	9618828	5034	pper03008:Ribosome biogenesis in eukaryotes	GO:0000478:endonucleolytic cleavage involved in rRNA processing;GO:0009220:pyrimidine ribonucleotide biosynthetic process;GO:0009909:regulation of flower development	-	-
Pp03_22312-5p	18788941	PRUPE_ppa008904mg	NCBI_Assembly:GCF_000346465.1	NW_006760385.1	+	31779319	31781934	2615	-	GO:0010207:photosystem II assembly	-	-
Pp03_22312-5p	18789072	PRUPE_ppb015618mg	NCBI_Assembly:GCF_000346465.1	NW_006760385.1	-	46849364	46853355	3991	-	GO:0007165:signal transduction	GO:0043531:ADP binding	-
Pp03_22312-5p	18789148	PRUPE_ppa012638mg	NCBI_Assembly:GCF_000346465.1	NW_006760385.1	+	36004896	36006401	1505	pper04120:Ubiquitin mediated proteolysis;pper03013:RNA transport	-	GO:0016874:ligase activity;GO:0019789:SUMO transferase activity	-

Pp03_22312-5p	18789190	PRUPE_ppa000584mg	NCBI_Assembly:GCF_000346465.1	NW_006760385.1	+		37673424	37680475	7051	-	GO:0006511:ubiquitin-dependent protein catabolic process;GO:0007062:sister chromatid cohesion;GO:0009640:photomorphogenesis;GO:0009845:seed germination;GO:0009880:embryonic pattern specification;GO:0009909:regulation of flower development;GO:0010072:primary shoot apical meristem specification;GO:0010162:seed dormancy process;GO:0010182:sugar mediated signaling pathway;GO:0010228:vegetative to reproductive phase	GO:0004843:ubiquitin-specific protease activity	GO:0005730:nucleolus
Pp03_22312-5p	18789875	PRUPE_ppa026955mg	NCBI_Assembly:GCF_000346465.1	NW_006760385.1	+		33853255	33853783	528	-	-	GO:0009055:electron carrier activity	-

Pp03_22312-5p	18789885	PRUPE_ppa020682mg	NCBI_Assembly:GCF_000346465.1	NW_006760385.1	+	567987	570525	2538	-	GO:0042545:cell wall modification	GO:0030599:pectinesterase activity;GO:0045330:aspartyl esterase activity	GO:0005618:cell wall
Pp03_22312-5p	18789954	PRUPE_ppa007506mg	NCBI_Assembly:GCF_000346465.1	NW_006760385.1	-	21294877	21298983	4106	-	-	-	-
Pp03_22312-5p	18789978	PRUPE_ppa022752mg	NCBI_Assembly:GCF_000346465.1	NW_006760385.1	-	46122183	46124532	2349	-	-	GO:0004497:monooxygenase activity;GO:0005506:iron ion binding;GO:0016705:oxidoreductase activity, acting on paired donors, with incorporation or reduction of molecular oxygen;GO:0020037:heme binding	-
Pp03_22312-5p	18790073	PRUPE_ppa006563mg	NCBI_Assembly:GCF_000346465.1	NW_006760385.1	+	31650293	31656168	5875	-	GO:0006479:protein methylation	GO:0008168:methyltransferase activity	-
Pp03_22312-5p	18790119	PRUPE_ppa000590mg	NCBI_Assembly:GCF_000346465.1	NW_006760385.1	+	6257961	6263782	5821	-	-	GO:0004712:protein serine/threonine/tyrosine kinase activity;GO:0005524:ATP binding	-

Pp03_22312-5p	18790125	PRUPE_ppa001427mg	NCBI_Assembly:GCF_000346465.1	NW_006760385.1	-	597425	600122	2697	-	-	GO:0004674:protein serine/threonine kinase activity;GO:0005524:ATP binding	GO:0005886:plasma membrane
Pp03_22312-5p	18790142	PRUPE_ppa016591mg	NCBI_Assembly:GCF_000346465.1	NW_006760385.1	-	22016878	22018174	1296	-	-	-	-
Pp03_22312-5p	18790173	PRUPE_ppa018609mg	NCBI_Assembly:GCF_000346465.1	NW_006760385.1	-	23827272	23831962	4690	pper01100:Metabolic pathways;pper00230:Purine metabolism;pper00240:Pyrimidine metabolism;pper03030:DNA replication	GO:0000724:double-strand break repair via homologous recombination;GO:0006270:DNA replication initiation;GO:0006275:regulation of DNA replication;GO:0006306:DNA methylation;GO:0008283:cell proliferation;GO:0009909:regulation of flower development;GO:0010389:regulation of G2/M transition of mitotic cell cycle;GO:0016458:gene silencing;GO:0051567:histone H3-K9 methylation	GO:0003677:DNA binding;GO:0003887:DNA-directed DNA polymerase activity	GO:0005634:nucleus;GO:0005739:mitochondrion

Pp03_22312-5p	18790232	PRUPE_ppa019388mg	NCBI_Assembly:GCF_000346465.1	NW_006760385.1	-	35465276	35466556	1280	-	-	-	-
Pp03_22312-5p	18790269	PRUPE_ppa019144mg	NCBI_Assembly:GCF_000346465.1	NW_006760385.1	+	35013898	35017696	3798	-	-	GO:0004970: ionotropic glutamate receptor activity	GO:0016021: integral component of membrane
Pp03_22312-5p	18790275	PRUPE_ppa016821mg	NCBI_Assembly:GCF_000346465.1	NW_006760385.1	-	22962054	22966764	4710	-	-	-	-
Pp03_22312-5p	18790281	PRUPE_ppa005300mg	NCBI_Assembly:GCF_000346465.1	NW_006760385.1	+	26360705	26364644	3939	-	-	-	-
Pp03_22312-5p	18790331	PRUPE_ppa012676mg	NCBI_Assembly:GCF_000346465.1	NW_006760385.1	+	9554566	9555468	902	-	GO:0006952: defense response;GO:0009607:response to biotic stimulus	-	-
Pp03_22312-5p	18790332	PRUPE_ppa011014mg	NCBI_Assembly:GCF_000346465.1	NW_006760385.1	+	27134558	27136178	1620	-	-	GO:0003723: RNA binding;GO:0033897:ribonuclease T2 activity	-
Pp03_22312-5p	18790430	PRUPE_ppa003242mg	NCBI_Assembly:GCF_000346465.1	NW_006760385.1	+	25516684	25520321	3637	pper03040:Spliceosome	-	-	-
Pp03_22312-5p	18790469	PRUPE_ppa1027170mg	NCBI_Assembly:GCF_000346465.1	NW_006760385.1	-	27292942	27294435	1493	-	-	-	-
Pp03_22312-5p	18790477	PRUPE_ppa012471mg	NCBI_Assembly:GCF_000346465.1	NW_006760385.1	+	35395745	35396452	707	-	-	-	-
Pp03_22312-5p	18790505	PRUPE_ppb024350mg	NCBI_Assembly:GCF_000346465.1	NW_006760385.1	-	6480170	6481837	1667	-	-	GO:0008234: cysteine-type peptidase activity	-
Pp03_22312-5p	18790577	PRUPE_ppa026477mg	NCBI_Assembly:GCF_000346465.1	NW_006760385.1	-	22509829	22511452	1623	-	-	GO:0003676: nucleic acid binding	-

Pp03_22312-5p	18790606	PRUPE_ppa003541mg	NCBI_Assembly:GCF_000346465.1	NW_006760385.1	-		33734171	33737797	3626	-	GO:0006351:transcription, DNA-templated;GO:0006355:regulation of transcription, DNA-templated	GO:0003677:DNA binding	GO:0005634:nucleus
Pp03_22312-5p	18790620	PRUPE_ppa017329mg	NCBI_Assembly:GCF_000346465.1	NW_006760385.1	+		23281910	23283517	1607	-	-	GO:0004497:monooxygenase activity;GO:0005506:iron ion binding;GO:0016705:oxidoreductase activity, acting on paired donors, with incorporation or reduction of molecular oxygen;GO:0020037:heme binding	-
Pp03_22312-5p	18790689	PRUPE_ppa002609mg	NCBI_Assembly:GCF_000346465.1	NW_006760385.1	-		303669	309654	5985	-	-	-	-
Pp03_22312-5p	18790702	PRUPE_ppa000323mg	NCBI_Assembly:GCF_000346465.1	NW_006760385.1	+		43176002	43196076	20074	pper04141:Protein processing in endoplasmic reticulum	GO:0006486:protein glycosylation	GO:0003980:UDP-glucose:glycoprotein glucosyltransferase activity	-
Pp03_22312-5p	18790763	PRUPE_ppa006233mg	NCBI_Assembly:GCF_000346465.1	NW_006760385.1	-		44905019	44908079	3060	-	-	GO:0003746:translation elongation factor activity	-

Pp03_22312-5p	18790766	PRUPE_ppa022821mg	NCBI_Assembly:GCF_000346465.1	NW_006760385.1	-	29520712	29521497	785	-	-	GO:0030247: polysaccharide binding	-
Pp03_22312-5p	18790812	PRUPE_ppa006218mg	NCBI_Assembly:GCF_000346465.1	NW_006760385.1	+	42590792	42597221	6429	-	-	GO:0004672: protein kinase activity;GO:0005524:ATP binding	-
Pp03_22312-5p	18790826	PRUPE_ppa024547mg	NCBI_Assembly:GCF_000346465.1	NW_006760385.1	-	11116112	11117344	1232	-	-	-	-
Pp03_22312-5p	18790882	PRUPE_ppa017169mg	NCBI_Assembly:GCF_000346465.1	NW_006760385.1	-	5133935	5135264	1329	-	-	GO:0003676: nucleic acid binding	-
Pp03_22312-5p	18790883	PRUPE_ppa020847mg	NCBI_Assembly:GCF_000346465.1	NW_006760385.1	+	28150971	28152566	1595	-	-	-	-
Pp03_22312-5p	18790906	PRUPE_ppa007940mg	NCBI_Assembly:GCF_000346465.1	NW_006760385.1	+	7671638	7673518	1880	-	-	-	GO:0016021: integral component of membrane
Pp03_22312-5p	18790930	PRUPE_ppa020259mg	NCBI_Assembly:GCF_000346465.1	NW_006760385.1	-	18692787	18693179	392	-	-	-	-
Pp03_22312-5p	18791005	PRUPE_ppa015249mg	NCBI_Assembly:GCF_000346465.1	NW_006760385.1	-	27642887	27643590	703	-	GO:0006351: transcription, DNA-templated;GO:0006355:regulation of transcription, DNA-templated	GO:0003677: DNA binding	GO:0005634: nucleus
Pp03_22312-5p	18791024	PRUPE_ppa025315mg	NCBI_Assembly:GCF_000346465.1	NW_006760385.1	-	6924475	6924906	431	-	-	-	-
Pp03_22312-5p	18791198	PRUPE_ppa024413mg	NCBI_Assembly:GCF_000346465.1	NW_006760385.1	+	43990695	43991615	920	-	-	-	-
Pp03_22312-5p	18791224	PRUPE_ppa025796mg	NCBI_Assembly:GCF_000346465.1	NW_006760385.1	+	12014125	12015238	1113	-	-	-	-

Pp03_22312-5p	18791235	PRUPE_ppa023941mg	NCBI_Assembly:GCF_000346465.1	NW_006760385.1	+	24500911	24503416	2505	-	-	GO:0000166:nucleotide binding;GO:0003676:nucleic acid binding	-	
Pp03_22312-5p	18791236	PRUPE_ppa019694mg	NCBI_Assembly:GCF_000346465.1	NW_006760385.1	+	23454123	23455702	1579	-	-	GO:0005506:iron ion binding;GO:0016705:oxidoreductase activity, acting on paired donors, with incorporation or reduction of molecular oxygen;GO:0020037:heme binding	-	
Pp03_22312-5p	18791352	PRUPE_ppa019926mg	NCBI_Assembly:GCF_000346465.1	NW_006760385.1	+	3532959	3533758	799	-	-	-	-	
Pp03_22312-5p	18791371	PRUPE_ppa022670mg	NCBI_Assembly:GCF_000346465.1	NW_006760385.1	-	38858720	38860445	1725	-	-	-	-	
Pp03_22312-5p	18791427	PRUPE_ppa009844mg	NCBI_Assembly:GCF_000346465.1	NW_006760385.1	-	19536646	19537921	1275	pper01110:Biosynthesis of secondary metabolites;pper00520:Amino sugar and nucleotide sugar metabolism	GO:0005975:carbohydrate metabolic process;GO:0006032:chitin catabolic process;GO:0016998:cell wall macromolecule catabolic process	GO:0004568:chitinase activity;GO:0008061:chitin binding	-	
Pp03_22312-5p	18791448	PRUPE_ppa018630mg	NCBI_Assembly:GCF_000346465.1	NW_006760385.1	-	3612879	3615121	2242	-	GO:0006810:transport	-	GO:0016021:integral component of membrane	

Pp03_22312-5p	18791451	PRUPE_ppa011820mg	NCBI_Assembly:GCF_000346465.1	NW_006760385.1	+	35874450	35876756	2306	-	-	-	GO:0005634:nucleus
Pp03_22312-5p	18791477	PRUPE_ppa003120mg	NCBI_Assembly:GCF_000346465.1	NW_006760385.1	+	26509508	26512411	2903	-	-	-	-
Pp03_22312-5p	18791482	PRUPE_ppa023214mg	NCBI_Assembly:GCF_000346465.1	NW_006760385.1	+	4970031	4973440	3409	-	-	GO:0004518:nuclease activity	-
Pp03_22312-5p	18791520	PRUPE_ppa004366mg	NCBI_Assembly:GCF_000346465.1	NW_006760385.1	+	26370161	26374199	4038	-	-	-	-
Pp03_22312-5p	18791543	PRUPE_ppa009221mg	NCBI_Assembly:GCF_000346465.1	NW_006760385.1	+	31240229	31242358	2129	-	GO:0006270:DNA replication initiation;GO:0006974:cellular response to DNA damage stimulus;GO:0007049:cell cycle;GO:0010228:vegetative to reproductive phase transition of meristem;GO:0048478:replication fork protection;GO:0051726:regulation of cell cycle	GO:0003676:nucleic acid binding;GO:0008270:zinc ion binding	GO:0005634:nucleus
Pp03_22312-5p	18791544	PRUPE_ppa010607mg	NCBI_Assembly:GCF_000346465.1	NW_006760385.1	+	30634218	30636668	2450	-	-	-	-

Pp03_22312-5p	18791553	PRUPE_ppa007067mg	NCBI_Assembly:GCF_000346465.1	NW_006760385.1	+	43379055	43381967	2912	-	-	GO:0016706: oxidoreductase activity, acting on paired donors, with incorporation or reduction of molecular oxygen, 2-oxoglutarate as one donor, and incorporation of one atom each of oxygen into both donors	-
Pp03_22312-5p	18791556	PRUPE_ppa026463mg	NCBI_Assembly:GCF_000346465.1	NW_006760385.1	+	42322104	42322655	551	-	-	-	-
Pp03_22312-5p	18791597	PRUPE_ppa015275mg	NCBI_Assembly:GCF_000346465.1	NW_006760385.1	+	34927227	34929454	2227	-	-	-	GO:0005777: peroxisome
Pp03_22312-5p	18791605	PRUPE_ppa006180mg	NCBI_Assembly:GCF_000346465.1	NW_006760385.1	-	4533566	4536836	3270	-	-	-	-
Pp03_22312-5p	18791663	PRUPE_ppa001669mg	NCBI_Assembly:GCF_000346465.1	NW_006760385.1	+	40710845	40715088	4243	pper01100:Metabolic pathways;pper04144:Endocytosis;pper00562:Inositol phosphate metabolism;pper04070:Phosphatidylinositol signaling system	-	GO:0016307: phosphatidylinositol phosphate kinase activity	-

Pp03_22312-5p	18791703	PRUPE_ppa004739mg	NCBI_Assembly:GCF_000346465.1	NW_006760385.1	-	42797851	42801888	4037	-	-	-	-
Pp03_22312-5p	18791717	PRUPE_ppa016183mg	NCBI_Assembly:GCF_000346465.1	NW_006760385.1	+	26421403	26423287	1884	-	-	-	-
Pp03_22312-5p	18791720	PRUPE_ppa026837mg	NCBI_Assembly:GCF_000346465.1	NW_006760385.1	+	17533913	17537152	3239	-	-	-	-
Pp03_22312-5p	18791766	PRUPE_ppa018816mg	NCBI_Assembly:GCF_000346465.1	NW_006760385.1	+	28747030	28747619	589	-	GO:0006974:cellular response to DNA damage stimulus	GO:0008413:8-oxo-7,8-dihydroguanosine triphosphate pyrophosphatase activity;GO:0019177:dihydroneopterin triphosphate pyrophosphohydrolase activity	GO:0005829:cytosol
Pp03_22312-5p	18791885	PRUPE_ppa008231mg	NCBI_Assembly:GCF_000346465.1	NW_006760385.1	-	36300916	36301938	1022	pper04075:Plant hormone signal transduction	GO:0010423:negative regulation of brassinosteroid biosynthetic process	-	GO:0005829:cytosol
Pp03_22312-5p	18791886	PRUPE_ppa015752mg	NCBI_Assembly:GCF_000346465.1	NW_006760385.1	-	32705628	32707193	1565	-	-	-	-
Pp03_22312-5p	18792017	PRUPE_ppa000428mg	NCBI_Assembly:GCF_000346465.1	NW_006760385.1	-	44743760	44755455	11695	-	-	GO:0003676:nucleic acid binding;GO:0005524:ATP binding	-
Pp03_22312-5p	18792026	PRUPE_ppa019577mg	NCBI_Assembly:GCF_000346465.1	NW_006760385.1	+	34233294	34234772	1478	-	-	GO:0004190:aspartic-type endopeptidase activity	-

Pp03_22312-5p	18792037	PRUPE_ppa007453mg	NCBI_Assembly:GCF_000346465.1	NW_006760385.1	-		1313071	1317998	4927	-		GO:0009966: regulation of signal transduction	GO:0004674: protein serine/threonine kinase activity;GO:0005524:ATP binding	-
Pp03_22312-5p	18792055	PRUPE_ppa003585mg	NCBI_Assembly:GCF_000346465.1	NW_006760385.1	+		41165262	41168730	3468	-		GO:0006400: tRNA modification; GO:0006897: endocytosis	GO:0003924: GTPase activity;GO:0005525:GTP binding	GO:0005622: intracellular
Pp03_22312-5p	18792071	PRUPE_ppa008776mg	NCBI_Assembly:GCF_000346465.1	NW_006760385.1	+		40879489	40882662	3173	-		GO:0006457: protein folding;GO:0009793:embryo development ending in seed dormancy;GO:0010027:thylakoid membrane organization; GO:0010228: vegetative to reproductive phase transition of meristem;GO:0016226:iron-sulfur cluster assembly;GO:0048481:ovule development	GO:0000774: adenyl-nucleotide exchange factor activity;GO:0005507:copper ion binding	GO:0005759: mitochondrial matrix;GO:0009570:chloroplast stroma

Pp03_22312-5p	18792075	PRUPE_ppa003696mg	NCBI_Assembly:GCF_000346465.1	NW_006760385.1	+	36612429	36615348	2919	-	GO:0006352:DNA-templated transcription, initiation	GO:0001053:plastid sigma factor activity;GO:0003677:DNA binding;GO:0003700:sequence-specific DNA binding transcription factor activity;GO:0003899:DNA-directed RNA polymerase activity	-
Pp03_22312-5p	18792115	PRUPE_ppa011188mg	NCBI_Assembly:GCF_000346465.1	NW_006760385.1	-	360157	362554	2397	-	GO:0016192:vesicle-mediated transport	-	GO:0016021:integral component of membrane
Pp03_22312-5p	18792161	PRUPE_ppa005842mg	NCBI_Assembly:GCF_000346465.1	NW_006760385.1	-	32742295	32747092	4797	-	-	GO:0046872:metal ion binding	-
Pp03_22312-5p	18792265	PRUPE_ppa012115mg	NCBI_Assembly:GCF_000346465.1	NW_006760385.1	+	4860322	4863458	3136	-	-	-	-

Pp03_22312-5p	18792266	PRUPE_ppa011707mg	NCBI_Assembly:GCF_000346465.1	NW_006760385.1	-	27234334	27235038	704	pper01100:Metabolic pathways;pper00195:Photosynthesis	GO:0006098:pentose-phosphate shunt;GO:0009767:photosynthetic electron transport chain	GO:0005509:calcium ion binding;GO:0045156:electron transporter, transferring electrons within the cyclic electron transport pathway of photosynthesis activity	GO:0009344:nitrite reductase complex [NAD(P)H];GO:0009543:chloroplast thylakoid lumen;GO:0009654:photosystem II oxygen evolving complex;GO:0019898:extrinsic component of membrane
Pp03_22312-5p	18792281	PRUPE_ppa024231mg	NCBI_Assembly:GCF_000346465.1	NW_006760385.1	-	12795066	12797665	2599	-	-	-	-
Pp03_22312-5p	18792353	PRUPE_ppa022707mg	NCBI_Assembly:GCF_000346465.1	NW_006760385.1	+	32065518	32069471	3953	pper01100:Metabolic pathways;pper00600:Sphingolipid metabolism	-	-	-
Pp03_22312-5p	18792378	PRUPE_ppa008151mg	NCBI_Assembly:GCF_000346465.1	NW_006760385.1	-	1536199	1539271	3072	-	GO:0006302:double-strand break repair;GO:0010212:response to ionizing radiation;GO:0051026:chiasma assembly	GO:0003677:DNA binding;GO:0005524:ATP binding;GO:0008094:DNA-dependent ATPase activity	GO:0005634:nucleus
Pp03_22312-5p	18792404	PRUPE_ppa016030mg	NCBI_Assembly:GCF_000346465.1	NW_006760385.1	-	26880399	26881583	1184	-	GO:0009416:response to light stimulus	-	-
Pp03_22312-5p	18792408	PRUPE_ppa006944mg	NCBI_Assembly:GCF_000346465.1	NW_006760385.1	+	35673079	35674326	1247	-	-	-	-

Pp03_22312-5p	18792424	PRUPE_ppa005580mg	NCBI_Assembly:GCF_000346465.1	NW_006760385.1	+	27169351	27171016	1665	-	GO:0006355:regulation of transcription, DNA-templated	GO:0003677:DNA binding	-
Pp03_22312-5p	18792497	PRUPE_ppa006845mg	NCBI_Assembly:GCF_000346465.1	NW_006760385.1	-	25470079	25472188	2109	-	GO:0000096:sulfur amino acid metabolic process;GO:0006546:glycine catabolic process;GO:0006636:unsaturated fatty acid biosynthetic process;GO:0006733:oxidoreduction coenzyme metabolic process;GO:0006766:vitamin metabolic process;GO:0008652:cellular amino acid biosynthetic process;GO:0009072:aromatic amino acid family metabolic process;GO:0009106:lipoate metabolic process;GO:0009108:coenzyme biosynthetic process;GO:	GO:0008270:zinc ion binding;GO:0035671:enone reductase activity;GO:0035798:2-alkenal reductase (NADP+) activity	GO:0009570:chloroplast stroma;GO:0009579:thylakoid;GO:0009941:chloroplast envelope;GO:0010319:stromule;GO:0048046:apoplast

Pp03_22312-5p	18792504	PRUPE_ppa020380mg	NCBI_Assembly:GCF_000346465.1	NW_006760385.1	+		10879364	10879926	562	-	GO:0030001: metal ion transport	GO:0046872: metal ion binding	-
Pp03_22312-5p	18792510	PRUPE_ppb018185mg	NCBI_Assembly:GCF_000346465.1	NW_006760385.1	-		19639001	19641659	2658	-	-	-	-
Pp03_22312-5p	18792546	PRUPE_ppa011061mg	NCBI_Assembly:GCF_000346465.1	NW_006760385.1	+		1377996	1380581	2585	-	-	GO:0003676: nucleic acid binding;GO:0008270:zinc ion binding	-
Pp03_22312-5p	18792636	PRUPE_ppa002600mg	NCBI_Assembly:GCF_000346465.1	NW_006760385.1	-		39222031	39224473	2442	-	GO:0048268: clathrin coat assembly	GO:0005545: 1-phosphatidylinositol binding	GO:0030136: clathrin-coated vesicle
Pp03_22312-5p	18792659	PRUPE_ppa023525mg	NCBI_Assembly:GCF_000346465.1	NW_006760385.1	-		38712607	38713608	1001	-	-	-	-
Pp03_22312-5p	18792701	PRUPE_ppa023819mg	NCBI_Assembly:GCF_000346465.1	NW_006760385.1	+		44578178	44586983	8805	-	GO:0007165: signal transduction	GO:0043531: ADP binding	-
Pp03_22312-5p	18792719	PRUPE_ppa011980mg	NCBI_Assembly:GCF_000346465.1	NW_006760385.1	+		13740822	13741795	973	-	-	-	-
Pp05_28899-3p(gma-miR1513a-5p)	18766058	PRUPE_ppa013019mg	NCBI_Assembly:GCF_000346465.1	NW_006760186.1	-		1296818	1298881	2063	pper01100:Metabolic pathways;pper00230:Purine metabolism;pper00240:Pyrimidine metabolism;pper03020:RNA polymerase	GO:0006351: transcription, DNA-templated	GO:0003677: DNA binding;GO:0003899:DNA-directed RNA polymerase activity	GO:0005665: DNA-directed RNA polymerase II, core complex
Pp05_28899-3p(gma-miR1513a-5p)	18766113	PRUPE_ppa021303mg	NCBI_Assembly:GCF_000346465.1	NW_006760186.1	-		1406240	1406719	479	-	-	-	-

Pp05_28899-3p(gma-miR1513a-5p)	18766147	PRUPE_ppa008402mg	NCBI_Assembly:GCF_000346465.1	NW_006760191.1	-	9032	10576	1544	pper01100:Metabolic pathways;pper01110:Biosynthesis of secondary metabolites;pper00941:Flavonoid biosynthesis;pper04712:Circadian rhythm - plant	GO:0009058:biosynthetic process	GO:0016747:transferase activity, transferring acyl groups other than amino-acyl groups	-
Pp05_28899-3p(gma-miR1513a-5p)	18766157	PRUPE_ppa011746mg	NCBI_Assembly:GCF_000346465.1	NW_006760194.1	+	7826773	7830158	3385	-	-	-	GO:0005774:vacuolar membrane
Pp05_28899-3p(gma-miR1513a-5p)	18766181	PRUPE_ppa023560mg	NCBI_Assembly:GCF_000346465.1	NW_006760194.1	-	13927251	13929179	1928	-	-	-	-
Pp05_28899-3p(gma-miR1513a-5p)	18766234	PRUPE_ppa027034mg	NCBI_Assembly:GCF_000346465.1	NW_006760194.1	+	12669650	12670138	488	pper04626:Plant-pathogen interaction;pper04075:Plant hormone signal transduction	-	-	GO:0005576:extracellular region
Pp05_28899-3p(gma-miR1513a-5p)	18766235	PRUPE_ppa006357mg	NCBI_Assembly:GCF_000346465.1	NW_006760194.1	-	16021971	16025844	3873	pper00330:Arginine and proline metabolism	-	GO:0004177:aminopeptidase activity	GO:0005829:cytosol;GO:0009507:chloroplast
Pp05_28899-3p(gma-miR1513a-5p)	18766272	PRUPE_ppa001038mg	NCBI_Assembly:GCF_000346465.1	NW_006760194.1	-	11043910	11050138	6228	-	GO:0007018:microtubule-based movement	GO:0003777:microtubule motor activity;GO:0005524:ATP binding	GO:0005871:kinesin complex;GO:0005874:microtubule

Pp05_28899-3p(gma-miR1513a-5p)	18766335	PRUPE_ppa002360mg	NCBI_Assembly:GCF_000346465.1	NW_006760194.1	-	7778189	7784740	6551	-	GO:0010039:response to iron ion;GO:0010343:singlet oxygen-mediated programmed cell death;GO:0055072:iron ion homeostasis	-	GO:0042651:thylakoid membrane
Pp05_28899-3p(gma-miR1513a-5p)	18766356	PRUPE_ppa016027mg	NCBI_Assembly:GCF_000346465.1	NW_006760194.1	+	688751	691569	2818	pper04626:Plant-pathogen interaction	-	GO:0043531:ADP binding	-
Pp05_28899-3p(gma-miR1513a-5p)	18766357	PRUPE_ppa021839mg	NCBI_Assembly:GCF_000346465.1	NW_006760194.1	+	641712	646274	4562	pper04626:Plant-pathogen interaction	-	GO:0043531:ADP binding	-
Pp05_28899-3p(gma-miR1513a-5p)	18766366	PRUPE_ppa013846mg	NCBI_Assembly:GCF_000346465.1	NW_006760194.1	-	10701099	10701876	777	pper04075:Plant hormone signal transduction	-	-	-
Pp05_28899-3p(gma-miR1513a-5p)	18766413	PRUPE_ppa012617mg	NCBI_Assembly:GCF_000346465.1	NW_006760194.1	-	15839347	15840059	712	pper04626:Plant-pathogen interaction;pper04075:Plant hormone signal transduction	-	-	GO:0005576:extracellular region

Pp05_28899-3p(gma-miR1513a-5p)	18766425	PRUPE_ppa007167mg	NCBI_Assembly:GCF_000346465.1	NW_006760194.1	+	1729246	1732214	2968	pper00350:Tyrosine metabolism;pper01100:Metabolic pathways;pper01110:Biosynthesis of secondary metabolites;pper00010:Glycolysis / Gluconeogenesis;pper00071:Fatty acid degradation;pper01220:Degradation of aromatic compounds	-	GO:0008270:zinc ion binding;GO:0016491:oxidoreductase activity	-
Pp05_28899-3p(gma-miR1513a-5p)	18766447	PRUPE_ppa025062mg	NCBI_Assembly:GCF_000346465.1	NW_006760194.1	+	20638583	20639068	485	-	-	-	-
Pp05_28899-3p(gma-miR1513a-5p)	18766525	PRUPE_ppa022791mg	NCBI_Assembly:GCF_000346465.1	NW_006760194.1	+	3756706	3757368	662	-	-	GO:0004672:protein kinase activity;GO:0005524:ATP binding	-
Pp05_28899-3p(gma-miR1513a-5p)	18766545	PRUPE_ppa004700mg	NCBI_Assembly:GCF_000346465.1	NW_006760194.1	-	18316822	18321421	4599	-	-	GO:0005215:transporter activity	GO:0005622:intracellular
Pp05_28899-3p(gma-miR1513a-5p)	18766557	PRUPE_ppa019737mg	NCBI_Assembly:GCF_000346465.1	NW_006760194.1	-	9023520	9025816	2296	-	-	GO:0003677:DNA binding	-

Pp05_28899-3p(gma-miR1513a-5p)	18766570	PRUPE_ppa023171mg	NCBI_Assembly:GCF_000346465.1	NW_006760194.1	+	20503760	20504728	968	pper01100:Metabolic pathways;pper01110:Biosynthesis of secondary metabolites;pper00908:Zeatin biosynthesis	GO:0007131:reciprocal meiotic recombination;GO:0008033:tRNA processing	GO:0009824:AMP dimethylallyltransferase activity;GO:0052622:ATP dimethylallyltransferase activity;GO:0052623:ADP dimethylallyltransferase activity	-
Pp05_28899-3p(gma-miR1513a-5p)	18766626	PRUPE_ppa017702mg	NCBI_Assembly:GCF_000346465.1	NW_006760194.1	+	21574097	21574426	329	-	-	-	-
Pp05_28899-3p(gma-miR1513a-5p)	18766648	PRUPE_ppb017510mg	NCBI_Assembly:GCF_000346465.1	NW_006760194.1	-	21467406	21467978	572	-	-	-	-
Pp05_28899-3p(gma-miR1513a-5p)	18766674	PRUPE_ppa012707mg	NCBI_Assembly:GCF_000346465.1	NW_006760194.1	-	21578853	21579470	617	-	-	GO:0004857:enzyme inhibitor activity;GO:0030599:pectinesterase activity	-
Pp05_28899-3p(gma-miR1513a-5p)	18766699	PRUPE_ppa000301mg	NCBI_Assembly:GCF_000346465.1	NW_006760194.1	-	20566094	20574867	8773	-	GO:2000762:regulation of phenylpropanoid metabolic process	-	GO:0016020:membrane;GO:0016592:mediator complex
Pp05_28899-3p(gma-miR1513a-5p)	18766738	PRUPE_ppa014150mg	NCBI_Assembly:GCF_000346465.1	NW_006760194.1	-	13364316	13364761	445	-	-	-	-
Pp05_28899-3p(gma-miR1513a-5p)	18766848	PRUPE_ppa016356mg	NCBI_Assembly:GCF_000346465.1	NW_006760194.1	-	1069540	1071787	2247	-	-	-	GO:0016021:integral component of membrane

Pp05_28899-3p(gma-miR1513a-5p)	18766897	PRUPE_ppa022093mg	NCBI_Assembly:GCF_000346465.1	NW_006760194.1	+	17051021	17051692	671	-	-	-	-
Pp05_28899-3p(gma-miR1513a-5p)	18766923	PRUPE_ppa023479mg	NCBI_Assembly:GCF_000346465.1	NW_006760194.1	+	19128488	19128802	314	-	-	-	-
Pp05_28899-3p(gma-miR1513a-5p)	18766929	PRUPE_ppa009751mg	NCBI_Assembly:GCF_000346465.1	NW_006760194.1	-	15125221	15126935	1714	-	-	GO:0008270: zinc ion binding	-
Pp05_28899-3p(gma-miR1513a-5p)	18766951	PRUPE_ppa008588mg	NCBI_Assembly:GCF_000346465.1	NW_006760194.1	-	15344747	15347583	2836	-	-	-	-
Pp05_28899-3p(gma-miR1513a-5p)	18767016	PRUPE_ppa013296mg	NCBI_Assembly:GCF_000346465.1	NW_006760194.1	+	16151193	16151637	444	-	-	-	-
Pp05_28899-3p(gma-miR1513a-5p)	18767029	PRUPE_ppb012676mg	NCBI_Assembly:GCF_000346465.1	NW_006760194.1	+	14475920	14477947	2027	-	-	-	-
Pp05_28899-3p(gma-miR1513a-5p)	18767078	PRUPE_ppa003352mg	NCBI_Assembly:GCF_000346465.1	NW_006760194.1	+	17130454	17135552	5098	-	-	GO:0015238: drug transmembrane transporter activity;GO:0015297:antiporter activity	GO:0016021: integral component of membrane
Pp05_28899-3p(gma-miR1513a-5p)	18767082	PRUPE_ppa022063mg	NCBI_Assembly:GCF_000346465.1	NW_006760194.1	+	16245243	16245809	566	-	-	-	-
Pp05_28899-3p(gma-miR1513a-5p)	18767086	PRUPE_ppb025610mg	NCBI_Assembly:GCF_000346465.1	NW_006760194.1	-	803320	803538	218	-	-	-	-

Pp05_28899-3p(gma-miR1513a-5p)	18767124	PRUPE_ppa009911mg	NCBI_Assembly:GCF_000346465.1	NW_006760194.1	-		16124715	16127302	2587	-	GO:0006621: protein retention in ER lumen	GO:0046923: ER retention sequence binding	GO:0005783: endoplasmic reticulum;GO:0016021:integral component of membrane
Pp05_28899-3p(gma-miR1513a-5p)	18767145	PRUPE_ppa021884mg	NCBI_Assembly:GCF_000346465.1	NW_006760194.1	+		5107996	5108598	602	pper01100:Metabolic pathways;pper01110:Biosynthesis of secondary metabolites;pper00330:Arginine and proline metabolism;pper00480:Glutathione metabolism	GO:0006596: polyamine biosynthetic process	GO:0003824: catalytic activity	-
Pp05_28899-3p(gma-miR1513a-5p)	18767161	PRUPE_ppa016398mg	NCBI_Assembly:GCF_000346465.1	NW_006760194.1	-		1500024	1501398	1374	-	GO:0045893: positive regulation of transcription, DNA-templated;GO:2000652:regulation of secondary cell wall biogenesis	GO:0003677: DNA binding;GO:0003682:chromatin binding;GO:0003700:sequence-specific DNA binding transcription factor activity	GO:0005634: nucleus
Pp05_28899-3p(gma-miR1513a-5p)	18767176	PRUPE_ppa006383mg	NCBI_Assembly:GCF_000346465.1	NW_006760194.1	-		16560479	16563217	2738	-	-	GO:0016491: oxidoreductase activity	-
Pp05_28899-3p(gma-miR1513a-5p)	18767232	PRUPE_ppa016509mg	NCBI_Assembly:GCF_000346465.1	NW_006760194.1	-		5717096	5717536	440	-	-	-	-

Pp05_28899-3p(gma-miR1513a-5p)	18767269	PRUPE_ppa008694mg	NCBI_Assembly:GCF_000346465.1	NW_006760194.1	-	10881177	10885691	4514	pper04141:Protein processing in endoplasmic reticulum	GO:0006511:ubiquitin-dependent protein catabolic process	-	-
Pp05_28899-3p(gma-miR1513a-5p)	18767305	PRUPE_ppa006618mg	NCBI_Assembly:GCF_000346465.1	NW_006760194.1	-	16452322	16455042	2720	-	-	-	GO:0005634:nucleus
Pp05_28899-3p(gma-miR1513a-5p)	18767378	PRUPE_ppa004886mg	NCBI_Assembly:GCF_000346465.1	NW_006760194.1	+	19095479	19098969	3490	-	-	GO:0003677:DNA binding;GO:0003682:chromatin binding	-
Pp05_28899-3p(gma-miR1513a-5p)	18767388	PRUPE_ppa007749mg	NCBI_Assembly:GCF_000346465.1	NW_006760194.1	-	14823809	14825849	2040	pper01100:Metabolic pathways;pper01110:Biosynthesis of secondary metabolites;pper00940:Phenylpropanoid biosynthesis	-	GO:0008270:zinc ion binding;GO:0016491:oxidoreductase activity	-
Pp05_28899-3p(gma-miR1513a-5p)	18767438	PRUPE_ppa025889mg	NCBI_Assembly:GCF_000346465.1	NW_006760194.1	+	19640316	19641161	845	-	-	-	-
Pp05_28899-3p(gma-miR1513a-5p)	18767474	PRUPE_ppa026071mg	NCBI_Assembly:GCF_000346465.1	NW_006760194.1	-	16557515	16559914	2399	-	-	GO:0016491:oxidoreductase activity	-

Pp05_28899-3p(gma-miR1513a-5p)	18767504	PRUPE_ppa010224mg	NCBI_Assembly:GCF_000346465.1	NW_006760194.1	-	20070542	20072756	2214	-	GO:0009408:response to heat	GO:0003700:sequence-specific DNA binding transcription factor activity;GO:0043565:sequence-specific DNA binding	GO:0005634:nucleus
Pp05_28899-3p(gma-miR1513a-5p)	18767530	PRUPE_ppa001973mg	NCBI_Assembly:GCF_000346465.1	NW_006760194.1	+	10192834	10197043	4209	pper03018:RNA degradation	GO:0006457:protein folding	GO:0005524:ATP binding	-
Pp05_28899-3p(gma-miR1513a-5p)	18767542	PRUPE_ppa002942mg	NCBI_Assembly:GCF_000346465.1	NW_006760194.1	+	2558783	2565353	6570	-	GO:0010228:vegetative to reproductive phase transition of meristem;GO:0016926:protein desumoylation;GO:0050665:hydrogen peroxide biosynthetic process	-	GO:0005829:cytosol
Pp05_28899-3p(gma-miR1513a-5p)	18767594	PRUPE_ppb021332mg	NCBI_Assembly:GCF_000346465.1	NW_006760194.1	-	12977400	12979664	2264	pper01100:Metabolic pathways;pper01110:Biosynthesis of secondary metabolites;pper00900:Terpenoid backbone biosynthesis	GO:0019288:isopentenyl diphosphate biosynthetic process, methylerythritol 4-phosphate pathway;GO:0050992:dimethylallyl diphosphate biosynthetic process	GO:0046872:metal ion binding;GO:0051745:4-hydroxy-3-methylbut-2-en-1-yl diphosphate reductase activity	-

Pp05_28899-3p(gma-miR1513a-5p)	18767662	PRUPE_ppa023184mg	NCBI_Assembly:GCF_000346465.1	NW_006760194.1	+	3284710	3286350	1640	-	-	-	-
Pp05_28899-3p(gma-miR1513a-5p)	18767681	PRUPE_ppa008903mg	NCBI_Assembly:GCF_000346465.1	NW_006760194.1	+	8002291	8005779	3488	pper00910:Nitrogen metabolism	GO:0015976:carbon utilization;GO:0019243:methylglyoxal catabolic process to D-lactate	GO:0004089:carbonate dehydratase activity;GO:0008270:zinc ion binding	GO:0009507:chloroplast
Pp05_28899-3p(gma-miR1513a-5p)	18767685	PRUPE_ppa004071mg	NCBI_Assembly:GCF_000346465.1	NW_006760194.1	+	21345419	21348426	3007	-	GO:0006826:iron ion transport;GO:0006829:zinc II ion transport;GO:0015693:magnesium ion transport;GO:0055085:transmembrane transport	-	GO:0016021:integral component of membrane
Pp05_28899-3p(gma-miR1513a-5p)	18767689	PRUPE_ppa004010mg	NCBI_Assembly:GCF_000346465.1	NW_006760194.1	+	4852558	4858364	5806	pper03015:mRNA surveillance pathway	GO:0031123:RNA 3'-end processing;GO:0045892:negative regulation of transcription, DNA-templated;GO:0048589:developmental growth	GO:0000166:nucleotide binding;GO:0003729:mRNA binding	-
Pp05_28899-3p(gma-miR1513a-5p)	18767699	PRUPE_ppa027193mg	NCBI_Assembly:GCF_000346465.1	NW_006760194.1	+	1649253	1650560	1307	-	-	-	-

Pp05_28899-3p(gma-miR1513a-5p)	18767707	PRUPE_ppa002730mg	NCBI_Assembly:GCF_000346465.1	NW_006760194.1	-	14155833	14164461	8628	-	-	GO:0008270:zinc ion binding;GO:0016491:oxidoreductase activity	-
Pp05_28899-3p(gma-miR1513a-5p)	18768799	PRUPE_ppa002976mg	NCBI_Assembly:GCF_000346465.1	NW_006760194.1	+	18590553	18595909	5356	-	GO:0016125:sterol metabolic process;GO:0030259:lipid glycosylation;GO:0048316:seed development	GO:0051507:beta-sitosterol UDP-glucosyltransferase activity	GO:0005886:plasma membrane
Pp05_28899-3p(gma-miR1513a-5p)	18768817	PRUPE_ppa018814mg	NCBI_Assembly:GCF_000346465.1	NW_006760194.1	-	867799	870733	2934	pper04626:Plant-pathogen interaction	-	GO:0043531:ADP binding	-
Pp05_28899-3p(gma-miR1513a-5p)	18768836	PRUPE_ppa018634mg	NCBI_Assembly:GCF_000346465.1	NW_006760194.1	+	4057829	4059891	2062	-	-	GO:0003676:nucleic acid binding	-
Pp05_28899-3p(gma-miR1513a-5p)	18768853	PRUPE_ppa008471mg	NCBI_Assembly:GCF_000346465.1	NW_006760194.1	-	10872618	10876264	3646	-	-	-	-
Pp05_28899-3p(gma-miR1513a-5p)	18768859	PRUPE_ppa001719mg	NCBI_Assembly:GCF_000346465.1	NW_006760194.1	-	17268068	17273184	5116	-	-	-	-
Pp05_28899-3p(gma-miR1513a-5p)	18768932	PRUPE_ppa021743mg	NCBI_Assembly:GCF_000346465.1	NW_006760194.1	-	15832852	15833251	399	-	-	-	-
Pp05_28899-3p(gma-miR1513a-5p)	18768997	PRUPE_ppa019154mg	NCBI_Assembly:GCF_000346465.1	NW_006760194.1	-	13329336	13329629	293	-	-	-	-
Pp05_28899-3p(gma-miR1513a-5p)	18768998	PRUPE_ppa022091mg	NCBI_Assembly:GCF_000346465.1	NW_006760194.1	+	429842	434039	4197	-	GO:0007165:signal transduction	GO:0043531:ADP binding	-

Pp05_28899-3p(gma-miR1513a-5p)	18769141	PRUPE_ppa007424mg	NCBI_Assembly:GCF_000346465.1	NW_006760201.1	+	11921952	11923760	1808	pper04120:Ubiquitin mediated proteolysis	-	-	-
Pp05_28899-3p(gma-miR1513a-5p)	18769163	PRUPE_ppa016114mg	NCBI_Assembly:GCF_000346465.1	NW_006760201.1	-	18085897	18088889	2992	-	-	-	GO:0005634: nucleus
Pp05_28899-3p(gma-miR1513a-5p)	18769178	PRUPE_ppa019316mg	NCBI_Assembly:GCF_000346465.1	NW_006760201.1	+	19697390	19702210	4820	-	-	-	-
Pp05_28899-3p(gma-miR1513a-5p)	18769227	PRUPE_ppa023057mg	NCBI_Assembly:GCF_000346465.1	NW_006760201.1	+	21127941	21128722	781	-	-	-	-
Pp05_28899-3p(gma-miR1513a-5p)	18769235	PRUPE_ppa017631mg	NCBI_Assembly:GCF_000346465.1	NW_006760201.1	-	18391304	18394005	2701	-	-	GO:0022891: substrate-specific transmembrane transporter activity	GO:0016021: integral component of membrane
Pp05_28899-3p(gma-miR1513a-5p)	18769284	PRUPE_ppa009644mg	NCBI_Assembly:GCF_000346465.1	NW_006760201.1	+	3687862	3692663	4801	-	-	GO:0000166: nucleotide binding;GO:0003676:nucleic acid binding	-
Pp05_28899-3p(gma-miR1513a-5p)	18769293	PRUPE_ppa010251mg	NCBI_Assembly:GCF_000346465.1	NW_006760201.1	-	19786201	19788526	2325	pper01100:Metabolic pathways;pper00750:Vitamin B6 metabolism	GO:0051262: protein tetramerization	GO:0016791: phosphatase activity	-
Pp05_28899-3p(gma-miR1513a-5p)	18769304	PRUPE_ppa015600mg	NCBI_Assembly:GCF_000346465.1	NW_006760201.1	-	12668874	12670763	1889	pper04120:Ubiquitin mediated proteolysis	-	-	-
Pp05_28899-3p(gma-miR1513a-5p)	18769306	PRUPE_ppa022420mg	NCBI_Assembly:GCF_000346465.1	NW_006760201.1	-	17889600	17891484	1884	-	-	GO:0008519: ammonium transmembrane transporter activity	GO:0016021: integral component of membrane

Pp05_28899-3p(gma-miR1513a-5p)	18769317	PRUPE_ppa019099mg	NCBI_Assembly:GCF_000346465.1	NW_006760201.1	-	21221167	21221958	791	pper01100:Metabolic pathways;pper00600:Sphingolipid metabolism	GO:0006633:fatty acid biosynthetic process	GO:0005506:iron ion binding;GO:0016491:oxidoreductase activity	GO:0016021:integral component of membrane
Pp05_28899-3p(gma-miR1513a-5p)	18769325	PRUPE_ppa024062mg	NCBI_Assembly:GCF_000346465.1	NW_006760201.1	-	21327709	21329366	1657	-	GO:0006098:pentose-phosphate shunt;GO:0006364:rRNA processing;GO:0006636:unsaturated fatty acid biosynthetic process;GO:0009409:response to cold;GO:0009697:salicylic acid biosynthetic process;GO:0009814:defense response, incompatible interaction;GO:0010027:thylakoid membrane organization;GO:0015995:chlorophyll biosynthetic process;GO:0016117:carotenoid biosynthetic process;GO:0019288:isopentenyl diphosphate biosynthetic [illegible]	-	GO:0009535:chloroplast thylakoid membrane;GO:0009570:chloroplast stroma;GO:0009941:chloroplast envelope;GO:0031977:thylakoid lumen

Pp05_28899-3p(gma-miR1513a-5p)	18769355	PRUPE_ppa017675mg	NCBI_Assembly:GCF_000346465.1	NW_006760201.1	-		7743203	7746773	3570	-	GO:0009058: biosynthetic process	GO:0016844: strictosidine synthase activity	GO:0005783: endoplasmic reticulum;GO:0005886:plasma membrane;GO:0009505:plant-type cell wall;GO:0009506:plasmodesma
Pp05_28899-3p(gma-miR1513a-5p)	18769358	PRUPE_ppa018638mg	NCBI_Assembly:GCF_000346465.1	NW_006760201.1	+		21301708	21303361	1653	-	-	-	-
Pp05_28899-3p(gma-miR1513a-5p)	18769368	PRUPE_ppa009402mg	NCBI_Assembly:GCF_000346465.1	NW_006760201.1	-		4139985	4145597	5612	-	GO:0045454: cell redox homeostasis	-	GO:0005623: cell
Pp05_28899-3p(gma-miR1513a-5p)	18769406	PRUPE_ppa008180mg	NCBI_Assembly:GCF_000346465.1	NW_006760201.1	-		22507992	22512897	4905	-	-	GO:0008270: zinc ion binding;GO:0016491:oxidoreductase activity	-
Pp05_28899-3p(gma-miR1513a-5p)	18769430	PRUPE_ppa006645mg	NCBI_Assembly:GCF_000346465.1	NW_006760201.1	-		19813769	19816700	2931	-	GO:0006479: protein methylation	GO:0008168: methyltransferase activity	-
Pp05_28899-3p(gma-miR1513a-5p)	18769433	PRUPE_ppa022013mg	NCBI_Assembly:GCF_000346465.1	NW_006760201.1	-		17612494	17614557	2063	-	-	-	-
Pp05_28899-3p(gma-miR1513a-5p)	18769455	PRUPE_ppa007444mg	NCBI_Assembly:GCF_000346465.1	NW_006760201.1	+		11232972	11235501	2529	-	GO:0009827: plant-type cell wall modification; GO:0009860: pollen tube growth	GO:0004713: protein tyrosine kinase activity;GO:0005524:ATP binding	-
Pp05_28899-3p(gma-miR1513a-5p)	18769567	PRUPE_ppa011781mg	NCBI_Assembly:GCF_000346465.1	NW_006760201.1	-		17335519	17337899	2380	-	-	-	-

Pp05_28899-3p(gma-miR1513a-5p)	18769584	PRUPE_ppa025651mg	NCBI_Assembly:GCF_000346465.1	NW_006760201.1	+	22246820	22247712	892	-	-	GO:0009055: electron carrier activity	-
Pp05_28899-3p(gma-miR1513a-5p)	18769614	PRUPE_ppa026335mg	NCBI_Assembly:GCF_000346465.1	NW_006760201.1	-	14195265	14196815	1550	-	-	-	-
Pp05_28899-3p(gma-miR1513a-5p)	18769649	PRUPE_ppa000164mg	NCBI_Assembly:GCF_000346465.1	NW_006760201.1	-	1864731	1872117	7386	-	GO:0006139: nucleobase-containing compound metabolic process;GO:0006357:regulation of transcription from RNA polymerase II promoter;GO:0032784:regulation of DNA-templated transcription, elongation	GO:0003676: nucleic acid binding	-
Pp05_28899-3p(gma-miR1513a-5p)	18769650	PRUPE_ppa012212mg	NCBI_Assembly:GCF_000346465.1	NW_006760201.1	-	756617	758359	1742	-	-	-	-
Pp05_28899-3p(gma-miR1513a-5p)	18769665	PRUPE_ppa021827mg	NCBI_Assembly:GCF_000346465.1	NW_006760201.1	+	19548269	19548718	449	-	-	-	-
Pp05_28899-3p(gma-miR1513a-5p)	18769715	PRUPE_ppa022952mg	NCBI_Assembly:GCF_000346465.1	NW_006760201.1	+	15610512	15613445	2933	-	-	-	-
Pp05_28899-3p(gma-miR1513a-5p)	18769717	PRUPE_ppa010561mg	NCBI_Assembly:GCF_000346465.1	NW_006760201.1	+	12914379	12915766	1387	-	-	-	GO:0005886: plasma membrane

Pp05_28899-3p(gma-miR1513a-5p)	18769742	PRUPE_ppa009201mg	NCBI_Assembly:GCF_000346465.1	NW_006760201.1	+	1273104	1279839	6735	pper03050:Proteasome	GO:0006094:gluconeogenesis;GO:0006096:glycolytic process;GO:0006833:water transport;GO:0006972:hyperosmotic response;GO:0007030:Golgi organization;GO:0009266:response to temperature stimulus;GO:0009651:response to salt stress;GO:0009853:photorespiration;GO:0043161:proteasome-mediated ubiquitin-dependent protein catabolic process;GO:0051788:response to misfolded protein;GO:0080129:proteasome core complex	GO:0004298:threonine-type endopeptidase activity	GO:0005634:nucleus;GO:0005774:vacuolar membrane;GO:0005829:cytosol;GO:0019773:proteasome core complex, alpha-subunit complex;GO:0048046:apoplast
Pp05_28899-3p(gma-miR1513a-5p)	18769754	PRUPE_ppa005968mg	NCBI_Assembly:GCF_000346465.1	NW_006760201.1	-	14415700	14419084	3384	pper01100:Metabolic pathways;pper00053:Ascorbate and aldarate metabolism	-	GO:0016491:oxidoreductase activity;GO:0050660:flavin adenine dinucleotide binding	-

Pp05_28899-3p(gma-miR1513a-5p)	18769755	PRUPE_ppa018709mg	NCBI_Assembly:GCF_000346465.1	NW_006760201.1	-	12520552	12523490	2938	-	-	-	-
Pp05_28899-3p(gma-miR1513a-5p)	18769806	PRUPE_ppa014801mg	NCBI_Assembly:GCF_000346465.1	NW_006760201.1	-	15379225	15381835	2610	-	-	GO:0003676: nucleic acid binding	-
Pp05_28899-3p(gma-miR1513a-5p)	18769810	PRUPE_ppa022574mg	NCBI_Assembly:GCF_000346465.1	NW_006760201.1	+	11273264	11274451	1187	-	-	-	-
Pp05_28899-3p(gma-miR1513a-5p)	18769829	PRUPE_ppa026881mg	NCBI_Assembly:GCF_000346465.1	NW_006760201.1	+	1826812	1828764	1952	-	-	-	-
Pp05_28899-3p(gma-miR1513a-5p)	18769841	PRUPE_ppa014409mg	NCBI_Assembly:GCF_000346465.1	NW_006760201.1	-	19001745	19002367	622	-	-	-	-
Pp05_28899-3p(gma-miR1513a-5p)	18769866	PRUPE_ppb022654mg	NCBI_Assembly:GCF_000346465.1	NW_006760201.1	+	6610590	6611117	527	-	GO:0005975: carbohydrate metabolic process	GO:0004650: polygalacturonase activity	-
Pp05_28899-3p(gma-miR1513a-5p)	18769867	PRUPE_ppa005749mg	NCBI_Assembly:GCF_000346465.1	NW_006760201.1	+	14311764	14314990	3226	-	-	GO:0003993: acid phosphatase activity;GO:0046872:metal ion binding	-
Pp05_28899-3p(gma-miR1513a-5p)	18769872	PRUPE_ppa013132mg	NCBI_Assembly:GCF_000346465.1	NW_006760201.1	-	7318719	7319747	1028	-	GO:0006351: transcription, DNA-templated;GO:0006355:regulation of transcription, DNA-templated	GO:0003677: DNA binding	GO:0005634: nucleus
Pp05_28899-3p(gma-miR1513a-5p)	18769917	PRUPE_ppa021775mg	NCBI_Assembly:GCF_000346465.1	NW_006760201.1	-	10264357	10264572	215	-	-	-	-

Pp05_28899-3p(gma-miR1513a-5p)	18769945	PRUPE_ppa017145mg	NCBI_Assembly:GCF_000346465.1	NW_006760201.1	+		13577250	13578755	1505	-	-	-	-
Pp05_28899-3p(gma-miR1513a-5p)	18770024	PRUPE_ppa022474mg	NCBI_Assembly:GCF_000346465.1	NW_006760201.1	+		15702204	15704862	2658	-	-	-	-
Pp05_28899-3p(gma-miR1513a-5p)	18770041	PRUPE_ppa014848mg	NCBI_Assembly:GCF_000346465.1	NW_006760201.1	-		7416260	7422872	6612	-	-	-	-
Pp05_28899-3p(gma-miR1513a-5p)	18770049	PRUPE_ppb017252mg	NCBI_Assembly:GCF_000346465.1	NW_006760201.1	+		4814000	4815093	1093	-	-	-	-
Pp05_28899-3p(gma-miR1513a-5p)	18770100	PRUPE_ppa012913mg	NCBI_Assembly:GCF_000346465.1	NW_006760201.1	+		11740748	11741782	1034	-	-	-	-
Pp05_28899-3p(gma-miR1513a-5p)	18770165	PRUPE_ppa023789mg	NCBI_Assembly:GCF_000346465.1	NW_006760201.1	-		19201086	19202060	974	-	-	GO:0003677:DNA binding;GO:0003682:chromatin binding	-
Pp05_28899-3p(gma-miR1513a-5p)	18770199	PRUPE_ppa008611mg	NCBI_Assembly:GCF_000346465.1	NW_006760201.1	+		9473870	9474884	1014	-	-	GO:0008270:zinc ion binding;GO:0016491:oxidoreductase activity	-
Pp05_28899-3p(gma-miR1513a-5p)	18770277	PRUPE_ppa021118mg	NCBI_Assembly:GCF_000346465.1	NW_006760201.1	+		15098336	15100024	1688	-	GO:0009664:plant-type cell wall organization	-	GO:0005576:extracellular region;GO:0005618:cell wall;GO:0016020:membrane
Pp05_28899-3p(gma-miR1513a-5p)	18770300	PRUPE_ppa010571mg	NCBI_Assembly:GCF_000346465.1	NW_006760201.1	+		8563813	8564678	865	-	-	-	-

Pp05_28899-3p(gma-miR1513a-5p)	18770334	PRUPE_ppa010050mg	NCBI_Assembly:GCF_000346465.1	NW_006760201.1	-	13372733	13373581	848	-	-	-	-	
Pp05_28899-3p(gma-miR1513a-5p)	18770337	PRUPE_ppa005964mg	NCBI_Assembly:GCF_000346465.1	NW_006760201.1	-	17973735	17976523	2788	-	-	-	-	
Pp05_28899-3p(gma-miR1513a-5p)	18770359	PRUPE_ppa000006mg	NCBI_Assembly:GCF_000346465.1	NW_006760201.1	+	17496972	17519724	22752	-	-	GO:0016773:phosphotransferase activity, alcohol group as acceptor	-	
Pp05_28899-3p(gma-miR1513a-5p)	18770383	PRUPE_ppa024884mg	NCBI_Assembly:GCF_000346465.1	NW_006760201.1	+	6006135	6006649	514	-	-	-	-	
Pp05_28899-3p(gma-miR1513a-5p)	18770392	PRUPE_ppa004251mg	NCBI_Assembly:GCF_000346465.1	NW_006760201.1	+	14003149	14005027	1878	pper03040:Spliceosome;pper04141:Protein processing in endoplasmic reticulum;pper04144:Endocytosis	-	GO:0005524:ATP binding	-	
Pp05_28899-3p(gma-miR1513a-5p)	18770467	PRUPE_ppa008425mg	NCBI_Assembly:GCF_000346465.1	NW_006760201.1	-	19758734	19760065	1331	-	-	GO:0003677:DNA binding;GO:0003682:chromatin binding;GO:0003700:sequence-specific DNA binding transcription factor activity;GO:0008270:zinc ion binding	-	

Pp05_28899-3p(gma-miR1513a-5p)	18770478	PRUPE_ppa024258mg	NCBI_Assembly:GCF_000346465.1	NW_006760201.1	+		11694119	11698419	4300	-	GO:0007165:signal transduction	GO:0043531:ADP binding	-
Pp05_28899-3p(gma-miR1513a-5p)	18770486	PRUPE_ppa022233mg	NCBI_Assembly:GCF_000346465.1	NW_006760201.1	-		12028657	12031458	2801	-	-	-	-
Pp05_28899-3p(gma-miR1513a-5p)	18770520	PRUPE_ppa001730mg	NCBI_Assembly:GCF_000346465.1	NW_006760201.1	-		9869886	9875854	5968	pper00052:Galactose metabolism	GO:0034484:raffinose catabolic process	GO:0052692:raffinose alpha-galactosidase activity	GO:0009506:plasmodesma
Pp05_28899-3p(gma-miR1513a-5p)	18770544	PRUPE_ppa018249mg	NCBI_Assembly:GCF_000346465.1	NW_006760201.1	+		6019076	6019782	706	pper03010:Ribosome	GO:0006412:translation	GO:0003735:structural constituent of ribosome	GO:0005840:ribosome
Pp05_28899-3p(gma-miR1513a-5p)	18770557	PRUPE_ppa022985mg	NCBI_Assembly:GCF_000346465.1	NW_006760201.1	+		22583029	22584006	977	-	-	-	-
Pp05_28899-3p(gma-miR1513a-5p)	18770559	PRUPE_ppa023701mg	NCBI_Assembly:GCF_000346465.1	NW_006760201.1	+		5651188	5651738	550	-	-	-	-
Pp05_28899-3p(gma-miR1513a-5p)	18770615	PRUPE_ppa000677mg	NCBI_Assembly:GCF_000346465.1	NW_006760201.1	-		12171016	12180434	9418	-	GO:0007018:microtubule-based movement	GO:0003777:microtubule motor activity;GO:0005524:ATP binding	GO:0005871:kinesin complex;GO:0005874:microtubule

Pp05_28899-3p(gma-miR1513a-5p)	18770729	PRUPE_ppa006328mg	NCBI_Assembly:GCF_000346465.1	NW_006760201.1	+	20832706	20837667	4961	pper01100:Metabolic pathways;pper00061:Fatty acid biosynthesis;pper01212:Fatty acid metabolism	-	GO:0000036:ACP phosphopantetheine attachment site binding involved in fatty acid biosynthetic process;GO:0016297:acyl-[acyl-carrier-protein] hydrolase activity	-
Pp05_28899-3p(gma-miR1513a-5p)	18770749	PRUPE_ppa009633mg	NCBI_Assembly:GCF_000346465.1	NW_006760201.1	-	21236838	21239393	2555	pper00860:Porphyrin and chlorophyll metabolism	GO:0006788:heme oxidation	GO:0004392:heme oxygenase (decyclizing) activity	-
Pp05_28899-3p(gma-miR1513a-5p)	18770775	PRUPE_ppa000206mg	NCBI_Assembly:GCF_000346465.1	NW_006760201.1	-	21814608	21820314	5706	-	GO:0009644:response to high light intensity;GO:0042542:response to hydrogen peroxide	GO:0004712:protein serine/threonine/tyrosine kinase activity;GO:0005524:ATP binding	-
Pp05_28899-3p(gma-miR1513a-5p)	18770782	PRUPE_ppa020417mg	NCBI_Assembly:GCF_000346465.1	NW_006760201.1	-	11935491	11936111	620	-	GO:0006351:transcription, DNA-templated	GO:0003677:DNA binding;GO:0003700:sequence-specific DNA binding transcription factor activity	GO:0005634:nucleus
Pp05_28899-3p(gma-miR1513a-5p)	18770788	PRUPE_ppa024680mg	NCBI_Assembly:GCF_000346465.1	NW_006760201.1	+	3281893	3283162	1269	-	-	-	-

Pp05_28899-3p(gma-miR1513a-5p)	18770850	PRUPE_ppa012455m1g	NCBI_Assembly:GCF_000346465.1	NW_006760201.1	-	2556244	2557443	1199	-	-	-	-
Pp05_28899-3p(gma-miR1513a-5p)	18770862	PRUPE_ppa000497m1g	NCBI_Assembly:GCF_000346465.1	NW_006760201.1	-	22686904	22699176	12272	-	-	GO:0003824:catalytic activity;GO:0046872:metal ion binding	-
Pp05_28899-3p(gma-miR1513a-5p)	18770900	PRUPE_ppa022167mg	NCBI_Assembly:GCF_000346465.1	NW_006760201.1	-	20083820	20087220	3400	-	-	GO:0004674:protein serine/threonine kinase activity;GO:0005524:ATP binding	GO:0016021:integral component of membrane
Pp05_28899-3p(gma-miR1513a-5p)	18770929	PRUPE_ppa011158mg	NCBI_Assembly:GCF_000346465.1	NW_006760201.1	+	19166492	19168401	1909	-	-	GO:0001104:RNA polymerase II transcription cofactor activity	GO:0016592:mediator complex
Pp05_28899-3p(gma-miR1513a-5p)	18770939	PRUPE_ppa002539mg	NCBI_Assembly:GCF_000346465.1	NW_006760201.1	-	16430294	16432386	2092	-	GO:0045087:innate immune response	GO:0004672:protein kinase activity;GO:0005524:ATP binding	-
Pp05_28899-3p(gma-miR1513a-5p)	18770968	PRUPE_ppa023459mg	NCBI_Assembly:GCF_000346465.1	NW_006760201.1	+	11735488	11739609	4121	-	GO:0007165:signal transduction	GO:0043531:ADP binding	-
Pp05_28899-3p(gma-miR1513a-5p)	18771011	PRUPE_ppa004640mg	NCBI_Assembly:GCF_000346465.1	NW_006760201.1	+	9718694	9725701	7007	-	-	-	-
Pp05_28899-3p(gma-miR1513a-5p)	18771056	PRUPE_ppa004791mg	NCBI_Assembly:GCF_000346465.1	NW_006760201.1	-	5226423	5234311	7888	-	-	-	-

Pp05_28899-3p(gma-miR1513a-5p)	18771059	PRUPE_ppa022019mg	NCBI_Assembly:GCF_000346465.1	NW_006760201.1	+	18294528	18297811	3283	-	-	GO:0004970: ionotropic glutamate receptor activity	GO:0016021: integral component of membrane
Pp05_28899-3p(gma-miR1513a-5p)	18771088	PRUPE_ppa022023mg	NCBI_Assembly:GCF_000346465.1	NW_006760201.1	+	11683983	11687924	3941	-	GO:0007165: signal transduction	GO:0043531: ADP binding	-
Pp05_28899-3p(gma-miR1513a-5p)	18771113	PRUPE_ppa003750mg	NCBI_Assembly:GCF_000346465.1	NW_006760201.1	+	1981106	1982761	1655	-	-	GO:0016773: phosphotransferase activity, alcohol group as acceptor	-
Pp05_28899-3p(gma-miR1513a-5p)	18771152	PRUPE_ppa011734mg	NCBI_Assembly:GCF_000346465.1	NW_006760201.1	-	22490910	22492306	1396	-	GO:0006412: translation	GO:0003723: RNA binding;GO:0003735:structural constituent of ribosome;GO:0016740:transferase activity	GO:0015934: large ribosomal subunit
Pp05_28899-3p(gma-miR1513a-5p)	18771165	PRUPE_ppa011317mg	NCBI_Assembly:GCF_000346465.1	NW_006760201.1	-	1108894	1111843	2949	-	-	-	GO:0005768: endosome;GO:0005802:trans-Golgi network
Pp05_28899-3p(gma-miR1513a-5p)	18771192	PRUPE_ppa008024mg	NCBI_Assembly:GCF_000346465.1	NW_006760201.1	+	16607379	16611066	3687	-	GO:0006351: transcription, DNA-templated;GO:0010310:regulation of hydrogen peroxide metabolic process;GO:0090342:regulation of cell aging	GO:0003700: sequence-specific DNA binding transcription factor activity;GO:0043565:sequence-specific DNA binding	GO:0005634: nucleus;GO:0005737:cytoplasm

Pp05_28899-3p(gma-miR1513a-5p)	18771262	PRUPE_ppa023563mg	NCBI_Assembly:GCF_000346465.1	NW_006760201.1	-	17576928	17578736	1808	-	-	-	-
Pp05_28899-3p(gma-miR1513a-5p)	18771268	PRUPE_ppa010130mg	NCBI_Assembly:GCF_000346465.1	NW_006760201.1	-	20261165	20262021	856	-	GO:0006351: transcription, DNA-templated	GO:0003677: DNA binding;GO:0003700:sequence-specific DNA binding transcription factor activity	GO:0005634: nucleus
Pp05_28899-3p(gma-miR1513a-5p)	18771278	PRUPE_ppa001232mg	NCBI_Assembly:GCF_000346465.1	NW_006760201.1	-	6345096	6350116	5020	-	GO:0005975: carbohydrate metabolic process	GO:0004553: hydrolase activity, hydrolyzing O-glycosyl compounds; GO:0030246: carbohydrate binding	-
Pp05_28899-3p(gma-miR1513a-5p)	18771302	PRUPE_ppa004692mg	NCBI_Assembly:GCF_000346465.1	NW_006760201.1	+	16411752	16415585	3833	-	-	GO:0008270: zinc ion binding	-

Pp05_28899-3p(gma-miR1513a-5p)	18771317	PRUPE_ppa005923mg	NCBI_Assembly:GCF_000346465.1	NW_006760201.1	-	8706180	8709661	3481	pper03013:RNA transport	GO:0000398:mRNA splicing, via spliceosome;GO:0001731:formation of translation preinitiation complex;GO:0006352:DNA-templated transcription, initiation;GO:0006406:mRNA export from nucleus;GO:0006446:regulation of translational initiation;GO:0006511:ubiquitin-dependent protein catabolic process;GO:0006606:protein import into nucleus;GO:0009640:photomorphogenesis;GO:0009793:embryo development ending in seed dormancy;G	GO:0003743:translation initiation factor activity	GO:0005829:cytosol;GO:0005852:eukaryotic translation initiation factor 3 complex;GO:0005886:plasma membrane;GO:0008180:COP9 signalosome;GO:0016282:eukaryotic 43S preinitiation complex;GO:0033290:eukaryotic 48S preinitiation complex
Pp05_28899-3p(gma-miR1513a-5p)	18771323	PRUPE_ppa003288mg	NCBI_Assembly:GCF_000346465.1	NW_006760201.1	+	10799950	10802729	2779	-	-	GO:0008270:zinc ion binding;GO:0047134:protein-disulfide reductase activity	-

Pp05_28899-3p(gma-miR1513a-5p)	18771338	PRUPE_ppa017495mg	NCBI_Assembly:GCF_000346465.1	NW_006760201.1	+	13772424	13773197	773	-	-	-	-
Pp05_28899-3p(gma-miR1513a-5p)	18771343	PRUPE_ppa004997mg	NCBI_Assembly:GCF_000346465.1	NW_006760201.1	+	16727032	16730197	3165	-	-	-	GO:0016021: integral component of membrane
Pp05_28899-3p(gma-miR1513a-5p)	18771395	PRUPE_ppa005513mg	NCBI_Assembly:GCF_000346465.1	NW_006760201.1	-	21141917	21145661	3744	-	GO:0000478: endonucleolytic cleavage involved in rRNA processing;GO:0001510:RNA methylation; GO:0006406: mRNA export from nucleus;GO:0006606:protein import into nucleus;GO:0006626:protein targeting to mitochondrion;GO:0009640:photomorphogenesis;GO:0009909:regulation of flower development;GO:0010388:cullin deneddylation	-	GO:0005730: nucleolus

Pp05_28899-3p(gma-miR1513a-5p)	18771401	PRUPE_ppa018352mg	NCBI_Assembly:GCF_000346465.1	NW_006760201.1	-	8321418	8322017	599	-	-	-	-
Pp05_28899-3p(gma-miR1513a-5p)	18771409	PRUPE_ppa020526mg	NCBI_Assembly:GCF_000346465.1	NW_006760201.1	+	10943095	10943607	512	-	-	-	-
Pp05_28899-3p(gma-miR1513a-5p)	18771501	PRUPE_ppa018170mg	NCBI_Assembly:GCF_000346465.1	NW_006760201.1	+	17827657	17830676	3019	-	-	-	-
Pp05_28899-3p(gma-miR1513a-5p)	18771509	PRUPE_ppa024781mg	NCBI_Assembly:GCF_000346465.1	NW_006760201.1	+	15684583	15686617	2034	-	GO:0016042:lipid catabolic process	GO:0016787:hydrolase activity	-
Pp05_28899-3p(gma-miR1513a-5p)	18771510	PRUPE_ppa013737mg	NCBI_Assembly:GCF_000346465.1	NW_006760201.1	-	13411681	13414819	3138	-	-	-	-
Pp05_28899-3p(gma-miR1513a-5p)	18771512	PRUPE_ppa018858mg	NCBI_Assembly:GCF_000346465.1	NW_006760201.1	+	2186231	2187817	1586	-	-	-	-
Pp05_28899-3p(gma-miR1513a-5p)	18771514	PRUPE_ppa024351mg	NCBI_Assembly:GCF_000346465.1	NW_006760201.1	-	13981761	13983378	1617	-	-	-	-
Pp05_28899-3p(gma-miR1513a-5p)	18771548	PRUPE_ppa002357mg	NCBI_Assembly:GCF_000346465.1	NW_006760201.1	+	14798173	14800786	2613	-	-	GO:0004672:protein kinase activity;GO:0005524:ATP binding	GO:0005886:plasma membrane;GO:0016021:integral component of membrane

Pp05_28899-3p(gma-miR1513a-5p)	18771620	PRUPE_ppa011731mg	NCBI_Assembly:GCF_000346465.1	NW_006760201.1	+	13609366	13611175	1809	-	GO:0000023:maltose metabolic process;GO:0006098:pentose-phosphate shunt;GO:0019252:starch biosynthetic process;GO:0043085:positive regulation of catalytic activity	-	GO:0009535:chloroplast thylakoid membrane;GO:0009570:chloroplast stroma
Pp05_28899-3p(gma-miR1513a-5p)	18771648	PRUPE_ppa004995mg	NCBI_Assembly:GCF_000346465.1	NW_006760201.1	-	18945716	18948772	3056	-	-	-	GO:0005768:endosome;GO:0005802:trans-Golgi network
Pp05_28899-3p(gma-miR1513a-5p)	18771665	PRUPE_ppa010958mg	NCBI_Assembly:GCF_000346465.1	NW_006760201.1	-	21509128	21510179	1051	-	-	-	GO:0009506:plasmodesma;GO:0009535:chloroplast thylakoid membrane;GO:0048046:apoplast
Pp05_28899-3p(gma-miR1513a-5p)	18771704	PRUPE_ppa027117mg	NCBI_Assembly:GCF_000346465.1	NW_006760201.1	+	14349268	14349594	326	-	-	-	-
Pp05_28899-3p(gma-miR1513a-5p)	18771721	PRUPE_ppa026457mg	NCBI_Assembly:GCF_000346465.1	NW_006760201.1	-	10016041	10016433	392	-	-	-	-
Pp05_28899-3p(gma-miR1513a-5p)	18771794	PRUPE_ppa021572mg	NCBI_Assembly:GCF_000346465.1	NW_006760201.1	+	12691696	12694521	2825	-	-	GO:0003676:nucleic acid binding	-

Pp05_28899-3p(gma-miR1513a-5p)	18771836	PRUPE_ppa022991mg	NCBI_Assembly:GCF_000346465.1	NW_006760201.1	+	19572277	19572726	449	-	-	-	-
Pp05_28899-3p(gma-miR1513a-5p)	18771857	PRUPE_ppa018296mg	NCBI_Assembly:GCF_000346465.1	NW_006760201.1	+	13060473	13061405	932	-	-	GO:0003677:DNA binding;GO:0003700:sequence-specific DNA binding transcription factor activity	-
Pp05_28899-3p(gma-miR1513a-5p)	18771881	PRUPE_ppa009379mg	NCBI_Assembly:GCF_000346465.1	NW_006760201.1	+	11226211	11230765	4554	pper04120:Ubiquitin mediated proteolysis	GO:0006511:ubiquitin-dependent protein catabolic process;GO:0007275:multicellular organismal development	GO:0004842:ubiquitin-protein transferase activity;GO:0008270:zinc ion binding;GO:0016874:ligase activity	GO:0005634:nucleus
Pp05_28899-3p(gma-miR1513a-5p)	18771885	PRUPE_ppa024770mg	NCBI_Assembly:GCF_000346465.1	NW_006760201.1	+	1746961	1747406	445	pper04120:Ubiquitin mediated proteolysis;pper04141:Protein processing in endoplasmic reticulum	GO:0006511:ubiquitin-dependent protein catabolic process	-	-
Pp05_28899-3p(gma-miR1513a-5p)	18771921	PRUPE_ppb017894mg	NCBI_Assembly:GCF_000346465.1	NW_006760207.1	-	4867	6675	1808	-	-	GO:0008168:methyltransferase activity	-
Pp05_28899-3p(gma-miR1513a-5p)	18771949	PRUPE_ppa010707mg	NCBI_Assembly:GCF_000346465.1	NW_006760208.1	+	6356803	6358379	1576	-	-	GO:0008234:cysteine-type peptidase activity	-

Pp05_28899-3p(gma-miR1513a-5p)	18771979	PRUPE_ppa015455mg	NCBI_Assembly:GCF_000346465.1	NW_006760208.1	-	25733182	25736715	3533	-	GO:0006270:DNA replication initiation;GO:0006275:regulation of DNA replication;GO:0006306:DNA methylation;GO:0007067:mitotic nuclear division;GO:0008283:cell proliferation;GO:0009909:regulation of flower development;GO:0010389:regulation of G2/M transition of mitotic cell cycle;GO:0031047:gene silencing by RNA;GO:0048451:petal formation;GO:0048453:sepal formation;GO:0051225:spindle assembly;GO:0051567:hi	GO:0005524:ATP binding;GO:0035402:histone kinase activity (H3-T11 specific);GO:0072354:histone kinase activity (H3-T3 specific)	GO:0005634:nucleus;GO:0005737:cytoplasm
Pp05_28899-3p(gma-miR1513a-5p)	18771985	PRUPE_ppb021895mg	NCBI_Assembly:GCF_000346465.1	NW_006760208.1	+	26883243	26883953	710	-	-	-	-

Pp05_28899-3p(gma-miR1513a-5p)	18771989	PRUPE_ppa000042mg	NCBI_Assembly:GCF_000346465.1	NW_006760208.1	+	28460783	28468191	7408	pper03040:Spliceosome	-	GO:0003676:nucleic acid binding;GO:0004386:helicase activity;GO:0005524:ATP binding	-
Pp05_28899-3p(gma-miR1513a-5p)	18772016	PRUPE_ppa009836mg	NCBI_Assembly:GCF_000346465.1	NW_006760208.1	-	20503287	20504997	1710	-	-	-	-
Pp05_28899-3p(gma-miR1513a-5p)	18772023	PRUPE_ppa012517mg	NCBI_Assembly:GCF_000346465.1	NW_006760208.1	-	2446550	2448638	2088	-	GO:0051252:regulation of RNA metabolic process	GO:0008428:ribonuclease inhibitor activity;GO:0008948:oxaloacetate decarboxylase activity;GO:0046872:metal ion binding;GO:0047443:4-hydroxy-4-methyl-2-oxoglutarate aldolase activity	-
Pp05_28899-3p(gma-miR1513a-5p)	18772048	PRUPE_ppa001036mg	NCBI_Assembly:GCF_000346465.1	NW_006760208.1	+	1508485	1512065	3580	-	-	-	-

Pp05_28899-3p(gma-miR1513a-5p)	18772065	PRUPE_ppa002099mg	NCBI_Assembly:GCF_000346465.1	NW_006760208.1	-	21775098	21778294	3196	pper00360:Phenylalanine metabolism;pper01100:Metabolic pathways;pper01110:Biosynthesis of secondary metabolites;pper00940:Phenylpropanoid biosynthesis	GO:0006559:L-phenylalanine catabolic process;GO:0009800:cinnamic acid biosynthetic process	GO:0045548:phenylalanine ammonia-lyase activity	GO:0005737:cytoplasm
Pp05_28899-3p(gma-miR1513a-5p)	18772117	PRUPE_ppa017934mg	NCBI_Assembly:GCF_000346465.1	NW_006760208.1	-	17587886	17589076	1190	-	-	-	-
Pp05_28899-3p(gma-miR1513a-5p)	18772127	PRUPE_ppa014252mg	NCBI_Assembly:GCF_000346465.1	NW_006760208.1	+	7006057	7006658	601	-	-	-	-
Pp05_28899-3p(gma-miR1513a-5p)	18772165	PRUPE_ppa005465mg	NCBI_Assembly:GCF_000346465.1	NW_006760208.1	+	2153581	2155437	1856	pper01100:Metabolic pathways	-	GO:0004497:monooxygenase activity;GO:0005506:iron ion binding;GO:0016705:oxidoreductase activity, acting on paired donors, with incorporation or reduction of molecular oxygen;GO:0020037:heme binding	-

Pp05_28899-3p(gma-miR1513a-5p)	18772212	PRUPE_ppa013748mg	NCBI_Assembly:GCF_000346465.1	NW_006760208.1	+	4683506	4685024	1518	-	-	-	-
Pp05_28899-3p(gma-miR1513a-5p)	18772215	PRUPE_ppa016189mg	NCBI_Assembly:GCF_000346465.1	NW_006760208.1	-	27755418	27756230	812	-	-	-	-
Pp05_28899-3p(gma-miR1513a-5p)	18772228	PRUPE_ppa005574mg	NCBI_Assembly:GCF_000346465.1	NW_006760208.1	-	24275804	24278006	2202	pper01212:Fatty acid metabolism;pper01040:Biosynthesis of unsaturated fatty acids	GO:0006629:lipid metabolic process	GO:0016717:oxidoreductase activity, acting on paired donors, with oxidation of a pair of donors resulting in the reduction of molecular oxygen to two molecules of water	-

Pp05_28899-3p(gma-miR1513a-5p)	18772293	PRUPE_ppa003438mg	NCBI_Assembly:GCF_000346465.1	NW_006760208.1	+	24359484	24363510	4026	-	GO:0006457: protein folding;GO:0009644:response to high light intensity;GO:0009688:abscisic acid biosynthetic process;GO:0009860:pollen tube growth;GO:0010286:heat acclimation;GO:0034605: cellular response to heat;GO:0042542:response to hydrogen peroxide	GO:0016491: oxidoreductase activity	GO:0005788: endoplasmic reticulum lumen;GO:0009506:plasmodesma
Pp05_28899-3p(gma-miR1513a-5p)	18772310	PRUPE_ppb019693mg	NCBI_Assembly:GCF_000346465.1	NW_006760208.1	-	27500735	27501420	685	-	-	-	-
Pp05_28899-3p(gma-miR1513a-5p)	18772312	PRUPE_ppa005524mg	NCBI_Assembly:GCF_000346465.1	NW_006760208.1	+	23087122	23091454	4332	-	-	-	-
Pp05_28899-3p(gma-miR1513a-5p)	18772319	PRUPE_ppa003728mg	NCBI_Assembly:GCF_000346465.1	NW_006760208.1	-	20048926	20051504	2578	-	-	GO:0003677: DNA binding;GO:0003682:chromatin binding	-
Pp05_28899-3p(gma-miR1513a-5p)	18772409	PRUPE_ppa018485mg	NCBI_Assembly:GCF_000346465.1	NW_006760208.1	-	22582504	22583182	678	-	GO:0006635: fatty acid beta-oxidation	-	-

Pp05_28899-3p(gma-miR1513a-5p)	18772448	PRUPE_ppa008584mg	NCBI_Assembly:GCF_000346465.1	NW_006760208.1	+	24230296	24231786	1490	-	GO:0006457:protein folding	-	-
Pp05_28899-3p(gma-miR1513a-5p)	18772460	PRUPE_ppa019413mg	NCBI_Assembly:GCF_000346465.1	NW_006760208.1	+	14102785	14103141	356	-	-	-	-
Pp05_28899-3p(gma-miR1513a-5p)	18772500	PRUPE_ppa014208mg	NCBI_Assembly:GCF_000346465.1	NW_006760208.1	-	18580401	18581269	868	-	-	-	-
Pp05_28899-3p(gma-miR1513a-5p)	18772650	PRUPE_ppa007264mg	NCBI_Assembly:GCF_000346465.1	NW_006760208.1	-	27666692	27670326	3634	pper01100:Metabolic pathways;pper01110:Biosynthesis of secondary metabolites;pper00053:Ascorbate and aldarate metabolism;pper00520:Amino sugar and nucleotide sugar metabolism	-	GO:0047918:GDP-mannose 3,5-epimerase activity;GO:0050662:coenzyme binding	-
Pp05_28899-3p(gma-miR1513a-5p)	18772670	PRUPE_ppa018073mg	NCBI_Assembly:GCF_000346465.1	NW_006760208.1	-	21084305	21085445	1140	-	-	-	-
Pp05_28899-3p(gma-miR1513a-5p)	18772689	PRUPE_ppa1027151mg	NCBI_Assembly:GCF_000346465.1	NW_006760208.1	-	2307551	2311492	3941	-	-	-	-
Pp05_28899-3p(gma-miR1513a-5p)	18772695	PRUPE_ppa004927mg	NCBI_Assembly:GCF_000346465.1	NW_006760208.1	+	3452372	3454982	2610	-	-	-	GO:0005886:plasma membrane

Pp05_28899-3p(gma-miR1513a-5p)	18772701	PRUPE_ppa025891mg	NCBI_Assembly:GCF_000346465.1	NW_006760208.1	-	23849426	23852003	2577	-	GO:0006857: oligopeptide transport	GO:0005215: transporter activity	GO:0016021: integral component of membrane
Pp05_28899-3p(gma-miR1513a-5p)	18772710	PRUPE_ppa014358mg	NCBI_Assembly:GCF_000346465.1	NW_006760208.1	+	24324445	24326164	1719	-	-	-	-
Pp05_28899-3p(gma-miR1513a-5p)	18772831	PRUPE_ppa017028mg	NCBI_Assembly:GCF_000346465.1	NW_006760208.1	-	27842932	27843327	395	-	-	-	-
Pp05_28899-3p(gma-miR1513a-5p)	18772833	PRUPE_ppa003720mg	NCBI_Assembly:GCF_000346465.1	NW_006760208.1	-	2323342	2328037	4695	-	GO:0000956: nuclear-transcribed mRNA catabolic process;GO:0006487:protein N-linked glycosylation	-	-
Pp05_28899-3p(gma-miR1513a-5p)	18772835	PRUPE_ppa006830mg	NCBI_Assembly:GCF_000346465.1	NW_006760208.1	-	27774221	27777190	2969	-	-	-	GO:0009507: chloroplast
Pp05_28899-3p(gma-miR1513a-5p)	18772837	PRUPE_ppb023695mg	NCBI_Assembly:GCF_000346465.1	NW_006760208.1	-	23196406	23196913	507	-	-	GO:0030246: carbohydrate binding	-

Pp05_28899-3p(gma-miR1513a-5p)	18772848	PRUPE_ppa012512mg	NCBI_Assembly:GCF_000346465.1	NW_006760208.1	-	28597961	28600262	2301	-	GO:0051252:regulation of RNA metabolic process	GO:0008428:ribonuclease inhibitor activity;GO:0008948:oxaloacetate decarboxylase activity;GO:0046872:metal ion binding;GO:0047443:4-hydroxy-4-methyl-2-oxoglutarate aldolase activity	-
Pp05_28899-3p(gma-miR1513a-5p)	18772858	PRUPE_ppa017544mg	NCBI_Assembly:GCF_000346465.1	NW_006760208.1	-	27141712	27145059	3347	-	-	GO:0004725:protein tyrosine phosphatase activity	-
Pp05_28899-3p(gma-miR1513a-5p)	18772879	PRUPE_ppa008802mg	NCBI_Assembly:GCF_000346465.1	NW_006760208.1	+	23370292	23371910	1618	-	-	GO:0016491:oxidoreductase activity	-
Pp05_28899-3p(gma-miR1513a-5p)	18772909	PRUPE_ppa004732mg	NCBI_Assembly:GCF_000346465.1	NW_006760208.1	-	781843	785114	3271	-	GO:0005975:carbohydrate metabolic process	GO:0004553:hydrolase activity, hydrolyzing O-glycosyl compounds	GO:0046658:anchored component of plasma membrane
Pp05_28899-3p(gma-miR1513a-5p)	18774397	PRUPE_ppb002198mg	NCBI_Assembly:GCF_000346465.1	NW_006760208.1	-	24660024	24661942	1918	-	-	-	-
Pp05_28899-3p(gma-miR1513a-5p)	18774402	PRUPE_ppa007701mg	NCBI_Assembly:GCF_000346465.1	NW_006760208.1	+	21631550	21633284	1734	pper03022:Basal transcription factors	-	-	-
Pp05_28899-3p(gma-miR1513a-5p)	18774403	PRUPE_ppa001860mg	NCBI_Assembly:GCF_000346465.1	NW_006760208.1	+	19141917	19148117	6200	-	GO:0055085:transmembrane transport	GO:0005215:transporter activity	GO:0005886:plasma membrane

Pp05_28899-3p(gma-miR1513a-5p)	18774413	PRUPE_ppa014662mg	NCBI_Assembly:GCF_000346465.1	NW_006760208.1	-	24818164	24820320	2156	-	GO:0006629: lipid metabolic process	GO:0016787: hydrolase activity	-
Pp05_28899-3p(gma-miR1513a-5p)	18774476	PRUPE_ppa018907mg	NCBI_Assembly:GCF_000346465.1	NW_006760208.1	-	10822570	10828704	6134	-	-	GO:0003676: nucleic acid binding	-
Pp05_28899-3p(gma-miR1513a-5p)	18775802	PRUPE_ppa023896mg	NCBI_Assembly:GCF_000346465.1	NW_006760212.1	-	1067576	1069267	1691	-	GO:0045454: cell redox homeostasis	-	GO:0005623: cell
Pp05_28899-3p(gma-miR1513a-5p)	18775812	PRUPE_ppa013321mg	NCBI_Assembly:GCF_000346465.1	NW_006760212.1	+	17955902	17957728	1826	-	GO:0006886: intracellular protein transport	-	GO:0005783: endoplasmic reticulum;GO:0016021:integral component of membrane
Pp05_28899-3p(gma-miR1513a-5p)	18775830	PRUPE_ppa000836mg	NCBI_Assembly:GCF_000346465.1	NW_006760212.1	+	10650619	10655953	5334	-	GO:0030001: metal ion transport	GO:0005507: copper ion binding;GO:0005524:ATP binding;GO:0019829:cation-transporting ATPase activity	GO:0016021: integral component of membrane
Pp05_28899-3p(gma-miR1513a-5p)	18775857	PRUPE_ppa022971mg	NCBI_Assembly:GCF_000346465.1	NW_006760212.1	-	5547349	5548070	721	-	-	GO:0004672: protein kinase activity;GO:0005524:ATP binding	-
Pp05_28899-3p(gma-miR1513a-5p)	18775908	PRUPE_ppa012406mg	NCBI_Assembly:GCF_000346465.1	NW_006760212.1	-	4958735	4960348	1613	-	-	-	-
Pp05_28899-3p(gma-miR1513a-5p)	18775914	PRUPE_ppa010445mg	NCBI_Assembly:GCF_000346465.1	NW_006760212.1	-	297512	299720	2208	-	-	-	-

Pp05_28899-3p(gma-miR1513a-5p)	18775968	PRUPE_ppa013054mg	NCBI_Assembly:GCF_000346465.1	NW_006760212.1	-	13294394	13295398	1004	-	GO:0006633: fatty acid biosynthetic process	-	-
Pp05_28899-3p(gma-miR1513a-5p)	18775971	PRUPE_ppa004567mg	NCBI_Assembly:GCF_000346465.1	NW_006760212.1	-	18119572	18122886	3314	pper01100:Metabolic pathways;pper01110:Biosynthesis of secondary metabolites;pper00430:Taurine and hypotaurine metabolism;pper00410:beta-Alanine metabolism;pper00650:Butanoate metabolism;pper00250:Alanine, aspartate and glutamate metabolism	GO:0006536: glutamate metabolic process	GO:0004351: glutamate decarboxylase activity;GO:0030170:pyridoxal phosphate binding	GO:0009506: plasmodesma
Pp05_28899-3p(gma-miR1513a-5p)	18775976	PRUPE_ppa020510mg	NCBI_Assembly:GCF_000346465.1	NW_006760212.1	-	13866988	13867934	946	-	-	-	-
Pp05_28899-3p(gma-miR1513a-5p)	18776028	PRUPE_ppa001299mg	NCBI_Assembly:GCF_000346465.1	NW_006760212.1	-	3389487	3394340	4853	-	-	-	-
Pp05_28899-3p(gma-miR1513a-5p)	18776029	PRUPE_ppa023181mg	NCBI_Assembly:GCF_000346465.1	NW_006760212.1	-	11969044	11969388	344	-	GO:0006869: lipid transport	GO:0008289: lipid binding	-

Pp05_28899-3p(gma-miR1513a-5p)	18776060	PRUPE_ppa022963mg	NCBI_Assembly:GCF_000346465.1	NW_006760212.1	-	16100373	16102258	1885	-	GO:0009704:de-etiolation	GO:0003690:double-stranded DNA binding;GO:0003700:sequence-specific DNA binding transcription factor activity	GO:0016607:nuclear speck
Pp05_28899-3p(gma-miR1513a-5p)	18776069	PRUPE_ppa005149mg	NCBI_Assembly:GCF_000346465.1	NW_006760212.1	+	12446925	12449724	2799	-	GO:0006499:N-terminal protein myristoylation	-	GO:0005886:plasma membrane
Pp05_28899-3p(gma-miR1513a-5p)	18776084	PRUPE_ppa021447mg	NCBI_Assembly:GCF_000346465.1	NW_006760212.1	-	12815551	12817032	1481	pper01100:Metabolic pathways;pper01110:Biosynthesis of secondary metabolites;pper00940:Phenylpropanoid biosynthesis	-	GO:0008171:O-methyltransferase activity	GO:0005829:cytosol
Pp05_28899-3p(gma-miR1513a-5p)	18776102	PRUPE_ppa002334mg	NCBI_Assembly:GCF_000346465.1	NW_006760212.1	+	8954769	8958305	3536	pper01100:Metabolic pathways;pper00500:Starch and sucrose metabolism;pper00052:Galactose metabolism	GO:0005975:carbohydrate metabolic process	GO:0004575:sucrose alpha-glucosidase activity	-
Pp05_28899-3p(gma-miR1513a-5p)	18776107	PRUPE_ppa012893mg	NCBI_Assembly:GCF_000346465.1	NW_006760212.1	-	2944530	2947197	2667	-	-	-	-

Pp05_28899-3p(gma-miR1513a-5p)	18776924	PRUPE_ppa026447mg	NCBI_Assembly:GCF_000346465.1	NW_006760212.1	+	17334470	17335909	1439	-	-	GO:0008375: acetylglucosaminyltransferase activity	GO:0016020: membrane
Pp05_28899-3p(gma-miR1513a-5p)	18777222	PRUPE_ppa003899mg	NCBI_Assembly:GCF_000346465.1	NW_006760212.1	-	13057393	13060169	2776	pper04141:Protein processing in endoplasmic reticulum;pper04145:Phagosome	GO:0006457: protein folding	GO:0005509: calcium ion binding	GO:0005783: endoplasmic reticulum
Pp05_28899-3p(gma-miR1513a-5p)	18777235	PRUPE_ppa006213mg	NCBI_Assembly:GCF_000346465.1	NW_006760212.1	+	170178	172338	2160	-	-	-	GO:0005886: plasma membrane
Pp05_28899-3p(gma-miR1513a-5p)	18777255	PRUPE_ppa001256mg	NCBI_Assembly:GCF_000346465.1	NW_006760212.1	-	15345311	15347972	2661	-	-	-	-
Pp05_28899-3p(gma-miR1513a-5p)	18777269	PRUPE_ppa006454m1g	NCBI_Assembly:GCF_000346465.1	NW_006760212.1	+	6536189	6536288	99	-	-	-	-
Pp05_28899-3p(gma-miR1513a-5p)	18777361	PRUPE_ppa002459mg	NCBI_Assembly:GCF_000346465.1	NW_006760212.1	-	11517333	11519670	2337	-	-	-	-

Pp05_28899-3p(gma-miR1513a-5p)	18777376	PRUPE_ppa006960mg	NCBI_Assembly:GCF_000346465.1	NW_006760212.1	+	8947078	8952344	5266	pper01100:Metabolic pathways;pper01110:Biosynthesis of secondary metabolites;pper01230:Biosynthesis of amino acids;pper01200:Carbon metabolism;pper00030:Pentose phosphate pathway	GO:0005975:carbohydrate metabolic process	GO:0003824:catalytic activity	-
Pp05_28899-3p(gma-miR1513a-5p)	18777496	PRUPE_ppa009609mg	NCBI_Assembly:GCF_000346465.1	NW_006760212.1	-	15671370	15674389	3019	-	-	-	-
Pp05_28899-3p(gma-miR1513a-5p)	18777503	PRUPE_ppa005959mg	NCBI_Assembly:GCF_000346465.1	NW_006760212.1	+	15161443	15163800	2357	pper04626:Plant-pathogen interaction	-	-	-
Pp05_28899-3p(gma-miR1513a-5p)	18777505	PRUPE_ppa009587mg	NCBI_Assembly:GCF_000346465.1	NW_006760212.1	-	16939825	16941559	1734	pper03013:RNA transport	-	GO:0003924:GTPase activity;GO:0005525:GTP binding	-
Pp05_28899-3p(gma-miR1513a-5p)	18777507	PRUPE_ppa012129mg	NCBI_Assembly:GCF_000346465.1	NW_006760212.1	-	233905	236374	2469	-	-	-	-

Pp05_28899-3p(gma-miR1513a-5p)	18777512	PRUPE_ppa025181mg	NCBI_Assembly:GCF_000346465.1	NW_006760212.1	+	15396292	15398624	2332	pper01100:Metabolic pathways;pper01110:Biosynthesis of secondary metabolites;pper00904:Diterpenoid biosynthesis	-	GO:0004497:monooxygenase activity;GO:0005506:iron ion binding;GO:0016705:oxidoreductase activity, acting on paired donors, with incorporation or reduction of molecular oxygen;GO:0020037:heme binding	-

Pp05_28899-3p(gma-miR1513a-5p)	18777568	PRUPE_ppa022781mg	NCBI_Assembly:GCF_000346465.1	NW_006760212.1	+	17809862	17812284	2422	-	GO:0000023:maltose metabolic process;GO:0006098:pentose-phosphate shunt;GO:0009902:chloroplast relocation;GO:0010027:thylakoid membrane organization;GO:0010190:cytochrome b6f complex assembly;GO:0010207:photosystem II assembly;GO:0019252:starch biosynthetic process;GO:0034660:ncRNA metabolic process;GO:0043085:positive regulation of catalytic activity	-	GO:0009535:chloroplast thylakoid membrane
Pp05_28899-3p(gma-miR1513a-5p)	18777582	PRUPE_ppa026895mg	NCBI_Assembly:GCF_000346465.1	NW_006760212.1	-	16689040	16690005	965	-	-	GO:0008146:sulfotransferase activity	-
Pp05_28899-3p(gma-miR1513a-5p)	18777595	PRUPE_ppa002488mg	NCBI_Assembly:GCF_000346465.1	NW_006760212.1	-	4963392	4965409	2017	pper03008:Ribosome biogenesis in eukaryotes	GO:0042254:ribosome biogenesis	GO:0005525:GTP binding	GO:0005730:nucleolus

Pp05_28899-3p(gma-miR1513a-5p)	18777628	PRUPE_ppa004159mg	NCBI_Assembly:GCF_000346465.1	NW_006760212.1	+	10609296	10612451	3155	-	-	-	GO:0005768:endosome;GO:0005802:trans-Golgi network
Pp05_28899-3p(gma-miR1513a-5p)	18777631	PRUPE_ppa001696mg	NCBI_Assembly:GCF_000346465.1	NW_006760212.1	+	841896	850310	8414	-	GO:0006893:Golgi to plasma membrane transport	-	GO:0000145:exocyst
Pp05_28899-3p(gma-miR1513a-5p)	18777678	PRUPE_ppa026664mg	NCBI_Assembly:GCF_000346465.1	NW_006760212.1	-	1873254	1874804	1550	-	-	GO:0004497:monooxygenase activity;GO:0005506:iron ion binding;GO:0016705:oxidoreductase activity, acting on paired donors, with incorporation or reduction of molecular oxygen;GO:0020037:heme binding	-
Pp05_28899-3p(gma-miR1513a-5p)	18777699	PRUPE_ppa024803mg	NCBI_Assembly:GCF_000346465.1	NW_006760212.1	-	12858132	12859367	1235	-	GO:0030001:metal ion transport	GO:0046872:metal ion binding	-
Pp05_28899-3p(gma-miR1513a-5p)	18777721	PRUPE_ppa022818mg	NCBI_Assembly:GCF_000346465.1	NW_006760212.1	+	17899514	17903700	4186	-	-	GO:0015079:potassium ion transmembrane transporter activity	GO:0016021:integral component of membrane

Pp05_28899-3p(gma-miR1513a-5p)	18777745	PRUPE_ppa003105mg	NCBI_Assembly:GCF_000346465.1	NW_006760212.1	+		15399678	15403518	3840	-	GO:0009855:determination of bilateral symmetry;GO:0009887:organ morphogenesis;GO:0010051:xylem and phloem pattern formation;GO:0048439:flower morphogenesis;GO:0048519:negative regulation of biological process	GO:0004674:protein serine/threonine kinase activity;GO:0005524:ATP binding	GO:0016021:integral component of membrane
Pp05_28899-3p(gma-miR1513a-5p)	18777746	PRUPE_ppa018068mg	NCBI_Assembly:GCF_000346465.1	NW_006760212.1	-		16653056	16654021	965	-	-	GO:0008146:sulfotransferase activity	-
Pp05_28899-3p(gma-miR1513a-5p)	18777794	PRUPE_ppa023517mg	NCBI_Assembly:GCF_000346465.1	NW_006760212.1	-		12927648	12928132	484	-	-	GO:0008270:zinc ion binding;GO:0016491:oxidoreductase activity	-
Pp05_28899-3p(gma-miR1513a-5p)	18777845	PRUPE_ppa022713mg	NCBI_Assembly:GCF_000346465.1	NW_006760212.1	+		13428015	13433625	5610	-	-	-	-
Pp05_28899-3p(gma-miR1513a-5p)	18777855	PRUPE_ppa014334mg	NCBI_Assembly:GCF_000346465.1	NW_006760212.1	-		13480520	13481376	856	-	-	-	-
Pp05_28899-3p(gma-miR1513a-5p)	18777859	PRUPE_ppa018620mg	NCBI_Assembly:GCF_000346465.1	NW_006760212.1	+		16887206	16887520	314	-	-	-	-

Pp05_28899-3p(gma-miR1513a-5p)	18777874	PRUPE_ppa005339mg	NCBI_Assembly:GCF_000346465.1	NW_006760212.1	+	15823666	15826061	2395	-	GO:0006355: regulation of transcription, DNA-templated	GO:0003677: DNA binding	-
Pp05_28899-3p(gma-miR1513a-5p)	18777882	PRUPE_ppa000386mg	NCBI_Assembly:GCF_000346465.1	NW_006760212.1	-	17156116	17162638	6522	-	GO:0006886: intracellular protein transport;GO:0016192:vesicle-mediated transport	GO:0005198: structural molecule activity	GO:0030126: COPI vesicle coat
Pp05_28899-3p(gma-miR1513a-5p)	18777908	PRUPE_ppa006554mg	NCBI_Assembly:GCF_000346465.1	NW_006760212.1	+	14227371	14230776	3405	-	GO:0007568: aging;GO:0009651:response to salt stress;GO:0009845:seed germination	GO:0004719: protein-L-isoaspartate (D-aspartate) O-methyltransferase activity	GO:0005829: cytosol
Pp05_28899-3p(gma-miR1513a-5p)	18777915	PRUPE_ppa000817mg	NCBI_Assembly:GCF_000346465.1	NW_006760212.1	+	13047101	13053605	6504	-	GO:0035194: posttranscriptional gene silencing by RNA	GO:0003712: transcription cofactor activity	GO:0016442: RISC complex
Pp05_28899-3p(gma-miR1513a-5p)	18777933	PRUPE_ppa006106mg	NCBI_Assembly:GCF_000346465.1	NW_006760212.1	+	18345471	18351122	5651	-	-	-	-
Pp05_28899-3p(gma-miR1513a-5p)	18777982	PRUPE_ppa010432mg	NCBI_Assembly:GCF_000346465.1	NW_006760212.1	-	1828794	1829857	1063	-	-	-	-
Pp05_28899-3p(gma-miR1513a-5p)	18777991	PRUPE_ppa011001mg	NCBI_Assembly:GCF_000346465.1	NW_006760212.1	+	6736793	6742010	5217	-	-	-	-

Pp05_28899-3p(gma-miR1513a-5p)	18777993	PRUPE_ppa001418mg	NCBI_Assembly:GCF_000346465.1	NW_006760212.1	+	860082	864310	4228	pper04141:Protein processing in endoplasmic reticulum	GO:0006397:mRNA processing	GO:0004540:ribonuclease activity;GO:0004672:protein kinase activity;GO:0005524:ATP binding	-
Pp05_28899-3p(gma-miR1513a-5p)	18778005	PRUPE_ppa018739mg	NCBI_Assembly:GCF_000346465.1	NW_006760212.1	+	7802715	7804481	1766	-	-	-	-
Pp05_28899-3p(gma-miR1513a-5p)	18778068	PRUPE_ppa025452mg	NCBI_Assembly:GCF_000346465.1	NW_006760212.1	+	9181699	9182178	479	-	-	-	-
Pp05_28899-3p(gma-miR1513a-5p)	18778082	PRUPE_ppa008250mg	NCBI_Assembly:GCF_000346465.1	NW_006760212.1	-	13923035	13926433	3398	pper01100:Metabolic pathways;pper01110:Biosynthesis of secondary metabolites;pper01230:Biosynthesis of amino acids;pper01200:Carbon metabolism;pper00010:Glycolysis / Gluconeogenesis;pper00710:Carbon fixation in photosynthetic organisms	GO:0006006:glucose metabolic process	GO:0016620:oxidoreductase activity, acting on the aldehyde or oxo group of donors, NAD or NADP as acceptor;GO:0050661:NADP binding;GO:0051287:NAD binding	-
Pp05_28899-3p(gma-miR1513a-5p)	18778086	PRUPE_ppa018561mg	NCBI_Assembly:GCF_000346465.1	NW_006760212.1	-	14446181	14447576	1395	-	-	GO:0003677:DNA binding;GO:0003682:chromatin binding	-

Pp05_28899-3p(gma-miR1513a-5p)	18778135	PRUPE_ppa021893mg	NCBI_Assembly:GCF_000346465.1	NW_006760219.1	+	280	837	557	-	-	-	-
Pp05_28899-3p(gma-miR1513a-5p)	18778173	PRUPE_ppa026781mg	NCBI_Assembly:GCF_000346465.1	NW_006760220.1	+	1282715	1285615	2900	-	-	GO:0004674:protein serine/threonine kinase activity;GO:0005524:ATP binding	-
Pp05_28899-3p(gma-miR1513a-5p)	18778227	PRUPE_ppa014083mg	NCBI_Assembly:GCF_000346465.1	NW_006760220.1	+	3965205	3966162	957	-	-	-	-
Pp05_28899-3p(gma-miR1513a-5p)	18778231	PRUPE_ppa014087mg	NCBI_Assembly:GCF_000346465.1	NW_006760220.1	+	11182744	11183404	660	-	-	-	-
Pp05_28899-3p(gma-miR1513a-5p)	18778237	PRUPE_ppa008302mg	NCBI_Assembly:GCF_000346465.1	NW_006760220.1	+	12985400	12988280	2880	-	GO:0006979:response to oxidative stress	-	GO:0009535:chloroplast thylakoid membrane;GO:0031977:thylakoid lumen
Pp05_28899-3p(gma-miR1513a-5p)	18778267	PRUPE_ppa007884mg	NCBI_Assembly:GCF_000346465.1	NW_006760220.1	-	7780642	7782382	1740	-	-	GO:0008270:zinc ion binding	-
Pp05_28899-3p(gma-miR1513a-5p)	18778282	PRUPE_ppa011448mg	NCBI_Assembly:GCF_000346465.1	NW_006760220.1	-	8881273	8882272	999	-	GO:0010167:response to nitrate;GO:0012501:programmed cell death;GO:0015706:nitrate transport;GO:0042742:defense response to bacterium	GO:0004866:endopeptidase inhibitor activity	GO:0005739:mitochondrion

Pp05_28899-3p(gma-miR1513a-5p)	18778331	PRUPE_ppa007355mg	NCBI_Assembly:GCF_000346465.1	NW_006760220.1	-	2092945	2095759	2814	-	-	GO:0004674:protein serine/threonine kinase activity;GO:0005524:ATP binding	-	
Pp05_28899-3p(gma-miR1513a-5p)	18778382	PRUPE_ppa006200mg	NCBI_Assembly:GCF_000346465.1	NW_006760220.1	-	2744782	2746835	2053	pper01100:Metabolic pathways;pper01110:Biosynthesis of secondary metabolites;pper00860:Porphyrin and chlorophyll metabolism	GO:0015979:photosynthesis;GO:0015995:chlorophyll biosynthetic process	GO:0005524:ATP binding;GO:0016851:magnesium chelatase activity	-	
Pp05_28899-3p(gma-miR1513a-5p)	18778399	PRUPE_ppa022934mg	NCBI_Assembly:GCF_000346465.1	NW_006760220.1	-	1217947	1218804	857	-	-	-	-	
Pp05_28899-3p(gma-miR1513a-5p)	18778406	PRUPE_ppa022811mg	NCBI_Assembly:GCF_000346465.1	NW_006760220.1	-	9122919	9124403	1484	-	-	GO:0016758:transferase activity, transferring hexosyl groups	-	
Pp05_28899-3p(gma-miR1513a-5p)	18778415	PRUPE_ppa006822mg	NCBI_Assembly:GCF_000346465.1	NW_006760220.1	+	3304826	3306070	1244	-	GO:0009553:embryo sac development;GO:0045892:negative regulation of transcription, DNA-templated	-	GO:0005730:nucleolus;GO:0030863:cortical cytoskeleton	
Pp05_28899-3p(gma-miR1513a-5p)	18778462	PRUPE_ppa013152mg	NCBI_Assembly:GCF_000346465.1	NW_006760220.1	-	3798505	3801362	2857	-	-	-	-	

Pp05_28899-3p(gma-miR1513a-5p)	18778556	PRUPE_ppa014686mg	NCBI_Assembly:GCF_000346465.1	NW_006760220.1	+	16890971	16892427	1456	-	-	-	-	
Pp05_28899-3p(gma-miR1513a-5p)	18778568	PRUPE_ppa003789mg	NCBI_Assembly:GCF_000346465.1	NW_006760220.1	+	23047134	23054125	6991	pper01100:Metabolic pathways;pper00240:Pyrimidine metabolism;pper00770:Pantothenate and CoA biosynthesis;pper00410:beta-Alanine metabolism	-	GO:0016810:hydrolase activity, acting on carbon-nitrogen (but not peptide) bonds	GO:0005737:cytoplasm	
Pp05_28899-3p(gma-miR1513a-5p)	18778619	PRUPE_ppa022594mg	NCBI_Assembly:GCF_000346465.1	NW_006760220.1	-	7135079	7136420	1341	-	-	-	-	
Pp05_28899-3p(gma-miR1513a-5p)	18778631	PRUPE_ppa008430mg	NCBI_Assembly:GCF_000346465.1	NW_006760220.1	+	10868817	10871787	2970	-	GO:0008284:positive regulation of cell proliferation;GO:0009560:embryo sac egg cell differentiation	-	-	
Pp05_28899-3p(gma-miR1513a-5p)	18778640	PRUPE_ppa022486mg	NCBI_Assembly:GCF_000346465.1	NW_006760220.1	-	2187892	2189560	1668	-	-	-	-	

Pp05_28899-3p(gma-miR1513a-5p)	18778648	PRUPE_ppa001558mg	NCBI_Assembly:GCF_000346465.1	NW_006760220.1	+	27311988	27325128	13140	-	GO:0006486:protein glycosylation;GO:0007062:sister chromatid cohesion;GO:0007129:synapsis;GO:0007131:reciprocal meiotic recombination;GO:0009887:organ morphogenesis;GO:0009888:tissue development;GO:0010332:response to gamma radiation;GO:0010413:glucuronoxylan metabolic process;GO:0010638:positive regulation of organelle organization;GO:0016926:protein desumoylation;GO:0032204:regulation of telomere maintenance;GO:0032504	-	-

Pp05_28899-3p(gma-miR1513a-5p)	18778661	PRUPE_ppa003305mg	NCBI_Assembly:GCF_000346465.1	NW_006760220.1	+	14951801	14956955	5154	-	GO:0009616:virus induced gene silencing;GO:0010050:vegetative phase change;GO:0045893:positive regulation of transcription, DNA-templated	GO:0003700:sequence-specific DNA binding transcription factor activity;GO:0043565:sequence-specific DNA binding	-
Pp05_28899-3p(gma-miR1513a-5p)	18778668	PRUPE_ppa006520mg	NCBI_Assembly:GCF_000346465.1	NW_006760220.1	-	7231630	7235188	3558	-	-	GO:0008324:cation transmembrane transporter activity	GO:0016021:integral component of membrane
Pp05_28899-3p(gma-miR1513a-5p)	18778702	PRUPE_ppa016544mg	NCBI_Assembly:GCF_000346465.1	NW_006760220.1	+	13759016	13767484	8468	-	GO:0005978:glycogen biosynthetic process;GO:0009791:post-embryonic development	GO:0003844:1,4-alpha-glucan branching enzyme activity;GO:0043169:cation binding	-
Pp05_28899-3p(gma-miR1513a-5p)	18778705	PRUPE_ppa019953mg	NCBI_Assembly:GCF_000346465.1	NW_006760220.1	-	29309240	29310994	1754	-	-	GO:0003677:DNA binding	-
Pp05_28899-3p(gma-miR1513a-5p)	18778813	PRUPE_ppa010467mg	NCBI_Assembly:GCF_000346465.1	NW_006760220.1	+	6200529	6202990	2461	-	GO:0006807:nitrogen compound metabolic process;GO:0048554:positive regulation of metalloenzyme activity	GO:0016151:nickel cation binding	-

Pp05_28899-3p(gma-miR1513a-5p)	18778829	PRUPE_ppa025207mg	NCBI_Assembly:GCF_000346465.1	NW_006760220.1	-		21075204	21078986	3782	-	-	-	-
Pp05_28899-3p(gma-miR1513a-5p)	18778833	PRUPE_ppa010564mg	NCBI_Assembly:GCF_000346465.1	NW_006760220.1	+		514035	515906	1871	-	-	GO:0005094:Rho GDP-dissociation inhibitor activity	GO:0005737:cytoplasm
Pp05_28899-3p(gma-miR1513a-5p)	18778834	PRUPE_ppa015743mg	NCBI_Assembly:GCF_000346465.1	NW_006760220.1	-		7143900	7144998	1098	-	-	GO:0005385:zinc ion transmembrane transporter activity	GO:0016021:integral component of membrane
Pp05_28899-3p(gma-miR1513a-5p)	18778855	PRUPE_ppa025513mg	NCBI_Assembly:GCF_000346465.1	NW_006760220.1	-		3253140	3256335	3195	-	GO:0006817:phosphate ion transport	GO:0005315:inorganic phosphate transmembrane transporter activity	GO:0016020:membrane
Pp05_28899-3p(gma-miR1513a-5p)	18778905	PRUPE_ppa002450mg	NCBI_Assembly:GCF_000346465.1	NW_006760220.1	+		3003212	3007893	4681	-	-	GO:0004672:protein kinase activity;GO:0005524:ATP binding	GO:0016021:integral component of membrane
Pp05_28899-3p(gma-miR1513a-5p)	18778910	PRUPE_ppa008638mg	NCBI_Assembly:GCF_000346465.1	NW_006760220.1	+		2970280	2971462	1182	pper04146:Peroxisome	GO:0006635:fatty acid beta-oxidation;GO:0016558:protein import into peroxisome matrix	-	GO:0080008:Cul4-RING E3 ubiquitin ligase complex
Pp05_28899-3p(gma-miR1513a-5p)	18778934	PRUPE_ppa018478mg	NCBI_Assembly:GCF_000346465.1	NW_006760220.1	-		1021441	1023753	2312	-	-	-	-

Pp05_28899-3p(gma-miR1513a-5p)	18778964	PRUPE_ppa017157mg	NCBI_Assembly:GCF_000346465.1	NW_006760220.1	-	11240333	11241625	1292	pper01100:Metabolic pathways;pper00053:Ascorbate and aldarate metabolism	-	GO:0016491:oxidoreductase activity;GO:0050660:flavin adenine dinucleotide binding	-	
Pp05_28899-3p(gma-miR1513a-5p)	18778975	PRUPE_ppa013389mg	NCBI_Assembly:GCF_000346465.1	NW_006760220.1	+	12092348	12093189	841	-	-	-	-	
Pp05_28899-3p(gma-miR1513a-5p)	18778990	PRUPE_ppa001902mg	NCBI_Assembly:GCF_000346465.1	NW_006760220.1	+	7070520	7074410	3890	pper01100:Metabolic pathways;pper01110:Biosynthesis of secondary metabolites;pper00904:Diterpenoid biosynthesis	-	GO:0000287:magnesium ion binding;GO:0010333:terpene synthase activity	-	
Pp05_28899-3p(gma-miR1513a-5p)	18779004	PRUPE_ppa025488mg	NCBI_Assembly:GCF_000346465.1	NW_006760220.1	+	922500	923907	1407	pper04140:Regulation of autophagy	GO:0006914:autophagy	-	-	
Pp05_28899-3p(gma-miR1513a-5p)	18779018	PRUPE_ppa019704mg	NCBI_Assembly:GCF_000346465.1	NW_006760220.1	-	29546458	29548667	2209	-	-	-	-	
Pp05_28899-3p(gma-miR1513a-5p)	18779042	PRUPE_ppa018332mg	NCBI_Assembly:GCF_000346465.1	NW_006760220.1	-	16028150	16030147	1997	-	-	GO:0005524:ATP binding;GO:0016887:ATPase activity	-	
Pp05_28899-3p(gma-miR1513a-5p)	18779080	PRUPE_ppa006676mg	NCBI_Assembly:GCF_000346465.1	NW_006760220.1	-	27572634	27581073	8439	-	GO:0006886:intracellular protein transport	-	GO:0005622:intracellular;GO:0005886:plasma membrane	
Pp05_28899-3p(gma-miR1513a-5p)	18779105	PRUPE_ppa012071mg	NCBI_Assembly:GCF_000346465.1	NW_006760220.1	-	5644596	5645799	1203	-	-	-	-	

Pp05_28899-3p(gma-miR1513a-5p)	18779108	PRUPE_ppa027134mg	NCBI_Assembly:GCF_000346465.1	NW_006760220.1	+	12840810	12844399	3589	-	-	GO:0008289: lipid binding	-
Pp05_28899-3p(gma-miR1513a-5p)	18779121	PRUPE_ppa026035mg	NCBI_Assembly:GCF_000346465.1	NW_006760220.1	-	18284228	18286865	2637	-	-	-	-
Pp05_28899-3p(gma-miR1513a-5p)	18779129	PRUPE_ppa006862mg	NCBI_Assembly:GCF_000346465.1	NW_006760220.1	+	1839035	1841863	2828	pper01100:Metabolic pathways;pper00920:Sulfur metabolism	GO:0006790: sulfur compound metabolic process;GO:0046854:phosphatidylinositol phosphorylation	GO:0008441: 3'(2'),5'-bisphosphate nucleotidase activity	-
Pp05_28899-3p(gma-miR1513a-5p)	18779136	PRUPE_ppa015696mg	NCBI_Assembly:GCF_000346465.1	NW_006760220.1	-	4679201	4681526	2325	-	-	GO:0004674: protein serine/threonine kinase activity;GO:0005524:ATP binding;GO:0030247:polysaccharide binding	-

Pp05_28899-3p(gma-miR1513a-5p)	18779147	PRUPE_ppa000091mg	NCBI_Assembly:GCF_000346465.1	NW_006760220.1	-		10268913	10278408	9495	-	GO:0000226:microtubule cytoskeleton organization;GO:0000911:cytokinesis by cell plate formation;GO:0009887:organ morphogenesis;GO:0009888:tissue development;GO:0010638:positive regulation of organelle organization;GO:0033044:regulation of chromosome organization	GO:0005524:ATP binding	GO:0009506:plasmodesma
Pp05_28899-3p(gma-miR1513a-5p)	18779164	PRUPE_ppa006643mg	NCBI_Assembly:GCF_000346465.1	NW_006760220.1	+		9765004	9768326	3322	-	-	-	-
Pp05_28899-3p(gma-miR1513a-5p)	18779187	PRUPE_ppa010388mg	NCBI_Assembly:GCF_000346465.1	NW_006760220.1	-		9009363	9012115	2752	-	-	-	-
Pp05_28899-3p(gma-miR1513a-5p)	18779191	PRUPE_ppb018758mg	NCBI_Assembly:GCF_000346465.1	NW_006760220.1	+		1320580	1321350	770	-	-	-	-
Pp05_28899-3p(gma-miR1513a-5p)	18779205	PRUPE_ppa007088mg	NCBI_Assembly:GCF_000346465.1	NW_006760220.1	-		3959911	3961403	1492	-	GO:0005975:carbohydrate metabolic process	GO:0004553:hydrolase activity, hydrolyzing O-glycosyl compounds	-

Pp05_28899-3p(gma-miR1513a-5p)	18779232	PRUPE_ppa019429mg	NCBI_Assembly:GCF_000346465.1	NW_006760220.1	-	27191861	27195995	4134	-	GO:0005975:carbohydrate metabolic process	GO:0004553:hydrolase activity, hydrolyzing O-glycosyl compounds	-
Pp05_28899-3p(gma-miR1513a-5p)	18779267	PRUPE_ppa006857mg	NCBI_Assembly:GCF_000346465.1	NW_006760220.1	+	22684500	22687159	2659	pper01100:Metabolic pathways;pper00500:Starch and sucrose metabolism;pper00040:Pentose and glucuronate interconversions	GO:0005975:carbohydrate metabolic process;GO:0071555:cell wall organization	GO:0004650:polygalacturonase activity	GO:0005576:extracellular region
Pp05_28899-3p(gma-miR1513a-5p)	18779284	PRUPE_ppa004738mg	NCBI_Assembly:GCF_000346465.1	NW_006760220.1	-	7329067	7331702	2635	-	GO:0006364:rRNA processing;GO:0006606:protein import into nucleus;GO:0009561:megagametogenesis;GO:0009791:post-embryonic development	GO:0003676:nucleic acid binding;GO:0004386:helicase activity;GO:0005524:ATP binding	GO:0005634:nucleus;GO:0090406:pollen tube
Pp05_28899-3p(gma-miR1513a-5p)	18779297	PRUPE_ppa022346mg	NCBI_Assembly:GCF_000346465.1	NW_006760220.1	+	20203741	20206199	2458	-	-	-	-
Pp05_28899-3p(gma-miR1513a-5p)	18779307	PRUPE_ppb020639mg	NCBI_Assembly:GCF_000346465.1	NW_006760220.1	+	2614045	2615091	1046	-	-	-	-
Pp05_28899-3p(gma-miR1513a-5p)	18779330	PRUPE_ppa021151mg	NCBI_Assembly:GCF_000346465.1	NW_006760220.1	-	27407147	27410197	3050	-	-	-	-

Pp05_28899-3p(gma-miR1513a-5p)	18779396	PRUPE_ppa007258mg	NCBI_Assembly:GCF_000346465.1	NW_006760220.1	-	2402287	2403910	1623	-	-	GO:0003677:DNA binding;GO:0003700:sequence-specific DNA binding transcription factor activity	-
Pp05_28899-3p(gma-miR1513a-5p)	18779412	PRUPE_ppb016606mg	NCBI_Assembly:GCF_000346465.1	NW_006760220.1	+	5476039	5479281	3242	-	-	-	-
Pp05_28899-3p(gma-miR1513a-5p)	18779488	PRUPE_ppa021974mg	NCBI_Assembly:GCF_000346465.1	NW_006760220.1	+	20606805	20608925	2120	-	-	-	-
Pp05_28899-3p(gma-miR1513a-5p)	18779505	PRUPE_ppb025216mg	NCBI_Assembly:GCF_000346465.1	NW_006760220.1	-	19216539	19218980	2441	-	-	-	-
Pp05_28899-3p(gma-miR1513a-5p)	18779524	PRUPE_ppa026626mg	NCBI_Assembly:GCF_000346465.1	NW_006760220.1	-	12907382	12907975	593	-	-	GO:0009055:electron carrier activity	-
Pp05_28899-3p(gma-miR1513a-5p)	18779525	PRUPE_ppa002305mg	NCBI_Assembly:GCF_000346465.1	NW_006760220.1	-	16579565	16583575	4010	-	GO:0006891:intra-Golgi vesicle-mediated transport	-	GO:0005829:cytosol;GO:0017119:Golgi transport complex
Pp05_28899-3p(gma-miR1513a-5p)	18779533	PRUPE_ppa014502mg	NCBI_Assembly:GCF_000346465.1	NW_006760220.1	+	30192609	30193116	507	-	GO:0005975:carbohydrate metabolic process	GO:0004650:polygalacturonase activity	-

Pp05_28899-3p(gma-miR1513a-5p)	18779550	PRUPE_ppa003970mg	NCBI_Assembly:GCF_000346465.1	NW_006760220.1	+	547679	552350	4671	pper03022:Basal transcription factors	GO:0006367:transcription initiation from RNA polymerase II promoter;GO:0015979:photosynthesis;GO:0032968:positive regulation of transcription elongation from RNA polymerase II promoter	GO:0003677:DNA binding;GO:0003824:catalytic activity	GO:0005634:nucleus;GO:0009522:photosystem I;GO:0016021:integral component of membrane
Pp05_28899-3p(gma-miR1513a-5p)	18779635	PRUPE_ppa016617mg	NCBI_Assembly:GCF_000346465.1	NW_006760220.1	-	4718440	4721173	2733	-	-	GO:0004672:protein kinase activity;GO:0005509:calcium ion binding;GO:0005524:ATP binding;GO:0030247:polysaccharide binding	-
Pp05_28899-3p(gma-miR1513a-5p)	18779662	PRUPE_ppa018894mg	NCBI_Assembly:GCF_000346465.1	NW_006760220.1	-	4775645	4778184	2539	-	-	GO:0030145:manganese ion binding;GO:0045735:nutrient reservoir activity	GO:0005576:extracellular region

Pp05_28899-3p(gma-miR1513a-5p)	18779667	PRUPE_ppa008211mg	NCBI_Assembly:GCF_000346465.1	NW_006760220.1	+	3917688	3919195	1507	pper00904:Diterpenoid biosynthesis	-	GO:0005506:iron ion binding;GO:0016706:oxidoreductase activity, acting on paired donors, with incorporation or reduction of molecular oxygen, 2-oxoglutarate as one donor, and incorporation of one atom each of oxygen into both donors	-
Pp05_28899-3p(gma-miR1513a-5p)	18779715	PRUPE_ppa007804mg	NCBI_Assembly:GCF_000346465.1	NW_006760220.1	+	15729379	15733205	3826	-	-	-	-
Pp05_28899-3p(gma-miR1513a-5p)	18779719	PRUPE_ppa002928mg	NCBI_Assembly:GCF_000346465.1	NW_006760220.1	+	27628234	27633408	5174	-	-	-	-
Pp05_28899-3p(gma-miR1513a-5p)	18779742	PRUPE_ppa010914mg	NCBI_Assembly:GCF_000346465.1	NW_006760220.1	+	3642539	3644481	1942	-	-	-	-
Pp05_28899-3p(gma-miR1513a-5p)	18779747	PRUPE_ppa019092mg	NCBI_Assembly:GCF_000346465.1	NW_006760220.1	+	2801100	2802706	1606	-	GO:0006629:lipid metabolic process	GO:0016298:lipase activity	-

Pp05_28899-3p(gma-miR1513a-5p)	18779762	PRUPE_ppa002439m2g	NCBI_Assembly:GCF_000346465.1	NW_006760220.1	-	15082891	15087217	4326	pper01100:Metabolic pathways;pper00071:Fatty acid degradation;pper01212:Fatty acid metabolism;pper04146:Peroxisome;pper00592:alpha-Linolenic acid metabolism;pper01040:Biosynthesis of unsaturated fatty acids	GO:0006635:fatty acid beta-oxidation	GO:0003995:acyl-CoA dehydrogenase activity;GO:0003997:acyl-CoA oxidase activity	GO:0005777:peroxisome
Pp05_28899-3p(gma-miR1513a-5p)	18779775	PRUPE_ppa015351mg	NCBI_Assembly:GCF_000346465.1	NW_006760220.1	+	1250906	1251586	680	-	-	-	-
Pp05_28899-3p(gma-miR1513a-5p)	18779871	PRUPE_ppa005787mg	NCBI_Assembly:GCF_000346465.1	NW_006760220.1	+	651733	654283	2550	-	-	-	-
Pp05_28899-3p(gma-miR1513a-5p)	18779875	PRUPE_ppa016292mg	NCBI_Assembly:GCF_000346465.1	NW_006760220.1	+	6884448	6887049	2601	-	-	GO:0000287:magnesium ion binding;GO:0010333:terpene synthase activity	-
Pp05_28899-3p(gma-miR1513a-5p)	18779886	PRUPE_ppa016075mg	NCBI_Assembly:GCF_000346465.1	NW_006760220.1	-	26914610	26915104	494	-	-	GO:0003676:nucleic acid binding	-

Pp05_28899-3p(gma-miR1513a-5p)	18780064	PRUPE_ppa017161mg	NCBI_Assembly:GCF_000346465.1	NW_006760220.1	-	4784296	4785071	775	-	-	GO:0030145:manganese ion binding;GO:0045735:nutrient reservoir activity	GO:0005576:extracellular region
Pp05_28899-3p(gma-miR1513a-5p)	18780081	PRUPE_ppa019148mg	NCBI_Assembly:GCF_000346465.1	NW_006760220.1	-	20600559	20602159	1600	pper01212:Fatty acid metabolism;pper01040:Biosynthesis of unsaturated fatty acids	GO:0006633:fatty acid biosynthetic process	GO:0016717:oxidoreductase activity, acting on paired donors, with oxidation of a pair of donors resulting in the reduction of molecular oxygen to two molecules of water	GO:0016021:integral component of membrane
Pp05_28899-3p(gma-miR1513a-5p)	18780099	PRUPE_ppb025232mg	NCBI_Assembly:GCF_000346465.1	NW_006760220.1	-	21853982	21854377	395	-	-	-	-
Pp05_28899-3p(gma-miR1513a-5p)	18780104	PRUPE_ppa027150m2g	NCBI_Assembly:GCF_000346465.1	NW_006760220.1	+	5016231	5016266	35	-	-	-	-
Pp05_28899-3p(gma-miR1513a-5p)	18780162	PRUPE_ppa020245mg	NCBI_Assembly:GCF_000346465.1	NW_006760220.1	-	2382512	2384719	2207	pper04146:Peroxisome	-	-	GO:0016021:integral component of membrane

Pp05_28899-3p(gma-miR1513a-5p)	18780175	PRUPE_ppa017318mg	NCBI_Assembly:GCF_000346465.1	NW_006760220.1	-	1089335	1097197	7862	-	GO:0000226:microtubule cytoskeleton organization;GO:0000911:cytokinesis by cell plate formation;GO:0006270:DNA replication initiation;GO:0006275:regulation of DNA replication;GO:0006306:DNA methylation;GO:0006346:methylation-dependent chromatin silencing;GO:0008283:cell proliferation;GO:0016246:RNA interference;GO:0031048:chromatin silencing by small RNA;GO:0048451:petal formation;GO:0048453:sepal formation;G	-	-
Pp05_28899-3p(gma-miR1513a-5p)	18780195	PRUPE_ppa006343mg	NCBI_Assembly:GCF_000346465.1	NW_006760220.1	-	7826922	7829255	2333	-	-	-	-

Pp05_28899-3p(gma-miR1513a-5p)	18780220	PRUPE_ppa019446mg	NCBI_Assembly:GCF_000346465.1	NW_006760220.1	+	4106164	4106490	326	-	GO:0045454: cell redox homeostasis	GO:0009055: electron carrier activity;GO:0015035:protein disulfide oxidoreductase activity	GO:0005623: cell
Pp05_28899-3p(gma-miR1513a-5p)	18780228	PRUPE_ppa021686mg	NCBI_Assembly:GCF_000346465.1	NW_006760220.1	-	25482989	25485098	2109	-	-	-	-
Pp05_28899-3p(gma-miR1513a-5p)	18780253	PRUPE_ppa026938mg	NCBI_Assembly:GCF_000346465.1	NW_006760220.1	-	20947178	20948280	1102	-	-	-	-
Pp05_28899-3p(gma-miR1513a-5p)	18780267	PRUPE_ppb015490mg	NCBI_Assembly:GCF_000346465.1	NW_006760220.1	+	17404090	17406948	2858	-	-	GO:0016758: transferase activity, transferring hexosyl groups	-
Pp05_28899-3p(gma-miR1513a-5p)	18780300	PRUPE_ppa001345mg	NCBI_Assembly:GCF_000346465.1	NW_006760220.1	-	27040118	27055295	15177	-	GO:0005975: carbohydrate metabolic process	GO:0004565: beta-galactosidase activity;GO:0030246:carbohydrate binding	GO:0009505: plant-type cell wall
Pp05_28899-3p(gma-miR1513a-5p)	18780348	PRUPE_ppa022992mg	NCBI_Assembly:GCF_000346465.1	NW_006760220.1	+	8946778	8949360	2582	-	GO:0006741: NADP biosynthetic process;GO:0019674:NAD metabolic process	GO:0003951: NAD+ kinase activity	-
Pp05_28899-3p(gma-miR1513a-5p)	18780352	PRUPE_ppa019363mg	NCBI_Assembly:GCF_000346465.1	NW_006760220.1	+	13254440	13255252	812	-	-	-	-
Pp05_28899-3p(gma-miR1513a-5p)	18780370	PRUPE_ppa016047mg	NCBI_Assembly:GCF_000346465.1	NW_006760220.1	+	1556024	1556362	338	-	-	GO:0005509: calcium ion binding	-

Pp05_28899-3p(gma-miR1513a-5p)	18780431	PRUPE_ppa022270mg	NCBI_Assembly:GCF_000346465.1	NW_006760220.1	+	7166022	7167104	1082	-	-	-	-
Pp05_28899-3p(gma-miR1513a-5p)	18780457	PRUPE_ppa018539mg	NCBI_Assembly:GCF_000346465.1	NW_006760220.1	+	23873323	23873685	362	-	-	GO:0030246:carbohydrate binding	-
Pp05_28899-3p(gma-miR1513a-5p)	18780458	PRUPE_ppa020220mg	NCBI_Assembly:GCF_000346465.1	NW_006760220.1	+	8367087	8367972	885	pper00480:Glutathione metabolism	-	-	-
Pp05_28899-3p(gma-miR1513a-5p)	18780485	PRUPE_ppa021215mg	NCBI_Assembly:GCF_000346465.1	NW_006760220.1	-	27609101	27610231	1130	-	-	-	-
Pp05_28899-3p(gma-miR1513a-5p)	18780528	PRUPE_ppa024747m1g	NCBI_Assembly:GCF_000346465.1	NW_006760220.1	-	28822168	28822833	665	-	-	-	-
Pp05_28899-3p(gma-miR1513a-5p)	18780533	PRUPE_ppa026254mg	NCBI_Assembly:GCF_000346465.1	NW_006760220.1	+	5870366	5873864	3498	-	-	GO:0003676:nucleic acid binding	-
Pp05_28899-3p(gma-miR1513a-5p)	18780573	PRUPE_ppa010773mg	NCBI_Assembly:GCF_000346465.1	NW_006760220.1	-	174576	177284	2708	pper01100:Metabolic pathways;pper00230:Purine metabolism;pper00240:Pyrimidine metabolism;pper03020:RNA polymerase	GO:0006351:transcription, DNA-templated	GO:0000166:nucleotide binding;GO:0003899:DNA-directed RNA polymerase activity	-
Pp05_28899-3p(gma-miR1513a-5p)	18780579	PRUPE_ppb018954mg	NCBI_Assembly:GCF_000346465.1	NW_006760220.1	-	12155661	12158551	2890	-	GO:0005975:carbohydrate metabolic process	GO:0003824:catalytic activity;GO:0030246:carbohydrate binding	-

Pp05_28899-3p(gma-miR1513a-5p)	18780593	PRUPE_ppa015892mg	NCBI_Assembly:GCF_000346465.1	NW_006760220.1	+	10380346	10382580	2234	-	-	GO:0004674:protein serine/threonine kinase activity;GO:0005524:ATP binding;GO:0030246:carbohydrate binding	-
Pp05_28899-3p(gma-miR1513a-5p)	18781429	PRUPE_ppa001355mg	NCBI_Assembly:GCF_000346465.1	NW_006760220.1	-	5249046	5255164	6118	-	-	GO:0004252:serine-type endopeptidase activity	-
Pp05_28899-3p(gma-miR1513a-5p)	18781431	PRUPE_ppa019980mg	NCBI_Assembly:GCF_000346465.1	NW_006760220.1	+	28087905	28088324	419	-	-	-	-
Pp05_28899-3p(gma-miR1513a-5p)	18781447	PRUPE_ppa016435mg	NCBI_Assembly:GCF_000346465.1	NW_006760220.1	-	21043472	21047254	3782	-	-	-	-
Pp05_28899-3p(gma-miR1513a-5p)	18781530	PRUPE_ppa025063mg	NCBI_Assembly:GCF_000346465.1	NW_006760220.1	-	7818909	7825543	6634	-	-	-	-
Pp05_28899-3p(gma-miR1513a-5p)	18781583	PRUPE_ppa007850mg	NCBI_Assembly:GCF_000346465.1	NW_006760268.1	+	13377712	13378799	1087	pper04626:Plant-pathogen interaction	-	GO:0004672:protein kinase activity;GO:0005524:ATP binding	-
Pp05_28899-3p(gma-miR1513a-5p)	18781592	PRUPE_ppa021100mg	NCBI_Assembly:GCF_000346465.1	NW_006760268.1	-	6276135	6276614	479	-	-	-	-
Pp05_28899-3p(gma-miR1513a-5p)	18781594	PRUPE_ppb018035mg	NCBI_Assembly:GCF_000346465.1	NW_006760268.1	-	6201763	6203325	1562	-	-	-	-

Pp05_28899-3p(gma-miR1513a-5p)	18781622	PRUPE_ppa007179mg	NCBI_Assembly:GCF_000346465.1	NW_006760268.1	+	20829570	20831781	2211	pper03040:Spliceosome	-	GO:0000166:nucleotide binding;GO:0003676:nucleic acid binding	GO:0005829:cytosol
Pp05_28899-3p(gma-miR1513a-5p)	18781636	PRUPE_ppa005102mg	NCBI_Assembly:GCF_000346465.1	NW_006760268.1	-	21130076	21133060	2984	-	-	GO:0016787:hydrolase activity	-
Pp05_28899-3p(gma-miR1513a-5p)	18781637	PRUPE_ppa002402mg	NCBI_Assembly:GCF_000346465.1	NW_006760268.1	-	4802417	4805581	3164	pper03018:RNA degradation	GO:0006457:protein folding	GO:0005524:ATP binding	-
Pp05_28899-3p(gma-miR1513a-5p)	18782821	PRUPE_ppa011307mg	NCBI_Assembly:GCF_000346465.1	NW_006760268.1	-	95677	96874	1197	pper00480:Glutathione metabolism	-	-	-
Pp05_28899-3p(gma-miR1513a-5p)	18782850	PRUPE_ppa025268mg	NCBI_Assembly:GCF_000346465.1	NW_006760268.1	-	989428	993857	4429	-	GO:0006950:response to stress	GO:0004672:protein kinase activity;GO:0005524:ATP binding	-
Pp05_28899-3p(gma-miR1513a-5p)	18782877	PRUPE_ppa006779mg	NCBI_Assembly:GCF_000346465.1	NW_006760268.1	-	14685185	14687061	1876	-	GO:0042545:cell wall modification	GO:0030599:pectinesterase activity;GO:0045330:aspartyl esterase activity	GO:0005618:cell wall
Pp05_28899-3p(gma-miR1513a-5p)	18782886	PRUPE_ppa017150mg	NCBI_Assembly:GCF_000346465.1	NW_006760268.1	-	14401736	14402248	512	-	-	GO:0004672:protein kinase activity;GO:0005524:ATP binding	-
Pp05_28899-3p(gma-miR1513a-5p)	18782942	PRUPE_ppa021437mg	NCBI_Assembly:GCF_000346465.1	NW_006760268.1	+	18455117	18457128	2011	-	-	-	GO:0016021:integral component of membrane

Pp05_28899-3p(gma-miR1513a-5p)	18782962	PRUPE_ppa009083mg	NCBI_Assembly:GCF_000346465.1	NW_006760268.1	+		6450391	6452867	2476	-	GO:0010264:myo-inositol hexakisphosphate biosynthetic process	GO:0015168:glycerol transmembrane transporter activity;GO:0015204:urea transmembrane transporter activity;GO:0046715:borate transmembrane transporter activity	GO:0005886:plasma membrane;GO:0016021:integral component of membrane
Pp05_28899-3p(gma-miR1513a-5p)	18782969	PRUPE_ppa008897mg	NCBI_Assembly:GCF_000346465.1	NW_006760268.1	-		12164075	12166280	2205	-	-	GO:0003824:catalytic activity	-
Pp05_28899-3p(gma-miR1513a-5p)	18782983	PRUPE_ppa014469mg	NCBI_Assembly:GCF_000346465.1	NW_006760268.1	+		21288904	21289681	777	-	-	-	-
Pp05_28899-3p(gma-miR1513a-5p)	18782989	PRUPE_ppa023112mg	NCBI_Assembly:GCF_000346465.1	NW_006760268.1	+		2188072	2190087	2015	pper03010:Ribosome	-	-	-
Pp05_28899-3p(gma-miR1513a-5p)	18783019	PRUPE_ppa007567mg	NCBI_Assembly:GCF_000346465.1	NW_006760268.1	-		20287558	20290017	2459	-	-	GO:0022857:transmembrane transporter activity	GO:0016021:integral component of membrane
Pp05_28899-3p(gma-miR1513a-5p)	18783021	PRUPE_ppa024744mg	NCBI_Assembly:GCF_000346465.1	NW_006760268.1	+		19091119	19092216	1097	-	-	GO:0016758:transferase activity, transferring hexosyl groups	-

Pp05_28899-3p(gma-miR1513a-5p)	18783028	PRUPE_ppa015815mg	NCBI_Assembly:GCF_000346465.1	NW_006760268.1	+	14897888	14898261	373	-	-	GO:0016758:transferase activity, transferring hexosyl groups	-
Pp05_28899-3p(gma-miR1513a-5p)	18783044	PRUPE_ppa008885mg	NCBI_Assembly:GCF_000346465.1	NW_006760268.1	-	12155301	12157278	1977	-	-	GO:0003824:catalytic activity	-
Pp05_28899-3p(gma-miR1513a-5p)	18783057	PRUPE_ppa013890mg	NCBI_Assembly:GCF_000346465.1	NW_006760268.1	-	10445817	10446368	551	-	-	-	-
Pp05_28899-3p(gma-miR1513a-5p)	18783165	PRUPE_ppb022456mg	NCBI_Assembly:GCF_000346465.1	NW_006760268.1	-	7110374	7110595	221	-	-	-	-
Pp05_28899-3p(gma-miR1513a-5p)	18783171	PRUPE_ppa001085mg	NCBI_Assembly:GCF_000346465.1	NW_006760268.1	+	1961559	1965756	4197	pper01100:Metabolic pathways;pper00591:Linoleic acid metabolism;pper00592:alpha-Linolenic acid metabolism	GO:0031408:oxylipin biosynthetic process	GO:0016702:oxidoreductase activity, acting on single donors with incorporation of molecular oxygen, incorporation of two atoms of oxygen;GO:0046872:metal ion binding	-
Pp05_28899-3p(gma-miR1513a-5p)	18783211	PRUPE_ppa010093mg	NCBI_Assembly:GCF_000346465.1	NW_006760268.1	-	14206348	14207814	1466	pper01100:Metabolic pathways;pper00195:Photosynthesis	GO:0015979:photosynthesis	GO:0005509:calcium ion binding	GO:0009654:photosystem II oxygen evolving complex;GO:0019898:extrinsic component of membrane

Pp05_28899-3p(gma-miR1513a-5p)	18783219	PRUPE_ppa001093mg	NCBI_Assembly:GCF_000346465.1	NW_006760268.1	+		16625236	16629452	4216	-	GO:0019722:calcium-mediated signaling;GO:0071230:cellular response to amino acid stimulus	GO:0004970:ionotropic glutamate receptor activity;GO:0005262:calcium channel activity	GO:0005622:intracellular;GO:0005886:plasma membrane;GO:0009506:plasmodesma;GO:0016021:integral component of membrane
Pp05_28899-3p(gma-miR1513a-5p)	18783235	PRUPE_ppa019142mg	NCBI_Assembly:GCF_000346465.1	NW_006760268.1	-		13964671	13967954	3283	-	-	GO:0043531:ADP binding	-
Pp05_28899-3p(gma-miR1513a-5p)	18783347	PRUPE_ppa001435mg	NCBI_Assembly:GCF_000346465.1	NW_006760268.1	+		17314264	17317380	3116	-	GO:0048544:recognition of pollen	GO:0004674:protein serine/threonine kinase activity;GO:0005524:ATP binding	-
Pp05_28899-3p(gma-miR1513a-5p)	18783391	PRUPE_ppa013398mg	NCBI_Assembly:GCF_000346465.1	NW_006760268.1	+		17148798	17149326	528	pper03010:Ribosome	GO:0006412:translation	GO:0003735:structural constituent of ribosome	GO:0005840:ribosome
Pp05_28899-3p(gma-miR1513a-5p)	18783408	PRUPE_ppa010088mg	NCBI_Assembly:GCF_000346465.1	NW_006760268.1	+		20860426	20862309	1883	-	-	GO:0008168:methyltransferase activity	-
Pp05_28899-3p(gma-miR1513a-5p)	18783450	PRUPE_ppa023158mg	NCBI_Assembly:GCF_000346465.1	NW_006760268.1	-		10148936	10150835	1899	-	-	GO:0004672:protein kinase activity;GO:0005524:ATP binding	-
Pp05_28899-3p(gma-miR1513a-5p)	18783453	PRUPE_ppa021752mg	NCBI_Assembly:GCF_000346465.1	NW_006760268.1	+		9177863	9179280	1417	-	-	GO:0003676:nucleic acid binding	-
Pp05_28899-3p(gma-miR1513a-5p)	18783465	PRUPE_ppa005861mg	NCBI_Assembly:GCF_000346465.1	NW_006760268.1	+		5274199	5278301	4102	-	-	-	-

Pp05_28899-3p(gma-miR1513a-5p)	18783468	PRUPE_ppa002875mg	NCBI_Assembly:GCF_000346465.1	NW_006760268.1	-	950340	956010	5670	-	GO:0015992: proton transport	GO:0004427: inorganic diphosphatase activity;GO:0009678:hydrogen-translocating pyrophosphatase activity	GO:0016020: membrane

Pp05_28899-3p(gma-miR1513a-5p)	18783470	PRUPE_ppa027152mg	NCBI_Assembly:GCF_000346465.1	NW_006760268.1	-	4228382	4239915	11533	-	GO:0006346:methylation-dependent chromatin silencing;GO:0007267:cell-cell signaling;GO:0009616:virus induced gene silencing;GO:0009855:determination of bilateral symmetry;GO:0010014:meristem initiation;GO:0010073:meristem maintenance;GO:0010267:production of ta-siRNAs involved in RNA interference;GO:0035196:production of miRNAs involved in gene silencing by miRNA	GO:0003677:DNA binding;GO:0004003:ATP-dependent DNA helicase activity;GO:0005524:ATP binding	GO:0005634:nucleus;GO:0005739:mitochondrion	
Pp05_28899-3p(gma-miR1513a-5p)	18783525	PRUPE_ppa024318mg	NCBI_Assembly:GCF_000346465.1	NW_006760268.1	-	19878339	19880404	2065	-	GO:0006351:transcription, DNA-templated	GO:0003677:DNA binding;GO:0003700:sequence-specific DNA binding transcription factor activity	GO:0005634:nucleus	

Pp05_28899-3p(gma-miR1513a-5p)	18783529	PRUPE_ppa019116mg	NCBI_Assembly:GCF_000346465.1	NW_006760268.1	+	19105243	19106720	1477	-	-	GO:0016758:transferase activity, transferring hexosyl groups	-
Pp05_28899-3p(gma-miR1513a-5p)	18783562	PRUPE_ppa026882mg	NCBI_Assembly:GCF_000346465.1	NW_006760268.1	+	10572145	10574377	2232	-	-	GO:0003700:sequence-specific DNA binding transcription factor activity;GO:0043565:sequence-specific DNA binding	GO:0016602:CCAAT-binding factor complex
Pp05_28899-3p(gma-miR1513a-5p)	18783602	PRUPE_ppa023194mg	NCBI_Assembly:GCF_000346465.1	NW_006760268.1	+	3267227	3268611	1384	-	-	GO:0004674:protein serine/threonine kinase activity;GO:0005524:ATP binding	-
Pp05_28899-3p(gma-miR1513a-5p)	18783622	PRUPE_ppa025639mg	NCBI_Assembly:GCF_000346465.1	NW_006760268.1	-	1923380	1924917	1537	pper01100:Metabolic pathways;pper04145:Phagosome;pper00190:Oxidative phosphorylation	GO:0015992:proton transport	GO:0016820:hydrolase activity, acting on acid anhydrides, catalyzing transmembrane movement of substances	GO:0016471:vacuolar proton-transporting V-type ATPase complex
Pp05_28899-3p(gma-miR1513a-5p)	18783675	PRUPE_ppa012959mg	NCBI_Assembly:GCF_000346465.1	NW_006760268.1	-	13108381	13109835	1454	pper00480:Glutathione metabolism	GO:0006888:ER to Golgi vesicle-mediated transport	-	GO:0005773:vacuole;GO:0005783:endoplasmic reticulum;GO:0005794:Golgi apparatus;GO:0016020:membrane

Pp05_28899-3p(gma-miR1513a-5p)	18783689	PRUPE_ppa016346mg	NCBI_Assembly:GCF_000346465.1	NW_006760268.1	-		10291657	10292508	851	-	-	GO:0030247:polysaccharide binding	-
Pp05_28899-3p(gma-miR1513a-5p)	18783754	PRUPE_ppa009608mg	NCBI_Assembly:GCF_000346465.1	NW_006760268.1	-		13672078	13673293	1215	-	GO:0006073:cellular glucan metabolic process;GO:0071555:cell wall organization	GO:0004553:hydrolase activity, hydrolyzing O-glycosyl compounds; GO:0016762:xyloglucan:xyloglucosyl transferase activity	GO:0005618:cell wall;GO:0048046:apoplast
Pp05_28899-3p(gma-miR1513a-5p)	18783763	PRUPE_ppa002316mg	NCBI_Assembly:GCF_000346465.1	NW_006760268.1	-		20664157	20666223	2066	-	-	-	-
Pp05_28899-3p(gma-miR1513a-5p)	18783797	PRUPE_ppa022761mg	NCBI_Assembly:GCF_000346465.1	NW_006760268.1	+		16392720	16394208	1488	-	-	-	-
Pp05_28899-3p(gma-miR1513a-5p)	18783819	PRUPE_ppb023624mg	NCBI_Assembly:GCF_000346465.1	NW_006760268.1	-		9051331	9051899	568	-	-	-	-
Pp05_28899-3p(gma-miR1513a-5p)	18783846	PRUPE_ppa015099mg	NCBI_Assembly:GCF_000346465.1	NW_006760268.1	+		2457881	2460314	2433	-	-	-	-
Pp05_28899-3p(gma-miR1513a-5p)	18783847	PRUPE_ppa024730mg	NCBI_Assembly:GCF_000346465.1	NW_006760268.1	+		17555911	17556828	917	pper04130:SNARE interactions in vesicular transport	GO:0006886:intracellular protein transport;GO:0016192:vesicle-mediated transport;GO:0061025:membrane fusion	-	GO:0005622:intracellular; GO:0016020:membrane

Pp05_28899-3p(gma-miR1513a-5p)	18783911	PRUPE_ppa023196mg	NCBI_Assembly:GCF_000346465.1	NW_006760268.1	-	15133458	15136920	3462	-	GO:0010228:vegetative to reproductive phase transition of meristem	-	-	
Pp05_28899-3p(gma-miR1513a-5p)	18783918	PRUPE_ppa026005mg	NCBI_Assembly:GCF_000346465.1	NW_006760268.1	-	3393089	3393343	254	-	-	-	-	
Pp05_28899-3p(gma-miR1513a-5p)	18783945	PRUPE_ppa002868mg	NCBI_Assembly:GCF_000346465.1	NW_006760268.1	+	1884605	1886813	2208	-	GO:0006887:exocytosis	-	GO:0000145:exocyst	
Pp05_28899-3p(gma-miR1513a-5p)	18784097	PRUPE_ppa008810mg	NCBI_Assembly:GCF_000346465.1	NW_006760268.1	-	13745513	13748865	3352	-	GO:0006783:heme biosynthetic process;GO:0009116:nucleoside metabolic process;GO:0009165:nucleotide biosynthetic process;GO:0009220:pyrimidine ribonucleotide biosynthetic process	GO:0000287:magnesium ion binding;GO:0004749:ribose phosphate diphosphokinase activity	GO:0005829:cytosol;GO:0005886:plasma membrane;GO:0009506:plasmodesma	
Pp05_28899-3p(gma-miR1513a-5p)	18784111	PRUPE_ppa026312mg	NCBI_Assembly:GCF_000346465.1	NW_006760268.1	+	5114422	5116112	1690	-	-	-	-	
Pp05_28899-3p(gma-miR1513a-5p)	18784178	PRUPE_ppa015754mg	NCBI_Assembly:GCF_000346465.1	NW_006760268.1	+	93365	93879	514	-	-	-	-	

Pp05_28899-3p(gma-miR1513a-5p)	18784192	PRUPE_ppa007697mg	NCBI_Assembly:GCF_000346465.1	NW_006760268.1	-	3337491	3341328	3837	-	-	GO:0003677:DNA binding;GO:0003682:chromatin binding;GO:0003700:sequence-specific DNA binding transcription factor activity;GO:0008270:zinc ion binding	-
Pp05_28899-3p(gma-miR1513a-5p)	18784194	PRUPE_ppa017921mg	NCBI_Assembly:GCF_000346465.1	NW_006760268.1	+	21079579	21081198	1619	-	-	-	-
Pp05_28899-3p(gma-miR1513a-5p)	18784215	PRUPE_ppa003100mg	NCBI_Assembly:GCF_000346465.1	NW_006760268.1	-	20042210	20044109	1899	-	-	GO:0004672:protein kinase activity;GO:0005524:ATP binding	GO:0016021:integral component of membrane
Pp05_28899-3p(gma-miR1513a-5p)	18784252	PRUPE_ppa019320mg	NCBI_Assembly:GCF_000346465.1	NW_006760268.1	+	18998050	18999448	1398	-	-	GO:0016747:transferase activity, transferring acyl groups other than amino-acyl groups	-
Pp05_28899-3p(gma-miR1513a-5p)	18784257	PRUPE_ppa023888mg	NCBI_Assembly:GCF_000346465.1	NW_006760268.1	+	425150	426616	1466	-	-	GO:0000166:nucleotide binding;GO:0003676:nucleic acid binding	-
Pp05_28899-3p(gma-miR1513a-5p)	18784315	PRUPE_ppa009467mg	NCBI_Assembly:GCF_000346465.1	NW_006760268.1	-	5328276	5330492	2216	-	-	GO:0004252:serine-type endopeptidase activity	GO:0016021:integral component of membrane

Pp05_28899-3p(gma-miR1513a-5p)	18784324	PRUPE_ppa012096mg	NCBI_Assembly:GCF_000346465.1	NW_006760268.1	-	12654996	12656260	1264	-	GO:0006334:nucleosome assembly;GO:0006355:regulation of transcription, DNA-templated	GO:0003677:DNA binding	GO:0000786:nucleosome;GO:0005634:nucleus
Pp05_28899-3p(gma-miR1513a-5p)	18784435	PRUPE_ppa005206mg	NCBI_Assembly:GCF_000346465.1	NW_006760268.1	-	16246893	16248699	1806	-	GO:0005975:carbohydrate metabolic process;GO:0007389:pattern specification process;GO:0008356:asymmetric cell division;GO:0008361:regulation of cell size;GO:0009926:auxin polar transport;GO:0010015:root morphogenesis;GO:0010075:regulation of meristem growth;GO:0040007:growth	GO:0004553:hydrolase activity, hydrolyzing O-glycosyl compounds	-
Pp05_28899-3p(gma-miR1513a-5p)	18784516	PRUPE_ppa015089m1g	NCBI_Assembly:GCF_000346465.1	NW_006760268.1	+	10081871	10082515	644	-	-	GO:0008375:acetylglucosaminyltransferase activity	GO:0016020:membrane

Pp05_28899-3p(gma-miR1513a-5p)	18784527	PRUPE_ppa012875mg	NCBI_Assembly:GCF_000346465.1	NW_006760281.1	-	8132	8642	510	-	-	GO:0016788:hydrolase activity, acting on ester bonds	-
Pp05_28899-3p(gma-miR1513a-5p)	18784573	PRUPE_ppa014906mg	NCBI_Assembly:GCF_000346465.1	NW_006760324.1	+	22403101	22403934	833	-	-	GO:0003700:sequence-specific DNA binding transcription factor activity;GO:0046872:metal ion binding	-
Pp05_28899-3p(gma-miR1513a-5p)	18784713	PRUPE_ppa011247mg	NCBI_Assembly:GCF_000346465.1	NW_006760324.1	+	24705774	24707664	1890	-	-	-	-
Pp05_28899-3p(gma-miR1513a-5p)	18784730	PRUPE_ppa001259mg	NCBI_Assembly:GCF_000346465.1	NW_006760324.1	-	25956077	25960666	4589	pper03013:RNA transport	GO:0006606:protein import into nucleus;GO:0052541:plant-type cell wall cellulose metabolic process;GO:0052546:cell wall pectin metabolic process	-	GO:0005634:nucleus;GO:0009507:chloroplast

Pp05_28899-3p(gma-miR1513a-5p)	18784775	PRUPE_ppa006573mg	NCBI_Assembly:GCF_000346465.1	NW_006760324.1	-	15668344	15673521	5177	pper01100:Metabolic pathways;pper01200:Carbon metabolism;pper00280:Valine, leucine and isoleucine degradation;pper00410:beta-Alanine metabolism;pper00640:Propanoate metabolism	-	GO:0003824:catalytic activity	-
Pp05_28899-3p(gma-miR1513a-5p)	18784788	PRUPE_ppa012782mg	NCBI_Assembly:GCF_000346465.1	NW_006760324.1	-	22458824	22461522	2698	-	-	-	GO:0005886:plasma membrane;GO:0016021:integral component of membrane
Pp05_28899-3p(gma-miR1513a-5p)	18784806	PRUPE_ppa000178mg	NCBI_Assembly:GCF_000346465.1	NW_006760324.1	-	20485707	20494195	8488	-	GO:0006306:DNA methylation;GO:0030422:production of siRNA involved in RNA interference	-	-
Pp05_28899-3p(gma-miR1513a-5p)	18785006	PRUPE_ppa021699mg	NCBI_Assembly:GCF_000346465.1	NW_006760324.1	+	15087711	15088190	479	-	-	-	-
Pp05_28899-3p(gma-miR1513a-5p)	18785029	PRUPE_ppa021062mg	NCBI_Assembly:GCF_000346465.1	NW_006760324.1	+	7506051	7510656	4605	-	GO:0007165:signal transduction	GO:0005524:ATP binding;GO:0043531:ADP binding	-

Pp05_28899-3p(gma-miR1513a-5p)	18785030	PRUPE_ppa021654mg	NCBI_Assembly:GCF_000346465.1	NW_006760324.1	-	2365867	2368299	2432	-	-	GO:0016491: oxidoreductase activity	-
Pp05_28899-3p(gma-miR1513a-5p)	18785036	PRUPE_ppa017993mg	NCBI_Assembly:GCF_000346465.1	NW_006760324.1	+	17841005	17842558	1553	-	GO:0006396: RNA processing	GO:0003723: RNA binding;GO:0004000:adenosine deaminase activity	-
Pp05_28899-3p(gma-miR1513a-5p)	18785062	PRUPE_ppa013803mg	NCBI_Assembly:GCF_000346465.1	NW_006760324.1	+	19856674	19857132	458	-	-	-	-
Pp05_28899-3p(gma-miR1513a-5p)	18785119	PRUPE_ppa013005mg	NCBI_Assembly:GCF_000346465.1	NW_006760324.1	+	26302324	26303913	1589	-	-	-	-
Pp05_28899-3p(gma-miR1513a-5p)	18785121	PRUPE_ppa010341mg	NCBI_Assembly:GCF_000346465.1	NW_006760324.1	+	22461817	22463517	1700	-	-	-	GO:0005759: mitochondrial matrix
Pp05_28899-3p(gma-miR1513a-5p)	18785161	PRUPE_ppa005481mg	NCBI_Assembly:GCF_000346465.1	NW_006760324.1	+	26486647	26491841	5194	-	GO:0000082: G1/S transition of mitotic cell cycle;GO:0006351:transcription, DNA-templated;GO:0010090:trichome morphogenesis;GO:0042023:DNA endoreduplication;GO:0051302:regulation of cell division	GO:0003677: DNA binding;GO:0003700:sequence-specific DNA binding transcription factor activity	GO:0005634: nucleus;GO:0005667:transcription factor complex

Pp05_28899-3p(gma-miR1513a-5p)	18785192	PRUPE_ppa009021mg	NCBI_Assembly:GCF_000346465.1	NW_006760324.1	+	17133123	17135222	2099	pper04145:Phagosome;pper04130:SNARE interactions in vesicular transport	GO:0000226:microtubule cytoskeleton organization;GO:0016192:vesicle-mediated transport	-	GO:0016020:membrane
Pp05_28899-3p(gma-miR1513a-5p)	18785199	PRUPE_ppa024723mg	NCBI_Assembly:GCF_000346465.1	NW_006760324.1	+	25878109	25880378	2269	-	-	GO:0008270:zinc ion binding	-
Pp05_28899-3p(gma-miR1513a-5p)	18785227	PRUPE_ppa016971mg	NCBI_Assembly:GCF_000346465.1	NW_006760324.1	+	2483001	2484603	1602	-	-	GO:0003924:GTPase activity;GO:0005525:GTP binding	-

Pp05_28899-3p(gma-miR1513a-5p)	18785240	PRUPE_ppa003263mg	NCBI_Assembly:GCF_000346465.1	NW_006760324.1	+	23569948	23574009	4061	-	GO:0000278:mitotic cell cycle;GO:0000724:double-strand break repair via homologous recombination;GO:0006270:DNA replication initiation;GO:0006275:regulation of DNA replication;GO:0006301:postreplication repair;GO:0006306:DNA methylation;GO:0007062:sister chromatid cohesion;GO:0008283:cell proliferation;GO:0009555:pollen development;GO:0010090:trichome morphogenesis;GO:0016444:somatic cell DNA recombination;GO:003104	-	GO:0009941:chloroplast envelope;GO:0042555:MCM complex;GO:0043601:nuclear replisome
Pp05_28899-3p(gma-miR1513a-5p)	18785242	PRUPE_ppa007790mg	NCBI_Assembly:GCF_000346465.1	NW_006760324.1	-	18995535	18999459	3924	-	GO:0055085:transmembrane transport	-	GO:0016021:integral component of membrane

Pp05_28899-3p(gma-miR1513a-5p)	18785295	PRUPE_ppa011869mg	NCBI_Assembly:GCF_000346465.1	NW_006760324.1	-	22679065	22682219	3154	-	-	GO:0008270:zinc ion binding	-
Pp05_28899-3p(gma-miR1513a-5p)	18785362	PRUPE_ppa024973mg	NCBI_Assembly:GCF_000346465.1	NW_006760324.1	-	6914703	6915095	392	-	-	-	-
Pp05_28899-3p(gma-miR1513a-5p)	18785450	PRUPE_ppa021059mg	NCBI_Assembly:GCF_000346465.1	NW_006760324.1	+	25740968	25743248	2280	-	GO:0000278:mitotic cell cycle;GO:0000724:double-strand break repair via homologous recombination;GO:0006261:DNA-dependent DNA replication;GO:0006275:regulation of DNA replication;GO:0006306:DNA methylation; GO:0006342:chromatin silencing;GO:0007267:cell-cell signaling;GO:0009555:pollen development;GO:0009616:virus induced gene silencing;GO:0010267:production of ta-siRNAs involved in RNA	-	-

Pp05_28899-3p(gma-miR1513a-5p)	18785458	PRUPE_ppa020366mg	NCBI_Assembly:GCF_000346465.1	NW_006760324.1	+	15063619	15066323	2704	-	GO:0006457:protein folding	GO:0003755:peptidyl-prolyl cis-trans isomerase activity	-
Pp05_28899-3p(gma-miR1513a-5p)	18785462	PRUPE_ppa004765mg	NCBI_Assembly:GCF_000346465.1	NW_006760324.1	-	17117352	17118994	1642	-	GO:0009793:embryo development ending in seed dormancy	-	-
Pp05_28899-3p(gma-miR1513a-5p)	18785490	PRUPE_ppa006818mg	NCBI_Assembly:GCF_000346465.1	NW_006760324.1	-	21759049	21763180	4131	-	GO:0010042:response to manganese ion;GO:0010264:myo-inositol hexakisphosphate biosynthetic process;GO:0030026:cellular manganese ion homeostasis;GO:0046688:response to copper ion	GO:0010486:manganese:proton antiporter activity	GO:0005770:late endosome;GO:0016021:integral component of membrane
Pp05_28899-3p(gma-miR1513a-5p)	18785530	PRUPE_ppa022724mg	NCBI_Assembly:GCF_000346465.1	NW_006760324.1	-	5114242	5114813	571	-	-	GO:0043531:ADP binding	-

Pp05_28899-3p(gma-miR1513a-5p)	18785572	PRUPE_ppa002129mg	NCBI_Assembly:GCF_000346465.1	NW_006760324.1	+		25154198	25158694	4496	-	GO:0006950:response to stress	GO:0004672:protein kinase activity;GO:0004842:ubiquitin-protein transferase activity;GO:0005524:ATP binding;GO:0016874:ligase activity	GO:0005886:plasma membrane
Pp05_28899-3p(gma-miR1513a-5p)	18785688	PRUPE_ppa010939mg	NCBI_Assembly:GCF_000346465.1	NW_006760324.1	+		20212785	20214825	2040	-	-	-	-
Pp05_28899-3p(gma-miR1513a-5p)	18785726	PRUPE_ppa014769mg	NCBI_Assembly:GCF_000346465.1	NW_006760324.1	-		21726931	21729663	2732	-	-	-	GO:0048046:apoplast
Pp05_28899-3p(gma-miR1513a-5p)	18785757	PRUPE_ppa009290mg	NCBI_Assembly:GCF_000346465.1	NW_006760324.1	+		17693304	17695477	2173	pper01110:Biosynthesis of secondary metabolites;pper00902:Monoterpenoid biosynthesis	-	GO:0016491:oxidoreductase activity	-
Pp05_28899-3p(gma-miR1513a-5p)	18785767	PRUPE_ppa005258mg	NCBI_Assembly:GCF_000346465.1	NW_006760324.1	+		16489029	16495046	6017	-	GO:0009790:embryo development;GO:0016556:mRNA modification;GO:0070526:threonylcarbamoyladenosine biosynthetic process	GO:0004222:metalloendopeptidase activity;GO:0016747:transferase activity, transferring acyl groups other than amino-acyl groups;GO:0046872:metal ion binding	GO:0005743:mitochondrial inner membrane

Pp05_28899-3p(gma-miR1513a-5p)	18785770	PRUPE_ppa013989mg	NCBI_Assembly:GCF_000346465.1	NW_006760324.1	+	14930273	14932313	2040	pper03010:Ribosome	GO:0006412:translation	GO:0003735:structural constituent of ribosome	GO:0022625:cytosolic large ribosomal subunit
Pp05_28899-3p(gma-miR1513a-5p)	18785776	PRUPE_ppa001456mg	NCBI_Assembly:GCF_000346465.1	NW_006760324.1	-	19166957	19171308	4351	-	-	GO:0003676:nucleic acid binding;GO:0004386:helicase activity;GO:0005524:ATP binding	-
Pp05_28899-3p(gma-miR1513a-5p)	18785781	PRUPE_ppa024626mg	NCBI_Assembly:GCF_000346465.1	NW_006760324.1	-	15752405	15757245	4840	-	GO:0007165:signal transduction	GO:0043531:ADP binding	-
Pp05_28899-3p(gma-miR1513a-5p)	18785823	PRUPE_ppa005753mg	NCBI_Assembly:GCF_000346465.1	NW_006760324.1	-	23383585	23384937	1352	-	-	-	-
Pp05_28899-3p(gma-miR1513a-5p)	18785832	PRUPE_ppa010370mg	NCBI_Assembly:GCF_000346465.1	NW_006760324.1	-	9571470	9572728	1258	-	GO:0015979:photosynthesis	GO:0005509:calcium ion binding	GO:0009654:photosystem II oxygen evolving complex;GO:0019898:extrinsic component of membrane
Pp05_28899-3p(gma-miR1513a-5p)	18785850	PRUPE_ppa000242mg	NCBI_Assembly:GCF_000346465.1	NW_006760324.1	+	15870084	15877153	7069	-	GO:0048208:COPII vesicle coating	-	GO:0000139:Golgi membrane
Pp05_28899-3p(gma-miR1513a-5p)	18785866	PRUPE_ppa019942mg	NCBI_Assembly:GCF_000346465.1	NW_006760324.1	+	732093	733181	1088	-	-	GO:0047196:long-chain-alcohol O-fatty-acyltransferase activity	-

Pp05_28899-3p(gma-miR1513a-5p)	18785878	PRUPE_ppa004511mg	NCBI_Assembly:GCF_000346465.1	NW_006760324.1	+		246558	253715	7157	pper00330:Arginine and proline metabolism;pper00410:beta-Alanine metabolism	GO:0006598:polyamine catabolic process	GO:0046592:polyamine oxidase activity;GO:0050660:flavin adenine dinucleotide binding	-
Pp05_28899-3p(gma-miR1513a-5p)	18785884	PRUPE_ppa019263mg	NCBI_Assembly:GCF_000346465.1	NW_006760324.1	-		653638	654511	873	-	-	GO:0022891:substrate-specific transmembrane transporter activity	GO:0016021:integral component of membrane
Pp05_28899-3p(gma-miR1513a-5p)	18785971	PRUPE_ppa026150mg	NCBI_Assembly:GCF_000346465.1	NW_006760324.1	+		13247309	13249427	2118	-	-	GO:0003677:DNA binding	-
Pp05_28899-3p(gma-miR1513a-5p)	18786016	PRUPE_ppa020952mg	NCBI_Assembly:GCF_000346465.1	NW_006760324.1	+		13319685	13321653	1968	-	GO:0006351:transcription, DNA-templated;GO:0006355:regulation of transcription, DNA-templated	GO:0003677:DNA binding	GO:0005634:nucleus
Pp05_28899-3p(gma-miR1513a-5p)	18786037	PRUPE_ppa023670mg	NCBI_Assembly:GCF_000346465.1	NW_006760324.1	+		3754018	3754542	524	-	GO:0006351:transcription, DNA-templated;GO:0006355:regulation of transcription, DNA-templated	GO:0003677:DNA binding	GO:0005634:nucleus
Pp05_28899-3p(gma-miR1513a-5p)	18786044	PRUPE_ppa019355mg	NCBI_Assembly:GCF_000346465.1	NW_006760324.1	+		21875835	21878210	2375	-	-	GO:0004674:protein serine/threonine kinase activity;GO:0005524:ATP binding	-

Pp05_28899-3p(gma-miR1513a-5p)	18786082	PRUPE_ppa024377mg	NCBI_Assembly:GCF_000346465.1	NW_006760324.1	-		1157298	1161347	4049	-	-	GO:0043531:ADP binding	-
Pp05_28899-3p(gma-miR1513a-5p)	18786091	PRUPE_ppb016037mg	NCBI_Assembly:GCF_000346465.1	NW_006760324.1	+		11034555	11035025	470	-	-	-	-
Pp05_28899-3p(gma-miR1513a-5p)	18786093	PRUPE_ppa008683mg	NCBI_Assembly:GCF_000346465.1	NW_006760324.1	-		24005476	24009151	3675	-	-	-	-
Pp05_28899-3p(gma-miR1513a-5p)	18786095	PRUPE_ppa023229mg	NCBI_Assembly:GCF_000346465.1	NW_006760324.1	-		24086021	24088165	2144	-	-	GO:0022891:substrate-specific transmembrane transporter activity	GO:0016021:integral component of membrane
Pp05_28899-3p(gma-miR1513a-5p)	18786148	PRUPE_ppa005062mg	NCBI_Assembly:GCF_000346465.1	NW_006760324.1	+		25461516	25463276	1760	-	GO:0006873:cellular ion homeostasis	GO:0008308:voltage-gated anion channel activity	GO:0005623:cell;GO:0016021:integral component of membrane
Pp05_28899-3p(gma-miR1513a-5p)	18786169	PRUPE_ppa008326m2g	NCBI_Assembly:GCF_000346465.1	NW_006760324.1	+		19046071	19046418	347	-	-	GO:0016758:transferase activity, transferring hexosyl groups	-
Pp05_28899-3p(gma-miR1513a-5p)	18786214	PRUPE_ppa000999mg	NCBI_Assembly:GCF_000346465.1	NW_006760324.1	-		18032008	18036356	4348	-	-	-	-
Pp05_28899-3p(gma-miR1513a-5p)	18786216	PRUPE_ppa000332mg	NCBI_Assembly:GCF_000346465.1	NW_006760324.1	-		23281641	23287317	5676	pper03440:Homologous recombination	-	GO:0003677:DNA binding;GO:0005524:ATP binding	-
Pp05_28899-3p(gma-miR1513a-5p)	18786260	PRUPE_ppa018314mg	NCBI_Assembly:GCF_000346465.1	NW_006760324.1	+		16755137	16756075	938	-	-	-	-

Pp05_28899-3p(gma-miR1513a-5p)	18786304	PRUPE_ppa000274mg	NCBI_Assembly:GCF_000346465.1	NW_006760324.1	-	472157	477921	5764	-	-	GO:0043531:ADP binding	-
Pp05_28899-3p(gma-miR1513a-5p)	18786305	PRUPE_ppa019347mg	NCBI_Assembly:GCF_000346465.1	NW_006760324.1	+	21889037	21892290	3253	-	-	GO:0000166:nucleotide binding;GO:0003676:nucleic acid binding;GO:0008270:zinc ion binding	-
Pp05_28899-3p(gma-miR1513a-5p)	18786310	PRUPE_ppa009027mg	NCBI_Assembly:GCF_000346465.1	NW_006760324.1	+	6008190	6012659	4469	pper01100:Metabolic pathways;pper00052:Galactose metabolism;pper00561:Glycerolipid metabolism;pper00040:Pentose and glucuronate interconversions;pper00051:Fructose and mannose metabolism	-	GO:0016491:oxidoreductase activity	-
Pp05_28899-3p(gma-miR1513a-5p)	18786570	PRUPE_ppa013895mg	NCBI_Assembly:GCF_000346465.1	NW_006760324.1	-	20846827	20847778	951	-	-	-	-
Pp05_28899-3p(gma-miR1513a-5p)	18786600	PRUPE_ppa023228mg	NCBI_Assembly:GCF_000346465.1	NW_006760324.1	+	11796687	11799294	2607	-	-	-	-
Pp05_28899-3p(gma-miR1513a-5p)	18786642	PRUPE_ppa011740mg	NCBI_Assembly:GCF_000346465.1	NW_006760324.1	-	2361009	2364643	3634	-	-	GO:0016491:oxidoreductase activity	-

Pp05_28899-3p(gma-miR1513a-5p)	18786689	PRUPE_ppa015867mg	NCBI_Assembly:GCF_000346465.1	NW_006760324.1	-	734005	736127	2122	-	-	-	-
Pp05_28899-3p(gma-miR1513a-5p)	18786742	PRUPE_ppa1027171mg	NCBI_Assembly:GCF_000346465.1	NW_006760324.1	-	24187075	24189120	2045	-	-	GO:0016491:oxidoreductase activity	-
Pp05_28899-3p(gma-miR1513a-5p)	18786808	PRUPE_ppa001538mg	NCBI_Assembly:GCF_000346465.1	NW_006760324.1	-	22947996	22950548	2552	-	GO:0006612:protein targeting to membrane;GO:0009723:response to ethylene;GO:0009738:abscisic acid-activated signaling pathway;GO:0010363:regulation of plant-type hypersensitive response;GO:0035556:intracellular signal transduction;GO:0043069:negative regulation of programmed cell death	GO:0004674:protein serine/threonine kinase activity;GO:0005524:ATP binding	GO:0005622:intracellular
Pp05_28899-3p(gma-miR1513a-5p)	18786809	PRUPE_ppa021965mg	NCBI_Assembly:GCF_000346465.1	NW_006760324.1	+	15396321	15396650	329	-	-	-	-
Pp05_28899-3p(gma-miR1513a-5p)	18786824	PRUPE_ppa013600mg	NCBI_Assembly:GCF_000346465.1	NW_006760324.1	-	24666908	24667855	947	-	-	-	-

Pp05_28899-3p(gma-miR1513a-5p)	18786865	PRUPE_ppa000528m1g	NCBI_Assembly:GCF_000346465.1	NW_006760324.1	+	26413550	26418447	4897	-	GO:0006355: regulation of transcription, DNA-templated	-	-
Pp05_28899-3p(gma-miR1513a-5p)	18786866	PRUPE_ppb013494mg	NCBI_Assembly:GCF_000346465.1	NW_006760324.1	+	2872250	2872666	416	-	-	GO:0046872: metal ion binding	-
Pp05_28899-3p(gma-miR1513a-5p)	18786892	PRUPE_ppb015558mg	NCBI_Assembly:GCF_000346465.1	NW_006760324.1	-	17623600	17625591	1991	-	-	-	-
Pp05_28899-3p(gma-miR1513a-5p)	18786894	PRUPE_ppa000482mg	NCBI_Assembly:GCF_000346465.1	NW_006760324.1	-	22231845	22237177	5332	-	GO:0006886: intracellular protein transport;GO:0006896:Golgi to vacuole transport;GO:0051453:regulation of intracellular pH;GO:0080171:lytic vacuole organization	-	GO:0030123: AP-3 adaptor complex
Pp05_28899-3p(gma-miR1513a-5p)	18786942	PRUPE_ppa011950mg	NCBI_Assembly:GCF_000346465.1	NW_006760324.1	-	20786045	20787551	1506	-	-	GO:0008270: zinc ion binding	-
Pp05_28899-3p(gma-miR1513a-5p)	18786953	PRUPE_ppa026509mg	NCBI_Assembly:GCF_000346465.1	NW_006760324.1	+	19057834	19059326	1492	-	GO:0006694: steroid biosynthetic process	GO:0003854: 3-beta-hydroxy-delta5-steroid dehydrogenase activity	-
Pp05_28899-3p(gma-miR1513a-5p)	18787000	PRUPE_ppa017011mg	NCBI_Assembly:GCF_000346465.1	NW_006760324.1	+	26492599	26497211	4612	-	-	-	-

Pp05_28899-3p(gma-miR1513a-5p)	18787047	PRUPE_ppa007992mg	NCBI_Assembly:GCF_000346465.1	NW_006760324.1	+	23361507	23363617	2110	-	-	-	-
Pp05_28899-3p(gma-miR1513a-5p)	18787056	PRUPE_ppa018124mg	NCBI_Assembly:GCF_000346465.1	NW_006760324.1	-	20219526	20221329	1803	-	GO:0006351:transcription, DNA-templated;GO:0006355:regulation of transcription, DNA-templated	GO:0003677:DNA binding	GO:0005634:nucleus
Pp05_28899-3p(gma-miR1513a-5p)	18787060	PRUPE_ppa010887mg	NCBI_Assembly:GCF_000346465.1	NW_006760324.1	+	24727554	24729143	1589	-	-	GO:0005506:iron ion binding;GO:0016706:oxidoreductase activity, acting on paired donors, with incorporation or reduction of molecular oxygen, 2-oxoglutarate as one donor, and incorporation of one atom each of oxygen into both donors;GO:0031418:L-ascorbic acid binding	-
Pp05_28899-3p(gma-miR1513a-5p)	18787085	PRUPE_ppa010334mg	NCBI_Assembly:GCF_000346465.1	NW_006760324.1	+	14469262	14470795	1533	-	-	GO:0016787:hydrolase activity	-

Pp05_28899-3p(gma-miR1513a-5p)	18787087	PRUPE_ppa001436mg	NCBI_Assembly:GCF_000346465.1	NW_006760324.1	+	17450801	17456118	5317	-	GO:0006355:regulation of transcription, DNA-templated	GO:0008289:lipid binding;GO:0043565:sequence-specific DNA binding	GO:0005634:nucleus	
Pp05_28899-3p(gma-miR1513a-5p)	18787097	PRUPE_ppb014638mg	NCBI_Assembly:GCF_000346465.1	NW_006760324.1	+	11170042	11170512	470	-	-	-	-	
Pp05_28899-3p(gma-miR1513a-5p)	18787123	PRUPE_ppa016405mg	NCBI_Assembly:GCF_000346465.1	NW_006760324.1	-	20810567	20810791	224	-	-	-	-	
Pp05_28899-3p(gma-miR1513a-5p)	18787134	PRUPE_ppa013899mg	NCBI_Assembly:GCF_000346465.1	NW_006760324.1	-	25268312	25268969	657	-	-	-	-	
Pp05_28899-3p(gma-miR1513a-5p)	18787138	PRUPE_ppa019341mg	NCBI_Assembly:GCF_000346465.1	NW_006760324.1	+	5597128	5601258	4130	-	GO:0007165:signal transduction	GO:0043531:ADP binding	-	
Pp05_28899-3p(gma-miR1513a-5p)	18787148	PRUPE_ppa007614mg	NCBI_Assembly:GCF_000346465.1	NW_006760324.1	+	25392571	25395627	3056	pper04626:Plant-pathogen interaction	-	-	-	
Pp05_28899-3p(gma-miR1513a-5p)	18787179	PRUPE_ppa018100mg	NCBI_Assembly:GCF_000346465.1	NW_006760324.1	-	25716113	25718476	2363	-	-	-	-	

Pp05_28899-3p(gma-miR1513a-5p)	18787196	PRUPE_ppa009051mg	NCBI_Assembly:GCF_000346465.1	NW_006760324.1	-	17047525	17050233	2708	-	GO:0000373:Group II intron splicing;GO:0000956:nuclear-transcribed mRNA catabolic process;GO:0009793:embryo development ending in seed dormancy;GO:0010048:vernalization response;GO:0048573:photoperiodism, flowering;GO:0097031:mitochondrial respiratory chain complex I biogenesis;GO:0097034:mitochondrial respiratory chain complex IV biogenesis	GO:0003723:RNA binding	-
Pp05_28899-3p(gma-miR1513a-5p)	18787272	PRUPE_ppa021756mg	NCBI_Assembly:GCF_000346465.1	NW_006760324.1	+	21528639	21533914	5275	-	-	GO:0004672:protein kinase activity;GO:0005216:ion channel activity;GO:0005524:ATP binding	GO:0016021:integral component of membrane

Pp05_28899-3p(gma-miR1513a-5p)	18787311	PRUPE_ppa004373mg	NCBI_Assembly:GCF_000346465.1	NW_006760324.1	-	10893438	10894989	1551	-	-	GO:0004497:monooxygenase activity;GO:0005506:iron ion binding;GO:0016705:oxidoreductase activity, acting on paired donors, with incorporation or reduction of molecular oxygen;GO:0020037:heme binding	-
Pp05_28899-3p(gma-miR1513a-5p)	18787312	PRUPE_ppa020445mg	NCBI_Assembly:GCF_000346465.1	NW_006760324.1	+	25166051	25166773	722	-	-	-	-
Pp05_28899-3p(gma-miR1513a-5p)	18787324	PRUPE_ppa000558mg	NCBI_Assembly:GCF_000346465.1	NW_006760324.1	+	22970775	22974897	4122	-	-	GO:0005524:ATP binding	-
Pp05_28899-3p(gma-miR1513a-5p)	18788385	PRUPE_ppa020373mg	NCBI_Assembly:GCF_000346465.1	NW_006760385.1	-	13326699	13328343	1644	-	-	-	-
Pp05_28899-3p(gma-miR1513a-5p)	18788437	PRUPE_ppa021991mg	NCBI_Assembly:GCF_000346465.1	NW_006760385.1	-	24585588	24588828	3240	-	-	-	-
Pp05_28899-3p(gma-miR1513a-5p)	18788440	PRUPE_ppa014059mg	NCBI_Assembly:GCF_000346465.1	NW_006760385.1	+	17538436	17539307	871	-	-	-	-
Pp05_28899-3p(gma-miR1513a-5p)	18788456	PRUPE_ppa016752mg	NCBI_Assembly:GCF_000346465.1	NW_006760385.1	-	12173617	12177880	4263	-	-	-	-

Pp05_28899-3p(gma-miR1513a-5p)	18788469	PRUPE_ppa003781mg	NCBI_Assembly:GCF_000346465.1	NW_006760385.1	-	32756624	32761406	4782	-	GO:0000278:mitotic cell cycle;GO:0007032:endosome organization;GO:0051301:cell division	GO:0003677:DNA binding	-
Pp05_28899-3p(gma-miR1513a-5p)	18788564	PRUPE_ppa011511mg	NCBI_Assembly:GCF_000346465.1	NW_006760385.1	+	26160010	26161735	1725	-	-	-	-
Pp05_28899-3p(gma-miR1513a-5p)	18788609	PRUPE_ppa010032mg	NCBI_Assembly:GCF_000346465.1	NW_006760385.1	-	22653728	22655127	1399	-	-	-	-
Pp05_28899-3p(gma-miR1513a-5p)	18788647	PRUPE_ppa000948mg	NCBI_Assembly:GCF_000346465.1	NW_006760385.1	+	37247423	37255909	8486	-	-	GO:0016772:transferase activity, transferring phosphorus-containing groups	-

Pp05_28899-3p(gma-miR1513a-5p)	18788658	PRUPE_ppb014724mg	NCBI_Assembly:GCF_000346465.1	NW_006760385.1	-	44027771	44029050	1279	pper01100:Metabolic pathways;pper01110:Biosynthesis of secondary metabolites;pper01200:Carbon metabolism;pper00010:Glycolysis / Gluconeogenesis;pper00051:Fructose and mannose metabolism;pper00710:Carbon fixation in photosynthetic organisms;pper00030:Pentose phosphate pathway	GO:0005975:carbohydrate metabolic process	GO:0042132:fructose 1,6-bisphosphate 1-phosphatase activity	-
Pp05_28899-3p(gma-miR1513a-5p)	18788679	PRUPE_ppa022179mg	NCBI_Assembly:GCF_000346465.1	NW_006760385.1	-	22688486	22689469	983	-	GO:0030001:metal ion transport	GO:0046872:metal ion binding	-
Pp05_28899-3p(gma-miR1513a-5p)	18788740	PRUPE_ppa002909mg	NCBI_Assembly:GCF_000346465.1	NW_006760385.1	+	1904466	1913208	8742	pper04141:Protein processing in endoplasmic reticulum	-	GO:0004571:mannosyl-oligosaccharide 1,2-alpha-mannosidase activity;GO:0005509:calcium ion binding	GO:0016020:membrane

Pp05_28899-3p(gma-miR1513a-5p)	18788761	PRUPE_ppa006270mg	NCBI_Assembly:GCF_000346465.1	NW_006760385.1	+	40069058	40071969	2911	pper00480:Glutathione metabolism;pper00053:Ascorbate and aldarate metabolism	GO:0006979:response to oxidative stress	GO:0004601:peroxidase activity;GO:0020037:heme binding	-
Pp05_28899-3p(gma-miR1513a-5p)	18788765	PRUPE_ppa001110mg	NCBI_Assembly:GCF_000346465.1	NW_006760385.1	-	36235235	36238191	2956	-	-	-	-
Pp05_28899-3p(gma-miR1513a-5p)	18788769	PRUPE_ppa002039mg	NCBI_Assembly:GCF_000346465.1	NW_006760385.1	-	30566743	30574889	8146	-	GO:0043550:regulation of lipid kinase activity	-	GO:0070772:PAS complex
Pp05_28899-3p(gma-miR1513a-5p)	18788781	PRUPE_ppa024419mg	NCBI_Assembly:GCF_000346465.1	NW_006760385.1	-	39110153	39112555	2402	-	-	GO:0004674:protein serine/threonine kinase activity;GO:0005524:ATP binding;GO:0030246:carbohydrate binding	-
Pp05_28899-3p(gma-miR1513a-5p)	18788815	PRUPE_ppa017763mg	NCBI_Assembly:GCF_000346465.1	NW_006760385.1	-	36503731	36508004	4273	-	-	GO:0008236:serine-type peptidase activity	-
Pp05_28899-3p(gma-miR1513a-5p)	18788844	PRUPE_ppa026386mg	NCBI_Assembly:GCF_000346465.1	NW_006760385.1	-	36983117	36983473	356	-	-	-	-
Pp05_28899-3p(gma-miR1513a-5p)	18788861	PRUPE_ppa025354mg	NCBI_Assembly:GCF_000346465.1	NW_006760385.1	+	41223924	41225982	2058	-	-	-	-
Pp05_28899-3p(gma-miR1513a-5p)	18788900	PRUPE_ppa018549mg	NCBI_Assembly:GCF_000346465.1	NW_006760385.1	-	31268734	31269978	1244	-	-	-	-

Pp05_28899-3p(gma-miR1513a-5p)	18788943	PRUPE_ppa013645mg	NCBI_Assembly:GCF_000346465.1	NW_006760385.1	-	32474519	32475138	619	-	-	-	-
Pp05_28899-3p(gma-miR1513a-5p)	18788985	PRUPE_ppa012507mg	NCBI_Assembly:GCF_000346465.1	NW_006760385.1	-	33026579	33028018	1439	-	-	GO:0003700:sequence-specific DNA binding transcription factor activity;GO:0043565:sequence-specific DNA binding	-
Pp05_28899-3p(gma-miR1513a-5p)	18789021	PRUPE_ppa021909mg	NCBI_Assembly:GCF_000346465.1	NW_006760385.1	-	44375178	44377547	2369	-	GO:0009693:ethylene biosynthetic process;GO:0010200:response to chitin	GO:0004674:protein serine/threonine kinase activity;GO:0005524:ATP binding	-
Pp05_28899-3p(gma-miR1513a-5p)	18789068	PRUPE_ppa008769mg	NCBI_Assembly:GCF_000346465.1	NW_006760385.1	+	41748566	41749665	1099	-	-	-	-

Pp05_28899-3p(gma-miR1513a-5p)	18789083	PRUPE_ppa004179mg	NCBI_Assembly:GCF_000346465.1	NW_006760385.1	-	33664435	33666129	1694	-	-	GO:0004497:monooxygenase activity;GO:0005506:iron ion binding;GO:0016705:oxidoreductase activity, acting on paired donors, with incorporation or reduction of molecular oxygen;GO:0020037:heme binding	-
Pp05_28899-3p(gma-miR1513a-5p)	18789090	PRUPE_ppa005868mg	NCBI_Assembly:GCF_000346465.1	NW_006760385.1	+	43493633	43499211	5578	-	-	-	-
Pp05_28899-3p(gma-miR1513a-5p)	18789206	PRUPE_ppa024584mg	NCBI_Assembly:GCF_000346465.1	NW_006760385.1	+	23730398	23730691	293	-	-	-	-
Pp05_28899-3p(gma-miR1513a-5p)	18789207	PRUPE_ppa025975mg	NCBI_Assembly:GCF_000346465.1	NW_006760385.1	-	45562726	45573740	11014	pper03015:mRNA surveillance pathway	-	-	-
Pp05_28899-3p(gma-miR1513a-5p)	18789870	PRUPE_ppa003527mg	NCBI_Assembly:GCF_000346465.1	NW_006760385.1	-	1134099	1137643	3544	-	-	GO:0015231:5-formyltetrahydrofolate transporter activity;GO:0015350:methotrexate transporter activity	GO:0009941:chloroplast envelope;GO:0016021:integral component of membrane

Pp05_28899-3p(gma-miR1513a-5p)	18789922	PRUPE_ppa001534mg	NCBI_Assembly:GCF_000346465.1	NW_006760385.1	+	30788175	30791446	3271	-	GO:0001708:cell fate specification;GO:0009880:embryonic pattern specification;GO:0090421:embryonic meristem initiation	-	-
Pp05_28899-3p(gma-miR1513a-5p)	18789949	PRUPE_ppa001779mg	NCBI_Assembly:GCF_000346465.1	NW_006760385.1	-	32185525	32188584	3059	-	GO:0055085:transmembrane transport	-	GO:0016020:membrane
Pp05_28899-3p(gma-miR1513a-5p)	18789987	PRUPE_ppa025589mg	NCBI_Assembly:GCF_000346465.1	NW_006760385.1	-	702535	703262	727	-	-	-	-
Pp05_28899-3p(gma-miR1513a-5p)	18790001	PRUPE_ppa005263mg	NCBI_Assembly:GCF_000346465.1	NW_006760385.1	-	25958835	25963689	4854	-	-	-	GO:0005739:mitochondrion;GO:0009941:chloroplast envelope
Pp05_28899-3p(gma-miR1513a-5p)	18790014	PRUPE_ppa018944mg	NCBI_Assembly:GCF_000346465.1	NW_006760385.1	-	41068726	41068983	257	pper03010:Ribosome	GO:0006412:translation	GO:0003735:structural constituent of ribosome	GO:0005840:ribosome
Pp05_28899-3p(gma-miR1513a-5p)	18790022	PRUPE_ppa014485mg	NCBI_Assembly:GCF_000346465.1	NW_006760385.1	+	29680137	29680900	763	-	-	-	-
Pp05_28899-3p(gma-miR1513a-5p)	18790027	PRUPE_ppa019344mg	NCBI_Assembly:GCF_000346465.1	NW_006760385.1	+	35398877	35402139	3262	-	GO:0006457:protein folding	-	-
Pp05_28899-3p(gma-miR1513a-5p)	18790057	PRUPE_ppa012649mg	NCBI_Assembly:GCF_000346465.1	NW_006760385.1	+	9560744	9561680	936	-	GO:0006952:defense response;GO:0009607:response to biotic stimulus	-	-

Pp05_28899-3p(gma-miR1513a-5p)	18790061	PRUPE_ppa012646mg	NCBI_Assembly:GCF_000346465.1	NW_006760385.1	+	9565714	9566578	864	-	GO:0006952:defense response;GO:0009607:response to biotic stimulus	-	-
Pp05_28899-3p(gma-miR1513a-5p)	18790101	PRUPE_ppa008202mg	NCBI_Assembly:GCF_000346465.1	NW_006760385.1	-	1664460	1667987	3527	pper00130:Ubiquinone and other terpenoid-quinone biosynthesis;pper01100:Metabolic pathways;pper01110:Biosynthesis of secondary metabolites	-	GO:0008168:methyltransferase activity	-
Pp05_28899-3p(gma-miR1513a-5p)	18790115	PRUPE_ppa004542mg	NCBI_Assembly:GCF_000346465.1	NW_006760385.1	-	3655374	3657084	1710	-	-	GO:0008519:ammonium transmembrane transporter activity	GO:0016021:integral component of membrane
Pp05_28899-3p(gma-miR1513a-5p)	18790124	PRUPE_ppa011975mg	NCBI_Assembly:GCF_000346465.1	NW_006760385.1	-	46595967	46598947	2980	-	GO:0010038:response to metal ion;GO:0048573:photoperiodism, flowering	GO:0005507:copper ion binding	-
Pp05_28899-3p(gma-miR1513a-5p)	18790161	PRUPE_ppa005124mg	NCBI_Assembly:GCF_000346465.1	NW_006760385.1	+	36863797	36865685	1888	-	-	-	-

Pp05_28899-3p(gma-miR1513a-5p)	18790174	PRUPE_ppa008063mg	NCBI_Assembly:GCF_000346465.1	NW_006760385.1	+		11313358	11317353	3995	pper01100:Metabolic pathways;pper00230:Purine metabolism;pper00240:Pyrimidine metabolism;pper00480:Glutathione metabolism	GO:0006281:DNA repair;GO:0007275:multicellular organismal development;GO:0009186:deoxyribonucleoside diphosphate metabolic process;GO:0009220:pyrimidine ribonucleotide biosynthetic process;GO:0051726:regulation of cell cycle	GO:0016491:oxidoreductase activity	GO:0005829:cytosol;GO:0005971:ribonucleoside-diphosphate reductase complex
Pp05_28899-3p(gma-miR1513a-5p)	18790192	PRUPE_ppa005422mg	NCBI_Assembly:GCF_000346465.1	NW_006760385.1	+		34126400	34128549	2149	-	GO:0006810:transport;GO:0016132:brassinosteroid biosynthetic process	-	GO:0016021:integral component of membrane
Pp05_28899-3p(gma-miR1513a-5p)	18790200	PRUPE_ppa011550mg	NCBI_Assembly:GCF_000346465.1	NW_006760385.1	+		36670557	36673900	3343	-	GO:0009723:response to ethylene	-	GO:0005739:mitochondrion

Pp05_28899-3p(gma-miR1513a-5p)	18790241	PRUPE_ppa1027180mg	NCBI_Assembly:GCF_000346465.1	NW_006760385.1	+	30867484	30869785	2301	-	GO:0010413:glucuronoxylan metabolic process;GO:0045492:xylan biosynthetic process;GO:0046856:phosphatidylinositol dephosphorylation	-	-
Pp05_28899-3p(gma-miR1513a-5p)	18790305	PRUPE_ppa016805mg	NCBI_Assembly:GCF_000346465.1	NW_006760385.1	-	19891790	19893919	2129	-	-	-	-
Pp05_28899-3p(gma-miR1513a-5p)	18790340	PRUPE_ppa010207mg	NCBI_Assembly:GCF_000346465.1	NW_006760385.1	+	10309219	10311758	2539	-	GO:0006419:alanyl-tRNA aminoacylation	GO:0003676:nucleic acid binding;GO:0004813:alanine-tRNA ligase activity;GO:0005524:ATP binding	-
Pp05_28899-3p(gma-miR1513a-5p)	18790393	PRUPE_ppb023497mg	NCBI_Assembly:GCF_000346465.1	NW_006760385.1	+	9400148	9400600	452	-	-	GO:0005215:transporter activity	GO:0016020:membrane
Pp05_28899-3p(gma-miR1513a-5p)	18790457	PRUPE_ppb018399mg	NCBI_Assembly:GCF_000346465.1	NW_006760385.1	+	4202779	4203231	452	-	-	-	-
Pp05_28899-3p(gma-miR1513a-5p)	18790510	PRUPE_ppb020255mg	NCBI_Assembly:GCF_000346465.1	NW_006760385.1	-	17240693	17243909	3216	-	-	GO:0003676:nucleic acid binding;GO:0004523:RNA-DNA hybrid ribonuclease activity	-

Pp05_28899-3p(gma-miR1513a-5p)	18790513	PRUPE_ppa017979mg	NCBI_Assembly:GCF_000346465.1	NW_006760385.1	-	4247557	4253169	5612	-	-	-	-
Pp05_28899-3p(gma-miR1513a-5p)	18790529	PRUPE_ppa007144mg	NCBI_Assembly:GCF_000346465.1	NW_006760385.1	-	16057083	16061606	4523	pper01100:Metabolic pathways;pper00780:Biotin metabolism	GO:0006084:acetyl-CoA metabolic process;GO:0007020:microtubule nucleation;GO:0009102:biotin biosynthetic process;GO:0010075:regulation of meristem growth;GO:0016126:sterol biosynthetic process;GO:0016132:brassinosteroid biosynthetic process;GO:0048653:anther development	GO:0004076:biotin synthase activity;GO:0008270:zinc ion binding;GO:0051537:2 iron, 2 sulfur cluster binding;GO:0051539:4 iron, 4 sulfur cluster binding	-
Pp05_28899-3p(gma-miR1513a-5p)	18790584	PRUPE_ppa021938mg	NCBI_Assembly:GCF_000346465.1	NW_006760385.1	-	43636914	43638948	2034	-	-	GO:0003676:nucleic acid binding	-
Pp05_28899-3p(gma-miR1513a-5p)	18790591	PRUPE_ppa009861mg	NCBI_Assembly:GCF_000346465.1	NW_006760385.1	+	37328203	37331377	3174	pper03030:DNA replication	-	-	GO:0005634:nucleus
Pp05_28899-3p(gma-miR1513a-5p)	18790593	PRUPE_ppa011565mg	NCBI_Assembly:GCF_000346465.1	NW_006760385.1	-	29778849	29780417	1568	-	GO:0006333:chromatin assembly or disassembly	-	GO:0005634:nucleus

Pp05_28899-3p(gma-miR1513a-5p)	18790607	PRUPE_ppa013258mg	NCBI_Assembly:GCF_000346465.1	NW_006760385.1	-	29643915	29645084	1169	-	-	-	-
Pp05_28899-3p(gma-miR1513a-5p)	18790716	PRUPE_ppa015209mg	NCBI_Assembly:GCF_000346465.1	NW_006760385.1	-	10292585	10293142	557	-	-	-	GO:0016021: integral component of membrane
Pp05_28899-3p(gma-miR1513a-5p)	18790747	PRUPE_ppa001845mg	NCBI_Assembly:GCF_000346465.1	NW_006760385.1	+	9912782	9917014	4232	pper01100:Metabolic pathways;pper00500:Starch and sucrose metabolism	GO:0005985: sucrose metabolic process;GO:0009058:biosynthetic process	GO:0016157: sucrose synthase activity	-
Pp05_28899-3p(gma-miR1513a-5p)	18790753	PRUPE_ppa005878mg	NCBI_Assembly:GCF_000346465.1	NW_006760385.1	+	30563111	30564976	1865	-	-	-	-
Pp05_28899-3p(gma-miR1513a-5p)	18790835	PRUPE_ppa007331mg	NCBI_Assembly:GCF_000346465.1	NW_006760385.1	-	33511146	33515038	3892	-	GO:0046621: negative regulation of organ growth;GO:0048317:seed morphogenesis;GO:0048482:ovule morphogenesis	GO:0008270: zinc ion binding	-
Pp05_28899-3p(gma-miR1513a-5p)	18790863	PRUPE_ppa002677mg	NCBI_Assembly:GCF_000346465.1	NW_006760385.1	+	249809	256506	6697	-	-	-	-
Pp05_28899-3p(gma-miR1513a-5p)	18790890	PRUPE_ppa018200mg	NCBI_Assembly:GCF_000346465.1	NW_006760385.1	-	5418208	5418546	338	-	-	-	-
Pp05_28899-3p(gma-miR1513a-5p)	18790913	PRUPE_ppa003015mg	NCBI_Assembly:GCF_000346465.1	NW_006760385.1	+	13754849	13761403	6554	-	-	-	-

Pp05_28899-3p(gma-miR1513a-5p)	18790923	PRUPE_ppa1027226mg	NCBI_Assembly:GCF_000346465.1	NW_006760385.1	+	10127193	10128470	1277	pper01110:Biosynthesis of secondary metabolites;pper00062:Fatty acid elongation	GO:0000038:very long-chain fatty acid metabolic process;GO:0006633:fatty acid biosynthetic process;GO:0009409:response to cold;GO:0042335:cuticle development	GO:0016747:transferase activity, transferring acyl groups other than amino-acyl groups	GO:0016020:membrane
Pp05_28899-3p(gma-miR1513a-5p)	18790935	PRUPE_ppa006739mg	NCBI_Assembly:GCF_000346465.1	NW_006760385.1	+	38980428	38982196	1768	-	-	-	-
Pp05_28899-3p(gma-miR1513a-5p)	18790952	PRUPE_ppa008163mg	NCBI_Assembly:GCF_000346465.1	NW_006760385.1	-	13867850	13870989	3139	-	-	-	-
Pp05_28899-3p(gma-miR1513a-5p)	18790968	PRUPE_ppa017218mg	NCBI_Assembly:GCF_000346465.1	NW_006760385.1	-	3980038	3981176	1138	pper00480:Glutathione metabolism	-	-	-
Pp05_28899-3p(gma-miR1513a-5p)	18791030	PRUPE_ppa013358mg	NCBI_Assembly:GCF_000346465.1	NW_006760385.1	-	14226569	14227202	633	-	-	-	-
Pp05_28899-3p(gma-miR1513a-5p)	18791123	PRUPE_ppa005535mg	NCBI_Assembly:GCF_000346465.1	NW_006760385.1	+	38942923	38944867	1944	-	-	-	-
Pp05_28899-3p(gma-miR1513a-5p)	18791127	PRUPE_ppa026792mg	NCBI_Assembly:GCF_000346465.1	NW_006760385.1	+	36254054	36256169	2115	-	-	GO:0005509:calcium ion binding	-
Pp05_28899-3p(gma-miR1513a-5p)	18791201	PRUPE_ppa005758mg	NCBI_Assembly:GCF_000346465.1	NW_006760385.1	+	35556998	35560410	3412	-	GO:0009651:response to salt stress	-	GO:0005634:nucleus;GO:0005737:cytoplasm

Pp05_28899-3p(gma-miR1513a-5p)	18791222	PRUPE_ppa005767mg	NCBI_Assembly:GCF_000346465.1	NW_006760385.1	+	38724616	38726517	1901	-	-	-	-
Pp05_28899-3p(gma-miR1513a-5p)	18791227	PRUPE_ppa011258mg	NCBI_Assembly:GCF_000346465.1	NW_006760385.1	-	25835324	25836823	1499	-	-	GO:0008080:N-acetyltransferase activity	-
Pp05_28899-3p(gma-miR1513a-5p)	18791237	PRUPE_ppa026417mg	NCBI_Assembly:GCF_000346465.1	NW_006760385.1	+	23524309	23524584	275	-	-	GO:0005506:iron ion binding;GO:0016705:oxidoreductase activity, acting on paired donors, with incorporation or reduction of molecular oxygen;GO:0020037:heme binding	-
Pp05_28899-3p(gma-miR1513a-5p)	18791339	PRUPE_ppa020906mg	NCBI_Assembly:GCF_000346465.1	NW_006760385.1	+	25752735	25754366	1631	-	-	GO:0022857:transmembrane transporter activity	GO:0016021:integral component of membrane
Pp05_28899-3p(gma-miR1513a-5p)	18791441	PRUPE_ppa009203mg	NCBI_Assembly:GCF_000346465.1	NW_006760385.1	-	33626906	33628955	2049	-	-	-	GO:0009941:chloroplast envelope

Pp05_28899-3p(gma-miR1513a-5p)	18791455	PRUPE_ppa003950mg	NCBI_Assembly:GCF_000346465.1	NW_006760385.1	+	34892690	34895323	2633	-	-	GO:0004497:monooxygenase activity;GO:0005506:iron ion binding;GO:0016705:oxidoreductase activity, acting on paired donors, with incorporation or reduction of molecular oxygen;GO:0020037:heme binding	-
Pp05_28899-3p(gma-miR1513a-5p)	18791476	PRUPE_ppa010446mg	NCBI_Assembly:GCF_000346465.1	NW_006760385.1	+	16605538	16610257	4719	-	GO:0016485:protein processing;GO:0043085:positive regulation of catalytic activity	-	GO:0016021:integral component of membrane
Pp05_28899-3p(gma-miR1513a-5p)	18791544	PRUPE_ppa010607mg	NCBI_Assembly:GCF_000346465.1	NW_006760385.1	+	30634218	30636668	2450	-	-	-	-
Pp05_28899-3p(gma-miR1513a-5p)	18791559	PRUPE_ppa026066mg	NCBI_Assembly:GCF_000346465.1	NW_006760385.1	+	26334144	26334597	453	-	-	-	-
Pp05_28899-3p(gma-miR1513a-5p)	18791573	PRUPE_ppa023221mg	NCBI_Assembly:GCF_000346465.1	NW_006760385.1	+	31249814	31252088	2274	-	-	-	-
Pp05_28899-3p(gma-miR1513a-5p)	18791583	PRUPE_ppa017760mg	NCBI_Assembly:GCF_000346465.1	NW_006760385.1	+	24782177	24788240	6063	-	-	-	-

Pp05_28899-3p(gma-miR1513a-5p)	18791608	PRUPE_ppa000805mg	NCBI_Assembly:GCF_000346465.1	NW_006760385.1	-		36873652	36877947	4295	-	GO:0009832:plant-type cell wall biogenesis;GO:0016049:cell growth;GO:0030243:cellulose metabolic process	-	-
Pp05_28899-3p(gma-miR1513a-5p)	18791633	PRUPE_ppa020618mg	NCBI_Assembly:GCF_000346465.1	NW_006760385.1	-		46279837	46283462	3625	-	-	-	-
Pp05_28899-3p(gma-miR1513a-5p)	18791647	PRUPE_ppa011918mg	NCBI_Assembly:GCF_000346465.1	NW_006760385.1	+		39268344	39272131	3787	pper01100:Metabolic pathways;pper00230:Purine metabolism	GO:0006168:adenine salvage;GO:0009116:nucleoside metabolic process	GO:0003999:adenine phosphoribosyltransferase activity	GO:0005737:cytoplasm
Pp05_28899-3p(gma-miR1513a-5p)	18791658	PRUPE_ppa006514mg	NCBI_Assembly:GCF_000346465.1	NW_006760385.1	+		30691064	30693571	2507	-	GO:0009408:response to heat	GO:0003700:sequence-specific DNA binding transcription factor activity;GO:0043565:sequence-specific DNA binding	GO:0005634:nucleus
Pp05_28899-3p(gma-miR1513a-5p)	18791703	PRUPE_ppa004739mg	NCBI_Assembly:GCF_000346465.1	NW_006760385.1	-		42797851	42801888	4037	-	-	-	-
Pp05_28899-3p(gma-miR1513a-5p)	18791705	PRUPE_ppa019472mg	NCBI_Assembly:GCF_000346465.1	NW_006760385.1	+		34593789	34594742	953	-	-	-	GO:0016020:membrane
Pp05_28899-3p(gma-miR1513a-5p)	18791753	PRUPE_ppa001711mg	NCBI_Assembly:GCF_000346465.1	NW_006760385.1	-		34144537	34148363	3826	-	-	-	-

Pp05_28899-3p(gma-miR1513a-5p)	18791783	PRUPE_ppb022959mg	NCBI_Assembly:GCF_000346465.1	NW_006760385.1	-	21627220	21627546	326	-	-	-	-
Pp05_28899-3p(gma-miR1513a-5p)	18791818	PRUPE_ppa025327mg	NCBI_Assembly:GCF_000346465.1	NW_006760385.1	-	2315535	2317407	1872	-	-	-	-
Pp05_28899-3p(gma-miR1513a-5p)	18791832	PRUPE_ppa004908mg	NCBI_Assembly:GCF_000346465.1	NW_006760385.1	+	29434369	29438997	4628	-	-	-	GO:0009536: plastid
Pp05_28899-3p(gma-miR1513a-5p)	18791845	PRUPE_ppa018252mg	NCBI_Assembly:GCF_000346465.1	NW_006760385.1	-	7938384	7943774	5390	pper02010:ABC transporters	-	GO:0005524: ATP binding;GO:0042626:ATPase activity, coupled to transmembrane movement of substances	GO:0016021: integral component of membrane
Pp05_28899-3p(gma-miR1513a-5p)	18791870	PRUPE_ppa023392mg	NCBI_Assembly:GCF_000346465.1	NW_006760385.1	-	39306687	39310455	3768	-	-	-	-
Pp05_28899-3p(gma-miR1513a-5p)	18791894	PRUPE_ppa004062mg	NCBI_Assembly:GCF_000346465.1	NW_006760385.1	-	46207539	46211407	3868	-	GO:0018342: protein prenylation	GO:0008318: protein prenyltransferase activity	-
Pp05_28899-3p(gma-miR1513a-5p)	18791907	PRUPE_ppa009485mg	NCBI_Assembly:GCF_000346465.1	NW_006760385.1	-	42098111	42099766	1655	-	-	GO:0003824: catalytic activity;GO:0050662:coenzyme binding	GO:0005886: plasma membrane
Pp05_28899-3p(gma-miR1513a-5p)	18791939	PRUPE_ppa003098m1g	NCBI_Assembly:GCF_000346465.1	NW_006760385.1	+	31833746	31834701	955	-	-	-	-

Pp05_28899-3p(gma-miR1513a-5p)	18792023	PRUPE_ppa000916mg	NCBI_Assembly:GCF_000346465.1	NW_006760385.1	+	32431726	32434913	3187	-	-	GO:0004672:protein kinase activity;GO:0005524:ATP binding	GO:0016021:integral component of membrane
Pp05_28899-3p(gma-miR1513a-5p)	18792075	PRUPE_ppa003696mg	NCBI_Assembly:GCF_000346465.1	NW_006760385.1	+	36612429	36615348	2919	-	GO:0006352:DNA-templated transcription, initiation	GO:0001053:plastid sigma factor activity;GO:0003677:DNA binding;GO:0003700:sequence-specific DNA binding transcription factor activity;GO:0003899:DNA-directed RNA polymerase activity	-
Pp05_28899-3p(gma-miR1513a-5p)	18792115	PRUPE_ppa011188mg	NCBI_Assembly:GCF_000346465.1	NW_006760385.1	-	360157	362554	2397	-	GO:0016192:vesicle-mediated transport	-	GO:0016021:integral component of membrane

Pp05_28899-3p(gma-miR1513a-5p)	18792142	PRUPE_ppa009047mg	NCBI_Assembly:GCF_000346465.1	NW_006760385.1	-	33518003	33519851	1848	pper01100:Metabolic pathways;pper00562:Inositol phosphate metabolism;pper04070:Phosphatidylinositol signaling system	GO:0046856:phosphatidylinositol dephosphorylation	GO:0004439:phosphatidylinositol-4,5-bisphosphate 5-phosphatase activity;GO:0034485:phosphatidylinositol-3,4,5-trisphosphate 5-phosphatase activity;GO:0043813:phosphatidylinositol-3,5-bisphosphate 5-phosphatase activity	-
Pp05_28899-3p(gma-miR1513a-5p)	18792148	PRUPE_ppa000748mg	NCBI_Assembly:GCF_000346465.1	NW_006760385.1	-	34758812	34762296	3484	-	-	-	-
Pp05_28899-3p(gma-miR1513a-5p)	18792161	PRUPE_ppa005842mg	NCBI_Assembly:GCF_000346465.1	NW_006760385.1	-	32742295	32747092	4797	-	-	GO:0046872:metal ion binding	-
Pp05_28899-3p(gma-miR1513a-5p)	18792169	PRUPE_ppa000960mg	NCBI_Assembly:GCF_000346465.1	NW_006760385.1	+	25589454	25594640	5186	pper03040:Spliceosome	GO:0006397:mRNA processing	GO:0000166:nucleotide binding;GO:0003676:nucleic acid binding	-
Pp05_28899-3p(gma-miR1513a-5p)	18792240	PRUPE_ppa025682mg	NCBI_Assembly:GCF_000346465.1	NW_006760385.1	-	42095373	42096301	928	-	-	GO:0008289:lipid binding	-

Pp05_28899-3p(gma-miR1513a-5p)	18792264	PRUPE_ppa011222mg	NCBI_Assembly:GCF_000346465.1	NW_006760385.1	-	29821703	29826111	4408	-	GO:0006351:transcription, DNA-templated;GO:0016480:negative regulation of transcription from RNA polymerase III promoter	-	GO:0005634:nucleus
Pp05_28899-3p(gma-miR1513a-5p)	18792281	PRUPE_ppa024231mg	NCBI_Assembly:GCF_000346465.1	NW_006760385.1	-	12795066	12797665	2599	-	-	-	-
Pp05_28899-3p(gma-miR1513a-5p)	18792359	PRUPE_ppa008656mg	NCBI_Assembly:GCF_000346465.1	NW_006760385.1	-	34739732	34740941	1209	-	GO:0006810:transport	-	GO:0016021:integral component of membrane
Pp05_28899-3p(gma-miR1513a-5p)	18792382	PRUPE_ppa012678mg	NCBI_Assembly:GCF_000346465.1	NW_006760385.1	-	9528025	9528733	708	-	GO:0006952:defense response;GO:0009607:response to biotic stimulus	-	-
Pp05_28899-3p(gma-miR1513a-5p)	18792418	PRUPE_ppa011805mg	NCBI_Assembly:GCF_000346465.1	NW_006760385.1	-	36857500	36860042	2542	-	-	GO:0004045:aminoacyl-tRNA hydrolase activity	-
Pp05_28899-3p(gma-miR1513a-5p)	18792446	PRUPE_ppa004892mg	NCBI_Assembly:GCF_000346465.1	NW_006760385.1	+	34196848	34199557	2709	-	-	GO:0003676:nucleic acid binding;GO:0046872:metal ion binding	-
Pp05_28899-3p(gma-miR1513a-5p)	18792471	PRUPE_ppa024978mg	NCBI_Assembly:GCF_000346465.1	NW_006760385.1	-	43514712	43515545	833	-	-	GO:0008270:zinc ion binding	-
Pp05_28899-3p(gma-miR1513a-5p)	18792612	PRUPE_ppa025384mg	NCBI_Assembly:GCF_000346465.1	NW_006760385.1	-	33153997	33154959	962	-	-	-	-

Pp05_28899-3p(gma-miR1513a-5p)	18792615	PRUPE_ppa015908mg	NCBI_Assembly:GCF_000346465.1	NW_006760385.1	+	40279354	40282007	2653	-	-	GO:0016788:hydrolase activity, acting on ester bonds	-
Pp05_28899-3p(gma-miR1513a-5p)	18792644	PRUPE_ppa011729mg	NCBI_Assembly:GCF_000346465.1	NW_006760385.1	-	10670249	10672100	1851	-	-	-	GO:0005886:plasma membrane;GO:0016021:integral component of membrane
Pp05_28899-3p(gma-miR1513a-5p)	18792683	PRUPE_ppa021787mg	NCBI_Assembly:GCF_000346465.1	NW_006760385.1	-	14797629	14798291	662	-	-	-	-
Pp05_28899-3p(gma-miR1513a-5p)	18792708	PRUPE_ppa020963mg	NCBI_Assembly:GCF_000346465.1	NW_006760385.1	-	37174864	37189398	14534	-	GO:0006261:DNA-dependent DNA replication;GO:0006310:DNA recombination;GO:0009640:photomorphogenesis;GO:0010468:regulation of gene expression	GO:0003677:DNA binding;GO:0003887:DNA-directed DNA polymerase activity;GO:0005524:ATP binding	-

Pp05_28899-3p(gma-miR1513a-5p)	18792781	PRUPE_ppa010590mg	NCBI_Assembly:GCF_000346465.1	NW_006760385.1	-	27540717	27542498	1781	-	GO:0000724:double-strand break repair via homologous recombination;GO:0007062:sister chromatid cohesion;GO:0007129:synapsis;GO:0007131:reciprocal meiotic recombination;GO:0007140:male meiosis;GO:0010332:response to gamma radiation;GO:0016444:somatic cell DNA recombination;GO:0032204:regulation of telomere maintenance;GO:0032504:multicellular organism reproduction;GO:0042023:DNA endoreduplication;GO:0042138:meiotic	-	GO:0005618:cell wall
Pp05_28899-3p(gma-miR1513a-5p)	18792797	PRUPE_ppa024675mg	NCBI_Assembly:GCF_000346465.1	NW_006760385.1	+	36370337	36370813	476	-	-	-	-

Pp06_35148-3p	18766024	PRUPE_ppa014722mg	NCBI_Assembly:GCF_000346465.1	NW_006760186.1	+	1552630	1554878	2248	-	-	-	GO:0005789:endoplasmic reticulum membrane;GO:0016021:integral component of membrane
Pp06_35148-3p	18766052	PRUPE_ppa021042mg	NCBI_Assembly:GCF_000346465.1	NW_006760186.1	+	1992071	1994951	2880	-	-	GO:0008270:zinc ion binding	-
Pp06_35148-3p	18766058	PRUPE_ppa013019mg	NCBI_Assembly:GCF_000346465.1	NW_006760186.1	-	1296818	1298881	2063	pper01100:Metabolic pathways;pper00230:Purine metabolism;pper00240:Pyrimidine metabolism;pper03020:RNA polymerase	GO:0006351:transcription, DNA-templated	GO:0003677:DNA binding;GO:0003899:DNA-directed RNA polymerase activity	GO:0005665:DNA-directed RNA polymerase II, core complex
Pp06_35148-3p	18766135	PRUPE_ppa005316mg	NCBI_Assembly:GCF_000346465.1	NW_006760186.1	-	905174	908852	3678	-	-	-	-
Pp06_35148-3p	18766168	PRUPE_ppa021935mg	NCBI_Assembly:GCF_000346465.1	NW_006760194.1	-	17903682	17904413	731	-	-	-	-
Pp06_35148-3p	18766230	PRUPE_ppa015347mg	NCBI_Assembly:GCF_000346465.1	NW_006760194.1	+	655695	655928	233	-	-	-	-
Pp06_35148-3p	18766242	PRUPE_ppa008037mg	NCBI_Assembly:GCF_000346465.1	NW_006760194.1	+	4336639	4340550	3911	pper01100:Metabolic pathways;pper00564:Glycerophospholipid metabolism	GO:0046474:glycerophospholipid biosynthetic process	GO:0016301:kinase activity	-
Pp06_35148-3p	18766298	PRUPE_ppa004914mg	NCBI_Assembly:GCF_000346465.1	NW_006760194.1	-	5524413	5526872	2459	-	-	-	-

Pp06_35148-3p	18766358	PRUPE_ppa010275mg	NCBI_Assembly:GCF_000346465.1	NW_006760194.1	+	19090029	19091121	1092	-	-	GO:0003993: acid phosphatase activity	-
Pp06_35148-3p	18766363	PRUPE_ppb017205mg	NCBI_Assembly:GCF_000346465.1	NW_006760194.1	-	15603161	15603643	482	-	-	-	-
Pp06_35148-3p	18766439	PRUPE_ppa014743mg	NCBI_Assembly:GCF_000346465.1	NW_006760194.1	+	16158663	16159619	956	-	GO:0006629: lipid metabolic process	GO:0008081: phosphoric diester hydrolase activity	-
Pp06_35148-3p	18766514	PRUPE_ppa011853mg	NCBI_Assembly:GCF_000346465.1	NW_006760194.1	+	3171802	3173223	1421	-	GO:0006950: response to stress	-	-
Pp06_35148-3p	18766604	PRUPE_ppa003178mg	NCBI_Assembly:GCF_000346465.1	NW_006760194.1	-	372084	378244	6160	-	-	-	-
Pp06_35148-3p	18766803	PRUPE_ppa004348mg	NCBI_Assembly:GCF_000346465.1	NW_006760194.1	+	8635688	8639281	3593	pper00400:Phenylalanine, tyrosine and tryptophan biosynthesis;pper01100:Metabolic pathways;pper01110:Biosynthesis of secondary metabolites;pper01230:Biosynthesis of amino acids	GO:0009073: aromatic amino acid family biosynthetic process	GO:0003849: 3-deoxy-7-phosphoheptulonate synthase activity	GO:0009534: chloroplast thylakoid
Pp06_35148-3p	18766863	PRUPE_ppa009953mg	NCBI_Assembly:GCF_000346465.1	NW_006760194.1	-	14745032	14748117	3085	-	GO:0019441: tryptophan catabolic process to kynurenine	GO:0004061: arylformamidase activity	-
Pp06_35148-3p	18766867	PRUPE_ppa013146mg	NCBI_Assembly:GCF_000346465.1	NW_006760194.1	+	19956671	19959121	2450	-	-	GO:0003677: DNA binding	GO:0000786: nucleosome; GO:0005634: nucleus

Pp06_35148-3p	18767062	PRUPE_ppb016885mg	NCBI_Assembly:GCF_000346465.1	NW_006760194.1	-	10265158	10266059	901	-	-	-	-
Pp06_35148-3p	18767227	PRUPE_ppa012136mg	NCBI_Assembly:GCF_000346465.1	NW_006760194.1	-	18628377	18630998	2621	-	-	-	-
Pp06_35148-3p	18767363	PRUPE_ppa005938mg	NCBI_Assembly:GCF_000346465.1	NW_006760194.1	+	12647388	12653078	5690	pper01100:Metabolic pathways;pper04141:Protein processing in endoplasmic reticulum;pper00510:N-Glycan biosynthesis	GO:0018279:protein N-linked glycosylation via asparagine	GO:0004579:dolichyl-diphosphooligosaccharide-protein glycotransferase activity	GO:0005789:endoplasmic reticulum membrane;GO:0016021:integral component of membrane
Pp06_35148-3p	18767386	PRUPE_ppa026225mg	NCBI_Assembly:GCF_000346465.1	NW_006760194.1	-	2330830	2331856	1026	-	-	GO:0030145:manganese ion binding;GO:0045735:nutrient reservoir activity	GO:0005576:extracellular region
Pp06_35148-3p	18767457	PRUPE_ppa013038mg	NCBI_Assembly:GCF_000346465.1	NW_006760194.1	+	15071230	15073829	2599	-	-	-	-
Pp06_35148-3p	18767662	PRUPE_ppa023184mg	NCBI_Assembly:GCF_000346465.1	NW_006760194.1	+	3284710	3286350	1640	-	-	-	-
Pp06_35148-3p	18767684	PRUPE_ppa009805mg	NCBI_Assembly:GCF_000346465.1	NW_006760194.1	+	21670813	21673297	2484	-	-	-	-
Pp06_35148-3p	18768806	PRUPE_ppa002687mg	NCBI_Assembly:GCF_000346465.1	NW_006760194.1	-	16767408	16770244	2836	-	GO:0006486:protein glycosylation	-	-

Pp06_35148-3p	18769017	PRUPE_ppa003201mg	NCBI_Assembly:GCF_000346465.1	NW_006760201.1	-	21530886	21538062	7176	-	GO:0001676:long-chain fatty acid metabolic process;GO:0002213:defense response to insect;GO:0006486:protein glycosylation;GO:0006633:fatty acid biosynthetic process	-	-
Pp06_35148-3p	18769217	PRUPE_ppa001801mg	NCBI_Assembly:GCF_000346465.1	NW_006760201.1	-	9388304	9392462	4158	-	-	-	-
Pp06_35148-3p	18769240	PRUPE_ppa015931mg	NCBI_Assembly:GCF_000346465.1	NW_006760201.1	+	3881225	3886243	5018	-	-	GO:0004190:aspartic-type endopeptidase activity	-
Pp06_35148-3p	18769266	PRUPE_ppa017771mg	NCBI_Assembly:GCF_000346465.1	NW_006760201.1	-	6257661	6258302	641	-	-	-	-
Pp06_35148-3p	18769287	PRUPE_ppa010075mg	NCBI_Assembly:GCF_000346465.1	NW_006760201.1	-	17743423	17745944	2521	-	-	-	-
Pp06_35148-3p	18769408	PRUPE_ppa005181mg	NCBI_Assembly:GCF_000346465.1	NW_006760201.1	-	22555296	22560674	5378	-	GO:0006396:RNA processing	GO:0000166:nucleotide binding;GO:0003723:RNA binding	GO:0005634:nucleus;GO:0030529:ribonucleoprotein complex
Pp06_35148-3p	18769421	PRUPE_ppa020239mg	NCBI_Assembly:GCF_000346465.1	NW_006760201.1	+	21452771	21453605	834	-	-	-	-

Pp06_35148-3p	18769547	PRUPE_ppa011319mg	NCBI_Assembly:GCF_000346465.1	NW_006760201.1	-	18059048	18060355	1307	pper04120:Ubiquitin mediated proteolysis;pper04141:Protein processing in endoplasmic reticulum	-	-	-
Pp06_35148-3p	18769609	PRUPE_ppa002734mg	NCBI_Assembly:GCF_000346465.1	NW_006760201.1	-	12823736	12826713	2977	pper04626:Plant-pathogen interaction	-	GO:0004672:protein kinase activity;GO:0005509:calcium ion binding;GO:0005524:ATP binding	-
Pp06_35148-3p	18769647	PRUPE_ppa023936mg	NCBI_Assembly:GCF_000346465.1	NW_006760201.1	+	11529275	11532228	2953	-	GO:0007165:signal transduction	GO:0043531:ADP binding	-
Pp06_35148-3p	18769748	PRUPE_ppa015604mg	NCBI_Assembly:GCF_000346465.1	NW_006760201.1	-	17763031	17764737	1706	-	-	-	-
Pp06_35148-3p	18769804	PRUPE_ppa006997mg	NCBI_Assembly:GCF_000346465.1	NW_006760201.1	+	20134635	20136376	1741	-	-	-	-
Pp06_35148-3p	18769829	PRUPE_ppa026881mg	NCBI_Assembly:GCF_000346465.1	NW_006760201.1	+	1826812	1828764	1952	-	-	-	-
Pp06_35148-3p	18769866	PRUPE_ppb022654mg	NCBI_Assembly:GCF_000346465.1	NW_006760201.1	+	6610590	6611117	527	-	GO:0005975:carbohydrate metabolic process	GO:0004650:polygalacturonase activity	-
Pp06_35148-3p	18769943	PRUPE_ppa014365mg	NCBI_Assembly:GCF_000346465.1	NW_006760201.1	-	16565799	16566792	993	-	-	-	-
Pp06_35148-3p	18770023	PRUPE_ppa000467mg	NCBI_Assembly:GCF_000346465.1	NW_006760201.1	+	9712264	9716957	4693	-	GO:0046856:phosphatidylinositol dephosphorylation	-	-

Pp06_35148-3p	18770174	PRUPE_ppa005975mg	NCBI_Assembly:GCF_000346465.1	NW_006760201.1	+	1539235	1545546	6311	pper03013:RNA transport	GO:0009909:regulation of flower development;GO:0034968:histone lysine methylation;GO:0048449:floral organ formation	-	GO:0005635:nuclear envelope;GO:0005783:endoplasmic reticulum

Pp06_35148-3p	18770200	PRUPE_ppa009552mg	NCBI_Assembly:GCF_000346465.1	NW_006760201.1	+	19778850	19779956	1106	pper00270:Cysteine and methionine metabolism;pper01100:Metabolic pathways;pper01230:Biosynthesis of amino acids;pper01200:Carbon metabolism;pper00920:Sulfur metabolism	GO:0006535:cysteine biosynthetic process from serine;GO:0006612:protein targeting to membrane;GO:0009611:response to wounding;GO:0009620:response to fungus;GO:0009695:jasmonic acid biosynthetic process;GO:0009863:salicylic acid mediated signaling pathway;GO:0009867:jasmonic acid mediated signaling pathway;GO:0010363:regulation of plant-type hypersensitive response	GO:0009001:serine O-acetyltransferase activity	GO:0005829:cytosol
Pp06_35148-3p	18770208	PRUPE_ppa017499mg	NCBI_Assembly:GCF_000346465.1	NW_006760201.1	+	16602920	16605949	3029	-	GO:0009966:regulation of signal transduction	GO:0004674:protein serine/threonine kinase activity;GO:0005524:ATP binding	-

Pp06_35148-3p	18770265	PRUPE_ppa006482mg	NCBI_Assembly:GCF_000346465.1	NW_006760201.1	-	13141781	13144832	3051	-	GO:0046482:para-aminobenzoic acid metabolic process	-	GO:0016592:mediator complex
Pp06_35148-3p	18770327	PRUPE_ppa017288mg	NCBI_Assembly:GCF_000346465.1	NW_006760201.1	+	1480610	1481070	460	-	-	GO:0004497:monooxygenase activity;GO:0005506:iron ion binding;GO:0016705:oxidoreductase activity, acting on paired donors, with incorporation or reduction of molecular oxygen;GO:0020037:heme binding	-
Pp06_35148-3p	18770427	PRUPE_ppa021008mg	NCBI_Assembly:GCF_000346465.1	NW_006760201.1	+	8688340	8690157	1817	-	-	-	-
Pp06_35148-3p	18770481	PRUPE_ppa025800mg	NCBI_Assembly:GCF_000346465.1	NW_006760201.1	+	911968	912833	865	-	-	-	-
Pp06_35148-3p	18770583	PRUPE_ppa018637mg	NCBI_Assembly:GCF_000346465.1	NW_006760201.1	+	6092836	6094099	1263	-	-	-	-
Pp06_35148-3p	18770689	PRUPE_ppa002742mg	NCBI_Assembly:GCF_000346465.1	NW_006760201.1	+	22218704	22223092	4388	-	GO:0006499:N-terminal protein myristoylation	-	GO:0005794:Golgi apparatus;GO:0016021:integral component of membrane

Pp06_35148-3p	18770773	PRUPE_ppa017917mg	NCBI_Assembly:GCF_000346465.1	NW_006760201.1	+	6055908	6056635	727	-	-	GO:0000166:nucleotide binding;GO:0003676:nucleic acid binding	-
Pp06_35148-3p	18770980	PRUPE_ppa003613mg	NCBI_Assembly:GCF_000346465.1	NW_006760201.1	+	18474018	18476637	2619	pper01100:Metabolic pathways;pper01110:Biosynthesis of secondary metabolites;pper00900:Terpenoid backbone biosynthesis	GO:0008299:isoprenoid biosynthetic process;GO:0015936:coenzyme A metabolic process	GO:0004420:hydroxymethylglutaryl-CoA reductase (NADPH) activity;GO:0050661:NADP binding	GO:0005789:endoplasmic reticulum membrane;GO:0016021:integral component of membrane
Pp06_35148-3p	18771272	PRUPE_ppa022131mg	NCBI_Assembly:GCF_000346465.1	NW_006760201.1	-	12018276	12018773	497	-	-	-	-
Pp06_35148-3p	18771569	PRUPE_ppa016895mg	NCBI_Assembly:GCF_000346465.1	NW_006760201.1	+	15647246	15647934	688	-	-	-	-
Pp06_35148-3p	18771684	PRUPE_ppa025926mg	NCBI_Assembly:GCF_000346465.1	NW_006760201.1	+	20439319	20442264	2945	pper00052:Galactose metabolism	-	GO:0003824:catalytic activity	-
Pp06_35148-3p	18771807	PRUPE_ppa008132mg	NCBI_Assembly:GCF_000346465.1	NW_006760201.1	-	20115789	20117034	1245	-	-	GO:0004616:phosphogluconate dehydrogenase (decarboxylating) activity;GO:0051287:NAD binding	GO:0005829:cytosol

Pp06_35148-3p	18771808	PRUPE_ppa007476mg	NCBI_Assembly:GCF_000346465.1	NW_006760201.1	-	22463243	22466085	2842	-	GO:0006355:regulation of transcription, DNA-templated;GO:0009553:embryo sac development;GO:0009555:pollen development;GO:0009954:proximal/distal pattern formation;GO:0010227:floral organ abscission;GO:0048439:flower morphogenesis;GO:0048573:photoperiodism, flowering	GO:0003712:transcription cofactor activity;GO:0004402:histone acetyltransferase activity;GO:0008270:zinc ion binding	GO:0005634:nucleus
Pp06_35148-3p	18772030	PRUPE_ppb014943mg	NCBI_Assembly:GCF_000346465.1	NW_006760208.1	+	14961518	14963872	2354	-	-	-	-
Pp06_35148-3p	18772356	PRUPE_ppa001342mg	NCBI_Assembly:GCF_000346465.1	NW_006760208.1	+	3380611	3385221	4610	-	-	GO:0003723:RNA binding	-
Pp06_35148-3p	18772410	PRUPE_ppa015654mg	NCBI_Assembly:GCF_000346465.1	NW_006760208.1	+	17016511	17018855	2344	-	-	GO:0008270:zinc ion binding	-
Pp06_35148-3p	18772465	PRUPE_ppa009771mg	NCBI_Assembly:GCF_000346465.1	NW_006760208.1	+	4891489	4894288	2799	-	-	GO:0008270:zinc ion binding	-
Pp06_35148-3p	18772526	PRUPE_ppa015169mg	NCBI_Assembly:GCF_000346465.1	NW_006760208.1	+	16392734	16392973	239	-	-	-	-
Pp06_35148-3p	18772653	PRUPE_ppa004178mg	NCBI_Assembly:GCF_000346465.1	NW_006760208.1	-	16464455	16470155	5700	-	-	-	-

Pp06_35148-3p	18772722	PRUPE_ppa005437mg	NCBI_Assembly:GCF_000346465.1	NW_006760208.1	-	7926942	7929233	2291	-	GO:0009834:plant-type secondary cell wall biogenesis;GO:0010417:glucuronoxylan biosynthetic process	GO:0015020:glucuronosyltransferase activity	-
Pp06_35148-3p	18774094	PRUPE_ppa003334mg	NCBI_Assembly:GCF_000346465.1	NW_006760208.1	+	252099	254354	2255	-	-	-	-
Pp06_35148-3p	18774479	PRUPE_ppa020227mg	NCBI_Assembly:GCF_000346465.1	NW_006760208.1	-	288635	290585	1950	pper00073:Cutin, suberine and wax biosynthesis	GO:0000041:transition metal ion transport;GO:0006631:fatty acid metabolic process;GO:0010345:suberin biosynthetic process	GO:0005506:iron ion binding;GO:0018685:alkane 1-monooxygenase activity;GO:0020037:heme binding	GO:0005773:vacuole
Pp06_35148-3p	18775804	PRUPE_ppa024168mg	NCBI_Assembly:GCF_000346465.1	NW_006760212.1	-	6882691	6883071	380	-	-	-	-
Pp06_35148-3p	18775830	PRUPE_ppa000836mg	NCBI_Assembly:GCF_000346465.1	NW_006760212.1	+	10650619	10655953	5334	-	GO:0030001:metal ion transport	GO:0005507:copper ion binding;GO:0005524:ATP binding;GO:0019829:cation-transporting ATPase activity	GO:0016021:integral component of membrane

Pp06_35148-3p	18776122	PRUPE_ppa002509mg	NCBI_Assembly:GCF_000346465.1	NW_006760212.1	+	11196842	11199543	2701	pper04141:Protein processing in endoplasmic reticulum;pper04626:Plant-pathogen interaction	GO:0006457:protein folding;GO:0006950:response to stress	GO:0005524:ATP binding	-
Pp06_35148-3p	18777240	PRUPE_ppa023340mg	NCBI_Assembly:GCF_000346465.1	NW_006760212.1	+	987207	988895	1688	-	-	-	-
Pp06_35148-3p	18777280	PRUPE_ppa015345m1g	NCBI_Assembly:GCF_000346465.1	NW_006760212.1	+	18157482	18159444	1962	-	-	GO:0008289:lipid binding	-
Pp06_35148-3p	18777290	PRUPE_ppa002815mg	NCBI_Assembly:GCF_000346465.1	NW_006760212.1	-	1215696	1223200	7504	-	GO:0006486:protein glycosylation;GO:0052325:cell wall pectin biosynthetic process;GO:0080147:root hair cell development	GO:0052636:arabinosyltransferase activity	GO:0005768:endosome;GO:0005802:trans-Golgi network
Pp06_35148-3p	18777407	PRUPE_ppa023778mg	NCBI_Assembly:GCF_000346465.1	NW_006760212.1	-	283038	284247	1209	-	-	-	-
Pp06_35148-3p	18777516	PRUPE_ppa010581mg	NCBI_Assembly:GCF_000346465.1	NW_006760212.1	-	8362552	8366919	4367	pper04146:Peroxisome	-	-	GO:0005777:peroxisome
Pp06_35148-3p	18777552	PRUPE_ppa009551m2g	NCBI_Assembly:GCF_000346465.1	NW_006760212.1	-	15072914	15073767	853	pper03410:Base excision repair	GO:0006284:base-excision repair	GO:0003824:catalytic activity	-
Pp06_35148-3p	18777634	PRUPE_ppa006454m2g	NCBI_Assembly:GCF_000346465.1	NW_006760212.1	+	6529485	6535689	6204	-	-	-	-
Pp06_35148-3p	18777644	PRUPE_ppb015702mg	NCBI_Assembly:GCF_000346465.1	NW_006760212.1	-	17457578	17460043	2465	-	-	-	-
Pp06_35148-3p	18777662	PRUPE_ppa021099mg	NCBI_Assembly:GCF_000346465.1	NW_006760212.1	-	10181446	10182661	1215	-	-	-	-

Pp06_35148-3p	18777696	PRUPE_ppa004407mg	NCBI_Assembly:GCF_000346465.1	NW_006760212.1	-	14143464	14146681	3217	-	-	-	-
Pp06_35148-3p	18777818	PRUPE_ppa000542m2g	NCBI_Assembly:GCF_000346465.1	NW_006760212.1	+	12149845	12155276	5431	-	-	GO:0008270:zinc ion binding	-
Pp06_35148-3p	18777865	PRUPE_ppa005266mg	NCBI_Assembly:GCF_000346465.1	NW_006760212.1	-	9957499	9962285	4786	pper01100:Metabolic pathways;pper01110:Biosynthesis of secondary metabolites;pper00280:Valine, leucine and isoleucine degradation;pper00900:Terpenoid backbone biosynthesis;pper00650:Butanoate metabolism;pper00072:Synthesis and degradation of ketone bodies	GO:0019287:isopentenyl diphosphate biosynthetic process, mevalonate pathway	GO:0004421:hydroxymethylglutaryl-CoA synthase activity	GO:0005829:cytosol;GO:0009506:plasmodesma
Pp06_35148-3p	18778066	PRUPE_ppa027215mg	NCBI_Assembly:GCF_000346465.1	NW_006760212.1	+	9214602	9215231	629	-	-	-	-

Pp06_35148-3p	18778090	PRUPE_ppa007072mg	NCBI_Assembly:GCF_000346465.1	NW_006760212.1	+	15759055	15761512	2457	-	GO:0000165:MAPK cascade;GO:0006355:regulation of transcription, DNA-templated;GO:0006612:protein targeting to membrane;GO:0009617:response to bacterium;GO:0009862:systemic acquired resistance, salicylic acid mediated signaling pathway;GO:0009867:jasmonic acid mediated signaling pathway;GO:0010310:regulation of hydrogen peroxide metabolic process;GO:0010363:regulation of plant-type hypersensitive	-	GO:0009535:chloroplast thylakoid membrane;GO:0009941:chloroplast envelope
Pp06_35148-3p	18778335	PRUPE_ppa021047mg	NCBI_Assembly:GCF_000346465.1	NW_006760220.1	+	399449	400225	776	-	GO:0048446:petal morphogenesis	-	-

Pp06_35148-3p	18778337	PRUPE_ppa015439mg	NCBI_Assembly:GCF_000346465.1	NW_006760220.1	+	835715	836731	1016	-	-	GO:0004672:protein kinase activity;GO:0005524:ATP binding	-
Pp06_35148-3p	18778357	PRUPE_ppa024108mg	NCBI_Assembly:GCF_000346465.1	NW_006760220.1	-	8000806	8001229	423	-	-	-	-
Pp06_35148-3p	18778590	PRUPE_ppa013691mg	NCBI_Assembly:GCF_000346465.1	NW_006760220.1	+	21527769	21528534	765	pper03010:Ribosome	-	-	-
Pp06_35148-3p	18778633	PRUPE_ppa009325mg	NCBI_Assembly:GCF_000346465.1	NW_006760220.1	+	7332623	7335850	3227	pper04146:Peroxisome	-	GO:0016491:oxidoreductase activity	-

Pp06_35148-3p	18778648	PRUPE_ppa001558mg	NCBI_Assembly:GCF_000346465.1	NW_006760220.1	+	27311988	27325128	13140	-	GO:0006486:protein glycosylation;GO:0007062:sister chromatid cohesion;GO:0007129:synapsis;GO:0007131:reciprocal meiotic recombination;GO:0009887:organ morphogenesis;GO:0009888:tissue development;GO:0010332:response to gamma radiation;GO:0010413:glucuronoxylan metabolic process;GO:0010638:positive regulation of organelle organization;GO:0016926:protein desumoylation;GO:0032204:regulation of telomere maintenance;GO:0032504	-	-

Pp06_35148-3p	18778679	PRUPE_ppa004855mg	NCBI_Assembly:GCF_000346465.1	NW_006760220.1	+	6451302	6455234	3932	-	GO:0006364:rRNA processing;GO:0006399:tRNA metabolic process;GO:0006655:phosphatidylglycerol biosynthetic process;GO:0009658:chloroplast organization;GO:0010103:stomatal complex morphogenesis;GO:0042793:transcription from plastid promoter;GO:0045036:protein targeting to chloroplast;GO:0045893:positive regulation of transcription, DNA-templated	-	GO:0009507:chloroplast;GO:0009508:plastid chromosome
Pp06_35148-3p	18778720	PRUPE_ppa006471mg	NCBI_Assembly:GCF_000346465.1	NW_006760220.1	-	25896489	25897875	1386	-	-	GO:0004842:ubiquitin-protein transferase activity;GO:0016874:ligase activity	-

Pp06_35148-3p	18778821	PRUPE_ppb014490mg	NCBI_Assembly:GCF_000346465.1	NW_006760220.1	+	1340088	1342853	2765	-	-	GO:0016772: transferase activity, transferring phosphorus-containing groups	-
Pp06_35148-3p	18779021	PRUPE_ppb023407mg	NCBI_Assembly:GCF_000346465.1	NW_006760220.1	-	20434290	20435258	968	-	-	-	-
Pp06_35148-3p	18779083	PRUPE_ppa014289mg	NCBI_Assembly:GCF_000346465.1	NW_006760220.1	-	27561030	27561484	454	-	-	-	-
Pp06_35148-3p	18779119	PRUPE_ppa002276mg	NCBI_Assembly:GCF_000346465.1	NW_006760220.1	+	333118	335524	2406	-	-	GO:0008270: zinc ion binding	-
Pp06_35148-3p	18779469	PRUPE_ppa017853mg	NCBI_Assembly:GCF_000346465.1	NW_006760220.1	-	14761057	14766856	5799	-	-	GO:0004674: protein serine/threonine kinase activity;GO:0005524:ATP binding	-
Pp06_35148-3p	18779770	PRUPE_ppa015170mg	NCBI_Assembly:GCF_000346465.1	NW_006760220.1	-	27188060	27189536	1476	-	-	-	-
Pp06_35148-3p	18779819	PRUPE_ppb001416mg	NCBI_Assembly:GCF_000346465.1	NW_006760220.1	+	29745034	29748657	3623	-	-	-	-
Pp06_35148-3p	18779828	PRUPE_ppa018356mg	NCBI_Assembly:GCF_000346465.1	NW_006760220.1	+	21973079	21973486	407	-	-	GO:0008270: zinc ion binding	-
Pp06_35148-3p	18779842	PRUPE_ppa006626mg	NCBI_Assembly:GCF_000346465.1	NW_006760220.1	-	926547	929833	3286	-	GO:0006486: protein glycosylation	GO:0008378: galactosyltransferase activity	GO:0005794: Golgi apparatus;GO:0016021:integral component of membrane
Pp06_35148-3p	18779844	PRUPE_ppa017511mg	NCBI_Assembly:GCF_000346465.1	NW_006760220.1	-	26695883	26696467	584	-	-	-	-

Pp06_35148-3p	18779972	PRUPE_ppa027072mg	NCBI_Assembly:GCF_000346465.1	NW_006760220.1	-		26376712	26377404	692	-	-	GO:0003700:sequence-specific DNA binding transcription factor activity;GO:0043565:sequence-specific DNA binding	GO:0005634:nucleus
Pp06_35148-3p	18780079	PRUPE_ppa015082mg	NCBI_Assembly:GCF_000346465.1	NW_006760220.1	-		24810888	24811947	1059	pper01100:Metabolic pathways;pper01110:Biosynthesis of secondary metabolites;pper00860:Porphyrin and chlorophyll metabolism	-	GO:0004659:prenyltransferase activity	GO:0016021:integral component of membrane
Pp06_35148-3p	18780083	PRUPE_ppa018961mg	NCBI_Assembly:GCF_000346465.1	NW_006760220.1	-		3229895	3233612	3717	-	-	-	-
Pp06_35148-3p	18780166	PRUPE_ppa019945mg	NCBI_Assembly:GCF_000346465.1	NW_006760220.1	+		28102359	28105053	2694	-	-	-	-
Pp06_35148-3p	18780253	PRUPE_ppa026938mg	NCBI_Assembly:GCF_000346465.1	NW_006760220.1	-		20947178	20948280	1102	-	-	-	-
Pp06_35148-3p	18780259	PRUPE_ppa013996mg	NCBI_Assembly:GCF_000346465.1	NW_006760220.1	-		9239703	9240211	508	-	-	-	-
Pp06_35148-3p	18780540	PRUPE_ppa018824mg	NCBI_Assembly:GCF_000346465.1	NW_006760220.1	-		7647533	7649132	1599	-	-	-	-

Pp06_35148-3p	18781668	PRUPE_ppa004502mg	NCBI_Assembly:GCF_000346465.1	NW_006760268.1	-	1888163	1890891	2728	-	GO:0007020:microtubule nucleation	-	GO:0005794:Golgi apparatus;GO:0016021:integral component of membrane
Pp06_35148-3p	18782844	PRUPE_ppa016387mg	NCBI_Assembly:GCF_000346465.1	NW_006760268.1	-	14493217	14495029	1812	-	-	GO:0004674:protein serine/threonine kinase activity;GO:0030247:polysaccharide binding	GO:0016021:integral component of membrane
Pp06_35148-3p	18782941	PRUPE_ppa020913mg	NCBI_Assembly:GCF_000346465.1	NW_006760268.1	+	15437449	15440114	2665	-	-	-	-
Pp06_35148-3p	18783287	PRUPE_ppa020737mg	NCBI_Assembly:GCF_000346465.1	NW_006760268.1	+	17489721	17490094	373	-	-	-	-
Pp06_35148-3p	18783351	PRUPE_ppa006304mg	NCBI_Assembly:GCF_000346465.1	NW_006760268.1	-	615190	617052	1862	pper01100:Metabolic pathways	-	GO:0008375:acetylglucosaminyltransferase activity	GO:0016020:membrane
Pp06_35148-3p	18783452	PRUPE_ppa009567mg	NCBI_Assembly:GCF_000346465.1	NW_006760268.1	-	5624837	5627233	2396	-	-	-	-
Pp06_35148-3p	18783500	PRUPE_ppa014784mg	NCBI_Assembly:GCF_000346465.1	NW_006760268.1	-	11768585	11768827	242	-	-	-	-
Pp06_35148-3p	18783531	PRUPE_ppa014910mg	NCBI_Assembly:GCF_000346465.1	NW_006760268.1	-	11116982	11117272	290	-	-	-	-
Pp06_35148-3p	18783629	PRUPE_ppa024733mg	NCBI_Assembly:GCF_000346465.1	NW_006760268.1	+	17645194	17649622	4428	pper00904:Diterpenoid biosynthesis	-	GO:0000287:magnesium ion binding;GO:0010333:terpene synthase activity	-

Pp06_35148-3p	18783700	PRUPE_ppa024541mg	NCBI_Assembly:GCF_000346465.1	NW_006760268.1	-	4983794	4985276	1482	-	GO:0045454: cell redox homeostasis	GO:0009055: electron carrier activity;GO:0015035:protein disulfide oxidoreductase activity	GO:0005623: cell
Pp06_35148-3p	18783877	PRUPE_ppa002483mg	NCBI_Assembly:GCF_000346465.1	NW_006760268.1	-	1143176	1149774	6598	-	-	-	GO:0005829: cytosol
Pp06_35148-3p	18783929	PRUPE_ppa026859mg	NCBI_Assembly:GCF_000346465.1	NW_006760268.1	-	174172	180878	6706	-	-	GO:0046872: metal ion binding	GO:0005773: vacuole;GO:0005886:plasma membrane
Pp06_35148-3p	18783999	PRUPE_ppa024040mg	NCBI_Assembly:GCF_000346465.1	NW_006760268.1	+	15449016	15449414	398	-	-	GO:0003676: nucleic acid binding	-
Pp06_35148-3p	18784060	PRUPE_ppa023475mg	NCBI_Assembly:GCF_000346465.1	NW_006760268.1	+	10690432	10691335	903	-	-	-	-
Pp06_35148-3p	18784154	PRUPE_ppa020324mg	NCBI_Assembly:GCF_000346465.1	NW_006760268.1	-	7797149	7799510	2361	-	-	-	-
Pp06_35148-3p	18784191	PRUPE_ppa009766mg	NCBI_Assembly:GCF_000346465.1	NW_006760268.1	-	21211815	21212784	969	-	-	-	GO:0016020: membrane
Pp06_35148-3p	18784258	PRUPE_ppa001533mg	NCBI_Assembly:GCF_000346465.1	NW_006760268.1	+	19717958	19720378	2420	-	GO:0009220: pyrimidine ribonucleotide biosynthetic process	GO:0016817: hydrolase activity, acting on acid anhydrides	-
Pp06_35148-3p	18784349	PRUPE_ppa022043mg	NCBI_Assembly:GCF_000346465.1	NW_006760268.1	-	14127308	14129738	2430	-	GO:0006351: transcription, DNA-templated;GO:0006355:regulation of transcription, DNA-templated	GO:0003677: DNA binding	GO:0005634: nucleus

Pp06_35148-3p	18784350	PRUPE_ppa002196mg	NCBI_Assembly:GCF_000346465.1	NW_006760268.1	+	13461891	13464438	2547	-	-	-	-
Pp06_35148-3p	18784371	PRUPE_ppb011914mg	NCBI_Assembly:GCF_000346465.1	NW_006760268.1	+	2566924	2567757	833	-	-	-	-
Pp06_35148-3p	18784417	PRUPE_ppa013099mg	NCBI_Assembly:GCF_000346465.1	NW_006760268.1	+	1472725	1473763	1038	-	-	-	-
Pp06_35148-3p	18784516	PRUPE_ppa015089m1g	NCBI_Assembly:GCF_000346465.1	NW_006760268.1	+	10081871	10082515	644	-	-	GO:0008375:acetylglucosaminyltransferase activity	GO:0016020:membrane
Pp06_35148-3p	18784523	PRUPE_ppa026065mg	NCBI_Assembly:GCF_000346465.1	NW_006760276.1	+	16570	20446	3876	-	GO:0007165:signal transduction	GO:0043531:ADP binding	-
Pp06_35148-3p	18784527	PRUPE_ppa012875mg	NCBI_Assembly:GCF_000346465.1	NW_006760281.1	-	8132	8642	510	-	-	GO:0016788:hydrolase activity, acting on ester bonds	-
Pp06_35148-3p	18784577	PRUPE_ppa000601mg	NCBI_Assembly:GCF_000346465.1	NW_006760324.1	-	26436646	26447351	10705	pper03013:RNA transport;pper03008:Ribosome biogenesis in eukaryotes	GO:0006886:intracellular protein transport	-	GO:0005622:intracellular
Pp06_35148-3p	18784735	PRUPE_ppa017245mg	NCBI_Assembly:GCF_000346465.1	NW_006760324.1	-	14923804	14926228	2424	-	-	GO:0000166:nucleotide binding;GO:0003676:nucleic acid binding	-

Pp06_35148-3p	18784908	PRUPE_ppa022719mg	NCBI_Assembly:GCF_000346465.1	NW_006760324.1	-		24808732	24809394	662	-	GO:0006351: transcription, DNA-templated	GO:0003677: DNA binding;GO:0003700:sequence-specific DNA binding transcription factor activity	GO:0005634: nucleus
Pp06_35148-3p	18784962	PRUPE_ppa021915mg	NCBI_Assembly:GCF_000346465.1	NW_006760324.1	+		3204873	3208022	3149	-	-	GO:0003676: nucleic acid binding	-
Pp06_35148-3p	18784966	PRUPE_ppb018863mg	NCBI_Assembly:GCF_000346465.1	NW_006760324.1	-		24709980	24713534	3554	-	-	-	-
Pp06_35148-3p	18785016	PRUPE_ppa027011mg	NCBI_Assembly:GCF_000346465.1	NW_006760324.1	-		17263817	17264461	644	-	GO:0005975: carbohydrate metabolic process	GO:0004553: hydrolase activity, hydrolyzing O-glycosyl compounds	-
Pp06_35148-3p	18785061	PRUPE_ppa021712mg	NCBI_Assembly:GCF_000346465.1	NW_006760324.1	-		12756526	12758489	1963	-	-	-	-
Pp06_35148-3p	18785125	PRUPE_ppa022097mg	NCBI_Assembly:GCF_000346465.1	NW_006760324.1	+		7335875	7337407	1532	-	-	-	-
Pp06_35148-3p	18785249	PRUPE_ppa002934mg	NCBI_Assembly:GCF_000346465.1	NW_006760324.1	+		20370714	20373535	2821	-	-	-	GO:0005829: cytosol
Pp06_35148-3p	18785251	PRUPE_ppa011750mg	NCBI_Assembly:GCF_000346465.1	NW_006760324.1	-		18847955	18850913	2958	pper03008:Ribosome biogenesis in eukaryotes	-	-	GO:0032040: small-subunit processome
Pp06_35148-3p	18785379	PRUPE_ppa025704mg	NCBI_Assembly:GCF_000346465.1	NW_006760324.1	-		12687799	12693189	5390	-	-	-	-
Pp06_35148-3p	18785422	PRUPE_ppb017509mg	NCBI_Assembly:GCF_000346465.1	NW_006760324.1	-		19715450	19717482	2032	-	-	GO:0005509: calcium ion binding	-

Pp06_35148-3p	18785536	PRUPE_ppa023035mg	NCBI_Assembly:GCF_000346465.1	NW_006760324.1	+	25114102	25114686	584	-	-	GO:0004497:monooxygenase activity;GO:0005506:iron ion binding;GO:0016705:oxidoreductase activity, acting on paired donors, with incorporation or reduction of molecular oxygen;GO:0020037:heme binding	-
Pp06_35148-3p	18785597	PRUPE_ppa024844mg	NCBI_Assembly:GCF_000346465.1	NW_006760324.1	-	19745577	19746476	899	-	-	-	-
Pp06_35148-3p	18785688	PRUPE_ppa010939mg	NCBI_Assembly:GCF_000346465.1	NW_006760324.1	+	20212785	20214825	2040	-	-	-	-
Pp06_35148-3p	18785691	PRUPE_ppa007399mg	NCBI_Assembly:GCF_000346465.1	NW_006760324.1	+	25893485	25897079	3594	pper00270:Cysteine and methionine metabolism;pper01100:Metabolic pathways;pper00330:Arginine and proline metabolism;pper00480:Glutathione metabolism;pper00410:beta-Alanine metabolism	GO:0006595:polyamine metabolic process	GO:0016740:transferase activity	-

Pp06_35148-3p	18785771	PRUPE_ppb022177mg	NCBI_Assembly:GCF_000346465.1	NW_006760324.1	-	24327317	24328031	714	-	-	-	-
Pp06_35148-3p	18785809	PRUPE_ppa021980mg	NCBI_Assembly:GCF_000346465.1	NW_006760324.1	-	13244289	13245686	1397	-	GO:0006633: fatty acid biosynthetic process	GO:0016747: transferase activity, transferring acyl groups other than amino-acyl groups	GO:0016020: membrane
Pp06_35148-3p	18785952	PRUPE_ppa013301mg	NCBI_Assembly:GCF_000346465.1	NW_006760324.1	+	26103315	26103943	628	pper04075:Plant hormone signal transduction	-	-	-
Pp06_35148-3p	18786029	PRUPE_ppa017031mg	NCBI_Assembly:GCF_000346465.1	NW_006760324.1	-	17935631	17938461	2830	-	-	-	-
Pp06_35148-3p	18786048	PRUPE_ppa022775mg	NCBI_Assembly:GCF_000346465.1	NW_006760324.1	+	15607871	15608251	380	-	-	-	-
Pp06_35148-3p	18786252	PRUPE_ppb015196mg	NCBI_Assembly:GCF_000346465.1	NW_006760324.1	-	23803300	23804047	747	pper01100:Metabolic pathways;pper00500:Starch and sucrose metabolism;pper00040:Pentose and glucuronate interconversions	GO:0042545: cell wall modification	GO:0030599: pectinesterase activity;GO:0045330:aspartyl esterase activity	GO:0005618: cell wall

Pp06_35148-3p	18786285	PRUPE_ppa005887mg	NCBI_Assembly:GCF_000346465.1	NW_006760324.1	+	26724406	26726833	2427	-	GO:0006810:transport;GO:0007623:circadian rhythm;GO:0009409:response to cold;GO:0009414:response to water deprivation;GO:0009737:response to abscisic acid	-	GO:0016021:integral component of membrane
Pp06_35148-3p	18786370	PRUPE_ppa012926mg	NCBI_Assembly:GCF_000346465.1	NW_006760324.1	+	13391432	13392525	1093	-	GO:0006351:transcription, DNA-templated;GO:0006355:regulation of transcription, DNA-templated	GO:0003677:DNA binding	GO:0005634:nucleus
Pp06_35148-3p	18786399	PRUPE_ppa007968mg	NCBI_Assembly:GCF_000346465.1	NW_006760324.1	+	2459908	2461986	2078	-	-	GO:0003700:sequence-specific DNA binding transcription factor activity	-
Pp06_35148-3p	18786428	PRUPE_ppa012559mg	NCBI_Assembly:GCF_000346465.1	NW_006760324.1	+	26650533	26653409	2876	-	GO:0006260:DNA replication	GO:0003677:DNA binding	GO:0005664:nuclear origin of replication recognition complex
Pp06_35148-3p	18786521	PRUPE_ppa011802mg	NCBI_Assembly:GCF_000346465.1	NW_006760324.1	-	22044624	22045692	1068	-	-	-	GO:0005886:plasma membrane;GO:0016021:integral component of membrane

Pp06_35148-3p	18786690	PRUPE_ppa021745mg	NCBI_Assembly:GCF_000346465.1	NW_006760324.1	-	10957778	10958536	758	-	-	-	-
Pp06_35148-3p	18786695	PRUPE_ppa006786mg	NCBI_Assembly:GCF_000346465.1	NW_006760324.1	-	20357554	20360409	2855	-	-	GO:0016787:hydrolase activity	-
Pp06_35148-3p	18786786	PRUPE_ppa003980mg	NCBI_Assembly:GCF_000346465.1	NW_006760324.1	+	25112006	25113619	1613	-	-	GO:0004497:monooxygenase activity;GO:0005506:iron ion binding;GO:0016705:oxidoreductase activity, acting on paired donors, with incorporation or reduction of molecular oxygen;GO:0020037:heme binding	-
Pp06_35148-3p	18786953	PRUPE_ppa026509mg	NCBI_Assembly:GCF_000346465.1	NW_006760324.1	+	19057834	19059326	1492	-	GO:0006694:steroid biosynthetic process	GO:0003854:3-beta-hydroxy-delta5-steroid dehydrogenase activity	-
Pp06_35148-3p	18787053	PRUPE_ppa014450mg	NCBI_Assembly:GCF_000346465.1	NW_006760324.1	+	9952485	9952982	497	-	-	-	-
Pp06_35148-3p	18788500	PRUPE_ppa003684mg	NCBI_Assembly:GCF_000346465.1	NW_006760385.1	-	44761841	44765436	3595	-	-	-	-
Pp06_35148-3p	18788503	PRUPE_ppa013587mg	NCBI_Assembly:GCF_000346465.1	NW_006760385.1	+	24959406	24960874	1468	-	-	-	-

Pp06_35148-3p	18788591	PRUPE_ppa002142mg	NCBI_Assembly:GCF_000346465.1	NW_006760385.1	+	5907048	5911889	4841	-	-	GO:0003958:NADPH-hemoprotein reductase activity;GO:0005506:iron ion binding;GO:0010181:FMN binding	GO:0005789:endoplasmic reticulum membrane
Pp06_35148-3p	18788599	PRUPE_ppa024089mg	NCBI_Assembly:GCF_000346465.1	NW_006760385.1	-	16436803	16438448	1645	-	-	GO:0005351:sugar:proton symporter activity	GO:0000139:Golgi membrane;GO:0016021:integral component of membrane
Pp06_35148-3p	18788655	PRUPE_ppa006898mg	NCBI_Assembly:GCF_000346465.1	NW_006760385.1	-	32790797	32794204	3407	-	GO:0009790:embryo development;GO:0048700:acquisition of desiccation tolerance in seed	-	GO:0080008:Cul4-RING E3 ubiquitin ligase complex
Pp06_35148-3p	18788680	PRUPE_ppa007555mg	NCBI_Assembly:GCF_000346465.1	NW_006760385.1	+	46625929	46627623	1694	-	GO:0048446:petal morphogenesis	GO:0003700:sequence-specific DNA binding transcription factor activity	-

Pp06_35148-3p	18788748	PRUPE_ppa005724mg	NCBI_Assembly:GCF_000346465.1	NW_006760385.1	-	24616229	24619139	2910	-	GO:0006499:N-terminal protein myristoylation;GO:0009697:salicylic acid biosynthetic process;GO:0009863:salicylic acid mediated signaling pathway;GO:0031348:negative regulation of defense response;GO:0045087:innate immune response	GO:0008270:zinc ion binding	-
Pp06_35148-3p	18788784	PRUPE_ppa008515mg	NCBI_Assembly:GCF_000346465.1	NW_006760385.1	-	7979449	7981602	2153	-	GO:0001522:pseudouridine synthesis;GO:0006730:one-carbon metabolic process;GO:0008033:tRNA processing	GO:0003723:RNA binding;GO:0009982:pseudouridine synthase activity	-
Pp06_35148-3p	18788867	PRUPE_ppa005999mg	NCBI_Assembly:GCF_000346465.1	NW_006760385.1	-	17923124	17926991	3867	-	-	GO:0003700:sequence-specific DNA binding transcription factor activity;GO:0043565:sequence-specific DNA binding	-

Pp06_35148-3p	18788999	PRUPE_ppa002630mg	NCBI_Assembly:GCF_000346465.1	NW_006760385.1	+	30828156	30832580	4424	-	-	-	GO:0005829:cytosol
Pp06_35148-3p	18789137	PRUPE_ppa024277mg	NCBI_Assembly:GCF_000346465.1	NW_006760385.1	+	21447694	21451515	3821	-	-	-	-
Pp06_35148-3p	18789235	PRUPE_ppa017075mg	NCBI_Assembly:GCF_000346465.1	NW_006760385.1	+	42507333	42508910	1577	-	GO:0006359:regulation of transcription from RNA polymerase III promoter	-	-
Pp06_35148-3p	18789840	PRUPE_ppa011357mg	NCBI_Assembly:GCF_000346465.1	NW_006760385.1	-	3298459	3302077	3618	pper03013:RNA transport	GO:0009615:response to virus;GO:0050687:negative regulation of defense response to virus	GO:0000340:RNA 7-methylguanosine cap binding;GO:0003743:translation initiation factor activity	GO:0005634:nucleus;GO:0005829:cytosol;GO:0005845:mRNA cap binding complex
Pp06_35148-3p	18789876	PRUPE_ppa022727mg	NCBI_Assembly:GCF_000346465.1	NW_006760385.1	+	25294797	25296842	2045	-	GO:0009688:abscisic acid biosynthetic process	-	-
Pp06_35148-3p	18790054	PRUPE_ppa023311mg	NCBI_Assembly:GCF_000346465.1	NW_006760385.1	+	32995619	32997520	1901	-	GO:0006355:regulation of transcription, DNA-templated	GO:0003677:DNA binding	GO:0005634:nucleus

Pp06_35148-3p	18790071	PRUPE_ppa012961mg	NCBI_Assembly:GCF_000346465.1	NW_006760385.1	-	43467373	43467895	522	-	GO:0009555:pollen development;GO:0009793:embryo development ending in seed dormancy;GO:0042744:hydrogen peroxide catabolic process	-	GO:0005886:plasma membrane
Pp06_35148-3p	18790119	PRUPE_ppa000590mg	NCBI_Assembly:GCF_000346465.1	NW_006760385.1	+	6257961	6263782	5821	-	-	GO:0004712:protein serine/threonine/tyrosine kinase activity;GO:0005524:ATP binding	-
Pp06_35148-3p	18790327	PRUPE_ppa012776mg	NCBI_Assembly:GCF_000346465.1	NW_006760385.1	-	40730863	40732397	1534	pper01100:Metabolic pathways;pper00230:Purine metabolism;pper00240:Pyrimidine metabolism;pper03020:RNA polymerase	GO:0006359:regulation of transcription from RNA polymerase III promoter	-	-
Pp06_35148-3p	18790332	PRUPE_ppa011014mg	NCBI_Assembly:GCF_000346465.1	NW_006760385.1	+	27134558	27136178	1620	-	-	GO:0003723:RNA binding;GO:0033897:ribonuclease T2 activity	-

Pp06_35148-3p	18790378	PRUPE_ppb019696mg	NCBI_Assembly:GCF_000346465.1	NW_006760385.1	-	25870101	25871332	1231	pper01100:Metabolic pathways;pper01110:Biosynthesis of secondary metabolites;pper01230:Biosynthesis of amino acids;pper00330:Arginine and proline metabolism;pper01210:2-Oxocarboxylic acid metabolism	GO:0006526:arginine biosynthetic process	GO:0004042:acetyl-CoA:L-glutamate N-acetyltransferase activity	GO:0005737:cytoplasm
Pp06_35148-3p	18790393	PRUPE_ppb023497mg	NCBI_Assembly:GCF_000346465.1	NW_006760385.1	+	9400148	9400600	452	-	-	GO:0005215:transporter activity	GO:0016020:membrane
Pp06_35148-3p	18790487	PRUPE_ppa023250mg	NCBI_Assembly:GCF_000346465.1	NW_006760385.1	+	36053172	36055225	2053	-	GO:0006351:transcription, DNA-templated;GO:0006355:regulation of transcription, DNA-templated	GO:0003677:DNA binding	GO:0005634:nucleus
Pp06_35148-3p	18790673	PRUPE_ppa1027158mg	NCBI_Assembly:GCF_000346465.1	NW_006760385.1	+	38063633	38064350	717	-	-	GO:0003676:nucleic acid binding;GO:0008270:zinc ion binding	-
Pp06_35148-3p	18790684	PRUPE_ppa005744mg	NCBI_Assembly:GCF_000346465.1	NW_006760385.1	+	26634972	26636738	1766	pper00040:Pentose and glucuronate interconversions	GO:0045490:pectin catabolic process	GO:0030570:pectate lyase activity;GO:0046872:metal ion binding	-

Pp06_35148-3p	18790877	PRUPE_ppa006744mg	NCBI_Assembly:GCF_000346465.1	NW_006760385.1	-	45630298	45636209	5911	pper04075:Plant hormone signal transduction	GO:0006351:transcription, DNA-templated;GO:0006355:regulation of transcription, DNA-templated;GO:0009734:auxin-activated signaling pathway	-	GO:0005634:nucleus
Pp06_35148-3p	18790991	PRUPE_ppa002141mg	NCBI_Assembly:GCF_000346465.1	NW_006760385.1	+	1484592	1491373	6781	-	GO:0008284:positive regulation of cell proliferation	-	-

Pp06_35148-3p	18791014	PRUPE_ppa005369mg	NCBI_Assembly:GCF_000346465.1	NW_006760385.1	-	33853870	33857562	3692	pper00270:Cysteine and methionine metabolism;pper00350:Tyrosine metabolism;pper00360:Phenylalanine metabolism;pper00400:Phenylalanine, tyrosine and tryptophan biosynthesis;pper00950:Isoquinoline alkaloid biosynthesis;pper00960:Tropane, piperidine and pyridine alkaloid biosynthesis;pper01100:Metabolic pathways;pper01110:Biosynthesis of secondary metabolites;pper01230:Biosynthesis of amino acids;pper00330:Arginine and proline metabolism;p	GO:0006520:cellular amino acid metabolic process;GO:0009693:ethylene biosynthetic process	GO:0004069:L-aspartate:2-oxoglutarate aminotransferase activity;GO:0030170:pyridoxal phosphate binding;GO:0080130:L-phenylalanine:2-oxoglutarate aminotransferase activity	GO:0009570:chloroplast stroma;GO:0009941:chloroplast envelope;GO:0010319:stromule;GO:0048046:apoplast
Pp06_35148-3p	18791173	PRUPE_ppa016275mg	NCBI_Assembly:GCF_000346465.1	NW_006760385.1	+	16446010	16447825	1815	pper03013:RNA transport	GO:0000245:spliceosomal complex assembly;GO:0000387:spliceosomal snRNP assembly	-	GO:0005681:spliceosomal complex

Pp06_35148-3p	18791517	PRUPE_ppa021831mg	NCBI_Assembly:GCF_000346465.1	NW_006760385.1	-	34710147	34712467	2320	-	-	GO:0004674:protein serine/threonine kinase activity;GO:0005524:ATP binding	-	
Pp06_35148-3p	18791528	PRUPE_ppa003501mg	NCBI_Assembly:GCF_000346465.1	NW_006760385.1	+	925909	929040	3131	-	GO:0006457:protein folding;GO:0010286:heat acclimation	GO:0005524:ATP binding	-	
Pp06_35148-3p	18791559	PRUPE_ppa026066mg	NCBI_Assembly:GCF_000346465.1	NW_006760385.1	+	26334144	26334597	453	-	-	-	-	
Pp06_35148-3p	18791618	PRUPE_ppa022573mg	NCBI_Assembly:GCF_000346465.1	NW_006760385.1	+	26726666	26727094	428	-	-	-	-	
Pp06_35148-3p	18791662	PRUPE_ppa026406mg	NCBI_Assembly:GCF_000346465.1	NW_006760385.1	-	30792439	30793403	964	-	GO:0055114:oxidation-reduction process	-	GO:0016021:integral component of membrane	
Pp06_35148-3p	18791819	PRUPE_ppa013246mg	NCBI_Assembly:GCF_000346465.1	NW_006760385.1	+	3528255	3528845	590	-	-	-	-	
Pp06_35148-3p	18791898	PRUPE_ppa008292mg	NCBI_Assembly:GCF_000346465.1	NW_006760385.1	-	22630522	22633554	3032	-	-	GO:0003824:catalytic activity	GO:0009507:chloroplast	
Pp06_35148-3p	18791925	PRUPE_ppa006432mg	NCBI_Assembly:GCF_000346465.1	NW_006760385.1	+	36013297	36014535	1238	-	-	GO:0003700:sequence-specific DNA binding transcription factor activity;GO:0046872:metal ion binding	-	
Pp06_35148-3p	18791968	PRUPE_ppa017353mg	NCBI_Assembly:GCF_000346465.1	NW_006760385.1	-	42268	42585	317	pper04075:Plant hormone signal transduction	-	-	-	

Pp06_35148-3p	18792015	PRUPE_ppa004440mg	NCBI_Assembly:GCF_000346465.1	NW_006760385.1	+		37953369	37955139	1770	pper03015:mRNA surveillance pathway	GO:0007165:signal transduction	GO:0008601:protein phosphatase type 2A regulator activity	GO:0000159:protein phosphatase type 2A complex
Pp06_35148-3p	18792021	PRUPE_ppa012264mg	NCBI_Assembly:GCF_000346465.1	NW_006760385.1	-		4766312	4767019	707	-	-	-	-
Pp06_35148-3p	18792061	PRUPE_ppa008310mg	NCBI_Assembly:GCF_000346465.1	NW_006760385.1	-		8489559	8493197	3638	pper00904:Diterpenoid biosynthesis	GO:0045487:gibberellin catabolic process	GO:0005506:iron ion binding;GO:0052634:C-19 gibberellin 2-beta-dioxygenase activity	GO:0016020:membrane
Pp06_35148-3p	18792152	PRUPE_ppa017530mg	NCBI_Assembly:GCF_000346465.1	NW_006760385.1	-		25003930	25004448	518	-	GO:0016226:iron-sulfur cluster assembly;GO:0051176:positive regulation of sulfur metabolic process	GO:0008047:enzyme activator activity	-
Pp06_35148-3p	18792219	PRUPE_ppa018441mg	NCBI_Assembly:GCF_000346465.1	NW_006760385.1	+		2466822	2469165	2343	-	-	GO:0001104:RNA polymerase II transcription cofactor activity	GO:0016592:mediator complex
Pp06_35148-3p	18792254	PRUPE_ppa011849mg	NCBI_Assembly:GCF_000346465.1	NW_006760385.1	+		9172208	9173741	1533	pper04141:Protein processing in endoplasmic reticulum	GO:0006886:intracellular protein transport;GO:0016192:vesicle-mediated transport	GO:0005525:GTP binding	GO:0005783:endoplasmic reticulum;GO:0005794:Golgi apparatus
Pp06_35148-3p	18792308	PRUPE_ppa018985mg	NCBI_Assembly:GCF_000346465.1	NW_006760385.1	-		14359514	14361874	2360	-	-	-	-

Pp06_35148-3p	18792372	PRUPE_ppa009924mg	NCBI_Assembly:GCF_000346465.1	NW_006760385.1	+	35605526	35606674	1148	-	-	GO:0046872: metal ion binding	-
Pp06_35148-3p	18792437	PRUPE_ppa019854mg	NCBI_Assembly:GCF_000346465.1	NW_006760385.1	+	41230670	41232079	1409	-	-	-	-
Pp06_35148-3p	18792726	PRUPE_ppb012608mg	NCBI_Assembly:GCF_000346465.1	NW_006760385.1	-	19010704	19011854	1150	-	-	-	-
Pp06_35148-5p	18766145	PRUPE_ppa018777m1g	NCBI_Assembly:GCF_000346465.1	NW_006760190.1	-	2132	2670	538	-	GO:0005975: carbohydrate metabolic process	GO:0004553: hydrolase activity, hydrolyzing O-glycosyl compounds	-
Pp06_35148-5p	18766243	PRUPE_ppa015782mg	NCBI_Assembly:GCF_000346465.1	NW_006760194.1	+	8079782	8080078	296	-	-	-	-
Pp06_35148-5p	18766304	PRUPE_ppa016892mg	NCBI_Assembly:GCF_000346465.1	NW_006760194.1	-	3086567	3088329	1762	-	-	-	-
Pp06_35148-5p	18766370	PRUPE_ppa001025mg	NCBI_Assembly:GCF_000346465.1	NW_006760194.1	+	12131307	12137485	6178	-	-	GO:0004672: protein kinase activity;GO:0005524:ATP binding	-
Pp06_35148-5p	18766599	PRUPE_ppa004856mg	NCBI_Assembly:GCF_000346465.1	NW_006760194.1	+	20592749	20594753	2004	-	-	GO:0004190: aspartic-type endopeptidase activity	-
Pp06_35148-5p	18766684	PRUPE_ppa000736mg	NCBI_Assembly:GCF_000346465.1	NW_006760194.1	-	17890327	17895712	5385	-	GO:0006629: lipid metabolic process	GO:0016787: hydrolase activity	-
Pp06_35148-5p	18766840	PRUPE_ppa024752mg	NCBI_Assembly:GCF_000346465.1	NW_006760194.1	-	18009290	18010490	1200	-	-	-	-

Pp06_35148-5p	18766855	PRUPE_ppa007545mg	NCBI_Assembly:GCF_000346465.1	NW_006760194.1	+	621011	623017	2006	-	-	GO:0005506:iron ion binding;GO:0016705:oxidoreductase activity, acting on paired donors, with incorporation or reduction of molecular oxygen;GO:0020037:heme binding	-
Pp06_35148-5p	18767016	PRUPE_ppa013296mg	NCBI_Assembly:GCF_000346465.1	NW_006760194.1	+	16151193	16151637	444	-	-	-	-
Pp06_35148-5p	18767041	PRUPE_ppa001587mg	NCBI_Assembly:GCF_000346465.1	NW_006760194.1	-	17083157	17087372	4215	-	-	-	-
Pp06_35148-5p	18767083	PRUPE_ppa007191mg	NCBI_Assembly:GCF_000346465.1	NW_006760194.1	-	176367	180463	4096	-	-	-	-
Pp06_35148-5p	18767267	PRUPE_ppa008703mg	NCBI_Assembly:GCF_000346465.1	NW_006760194.1	-	19413161	19415412	2251	-	-	-	-
Pp06_35148-5p	18767304	PRUPE_ppa003483mg	NCBI_Assembly:GCF_000346465.1	NW_006760194.1	-	16128809	16132727	3918	-	-	GO:0033926:glycopeptide alpha-N-acetylgalactosaminidase activity	-
Pp06_35148-5p	18767345	PRUPE_ppa000182mg	NCBI_Assembly:GCF_000346465.1	NW_006760194.1	+	21256059	21262798	6739	-	-	GO:0005524:ATP binding;GO:0042626:ATPase activity, coupled to transmembrane movement of substances	GO:0016021:integral component of membrane

Pp06_35148-5p	18767454	PRUPE_ppa014671mg	NCBI_Assembly:GCF_000346465.1	NW_006760194.1	-	16634917	16635768	851	-	-	-	-
Pp06_35148-5p	18767473	PRUPE_ppa006130mg	NCBI_Assembly:GCF_000346465.1	NW_006760194.1	+	21482868	21485119	2251	-	GO:0005975:carbohydrate metabolic process;GO:0071555:cell wall organization	GO:0004650:polygalacturonase activity	GO:0005576:extracellular region
Pp06_35148-5p	18767601	PRUPE_ppa026016mg	NCBI_Assembly:GCF_000346465.1	NW_006760194.1	+	19046658	19047960	1302	-	-	-	-
Pp06_35148-5p	18767681	PRUPE_ppa008903mg	NCBI_Assembly:GCF_000346465.1	NW_006760194.1	+	8002291	8005779	3488	pper00910:Nitrogen metabolism	GO:0015976:carbon utilization;GO:0019243:methylglyoxal catabolic process to D-lactate	GO:0004089:carbonate dehydratase activity;GO:0008270:zinc ion binding	GO:0009507:chloroplast
Pp06_35148-5p	18767719	PRUPE_ppa009885mg	NCBI_Assembly:GCF_000346465.1	NW_006760194.1	+	17274610	17276690	2080	-	-	-	-
Pp06_35148-5p	18769336	PRUPE_ppa011563mg	NCBI_Assembly:GCF_000346465.1	NW_006760201.1	-	18128627	18130029	1402	-	-	GO:0003700:sequence-specific DNA binding transcription factor activity	-
Pp06_35148-5p	18769405	PRUPE_ppa008871mg	NCBI_Assembly:GCF_000346465.1	NW_006760201.1	+	19733009	19735577	2568	-	-	GO:0008168:methyltransferase activity	GO:0009507:chloroplast

Pp06_35148-5p	18769453	PRUPE_ppa016580mg	NCBI_Assembly:GCF_000346465.1	NW_006760201.1	+	4615077	4617310	2233	-	GO:0001708:cell fate specification;GO:0003333:amino acid transmembrane transport;GO:0006863:purine nucleobase transport	-	-
Pp06_35148-5p	18769566	PRUPE_ppa002380mg	NCBI_Assembly:GCF_000346465.1	NW_006760201.1	-	19768721	19772416	3695	-	-	GO:0004672:protein kinase activity;GO:0005524:ATP binding	-
Pp06_35148-5p	18769577	PRUPE_ppa000925mg	NCBI_Assembly:GCF_000346465.1	NW_006760201.1	-	18740357	18746048	5691	-	GO:0007018:microtubule-based movement;GO:0007112:male meiosis cytokinesis;GO:0009555:pollen development;GO:0009558:embryo sac cellularization;GO:0010245:radial microtubular system formation	GO:0003777:microtubule motor activity;GO:0005524:ATP binding	GO:0005871:kinesin complex;GO:0005874:microtubule;GO:0009506:plasmodesma
Pp06_35148-5p	18769612	PRUPE_ppa011341mg	NCBI_Assembly:GCF_000346465.1	NW_006760201.1	-	12537040	12538931	1891	pper03010:Ribosome	GO:0006412:translation	GO:0003723:RNA binding;GO:0003735:structural constituent of ribosome	GO:0015935:small ribosomal subunit

Pp06_35148-5p	18769720	PRUPE_ppa019447mg	NCBI_Assembly:GCF_000346465.1	NW_006760201.1	-	8016461	8017339	878	-	-	-	-
Pp06_35148-5p	18769968	PRUPE_ppa003792mg	NCBI_Assembly:GCF_000346465.1	NW_006760201.1	+	21873334	21876962	3628	-	-	-	-
Pp06_35148-5p	18770229	PRUPE_ppb013740mg	NCBI_Assembly:GCF_000346465.1	NW_006760201.1	+	18910103	18912484	2381	pper00196:Photosynthesis - antenna proteins	GO:0009765:photosynthesis, light harvesting;GO:0018298:protein-chromophore linkage	GO:0016168:chlorophyll binding	GO:0009507:chloroplast;GO:0009523:photosystem II;GO:0016021:integral component of membrane
Pp06_35148-5p	18770516	PRUPE_ppa003667mg	NCBI_Assembly:GCF_000346465.1	NW_006760201.1	+	3266368	3272645	6277	-	GO:0009658:chloroplast organization;GO:0009959:negative gravitropism;GO:0010027:thylakoid membrane organization;GO:0010207:photosystem II assembly;GO:0043157:response to cation stress;GO:0048564:photosystem I assembly;GO:0060359:response to ammonium ion	GO:0004222:metalloendopeptidase activity	GO:0009507:chloroplast

Pp06_35148-5p	18770615	PRUPE_ppa000677mg	NCBI_Assembly:GCF_000346465.1	NW_006760201.1	-	12171016	12180434	9418	-	GO:0007018:microtubule-based movement	GO:0003777:microtubule motor activity;GO:0005524:ATP binding	GO:0005871:kinesin complex;GO:0005874:microtubule
Pp06_35148-5p	18770624	PRUPE_ppa023912mg	NCBI_Assembly:GCF_000346465.1	NW_006760201.1	-	1577847	1580384	2537	-	-	-	-
Pp06_35148-5p	18770768	PRUPE_ppa023266mg	NCBI_Assembly:GCF_000346465.1	NW_006760201.1	+	21031578	21034684	3106	-	-	GO:0004672:protein kinase activity;GO:0005524:ATP binding	-
Pp06_35148-5p	18771015	PRUPE_ppa014930mg	NCBI_Assembly:GCF_000346465.1	NW_006760201.1	+	16852836	16853936	1100	-	GO:0009790:embryo development;GO:0009909:regulation of flower development;GO:0048446:petal morphogenesis	GO:0003700:sequence-specific DNA binding transcription factor activity;GO:0008270:zinc ion binding;GO:0043565:sequence-specific DNA binding	-
Pp06_35148-5p	18771072	PRUPE_ppa015675mg	NCBI_Assembly:GCF_000346465.1	NW_006760201.1	-	7068372	7069291	919	-	-	-	-
Pp06_35148-5p	18771130	PRUPE_ppa020995mg	NCBI_Assembly:GCF_000346465.1	NW_006760201.1	-	9188651	9193252	4601	-	-	-	-
Pp06_35148-5p	18771265	PRUPE_ppa026426mg	NCBI_Assembly:GCF_000346465.1	NW_006760201.1	-	18399486	18402286	2800	-	-	GO:0022891:substrate-specific transmembrane transporter activity	GO:0016021:integral component of membrane

Pp06_35148-5p	18771796	PRUPE_ppa019594mg	NCBI_Assembly:GCF_000346465.1	NW_006760201.1	+		21342805	21343371	566	-	-	GO:0009055:electron carrier activity	-
Pp06_35148-5p	18771814	PRUPE_ppa011109mg	NCBI_Assembly:GCF_000346465.1	NW_006760201.1	-		22207952	22209916	1964	-	GO:0042023:DNA endoreduplication;GO:0043161:proteasome-mediated ubiquitin-dependent protein catabolic process;GO:0043248:proteasome assembly;GO:0051510:regulation of unidimensional cell growth;GO:0051788:response to misfolded protein	-	-
Pp06_35148-5p	18772106	PRUPE_ppa011216mg	NCBI_Assembly:GCF_000346465.1	NW_006760208.1	-		23676923	23679563	2640	-	-	GO:0008270:zinc ion binding	-
Pp06_35148-5p	18772204	PRUPE_ppa022075mg	NCBI_Assembly:GCF_000346465.1	NW_006760208.1	+		23449816	23451769	1953	-	-	GO:0005215:transporter activity	GO:0016020:membrane
Pp06_35148-5p	18772328	PRUPE_ppa001078mg	NCBI_Assembly:GCF_000346465.1	NW_006760208.1	+		11224654	11231075	6421	pper03022:Basal transcription factors	GO:0006352:DNA-templated transcription, initiation	-	GO:0005669:transcription factor TFIID complex

Pp06_35148-5p	18772636	PRUPE_ppa003308mg	NCBI_Assembly:GCF_000346465.1	NW_006760208.1	-	23146966	23149384	2418	-	GO:0046274: lignin catabolic process	GO:0005507: copper ion binding;GO:0052716:hydroquinone:oxygen oxidoreductase activity	GO:0048046: apoplast
Pp06_35148-5p	18772668	PRUPE_ppa015441mg	NCBI_Assembly:GCF_000346465.1	NW_006760208.1	-	24571511	24574140	2629	-	GO:0008283: cell proliferation	GO:0004672: protein kinase activity;GO:0005524:ATP binding	GO:0009505: plant-type cell wall;GO:0009506:plasmodesma;GO:0016021:integral component of membrane
Pp06_35148-5p	18772711	PRUPE_ppa002678mg	NCBI_Assembly:GCF_000346465.1	NW_006760208.1	-	25979044	25982463	3419	-	-	-	-
Pp06_35148-5p	18772723	PRUPE_ppa013461mg	NCBI_Assembly:GCF_000346465.1	NW_006760208.1	-	26532608	26533335	727	-	-	-	-
Pp06_35148-5p	18772819	PRUPE_ppa018534mg	NCBI_Assembly:GCF_000346465.1	NW_006760208.1	-	18257643	18258330	687	-	-	-	-
Pp06_35148-5p	18772893	PRUPE_ppa004677mg	NCBI_Assembly:GCF_000346465.1	NW_006760208.1	-	10146412	10150861	4449	-	-	GO:0000166: nucleotide binding;GO:0003676:nucleic acid binding	-
Pp06_35148-5p	18772923	PRUPE_ppa011138mg	NCBI_Assembly:GCF_000346465.1	NW_006760208.1	-	28514318	28516844	2526	-	-	GO:0016787: hydrolase activity	GO:0009507: chloroplast
Pp06_35148-5p	18774539	PRUPE_ppa025423mg	NCBI_Assembly:GCF_000346465.1	NW_006760208.1	+	1650389	1652822	2433	-	GO:0042138: meiotic DNA double-strand break formation	-	-
Pp06_35148-5p	18775805	PRUPE_ppa015669mg	NCBI_Assembly:GCF_000346465.1	NW_006760212.1	+	11359439	11360035	596	-	-	-	-

Pp06_35148-5p	18775816	PRUPE_ppb016228mg	NCBI_Assembly:GCF_000346465.1	NW_006760212.1	-	14779730	14783081	3351	-	-	-	-
Pp06_35148-5p	18775851	PRUPE_ppa017201mg	NCBI_Assembly:GCF_000346465.1	NW_006760212.1	-	8533950	8534510	560	-	-	-	-
Pp06_35148-5p	18775908	PRUPE_ppa012406mg	NCBI_Assembly:GCF_000346465.1	NW_006760212.1	-	4958735	4960348	1613	-	-	-	-
Pp06_35148-5p	18775910	PRUPE_ppa006016mg	NCBI_Assembly:GCF_000346465.1	NW_006760212.1	-	18126802	18130939	4137	-	-	GO:0003824:catalytic activity	-
Pp06_35148-5p	18775972	PRUPE_ppa004942mg	NCBI_Assembly:GCF_000346465.1	NW_006760212.1	+	14357685	14359153	1468	-	-	GO:0003746:translation elongation factor activity;GO:0003924:GTPase activity;GO:0005525:GTP binding	GO:0005622:intracellular
Pp06_35148-5p	18776089	PRUPE_ppa013381mg	NCBI_Assembly:GCF_000346465.1	NW_006760212.1	+	18212861	18214562	1701	-	-	-	-

Pp06_35148-5p	18776112	PRUPE_ppa007415mg	NCBI_Assembly:GCF_000346465.1	NW_006760212.1	-	12189050	12191284	2234	-	GO:0000165:MAPK cascade;GO:0006499:N-terminal protein myristoylation;GO:0006612:protein targeting to membrane;GO:0009409:response to cold;GO:0009738:abscisic acid-activated signaling pathway;GO:0009862:systemic acquired resistance, salicylic acid mediated signaling pathway;GO:0009867:jasmonic acid mediated signaling pathway;GO:0010363:regulation of plant-type hypersensitive response;GO:0030968:en [illegible]	-	GO:0005622:intracellular
Pp06_35148-5p	18776116	PRUPE_ppa005002mg	NCBI_Assembly:GCF_000346465.1	NW_006760212.1	-	17416411	17419943	3532	-	GO:0015996:chlorophyll catabolic process	-	-
Pp06_35148-5p	18777285	PRUPE_ppa000575m1g	NCBI_Assembly:GCF_000346465.1	NW_006760212.1	-	2103076	2105374	2298	-	-	-	-

Pp06_35148-5p	18777449	PRUPE_ppa010725mg	NCBI_Assembly:GCF_000346465.1	NW_006760212.1	+	12059217	12062126	2909	pper01100:Metabolic pathways;pper01110:Biosynthesis of secondary metabolites;pper00230:Purine metabolism;pper00240:Pyrimidine metabolism	GO:0006183:GTP biosynthetic process;GO:0006228:UTP biosynthetic process;GO:0006241:CTP biosynthetic process	GO:0004550:nucleoside diphosphate kinase activity;GO:0005524:ATP binding	-
Pp06_35148-5p	18777464	PRUPE_ppa021779mg	NCBI_Assembly:GCF_000346465.1	NW_006760212.1	-	18105895	18107105	1210	-	-	GO:0003677:DNA binding;GO:0003682:chromatin binding	-
Pp06_35148-5p	18777773	PRUPE_ppa024005mg	NCBI_Assembly:GCF_000346465.1	NW_006760212.1	-	12008447	12009849	1402	-	-	GO:0008270:zinc ion binding;GO:0030247:polysaccharide binding	-
Pp06_35148-5p	18777846	PRUPE_ppa012578mg	NCBI_Assembly:GCF_000346465.1	NW_006760212.1	-	17237581	17239428	1847	-	-	-	-
Pp06_35148-5p	18777878	PRUPE_ppa013330mg	NCBI_Assembly:GCF_000346465.1	NW_006760212.1	-	5027311	5030261	2950	-	-	-	-
Pp06_35148-5p	18777904	PRUPE_ppa017924mg	NCBI_Assembly:GCF_000346465.1	NW_006760212.1	+	519684	522366	2682	-	GO:0055085:transmembrane transport	-	GO:0016021:integral component of membrane
Pp06_35148-5p	18778028	PRUPE_ppa017459mg	NCBI_Assembly:GCF_000346465.1	NW_006760212.1	-	1534080	1535834	1754	-	-	-	-
Pp06_35148-5p	18778109	PRUPE_ppa011465mg	NCBI_Assembly:GCF_000346465.1	NW_006760212.1	+	13239174	13241913	2739	-	-	-	-
Pp06_35148-5p	18778191	PRUPE_ppb022709mg	NCBI_Assembly:GCF_000346465.1	NW_006760220.1	-	19446200	19447585	1385	-	-	-	-

Pp06_35148-5p	18778209	PRUPE_ppa016488mg	NCBI_Assembly:GCF_000346465.1	NW_006760220.1	-	9759834	9761654	1820	-	-	GO:0005524:ATP binding;GO:0016887:ATPase activity	GO:0016020:membrane
Pp06_35148-5p	18778409	PRUPE_ppa022123mg	NCBI_Assembly:GCF_000346465.1	NW_006760220.1	+	16835842	16837329	1487	-	GO:0006952:defense response	-	-
Pp06_35148-5p	18778455	PRUPE_ppa024787mg	NCBI_Assembly:GCF_000346465.1	NW_006760220.1	-	9512444	9513682	1238	-	-	-	-
Pp06_35148-5p	18778539	PRUPE_ppa006876mg	NCBI_Assembly:GCF_000346465.1	NW_006760220.1	+	1115783	1117251	1468	-	-	-	-
Pp06_35148-5p	18778586	PRUPE_ppa012683mg	NCBI_Assembly:GCF_000346465.1	NW_006760220.1	+	29669470	29670079	609	-	-	-	-
Pp06_35148-5p	18778631	PRUPE_ppa008430mg	NCBI_Assembly:GCF_000346465.1	NW_006760220.1	+	10868817	10871787	2970	-	GO:0008284:positive regulation of cell proliferation;GO:0009560:embryo sac egg cell differentiation	-	-
Pp06_35148-5p	18778657	PRUPE_ppa020491mg	NCBI_Assembly:GCF_000346465.1	NW_006760220.1	-	21821993	21822226	233	-	-	-	-
Pp06_35148-5p	18778669	PRUPE_ppa001630mg	NCBI_Assembly:GCF_000346465.1	NW_006760220.1	-	5179025	5182292	3267	-	-	-	-
Pp06_35148-5p	18778746	PRUPE_ppa003152mg	NCBI_Assembly:GCF_000346465.1	NW_006760220.1	+	1084954	1086867	1913	-	-	GO:0046872:metal ion binding	-
Pp06_35148-5p	18778750	PRUPE_ppb020871mg	NCBI_Assembly:GCF_000346465.1	NW_006760220.1	-	1984327	1986116	1789	-	-	GO:0004097:catechol oxidase activity	-
Pp06_35148-5p	18778797	PRUPE_ppa015242mg	NCBI_Assembly:GCF_000346465.1	NW_006760220.1	+	25697728	25701959	4231	-	-	-	-

Pp06_35148-5p	18778804	PRUPE_ppb018776mg	NCBI_Assembly:GCF_000346465.1	NW_006760220.1	+		26449832	26453050	3218	-	-	-	-
Pp06_35148-5p	18778940	PRUPE_ppa025399mg	NCBI_Assembly:GCF_000346465.1	NW_006760220.1	-		1427653	1429526	1873	-	GO:0046777: protein autophosphorylation	-	-
Pp06_35148-5p	18778958	PRUPE_ppa009833mg	NCBI_Assembly:GCF_000346465.1	NW_006760220.1	-		29837419	29838351	932	-	-	GO:0003677: DNA binding	-
Pp06_35148-5p	18779074	PRUPE_ppa021126mg	NCBI_Assembly:GCF_000346465.1	NW_006760220.1	+		20646683	20647900	1217	-	-	-	-
Pp06_35148-5p	18779076	PRUPE_ppa000652mg	NCBI_Assembly:GCF_000346465.1	NW_006760220.1	-		10506345	10509503	3158	-	GO:0009611: response to wounding	GO:0001653: peptide receptor activity;GO:0004674:protein serine/threonine kinase activity;GO:0005524:ATP binding	GO:0009507: chloroplast;GO:0016021:integral component of membrane
Pp06_35148-5p	18779102	PRUPE_ppa007390mg	NCBI_Assembly:GCF_000346465.1	NW_006760220.1	+		8454390	8458372	3982	pper01100:Metabolic pathways;pper01110:Biosynthesis of secondary metabolites;pper00630:Glyoxylate and dicarboxylate metabolism;pper04146:Peroxisome	-	GO:0010181: FMN binding;GO:0016491:oxidoreductase activity	-

Pp06_35148-5p	18779227	PRUPE_ppa002050mg	NCBI_Assembly:GCF_000346465.1	NW_006760220.1	+	4251478	4255583	4105	-	-	-	GO:0005774:vacuolar membrane;GO:0005886:plasma membrane;GO:0009506:plasmodesma;GO:0009941:chloroplast envelope
Pp06_35148-5p	18779268	PRUPE_ppa015864mg	NCBI_Assembly:GCF_000346465.1	NW_006760220.1	-	10807821	10808087	266	-	-	GO:0008270:zinc ion binding	-
Pp06_35148-5p	18779417	PRUPE_ppa017824mg	NCBI_Assembly:GCF_000346465.1	NW_006760220.1	-	16113499	16114533	1034	-	-	GO:0030247:polysaccharide binding	-
Pp06_35148-5p	18779533	PRUPE_ppa014502mg	NCBI_Assembly:GCF_000346465.1	NW_006760220.1	+	30192609	30193116	507	-	GO:0005975:carbohydrate metabolic process	GO:0004650:polygalacturonase activity	-
Pp06_35148-5p	18779554	PRUPE_ppa004781mg	NCBI_Assembly:GCF_000346465.1	NW_006760220.1	-	1713119	1716755	3636	pper01100:Metabolic pathways;pper00600:Sphingolipid metabolism	-	GO:0016301:kinase activity	-
Pp06_35148-5p	18779587	PRUPE_ppa008862mg	NCBI_Assembly:GCF_000346465.1	NW_006760220.1	+	20176779	20178611	1832	-	-	-	-
Pp06_35148-5p	18779618	PRUPE_ppa012633mg	NCBI_Assembly:GCF_000346465.1	NW_006760220.1	-	1425797	1426706	909	-	-	-	-
Pp06_35148-5p	18779657	PRUPE_ppa1027208mg	NCBI_Assembly:GCF_000346465.1	NW_006760220.1	-	9098602	9099588	986	-	-	-	-

Pp06_35148-5p	18779688	PRUPE_ppa009647mg	NCBI_Assembly:GCF_000346465.1	NW_006760220.1	+	3206039	3207150	1111	-	-	GO:0003700:sequence-specific DNA binding transcription factor activity;GO:0043565:sequence-specific DNA binding	-
Pp06_35148-5p	18779771	PRUPE_ppa002854mg	NCBI_Assembly:GCF_000346465.1	NW_006760220.1	-	29706204	29713908	7704	-	GO:0006397:mRNA processing	GO:0000166:nucleotide binding;GO:0003723:RNA binding	GO:0005634:nucleus
Pp06_35148-5p	18779779	PRUPE_ppa015386mg	NCBI_Assembly:GCF_000346465.1	NW_006760220.1	+	21790612	21793289	2677	-	-	-	-
Pp06_35148-5p	18779886	PRUPE_ppa016075mg	NCBI_Assembly:GCF_000346465.1	NW_006760220.1	-	26914610	26915104	494	-	-	GO:0003676:nucleic acid binding	-
Pp06_35148-5p	18780036	PRUPE_ppa019856mg	NCBI_Assembly:GCF_000346465.1	NW_006760220.1	+	6849167	6851442	2275	-	-	GO:0000287:magnesium ion binding;GO:0010333:terpene synthase activity	-

Pp06_35148-5p	18780066	PRUPE_ppa001879mg	NCBI_Assembly:GCF_000346465.1	NW_006760220.1	+	18668341	18672897	4556	pper01100:Metabolic pathways;pper00920:Sulfur metabolism	GO:0006096:glycolytic process;GO:0006275:regulation of DNA replication;GO:0006323:DNA packaging;GO:0006833:water transport;GO:0006972:hyperosmotic response;GO:0007030:Golgi organization;GO:0009266:response to temperature stimulus;GO:0009684:indoleacetic acid biosynthetic process;GO:0016126:sterol biosynthetic process;GO:0019344:cysteine biosynthetic process;GO:0019424:sulfide oxidation, using	GO:0003677:DNA binding;GO:0005507:copper ion binding;GO:0016002:sulfite reductase activity;GO:0020037:heme binding;GO:0050311:sulfite reductase (ferredoxin) activity;GO:0051539:4 iron, 4 sulfur cluster binding	GO:0009941:chloroplast envelope;GO:0010319:stromule;GO:0016020:membrane;GO:0042644:chloroplast nucleoid;GO:0048046:apoplast
Pp06_35148-5p	18780119	PRUPE_ppa018175mg	NCBI_Assembly:GCF_000346465.1	NW_006760220.1	-	9218009	9218959	950	-	-	-	-
Pp06_35148-5p	18780121	PRUPE_ppa025658mg	NCBI_Assembly:GCF_000346465.1	NW_006760220.1	+	16272145	16273285	1140	-	-	GO:0008234:cysteine-type peptidase activity	-

Pp06_35148-5p	18780409	PRUPE_ppa004663mg	NCBI_Assembly:GCF_000346465.1	NW_006760220.1	-	5310211	5316857	6646	pper00230:Purine metabolism	-	GO:0016462:pyrophosphatase activity	GO:0005737:cytoplasm
Pp06_35148-5p	18780425	PRUPE_ppa009777mg	NCBI_Assembly:GCF_000346465.1	NW_006760220.1	+	2167459	2168977	1518	-	-	-	-
Pp06_35148-5p	18780436	PRUPE_ppa022607mg	NCBI_Assembly:GCF_000346465.1	NW_006760220.1	-	3638693	3641636	2943	-	GO:0006813:potassium ion transport;GO:0006814:sodium ion transport	GO:0005451:monovalent cation:proton antiporter activity	GO:0016021:integral component of membrane
Pp06_35148-5p	18780537	PRUPE_ppa004984m1g	NCBI_Assembly:GCF_000346465.1	NW_006760220.1	+	5272987	5273288	301	-	-	GO:0004497:monooxygenase activity;GO:0005506:iron ion binding;GO:0016705:oxidoreductase activity, acting on paired donors, with incorporation or reduction of molecular oxygen;GO:0020037:heme binding	-

Pp06_35148-5p	18780545	PRUPE_ppa006368mg	NCBI_Assembly:GCF_000346465.1	NW_006760220.1	-	10306823	10312636	5813	pper00130:Ubiquinone and other terpenoid-quinone biosynthesis;pper00270:Cysteine and methionine metabolism;pper00350:Tyrosine metabolism;pper00360:Phenylalanine metabolism;pper00400:Phenylalanine, tyrosine and tryptophan biosynthesis;pper00950:Isoquinoline alkaloid biosynthesis;pper00960:Tropane, piperidine and pyridine alkaloid biosynthesis;pper01100:Metabolic pathways;pper01110:Biosynthesis of secondary metabolites;pper01230:Bio	GO:0006520:cellular amino acid metabolic process;GO:0009058:biosynthetic process	GO:0004838:L-tyrosine:2-oxoglutarate aminotransferase activity;GO:0030170:pyridoxal phosphate binding	-
Pp06_35148-5p	18781497	PRUPE_ppa021519mg	NCBI_Assembly:GCF_000346465.1	NW_006760220.1	-	7021375	7023074	1699	-	-	GO:0022857:transmembrane transporter activity	GO:0016021:integral component of membrane

Pp06_35148-5p	18781600	PRUPE_ppa003326mg	NCBI_Assembly:GCF_000346465.1	NW_006760268.1	-	12971901	12974091	2190	-	GO:0009553:embryo sac development;GO:0009555:pollen development;GO:0009790:embryo development	-	GO:0005887:integral component of plasma membrane
Pp06_35148-5p	18782725	PRUPE_ppa015833mg	NCBI_Assembly:GCF_000346465.1	NW_006760268.1	-	2130011	2133106	3095	-	GO:0006351:transcription, DNA-templated;GO:0009686:gibberellin biosynthetic process	GO:0003677:DNA binding;GO:0003700:sequence-specific DNA binding transcription factor activity	GO:0005634:nucleus
Pp06_35148-5p	18783065	PRUPE_ppa017033mg	NCBI_Assembly:GCF_000346465.1	NW_006760268.1	-	20682844	20683494	650	-	GO:0009236:cobalamin biosynthetic process	GO:0016852:sirohydrochlorin cobaltochelatase activity	-
Pp06_35148-5p	18783328	PRUPE_ppa017871mg	NCBI_Assembly:GCF_000346465.1	NW_006760268.1	-	13334843	13338189	3346	-	-	GO:0004674:protein serine/threonine kinase activity;GO:0005524:ATP binding	GO:0016021:integral component of membrane
Pp06_35148-5p	18783341	PRUPE_ppb022980m2g	NCBI_Assembly:GCF_000346465.1	NW_006760268.1	+	3085047	3086179	1132	-	-	GO:0004672:protein kinase activity;GO:0005524:ATP binding	-
Pp06_35148-5p	18783496	PRUPE_ppa001625mg	NCBI_Assembly:GCF_000346465.1	NW_006760268.1	+	9190015	9202627	12612	-	-	GO:0004674:protein serine/threonine kinase activity;GO:0005524:ATP binding	-

Pp06_35148-5p	18783576	PRUPE_ppa024438mg	NCBI_Assembly:GCF_000346465.1	NW_006760268.1	-	17268726	17272280	3554	-	GO:0048544: recognition of pollen	GO:0004672: protein kinase activity;GO:0005524:ATP binding	-
Pp06_35148-5p	18783691	PRUPE_ppa002680mg	NCBI_Assembly:GCF_000346465.1	NW_006760268.1	+	4268606	4271122	2516	-	-	-	-
Pp06_35148-5p	18783699	PRUPE_ppa001190mg	NCBI_Assembly:GCF_000346465.1	NW_006760268.1	+	18774840	18777497	2657	-	-	GO:0004674: protein serine/threonine kinase activity;GO:0005524:ATP binding	-
Pp06_35148-5p	18783780	PRUPE_ppa016307mg	NCBI_Assembly:GCF_000346465.1	NW_006760268.1	+	20526727	20532543	5816	-	-	GO:0004672: protein kinase activity;GO:0005524:ATP binding	GO:0005886: plasma membrane
Pp06_35148-5p	18783853	PRUPE_ppb009532mg	NCBI_Assembly:GCF_000346465.1	NW_006760268.1	-	19510291	19512202	1911	-	GO:0006810: transport	-	GO:0016021: integral component of membrane
Pp06_35148-5p	18783872	PRUPE_ppa025740mg	NCBI_Assembly:GCF_000346465.1	NW_006760268.1	-	5651819	5654451	2632	-	-	-	-
Pp06_35148-5p	18783998	PRUPE_ppa019779mg	NCBI_Assembly:GCF_000346465.1	NW_006760268.1	-	21671796	21674351	2555	-	-	-	GO:0005773: vacuole
Pp06_35148-5p	18784204	PRUPE_ppa010970mg	NCBI_Assembly:GCF_000346465.1	NW_006760268.1	+	15939449	15942399	2950	-	-	GO:0005094: Rho GDP-dissociation inhibitor activity	GO:0005737: cytoplasm

Pp06_35148-5p	18784457	PRUPE_ppa006945mg	NCBI_Assembly:GCF_000346465.1	NW_006760268.1	+	6616397	6621583	5186	pper01100:Metabolic pathways;pper01110:Biosynthesis of secondary metabolites;pper00940:Phenylpropanoid biosynthesis	-	GO:0003824:catalytic activity;GO:0050662:coenzyme binding	-
Pp06_35148-5p	18784547	PRUPE_ppa025200mg	NCBI_Assembly:GCF_000346465.1	NW_006760322.1	-	1025	3032	2007	-	-	-	-
Pp06_35148-5p	18784583	PRUPE_ppa000661mg	NCBI_Assembly:GCF_000346465.1	NW_006760324.1	+	23775166	23780731	5565	-	-	-	-
Pp06_35148-5p	18784600	PRUPE_ppb013270mg	NCBI_Assembly:GCF_000346465.1	NW_006760324.1	+	23096613	23097213	600	-	-	-	-
Pp06_35148-5p	18784644	PRUPE_ppa003612mg	NCBI_Assembly:GCF_000346465.1	NW_006760324.1	+	23538990	23545701	6711	-	-	-	-
Pp06_35148-5p	18784704	PRUPE_ppa027146mg	NCBI_Assembly:GCF_000346465.1	NW_006760324.1	-	2025468	2027319	1851	-	-	GO:0003676:nucleic acid binding	-
Pp06_35148-5p	18784730	PRUPE_ppa001259mg	NCBI_Assembly:GCF_000346465.1	NW_006760324.1	-	25956077	25960666	4589	pper03013:RNA transport	GO:0006606:protein import into nucleus;GO:0052541:plant-type cell wall cellulose metabolic process;GO:0052546:cell wall pectin metabolic process	-	GO:0005634:nucleus;GO:0009507:chloroplast

Pp06_35148-5p	18784774	PRUPE_ppa006173mg	NCBI_Assembly:GCF_000346465.1	NW_006760324.1	+	18939395	18944692	5297	-	GO:0006351:transcription, DNA-templated	GO:0003700:sequence-specific DNA binding transcription factor activity;GO:0043565:sequence-specific DNA binding	GO:0005634:nucleus
Pp06_35148-5p	18784837	PRUPE_ppa001918mg	NCBI_Assembly:GCF_000346465.1	NW_006760324.1	+	4277531	4285391	7860	-	-	GO:0004252:serine-type endopeptidase activity	-

Pp06_35148-5p	18784840	PRUPE_ppa022446mg	NCBI_Assembly:GCF_000346465.1	NW_006760324.1	+	24851595	24853223	1628	-	GO:0009089:lysine biosynthetic process via diaminopimelate;GO:0009773:photosynthetic electron transport in photosystem I;GO:0009965:leaf morphogenesis;GO:0010103:stomatal complex morphogenesis;GO:0010207:photosystem II assembly;GO:0016556:mRNA modification;GO:0030154:cell differentiation;GO:0042742:defense response to bacterium;GO:0045893:positive regulation of transcription, DNA-templated	GO:0008839:4-hydroxy-tetrahydrodipicolinate reductase	GO:0009570:chloroplast stroma	

Pp06_35148-5p	18784883	PRUPE_ppa000245mg	NCBI_Assembly:GCF_000346465.1	NW_006760324.1	+		21862980	21870834	7854	pper02010:ABC transporters	-	GO:0005524:ATP binding;GO:0042626:ATPase activity, coupled to transmembrane movement of substances	GO:0016021:integral component of membrane
Pp06_35148-5p	18784898	PRUPE_ppa006742mg	NCBI_Assembly:GCF_000346465.1	NW_006760324.1	-		17400633	17405055	4422	-	-	-	-
Pp06_35148-5p	18785025	PRUPE_ppa004195mg	NCBI_Assembly:GCF_000346465.1	NW_006760324.1	+		25058127	25061849	3722	pper01100:Metabolic pathways;pper00230:Purine metabolism;pper00240:Pyrimidine metabolism;pper03020:RNA polymerase	GO:0006351:transcription, DNA-templated	GO:0003677:DNA binding;GO:0003899:DNA-directed RNA polymerase activity	-
Pp06_35148-5p	18785081	PRUPE_ppa010386mg	NCBI_Assembly:GCF_000346465.1	NW_006760324.1	-		16158063	16160063	2000	-	-	-	-
Pp06_35148-5p	18785132	PRUPE_ppa014222mg	NCBI_Assembly:GCF_000346465.1	NW_006760324.1	-		23360274	23361102	828	-	GO:0030001:metal ion transport	GO:0046872:metal ion binding	-
Pp06_35148-5p	18785155	PRUPE_ppa022999mg	NCBI_Assembly:GCF_000346465.1	NW_006760324.1	-		25040873	25043118	2245	-	GO:0009560:embryo sac egg cell differentiation	GO:0003676:nucleic acid binding;GO:0008270:zinc ion binding	-

Pp06_35148-5p	18785161	PRUPE_ppa005481mg	NCBI_Assembly:GCF_000346465.1	NW_006760324.1	+	26486647	26491841	5194	-	GO:0000082:G1/S transition of mitotic cell cycle;GO:0006351:transcription, DNA-templated;GO:0010090:trichome morphogenesis;GO:0042023:DNA endoreduplication;GO:0051302:regulation of cell division	GO:0003677:DNA binding;GO:0003700:sequence-specific DNA binding transcription factor activity	GO:0005634:nucleus;GO:0005667:transcription factor complex
Pp06_35148-5p	18785228	PRUPE_ppa001348mg	NCBI_Assembly:GCF_000346465.1	NW_006760324.1	-	19342590	19348710	6120	-	GO:0006355:regulation of transcription, DNA-templated	GO:0008270:zinc ion binding	-
Pp06_35148-5p	18785464	PRUPE_ppa026643mg	NCBI_Assembly:GCF_000346465.1	NW_006760324.1	-	22341023	22341733	710	-	-	-	-
Pp06_35148-5p	18785516	PRUPE_ppb014702mg	NCBI_Assembly:GCF_000346465.1	NW_006760324.1	-	17278487	17280278	1791	-	-	-	-
Pp06_35148-5p	18785598	PRUPE_ppa009474mg	NCBI_Assembly:GCF_000346465.1	NW_006760324.1	+	23344843	23348605	3762	-	-	-	GO:0009507:chloroplast
Pp06_35148-5p	18785602	PRUPE_ppa000626mg	NCBI_Assembly:GCF_000346465.1	NW_006760324.1	+	22332874	22340481	7607	-	-	GO:0003723:RNA binding	-
Pp06_35148-5p	18785653	PRUPE_ppa015549mg	NCBI_Assembly:GCF_000346465.1	NW_006760324.1	-	18010712	18014945	4233	-	-	-	-

Pp06_35148-5p	18785870	PRUPE_ppa001671mg	NCBI_Assembly:GCF_000346465.1	NW_006760324.1	-	6830498	6836546	6048	-	-	GO:0004672:protein kinase activity;GO:0005524:ATP binding	-
Pp06_35148-5p	18786096	PRUPE_ppa005682mg	NCBI_Assembly:GCF_000346465.1	NW_006760324.1	+	19222859	19225449	2590	-	-	-	-
Pp06_35148-5p	18786116	PRUPE_ppa024105mg	NCBI_Assembly:GCF_000346465.1	NW_006760324.1	-	1813055	1815160	2105	-	-	GO:0004252:serine-type endopeptidase activity	-
Pp06_35148-5p	18786162	PRUPE_ppb021184mg	NCBI_Assembly:GCF_000346465.1	NW_006760324.1	-	11857673	11858723	1050	pper01100:Metabolic pathways;pper01110:Biosynthesis of secondary metabolites;pper00460:Cyanoamino acid metabolism;pper00500:Starch and sucrose metabolism;pper00940:Phenylpropanoid biosynthesis	GO:0005975:carbohydrate metabolic process	GO:0004553:hydrolase activity, hydrolyzing O-glycosyl compounds	-

Pp06_35148-5p	18786410	PRUPE_ppa019828mg	NCBI_Assembly:GCF_000346465.1	NW_006760324.1	+	24632050	24634021	1971	pper04075:Plant hormone signal transduction	GO:0006470:protein dephosphorylation;GO:0009062:fatty acid catabolic process;GO:0009640:photomorphogenesis;GO:0009737:response to abscisic acid;GO:0009793:embryo development ending in seed dormancy;GO:0009845:seed germination;GO:0009909:regulation of flower development;GO:0009933:meristem structural organization;GO:0010162:seed dormancy process;GO:0010182:sugar mediated signaling pathway;GO:	GO:0004722:protein serine/threonine phosphatase activity;GO:0046872:metal ion binding	-
Pp06_35148-5p	18786449	PRUPE_ppa017975mg	NCBI_Assembly:GCF_000346465.1	NW_006760324.1	-	20675915	20676247	332	-	-	-	-

Pp06_35148-5p	18786713	PRUPE_ppa008765mg	NCBI_Assembly:GCF_000346465.1	NW_006760324.1	+	23842589	23845231	2642	-	-	GO:0004784:superoxide dismutase activity;GO:0005507:copper ion binding;GO:0046872:metal ion binding	GO:0005739:mitochondrion;GO:0005886:plasma membrane;GO:0009579:thylakoid;GO:0009941:chloroplast envelope
Pp06_35148-5p	18786795	PRUPE_ppa017046mg	NCBI_Assembly:GCF_000346465.1	NW_006760324.1	+	25782827	25784766	1939	-	-	GO:0016773:phosphotransferase activity, alcohol group as acceptor	GO:0005777:peroxisome
Pp06_35148-5p	18786848	PRUPE_ppa009741mg	NCBI_Assembly:GCF_000346465.1	NW_006760324.1	-	6169905	6173394	3489	-	GO:0006897:endocytosis	-	GO:0016020:membrane
Pp06_35148-5p	18786886	PRUPE_ppb020100m1g	NCBI_Assembly:GCF_000346465.1	NW_006760324.1	-	11038850	11038966	116	-	-	-	-
Pp06_35148-5p	18786997	PRUPE_ppa002503mg	NCBI_Assembly:GCF_000346465.1	NW_006760324.1	-	22424369	22428564	4195	-	-	GO:0008168:methyltransferase activity	-
Pp06_35148-5p	18787061	PRUPE_ppa024293mg	NCBI_Assembly:GCF_000346465.1	NW_006760324.1	+	26124208	26126583	2375	-	GO:0006355:regulation of transcription, DNA-templated;GO:0048366:leaf development	GO:0005524:ATP binding;GO:0016818:hydrolase activity, acting on acid anhydrides, in phosphorus-containing anhydrides	GO:0005634:nucleus

Pp06_35148-5p	18787745	PRUPE_ppa009555mg	NCBI_Assembly:GCF_000346465.1	NW_006760334.1	-	170467	175718	5251	pper04140:Regulation of autophagy	-	GO:0004672:protein kinase activity;GO:0005524:ATP binding	-	
Pp06_35148-5p	18788002	PRUPE_ppa009015mg	NCBI_Assembly:GCF_000346465.1	NW_006760384.1	-	63395	65344	1949	-	-	-	-	
Pp06_35148-5p	18788421	PRUPE_ppa013594mg	NCBI_Assembly:GCF_000346465.1	NW_006760385.1	-	9134417	9136724	2307	pper01100:Metabolic pathways;pper00230:Purine metabolism;pper00240:Pyrimidine metabolism;pper03020:RNA polymerase	GO:0006351:transcription, DNA-templated	GO:0003677:DNA binding;GO:0003899:DNA-directed RNA polymerase activity;GO:0008270:zinc ion binding	GO:0005634:nucleus	
Pp06_35148-5p	18788515	PRUPE_ppa007732mg	NCBI_Assembly:GCF_000346465.1	NW_006760385.1	-	28487686	28490400	2714	pper00270:Cysteine and methionine metabolism;pper01100:Metabolic pathways;pper00330:Arginine and proline metabolism	GO:0006557:S-adenosylmethioninamine biosynthetic process;GO:0006597:spermine biosynthetic process;GO:0008295:spermidine biosynthetic process	GO:0004014:adenosylmethionine decarboxylase activity	-	
Pp06_35148-5p	18788677	PRUPE_ppa020405mg	NCBI_Assembly:GCF_000346465.1	NW_006760385.1	-	32317244	32318712	1468	-	GO:0006629:lipid metabolic process;GO:0019761:glucosinolate biosynthetic process	GO:0016298:lipase activity	-	

Pp06_35148-5p	18788699	PRUPE_ppa020455mg	NCBI_Assembly:GCF_000346465.1	NW_006760385.1	+	21500575	21502539	1964	-	-	-	-
Pp06_35148-5p	18788815	PRUPE_ppa017763mg	NCBI_Assembly:GCF_000346465.1	NW_006760385.1	-	36503731	36508004	4273	-	-	GO:0008236: serine-type peptidase activity	-
Pp06_35148-5p	18788870	PRUPE_ppa012353mg	NCBI_Assembly:GCF_000346465.1	NW_006760385.1	+	14335025	14335794	769	-	-	-	-
Pp06_35148-5p	18788919	PRUPE_ppa007034mg	NCBI_Assembly:GCF_000346465.1	NW_006760385.1	+	23184324	23187418	3094	-	-	-	-
Pp06_35148-5p	18788930	PRUPE_ppa021422mg	NCBI_Assembly:GCF_000346465.1	NW_006760385.1	-	15893075	15893584	509	-	-	-	-
Pp06_35148-5p	18788980	PRUPE_ppa013171mg	NCBI_Assembly:GCF_000346465.1	NW_006760385.1	+	9473550	9474505	955	-	-	GO:0003700: sequence-specific DNA binding transcription factor activity;GO:0008270:zinc ion binding;GO:0043565:sequence-specific DNA binding	-
Pp06_35148-5p	18789035	PRUPE_ppa011320mg	NCBI_Assembly:GCF_000346465.1	NW_006760385.1	+	728594	733658	5064	-	GO:0006621: protein retention in ER lumen;GO:0015031:protein transport	GO:0046923: ER retention sequence binding	GO:0005789: endoplasmic reticulum membrane;GO:0016021:integral component of membrane

Pp06_35148-5p	18789050	PRUPE_ppa011168mg	NCBI_Assembly:GCF_000346465.1	NW_006760385.1	+	38343628	38345992	2364	pper03013:RNA transport;pper03008:Ribosome biogenesis in eukaryotes	GO:0006886:intracellular protein transport;GO:0006913:nucleocytoplasmic transport;GO:0007264:small GTPase mediated signal transduction	GO:0003924:GTPase activity;GO:0005525:GTP binding	GO:0005622:intracellular
Pp06_35148-5p	18789140	PRUPE_ppa010793mg	NCBI_Assembly:GCF_000346465.1	NW_006760385.1	+	37535807	37539046	3239	-	GO:0006661:phosphatidylinositol biosynthetic process;GO:0034508:centromere complex assembly	-	GO:0000776:kinetochore
Pp06_35148-5p	18789976	PRUPE_ppa001373mg	NCBI_Assembly:GCF_000346465.1	NW_006760385.1	+	25652971	25658468	5497	-	-	GO:0015079:potassium ion transmembrane transporter activity	GO:0009507:chloroplast;GO:0016021:integral component of membrane
Pp06_35148-5p	18790207	PRUPE_ppa000142mg	NCBI_Assembly:GCF_000346465.1	NW_006760385.1	+	11119812	11131187	11375	-	-	-	GO:0034399:nuclear periphery
Pp06_35148-5p	18790278	PRUPE_ppa015668mg	NCBI_Assembly:GCF_000346465.1	NW_006760385.1	+	25393885	25396439	2554	-	-	GO:0003700:sequence-specific DNA binding transcription factor activity	-
Pp06_35148-5p	18790393	PRUPE_ppb023497mg	NCBI_Assembly:GCF_000346465.1	NW_006760385.1	+	9400148	9400600	452	-	-	GO:0005215:transporter activity	GO:0016020:membrane
Pp06_35148-5p	18790511	PRUPE_ppa007281mg	NCBI_Assembly:GCF_000346465.1	NW_006760385.1	+	27632811	27635985	3174	-	-	-	-

Pp06_35148-5p	18790523	PRUPE_ppa013222mg	NCBI_Assembly:GCF_000346465.1	NW_006760385.1	-	2968157	2970226	2069	pper03010:Ribosome	GO:0006412:translation	GO:0003735:structural constituent of ribosome	GO:0005840:ribosome
Pp06_35148-5p	18790610	PRUPE_ppa015144mg	NCBI_Assembly:GCF_000346465.1	NW_006760385.1	+	25568443	25570095	1652	-	GO:0009966:regulation of signal transduction	GO:0004674:protein serine/threonine kinase activity;GO:0005524:ATP binding	-
Pp06_35148-5p	18790629	PRUPE_ppa006302mg	NCBI_Assembly:GCF_000346465.1	NW_006760385.1	-	27762710	27766402	3692	-	-	GO:0015095:magnesium ion transmembrane transporter activity	GO:0016020:membrane
Pp06_35148-5p	18790660	PRUPE_ppa020377mg	NCBI_Assembly:GCF_000346465.1	NW_006760385.1	-	18621268	18624310	3042	-	-	GO:0004674:protein serine/threonine kinase activity;GO:0005524:ATP binding	GO:0016021:integral component of membrane
Pp06_35148-5p	18790661	PRUPE_ppa012907mg	NCBI_Assembly:GCF_000346465.1	NW_006760385.1	-	1660475	1662387	1912	pper04626:Plant-pathogen interaction	-	GO:0005509:calcium ion binding	-
Pp06_35148-5p	18790666	PRUPE_ppa009249mg	NCBI_Assembly:GCF_000346465.1	NW_006760385.1	-	17803311	17806734	3423	-	-	-	-

Pp06_35148-5p	18790746	PRUPE_ppa001426mg	NCBI_Assembly:GCF_000346465.1	NW_006760385.1	+	40668439	40673721	5282	-	GO:0000226:microtubule cytoskeleton organization;GO:0000911:cytokinesis by cell plate formation;GO:0006306:DNA methylation;GO:0006346:methylation-dependent chromatin silencing;GO:0007267:cell-cell signaling;GO:0009616:virus induced gene silencing;GO:0009909:regulation of flower development;GO:0010267:production of ta-siRNAs involved in RNA interference;GO:0031048:chromatin silencing by small RNA;GO:0035196:product [illegible]	GO:0004672:protein kinase activity;GO:0005524:ATP binding	-
Pp06_35148-5p	18790747	PRUPE_ppa001845mg	NCBI_Assembly:GCF_000346465.1	NW_006760385.1	+	9912782	9917014	4232	pper01100:Metabolic pathways;pper00500:Starch and sucrose metabolism	GO:0005985:sucrose metabolic process;GO:0009058:biosynthetic process	GO:0016157:sucrose synthase activity	-

Pp06_35148-5p	18791051	PRUPE_ppa020774mg	NCBI_Assembly:GCF_000346465.1	NW_006760385.1	-	13433132	13439500	6368	-	-	-	-
Pp06_35148-5p	18791088	PRUPE_ppa023100mg	NCBI_Assembly:GCF_000346465.1	NW_006760385.1	-	28064302	28068880	4578	-	-	GO:0004497:monooxygenase activity;GO:0005506:iron ion binding;GO:0016705:oxidoreductase activity, acting on paired donors, with incorporation or reduction of molecular oxygen;GO:0020037:heme binding	-
Pp06_35148-5p	18791446	PRUPE_ppa019235mg	NCBI_Assembly:GCF_000346465.1	NW_006760385.1	+	840902	842029	1127	-	-	GO:0043531:ADP binding	-
Pp06_35148-5p	18791510	PRUPE_ppa000009mg	NCBI_Assembly:GCF_000346465.1	NW_006760385.1	-	46082352	46098486	16134	pper04120:Ubiquitin mediated proteolysis	-	GO:0004842:ubiquitin-protein transferase activity;GO:0016874:ligase activity	-

Pp06_35148-5p	18791546	PRUPE_ppa004343mg	NCBI_Assembly:GCF_000346465.1	NW_006760385.1	-	24625651	24627353	1702	-	-	GO:0004497:monooxygenase activity;GO:0005506:iron ion binding;GO:0016705:oxidoreductase activity, acting on paired donors, with incorporation or reduction of molecular oxygen;GO:0020037:heme binding	-
Pp06_35148-5p	18791585	PRUPE_ppa006980mg	NCBI_Assembly:GCF_000346465.1	NW_006760385.1	+	34293156	34299145	5989	-	GO:0006470:protein dephosphorylation	GO:0004722:protein serine/threonine phosphatase activity;GO:0046872:metal ion binding	-
Pp06_35148-5p	18791595	PRUPE_ppa005733mg	NCBI_Assembly:GCF_000346465.1	NW_006760385.1	+	35515951	35519704	3753	pper04120:Ubiquitin mediated proteolysis;pper03420:Nucleotide excision repair	-	-	-

Pp06_35148-5p	18791608	PRUPE_ppa000805mg	NCBI_Assembly:GCF_000346465.1	NW_006760385.1	-	36873652	36877947	4295	-	GO:0009832:plant-type cell wall biogenesis;GO:0016049:cell growth;GO:0030243:cellulose metabolic process	-	-
Pp06_35148-5p	18791625	PRUPE_ppa009348mg	NCBI_Assembly:GCF_000346465.1	NW_006760385.1	+	8553036	8556050	3014	-	GO:0006412:translation;GO:0019243:methylglyoxal catabolic process to D-lactate	GO:0003735:structural constituent of ribosome;GO:0019843:rRNA binding	GO:0005840:ribosome
Pp06_35148-5p	18791701	PRUPE_ppa020409mg	NCBI_Assembly:GCF_000346465.1	NW_006760385.1	-	25705585	25706703	1118	-	-	GO:0008270:zinc ion binding	-
Pp06_35148-5p	18791712	PRUPE_ppa001193mg	NCBI_Assembly:GCF_000346465.1	NW_006760385.1	+	34773903	34779308	5405	-	-	GO:0008237:metallopeptidase activity;GO:0008270:zinc ion binding	-
Pp06_35148-5p	18791743	PRUPE_ppa007500mg	NCBI_Assembly:GCF_000346465.1	NW_006760385.1	-	37561528	37565095	3567	-	-	-	-
Pp06_35148-5p	18791760	PRUPE_ppa006790mg	NCBI_Assembly:GCF_000346465.1	NW_006760385.1	+	33956580	33958440	1860	-	-	-	-
Pp06_35148-5p	18791826	PRUPE_ppa002791mg	NCBI_Assembly:GCF_000346465.1	NW_006760385.1	+	44758200	44760263	2063	-	-	-	-
Pp06_35148-5p	18791830	PRUPE_ppa000186mg	NCBI_Assembly:GCF_000346465.1	NW_006760385.1	+	28673586	28678668	5082	-	GO:0002100:tRNA wobble adenosine to inosine editing	GO:0008251:tRNA-specific adenosine deaminase activity;GO:0008270:zinc ion binding	GO:0005886:plasma membrane

Pp06_35148-5p	18791874	PRUPE_ppa011277mg	NCBI_Assembly:GCF_000346465.1	NW_006760385.1	+	32812568	32814040	1472	pper01100:Metabolic pathways;pper04145:Phagosome;pper00190:Oxidative phosphorylation	GO:0015991:ATP hydrolysis coupled proton transport	GO:0015078:hydrogen ion transmembrane transporter activity	GO:0016021:integral component of membrane;GO:0033179:proton-transporting V-type ATPase, V0 domain
Pp06_35148-5p	18791907	PRUPE_ppa009485mg	NCBI_Assembly:GCF_000346465.1	NW_006760385.1	-	42098111	42099766	1655	-	-	GO:0003824:catalytic activity;GO:0050662:coenzyme binding	GO:0005886:plasma membrane
Pp06_35148-5p	18792020	PRUPE_ppa010764mg	NCBI_Assembly:GCF_000346465.1	NW_006760385.1	-	4016221	4019411	3190	-	GO:0016568:chromatin modification	GO:0008270:zinc ion binding	GO:0005634:nucleus
Pp06_35148-5p	18792072	PRUPE_ppa003886mg	NCBI_Assembly:GCF_000346465.1	NW_006760385.1	-	46691428	46697914	6486	pper01100:Metabolic pathways;pper00750:Vitamin B6 metabolism	GO:0008615:pyridoxine biosynthetic process;GO:0019761:glucosinolate biosynthetic process;GO:0042817:pyridoxal metabolic process	GO:0004733:pyridoxamine-phosphate oxidase activity;GO:0010181:FMN binding	GO:0005829:cytosol
Pp06_35148-5p	18792308	PRUPE_ppa018985mg	NCBI_Assembly:GCF_000346465.1	NW_006760385.1	-	14359514	14361874	2360	-	-	-	-

Pp06_35148-5p	18792366	PRUPE_ppa000255mg	NCBI_Assembly:GCF_000346465.1	NW_006760385.1	+	27273141	27281372	8231	-	GO:0006355:regulation of transcription, DNA-templated;GO:0009630:gravitropism;GO:0030244:cellulose biosynthetic process;GO:0048193:Golgi vesicle transport	GO:0005509:calcium ion binding	GO:0005829:cytosol
Pp06_35148-5p	18792405	PRUPE_ppa002255mg	NCBI_Assembly:GCF_000346465.1	NW_006760385.1	-	40342720	40348397	5677	pper01100:Metabolic pathways;pper00071:Fatty acid degradation;pper00061:Fatty acid biosynthesis;pper01212:Fatty acid metabolism;pper04146:Peroxisome	-	GO:0003824:catalytic activity	GO:0016020:membrane
Pp06_35148-5p	18792629	PRUPE_ppa024372mg	NCBI_Assembly:GCF_000346465.1	NW_006760385.1	+	9411144	9412907	1763	-	-	GO:0005215:transporter activity	GO:0016020:membrane
Pp06_35148-5p	18792723	PRUPE_ppa010924mg	NCBI_Assembly:GCF_000346465.1	NW_006760385.1	+	33946167	33948377	2210	pper03060:Protein export	GO:0006465:signal peptide processing	GO:0008233:peptidase activity	GO:0005787:signal peptidase complex;GO:0016021:integral component of membrane

Pp06_35148-5p	18792765	PRUPE_ppa018001mg	NCBI_Assembly:GCF_000346465.1	NW_006760385.1	+	19038888	19040659	1771	-	-	GO:0003676:nucleic acid binding;GO:0004523:RNA-DNA hybrid ribonuclease activity	-
Pp06_35148-5p	18792792	PRUPE_ppa020612mg	NCBI_Assembly:GCF_000346465.1	NW_006760385.1	+	38709527	38712125	2598	-	-	GO:0003676:nucleic acid binding	-
