# Supplementary material for: Identification and characterization of Prunus persica miRNAs in response to UVB radiation in greenhouse through high-throughput sequencing
Source: BMC Genomics. 2017 Dec 2;18:938. doi: 10.1186/s12864-017-4347-5 (PMC5712094; doi:10.1186/s12864-017-4347-5)
Supplement: Supplementary file 5 — Primers for qRT-PCR verification of miRNAs. (PDF 17 kb) [file 12864_2017_4347_MOESM5_ESM.pdf]

Name	Forward primer	Reverse primer
miR1511	CGTGGTATCAGAGTCATGTTA	CGUGGUAUCAGAGUCAUGUUA
miR156a	TGACAGAAGAAAGAGAGCAC	UGACAGAAGAAAGAGAGCAC
miR159	ATCTCGAGGGAAGTTAGGTTT	AUCUCGAGGGAAGUUAGGUUU
miR160a	CCTGCTCCCTGTATGCCAAA	UGCCUGGCUCCCUGUAUGCCA
miR166a	CTTACTTCGGACCAGGCTAA	CCCCUUACUUCGGACCAGGCU
miR171c	GTTTGAGCCGTGCCAATATC	UGAUUGAGCCGUGCCAAUAUC
miR171d-3p	GCCGAGCCGAATCAATATCACTC	CGAGCCGAAUCAAUAUCACUC
miR319a	CGTTGGACTGAAGGGAGCT	UUGGACUGAAGGGAGCUCCC
miR319b	TAGCTGCCGAGTCATTCATCCA	UAGCUGCCGAGUCAUUCAUCCA
miR3627-3p	TGGTGTCATCCCTCCTGTGA	UGGUGUCAUCCCUCCUGUGACC
miR3627-5p	TCGCAGGAGAGATGGCACTGTCA	UCGCAGGAGAGAUGGCACUGUC
miR393a	CCAAAGGGATCGCATTGAC	CAUCCAAAGGGAUCGCAUUGA
miR395d	CTCAAGGGGGTTTGTGAAGT	CUCAAGGGGGUUUGUGAAGU
miR395e	TGAAGTGTTTGGGGGAACTC	CUGAAGUGUUUGGGGGAACUC
miR397	TCATTGAGTGCAGCGTTGATG	UCAUUGAGUGCAGCGUUGAUG
miR398a-3p	AACGCTTGTCGCCCCTGAAA	UGUGUUCUCAGGUCGCCCCUG
miR398a-5p	AGCGACCTGGGATCACATG	GGAGCGACCUGGGAUCACAUG
miR399a	CCGTTGAGAGGAAACCGCAAA	UUCCCGUUGAGAGGAAACCGC
miR399b	TCTGCCAAAGGAGAATTGCC	UCUGCCAAAGGAGAAUUGCCC
miR402	GCTTCGAGGCCTATTAAACC	UUCGAGGCCUAUUAAACCUCUG
miR5059	TCTGGGCAGCACCACCAAA	CGGCCUGGGCAGCACCACCA
miR5072	GATATCGATTCCCCAGCGG	CGAUUCCCCAGCGGAGUCGCCA
miR6260	TGGAGTGAGAGAATGGGAGGT	UGGAGUGAGAGAAUGGGAGGU
miR6263	AAGTGGACAAAAGGGGAGTG	AAGUGGACAAAAGGGGAGUGG
miR7122b-5p	GCCTTATACAATGAAATCACGGTCG	UUAUACAAUGAAAUCACGGUCG
miR8133-3p	TAACTTCCGAACGTCCGCATA	UAACUUCCGAACGUCCGCAUA
Pp03_22312-3p	TTCCATCTTCCTGTGACATGA	UUCCAUCUUCCUGUGACAUGA
Pp03_22312-5p	ATGTCGCAGGAGAGATGGCA	AUGUCGCAGGAGAGAUGGCACGGGA
Pp03-19842-3p	GAATTTCCAAGCCAGAACTCT	AAUUUCCAAGCCAGAACUCUGAUCUU
Pp05-28899-3p	GCAGAGAGAATGAAAGTCGTAGTGTA	AGAGAGAAUGAAAGUCGUAGUGUA
Pp06_35148-3p	TCATATTTGTTCCACTGCCTGCGG	UCAUAUUUGUUCCACUGCCUGCGG

Pp06-35148-5p	ACAGGCGGTGGATCAAATATGAAT	ACAGGCGGUGGAUCAAAUAUGAAU
