# Supplementary material for: Identification and characterization of Prunus persica miRNAs in response to UVB radiation in greenhouse through high-throughput sequencing
Source: BMC Genomics. 2017 Dec 2;18:938. doi: 10.1186/s12864-017-4347-5 (PMC5712094; doi:10.1186/s12864-017-4347-5)
Supplement: Supplementary file 6 — The names, Genbank IDs, involved pathways of verified target genes and their primers for qRT-PCR tests. (PDF 79 kb) [file 12864_2017_4347_MOESM6_ESM.pdf]

Target gene	Description	Pathway	Genbank	qPCR forward primer	qPCR reverse primer
beta-actin	beta-actin	reference	-	GTTATTCTTCATCGGCGTCTTCG	CTTCACCATTCCAGTTCCATTGTC
SCL	Scarecrow-like protein	chlorophyll	XP_020419504.1	CGGCTCTACAGACACAAC	AGACTCAGGCAAGACACT
PORC	Pchlide oxidoreductase C	chlorophyll	OAP13341.1	CATCAAGGAGGCTCATCAT	CGATCATGGACGAACTGT
CHLG	Chlorophyll synthase	chlorophyll	XM_020567187.1	TTGGCGCCGATTTCTGTTTC	TACCAGGGGAGGCCAAGTTA
RCCR	Red chlorophyll catabolite reductase	chlorophyll	XM_007202364.2	TGTCCTCCACCCTGACTACC	GGAGACACAACACTGCGGAT
CBR	Chlorophyll a-b binding protein	chlorophyll	XM_007222653.2	GGCTTTGATCCCCTTGGTCT	GCCCACCCAATCAAAACCAG
SUS4	Sucrose synthase 4	sucrose	NM_001339091.1	CAAGAGATTGCAGGAACGAA	CTGCTCCAGGAGACACGATA
NIV8	Neutral invertase 8	sucrose	-	TCATAGAATCACGGTGGGAA	AAGACCCTCCGTTGTGGTAG
SPS	Sucrose phosphate synthase	sucrose	ABV32551.1	AGGACGGTGATATGGATGGT	CAAGAATCATAGGCTTGCGA
SUT2	Sucrose transporter 2	sucrose	AHN92214.2	TTTGGAGCCATTTAATGCAA	CGAAGGAAATCGAGAAGAGG
SUT4	Sucrose transporter 4	sucrose	ALX37955.1	CAAGAGATTGCAGGAACGAA	CTGCTCCAGGAGACACGATA
TMT2	Tonoplast monosaccharide transporter	sucrose	-	GTGGAGAAGGAGCTGGTAGC	CCCTCCTGGTGCAAATAAAT
S6PDH	Sorbitol -6- phosphate dehydrogenase	sorbitol	EF576940.2	TCCAGCGTGAATCTCTCGTC	GGTGTGCCCCTCTCAATGTT
NAD-SDH	NAD-sorbitol dehydrogenase	sorbitol	AB025969.1	GCTTTGACTGCGTGGGTTTT	TCCGAAACGGTGCGTTATGA
NAD-SDH2	NAD-sorbitol dehydrogenase 2	sorbitol	AB025969.1	TGCTGGGATCGTTGACGAAG	CCCTTGCACTGCTCACATCT
NADP-SDH	NADP-sorbitol dehydrogenase	sorbitol	AF414988.1	GATCCAGGTTCTCGTGCGAT	GTGCCCCTCTCAATGTTCCA
SOT	Sorbitol transporter	sorbitol	AY924379.1	CCTCCGTTATTCTCGCCGTT	CTGAACAACGTCTTCGGTGC
HEX6	Hexose carrier protein	hexose	XM_007219552.2	ATCCAAAGCACCAACGACCA	TCTGCTTGGACATCTTCGCA
HET	Hexose transporter	hexose	AF367455.1	TTCTTGCCCTCCTATGTGCG	AGGCAGGAACACGTAAACGA
PFP-β	Pyrophosphate--fructose 6-phosphate 1-phosphotransferase subunit beta	hexose	XM_007215454.2	AGCAGCGCTCTAAAACCCAT	AAGGGAGAGGAAGCGAAACG
PFP-α	Pyrophosphate--fructose 6-phosphate 1-phosphotransferase subunit alpha	hexose	XM_020563552.1	TCACAACCCCAAAAGCACCT	TGGGCAGCATCGGTATTTGA
FLN1	Fructokinase-like 1	hexose	XM_007204994.2	AAGCTAAACCCACCCAGAGC	GCTTTTTCCGAACGCTTGGT
FLN2	Fructokinase-like 2	hexose	XM_007222858.2	CCCAATAGCCCGTCACTGAA	ATGCCGCTTTCCTACGAGTT
FBpase	Fructose-1,6-bisphosphatase, chloroplastic	hexose	XM_007199828.2	TGGGCGATGATCTTGTGCTT	CCGGCAGCAAAACTCGTATG
PFK	Phosphofructokinase	hexose	KC700019.1	AGGAGCAGGGCAGGAATATG	TTTCAGTCAGCCATAGGCCA
ppa025787mg	polygalacturonase			GCCACTGCGGTTACATACA	CTGAATACCACATTGCGAAG
ppa007623mg	Glucuronokinase			ACGCAGGAGTTTGAGTTCG	TGCCAAGGCTGAAGGAGAT
ppa016917mg	Major pollen allergen-like protein			AAGTGACAGTGCGTTGGA	ACCTGAAAGACCAGCAAGC
ppa021860mg	Major pollen allergen-like protein			CGCTTTGTTCTGTGCCTT	GTTGTTGCTCATCTTCCCA

ppa007934mg	Succinate dehydrogenase (ubiquinone) Iron-sulfur subunit	TGGATAAGTGACAGTCGTGAC	ACTCAAGCACCAACTCCTAAC

the primer of NIV8 and TMT2 is from Sornkanok V. [64]
