# Supplementary figures and images for: Identification and characterization of Prunus persica miRNAs in response to UVB radiation in greenhouse through high-throughput sequencing
Source: BMC Genomics. 2017 Dec 2;18:938. doi: 10.1186/s12864-017-4347-5 (PMC5712094; doi:10.1186/s12864-017-4347-5)

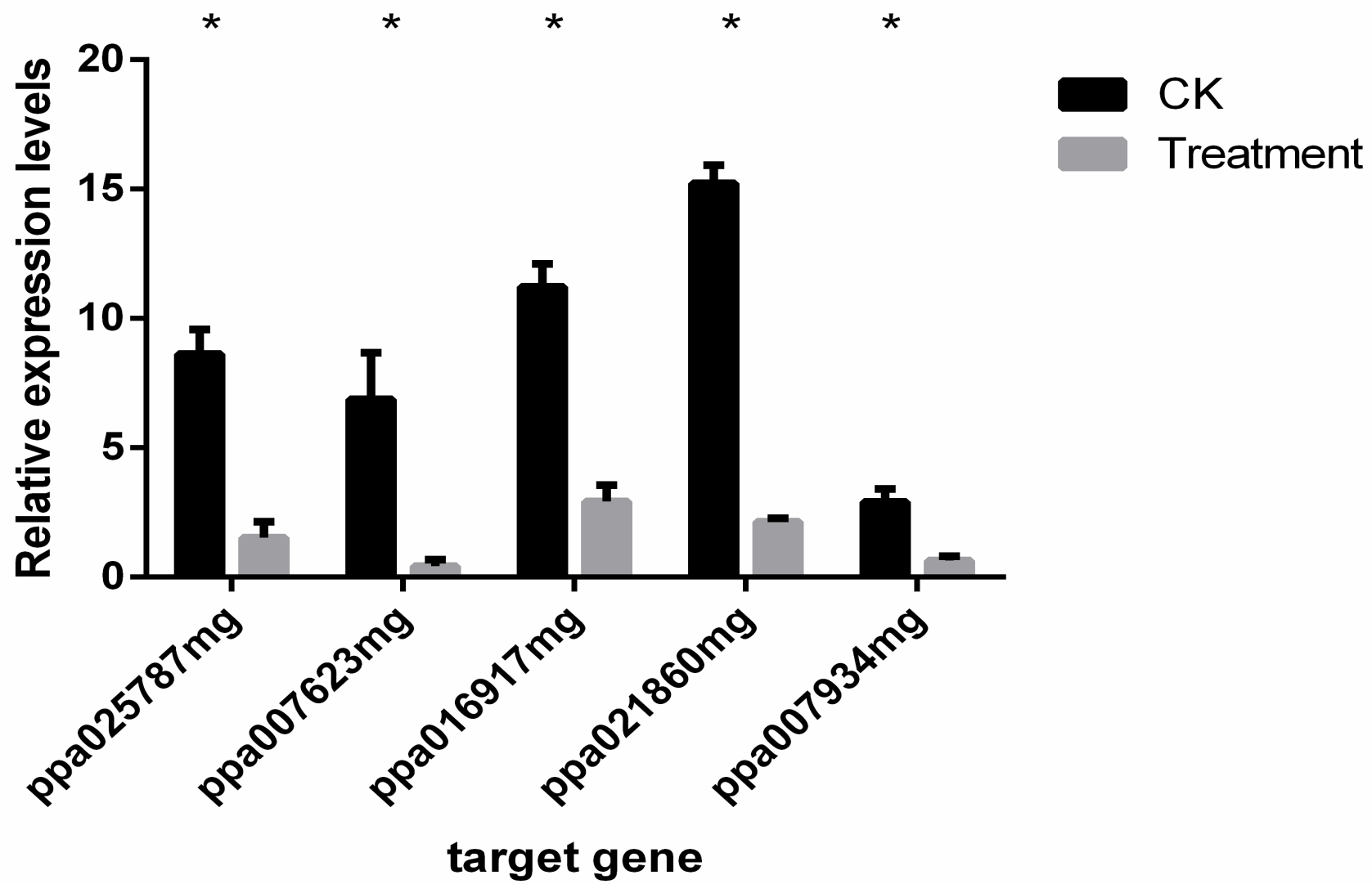

Supplement: Supplementary file 7 — qRT-PCR analysis of five target genes predicted for miR3627-5p. Beta-actin was the internal control. Each experiment was performed with three biological replicates. (PDF 66 kb) [file 12864_2017_4347_MOESM7_ESM.pdf]
